# Pd-catalyzed regiodivergent arylation of cyclic allylboronates

**DOI:** 10.1039/d5sc07577g

**Published:** 2025-10-24

**Authors:** Cheng Zhang, Baptiste Leforestier, Céline Besnard, Clément Mazet

**Affiliations:** a Department of Organic Chemistry, University of Geneva 30 Quai Ernest Ansermet 1211 Geneva Switzerland clement.mazet@unige.ch; b Laboratory of Crystallography, University of Geneva 24 Quai Ernest Ansermet 1211 Geneva Switzerland

## Abstract

Two complementary regiodivergent Pd-catalyzed arylations of the lithium salts of 6-membered cyclic allylboronates are reported. The two systems provide access to products featuring a polysubstituted alkene and a boronic ester and are both compatible with a broad range of aryl bromides or heteroaryl bromides. When a (*P*,*N*) ligand is employed, a highly C1-selective arylation is favored. With the use of a Buchwald-type monophosphine ligand, a highly C3-selective arylation is favored. DFT calculations served to establish that a solvated dimer of the substrate is involved rather than a monomeric allylboronate. For the C3-arylation pathway, regioselectivity is governed mostly by steric factors during binding of the substrate to the metal center. A Curtin–Hammett equilibrium is crucial in determining C1 selectivity for the second catalytic system. A pivotal isomerization between two η^2^-alkene-Pd(ii) intermediates precedes ring-opening transition states and irreversible reductive elimination.

## Abstract 2

Two complementary regiodivergent Pd-catalyzed arylations of the lithium salts of 6-membered cyclic allylboronates are reported.

## Graphical abstract

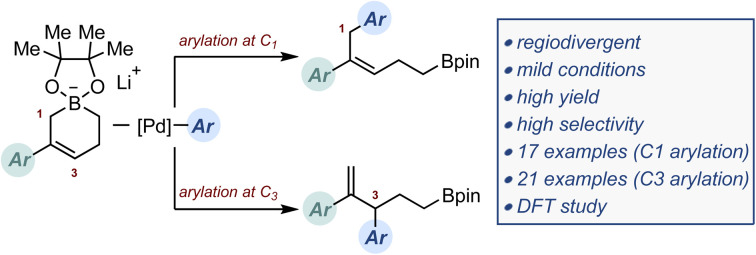

## Introduction

**Fig. 1:**
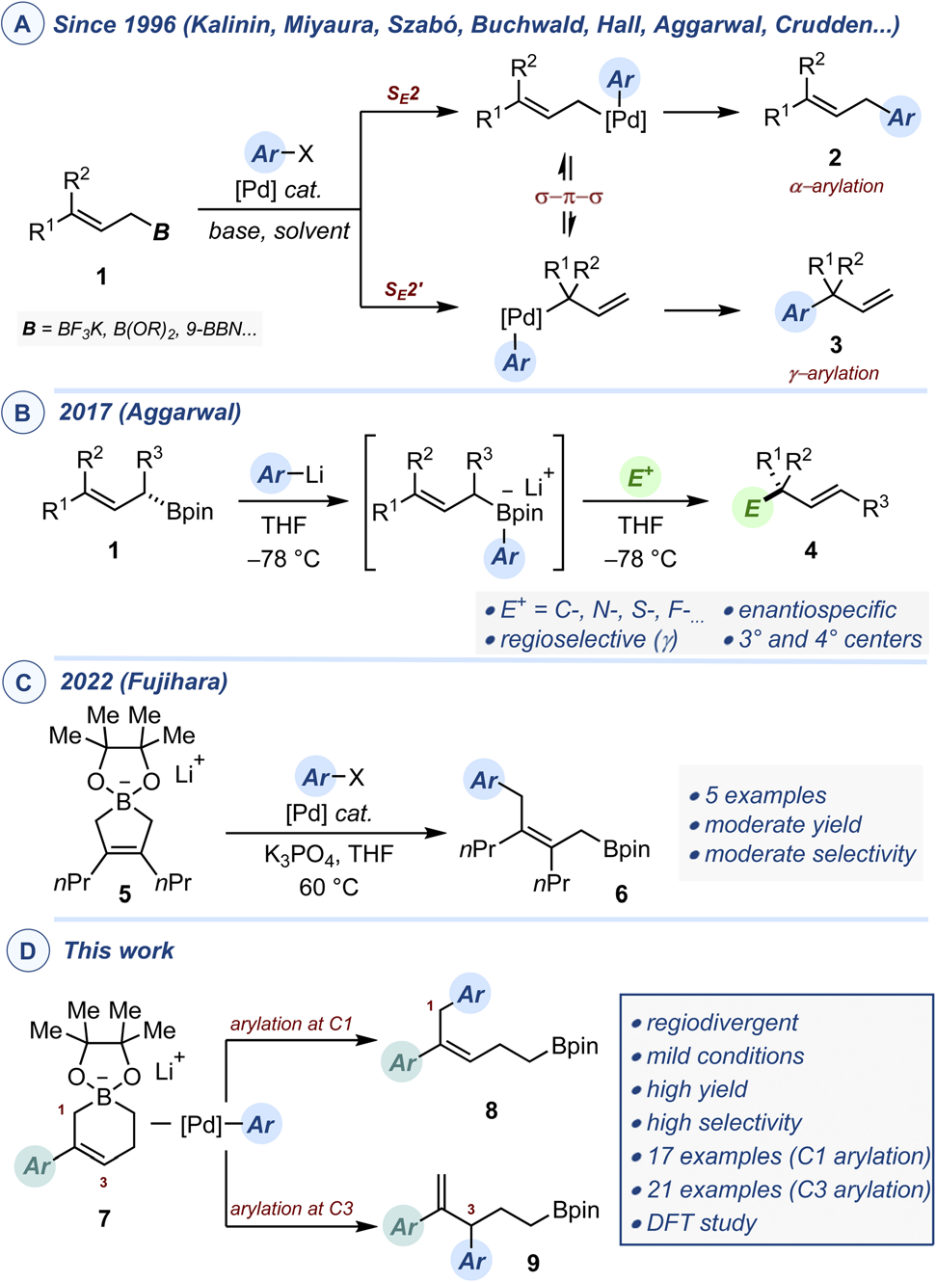
(A) Pd-catalyzed regioselective arylation of allylboron reagents. (B) Stereospecific functionalization of allylboronates with electrophiles. (C) Pd-catalyzed arylation of 5-membered allylboronates. (D) Regiodivergent Pd-catalyzed arylation of 6-membered allylboronates.

Transition metal-catalyzed cross-coupling reactions have revolutionized the practice of organic synthesis in both academic circles and industrial settings.^1^ Their matricial nature, operational simplicity, robustness and broad scope have accelerated the pace of discovery in agrochemicals, material sciences and pharmaceutical research. Despite repeated advances over the last decades, the need to identify novel substrate classes and/or reactivity modes to explore uncharted chemical spaces and access novel classes of molecules remains relevant and topical. In this context, the regiocontrolled Pd-catalyzed arylation of allylboron reagents provides an opportunity to access selectively a variety of linear allylation products (2, α-selectivity) or branched allylation products (3, γ-selectivity), both of which are highly prevalent motifs in natural products and bioactive compounds, as well as primary building blocks for synthesis (Fig. 1A).^2,3^ While Miyaura^3*d*^ and Szabo^3*f*^ have independently developed conditions to generate the branched products with high selectivity, Kalinin^3*a*^ and Organ^3*h*^ have reported complementary methods to obtain α-selective arylation. Studies by Aggarwal, Crudden,^3*i*,*j*^ Hall^3*m*,*n*^ and Morken^3*o*^ demonstrated that cross-coupling using enantioenriched secondary allylboron reagents could be achieved with exquisite enantiospecificity. Catalytic systems using 3,3-disubstituted allylboron reagents with γ-selectivity offering access to quaternary centers remain uncommon to date, with the sole contributions of Buchwald^3*k*^ and Morken.^3*o*^ Equally unusual are enantioselective couplings between allylborons and aryl electrophiles. Such reactions have been accomplished intermolecularly by Miyaura^3*e*–*g*^ and intramolecularly by Morken^3*l*^ to generate tertiary stereocenters with high enantioselectivity. Mechanistically, Suzuki–Miyaura reactions begin with oxidative addition of a Pd(0) complex with the aryl halide or pseudo-halide, followed by transmetallation to an *in situ* generated boronate and the product is released by a final reductive elimination. With allylboron reagents acting as nucleophiles, regioselectivity is controlled by directing the transmetallation *via* either a S_E_2 pathway (α-selectivity) or a S_E_2′ pathway (γ-selectivity). Potential equilibration of Pd-allyl intermediates *via* π–σ–π isomerization prior to reductive elimination accentuates the difficulty in achieving regiocontrol. As an elegant complement to the Pd-catalyzed arylation cross-coupling, Aggarwal showed that addition of an arylithium converted weakly nucleophilic allylboron esters into potent nucleophiles (Fig. 1B).^4^ These were reacted *in situ* with a variety of C-, S-, N- and F-centered electrophiles with excellent regioselectivity and stereospecificity to afford the corresponding allyl derivatives (4) featuring either tertiary or quaternary stereocenters. Recently, the Fujihara group reported the Cu-catalyzed synthesis of 5-membered cyclic allylboronates (5) from 1,3-dienes and bis-(pinacolato)diboron (B_2_pin_2_).^5^ These well-defined crystalline alkali salts constitute a rare case of isolable highly nucleophilic cyclic allylboronates.^6^ When C_2_-symmetric derivatives were subjected to prototypical conditions for Pd-catalyzed Suzuki–Miyaura reaction, 2,3,3-trisusbtituted allylborons (6) were obtained in moderate to practical yield (Fig. 1C). Our own group recently disclosed a Cu-catalyzed borylation of unactivated vinyl-cyclopropanes that yields an array of non-symmetrical 6-membered cyclic allylboronates (7).^7^ Having explored the reactivity of the lithium salts of these allylboronates towards a variety of electrophilic reagents, we wished to investigate whether the development of catalytic regiodivergent arylation was possible (Fig. 1D). If successful, this could form the basis for a modular synthesis of small-molecule building blocks (8 and 9) featuring a polysubstituted alkene and a boronic ester, two of the most flexible functionalities, allowing multiple post-catalytic derivatizations. We also recognized that the C3-selective arylation process could potentially be amenable to enantioselective catalysis.

## Results and discussion

### Reaction development

Our investigations commenced with the evaluation of a set of representative ligand structures (L1–L12) in toluene using standard palladium precursors (Table 1). Initially, 7a and bromobenzene 10a were selected as cross-coupling partners. As the reactions used preformed borate salts, no base was added. While no reaction occurred when *rac*-Binap (L1) was tested at room temperature, a mixture of inseparable C1- and C3-arylation products 8a and 9a was obtained in a 1 : 1.5 ratio at 80 °C with a promising conversion of 64% (entries 1–2). The evaluation of other bisphosphine ligands (L2–L3) and of a (*N*,*N*) ligand (L4) did not provide better results (entries 3–5). By contrast, despite a low conversion, a phenyloxazoline derivative (L5) led to a much-improved regioselectivity (rr_8/9_ = 10 : 1; entry 6). Gratifyingly, L6, a (*P*,*N*) ligand we recently employed in a Ni-catalyzed Kumada–Corriu cross-coupling process,^8^ afforded almost exclusively the C1-arylation product 8a (rr_8/9_ > 20 : 1; entry 7). Consistent with previous observations, no reaction took place at lower temperature (entry 8). A series of Buchwald-type dialkyl-biaryl monophosphines was surveyed next in combination with [(nbd)Pd(ma)] (Pd2) (entries 9–13).^9,10^ While, they all gave satisfactory levels of reactivity, none led to a significant improvement in regioselectivity, either in favor of the C1- or C3-arylation product. We found that using the commercially available palladium G3-(4-(*N*,*N*-dimethyl-amino)phenyl)-di-*tert*-butyl-phosphine precatalyst (noted Pd3) at room temperature afforded the C3-arylation product (9a) in 81% conversion with markedly improved regioselectivity (rr_8/9_ 1 : 15) (entries 14–17). Attempts to further improve this result by evaluating other solvents were not met with success (entries 18–20). As a side note, during our optimization campaign, we observed ring-opened alkenylboranes 11a, 12a and 13a in proportions ranging from 5% to 30% in several reactions. In summary, the two complementary protocols developed lead to high selectivity levels in favor of 8a (rr_8/9_ > 20 : 1) and 9a (rr_8/9_ = 1 : 15) and enable each arylation product to be obtained in pure form after alkaline oxidation and purification of the corresponding alcohol by column chromatography (see SI).

**Table 1:** Reaction optimization^a^

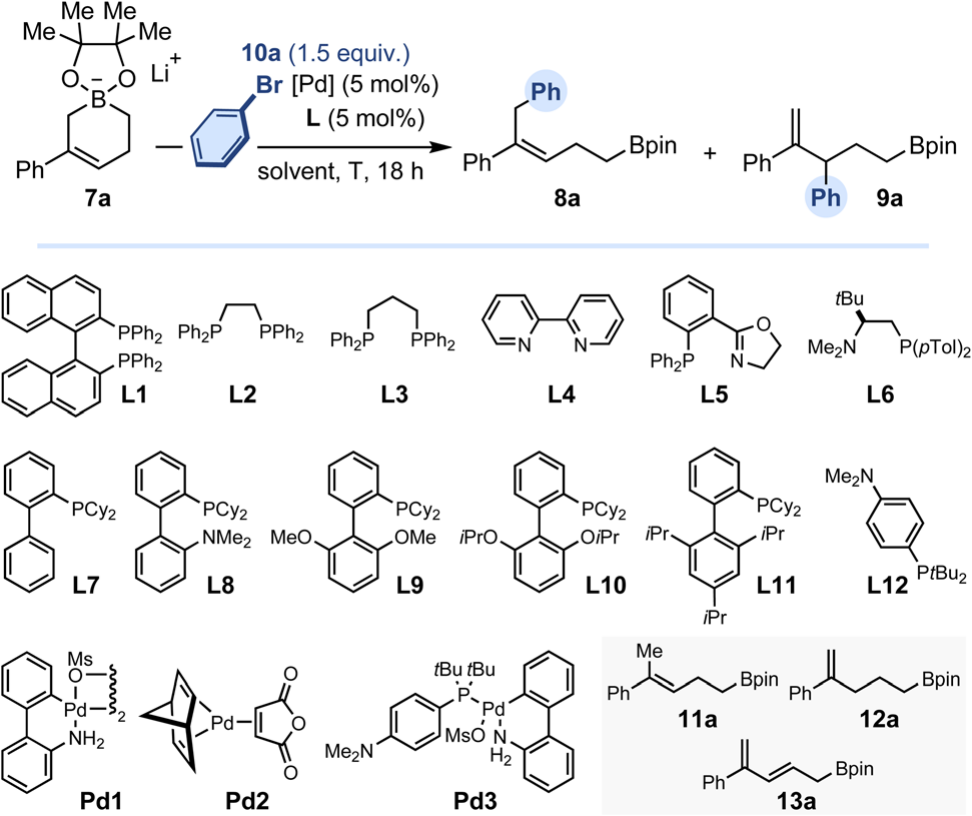					
Entry	L/[Pd]	Solvent	*T* (°C)	Conv^b^. 8a + 9a (%)	rr_8/9_^b^
1	L1/Pd1	Toluene	25	<5	nd
2	L1/Pd1	Toluene	80	64	1 : 1.5
3	L2/Pd1	Toluene	80	21	1.3 : 1
4	L3/Pd1	Toluene	80	<5	nd
5	L4/Pd1	Toluene	80	13	2.3 : 1
6	L5/Pd1	Toluene	80	43	10 : 1
7	L6/Pd1	Toluene	80	72	>20 : 1
8	L6/Pd1	Toluene	25	<5	nd
9	L7/Pd2	Toluene	80	77	1 : 1.1
10	L8/Pd2	Toluene	80	71	1 : 1.8
11	L9/Pd2	Toluene	80	75	1.5 : 1
12	L10/Pd2	Toluene	80	65	2.4 : 1
13	L11/Pd2	Toluene	80	64	1.8 : 1
14	L12/Pd2	Toluene	80	58	1 : 1.2
15	Pd3	Toluene	80	68	1 : 2.8
16	Pd3	Toluene	40	70	1 : 6.0
17	Pd3	Toluene	25	81	1 : 15
18	Pd3	Dioxane	25	61	1 : 3.4
19	Pd3	DME	25	70	1 : 2.0
20	Pd3	THF	25	71	1 : 1.9

a Reactions conditions: 7a (0.1 mmol).

b Determined by ^1^H NMR analysis of the crude reaction mixture using an internal standard.

### Reaction scope

**Fig. 2:**
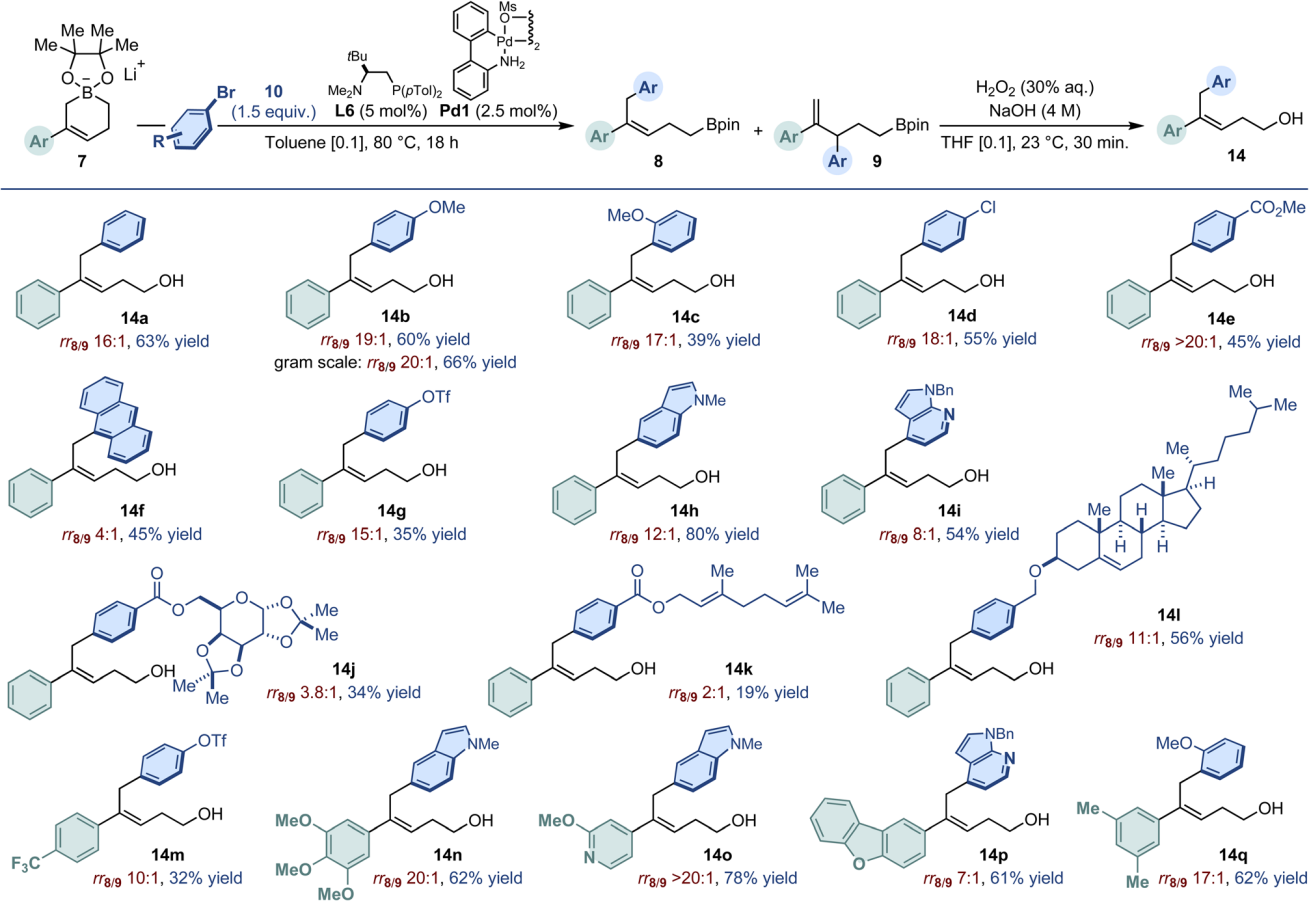
Scope of the Pd-catalyzed C1-selective arylation of cyclic allylboronates 7a–f (0.3 mmol scale). Regioselectivity (rr_8/9_) determined by ^1^H NMR spectroscopy after arylation using an internal standard. Yield over two steps of regio-isomerically pure homoallylic alcohols after oxidation and purification by column chromatography.

The scope of the C1-selective arylation of cyclic allylboronates 7 was delineated using the optimized conditions described in entry 7 of Table 1 and Fig. 2. To assess catalytic efficiency with the highest fidelity, the regioselectivity (rr_8/9_) was measured after catalysis and, for ease of purification and characterization, all products were isolated after alkali oxidation to the corresponding homoallylic alcohols (14). Therefore, the yields reported correspond to a two-step sequence. Our model substrate (7a) was first combined with a series of electronically and sterically diversified aryl bromides. While 4-bromoanisole delivered the cross-coupling product (14b) in 60% yield, its sterically more demanding isomer 2-bromoanisole led to a slightly diminished level of productivity (14c). Both reactions displayed excellent levels of regioselectivity. Reactivity was diminished using electron-deficient and/or π-extended bromoarenes (14d–14f). We observed that sensitive functionality such as triflate or esters were compatible with the optimized protocol even though the yields remained modest (14g, 14j, 14k). In contrast, *N*-containing heterocycles and a stereochemically complex steroid derivative afforded the cross-coupling products with excellent regioselectivity and practical yield (14h, 14i, 14l). To showcase the diversity offered by the catalytic method, we next randomly combined various cyclic boronates (7b–f) featuring either electron-rich, electron-poor aryls or heteroaryls with some of the most demanding aryl bromides already tested. Pleasingly, aside from 14m which was isolated in a modest 32% yield, all other combinations delivered the C1-arylation products with high levels of regioselectivity and the corresponding homoallylic alcohols in yields ranging from 61% to 78% (14n–14q). Noticeably, the scalability of the catalytic reaction was demonstrated using 7a (1.16 g, 3.6 mmol) and 4-bromoanisole as coupling partners to generate 14b in 66% yield (0.632 g) without affecting the level of regiocontrol (rr_8/9_ 20 : 1).

**Fig. 3:**
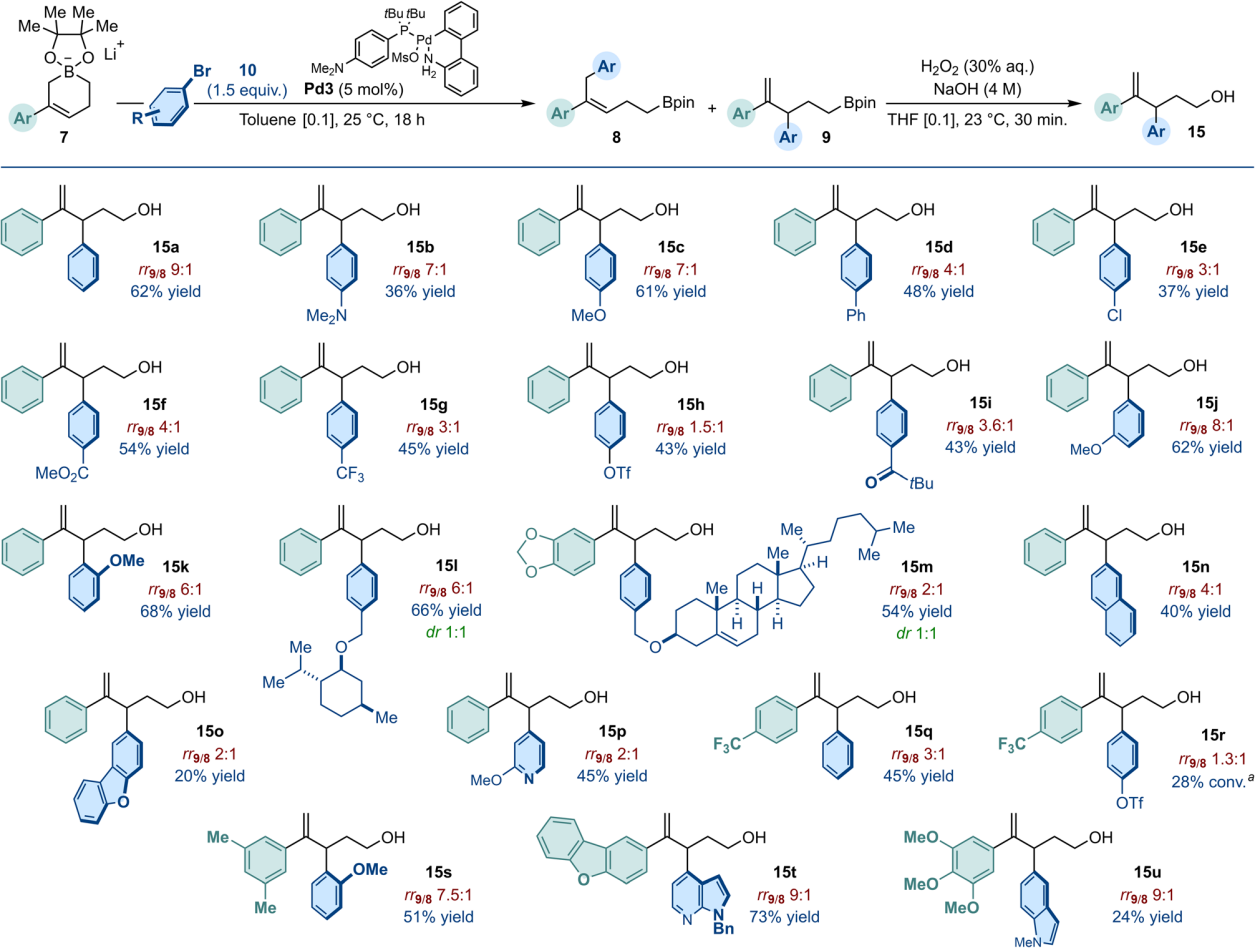
Scope of the Pd-catalyzed C3-selective arylation of cyclic allylboronates 7a–c, 7e–g (0.3 mmol scale). Regioselectivity (rr_9/8_) determined by ^1^H NMR spectroscopy after arylation using an internal standard. Yield over two steps of regio-isomerically pure homoallylic alcohols after oxidation and purification by column chromatography. ^*a*^Total conversion (8 + 9).

Emphasis was next placed on assessing the generality of the Pd-catalyzed C3-selective arylation of cyclic allylboronates 7 (Fig. 3). Using aryl bromides with either electron-donating, electron-neutral or electron-withdrawing *para*-substituents, we obtained yields ranging from 36% to 62% (15a–15i). These values typically reflect the regioisomeric ratio measured (1.5 : 1 < rr_9/8_ < 9 : 1) as well as the difficulty in separating the products of C1- and C3-arylation. Nonetheless, the functional group tolerance of the catalytic method is to be noted as ether, ester, ketone, halide, amine, trifluoromethyl and triflate were all compatible with the optimized reaction conditions. 2-Bromoanisole and 3-bromoanisole delivered the cross-coupling products in similarly high yield and regioselectivity (15j: rr_9/8_ 8 : 1, 62% yield; 15k: rr_9/8_ 6 : 1, 68% yield). Aryl bromides incorporating structurally complex scaffolds were equally well tolerated and 15l and 15m were isolated in 66% and 54% yield as 1 : 1 mixtures of diastereoisomers. Comparatively, heteroaromatic bromides and π-extended systems led to reduced yield and selectivity (15n–15o). We established that the electronic nature of the (hetero)aryl substituent of the cyclic boronate could be varied (7b–c, 7e–f) and combined with a diversity of (hetero)aryl bromides to afford the C3-arylation products with performances consistent with those obtained with our model substrate (15q–15u).

## Supporting organometallic study

**Fig. 4:**
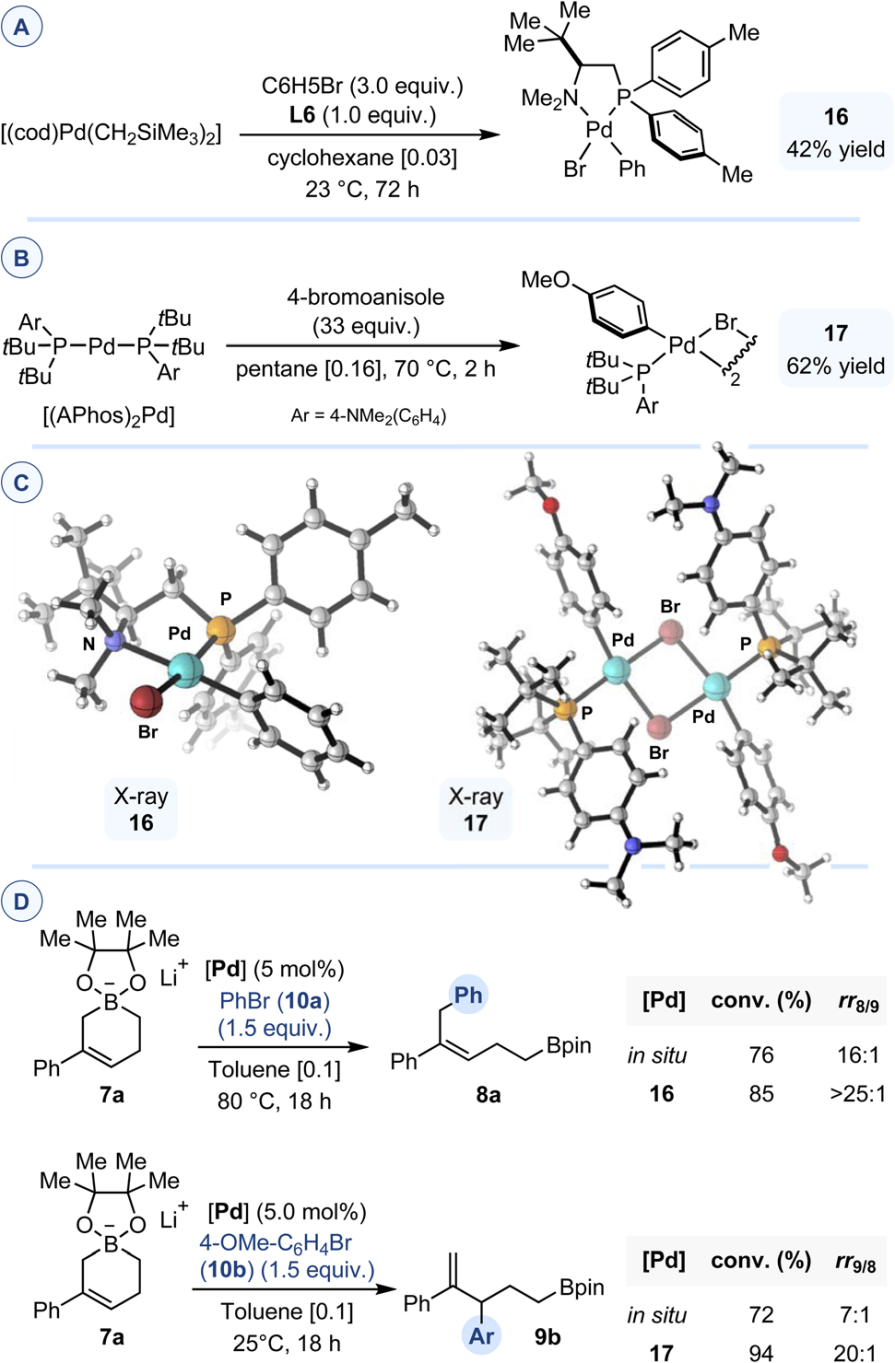
(A) Synthesis of oxidative addition complex 16. (B) Synthesis of oxidative addition complex 17. (C) X-ray structures of 16 and 17. (D) Comparative study between 16 or 17 and the corresponding *in situ* reaction conditions for C1- or C3-selective arylation of cyclic allylboronate 7a.

In a recent study, we showed that, depending on the initial stoichiometry between the metal precursor and the ligand, L6 can act as either a bidentate chelating (*P*,*N*) ligand or a monodentate phosphine ligand to generate Ni(ii) complexes adopting distorted tetrahedral geometries in both cases.^8^ Treatment of [(cod)Pd(CH_2_SiMe_3_)_2_] with 1.0 equivalent of L6 and 3.0 equivalent of bromobenzene led to the formation of the corresponding diamagnetic Pd(ii) oxidative addition complex 16 in 42% yield (Fig. 4A). Crystallographic analysis of 16 reveals formation of a 5-membered chelate by binding of both the P and N donor atoms to palladium, which imparts a slightly distorted square planar geometry to the metal center (P–Pd–N = 85.69(15)°; Br–Pd–C1 = 91.3(2)°; Pd–Br = 2.4951(8)Å; Pd–C1 = 2.007(7)Å). The strongest *trans* influence of the phosphorus atom in the chelating ligand dictates the relative positions of the bromine atom and the aryl ring and leads to the formation of a single isomeric structure. Reacting [(APhos)_2_Pd] with a large excess of 4-bromoanisole in pentane at 70 °C, led to the formation of the corresponding oxidative addition complex 17, which was isolated as a yellow solid in 62% yield (Fig. 4B). X-ray diffraction study of single crystals obtained by vapor diffusion showed a single isomer of a μ-bromo-bridged dinuclear palladium(ii) complex, with noticeable deviation from the ideal square planar geometry around each metal atom (P–Pd–C = 96.40(8)°, 95.82(8)°; Br–Pd–Br = 82.484(10)°, 82.533(10)°).^11^

When engaged in the cross-coupling reaction between 7a and bromobenzene, complex 16 displayed increased reactivity and regioselectivity to those obtained with the *in situ* protocol developed using L6 and Pd2. Improved conversion and regioselectivity (rr_9/8_ 20 : 1) were also achieved in the reaction between 7a and 4-bromoanisole using 17 as precatalyst compared to the protocol using Pd3 (Fig. 4D).

### Preliminary results in asymmetric catalysis and post-catalytic derivatization

**Fig. 5:**
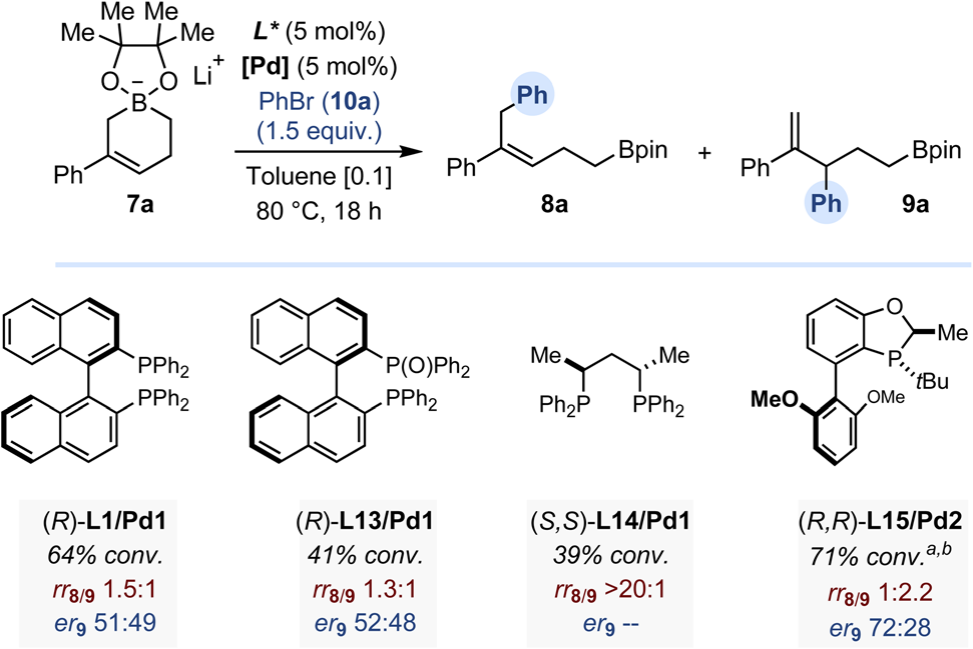
Pd-catalyzed enantioselective C3-arylation of allylboronate 7a (0.1 mmol scale). Conversion determined by ^1^H NMR against an internal standard. Enantiomeric ratio determined by HPLC equipped with a chiral stationary phase.^*a*^ At 25 °C. b 10 mol% (*R*,*R*)-L15.

Despite the potentially stereolabile nature of the allylic/benzylic tertiary stereocenter generated, the possibility to develop an enantioselective version of the Pd-catalyzed C3-selective arylation of cyclic allylboronates 7a was investigated by evaluating several chiral ligands and using bromobenzene as electrophile. A representative selection of our results is disclosed in Fig. 5 (see SI for details). Even though we could not achieve a regioselectivity comparable to that obtained with L6, we found that monophosphine L15 imparted a promising level of enantiocontrol, affording 9a in 72 : 28 er.^12^

**Fig. 6:**
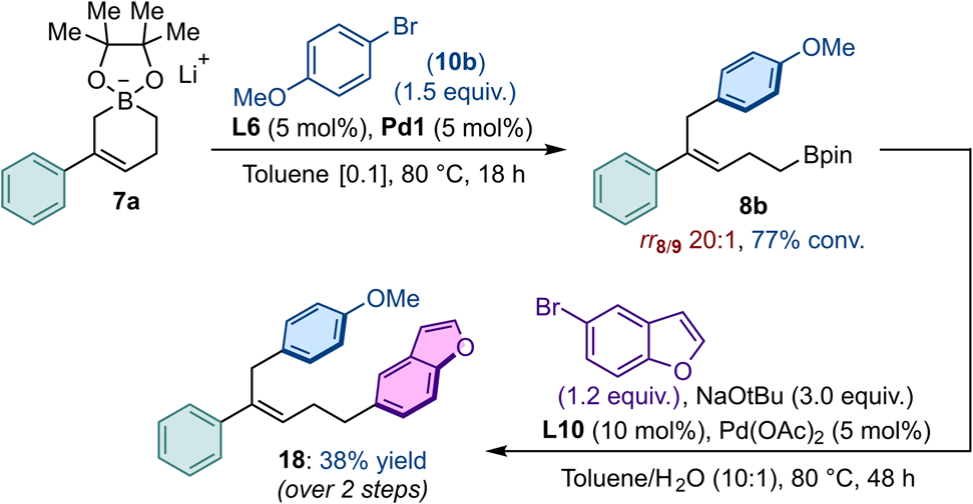
Post-catalytic functionalizations.

To demonstrate the potential of the Pd-catalyzed C1-arylation process, the homoallylborane obtained by reacting 7a with bromoanisole (10b) under the optimized protocol, was directly engaged in a subsequent Pd-catalyzed Suzuki–Miyaura cross-coupling using 5-bromobenzofuran under prototypical reaction conditions (L10, Pd(OAc)_2_, NaO*t*Bu). The corresponding polyarylated product (18) was isolated essentially as a single regioisomer in 38% overall yield (Fig. 6).

### Computational study

We sought to glean insights into the mechanisms of the C1- and C3-selective arylations of the cyclic boronates to get a preliminary understanding of the factors that govern selectivity for both systems. Preliminary Density Functional Theory calculations (DFT) revealed challenges inherent to the molecular systems under consideration, pertaining to the aggregation of the allylboronate (7a) and the potential requirement for explicit solvent molecules. Additionally, we consistently identified the lowest-energy reaction pathways as involving a dimeric form of the substrate and the participation of explicit solvent molecules (*vide infra*). This increased significantly the size of the systems and consequently the required computational resources as well as the conformational complexity. In light of these considerations, we employed the Universal Model for Atoms (UMA), recently developed and released by the Meta FAIR team, as a promising approach to retain DFT accuracy at the cost of semi-empirical methods.^13^ An interface was implemented *via* the “ExtOpt” functionality to fully exploit the advanced capabilities for geometry optimization and conformational sampling of ORCA 6.^14^ Implicit solvent effects were accounted for by integrating the ALPB solvation model from the xtb package as an additional correction to UMA-generated energies and gradients.^15^ The computational results presented herein were obtained at the ωB97M-V/def2-TZVPD//UMA-s-1 level of theory,^16^ with implicit ALPB solvation in toluene (see SI for details).^17^

### Speciation of allylboronate 7a in toluene

**Fig. 7:**
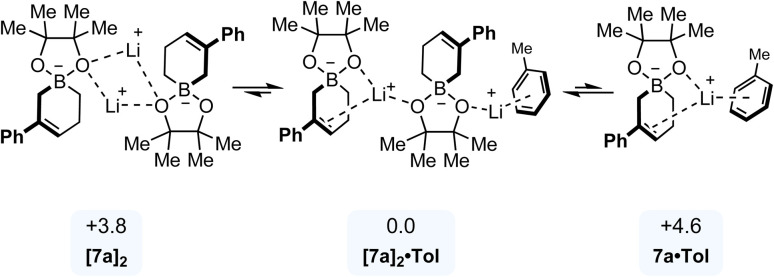
Speciation of 7a in toluene. Relative free energies in kcal mol^−1^ normalized to one monomer unit of 7a.

Considering the charged nature of the reactant in solution, we expected significant aggregation behavior in an apolar solvent such as toluene. In the absence of an experimentally determined X-ray structure of allylboronate 7a, an initial dimeric structure featuring bridging lithium cations was assumed (noted [7a]_2_, Fig. 7). To further investigate explicit solvation effects, molecular dynamics simulations were conducted first. The structure of [7a]_2_ was placed in a spherical cell containing 25 toluene molecules and bounded by repulsive harmonic walls, with the sphere radius adjusted to match the experimental density of toluene (see SI). Over the course of a 50 ps trajectory at 300 K, [7a]_2_ spontaneously reorganized into a more stable dimer ([7a]_2_·Tol) where one lithium cation interacts with one oxygen atom from each Bpin moieties and the alkene of one substrate, while the second lithium cation is bound to one oxygen atom of the (pinacolato)boron unit of the other substrate as well as an explicit molecule of toluene. A similar approach was employed to examine the monomeric form of 7a. The monomer was placed in a spherical cell with 25 toluene molecules and subjected to a 50 ps molecular dynamics simulation at 300 K, resulting in the microsolvated structure 7a·Tol. Both [7a]_2_·Tol and 7a·Tol were reoptimized at the (ωB97M-V/def2-TZVPD//UMA-s-1)ALPB-toluene level of theory. The former was found to be more stable by 4.6 kcal mol^−1^, thereby confirming the aggregated nature of 7a in toluene and underscoring the necessity of considering the dimer form in all subsequent investigations.

### C3-selective arylation

**Fig. 8:**
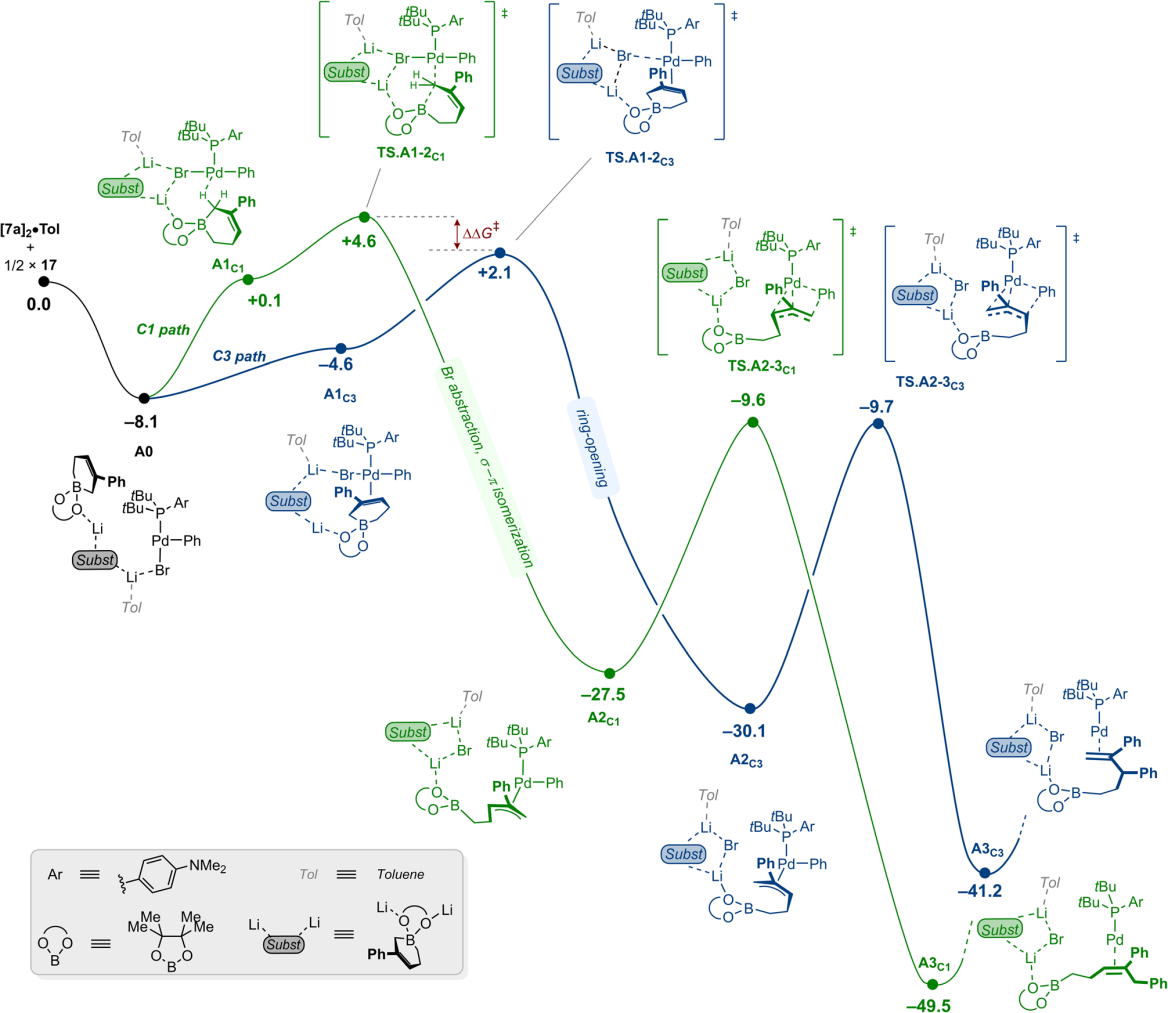
Computed free energy reaction profile (kcal mol^−1^) for the Pd-catalyzed C3-selective arylation of cyclic allylboronate 7a using the APhos-based catalytic system (Pd3). The structures being overall neutral, the individual charges on each atom have not been represented for the sake of clarity. Elementary steps that do not affect the overall analysis have been gathered in labelled boxes for clarity. See SI for the complete energetic landscape.

We first examined the C3-selective arylation pathway using the APhos-based precatalyst Pd3. The corresponding oxidative addition complex (17) was taken as energy reference together with the energetically most stable substrate aggregate [7a]_2_·Tol (Fig. 8). The initial formation of A0 as a simple adduct through electrostatic and van der Waals interactions was calculated to be favorable by 8.1 kcal mol^−1^. Noticeably, this intermediate displays an interaction between the bromine atom and the solvated Li ion. From A0, coordination of the substrate trans to the APhos ligand affords intermediate A1_C3_ at −4.6 kcal mol^−1^. Bromide abstraction (TS.A1-2_C3_, +2.1 kcal mol^−1^) followed by a barrierless ring-opening results in the irreversible formation of the π-allyl intermediate A2_C3_, located at −30.1 kcal mol^−1^. Subsequent reductive elimination occurs through TS.A2-3_C3_ with the highest overall activation barrier (+20.4 kcal mol^−1^), a value coherent with room-temperature conditions. From the product-bound intermediate A3_C3_, completion of the catalytic cycle only requires displacement of the product (15a) by coordination of bromobenzene (10a) and oxidative addition. The corresponding transition state was located at −24.5 kcal mol^−1^, which corresponds to a barrier of 16.7 kcal mol^−1^ from A3_C3_ (see SI).

For the competing C1-selective pathway using the APhos-based catalytic system, a sequence of similar elementary steps was initially explored (see SI). Coordination of the alkene of one allylboronate *cis* to the P-donor atom and subsequent halide abstraction led to a barrier located 6.0 kcal mol^−1^ higher than the corresponding pro-C3 transition state (TS.A1-2_C3_). At this stage, whilst preference for C3-arylation would be compatible with the observed experimental outcome, such a significant free energy difference would be inconsistent with the C1/C3 ratios measured experimentally. We tentatively attribute this important energetic penalty to the steric buttressing imposed by the APhos ligand, which disfavors binding at a *cis* coordination site. Alternatively, we could identify a distinct activation mode, which results in direct ring-opening of the cyclic allylboronate 7a. It proceeds first *via* intermediate A1_C1_ characterized by an agostic interaction with one of the H atoms at C1. The ensuing transition state (TS.A1-2_C1_) was located at +4.6 kcal mol^−1^, merely 2.5 kcal mol^−1^ higher than the barrier for the pro-C3 pathway – an energy difference more in line with our experimental observations (*vide supra*). This pathway next leads to the formation of σ-allyl intermediate, which undergoes concerted halide abstraction and σ–π isomerization to irreversibly form the pro-C1 π-allyl complex A2_C1_.

Similarly to the C3-selective pathway, reductive elimination occurs next *via*TS.A2-3_C1_ by overcoming a barrier of 17.9 kcal mol^−1^ to lead to A3_C1_, which lies at −49.5 kcal mol^−1^. Of important note, no energetically accessible interconversion pathway could be found between the two π-allyl intermediates A2_C1_ and A2_C3_, ruling out any Curtin–Hammett scenario in which regioselectivity would be dictated by the energetic barriers of the reductive elimination transition states. Overall, the origin of regioselectivity is determined at the early stages of the catalytic reaction by the orientation of the substrate upon binding to the Pd center (compare TS.A1-2_C3_ with TS.A1-2_C1_).

### C1-selective arylation

**Fig. 9:**
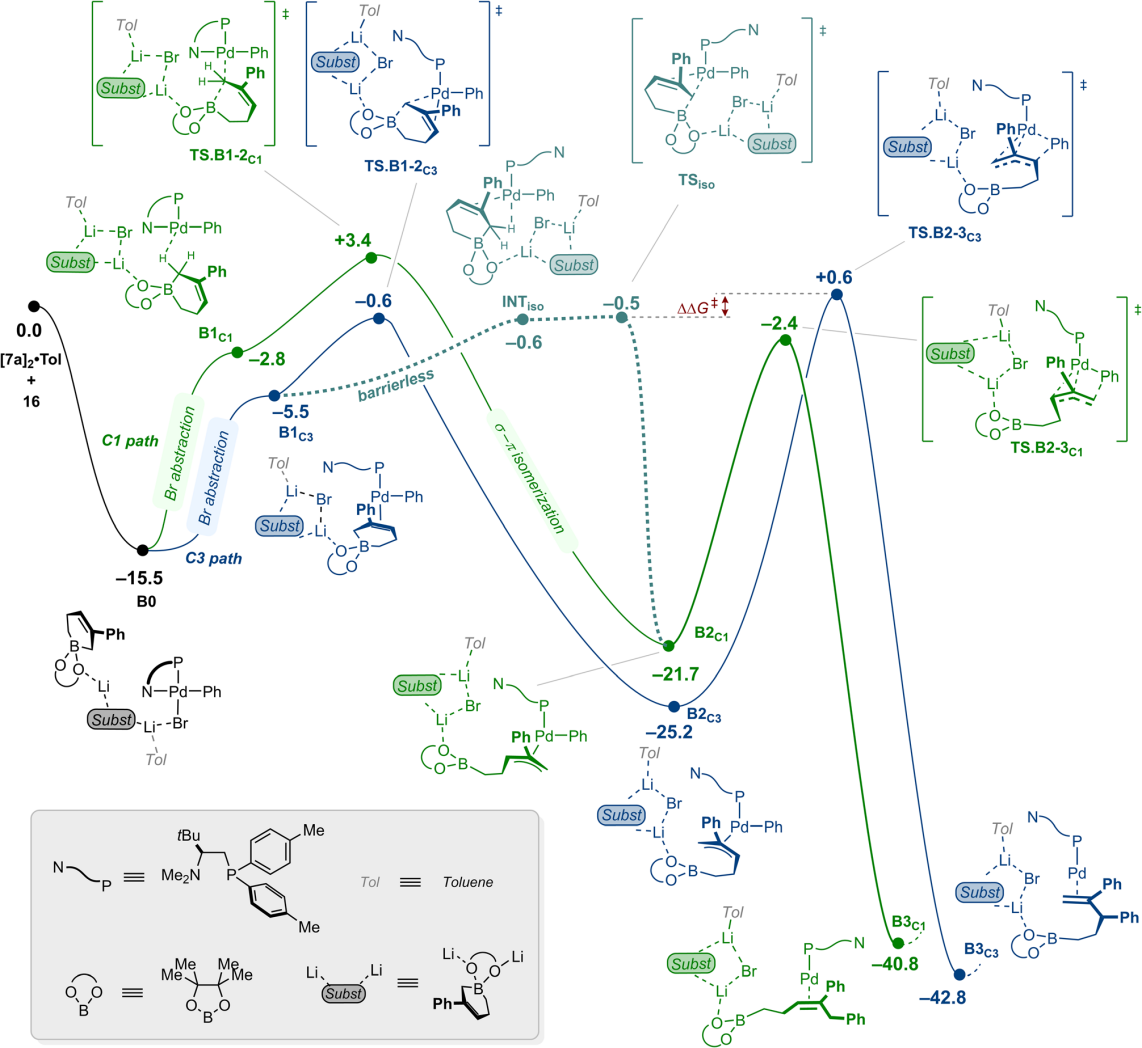
Computed free energy reaction profile (kcal mol^−1^) for the Pd-catalyzed C1-selective arylation of cyclic allylboronate 7a using the catalytic system using L6/Pd1. The structures being overall neutral, the individual charges on each atom have not been represented for the sake of clarity. Elementary steps that do not affect the overall analysis have been gathered in labelled boxes for clarity. See SI for the complete energetic landscape.

We next turned our attention to the L6/Pd1 catalytic system (Fig. 9). With the preceding results in hand, a similar pathway leading to the C3-arylation product was identified. Initial formation of an adduct of 16 with the substrate dimer [7a]_2_·Tol where L6 acts as a monodentate phosphine ligand was found to be thermodynamically downhill by 15.5 kcal mol^−1^ (B0). From B0, bromide abstraction and decoordination of the N-donor of the ligand places one alkene of the substrate aggregate *trans* to the P atom of L6 to form the coordinatively unsaturated inter-mediate B1_C3_ located at −5.5 kcal mol^−1^. Subsequent ring-opening occurred through TS.B2-3_C3_ calculated at −0.6 kcal mol^−1^ and led to the π-allyl complex B2_C3_, which lies in a thermodynamic well at −25.2 kcal mol^−1^. The ensuing reductive elimination passed through TS.B2-3_C3_ at +0.6 kcal mol^−1^ to yield the C3-arylation adduct B3_C3_. The latter is located downhill at −42.8 kcal mol^−1^, underscoring the irreversible nature of the product forming step.

A pathway leading to the C1-arylation product involving a similar succession of elementary transformation was found to be kinetically disfavored as it involved coordination of the substrate in a *cis* position relative to the sterically demanding P-donor atom of the ligand (See SI for details). Seeking to rationalize the experimentally observed regioselectivity preference for the formation of the C1-arylation product, an alternative solution involving once more a direct ring-opening by formal electrophilic substitution at C1 of the allylboronate was investigated (green path, Fig. 9). The most competitive pathway we could identify features, sequentially, a bromide abstraction followed by ring-opening of the allylboronate *via*TS.B1-2_C1_ located at +3.4 kcal mol^−1^. The resulting σ-allyl complex is prone to a rapid σ–to–π–allyl isomerization to afford π-allyl intermediate B2_C1_ at −21.7 kcal mol^−1^. Overall, this sequence fails to compete with the pro-C3 pathway, which funnels the system towards an accumulation of π-allyl complex B2_C3_. However, the obtained pro-C3 profile suggests that it is easier to reversibly ring-close the allylboronate rather than to proceed forward by reductive elimination. This is in stark contrast to the relative energetics observed for Pd3 (*vide supra*). This possibility was examined likewise from B2_C1_, yielding intermediate INT_iso_ (−0.6 kcal mol^−1^) *via* an accessible barrier for ring-closing located at −0.5 kcal mol^−1^ (TS_iso_). While attempting to improve the conformation of INT_iso_ by means of a molecular dynamics simulation, spontaneous and fast (<10 ps) isomerization to the pro-C3 intermediate B1_C3_ was observed. This strongly suggests a barrierless process between INT_iso_ and B1_C3_ (dashed line, Fig. 9). Overall, this process becomes key as it allows the system to interconvert the most stable and kinetically accessible π-allyl complex B2_C3_ into B2_C1_ through reversible ring-opening of the allylboronate and isomerization between a pro-C1 and a pro-C3 intermediate. With these results considered collectively, barriers governing regioselectivity can be calculated from B2_C3_ as follows: 25.8 kcal mol^−1^ through direct reductive elimination to provide the C3-arylation product; 24.7 kcal mol^−1^ through TS_iso_ to provide the C1-arylation product, both being compatible with the experimental reaction conditions (80 °C). Finally, completion of the catalytic reaction from adduct B3_C1_ is achieved through substitution of the product by bromobenzene (10a) and subsequent oxidative addition with an associated barrier of 22.4 kcal mol^−1^ (see SI).^18^

Of important note, the pathways presented herein feature transition states for reductive elimination, allylboronate ring-opening and bromide abstraction that lie within a narrow energetic range of [–2.4, +3.4]. Consequently, clearly assigning the regio determining steps can remain somewhat nontrivial owing to intrinsic DFT inaccuracies. Additionally, substrates electronics and sterics across the substrate scopes may further affect the relative energetics of these transition states, potentially changing the overall picture. Specifically, the situation may shift from (i) a Curtin–Hammett scenario such as presented in Fig. 8, where reductive elimination and reversible ring-opening of the allylboronate primarily determine regioselectivity, to (ii) another scenario more akin to that of the sterically demanding APhos system, in which regioselectivity is established earlier during the activation of the allylboronate in the absence of reversibility in the ring-opening of the allylboronate.

## Conclusions

In conclusion, we have developed two complementary protocols for the Pd-catalyzed regiodivergent arylation of the lithium salts of 6-membered cyclic allylboronates recently discovered in our laboratory. While a monodentate Buchwald-type ligand was identified for the C3-selective arylation reaction, a (*P*,*N*) ligand turned out to be optimal for the C1-selective arylation process. For both systems, using a broad array of aryl bromides or heteroaryl bromides, we could access small-molecule building blocks featuring a polysubstituted alkene and a boronic ester with high to very levels of regioselectivity. Despite the stereolabile nature of the product generated, preliminary results shed light on the possibility to develop an enantioselective variant of the C3-selective arylation. Mechanistically, computational investigations established the necessity to consider the substrate as a solvated dimer rather than a monomeric entity. We found that C3 selectivity is set at early stages of the catalytic cycle. Discrimination occurs during substrate coordination, a process that appears to be primarily governed by steric factors. By contrast, C1 selectivity occurs at a more advanced stage of the catalytic reaction. The system is in Curtin–Hammett equilibrium *via* an isomerization process between two η^2^-alkene-Pd(ii) intermediates that precede ring-opening transition states and the irreversible product-forming reductive elimination step.

## Author contributions

The manuscript was written through the contributions of all authors, and all authors have given approval to the final version.

## Conflicts of interest

There are no conflicts to declare.

## Supplementary Material

SC-OLF-D5SC07577G-s001

SC-OLF-D5SC07577G-s002

## Data Availability

The data supporting this article has been included as part of the supplementary information (SI). Supplementary information: this includes experimental procedures, characterization of all new compounds, spectroscopic data X-ray crystallographic data and molecular coordinates of computed structures (xyz), microkinetics models and an animation for the barrierless process between INTiso and B1C3 (mp4). See DOI: https://doi.org/10.1039/d5sc07577g. CCDC 2475759 (16) and 2475760 (17) contain the supplementary crystallographic data for this paper. All structures disclosed in this study have been generated using CylView.^19,20*a*,*b*^
