# Supplementary material for: Pd-catalyzed regiodivergent arylation of cyclic allylboronates
Source: Chem Sci. 2025 Oct 24;16(47):22656–65. doi: 10.1039/d5sc07577g (PMC12570269; doi:10.1039/d5sc07577g)

Pd-Catalyzed Regiodivergent Arylation of Cyclic Allylboronates

Cheng Zhang,[a] Baptiste Leforestier,[a] Céline Besnard[b] and Clément Mazet*,[a]

[a] Department of Organic Chemistry, University of Geneva, 30 quai Ernest Ansermet, 1211 Geneva, Switzerland.

[b] Laboratory of Crystallography, University of Geneva, 24 quai Ernest Ansermet, 1211 Geneva, Switzerland

clement.mazet@unige.ch

1. General information 3

2. Scope of cyclic allylboronates 4

3. Scope of aryl bromides 5

4. General Procedure for reaction optimization 6

4.1 Optimization for regiodivergent arylation 6

4.2 Chiral ligands screening for enantioselective C3 arylation 8

5. Scope of the Pd-catalyzed regiodivergent arylation reactions 10

5.1 General procedure for C1 arylation 10

5.2 General procedure for C3 arylation 23

5.3 Limitation of the methods 39

5.3.1 Limitation of C1 arylation 39

5.3.2 Limitation of C3 arylation 39

6. Gram scale experiment 40

7. Mechanistic investigations 41

7.1 Synthesis of Pd complex **16** 41

7.2 Synthesis of Pd complex **17** 43

7.3 Mechanistic experiments 44

7.3.1 Catalytic experiment with Pd complex **16** 44

7.3.2 Catalytic experiment with Pd complex **17** 44

8. Synthetic application 45

9. X-ray analyses 47

10. Computational studies 52

10.1 Geometry benchmarks 53

10.2 Substrate speciation 55

10.3 Pd-catalyzed C3-arylation pathway with **Pd3** 57

10.3.1 Main mechanism 57

10.3.2 Compared energetics with monomeric **7a** 60

10.3.3 Compared energetics with loss of LiBr – LiBr aggregation 61

10.4 Pd-catalyzed C1-arylation pathway with **L6/Pd1** 62

10.5 Deviation between UMA-s-1, UMA-m-1, DFT. 64

10.6 Deviation between DFT and g-xTB 66

11. References 68

12. NMR spectra 69

1. General information

Unless otherwise noted, all reactions were carried out under an inert atmosphere of nitrogen using either a two-manifold vacuum/inert gas lines or a M. Braun glovebox. Solvents were dried over activated alumina columns and further degassed by three successive "freeze-pump-thaw" cycles. Commercial reagents were purchased from Fluka, ABCR, TCI, Acros or Strem and used without purification unless otherwise noted. Liquid reagents were transferred with stainless steel syringes or cannula. Thin layer chromatography (TLC) was performed on plates of silica precoated with 0.25 mm Kieselgel 60 F_{254} from Merck. Flash chromatography was performed using silica gel SiliaFlash® P60 (230-400 mesh) from Silicycle.

NMR spectra were acquired at the University of Geneva NMR platform (https://www.unige.ch/sciences/chiorg/nmr/) using a 500 MHz Avance III Bruker NMR spectrometer equipped with a helium-cooled cryogenic 5-mm DCH ^{13}C-^{1}H/D Bruker probe, a 400 MHz Avance III HD NanoBay spectrometer equipped with a N_2 prodigy cryogenic 5 mm CPP BB(F)-H-D probe or a 300 MHz Avance III, HD NanoBay spectrometer, equipped with a 5 mm PA BBO, BB(F)-H-D probe. ^{1}H NMR spectra were referenced to $CDCl_3$ (7.26 ppm) and $^{13}C\{^{1}H\}$ NMR spectra were referenced to $CDCl_3$ (77.16 ppm). $^{19}F\{^{1}H\}$ NMR chemical shifts are reported in ppm with absolute reference relative to ^{1}H. HRMS data were obtained on a Xevo G2 Tof spectrometer (Ionization mode: ESI positive polarity; Mobile phase: MeOH 100 µl/min). Mass spectrum is calibrated using the MS lockspray system (LeuEnk calibration solution). Infrared spectra were obtained on a Perkin−Elmer 1650 FT-IR spectrometer using neat samples on a diamond ATR Golden Gate sampler. Melting points were recorded on a Buchi SMP-20 melting point apparatus using open glass capillaries.

The enantiomeric ratios (*er*) were determined by HPLC analyses. HPLC analyses were performed on a Shimadzu CTO-20AA equipped with DAICEL OD-H, OJ-H, AD-H and IC columns.

The allylboronates were prepared according to our previous report.[1] The palladium precursor $[(PdG3)_2]$ and ligand **L6** were synthesized according to the literature.[2-3] The palladium precursor APhosPdG3 was purchased from Strem and used as received.

2. Scope of cyclic allylboronates

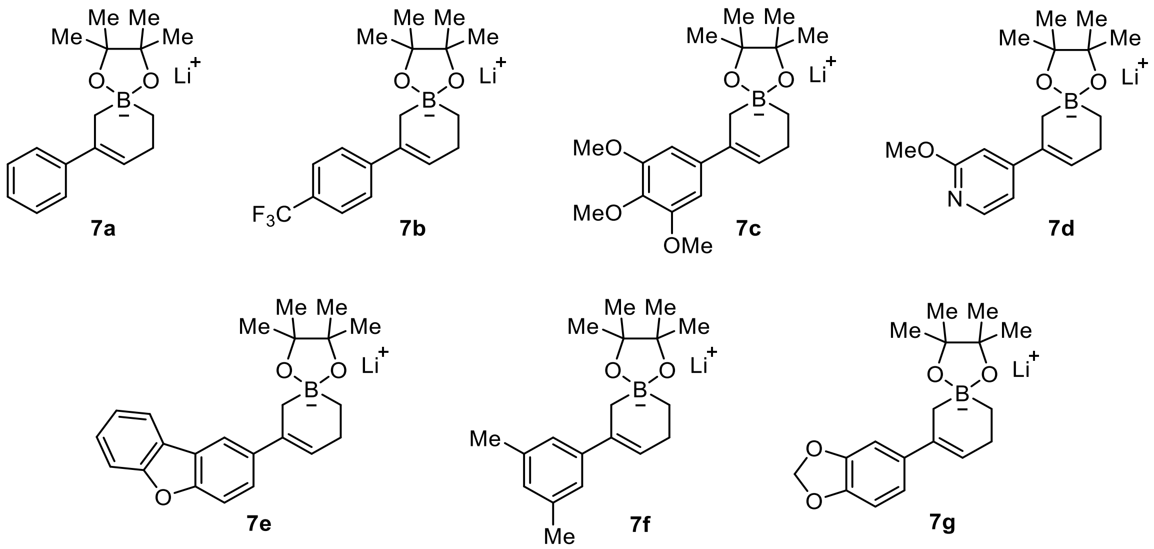

Figure S1. Cyclic allylboronates used in this work

3. Scope of aryl bromides

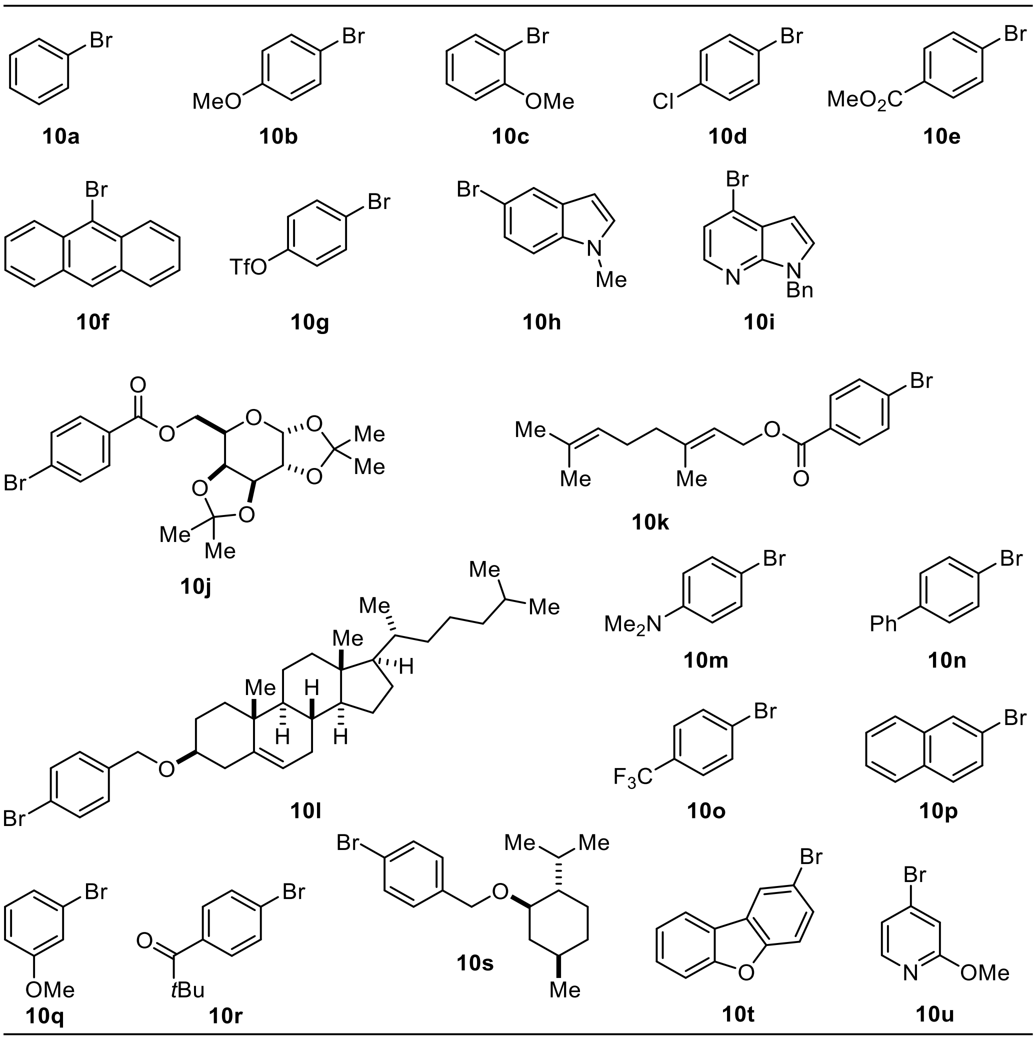

Figure S2. Aryl bromides used in this work

The aryl bromides **10a-f**, **10h-l**, **10m-q** and **10t-u** were commercially available, aryl bromides **10g**, **10j-l** and **10r-s** were synthesized according to the literature.[4-8]

4. General Procedure for reaction optimization

4.1 Optimization for regiodivergent arylation

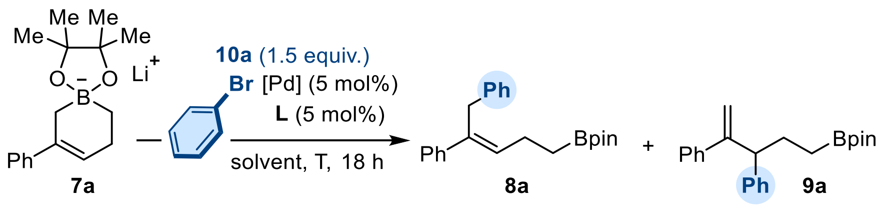

Figure S3. Reaction optimization

In a glovebox, to an oven dried Young-valve Schlenk equipped with a magnetic stir bar the appropriate precatalyst [Pd] (0.005 mmol, 5 mol%) and the appropriate ligand **L** (0.005 mmol, 5 mol%) were dissolved in the appropriate solvent (0.5 mL). After 30 min. at 25 °C, cyclic allylboronate **7a** (32 mg, 0.1 mmol, 1.0 equiv.) was added, followed by another 0.5 mL of solvent. Finally, PhBr **10a** (16 μL, 0.15 mmol, 1.5 equiv.) was added by microsyringe. The system was closed, and the mixture was stirred at the appropriate temperature. After 18 h, the mixture filtered on Celite, washed with Et_2O (3 × 5 mL) and concentrated under vacuum. The conversion and regioselectivity ($rr_{8/9}$) were measured by ^1H NMR against an internal standard.

Table S1. Optimization of the Pd-catalyzed regiodivergent arylation

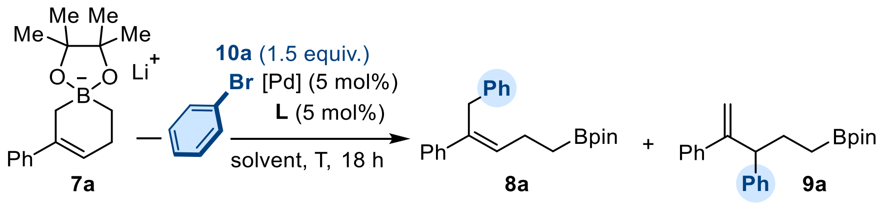

Entry	L/[Pd]	Solvent	T (°C)	conv. **8a**+**9a** (%)	$rr_{8/9}$
1	**L1**/**Pd1**	Toluene	25	<5	nd
2	**L1**/**Pd1**	Toluene	80	64	1 : 1.5
3	**L2**/**Pd1**	Toluene	80	21	1.3 : 1
4	**L3**/**Pd1**	Toluene	80	<5	nd
5	**L4**/**Pd1**	Toluene	80	13	2.3 : 1
6	**L5**/**Pd1**	Toluene	80	43	10:1
7	**L6**/**Pd1**	Toluene	80	72	>20 : 1
8	**L6**/**Pd1**	Toluene	25	<5	nd
9	**L7**/**Pd2**	Toluene	80	77	1 : 1.1
10	**L8**/**Pd2**	Toluene	80	71	1 : 1.8
11	**L9**/**Pd2**	Toluene	80	75	1.5 : 1
12	**L10**/**Pd2**	Toluene	80	65	2.4 : 1
13	**L11**/**Pd2**	Toluene	80	64	1.8 : 1
14	**L12**/**Pd2**	Toluene	80	58	1 : 1.2
15	**Pd3**	Toluene	80	68	1 : 2.8
16	**Pd3**	Toluene	40	70	1 : 6.0
17	**Pd3**	Toluene	25	81	1 : 15
18	**Pd3**	Dioxane	25	61	1 : 3.4
19	**Pd3**	DME	25	70	1 : 2.0
20	**Pd3**	THF	25	71	1 : 1.9

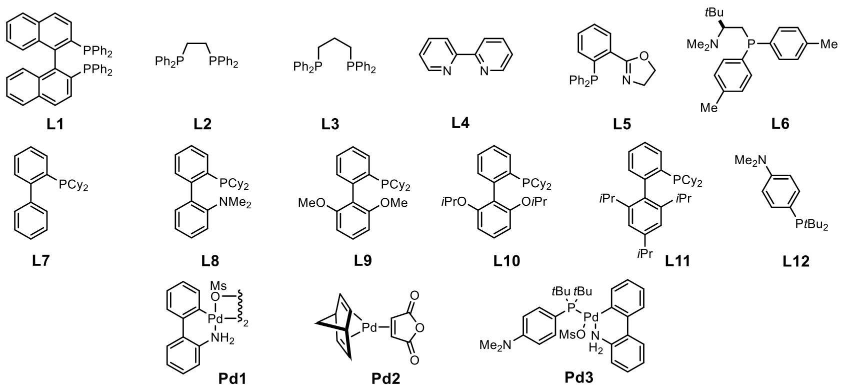

4.2 Chiral ligands screening for enantioselective C3 arylation

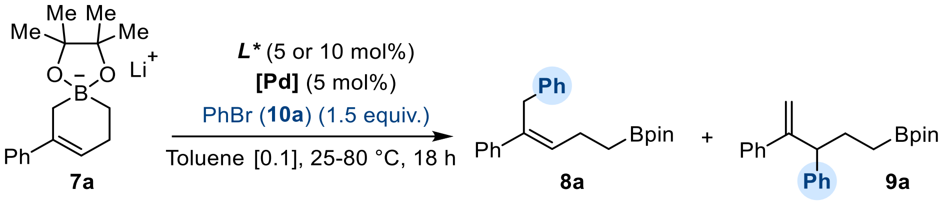

In a glovebox, to an oven dried Young-valve Schlenk equipped with a magnetic stir bar the appropriate precatalyst [Pd] (0.005 mmol, 5 mol%) and chiral ligand (5 or 10 mol%) were dissolved in toluene (0.5 mL), and stirred at 25 °C. After 30 min., the cyclic allylboronate **7a** (32 mg, 0.1 mmol, 1.0 equiv.) was added, followed by another 0.5 mL of toluene. Subsequently, PhBr **10a** (16 μL, 0.15 mmol, 1.5 equiv.) was added by microsyringe. The system was closed, and the mixture was stirred at the appropriate temperature. After 18 h, the solution was cooled to room temperature and the mixture filtered on Celite, washed with Et_2O (3 × 5 mL), and the solution was concentrated under vacuum. The conversion and regioselectivity ($rr_{8/9}$) were measured by ^{1}H NMR against an internal standard. The enantioselectivity (er_9) was measured after oxidation to homoallylic alcohol **15a**.

Selected chiral ligands evaluated in this study:

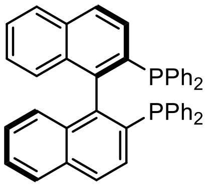

with [PdG3]$_2$

5 mol% (*R*)-**L1**
64% conv.
rr$_{8/9}$ 1.5:1
er$_9$ 51:49

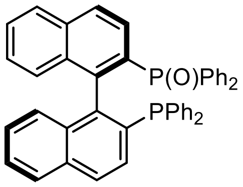

with [PdG3]$_2$

5 mol% (*R*)-**L13**
41% conv.
rr$_{8/9}$ 1.3:1
er$_9$ 52:48

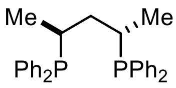

with [PdG3]$_2$

5 mol% (*S*,*S*)-**L14**
39% conv.
rr$_{8/9}$ >20:1
er$_9$ --

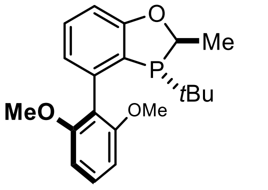

with [Pd(ma)(nbd)]

10 mol% (*R*,*R*)-**L15**
71% conv.
rr$_{8/9}$ 1:2.2
er$_9$ 72:28

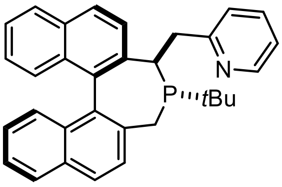

with [PdG3]$_2$

5 mol% (*R*,*R*,*R*)-**L16**
58% conv.
rr$_{8/9}$ 2.8:1
er$_9$ 68.5:31.5

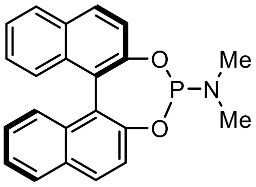

with [PdG3]$_2$

10 mol% (*R*)-**L17**
57% conv.
rr$_{8/9}$ 1.7:1
er$_9$ 51:49

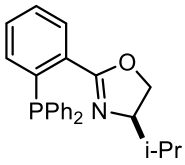

with [PdG3]$_2$

5 mol% (*R*)-**L18**
63% conv.
rr$_{8/9}$ 15:1
er$_9$ --

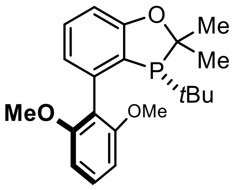

with [Pd(ma)(nbd)]

10 mol% (*R*)-**L19**
55% conv.
rr$_{8/9}$ 5:1
er$_9$ 59:41

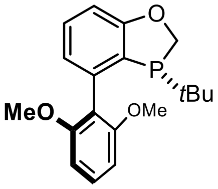

with [PdG3]$_2$

10 mol% (*R*)-**L20**
65% conv.
rr$_{8/9}$ 1:3
er$_9$ 51:49

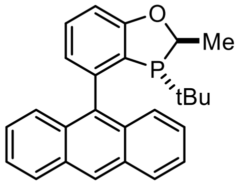

with [Pd(ma)(nbd)]

10 mol% (*R*,*R*)-**L21**
37% conv.
rr$_{8/9}$ 1.5:1
er$_9$ 51:49

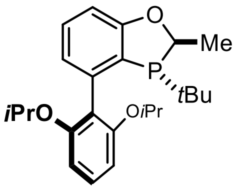

with [Pd(ma)(nbd)]

10 mol% (*R*,*R*)-**L22**
62% conv.
rr$_{8/9}$ 4.2:1
er$_9$ 54:46

Note: Ligands **L15**, and **L19-22** were tested at 25 °C, the others were tested at 80 °C.

5. Scope of the Pd-catalyzed regiodivergent arylation reactions

5.1 General procedure for C1 arylation

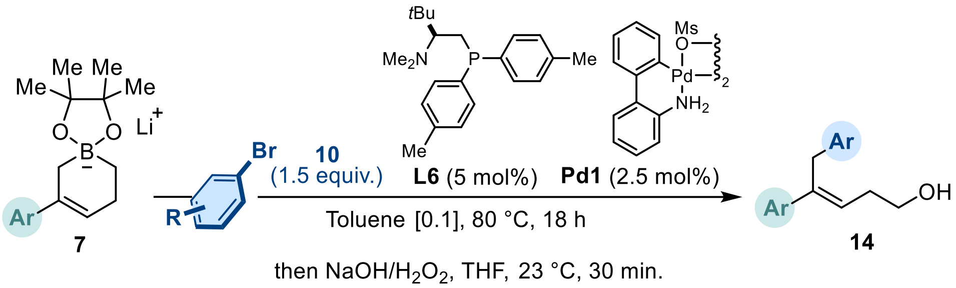

Figure S4. Pd-catalyzed C1-selective arylation of cyclic allylboronates

In a glovebox, to an oven dried screw capped Schlenk equipped with a magnetic stir bar, $(PdG_3)_2$ (5.6 mg, 2.5 mol%) and the ligand **L6** (5.2 mg, 5 mol%) were dissolved in toluene (0.5 mL), and stirred at 25 °C. After 30 min., the cyclic allylboronate **7** (0.3 mmol) was added, followed by another 2.5 mL of toluene. Subsequently, the ArBr **10** (0.45 mmol, 1.5 equiv.) was added. The system was closed, and the mixture was stirred at 80 °C. After 18 h, the solution was cooled to room temperature and the mixture filtered on Celite, washed with Et_2O (3 × 5 mL), and the solution was concentrated under vacuum. The alkali oxidation was conducted next. The crude reaction mixture was dissolved in THF (3.0 mL), and an aqueous NaOH solution (1.5 mL, 4 M) and 30% aq. H_2O_2 solution (1.5 mL) were added at room temperature. After 30 min. at 23 °C, the reaction mixture was extracted with CH_2Cl_2 (3 × 10 mL). The combined organic phases were dried over Na_2SO_4 and concentrated under reduced pressure after filtration. The residue was purified by flash column chromatography on silica gel using pentane/EtOAc as eluent to afford homoallylic alcohol **14**.

(*E*)-4,5-diphenylpent-3-en-1-ol (14a)

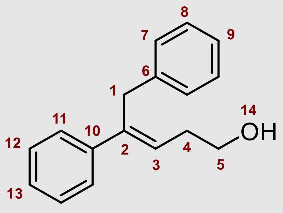

14a

Following the general procedure in **5.1** using boronate **7a** (0.3 mmol, 1.0 equiv.) and PhBr **10a** (0.45 mmol, 1.5 equiv.), compound **14a** was obtained as a yellow oil (45 mg, 63% yield, $rr_{8/9}$ = 16:1) after chromatography on silica gel (pentane/EtOAc, from 10:1 to 6:1).

TLC: 0.3, pentane/EtOAc = 5:1

^{1}H NMR (400 MHz, $CDCl_3$) δ (ppm) = 7.39 – 7.31 (m, 2H, H-Ar), 7.30 – 7.10 (m, 8H, H-Ar), 5.99 (t, $^3J_{H\text{-}H}$ = 7.3 Hz, 1H, H-3), 3.93 (s, 2H, H-1), 3.77 (q, $^3J_{H\text{-}H}$ = 6.2 Hz, 2H, H-5), 2.55 (q, $^3J_{H\text{-}H}$ = 6.7 Hz, 2H, H-4), 1.36 (t, $^3J_{H\text{-}H}$ = 5.6 Hz, 1H, H-14).

^{13}C{^{1}H} NMR (101 MHz, $CDCl_3$) δ (ppm) = 142.7 (C-10), 140.5 (C-2), 139.7 (C-6), 128.6 (C-Ar), 128.4 (C-Ar), 128.3 (C-Ar), 127.1 (C-Ar), 126.5 (C-Ar), 126.4 (C-3), 126.1 (C-Ar), 62.6 (C-5), 36.1 (C-1), 32.7 (C-4).

HRMS (ESI +**)**: calculated for $C_{17}H_{18}ONa$ $[M+Na]^+$: 261.1250; found: 261.1248.

IR (neat) ν (cm^{-1}) = 3330, 2924, 1600, 1492, 1451, 1045, 887, 750, 722, 694.

(*E*)-5-(4-methoxyphenyl)-4-phenylpent-3-en-1-ol (14b)

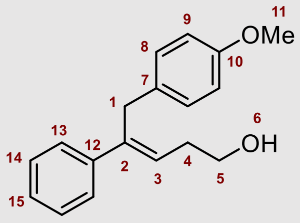

14b

Following the general procedure in **5.1** using boronate **7a** (0.3 mmol, 1.0 equiv.) and 1-bromo-4-methoxybenzene **10b** (0.45 mmol, 1.5 equiv.), compound **14b** was obtained as a red oil (48.3 mg, 60% yield, $rr_{8/9}$ = 19:1) after chromatography on silica gel (pentane/EtOAc, from 10:1 to 6:1).

TLC: 0.2, pentane/EtOAc = 5:1

^{1}H NMR (400 MHz, $CDCl_3$) δ (ppm) = 7.38 – 7.30 (m, 2H, H-13), 7.30 – 7.18 (m, 2H, H-14), 7.23 – 7.14 (m, 1H, H-15), 7.08 (d, $^3J_{H\text{-}H}$ = 8.8 Hz, 2H, H-8), 6.77 (d, $^3J_{H\text{-}zH}$ = 8.7 Hz, 2H, H-9), 5.96 (t, $^3J_{H\text{-}H}$ = 7.3 Hz, 1H, H-3), 3.86 (s, 2H, H-1), 3.77 (q, $^3J_{H\text{-}H}$ = 6.2 Hz, 2H, H-5), 3.75 (s, 3H, H-11), 2.55 (q, $^3J_{H\text{-}H}$ = 6.7 Hz, 2H, H-4), 1.36 (t, $^3J_{H\text{-}H}$ = 5.6 Hz, 1H, H-6).

^{13}C{^{1}H} NMR (101 MHz, $CDCl_3$) δ (ppm) = 158.0 (C-7), 142.8 (C-12), 140.9 (C-2), 131.7 (C-10), 129.2 (C-8), 128.4 (C-14), 127.0 (C-15), 126.5 (C-13), 126.2 (C-3), 114.0 (C-9), 62.6 (C-5), 55.4 (C-11), 35.1 (C-1), 32.7 (C-4).

HRMS (ESI +**)**: calculated for $C_{17}H_{21}O_2Na$ $[M+H]^+$: 269.1537; found: 269.1538.

IR (neat) ν (cm^{-1}) = 3402, 2917, 1608, 1509, 1443, 1300, 1243, 1176, 1033, 873, 809, 696.

(*E*)-5-(2-methoxyphenyl)-4-phenylpent-3-en-1-ol (14c)

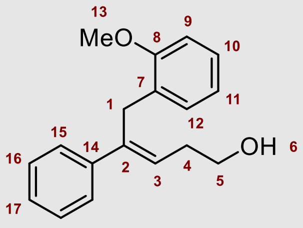

14c

Following the general procedure in **5.1** using boronate **7a** (0.3 mmol, 1.0 equiv.) and 1-bromo-2-methoxybenzene **10c** (0.45 mmol, 1.5 equiv.), compound **14c** was obtained as a yellow oil (31.2 mg, 39% yield, $rr_{8/9}$ = 17:1) after chromatography on silica gel (pentane/EtOAc, from 10:1 to 6:1).

TLC: 0.3, pentane/EtOAc = 5:1

^{1}H NMR (400 MHz, $CDCl_3$) δ (ppm) = 7.41 – 7.30 (m, 2H, H-Ar), 7.28 – 7.15 (m, 3H, H-Ar), 7.14-7.10 (m, 1H, H-Ar), 7.07-7.00 (m, 1H, H-Ar), 6.85-6.73 (m, 2H, H-Ar), 6.03 (t, $^3J_{H-H}$ = 7.3 Hz, 1H, H-3), 3.87 (s, 2H, H-1), 3.85 (s, 3H, H-13), 3.81 – 3.69 (m, 2H, H-5), 2.50 (q, $^3J_{H-H}$ = 6.8 Hz, 2H, H-4), 1.37 (t, $^3J_{H-H}$ = 5.8 Hz, 1H, H-6).

^{13}C{^{1}H} NMR (101 MHz, $CDCl_3$) δ (ppm) = 157.4 (C-7), 142.9 (C-14), 140.1 (C-2), 128.7 (C-Ar), 128.3 (C-Ar), 127.8 (C-Ar), 127.2 (C-Ar), 126.9 (C-Ar), 126.7 (C-3), 126.3 (C-Ar), 120.6 (C-10), 110.0 (C-Ar), 62.6 (C-5), 55.4 (C-13), 32.6 (C-4), 29.8 (C-1).

HRMS (ESI +**)**: calculated for $C_{18}H_{20}O_2Na$ $[M+Na]^+$: 291.1356; found: 291.1357.

IR (neat) ν (cm^{-1}) = 3346, 2932, 1685, 1598, 1489, 1461, 1239, 1105, 1050, 1029, 748, 696.

(*E*)-5-(4-chlorophenyl)-4-phenylpent-3-en-1-ol (14d)

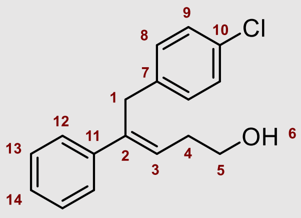

14d

Following the general procedure in **5.1** using boronate **7a** (0.3 mmol, 1.0 equiv.) and 1-bromo-4-chlorobenzene **10d** (0.45 mmol, 1.5 equiv.), compound **14d** was obtained as a yellow oil (45 mg, 55% yield, $rr_{8/9}$ = 18:1) after chromatography on silica gel (pentane/EtOAc, from 10:1 to 6:1).

TLC: 0.3, pentane/EtOAc = 5:1

^{1}H NMR (400 MHz, $CDCl_3$) δ (ppm) = 7.35 – 7.28 (m, 2H, H-Ar), 7.30 – 7.21 (m, 2H, H-Ar), 7.24 – 7.15 (m, 3H, H-Ar), 7.14 – 7.06 (m, 2H, H-8), 5.99 (t, $^3J_{H-H}$ = 7.3 Hz, 1H, H-3), 3.88 (s, 2H, H-1), 3.77 (dd, $^3J_{H-H}$ = 7.0, 4.5 Hz, 2H, H-5), 2.53 (q, $^3J_{H-H}$ = 6.7 Hz, 2H, H-4), 1.37 (t, $^3J_{H-H}$ = 5.5 Hz, 1H, H-6).

$^{13}C\{^{1}H\}$ NMR (101 MHz, $CDCl_3$) δ (ppm) = 142.4 (C-11), 140.1 (C-2), 138.2 (C-7), 131.8 (C-10), 129.6 (C-8), 128.7 (C-Ar), 128.5 (C-Ar), 127.2 (C-Ar), 126.8 (C-3), 126.4 (C-Ar), 62.5 (C-5), 35.4 (C-1), 32.7 (C-4).

HRMS (ESI +**)**: calculated for $C_{17}H_{17}ClONa$ $[M+Na]^+$: 295.0861; found: 295.0870.

IR (neat) ν (cm^{-1}) = 3345, 2923, 1683, 1489, 1444, 1274, 1090, 1045, 1014, 801, 761, 696.

methyl (*E*)-4-(5-hydroxy-2-phenylpent-2-en-1-yl)benzoate (14e)

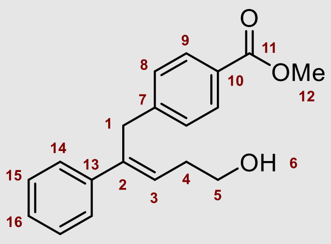

14e

Following the general procedure in **5.1** using boronate **7a** (0.3 mmol, 1.0 equiv.) and methyl 4-bromobenzoate **10e** (0.45 mmol, 1.5 equiv.), compound **14e** was obtained as a yellow oil (40 mg, 45% yield, $rr_{8/9}$ >20:1) after chromatography on silica gel (pentane/EtOAc, from 10:1 to 6:1).

TLC: 0.3, pentane/EtOAc = 5:1

^{1}H NMR (400 MHz, $CDCl_3$) δ (ppm) = 7.91 (d, $^3J_{H\text{-}H}$ = 8.3 Hz, 2H, H-9), 7.38 – 7.29 (m, 2H, H-Ar), 7.30 – 7.22 (m, 4H, H-Ar), 7.25 – 7.16 (m, 1H, H-Ar), 6.03 (t, $^3J_{H\text{-}H}$ = 7.3 Hz, 1H, H-3), 3.98 (s, 2H, H-1), 3.88 (s, 3H, H-12), 3.79 (t, $^3J_{H\text{-}H}$ = 6.6 Hz, 2H, H-5), 2.55 (q, $^3J_{H\text{-}H}$ = 6.7 Hz, 2H, H-4), 1.47 (s, 1H, H-6).

$^{13}C\{^{1}H\}$ NMR (101 MHz, $CDCl_3$) δ (ppm) = 167.2 (C-11), 145.3 (C-7), 142.3 (C-13), 139.7 (C-2), 129.9 (C-9), 128.5 (C-Ar), 128.3 (C-Ar), 128.1 (C-Ar), 127.2 (C-Ar), 127.0 (C-3), 126.4 (C-Ar), 62.5 (C-5), 52.1 (C-12), 36.1 (C-1), 32.7 (C-4).

HRMS (ESI +**)**: calculated for $C_{19}H_{21}O_3Na$ $[M+H]^+$: 297.1486; found: 297.1467.

IR (neat) ν (cm^{-1}) = 3424, 2910, 1706, 1607, 1427, 1277, 1102, 1038, 889, 760, 733, 698.

(*E*)-5-(anthracen-9-yl)-4-phenylpent-3-en-1-ol (14f)

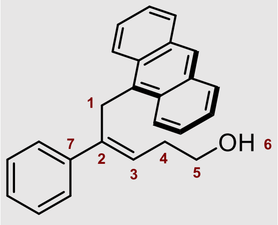

14f

Following the general procedure in **5.1** using boronate **7a** (0.3 mmol, 1.0 equiv.) and 9-bromoanthracene **10f** (0.45 mmol, 1.5 equiv.), compound **14f** was obtained as a yellow solid (45.7 mg, 45% yield, $rr_{8/9}$ = 4:1) after chromatography on silica gel (pentane/EtOAc, from 10:1 to 6:1).

TLC: 0.2, pentane/EtOAc = 5:1

^{1}H NMR (400 MHz, $CDCl_3$) δ (ppm) = 8.32 – 8.22 (m, 3H, H-Ar), 7.98 – 7.91 (m, 2H, H-Ar), 7.51 – 7.37 (m, 4H, H-Ar), 7.11 – 7.04 (m, 2H, H-Ar), 7.04 – 6.95 (m, 3H, H-Ar), 5.65 (tt, $^3J_{H-H}$ = 7.2, 1.7 Hz, 1H, H-3), 4.82 (s, 2H, H-1), 3.61 (s, 2H, H-5), 2.38 (q, $^3J_{H-H}$ = 6.5 Hz, 2H, H-4), 1.22 (s, 1H, H-6).

^{13}C{^{1}H} NMR (101 MHz, $CDCl_3$) δ (ppm) = 143.7 (C-7), 142.8 (C-2), 131.9 (C-Ar), 131.5 (C-Ar), 130.7 (C-Ar), 129.3 (C-Ar), 127.7 (C-Ar), 127.1 (C-Ar), 126.60 (C-Ar), 126.57 (C-Ar), 126.4 (C-3), 125.6 (C-Ar), 124.91 (C-Ar), 124.86 (C-Ar), 62.6 (C-5), 32.1 (C-4), 30.6 (C-1).

HRMS (ESI +**)**: calculated for $C_{25}H_{23}O$ $[M+H]^+$: 339.1744; found: 339.1743.

IR (neat) ν (cm^{-1}) = 3319, 2923, 1442, 1343, 1041, 889, 837, 766, 727, 698, 545.

m.p.: 100.1–100.8 °C

(*E*)-4-(5-hydroxy-2-phenylpent-2-en-1-yl)phenyl trifluoromethanesulfonate (14g)

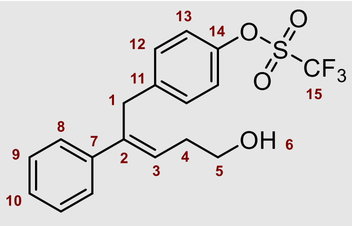

14g

Following the general procedure in **5.1** using boronate **7a** (0.3 mmol, 1.0 equiv.) and 4-bromophenyl trifluoromethanesulfonate **10g** (0.45 mmol, 1.5 equiv.), compound **14g** was obtained as a yellow oil (40 mg, 35% yield, $rr_{8/9}$ = 15:1) after chromatography on silica gel (pentane/EtOAc, from 10:1 to 6:1).

TLC: 0.2, pentane/EtOAc = 5:1

^{1}H NMR (400 MHz, $CDCl_3$) δ (ppm) = 7.35 – 7.28 (m, 2H, H-Ar), 7.31 – 7.24 (m, 2H, H-Ar), 7.27 – 7.19 (m, 3H, H-Ar), 7.17 – 7.08 (m, 2H, H-Ar), 6.02 (t, $^3J_{H-H}$ = 7.3 Hz, 1H, H-3), 3.94 (s, 2H, H-1), 3.82 – 3.74 (m, 2H, H-5), 2.52 (q, $^3J_{H-H}$ = 6.7 Hz, 2H, H-4), 1.42 (s, 1H, H-6).

^{13}C{^{1}H} NMR (101 MHz, $CDCl_3$) δ (ppm) = 148.0 (C-11), 142.1 (C-7), 140.3 (C-14), 139.7 (C-2), 130.0 (C-12), 128.6 (C-Ar), 127.4 (C-Ar), 127.2 (C-3), 126.4 (C-Ar), 121.4 (C-Ar), 118.9 (q, $^1J_{C-F}$ = 320.8 Hz, C-15), 62.4 (C-5), 35.4 (C-1), 32.6 (C-4).

^{19}F{^{1}H} NMR (282 MHz, $CDCl_3$) δ (ppm) = -72.9

HRMS (ESI +**)**: calculated for $C_{18}H_{17}F_3O_4SNa$ $[M+Na]^+$: 409.0692; found: 409.0673.

IR (neat) ν (cm^{-1}) = 3347, 2928, 1598, 1498, 1416, 1206, 1136, 1046, 1016, 884, 751, 697.

(*E*)-5-(1-methyl-1H-indol-5-yl)-4-phenylpent-3-en-1-ol (14h)

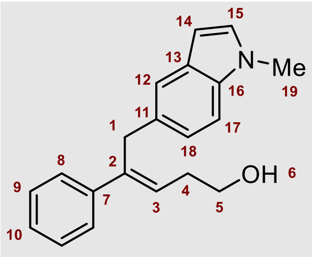

14h

Following the general procedure in **5.1** using boronate **7a** (0.3 mmol, 1.0 equiv.) and 5-bromo-1-methyl-1H-indole **10h** (0.45 mmol, 1.5 equiv.), compound **14h** was obtained as a yellow oil (70 mg, 80% yield, $rr_{8/9}$ = 12:1) after chromatography on silica gel (pentane/EtOAc, from 5:1 to 2:1).

TLC: 0.2, pentane/EtOAc = 1:1

^{1}H NMR (400 MHz, $CDCl_3$) δ (ppm) = 7.52 – 7.43 (m, 3H, H-Ar), 7.35 – 7.20 (m, 4H, H-Ar), 7.14 (dd, J_{H-H} = 8.4, 1.7 Hz, 1H, H-Ar), 7.06 (d, $^3J_{H-H}$ = 3.1 Hz, 1H, H-15), 6.44 (dd, J_{H-H} = 3.1, 0.9 Hz, 1H, H-14), 6.08 (t, $^3J_{H-H}$ = 7.3 Hz, 1H, H-3), 4.10 (s, 2H, H-1), 3.85 (s, 2H, H-5), 3.81 (s, 3H, H-19), 2.67 (q, $^3J_{H-H}$ = 6.7 Hz, 2H, H-4), 1.45 (s, 1H, H-6).

^{13}C{^{1}H} NMR (101 MHz, $CDCl_3$) δ (ppm) = 143.1 (C-7), 141.3 (C-2), 135.6 (C-16), 130.4 (C-11), 129.0 (C-15), 128.8 (C-13), 128.3 (C-Ar), 126.9 (C-Ar), 126.5 (C-Ar), 126.0 (C-3), 122.3 (C-Ar), 119.9 (C-Ar), 109.2 (C-Ar), 100.7 (C-14), 62.7 (C-5), 36.0 (C-1), 33.0 (C-19), 32.8 (C-4).

HRMS (ESI +**)**: calculated for $C_{20}H_{21}NONa$ $[M+Na]^+$: 314.1516; found: 314.1506.

IR (neat) ν (cm^{-1}) = 3348, 2921, 1491, 1444, 1336, 1244, 1154, 1044, 875, 757, 695.

(*E*)-5-(1-benzyl-1H-pyrrolo[2,3-b]pyridin-4-yl)-4-phenylpent-3-en-1-ol (14i)

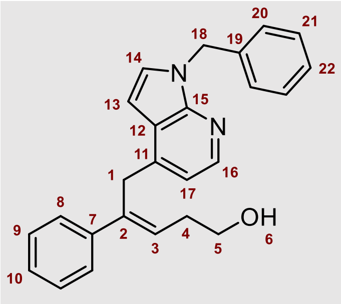

14i

Following the general procedure in **5.1** using boronate **7a** (0.3 mmol, 1.0 equiv.) and 1-benzyl-4-bromo-1H-pyrrolo[2,3-b]pyridine **10i** (0.45 mmol, 1.5 equiv.), compound **14i** was obtained as a yellow oil (60 mg, 54% yield, *rr*$_{8/9}$ = 8:1) after chromatography on silica gel (pentane/EtOAc, from 5:1 to 2:1).

TLC: 0.3, pentane/EtOAc = 1:1

^{1}H NMR (400 MHz, $CDCl_3$) δ (ppm) = 8.20 (d, $^3J_{H\text{-}H}$ = 5.0 Hz, 1H, H-16), 7.43 – 7.34 (m, 2H, H-Ar), 7.36 – 7.18 (m, 8H, H-Ar), 7.16 (d, $^3J_{H\text{-}H}$ = 3.5 Hz, 1H, H-14), 6.88 (d, $^3J_{H\text{-}H}$ = 4.9 Hz, 1H, H-17), 6.56 (d, $^3J_{H\text{-}H}$ = 3.6 Hz, 1H, H-13), 6.14 (t, $^3J_{H\text{-}H}$ = 7.3 Hz, 1H, H-3), 5.49 (s, 2H, H-18), 4.19 (s, 2H, H-1), 3.78 (q, $^3J_{H\text{-}H}$ = 6.5 Hz, 2H, H-5), 2.54 (q, $^3J_{H\text{-}H}$ = 6.7 Hz, 2H, H-4), 1.48 (t, $^3J_{H\text{-}H}$ = 5.6 Hz, 1H, H-6).

^{13}C{^{1}H} NMR (101 MHz, $CDCl_3$) δ (ppm) = 147.7 (C-15), 143.6 (C-16), 142.5 (C-7), 141.5 (C-11), 138.7 (C-2), 137.9 (C-19), 128.8 (C-Ar), 128.5 (C-Ar), 127.72 (C-Ar), 127.67 (C-20), 127.5 (C-3), 127.3 (C-Ar), 127.2 (C-14), 126.2 (C-Ar), 120.2 (C-12), 114.9 (C-17), 98.6 (C-13), 62.5 (C-5), 48.1 (C-18), 33.1 (C-1), 32.8 (C-4).

HRMS (ESI +**)**: calculated for $C_{25}H_{25}N_2O$ $[M+H]^+$: 369.1962; found: 369.1989.

IR (neat) ν (cm^{-1}) = 3320, 2922, 1584, 1509, 1494, 1417, 1346, 1311, 1047, 908, 822, 719.

((3aR,5R,5aS,8aS,8bR)-2,2,7,7-tetramethyltetrahydro-5H-bis([1,3]dioxolo)[4,5-b:4',5'-d]pyran-5-yl)methyl 4-((*E*)-5-hydroxy-2-phenylpent-2-en-1-yl)benzoate (14j)

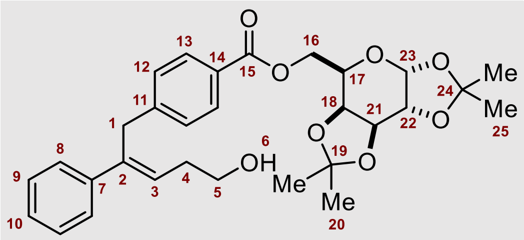

14j

Following the general procedure in **5.1** using boronate **7a** (0.3 mmol, 1.0 equiv.) and ArBr **10j** (0.45 mmol, 1.5 equiv.), compound **14j** was obtained as a yellow oil (54 mg, 34% yield, *rr*$_{8/9}$ = 3.8:1) after chromatography on silica gel (pentane/EtOAc, from 10:1 to 5:1).

TLC: 0.3, pentane/EtOAc = 5:1

^{1}H NMR (400 MHz, $CDCl_3$) δ (ppm) = 7.91 (d, $^3J_{H\text{-}H}$ = 8.3 Hz, 2H, H-13), 7.35 – 7.27 (m, 2H, H-Ar), 7.29 – 7.15 (m, 5H, H-Ar), 6.02 (t, $^3J_{H\text{-}H}$ = 7.3 Hz, 1H, H-3), 5.55 (d, $^3J_{H\text{-}H}$ = 5.0 Hz, 1H, H-23), 4.64 (dd, $^3J_{H\text{-}H}$ = 7.8, 2.5 Hz, 1H, H-18 or H-21 or H-22), 4.48 (dd, $^3J_{H\text{-}H}$ = 11.4, 5.1 Hz,

1H, H-16), 4.39 (dd, $^3J_{H-H}$ = 11.5, 7.4 Hz, 1H, H-16), 4.37 – 4.27 (m, 2H, H-18 or H-21 or H-22), 4.18 – 4.10 (m, 1H, H-17), 3.96 (s, 2H, H-1), 3.82 – 3.73 (m, 2H, H-5), 2.53 (q, $^3J_{H-H}$ = 6.7 Hz, 2H, H-4), 1.50 (s, 3H, H-20 or H-25), 1.47 (s, 3H, H-20 or H-25), 1.40 (t, $^3J_{H-H}$ = 5.6 Hz, 1H, H-6), 1.35 (s, 3H, H-20 or H-25), 1.33 (s, 3H, H-20 or H-25).

^{13}C{^{1}H} NMR (101 MHz, $CDCl_3$) δ (ppm) = 166.5 (C-15), 145.4 (C-11), 142.3 (C-7), 139.8 (C-2), 130.1 (C-12 or C-13), 128.5 (C-Ar), 128.3 (C-Ar), 128.1 (C-14), 127.2 (C-3), 127.0 (C-Ar), 126.4 (C-Ar), 109.8 (C-19 or C-24), 108.9 (C-19 or C-24), 96.5 (C-23), 71.3 (C-18), 70.9 (C-21 or C-22), 70.7 (C-21 or C-22), 66.3 (C-17), 63.8 (C-16), 62.5 (C-5), 36.2 (C-1), 32.7 (C-4), 26.2 (C-20 or C-25), 26.1 (C-20 or C-25), 25.1 (C-20 or C-25), 24.7 (C-20 or C-25).

HRMS (ESI +**)**: calculated for $C_{30}H_{37}O_8$ $[M+H]^+$: 525.2483; found: 525.2460.

IR (neat) ν (cm^{-1}) = 3443, 2923, 1716, 1609, 1381, 1274, 1175, 1101, 1066, 1004, 891, 755, 697.

(*E*)-3,7-dimethylocta-2,6-dien-1-yl 4-((*E*)-5-hydroxy-2-phenylpent-2-en-1-yl)benzoate (14k)

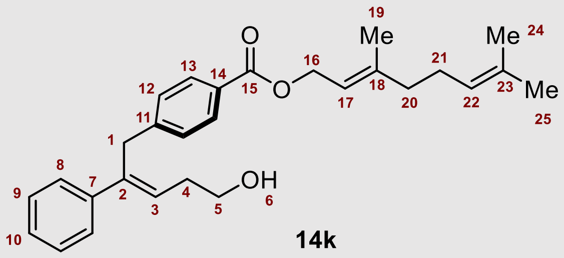

Following the general procedure in **5.1** using boronate **7a** (0.3 mmol, 1.0 equiv.) and ArBr **10k** (0.45 mmol, 1.5 equiv.), compound **14k** was obtained as a yellow oil (24 mg, 19% yield, $rr_{8/9}$ = 2:1) after chromatography on silica gel (pentane/EtOAc, from 10:1 to 6:1).

TLC: 0.3, pentane/EtOAc = 5:1

^{1}H NMR (400 MHz, $CDCl_3$) δ (ppm) = 7.91 (d, $^3J_{H-H}$ = 8.3 Hz, 2H, H-13), 7.35 – 7.27 (m, 2H, H-Ar), 7.32 – 7.14 (m, 5H, H-Ar), 6.01 (t, $^3J_{H-H}$ = 7.3 Hz, 1H, H-3), 5.48 – 5.38 (m, 1H, H-17), 5.13 – 5.03 (m, 1H, H-22), 4.80 (d, $^3J_{H-H}$ = 7.1 Hz, 2H, H-16), 3.96 (s, 2H, H-1), 3.82 – 3.73 (m, 2H, H-5), 2.59 – 2.48 (m, 2H, H-4), 2.17 – 1.98 (m, 4H, H-20 and H-21), 1.74 (s, 3H, H-19), 1.67 (s, 3H, H-24 or H-25), 1.59 (s, 3H, H-24 or H-25), 1.39 (t, $^3J_{H-H}$ = 5.6 Hz, 1H, H-6).

^{13}C{^{1}H} NMR (101 MHz, $CDCl_3$) δ (ppm) = 166.7 (C-15), 145.2 (C-11), 142.4 (C-7 or C-14), 142.3 (C-7-or C-14), 139.9 (C-2), 132.0 (C-23), 129.9 (C-13), 128.53 (C-18), 128.46 (C-Ar), 128.3 (C-Ar), 127.2 (C-Ar), 127.0 (C-3), 126.4 (C-Ar), 123.9 (C-22), 118.6 (C-17), 62.5 (C-5), 61.9 (C-16), 39.7 (C-20 or C-21), 36.2 (C-1), 32.7 (C-4), 26.5 (C-20 or C-21), 25.8 (C-24 or C-25), 17.8 (C-24 or C-25), 16.7 (C-19).

HRMS (ESI +**)**: calculated for $C_{28}H_{35}O_3$ $[M+H]^+$: 436.2847; found: 436.2842.

IR (neat) ν (cm^{-1}) = 3423, 2921, 1714, 1609, 1444, 1269, 1176, 1098, 1018, 938, 754, 696.

(*E*)-5-(4-((((3S,8S,9S,10R,13R,14S,17R)-10,13-dimethyl-17-((R)-6-methylheptan-2-yl)-2,3,4,7,8,9,10,11,12,13,14,15,16,17-tetradecahydro-1H-cyclopenta[a]phenanthren-3-yl)oxy)methyl)phenyl)-4-phenylpent-3-en-1-ol (14l)

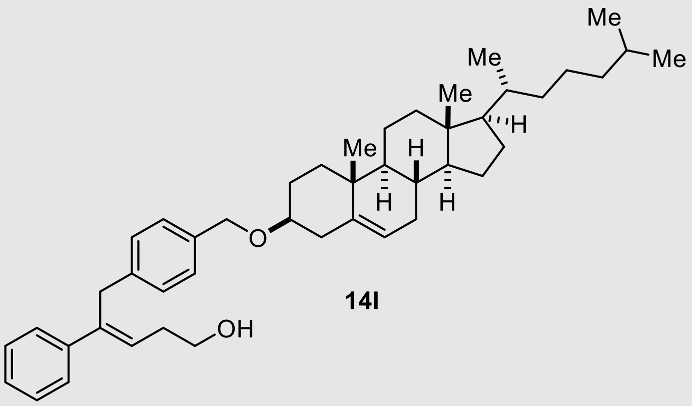

14l

Following the general procedure in **5.1** using boronate **7a** (0.3 mmol, 1.0 equiv.) and ArBr **10l** (0.45 mmol, 1.5 equiv.), compound **14l** was obtained as a yellow solid (107 mg, 56% yield, $rr_{8/9}$ = 11:1) after chromatography on silica gel (pentane/EtOAc, from 10:1 to 5:1).

TLC: 0.2, pentane/EtOAc = 5:1

^{1}H NMR (400 MHz, $CDCl_3$) δ (ppm) = 7.37 – 7.30 (m, 2H), 7.27 – 7.16 (m, 5H), 7.14 (d, $^3J_{H\text{-}H}$ = 8.2 Hz, 2H), 5.98 (t, $^3J_{H\text{-}H}$ = 7.3 Hz, 1H), 5.41 – 5.22 (m, 1H), 4.48 (s, 2H), 3.90 (s, 2H), 3.80-3.71 (m, 2H), 3.25 (tt, $^3J_{H\text{-}H}$ = 11.2, 4.5 Hz, 1H), 2.54 (q, $^3J_{H\text{-}H}$ = 6.7 Hz, 2H), 2.39 (ddd, $^3J_{H\text{-}H}$ = 13.2, 4.8, 2.3 Hz, 1H), 2.31 – 2.19 (m, 1H), 2.05 – 1.75 (m, 5H), 1.63 – 1.20 (m, 14H), 1.20 – 1.04 (m, 6H), 1.03 – 0.97 (m, 5H), 0.91 (d, $^3J_{H\text{-}H}$ = 6.5 Hz, 3H), 0.87 (d, $^3J_{H\text{-}H}$ = 1.8 Hz, 3H), 0.86 (d, $^3J_{H\text{-}H}$ = 1.8 Hz, 3H), 0.67 (s, 3H).

^{13}C{^{1}H} NMR (101 MHz, $CDCl_3$) δ (ppm) = 142.7, 141.2, 140.5, 138.9, 136.8, 128.4, 128.3, 128.0, 127.1, 126.4, 121.7, 78.8, 69.9, 62.6, 56.9, 56.3, 50.4, 42.5, 39.9, 39.7, 39.3, 37.4, 37.1, 36.3, 35.9, 35.8, 32.7, 32.1, 32.0, 28.6, 28.4, 28.2, 24.4, 24.0, 23.0, 22.7, 21.2, 19.5, 18.9, 12.0.

HRMS (ESI +**)**: calculated for $C_{45}H_{65}O_2$ [M+H]$^+$: 637.4980; found: 637.4979.

IR (neat) ν (cm^{-1}) = 3351, 2931, 2866, 1512, 1446, 1375, 1090, 1020, 797, 748, 696.

m.p.: 95-96 °C

(*E*)-4-(5-hydroxy-2-(4-(trifluoromethyl)phenyl)pent-2-en-1-yl)phenyl trifluoromethanesulfonate (14m)

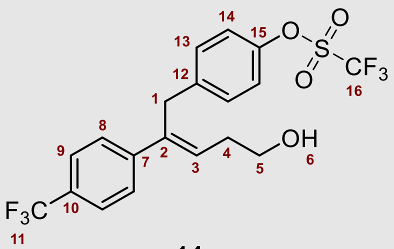

14m

Following the general procedure in **5.1** using boronate **7b** (0.3 mmol, 1.0 equiv.) and 4-bromophenyl trifluoromethanesulfonate **10g** (0.45 mmol, 1.5 equiv.), compound **14m** was obtained as a yellow oil (43.6 mg, 32% yield, *rr*$_{8/9}$ = 10:1) after chromatography on silica gel (pentane/EtOAc, from 5:1 to 2:1).

TLC: 0.2, pentane/EtOAc = 3:1

^{1}H NMR (400 MHz, $CDCl_3$) δ (ppm) = 7.51 (d, $^3J_{H-H}$ = 8.2 Hz, 2H, H-9), 7.41 (d, $^3J_{H-H}$ = 8.9 Hz, 2H, H-8), 7.23 (d, $^3J_{H-H}$ = 8.9 Hz, 2H), 7.14 (d, $^3J_{H-H}$ = 8.8 Hz, 2H), 6.11 (t, $^3J_{H-H}$ = 7.3 Hz, 1H, H-3), 3.94 (s, 2H, H-1), 3.81 (t, $^3J_{H-H}$ = 6.5 Hz, 2H, H-5), 2.55 (q, $^3J_{H-H}$ = 6.6 Hz, 2H, H-4), 1.41 (s, 1H, H-6).

^{13}C{^{1}H} NMRZ (101 MHz, $CDCl_3$) δ (ppm) = 148.1, 145.6, 139.7, 138.5, 129.9, 129.6, 129.5, 129.2, 128.9, 128.8, 127.5, 126.6, 125.6, 125.54, 125.50, 125.46, 122.9, 121.6, 120.5, 117.3, 62.2, 35.2, 32.6.

^{19}F {^{1}H} NMR (282 MHz, $CDCl_3$) δ (ppm) = -62.52 (F-CF_3), -72.91(F-OTf)

HRMS (ESI +**)**: calculated for $C_{19}H_{20}F_6NO_4S$ $[M+NH_4]^+$: 472.1012; found: 472.1010.

IR (neat) ν (cm^{-1}) = 3368, 2931, 1615, 1499, 1420, 1324, 1210, 1121, 1068, 1015, 885, 833, 607.

(*E*)-5-(1-methyl-1H-indol-5-yl)-4-(3,4,5-trimethoxyphenyl)pent-3-en-1-ol (14n)

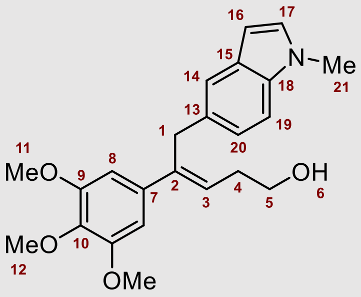

14n

Following the general procedure in **5.1** using boronate **7c** (0.3 mmol, 1.0 equiv.) and 5-bromo-1-methyl-1H-indole **10h** (0.45 mmol, 1.5 equiv.), compound **14n** was obtained as a yellow oil (70.6 mg, 62% yield, *rr*$_{8/9}$ = 20:1) after chromatography on silica gel (pentane/EtOAc, from 5:1 to 2:1 to 1:1).

TLC: 0.2, pentane/EtOAc = 1:1

^{1}H NMR (400 MHz, $CDCl_3$) δ (ppm) = 7.43 (s, 1H), 7.21 (d, $^3J_{H-H}$ = 8.4 Hz, 1H), 7.07 (dd, $^3J_{H-H}$ = 8.4, 1.7 Hz, 1H), 7.00 (d, $^3J_{H-H}$ = 3.1 Hz, 1H), 6.62 (s, 2H, H-8), 6.38 (dd, $^3J_{H-H}$ = 3.1, 0.9 Hz,

1H), 5.96 (t, $^3J_{H-H}$ = 7.3 Hz, 1H, H-3), 3.98 (s, 2H, H-1), 3.85 – 3.71 (m, 14H, H-11, H-12, H-21 and H-5), 2.61 (q, $^3J_{H-H}$ = 6.5 Hz, 2H, H-4), 1.41 (t, $^3J_{H-H}$ = 5.7 Hz, 1H, H-6).

^{13}C{^{1}H} NMR (101 MHz, $CDCl_3$) δ (ppm) = 152.9 (C-9 or C-10), 141.4 (C-2), 139.0 (C-7), 137.3 (C-9 or C-10), 135.6 (C-15 or C-18), 130.5 (C-13), 129.1 (C-Ar), 128.9 (C-15 or C-18), 125.5 (C-3), 122.3 (C-Ar), 120.0 (C-Ar), 109.3 (C-Ar), 103.9 (C-8), 100.7 (C-Ar), 62.6 (C-5), 61.0 (C-11 or C-12 or C-21), 56.2 (C-11 or C-12 or C-21), 36.3 (C-1), 33.0 (C-11 or C-12 or C-21), 32.7 (C-4).

HRMS (ESI +**)**: calculated for $C_{23}H_{28}NO_4$ $[M+H]^+$: 382.2013; found: 382.2030.

IR (neat) ν (cm^{-1}) = 3407, 2935, 1579, 1507, 1410, 1242, 1122, 1005, 910, 724.

(*E*)-4-(2-methoxypyridin-4-yl)-5-(1-methyl-1H-indol-5-yl)pent-3-en-1-ol (14o)

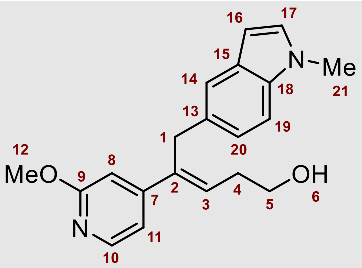

14o

Following the general procedure in **5.1** using boronate **7d** (0.3 mmol, 1.0 equiv.) and 5-bromo-1-methyl-1H-indole **10h** (0.45 mmol, 1.5 equiv.), compound **14o** was obtained as a yellow oil (75 mg, 78% yield, $rr_{8/9}$ >20:1) after chromatography on silica gel (pentane/EtOAc, from 5:1 to 2:1 to 1:1).

TLC: 0.3, pentane/EtOAc = 1:1

^{1}H NMR (400 MHz, $CDCl_3$) δ (ppm) = 7.99 (dd, $^3J_{H-H}$ = 5.5, 0.7 Hz, 1H, H-10), 7.37 (s, 1H, H-14), 7.20 (d, $^3J_{H-H}$ = 8.4 Hz, 1H, H-16), 7.03 (dd, $^3J_{H-H}$ = 8.4, 1.7 Hz, 1H, H-17), 7.00 (d, $^3J_{H-H}$ = 3.1 Hz, 1H, H-20), 6.90 (dd, $^3J_{H-H}$ = 5.5, 1.6 Hz, 1H, H-11), 6.73 (dd, $^3J_{H-H}$ = 1.6, 0.7 Hz, 1H, H-8), 6.37 (dd, $^3J_{H-H}$ = 3.1, 0.9 Hz, 1H, H-19), 6.20 (t, $^3J_{H-H}$ = 7.3 Hz, 1H, H-3), 3.97 (s, 2H, H-1), 3.87 (s, 3H, H-12), 3.79 (q, $^3J_{H-H}$ = 6.6 Hz, 2H, H-5), 3.74 (s, 3H, H-21), 2.61 (q, $^3J_{H-H}$ = 6.7 Hz, 2H, H-4), 1.42 (t, $^3J_{H-H}$ = 5.6 Hz, 1H, H-6).

^{13}C{^{1}H} NMR (101 MHz, $CDCl_3$) δ (ppm) = 164.8 (C-9), 153.1 (C-7), 146.6 (C-10), 139.1 (C-2), 135.7 (C-18), 129.6 (C-13 or C-15), 129.2 (C-3), 129.0 (C-20), 128.9 (C-13 or C-15), 122.2 (C-17), 119.8 (C-14), 114.9 (C-11), 109.4 (C-16), 108.1 (C-8), 100.7 (C-19), 62.4 (C-5), 53.5 (C-12), 35.2 (C-1), 33.0 (C-21), 32.6 (C-4).

HRMS (ESI +**)**: calculated for $C_{20}H_{23}N_2O_2$ $[M+H]^+$: 323.1755; found: 323.1761.

IR (neat) ν (cm^{-1}) = 3351, 2943, 1602, 1543, 1390, 1316, 1244, 1046, 908, 793, 722.

(*E*)-5-(1-benzyl-1H-pyrrolo[2,3-b]pyridin-4-yl)-4-(dibenzo[b,d]furan-2-yl)pent-3-en-1-ol (14p)

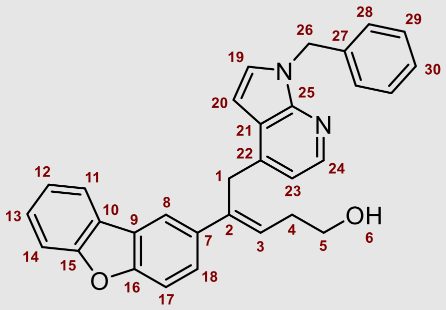

14p

Following the general procedure in **5.1** using boronate **7e** (0.3 mmol, 1.0 equiv.) and 1-benzyl-4-bromo-1H-pyrrolo[2,3-b]pyridine **10i** (0.45 mmol, 1.5 equiv.), compound **14p** was obtained as a yellow foam (84.3 mg, 61% yield, $rr_{8/9}$ = 7:1) after chromatography on silica gel (pentane/EtOAc, from 5:1 to 2:1 to 1:1).

TLC: 0.1, pentane/EtOAc = 2:1

^{1}H NMR (400 MHz, $CDCl_3$) δ (ppm) = 8.20 (d, $^3J_{H-H}$ = 4.9 Hz, 1H, H-24), 7.94 (d, $^3J_{H-H}$ = 1.8 Hz, 1H), 7.86 (d, $^3J_{H-H}$ = 6.6 Hz, 1H), 7.53 (d, $^3J_{H-H}$ = 8.3 Hz, 1H), 7.51 – 7.38 (m, 3H), 7.34 – 7.22 (m, 4H), 7.22 –7.17 (m, 2H), 7.16 (d, $^3J_{H-H}$ = 3.7 Hz, 1H, H-19), 6.92 (d, $^3J_{H-H}$ = 5.0 Hz, 1H, H-23), 6.58 (d, $^3J_{H-H}$ = 3.5 Hz, 1H, H-20), 6.17 (t, $^3J_{H-H}$ = 7.2 Hz, 1H, H-3), 5.48 (s, 2H, H-26), 4.27 (s, 2H, H-1), 3.82 (s, 2H, H-5), 2.59 (q, $^3J_{H-H}$ = 6.7 Hz, 2H, H-4), 1.62 (s, 1H, H-6).

^{13}C{^{1}H} NMR (101 MHz, $CDCl_3$) δ (ppm) = 156.7 (C-15 or C-16), 155.6 (C-15 or C-16), 147.8 (C-25), 143.6 (C-24), 141.4 (C-22), 138.9 (C-2), 137.9 (C-9 or C-10), 137.8 (C-7), 128.8 (C-Ar), 127.7 (C-Ar), 127.6 (C-Ar), 127.43 (C-Ar), 127.38 (C-Ar), 127.3 (C-3), 125.8 (C-Ar), 124.4 (C-9 or C-10), 122.8 (C-Ar), 120.8 (C-Ar), 120.2 (C-21), 118.4 (C-Ar), 115.1 (C-23), 111.8 (C-Ar), 111.4 (C-Ar), 98.6 (C-20), 62.5 (C-5), 48.1 (C-26), 33.7 (C-1), 32.9 (C-4).

HRMS (ESI +**)**: calculated for $C_{31}H_{27}N_2O_2$ $[M+H]^+$: 459.2068; found: 459.2080.

IR (neat) ν (cm^{-1}) = 3351, 2922, 1584, 1449, 1346, 1195, 1022, 813, 749, 718, 623.

m.p.: 118.2-118.9 °C

(*E*)-4-(3,5-dimethylphenyl)-5-(2-methoxyphenyl)pent-3-en-1-ol (14q)

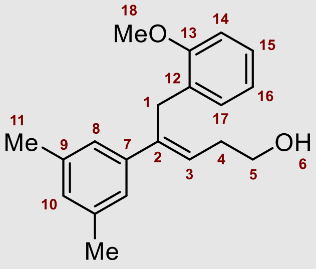

14q

Following the general procedure in **5.1** using boronate **7f** (0.3 mmol, 1.0 equiv.) and 1-bromo-2-methoxybenzene **10c** (0.45 mmol, 1.5 equiv.), compound **14q** was obtained as a red oil (54.9 mg, 62% yield, $rr_{8/9}$ = 17:1) after chromatography on silica gel (pentane/EtOAc, from 10:1 to 6:1).

TLC: 0.2, pentane/EtOAc = 5:1

^{1}H NMR (400 MHz, $CDCl_3$) δ (ppm) = 7.15 (ddd, $^3J_{H\text{-}H}$ = 8.2, 7.4, 1.7 Hz, 1H, H-15), 7.06 (ddt, $^3J_{H\text{-}H}$ = 7.5, 1.8, 1.0 Hz, 1H, H-17), 7.00 (s, 2H, H-8), 6.87 – 6.77 (m, 3H, H-10, H-14 and H-16), 5.99 (t, $^3J_{H\text{-}H}$ = 7.3 Hz, 1H, H-3), 3.86 (s, 3H, H-18), 3.85 (s, 2H, H-1), 3.73 (q, $^3J_{H\text{-}H}$ = 6.5 Hz, 2H, H-5), 2.46 (q, $^3J_{H\text{-}H}$ = 6.7 Hz, 2H, H-4), 2.26 (s, 6H, H-11), 1.39 (t, $^3J_{H\text{-}H}$ = 5.8 Hz, 1H, H-6).

^{13}C{^{1}H} NMR (101 MHz, $CDCl_3$) δ (ppm) = 157.4 (C-13), 143.0 (C-7), 140.3 (C-2), 137.7 (C-9), 128.70 (C-Ar), 128.68 (C-Ar), 128.0 (C-12), 127.1 (C-15), 126.3 (C-3), 124.2 (C-8), 120.6 (C-Ar), 110.0 (C-Ar), 62.6 (C-5), 55.4 (C-18), 32.6 (C-4), 29.7 (C-1), 21.5 (C-11).

HRMS (ESI +**)**: calculated for $C_{20}H_{25}O_2$ $[M+H]^+$: 297.1850; found: 297.1843.

IR (neat) ν (cm^{-1}) = 3341, 2916, 1598, 1489, 1461, 1238, 1105, 1029, 845, 750.

5.2 General procedure for C3 arylation

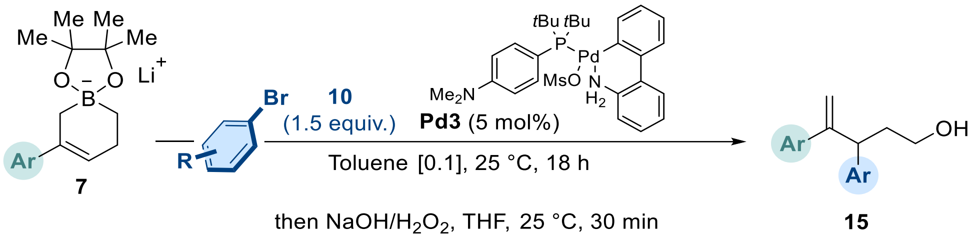

Figure S5. Pd-catalyzed C3-selective arylation of cyclic allylboronates

In a glovebox, to an oven dried screw capped Schlenk equipped with a magnetic stir bar, **Pd3** [APhosPdG3] (11.1 mg, 5 mol%) and allylboronate **7** (0.3 mmol, 1.0 equiv.) were dissolved in 3 mL of toluene. Subsequently, ArBr (0.45 mmol, 1.5 equiv.) was added. The system was closed, and the mixture was stirred at 25 °C. After 18 h, the mixture was filtered on Celite, washed with Et_2O (3 × 5 mL), and the solution was concentrated under vacuum. The alkali oxidation was conducted next. The crude reaction mixture was dissolved in THF (3.0 mL), and an aqueous NaOH solution (1.5 mL, 4 M) and 30% aq. H_2O_2 solution (1.5 mL) were added at room temperature. After 30 min., at 25 °C, the reaction mixture was extracted with CH_2Cl_2 (3 × 10 mL). The combined organic phases were dried over Na_2SO_4 and concentrated under reduced pressure after filtration. The residue was purified by flash column chromatography on silica gel using pentane/EtOAc as eluent to afford the homoallylic alcohol **15**.

3,4-diphenylpent-4-en-1-ol (15a)

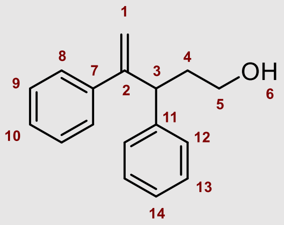

15a

Following the general procedure in **5.2** using boronate **7a** (0.3 mmol, 1.0 equiv.) and PhBr **10a** (0.45 mmol, 1.5 equiv.), compound **15a** was obtained as a yellow oil (44.3 mg, 62% yield, $rr_{9/8}$ = 9:1) after chromatography on silica gel (pentane/EtOAc, from 10:1 to 6:1).

TLC: 0.3, pentane/EtOAc = 5:1

^{1}H NMR (400 MHz, $CDCl_3$) δ (ppm) = 7.30 – 7.14 (m, 10H, H-Ar), 5.41 (s, 1H, H-1), 5.21 (s, 1H, H-1), 4.07-3.98 (m, 1H, H-3), 3.71 – 3.52 (m, 2H, H-5), 2.21 (ddt, $^3J_{H-H}$ = 13.7, 7.2, 6.5 Hz, 1H, H-4), 2.05 (ddt, $^3J_{H-H}$ = 13.7, 9.0, 6.0 Hz, 1H, H-4), 1.33 (s, 1H, H-6).

^{13}C{^{1}H} NMR (101 MHz, $CDCl_3$) δ (ppm) = 151.65 (C-2), 142.83 (C-11), 142.44 (C-7), 128.58 (C-Ar), 128.27 (C-Ar), 128.22 (C-Ar), 127.36 (C-Ar), 126.93 (C-Ar), 126.55 (C-Ar), 113.47 (C-1), 61.17 (C-5), 46.59 (C-3), 37.84 (C-4).

HRMS (ESI +**)**: calculated for $C_{17}H_{18}ONa$ $[M+Na]^+$: 261.1250; found: 261.1248.

IR (neat) ν (cm^{-1}) = 3340, 2924, 1625, 1492, 1451, 1027, 903, 777, 734, 697.

HPLC: OJ, 10% *i*PrOH in *n*-hexane, 1.0 mL/min., λ = 205 nm.

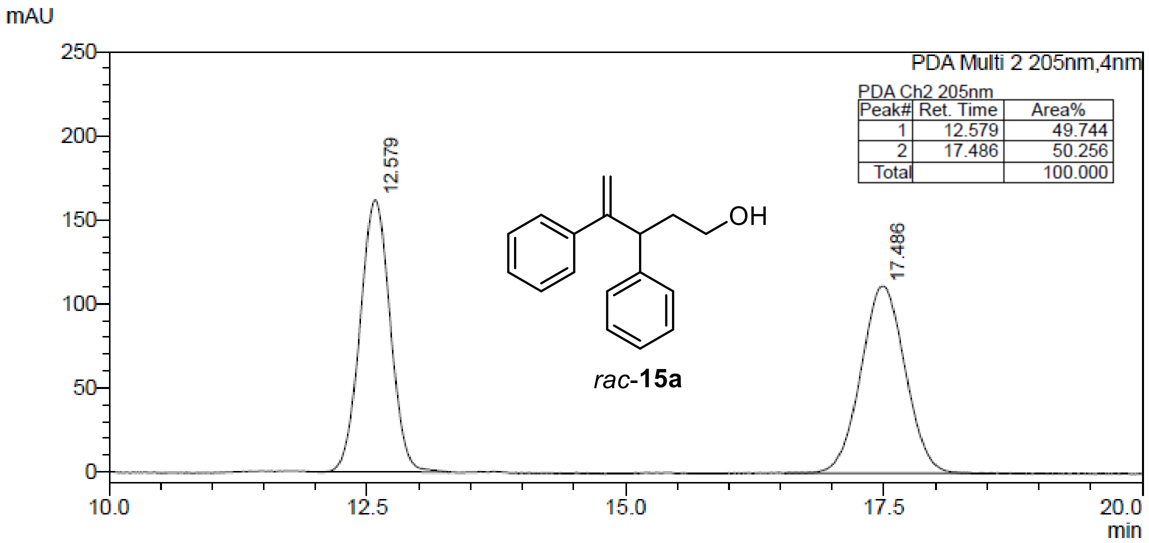

28:72 *er* obtained when using (*R*,*R*)-**L15:**

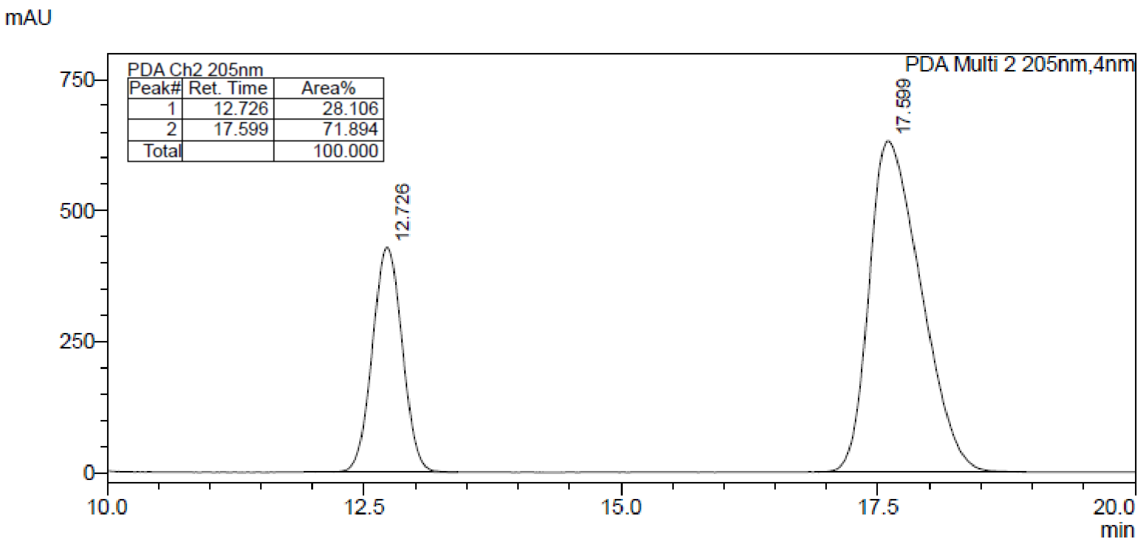

3-(4-(dimethylamino)phenyl)-4-phenylpent-4-en-1-ol (15b)

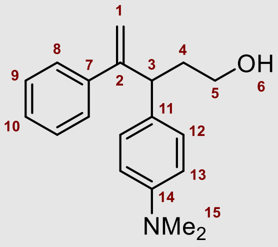

15b

Following the general procedure in **5.2** using boronate **7a** (0.3 mmol, 1.0 equiv.) and 4-bromo-N,N-dimethylaniline **10m** (0.45 mmol, 1.5 equiv.), compound **15b** was obtained as a yellow oil (30 mg, 36% yield, $rr_{9/8}$ = 7:1) after chromatography on silica gel (pentane/EtOAc, from 10:1 to 6:1).

TLC: 0.3, pentane/EtOAc = 3:1

^{1}H NMR (400 MHz, $CDCl_3$) δ (ppm) = 7.32 – 7.25 (m, 2H, H-8), 7.28 – 7.16 (m, 3H, H-9 and H-10), 7.12 (d, $^{3}J_{H\text{-}H}$ = 8.7 Hz, 2H, H-12), 6.67 (d, $^{3}J_{H\text{-}H}$ = 8.7 Hz, 2H, H-13), 5.36 (s, 1H, H-1), 5.16 (s, 1H, H-1), 3.91 (dd, $^{3}J_{H\text{-}H}$ = 9.1, 6.1 Hz, 1H, H-3), 3.64 (brs, 2H, H-5), 2.91 (s, 6H, H-15), 2.24 – 2.09 (m, 1H, H-4), 2.08 – 1.94 (m, 1H, H-4), 1.20 (s, 1H, H-6).

^{13}C{^{1}H} NMR (101 MHz, $CDCl_3$) δ (ppm) = 152.3 (C-2), 149.4 (C-14), 142.7 (C-7), 130.6 (C-11), 128.9 (C-12), 128.2 (C-9 or C-10), 127.2 (C-9 or C-10), 127.0 (C-8), 113.0 (C-1), 112.9 (C-13), 61.6 (C-5), 45.8 (C-3), 40.8 (C-15), 38.0 (C-4).

HRMS (ESI +**)**: calculated for $C_{19}H_{23}NONa$ $[M+Na]^+$: 304.1672; found: 304.1676.

IR (neat) ν (cm^{-1}) = 3402, 2930, 1612, 1519, 1443, 1349, 1224, 1165, 1032, 891, 813, 777, 699, 555.

3-(4-methoxyphenyl)-4-phenylpent-4-en-1-ol (15c)

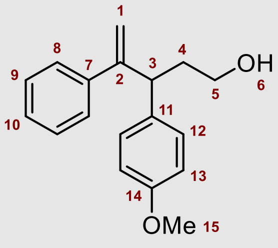

15c

Following the general procedure in **5.2** using boronate **7a** (0.3 mmol, 1.0 equiv.) and 1-bromo-4-methoxybenzene **10b** (0.45 mmol, 1.5 equiv.), compound **15c** was obtained as a yellow oil (50 mg, 61% yield, $rr_{9/8}$ = 7:1) after chromatography on silica gel (pentane/EtOAc, from 6:1 to 3:1).

TLC: 0.3, pentane/EtOAc = 2:1

^{1}H NMR (400 MHz, $CDCl_3$) δ (ppm) = 7.33 – 7.20 (m, 5H, H-8, H-9 and H-10), 7.19 (d, $^{3}J_{H\text{-}H}$ = 8.7 Hz, 2H, H-12), 6.84 (d, $^{3}J_{H\text{-}H}$ = 8.7 Hz, 2H, H-13), 5.40 (s, 1H, H-1), 5.21 (s, 1H, H-1), 4.00 (ddd, $^{3}J_{H\text{-}H}$ = 9.3, 6.1, 1.1 Hz, 1H, H-3), 3.79 (s, 3H, H-15), 3.74 – 3.63 (m, 1H, H-5), 3.66 – 3.56 (m, 1H, H-5), 2.27 – 2.14 (m, 1H, H-4), 2.10 – 1.95 (m, 1H, H-4), 1.31 (s, 1H, H-6).

$^{13}C\{^{1}H\}$ NMR (101 MHz, $CDCl_3$) δ (ppm) = 158.2 (C-14), 152.0 (C-2), 142.5 (C-7), 134.8 (C-11), 129.2 (C-12), 128.2 (C-Ar), 127.3 (C-Ar), 126.9 (C-Ar), 114.0 (C-13), 113.2 (C-1), 61.2 (C-5), 55.3 (C-15), 45.8 (C-3), 37.9 (C-4).

HRMS (ESI +**)**: calculated for $C_{18}H_{21}O_2$ $[M+H]^+$: 269.1537; found: 269.1538.

IR (neat) ν (cm^{-1}) = 3351, 2933, 1608, 1508, 1244, 1176, 1028, 900, 829, 777, 700, 560.

3-([1,1'-biphenyl]-4-yl)-4-phenylpent-4-en-1-ol (15d)

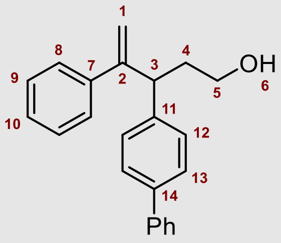

15d

Following the general procedure in **5.2** using boronate **7a** (0.3 mmol, 1.0 equiv.) and 4-bromo-1,1'-biphenyl **10n** (0.45 mmol, 1.5 equiv.), compound **15d** was obtained as a yellow oil (45 mg, 48% yield, $rr_{9/8}$ = 4:1) after chromatography on silica gel (pentane/EtOAc, from 10:1 to 6:1).

TLC: 0.3, pentane/EtOAc = 5:1

^{1}H NMR (400 MHz, $CDCl_3$) δ (ppm) = 7.54 – 7.47 (m, 2H, H-Ar), 7.49 – 7.41 (m, 2H, H-Ar), 7.40 – 7.31 (m, 2H, H-Ar), 7.31 – 7.21 (m, 5H, H-Ar), 7.23 – 7.11 (m, 3H, H-Ar), 5.38 (s, 1H, H-1), 5.18 (s, 1H, H-1), 4.07 – 3.99 (m, 1H, H-3), 3.69 – 3.52 (m, 2H, H-5), 2.18 (dq, $^{3}J_{H\text{-}H}$ = 13.5, 6.6 Hz, 1H, H-4), 2.02 (ddt, $^{3}J_{H\text{-}H}$ = 13.7, 8.9, 6.0 Hz, 1H, H-4), 0.82 (s, 1H, H-6).

$^{13}C\{^{1}H\}$ NMR (101 MHz, $CDCl_3$) δ (ppm) = 151.6 (C-2), 142.4 (C-7), 142.0 (C-11), 141.0 (C-Ar), 139.4 (C-Ar), 128.8 (C-Ar), 128.7 (C-Ar), 128.3 (C-Ar), 127.4 (C-Ar), 127.3 (C-Ar), 127.2 (C-Ar), 127.1 (C-Ar), 127.0 (C-Ar), 113.6 (C-1), 61.2 (C-5), 46.2 (C-3), 37.9 (C-4).

HRMS (ESI +**)**: calculated for $C_{23}H_{23}O$ $[M+H]^+$: 315.1744; found: 315.1741.

IR (neat) ν (cm^{-1}) = 3370, 2921, 1624, 1484, 1363, 1035, 905, 835, 760, 696, 574.

3-(4-chlorophenyl)-4-phenylpent-4-en-1-ol (15e)

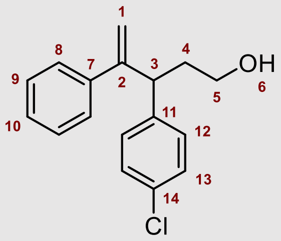

15e

Following the general procedure in **5.2** using boronate **7a** (0.3 mmol, 1.0 equiv.) and 1-bromo-4-chlorobenzene **10d** (0.45 mmol, 1.5 equiv.), compound **15e** was obtained as a yellow oil (30 mg, 37% yield, $rr_{9/8}$ = 3:1) after chromatography on silica gel (pentane/EtOAc, from 10:1 to 6:1).

TLC: 0.3, pentane/EtOAc = 5:1

^{1}H NMR (400 MHz, $CDCl_3$) δ (ppm) = 7.33 – 7.16 (m, 9H, H-Ar), 5.43 (s, 1H, H-1), 5.22 (s, 1H, H-1), 4.06 (ddd, $^3J_{H\text{-}H}$ = 9.0, 6.2, 1.2 Hz, 1H, H-3), 3.73 – 3.62 (m, 1H, H-5), 3.63 – 3.52 (m, 1H, H-5), 2.30 – 2.15 (m, 1H, H-4), 2.07 – 1.94 (m, 1H, H-4), 1.29 (s, 1H, H-6).

^{13}C{^{1}H} NMR (101 MHz, $CDCl_3$) δ (ppm) = 151.3 (C-2), 142.1 (C-7), 141.4 (C-11), 132.2 (C-14), 129.6 (C-12), 128.7 (C-Ar), 128.3 (C-Ar), 127.5 (C-Ar), 126.9 (C-Ar), 113.6 (C-1), 60.8 (C-5), 45.8 (C-3), 37.6 (C-4).

HRMS (ESI +**)**: calculated for $C_{17}H_{17}ClONa$ $[M+Na]^+$: 295.0861; found: 295.0870.

IR (neat) ν (cm^{-1}) = 3330, 2928, 1625, 1489, 1407, 1090, 1013, 902, 826, 776, 697.

methyl 4-(5-hydroxy-2-phenylpent-1-en-3-yl)benzoate (15f)

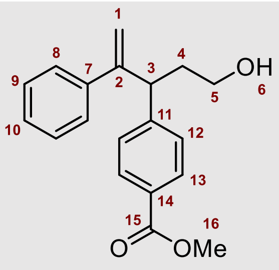

15f

Following the general procedure in **5.2** using boronate **7a** (0.3 mmol, 1.0 equiv.) and methyl 4-bromobenzoate **10e** (0.45 mmol, 1.5 equiv.), compound **15f** was obtained as a yellow oil (50 mg, 54% yield, $rr_{9/8}$ = 4:1) after chromatography on silica gel (pentane/EtOAc, from 10:1 to 6:1).

TLC: 0.3, pentane/EtOAc = 5:1

^{1}H NMR (400 MHz, $CDCl_3$) δ (ppm) = 7.96 (d, $^3J_{H\text{-}H}$ = 8.4 Hz, 2H, H-13), 7.35 (d, $^3J_{H\text{-}H}$ = 8.4 Hz, 2H, H-12), 7.31 – 7.18 (m, 5H, H-8, H-9 and H-10), 5.46 (s, 1H, H-1), 5.25 (s, 1H, H-1), 4.20 – 4.11 (m, 1H, H-3), 3.91 (s, 3H, H-16), 3.74 – 3.64 (m, 1H, H-5), 3.64 – 3.53 (m, 1H, H-5), 2.26 (td, $^3J_{H\text{-}H}$ = 13.8, 6.4 Hz, 1H, H-4), 2.07 (ddt, $^3J_{H\text{-}H}$ = 13.7, 8.9, 5.9 Hz, 1H, H-4), 1.53 (s, 1H, H-6).

^{13}C{^{1}H} NMR (101 MHz, $CDCl_3$) δ (ppm) = 167.2 (C-15), 151.0 (C-2), 148.5 (C-11), 142.0 (C-7), 129.9 (C-13), 128.5 (C-14), 128.32 (C-Ar), 128.29 (C-Ar), 127.5 (C-Ar), 126.9 (C-Ar), 113.9 (C-1), 60.7 (C-5), 52.1 (C-16), 46.4 (C-3), 37.5 (C-4).

HRMS (ESI +**)**: calculated for $C_{19}H_{20}O_3Na$ $[M+Na]^+$: 319.1305; found: 319.1313.

IR (neat) ν (cm^{-1}) = 3388, 2953, 1718, 1607, 1434, 1277, 1179, 1110, 1018, 903, 776, 704.

4-phenyl-3-(4-(trifluoromethyl)phenyl)pent-4-en-1-ol (15g)

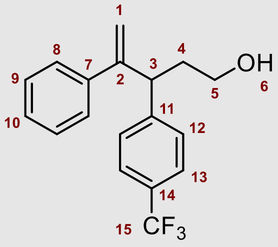

15g

Following the general procedure in **5.2** using boronate **7a** (0.3 mmol, 1.0 equiv.) and 1-bromo-4-(trifluoromethyl)benzene **10o** (0.45 mmol, 1.5 equiv.), compound **15g** was obtained as a yellow oil (41 mg, 45% yield, *rr*$_{9/8}$ = 3:1) after chromatography on silica gel (pentane/EtOAc, from 10:1 to 6:1).

TLC: 0.2, pentane/EtOAc = 5:1

^{1}H NMR (400 MHz, $CDCl_3$) δ (ppm) = 7.52 (d, $^3J_{H\text{-}H}$ = 8.0 Hz, 2H, H-13), 7.38 (d, $^3J_{H\text{-}H}$ = 8.1 Hz, 2H, H-12), 7.31 – 7.16 (m, 5H, H-8, H-9 and H-10), 5.46 (s, 1H, H-1), 5.24 (s, 1H, H-1), 4.16 (t, $^3J_{H\text{-}H}$ = 7.6 Hz, 1H, H-3), 3.75 – 3.62 (m, 1H, H-5), 3.62 – 3.49 (m, 1H, H-5), 2.25 (td, $^3J_{H\text{-}H}$ = 13.8, 6.4 Hz, 1H, H-4), 2.10 – 1.97 (m, 1H, H-4), 1.36 (s, 1H, H-6).

^{13}C{^{1}H} NMR (101 MHz, $CDCl_3$) δ (ppm) = 150.9 (C-2), 147.1 (C-11), 142.0 (C-7), 128.8 (q, $^2J_{C\text{-}F}$ =32.3, C-14), 128.6 (C-12), 128.4 (C-Ar), 127.6 (C-Ar), 126.9 (C-Ar), 125.5 (q, $^3J_{C\text{-}F}$ = 4.0 Hz, C-13), 124.4 (q, $^1J_{C\text{-}F}$ = 272.7, C-15) 114.0 (C-1), 60.7 (C-5), 46.2 (C-3), 37.6 (C-4).

^{19}F{^{1}H} NMR (282 MHz, $CDCl_3$) δ (ppm) = –62.3

HRMS (ESI +**)**: calculated for $C_{18}H_{17}F_3ONa$ $[M+Na]^+$: 329.1124; found: 329.1099.

IR (neat) ν (cm^{-1}) = 3326, 2937, 1617, 1322, 1162, 1114, 1067, 1017, 905, 838, 777, 698, 610.

4-(5-hydroxy-2-phenylpent-1-en-3-yl)phenyl trifluoromethanesulfonate (15h)

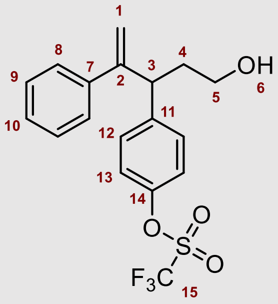

15h

Following the general procedure in **5.2** using boronate **7a** (0.3 mmol, 1.0 equiv.) and 4-bromophenyl trifluoromethanesulfonate **10g** (0.45 mmol, 1.5 equiv.), compound **15h** was obtained as a yellow oil (50 mg, 43% yield, *rr*$_{9/8}$ = 1.5:1) after chromatography on silica gel (pentane/EtOAc, from 10:1 to 6:1).

TLC: 0.2, pentane/EtOAc = 5:1

^{1}H NMR (400 MHz, $CDCl_3$) δ (ppm) = 7.32 (d, J = 8.7 Hz, 2H, H-12), 7.27 – 7.19 (m, 5H, H-8, H-9 and H-10), 7.16 (d, J = 8.8 Hz, 2H, H-13), 5.43 (s, 1H, H-1), 5.21 (s, 1H, H-1), 4.12 (t, J =

7.3 Hz, 1H, H-3), 3.74 – 3.63 (m, 1H, H-5), 3.62 – 3.50 (m, 1H, H-5), 2.33 – 2.14 (m, 1H, H-4), 2.08 – 1.91 (m, 1H, H-4), 1.26 (s, 1H, H-6).

^{13}C{^{1}H} NMR (101 MHz, $CDCl_3$) δ (ppm) = 151.0 (C-2), 148.2 (C-14), 143.6 (C-11), 141.9 (C-7), 130.0 (C-12), 128.4 (C-8 or C-9 or C-10), 127.7 (C-8 or C-9 or C-10), 126.9 (C-8 or C-9 or C-10), 121.4 (C-13), 118.9 (q, $^{1}J_{C\text{-}F}$ = 322, C-15), 114.0 (C-1), 60.7 (C-5), 45.8 (C-3), 37.6 (C-4).

^{19}F{^{1}H} NMR (282 MHz, $CDCl_3$) δ (ppm) = –72.9.

HRMS (ESI +**)**: calculated for $C_{18}H_{21}F_3NO_4S$ $[M+NH_4]^+$: 404.1138; found: 404.1123.

IR (neat) ν (cm^{-1}) = 3358, 2936, 1497, 1417, 1249, 1206, 1135, 1016, 884, 838, 778, 700.

1-(4-(5-hydroxy-2-phenylpent-1-en-3-yl)phenyl)-2,2-dimethylpropan-1-one (15i)

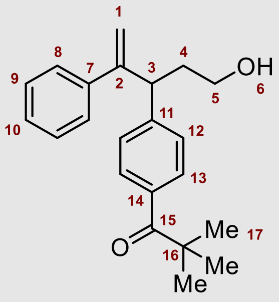

15i

Following the general procedure in **5.2** using boronate **7a** (0.3 mmol, 1.0 equiv.) and 1-(4-bromophenyl)-2,2-dimethylpropan-1-one **10r** (0.45 mmol, 1.5 equiv.), compound **15i** was obtained as a yellow oil (41.6 mg, 43% yield, $rr_{9/8}$ = 3.6:1) after chromatography on silica gel (pentane/EtOAc, from 10:1 to 6:1).

TLC: 0.3, pentane/EtOAc = 5:1

^{1}H NMR (400 MHz, $CDCl_3$) δ (ppm) = 7.65 (d, $^{3}J_{H\text{-}H}$ = 8.4 Hz, 2H, H-13), 7.29 (d, $^{3}J_{H\text{-}H}$ = 8.2 Hz, 2H, H-12), 7.29 – 7.17 (m, 5H, H-8, H-9 and H-10), 5.44 (s, 1H, H-1), 5.22 (s, 1H, H-1), 4.14 – 4.04 (m, 1H, H-3), 3.73 – 3.62 (m, 1H, H-5), 3.64 – 3.52 (m, 1H, H-5), 2.22 (ddt, $^{3}J_{H\text{-}H}$ = 13.7, 7.3, 6.5 Hz, 1H, H-4), 2.04 (ddt, $^{3}J_{H\text{-}H}$ = 13.8, 8.8, 5.9 Hz, 1H, H-4), 1.33 (s, 9H, H-17), 1.25 (s, 1H, H-6).

^{13}C{^{1}H} NMR (101 MHz, $CDCl_3$) δ (ppm) = 208.6 (C-15), 151.0 (C-2), 146.3 (C-11), 142.1 (C-7), 136.6 (C-14), 128.6 (C-8 or C-9 or C-10), 128.3 (C-13), 128.0 (C-12), 127.5 (C-8 or C-9 or C-10), 126.9 (C-8 or C-9 or C-10), 113.9 (C-1), 60.9 (C-5), 46.3 (C-3), 44.2 (C-16), 37.6 (C-4), 28.3 (C-17).

HRMS (ESI +**)**: calculated for $C_{22}H_{27}O_2$ $[M+H]^+$: 323.2006; found: 323.2014.

IR (neat) ν (cm^{-1}) = 3401, 2960, 1668, 1603, 1476, 1276, 1170, 1038, 959, 902, 844, 777, 698, 581.

3-(3-methoxyphenyl)-4-phenylpent-4-en-1-ol (15j)

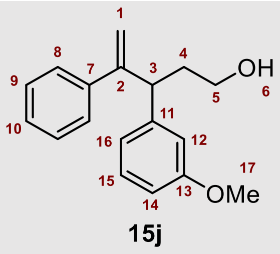

Following the general procedure in **5.2** using boronate **7a** (0.3 mmol, 1.0 equiv.) and 1-bromo-3-methoxybenzene **10q** (0.45 mmol, 1.5 equiv.), compound **15j** was obtained as a red oil (50 mg, 62% yield, $rr_{9/8}$ = 8:1) after chromatography on silica gel (pentane/EtOAc, from 10:1 to 6:1).

TLC: 0.3, pentane/EtOAc = 5:1

^{1}H NMR (400 MHz, $CDCl_3$) δ (ppm) = 7.36 – 7.16 (m, 6H, H-8, H-9, H-10 and H-15), 6.89 (d, $^3J_{H\text{-}H}$ = 7.7 Hz, 1H, H-16), 6.84 (dd, $^3J_{H\text{-}H}$ = 2.6, 1.6 Hz, 1H, H-12), 6.75 (ddd, $^3J_{H\text{-}H}$ = 8.1, 2.6, 0.9 Hz, 1H, H-14), 5.44 (s, 1H, H-1), 5.24 (s, 1H, H-1), 4.07 – 3.98 (m, 1H, H-3), 3.79 (s, 3H, H-17), 3.74 – 3.55 (m, 2H, H-5), 2.28 – 2.15 (m, 1H, H-4), 2.13 – 1.99 (m, 1H, H-4), 1.37 (s, 1H, H-6).

^{13}C{^{1}H} NMR (101 MHz, $CDCl_3$) δ (ppm) = 159.8 (C-13), 151.4 (C-2), 144.6 (C-11), 142.4 (C-7), 129.5 (C-15), 128.2 (C-8 or C-9 or C-10), 127.4 (C-8 or C-9 or C-10), 126.9 (C-8 or C-9 or C-10), 120.8 (C-16), 114.2 (C-12), 113.5 (C-1), 111.6 (C-14), 61.2 (C-5), 55.2 (C-17), 46.6 (C-3), 37.8 (C-4).

HRMS (ESI +**)**: calculated for $C_{18}H_{20}O_2Na$ $[M+Na]^+$: 291.1356; found: 291.1357.Z

IR (neat) ν (cm^{-1}) = 3350, 2938, 1597, 1583, 1485, 1259, 1147, 1037, 902, 777, 697.

3-(2-methoxyphenyl)-4-phenylpent-4-en-1-ol (15k)

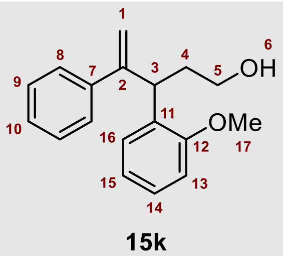

Following the general procedure in **5.2** using boronate **7a** (0.3 mmol, 1.0 equiv.) and 1-bromo-2-methoxybenzene **10c** (0.45 mmol, 1.5 equiv.), compound **15k** was obtained as a red oil (55 mg, 68% yield, $rr_{9/8}$ = 6:1) after chromatography on silica gel (pentane/EtOAc, from 10:1 to 6:1).

TLC: 0.2, pentane/EtOAc = 5:1

^{1}H NMR (400 MHz, $CDCl_3$) δ (ppm) = 7.38 – 7.31 (m, 2H, H-Ar), 7.26 – 7.10 (m, 5H, H-Ar), 6.93 – 6.81 (m, 2H, H-Ar), 5.48 (s, 1H, H-1), 5.23 (s, 1H, H-1), 4.61 (dd, $^3J_{H\text{-}H}$ = 10.3, 5.0 Hz, 1H, H-3), 3.86 (s, 3H, H-17), 3.69 – 3.55 (m, 1H, H-5), 3.51 – 3.36 (m, 1H, H-5), 2.33 – 2.18 (m, 1H, H-4), 2.09 (s, 1H, H-6), 1.96 – 1.83 (m, 1H, H-4).

^{13}C{^{1}H} NMR (101 MHz, $CDCl_3$) δ (ppm) = 157.1 (C-12), 151.5 (C-2), 142.4 (C-7), 131.0 (C-11), 128.20 (C-Ar), 128.15 (C-Ar), 127.6 (C-Ar), 127.3 (C-Ar), 126.6 (C-Ar), 121.4 (C-Ar), 112.9 (C-1), 110.8 (C-Ar), 61.0 (C-5), 55.9 (C-17), 37.9 (C-4), 36.9 (C-3).

HRMS (ESI +**)**: calculated for $C_{18}H_{20}O_2Na$ $[M+Na]^+$: 291.1356; found: 291.1357.

IR (neat) ν (cm^{-1}) = 3350, 2937, 1597, 1489, 1462, 1240, 1026, 898, 751, 701, 586.

3-(4-((((1R,2S,5R)-2-isopropyl-5-methylcyclohexyl)oxy)methyl)phenyl)-4-phenylpent-4-en-1-ol (15l)

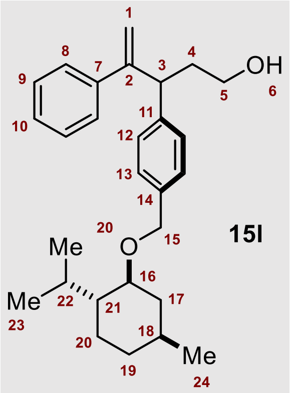

Following the general procedure in **5.2** using boronate **7a** (0.3 mmol, 1.0 equiv.) and ArBr **10s** (0.45 mmol, 1.5 equiv.), compound **15l** was obtained as a yellow oil (80 mg, 66% yield, $rr_{9/8}$ = 6:1, *dr* = 1:1) after chromatography on silica gel (pentane/EtOAc, from 10:1 to 6:1).

TLC: 0.3, pentane/EtOAc = 5:1

^{1}H NMR (400 MHz, $CDCl_3$) δ (ppm) = 7.31 – 7.13 (m, 9H, H-Ar), 5.40 (s, 1H, H-1), 5.20 (s, 1H, H-1), 4.60 (d, $^3J_{H\text{-}H}$ = 11.6 Hz, 1H), 4.34 (d, $^3J_{H\text{-}H}$ = 11.4 Hz, 1H), 4.07 – 3.98 (m, 1H), 3.71 – 3.47 (m, 2H), 3.13 (td, $^3J_{H\text{-}H}$ = 10.5, 4.1 Hz, 1H), 2.33 – 2.11 (m, 3H), 2.08 – 1.94 (m, 1H), 1.70 – 1.56 (m, 2H), 1.43 – 1.13 (m, 3H), 1.03 – 0.77 (m, 9H), 0.65 (dd, $^3J_{H\text{-}H}$ = 6.9, 3.7 Hz, 3H).

^{13}C{^{1}H} NMR (101 MHz, $CDCl_3$) δ (ppm) = 151.6, 142.4, 142.08, 142.07, 137.3, 128.32, 128.30, 128.24, 128.22, 127.4, 126.93, 126.92, 113.44, 113.40, 78.8, 70.4, 61.2, 48.4, 46.28, 46.26, 40.5, 38.0, 34.7, 31.7, 25.6, 23.3, 22.5, 21.2, 16.13, 16.12.

HRMS (ESI +**)**: calculated for $C_{28}H_{42}O_2N$ $[M+NH_4]^+$: 424.3211; found: 424.3240.

IR (neat) ν (cm^{-1}) = 3349, 2920, 2867, 1623, 1454, 1368, 1050, 900, 776, 700.

4-(benzo[d][1,3]dioxol-5-yl)-3-(4-((((3S,8S,9S,10R,13R,14S,17R)-10,13-dimethyl-17-((R)-6-methylheptan-2-yl)-2,3,4,7,8,9,10,11,12,13,14,15,16,17-tetradecahydro-1H-cyclopenta[a]phenanthren-3-yl)oxy)methyl)phenyl)pent-4-en-1-ol (15m)

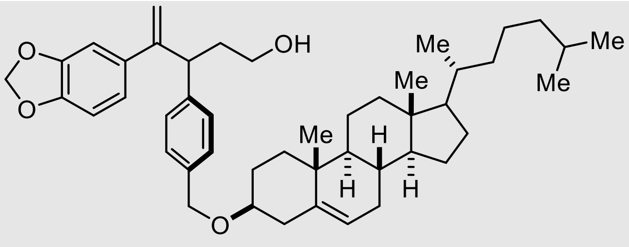

15m

Following the general procedure in **5.2** using boronate **7g** (0.3 mmol, 1.0 equiv.) and ArBr **10l** (0.45 mmol, 1.5 equiv.), compound **15m** was obtained as a white solid (110 mg, 54% yield, *rr*$_{9/8}$ = 2:1, *dr* = 1:1) after chromatography on silica gel (pentane/EtOAc, from 10:1 to 5:1).

TLC: 0.2, pentane/EtOAc = 3:1

^{1}H NMR (400 MHz, $CDCl_3$) δ (ppm) = 7.33 – 7.22 (m, 3H), 6.84 – 6.77 (m, 2H), 6.70 (d, $^3J_{H-H}$ = 8.6 Hz, 2H), 5.94 (s, 2H), 5.42 – 5.33 (m, 2H), 5.18 (s, 1H), 4.54 (s, 2H), 3.99 (dd, $^3J_{H-H}$ = 9.2, 5.9 Hz, 1H), 3.72 – 3.54 (m, 2H), 3.31 (ddd, $^3J_{H-H}$ = 15.8, 11.2, 4.5 Hz, 1H), 2.46 (ddd, $^3J_{H-H}$ = 13.3, 4.8, 2.3 Hz, 1H), 2.37 – 2.27 (m, 1H), 2.22 (dq, $^3J_{H-H}$ = 13.6, 6.5 Hz, 1H), 2.11 – 1.96 (m, 4H), 1.94 – 1.81 (m, 2H), 1.68 – 1.07 (m, 21H), 1.05 (s, 3H), 0.96 (d, $^3J_{H-H}$ = 6.5 Hz, 3H), 0.92 (d, $^3J_{H-H}$ = 1.8 Hz, 3H), 0.90 (d, $^3J_{H-H}$ = 1.8 Hz, 3H), 0.72 (s, 3H).

^{13}C{^{1}H} NMR (101 MHz, $CDCl_3$) δ (ppm) = 151.0, 147.5, 146.9, 142.1, 141.1, 137.3, 136.7, 128.2, 128.0, 121.7, 120.4, 112.7, 108.0, 107.6, 101.0, 79.0, 70.0, 61.1, 56.9, 56.3, 50.3, 46.3, 42.5, 39.9, 39.7, 39.3, 37.9, 37.4, 37.0, 36.3, 35.9, 32.1, 32.0, 28.6, 28.4, 28.2, 24.4, 24.0, 23.0, 22.7, 21.2, 19.5, 18.9, 12.0.

HRMS (ESI +**)**: calculated for $C_{46}H_{65}O_4$ $[M+H]^+$: 681.4878; found: 681.4871.

IR (neat) ν (cm^{-1}) = 2934, 1610, 1503, 1488, 1435, 1236, 1096, 1040, 906, 818, 733.

m.p.: 76.1-76.7 °C

3-(naphthalen-2-yl)-4-phenylpent-4-en-1-ol (15n)

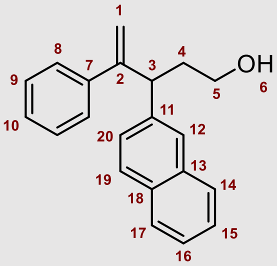

15n

Following the general procedure in **5.2** using boronate **7a** (0.3 mmol, 1.0 equiv.) and 2-bromonaphthalene **10p** (0.45 mmol, 1.5 equiv.), compound **15n** was obtained as a yellow oil (35 mg, 40% yield, $rr_{9/8}$ = 4:1) after chromatography on silica gel (pentane/EtOAc, from 10:1 to 6:1).

TLC: 0.3, pentane/EtOAc = 5:1

^{1}H NMR (400 MHz, $CDCl_3$) δ (ppm) = 7.83 – 7.74 (m, 3H, H-Ar), 7.70 (s, 1H, H-Ar), 7.49 – 7.38 (m, 3H, H-Ar), 7.35 – 7.27 (m, 2H, H-Ar), 7.28 – 7.14 (m, 3H, H-Ar), 5.47 (s, 1H, H-1), 5.28 (s, 1H, H-1), 4.22 (dd, $^3J_{H\text{-}H}$ = 9.0, 6.2 Hz, 1H, H-3), 3.76 – 3.53 (m, 2H, H-5), 2.29 (dq, $^3J_{H\text{-}H}$ = 13.5, 6.7 Hz, 1H, H-4), 2.22 – 2.07 (m, 1H, H-4), 1.30 (s, 1H, H-6).

^{13}C{^{1}H} NMR (101 MHz, $CDCl_3$) δ (ppm) = 151.5 (C-2), 142.4 (C-7), 140.3 (C-11), 133.7 (C-13 or C-18), 132.5 (C-13 or C-18), 128.3 (C-Ar), 127.8 (C-Ar), 127.7 (C-Ar), 127.4 (C-Ar), 126.9 (C-Ar), 126.8 (C-Ar), 126.7 (C-Ar), 126.0 (C-Ar), 125.6 (C-Ar), 113.8 (C-1), 61.1 (C-5), 46.6 (C-3), 37.8 (C-4).z

HRMS (ESI +**)**: calculated for $C_{21}H_{20}ONa$ $[M+Na]^+$: 311.1407; found: 311.1406.

IR (neat) ν (cm^{-1}) = 3329, 3052, 2926, 1624, 1506, 1271, 1027, 900, 855, 818, 777, 744, 699, 660.

3-(dibenzo[b,d]furan-2-yl)-4-phenylpent-4-en-1-ol (15o)

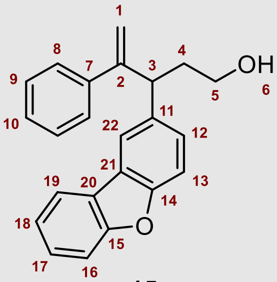

15o

Following the general procedure in **5.2** using boronate **7a** (0.3 mmol, 1.0 equiv.) and ArBr **10t** (0.45 mmol, 1.5 equiv.), compound **15o** was obtained as a yellow oil (20 mg, 20% yield, $rr_{9/8}$ = 2:1) after chromatography on silica gel (pentane/EtOAc, from 10:1 to 6:1).

TLC: 0.2, pentane/EtOAc = 5:1

^{1}H NMR (400 MHz, $CDCl_3$) δ (ppm) = 7.92 (ddd, $^3J_{H\text{-}H}$ = 7.7, 1.4, 0.7 Hz, 1H, H-Ar), 7.83 (d, $^3J_{H\text{-}H}$ = 1.8 Hz, 1H, H-Ar), 7.54 (dt, $^3J_{H\text{-}H}$ = 8.2, 0.9 Hz, 1H, H-Ar), 7.50 – 7.39 (m, 2H, H-Ar), 7.39 – 7.26 (m, 4H, H-Ar), 7.27 – 7.14 (m, 3H, H-Ar), 5.45 (s, 1H, H-1), 5.28 (s, 1H, H-1), 4.26

– 4.18 (m, 1H, H-3), 3.77 – 3.56 (m, 2H, H-5), 2.37 – 2.24 (m, 1H, H-4), 2.21 – 2.06 (m, 1H, H-4), 1.23 (s, 1H, H-6).

^{13}C{^{1}H} NMR (101 MHz, CDCl$_3$) δ (ppm) = 156.7 (C-14 or C-15), 155.2 (C-14 or C-15), 152.0 (C-2), 142.4 (C-7), 137.4 (C-11), 128.3 (C-Ar), 127.5 (C-Ar), 127.4 (C-Ar), 127.2 (C-Ar), 127.0 (C-Ar), 124.5 (C-Ar), 124.4 (C-Ar), 122.7 (C-Ar), 120.8 (C-Ar), 120.1 (C-12 or C-22), 113.5 (C-1), 111.8 (C-Ar), 111.6 (C-Ar), 61.2 (C-5), 46.5 (C-3), 38.3 (C-4).

HRMS (ESI +**)**: calculated for $C_{23}H_{21}O_2$ [M+H]$^+$: 329.1537; found: 329.1544.

IR (neat) ν (cm^{-1}) = 3321, 2924, 1732, 1628, 1476, 1447, 1195, 1022, 901, 841, 777, 747, 702, 637.

3-(2-methoxypyridin-4-yl)-4-phenylpent-4-en-1-ol (15p)

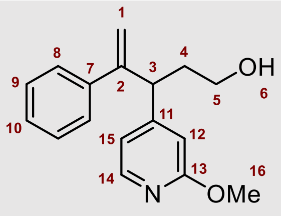

15p

Following the general procedure in **5.2** using boronate **7a** (0.3 mmol, 1.0 equiv.) and 4-bromo-2-methoxypyridine **10u** (0.45 mmol, 1.5 equiv.), compound **15p** was obtained as a yellow oil (36 mg, 45% yield, $rr_{9/8}$ = 2:1) after chromatography on silica gel (pentane/EtOAc, from 10:1 to 6:1).

TLC: 0.2, pentane/EtOAc = 5:1

^{1}H NMR (400 MHz, CDCl$_3$) δ (ppm) = 8.04 (d, J = 5.4 Hz, 1H, H-14), 7.29 – 7.18 (m, 5H, H-8, H-9 and H-10), 6.79 (dd, $^3J_{H-H}$ = 5.4, 1.5 Hz, 1H, H-15), 6.67 – 6.62 (m, 1H, H-12), 5.46 (s, 1H, H-1), 5.23 (s, 1H, H-1), 4.02 (t, $^3J_{H-H}$ = 7.6 Hz, 1H, H-3), 3.90 (s, 3H, H-16), 3.73 – 3.54 (m, 2H, H-5), 2.19 (ddt, $^3J_{H-H}$ = 13.8, 7.3, 6.5 Hz, 1H, H-4), 2.01 (ddt, $^3J_{H-H}$ = 13.8, 8.6, 5.9 Hz, 1H, H-4), 1.29 (s, 1H, H-6).

^{13}C{^{1}H} NMR (101 MHz, CDCl$_3$) δ (ppm) = 164.7 (C-13), 155.1 (C-11), 150.0 (C-2), 146.9 (C-14), 141.8 (C-7), 128.4 (C-9 or C-10), 127.7 (C-9 or C-10), 126.8 (C-8), 117.1 (C-15), 114.3 (C-1), 110.3 (C-12), 60.7 (C-5), 53.5 (C-16), 45.7 (C-3), 37.0 (C-4).

HRMS (ESI +**)**: calculated for $C_{17}H_{19}NO_2Na$ [M+Na]$^+$: 292.1308; found: 292.1315.

IR (neat) ν (cm^{-1}) = 3347, 2944, 1605, 1557, 1480, 1447, 1396, 1316, 1153, 1036, 904, 827, 777, 700.

3-phenyl-4-(4-(trifluoromethyl)phenyl)pent-4-en-1-ol (15q)

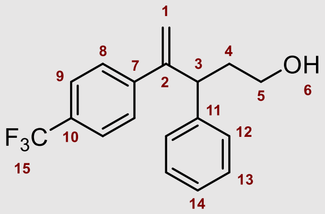

15q

Following the general procedure in **5.2** using boronate **7b** (0.3 mmol, 1.0 equiv.) and PhBr **10a** (0.45 mmol, 1.5 equiv.), compound **15q** was obtained as a yellow oil (41 mg, 45% yield, $rr_{9/8}$ = 3:1) after chromatography on silica gel (pentane/EtOAc, from 10:1 to 6:1).

TLC: 0.2, pentane/EtOAc = 5:1

^{1}H NMR (400 MHz, $CDCl_3$) δ (ppm) = 7.48 (d, $^3J_{H\text{-}H}$ = 8.1 Hz, 2H, H-9), 7.36 (d, $^3J_{H\text{-}H}$ = 7.8 Hz, 2H, H-8), 7.32 – 7.15 (m, 5H, H-12, H-13 and H-14), 5.45 (s, 1H, H-1), 5.32 (s, 1H, H-1), 4.04 (ddd, $^3J_{H\text{-}H}$ = 9.2, 6.1, 1.2 Hz, 1H, H-3), 3.68 (dt, $^3J_{H\text{-}H}$ = 10.6, 6.0 Hz, 1H, H-5), 3.58 (ddd, $^3J_{H\text{-}H}$ = 10.6, 7.4, 5.9 Hz, 1H, H-5), 2.21 (ddt, $^3J_{H\text{-}H}$ = 13.7, 7.5, 6.2 Hz, 1H, H-4), 2.05 (ddt, $^3J_{H\text{-}H}$ = 13.7, 9.2, 5.8 Hz, 1H, H-4), 1.57 (s, 1H, H-6).

^{13}C{^{1}H} NMR (101 MHz, $CDCl_3$) δ (ppm) = 150.7 (C-2), 146.0 (C-7), 142.2 (C-11), 129.4 (q, $^2J_{C\text{-}F}$ = 32.6 Hz, C-10), 128.7 (C-13 or C-14), 128.2 (C-12), 127.3 (C-8), 126.8 (C-13 or C-14), 125.2 (q, $^3J_{C\text{-}F}$ = 3.9 Hz, C-9), 124.3 (q, $^1J_{C\text{-}F}$ = 272.0 Hz, C-15), 115.0 (C-1), 60.9 (C-5), 46.4 (C-3), 37.6 (C-4).

^{19}F{^{1}H} NMR (282 MHz, $CDCl_3$) δ (ppm) = –62.5

HRMS (ESI +**)**: calculated for $C_{18}H_{17}F_3ONa$ $[M+Na]^+$: 329.1124; found: 329.1099.

IR (neat) ν (cm^{-1}) = 3323, 2926, 1616, 1493, 1404, 1322, 1163, 1116, 1066, 1014, 910, 846, 699, 599.

4-(3,5-dimethylphenyl)-3-(2-methoxyphenyl)pent-4-en-1-ol (15s)

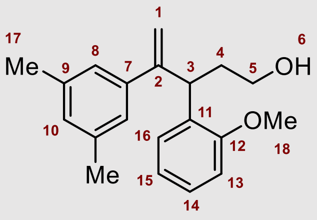

15s

Following the general procedure in **5.2** using boronate **7f** (0.3 mmol, 1.0 equiv.) and 1-bromo-2-methoxybenzene **10c** (0.45 mmol, 1.5 equiv.), compound **15s** was obtained as a yellow oil (45 mg, 51% yield, $rr_{9/8}$ = 7.5:1) after chromatography on silica gel (pentane/EtOAc, from 10:1 to 7.5:1).

TLC: 0.3, pentane/EtOAc = 5:1

^{1}H NMR (400 MHz, $CDCl_3$) δ (ppm) = 7.20 (dd, J = 7.6, 1.7 Hz, 1H, H-13), 7.14 (ddd, J = 8.1, 7.4, 1.7 Hz, 1H, H-14), 6.98 (s, 2H, H-8), 6.89 (td, J = 7.5, 1.2 Hz, 1H, H-15), 6.85 (dd, J = 8.2, 1.2 Hz, 1H, H-16), 6.81 (s, 1H, H-10), 5.45 (s, 1H, H-1), 5.17 (s, 1H, H-1), 4.59 (dd, J = 10.4,

4.9 Hz, 1H, H-3), 3.88 (s, 3H, H-18), 3.68 – 3.55 (m, 1H, H-5), 3.48 – 3.33 (m, 1H, H-5), 2.28 – 2.15 (m, 7H, H-17 and H-4), 2.05 (brs, 1H, H-6), 1.93 – 1.79 (m, 1H, H-4).

^{13}C{^{1}H} NMR (101 MHz, CDCl$_3$) δ (ppm) = 157.1 (C-12), 151.6 (C-2), 142.3 (C-7), 137.4 (C-9), 131.2 (C-11), 129.0 (C-10), 128.2 (C-13), 127.5 (C-14), 124.5 (C-8), 121.4 (C-15), 112.4 (C-1), 110.8 (C-16), 61.0 (C-5), 56.0 (C-18), 37.9 (C-4), 36.8 (C-3), 21.5 (C-17).

HRMS (ESI +**)**: calculated for $C_{20}H_{25}O_2$ [M+H]$^+$: 297.1850; found: 297.1843.

IR (neat) ν (cm^{-1}) = 3367, 2938, 1597, 1489, 1239, 1104, 1028, 894, 850, 751.

3-(1-benzyl-1H-pyrrolo[2,3-b]pyridin-4-yl)-4-(dibenzo[b,d]furan-2-yl)pent-4-en-1-ol (15t)

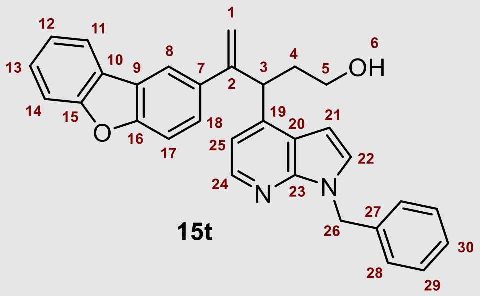

Following the general procedure in **5.2** using boronate **7e** (0.3 mmol, 1.0 equiv.) and 1-benzyl-4-bromo-1H-pyrrolo[2,3-b]pyridine **10i** (0.45 mmol, 1.5 equiv.), compound **15t** was obtained as a yellow oil (100 mg, 73% yield, $rr_{9/8}$ = 9:1) after chromatography on silica gel (pentane/EtOAc, from 5:1 to 2:1 to 1:1).

TLC: 0.1, pentane/EtOAc = 1:1

^{1}H NMR (400 MHz, CDCl$_3$) δ (ppm) = 8.25 (d, J = 5.0 Hz, 1H, H-24), 7.87 (dd, J = 1.8, 0.7 Hz, 1H, H-8 or H-18), 7.82 (ddd, J = 7.7, 1.4, 0.7 Hz, 1H, H-Ar), 7.55 – 7.48 (m, 1H, H-Ar), 7.46 – 7.35 (m, 3H, H-Ar), 7.30 (dd, J = 7.6, 1.0 Hz, 1H, H-Ar), 7.30 – 7.21 (m, 3H, H-Ar), 7.21 – 7.14 (m, 2H, H-Ar), 7.14 (d, J = 3.6 Hz, 1H, H-22), 7.03 (d, J = 5.0 Hz, 1H, H-25), 6.65 (d, J = 3.6 Hz, 1H, H-21), 5.57 (s, 1H, H-1), 5.46 (d, J = 3.8 Hz, 2H, H-26), 5.39 (s, 1H, H-1), 4.66 – 4.57 (m, 1H, H-3), 3.77 – 3.67 (m, 1H, H-5), 3.67 – 3.54 (m, 1H, H-5), 2.46 – 2.33 (m, 1H, H-4), 2.33 – 2.17 (m, 1H, H-4), 1.41 (s, 1H, H-6).

^{13}C{^{1}H} NMR (101 MHz, CDCl$_3$) δ (ppm) = 156.7 (C-15 or C-16), 155.7 (C-15 or C-16), 150.3 (C-2), 148.0 (C-23), 144.8 (C-19), 143.5 (C-24), 137.8 (C-27), 137.2 (C-7), 128.8 (C-Ar), 127.71 (C-Ar), 127.66 (C-Ar), 127.3 (C-Ar), 126.2 (C-Ar), 124.3 (C-9 or C-10), 124.1 (C-9 or C-10), 122.8 (C-Ar), 120.7 (C-Ar), 120.5 (C-20), 118.9 (C-Ar), 114.6 (C-25), 114.0 (C-1), 111.8 (C-Ar), 111.3 (C-Ar), 98.8 (C-21), 61.0 (C-5), 48.1 (C-26), 43.8 (C-3), 37.3 (C-4).

HRMS (ESI +**)**: calculated for $C_{31}H_{27}N_2O_2$ [M+H]$^+$: 459.2068; found: 459.2080.

IR (neat) ν (cm^{-1}) = 3420, 2975, 1735, 1582, 1449, 1240, 1196, 1113, 1044, 820, 749, 720.

3-(1-methyl-1H-indol-5-yl)-4-(3,4,5-trimethoxyphenyl)pent-4-en-1-ol (15u)

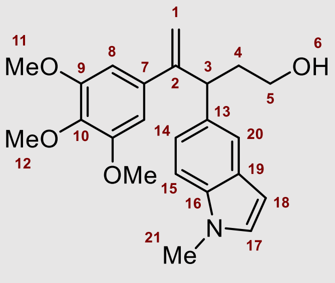

15u

Following the general procedure in **5.2** using boronate **7c** (0.3 mmol, 1.0 equiv.) and 5-bromo-1-methyl-1H-indole **10h** (0.45 mmol, 1.5 equiv.), compound **15u** was obtained as a yellow oil (27.5 mg, 24% yield, $rr_{9/8}$ = 9:1) after chromatography on silica gel (pentane/EtOAc, from 5:1 to 2:1 to 1:1).

TLC: 0.2, pentane/EtOAc = 1:1

^{1}H NMR (400 MHz, $CDCl_3$) δ (ppm) = 7.52 (d, J = 1.6 Hz, 1H, H-20), 7.24 (s, 1H, H-15), 7.16 (dd, J = 8.5, 1.7 Hz, 1H, H-14), 7.02 (d, J = 3.1 Hz, 1H, H-17), 6.52 (s, 2H, H-8), 6.41 (dd, J = 3.2, 0.8 Hz, 1H, H-18), 5.40 (s, 1H, H-1), 5.23 (s, 1H, H-1), 4.05 (dd, J = 9.3, 5.9 Hz, 1H, H-3), 3.78 (s, 3H, H-12), 3.77 (s, 3H, H-21), 3.73 (s, 6H, H-11), 3.72 – 3.62 (m, 1H, H-5), 3.65 – 3.55 (m, 1H, H-5), 2.30 – 2.17 (m, 1H, H-4), 2.18 – 2.03 (m, 1H, H-4), 1.21 (s, 1H, H-6).

^{13}C{^{1}H} NMR (101 MHz, $CDCl_3$) δ (ppm) = 152.8 (C-9), 152.3 (C-2), 138.4 (C-7), 137.4 (C-10), 135.9 (C-16), 133.6 (C-13), 129.2 (C-17), 128.8 (C-19), 122.2 (C-14), 120.2 (C-20), 112.7 (C-1), 109.4 (C-15), 104.4 (C-8), 100.8 (C-18), 61.5 (C-5), 60.9 (C-12), 56.1 (C-11), 46.9 (C-3), 38.3 (C-4), 33.0 (C-21).

HRMS (ESI +**)**: calculated for $C_{23}H_{28}NO_4$ $[M+H]^+$: 382.2013; found: 382.2030.

IR (neat) ν (cm^{-1}) = 3401, 2933, 1716, 1579, 1506, 1408, 1335, 1240, 1122, 1005, 723.

5.3 Limitation of the methods

5.3.1 Limitation of C1 arylation

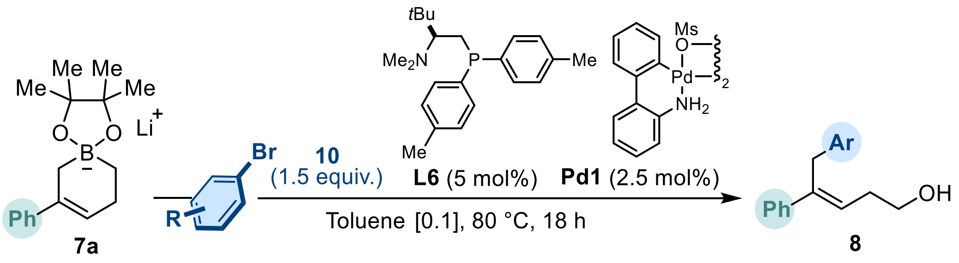

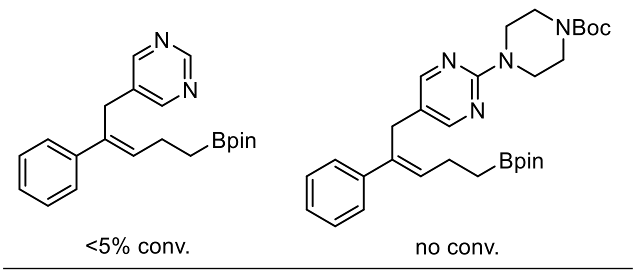

5.3.2 Limitation of C3 arylation

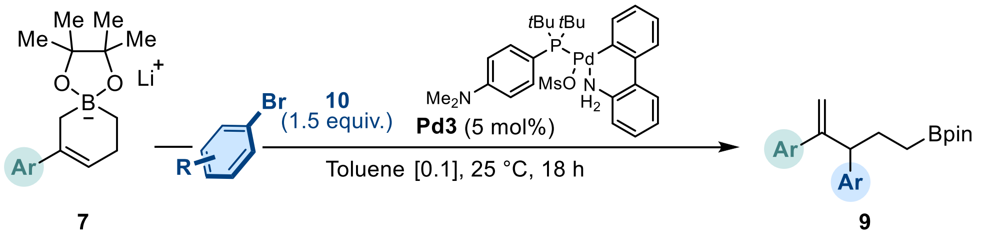

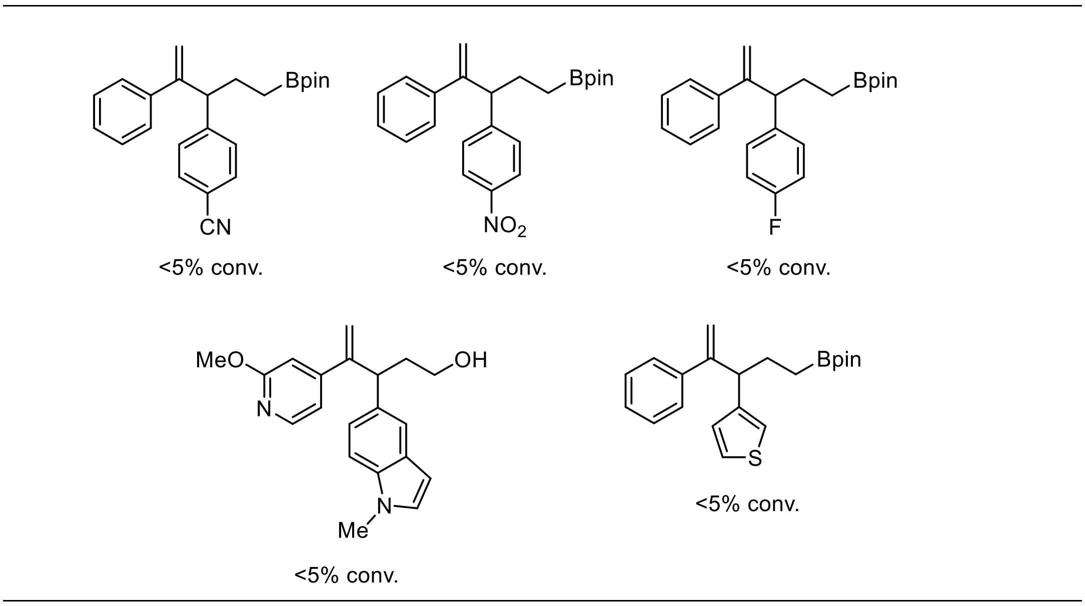

6. Gram scale experiment

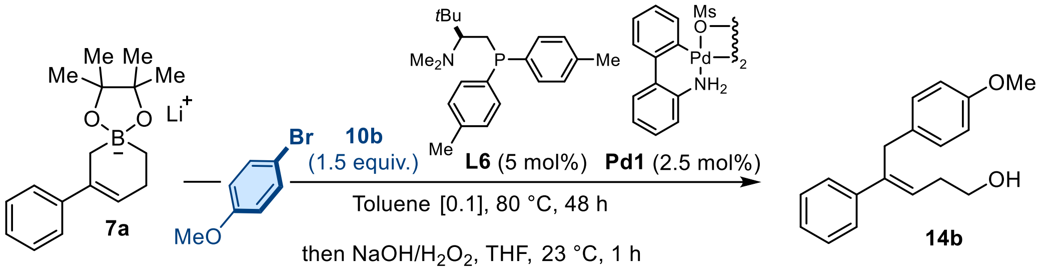

Figure S6. Gram scale experiment

In a nitrogen filled glovebox, to an oven dried screw capped Schlenk equipped with a magnetic stir bar, $(PdG_3)_2$ (67 mg, 0.09 mmol, 2.5 mol%) and ligand **L6** (62 mg, 0.18 mmol, 5 mol%) were dissolved in toluene (6 mL), and stirred at 25 °C. After 30 min., cyclic allylboronate **7a** (1.16 g, 3.6 mmol, 1.0 equiv.) was added, followed by 30 mL of toluene. Subsequently, *p*-bromoanisole **10b** (675 µL, 5.4 mmol, 1.5 equiv.) was added by microsyringe. The system was closed, and the mixture was stirred at 80 °C. After 48 h, the solution was cooled to room temperature and the mixture filtered on Celite, washed with Et_2O (3 × 10 mL), and the solution was concentrated under vacuum. The alkali oxidation was conducted next. The crude reaction mixture was dissolved in THF (20 mL), and an aqueous NaOH solution (10 mL, 4 M) and 30% aq. H_2O_2 solution (10 mL) were added at room temperature. After 1 h at 23 °C, the reaction mixture was extracted with CH_2Cl_2 (3 × 10 mL). The combined organic phases were dried over Na_2SO_4 and concentrated under reduced pressure after filtration. The residue was purified by flash column chromatography on silica gel using pentane/EtOAc as eluent from 10:1 to 6:1, to afford homoallylic alcohol **14b** as an oil in 66% yield (632 mg).

7. Mechanistic investigations

7.1 Synthesis of Pd complex 16

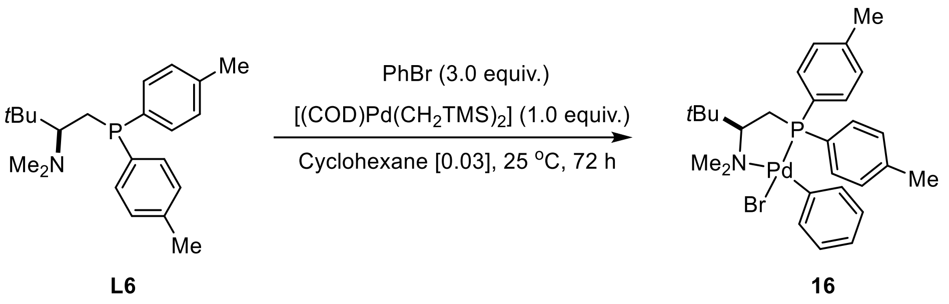

Figure S7. Synthesis of complex **16**

In a glovebox, to an oven dried screw capped Schlenk equipped with a magnetic stir bar, the ligand **L6** (27.3 mg, 0.08 mmol, 1.0 equiv.) and [(COD)Pd(CH_2TMS)$_2$] (31.1 mg, 0.08 mmol, 1.0 equiv.) were added. Subsequently, cyclohexane (3.0 mL) and bromobenzene (25 μL, 0.24 mmol, 3.0 equiv.) were added by syringe. The system was closed and the mixture was stirred at 25 °C. After 72 h, the mixture was filtered over celite, washing with cyclohexane (1 mL × 3). The filtrate was concentrated on vacuum, and the resulting solid was dissolved into Et_2O (1 mL) in a 10 mL vial, followed by slowly adding 3 mL pentane. The system was kept at –30 °C. After 24 h, the solvent was removed by pipette, and the remaining solid was further purified by layering in Et_2O/pentane (1 mL/3 mL) at –30 °C. After 24 h, the solvent was removed by pipette, and the remaining yellow solid was further washed with pentane (2 mL × 3), and dried on vacuum, affording complex **16** in 42% yield (20 mg).

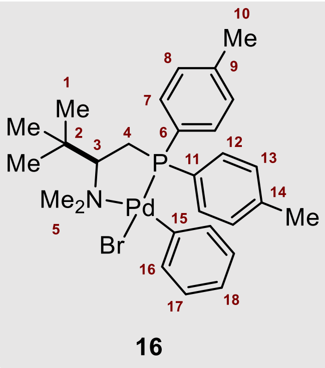

^{1}H NMR (400 MHz, C_6D_6) δ (ppm) = 7.73 (dd, *J* = 11.4, 8.1 Hz, 2H, H-Ar), 7.52 (ddd, *J* = 8.1, 3.3, 1.3 Hz, 2H, H-Ar), 7.02 (dd, *J* = 11.1, 8.2 Hz, 2H, H-Ar), 6.98 – 6.84 (m, 6H, H-Ar), 6.85 – 6.77 (m, 1H, H-Ar), 3.38 (s, 3H, H-5), 2.96 (s, 3H, H-5), 2.67 (td, *J* = 13.8, 3.3 Hz, 1H, H-4), 2.19 (ddd, *J* = 13.6, 12.0, 1.8 Hz, 1H, H-3), 2.09 (ddd, *J* = 15.6, 14.1, 1.8 Hz, 1H, H-4), 1.99 (s, 3H, H-10 or H-15), 1.97 (s, 3H, H-10 or H-15), 0.58 (s, 9H, H-1).

^{13}C {^{1}H} NMR (101 MHz, C_6D_6) δ (ppm) = 142.2 (d, *J* = 2.7 Hz, C-Ar), 141.9 (C-Ar), 140.7 (d, *J* = 2.9 Hz, C-Ar), 138.0 (d, *J* = 5.1 Hz, C-Ar), 135.0 (d, *J* = 12.9 Hz, C-Ar), 132.4 (d, *J* = 9.9 Hz, C-Ar), 130.1 (d, *J* = 11.2 Hz, C-Ar), 129.4 (d, *J* = 10.9 Hz, C-Ar), 127.2 (d, *J* = 2.3 Hz, C-Ar), 122.8 (C-Ar), 71.8 (d, $^2J_{C\text{-}P}$ = 5.0 Hz, C-3), 51.8 (C-5), 41.4 (C-5), 37.4 (d, $^3J_{C\text{-}P}$ = 13.0 Hz, C-2), 33.8 (d, $^1J_{C\text{-}P}$ = 30.0 Hz), 30.1 (C-1), 21.3 (C-10).

^{31}P {^{1}H} NMR (162 MHz, C_6D_6) δ (ppm) = 24.2

HRMS (ESI +**)**: calculated for C28H37NPPd [M – Br]+: 524.1693; found: 524.1710.

IR (neat) ν (cm^{-1}) = 2968, 1564, 1469, 1398, 1102, 1060, 1019, 967, 904, 802, 727, 692.

m.p.: 170-172 °C

7.2 Synthesis of Pd complex 17

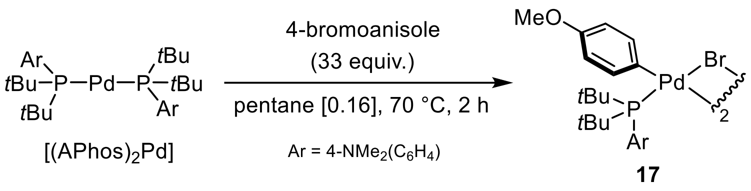

Figure S8. Synthesis of complex **17**

In a glovebox, to an oven dried screw capped Schlenk equipped with a magnetic stir bar, [(APhos)$_2$Pd] (50 mg, 0.079 mmol, 1.0 equiv.) was added, followed by pentane (5.0 mL). Subsequently, 4-Bromoanisole (325 μL, 2.59 mmol, 33 equiv.) was added by microsyringe. The system was closed and the mixture was stirred at 70 °C for 2 h, during which time a yellow solid precipitated. After cooling to room temperature, the solid was collected by filtration and washed with pentane (10 mL × 3). The yellow solid was dried under vacuum for 24 h, affording complex **17** in 62% yield (27 mg).

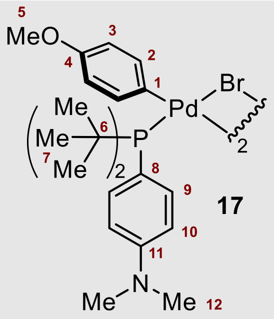

^{1}H NMR (400 MHz, THF-d$_8$) δ (ppm) = 7.85 (t, $^3J_{H\text{-}H}$ = 8.9 Hz, 2H, H-9 or H-10), 7.20 (dd, J = 8.8, 2.2 Hz, 2H, H-2), 6.67 – 6.57 (m, 2H, H-9 or H-10), 6.44 (d, J = 8.7 Hz, 2H, H-3), 3.58 (s, 3H, H-5), 2.96 (s, 6H, H-12), 1.31 (d, J = 13.6 Hz, 18H, H-7).

^{13}C {^{1}H} NMR (101 MHz, THF-d$_8$) δ (ppm) = 157.5 (C-4), 152.1 (C-11), 139.2 (d, $^2J_{C\text{-}P}$ = 11.2 Hz, C-9), 138.8 (d, $^3J_{C\text{-}P}$ = 3.0 Hz, C-2), 133.7 (C-1), 116.6 (d, $^1J_{C\text{-}P}$ = 40.8 Hz, C-8), 113.2 (C-3), 110.7 (d, $^3J_{C\text{-}P}$ = 10.5 Hz, C-10), 55.0 (C-5), 40.1 (C-12), 38.5 (d, $^1J_{C\text{-}P}$ = 16.7 Hz, C-6), 31.4 (d, $^2J_{C\text{-}P}$ = 4.1 Hz, C-7).

^{31}P {^{1}H} NMR (162 MHz, THF-d$_8$) δ (ppm) = 60.1

HRMS (ESI +**)**: calculated for $C_{23}H_{35}NOPPd$ [M – Br]$^+$: 478.1486; found: 478.1475.

IR (neat) ν (cm^{-1}) = 2922, 1597, 1513, 1478, 1359, 1228, 1174, 1096, 1001, 810.

m.p.: 145-146 °C.

7.3 Mechanistic experiments

7.3.1 Catalytic experiment with Pd complex 16

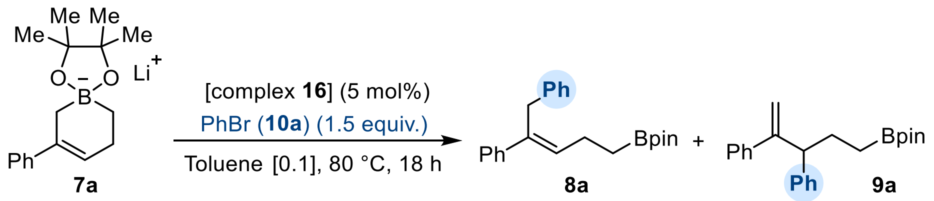

Figure S9. Catalytic experiment with Pd complex **16**.

In a glovebox, to an oven dried screw capped Schlenk equipped with a magnetic stir bar, Pd complex **16** (3.0 mg, 0.005 mmol, 5 mol%) and cyclic boronate **7a** (33 mg, 0.1 mmol, 1.0 equiv.) were dissolved in toluene (1.0 mL). Subsequently, PhBr **10a** (16 μL, 1.5 equiv.) was added by microsyringe. The system was closed, and the mixture was stirred at 80 °C. After 18 h, the solution was cooled to room temperature and the mixture filtered on Celite, washed with Et_2O (3 × 5 mL), and the solution was concentrated under vacuum. The conversion and regioselectivity were measured by ^1H NMR against an internal standard, indicating 85% conversion with 27:1 $rr_{\mathbf{8a/9a}}$.

7.3.2 Catalytic experiment with Pd complex 17

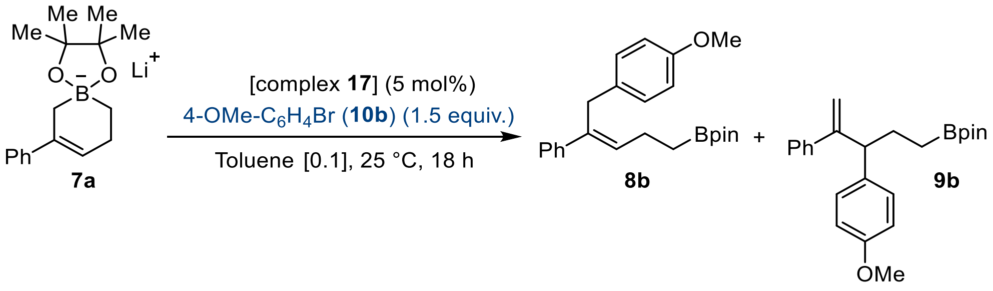

Figure S10. Catalytic experiment with Pd complex **10**.

In a glovebox, to an oven dried screw capped Schlenk equipped with a magnetic stir bar, Pd complex **17** (2.8 mg, 2.5 mol%) and cyclic boronate **7a** (33 mg, 0.1 mmol, 1.0 equiv.) were dissolved in toluene (1.0 mL). Subsequently, *p*-bromoanisole **10b** (19 μL, 1.5 equiv.) was added by microsyringe. The system was closed, and the mixture was stirred at 25 °C. After 18 h, the mixture filtered on Celite, washed with Et_2O (3 × 5 mL), and the solution was concentrated under vacuum. The conversion and regioselectivity were measured by ^1H NMR against an internal standard, indicating 94% conversion with 20:1 $rr_{\mathbf{9b/8b}}$.

8. Synthetic application

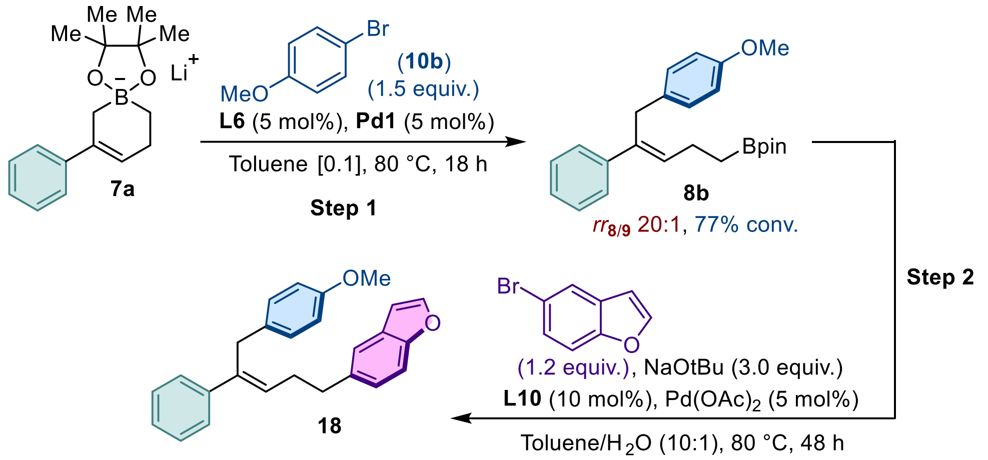

Step 1:

In glovebox, to an oven dried screw capped Schlenk equipped with a magnetic stir bar, $(PdG_3)_2$ (5.6 mg, 2.5 mol%) and the ligand **L6** (5.2 mg, 5 mol%) were dissolved in toluene (0.5 mL), and stirred at 25 °C. After 30 min., the cyclic allylboronate **7a** (0.3 mmol, 1.0 equiv.) was added, followed by another 2.5 mL of toluene. Subsequently, the ArBr **10b** (0.45 mmol, 1.5 equiv.) was added. The system was closed, and the mixture was stirred at 80 °C. After 18 h, the solution was cooled to room temperature and the mixture filtered on Celite, washed with Et_2O (3 × 5 mL), and the solution was concentrated under vacuum. The crude was further purified by flash column chromatography on silica gel using pentane/EtOAc (from 30:1 to 15:1) as eluent to afford the mixture of homoallylic boronic esters **8b** and **9b** (77% conv., $rr_{\mathbf{8b/9b}}$ = 20:1). The mixture was used directly in next step without further purification.

Step 2:

In glovebox, to an oven dried screw capped Schlenk equipped with a magnetic stir bar, $Pd(OAc)_2$ (3.4 mg, 0.015 mmol, 5 mol%), RuPhos (14 mg, 0.03 mmol, 10 mol%) and NaOtBu (87 mg, 0.9 mmol, 3.0 equiv.) were added. Subsequently, the mixture of **8b** and **9b** from step 1 was added with toluene (2.6 mL), and the tube was closed and taken out of glovebox. Under the N_2, 5-Bromobenzofuran (45 μL, 0.36 mmol, 1.2 equiv.) and H_2O (0.26 mL) were added by syringe. After addition, the tube was closed and the mixture was stirred at 80 °C. After 48 h, the mixture was filtered on Celite®, washed with Et_2O (3 × 5 mL), and the solution was concentrated under vacuum. The crude was purified by flash column chromatography on silica gel using pentane/EtOAc (100:1) as eluent to afford the product **18** in 38% yield over two steps (42 mg) as a yellow oil.

(*E*)-5-(5-(4-methoxyphenyl)-4-phenylpent-3-en-1-yl)benzofuran (18)

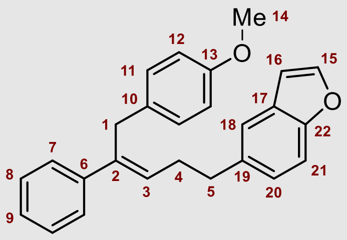

18

Following the general procedure in **8**, compound **18** was obtained as a yellow oil (42 mg, 38% yield over two steps) after chromatography on silica gel (pentane/EtOAc, 100:1).

TLC: 0.8, pentane/EtOAc = 20:1

^{1}H NMR (400 MHz, $CDCl_3$) δ (ppm) = 7.46 (d, $^3J_{H-H}$ = 2.2 Hz, 1H, H-15), 7.28 (s, 1H, H-18), 7.19 – 7.12 (m, 2H, H-7), 7.14 – 7.08 (m, 2H, H-8), 7.07 (s, 1H, H-9), 7.05 – 6.96 (m, 2H, H-16 and H-20), 6.83 (d, $^3J_{H-H}$ = 8.9 Hz, 2H, H-11), 6.60 – 6.52 (m, 3H, H-12 and H-21), 5.86 (t, $^3J_{H-H}$ = 7.2 Hz, 1H, H-3), 3.61 (s, 2H, H-1), 3.60 (s, 3H, H-14), 2.73 (t, $^3J_{H-H}$ = 7.5 Hz, 2H, H-5), 2.47 (q, $^3J_{H-H}$ = 7.6 Hz, 2H, H-4).

^{13}C{^{1}H} NMR (101 MHz, $CDCl_3$) δ (ppm) = 157.9 (C-13), 153.8 (C-22), 145.2 (C-15), 143.1 (C-6), 138.6 (C-2), 136.4 (C-19), 131.8 (C-10), 129.8 (C-3), 129.2 (C-11), 128.3 (C-Ar), 127.7 (C-17), 126.8 (C-Ar), 126.4 (C-7 or C-8 or C-9), 125.2 (C-16 or C-20 or C-21), 120.7 (C-18), 113.9 (C-12), 111.2 (C-7 or C-8 or C-9), 106.6 (C-16 or C-20 or C-21), 55.3 (C-14), 36.0 (C-5), 35.0 (C-1), 31.7 (C-4).

HRMS (ESI +**)**: calculated for $C_{26}H_{25}O_2$ $[M+H]^+$: 369.1850; found: 369.1832.

IR (neat) ν (cm^{-1}) = 2926, 1609, 1508, 1466, 1442, 1242, 1175, 1125, 1031, 881, 809, 734.

9. X-ray analyses

Suitable crystals were selected and X-ray intensity data were collected on a. Rigaku XtaLAB Synergy, Dualflex, HyPix-Arc 150° diffractometer using Cu Kα radiation (λ = 1.54184 Å). Using Olex210, the structures were solved with the SHELXT11 structure solution program using dual space methods and refined with the SHELXL12 refinement package using Least Squares minimization. Summaries of crystal data and structure refinement are given in the following tables.

CCDC Number	2475759	
Empirical formula	C28 H37 Br N P Pd	
Formula weight	604.86	
Temperature	120 K	
Wavelength	1.54184 Å	
Crystal system	Orthorhombic	
Space group	$P2_12_12_1$	
Unit cell dimensions	a = 10.12340 (10) Å	α= 90°
	b = 10.90100 (10) Å	β= 90°
	c = 51.1310 (3) Å	γ= 90°
Volume	5642.57 (8) $Å^3$	
Z	8	
Density (calculated)	1.424 Mg/m^3	
Absorption coefficient	7.599 mm^{-1}	
F(000)	2464	
Crystal size	0.327 x 0.227 x 0.196 mm^3	
Theta range for data collection	3.458 to 74.910°	
Index ranges	-10<=h<=12, -13<=k<=13, -63<=l<=63	
Reflections collected	160153	
Independent reflections	11544 [R(int) = 0.0720]	
Completeness to theta = 67.684°	100.0 %	
Absorption correction	Gaussian	
Max. and min. transmission	0.664 and 0.062	
Refinement method	Full-matrix least-squares on F^2	
Data / restraints / parameters	11544 / 0 / 592	
Goodness-of-fit on F^2	1.102	
Final R indices [I>2sigma(I)]	R1 = 0.0415, wR2 = 0.1099	
R indices (all data)	R1 = 0.0416, wR2 = 0.1100	
Absolute structure parameter	0.021(10)	
Extinction coefficient	n/a	
Largest diff. peak and hole	1.422 and -1.404 e.$Å^{-3}$	

Table S2. Crystal data and structure refinement for complex **16**.

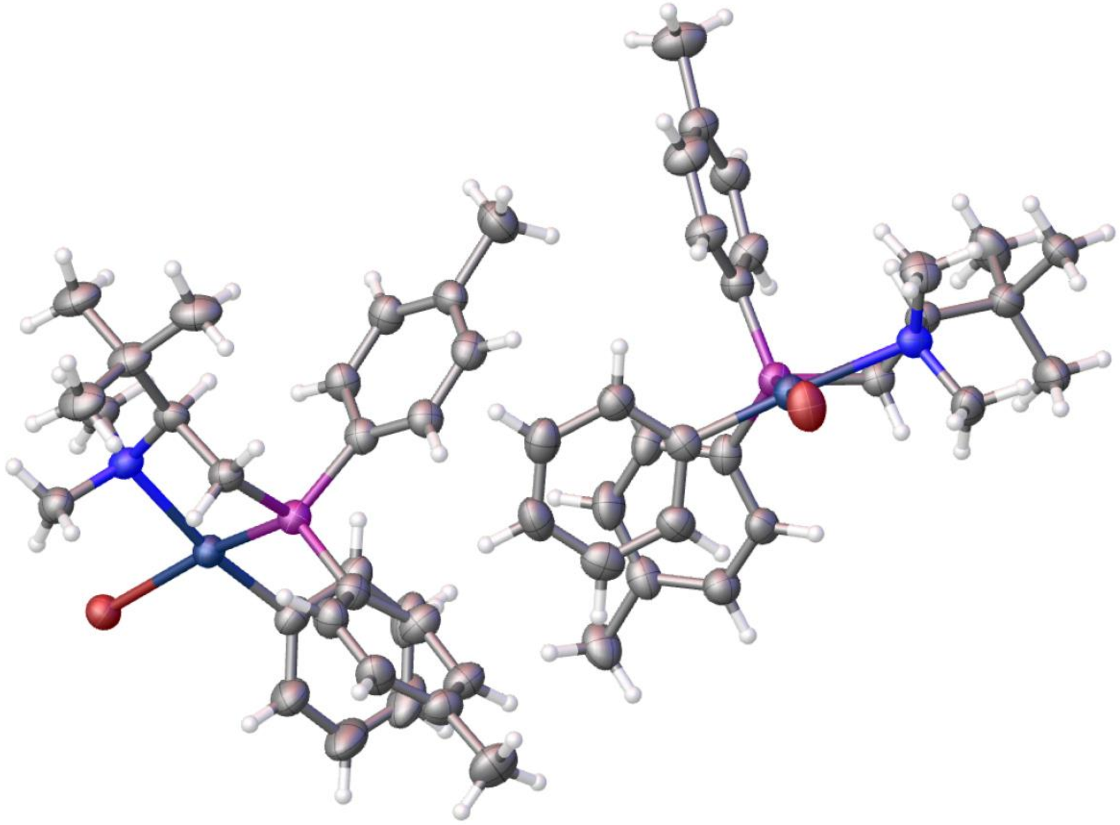

Figure S11. View of the asymmetric unit of complex **16**, with displacement ellipsoids at 50 percent probability level.

CCDC Number	2475760	
Empirical formula	C50 H80 Br2 N2 O3 P2 Pd2	
Formula weight	1191.72	
Temperature	120 K	
Wavelength	1.54184 Å	
Crystal system	Monoclinic	
Space group	$P2_1/c$	
Unit cell dimensions	a = 19.26540 (10) Å	α= 90°
	b = 15.40890 (10) Å	β= 99°
	c = 18.56470 (10) Å	γ= 90°
Volume	5443.19 (6) $Å^3$	
Z	4	
Density (calculated)	1.454 Mg/m^3	
Absorption coefficient	7.905 mm^{-1}	
F(000)	2440	
Crystal size	0.448 x 0.096 x 0.031 mm^3	
Theta range for data collection	2.322 to 74.782°	
Index ranges	-24<=h<=24, -19<=k<=18, -23<=l<=18	
Reflections collected	65425	
Independent reflections	11035 [R(int) = 0.0676]	
Completeness to theta = 67.684°	99.8 %	
Absorption correction	Gaussian	
Max. and min. transmission	1.000 and 0.221	
Refinement method	Full-matrix least-squares on F^2	
Data / restraints / parameters	11035 / 0 / 570	
Goodness-of-fit on F^2	1.079	
Final R indices [I>2sigma(I)]	R1 = 0.0353, wR2 = 0.1022	
R indices (all data)	R1 = 0.0363, wR2 = 0.1031	
Extinction coefficient	n/a	
Largest diff. peak and hole	0.942 and -1.423 e.$Å^{-3}$	

Table S3. Crystal data and structure refinement for complex **17**.

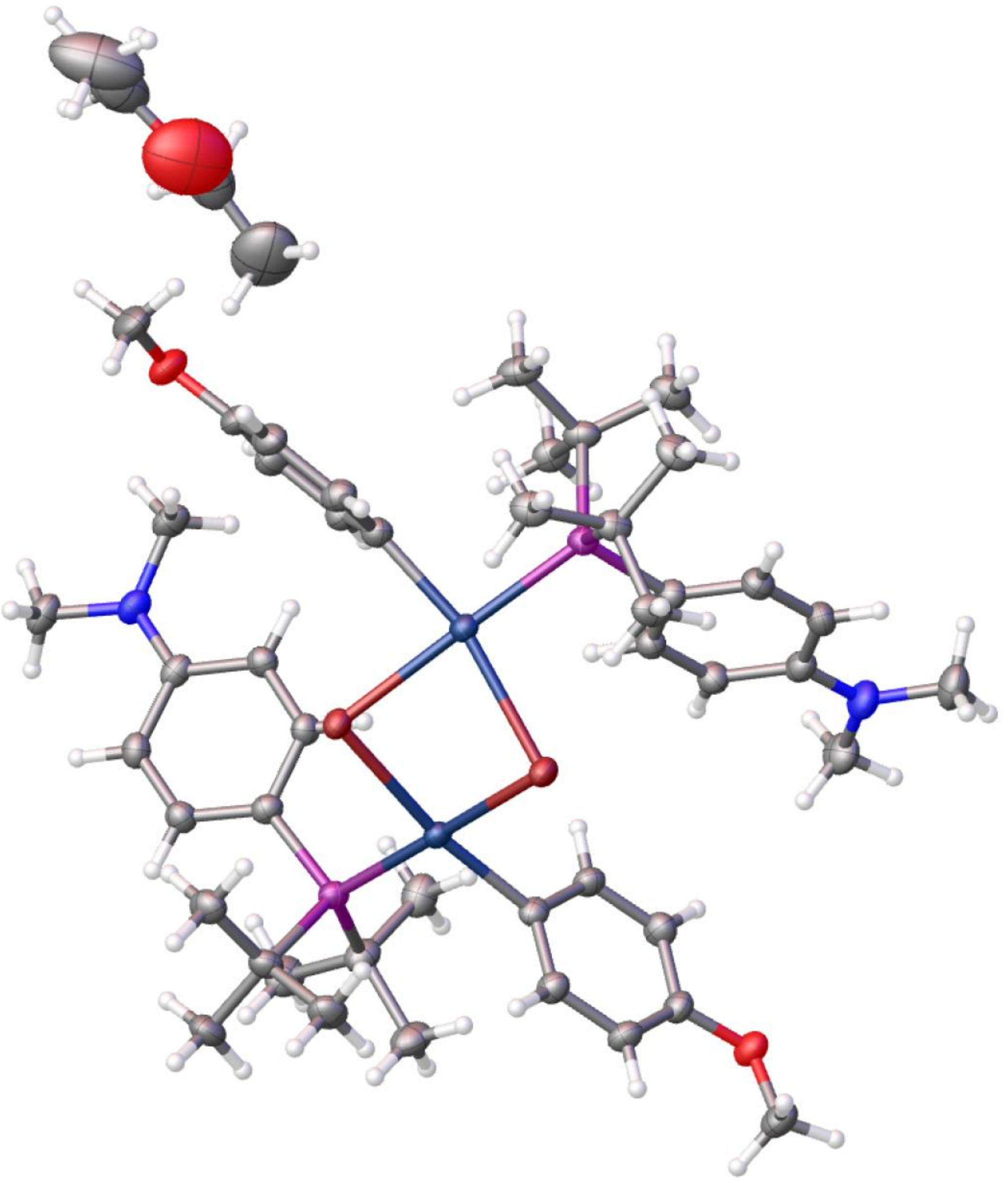

Figure S12. Asymmetric unit of complex **17**, with displacement ellipsoids at 50 percent probability level.

10. Computational studies

Level of theory. All calculations were carried out using the ORCA 6.0.0 package.[9–11] Geometries were optimized using the UMA-s-1 model,[12] with ALPB solvation contributions in toluene from xTB version 6.7.1,[13,14] through the "ExtOpt" functionality of ORCA and making use of custom wrapper scripts. These scripts are freely available along with the necessary documentation on Github (github.com/leforesb/ORCA_wrapper_UMA). All stationary points were verified to be minima (zero imaginary frequency) or transition states (one imaginary frequency) by frequency analysis at the same level of theory (numerical hessian). Thermodynamics were computed within the Rigid-Rotor-Harmonic-Oscillator (RRHO) approximation at 298.15K and grouped in a term $G_{corr} = G - E(el)$ where G is the free energy of the species after all thermodynamics corrections and E(el) is the electronic potential energy of the species.

Single point energy corrections were carried out on all geometries using the level of theory targeted by the UMA-s-1 model, namely using the ωB97M-V functional with the def2-TZVPD basis set on all atoms.[15–19] The integration grid was set to DefGrid3 defaults, with TightSCF thresholds. Note that the SCF convergence for the molecular systems with diffuse functions was found to be more systematically robust when using the KDIIS algorithm followed by SOSCF, with switching thresholds left as defaults and starting from either a PModel guess or by reading the orbitals from a preliminary ωB97M-V/def2-TZVP calculation in difficult cases.

Conformational Sampling. Conformational searches were run for all species using the GOAT sampling algorithm as implemented in ORCA 6.0.0, using the UMA-s-1 model with ALPB solvation contributions in toluene. For sampling around transition states, the relevant coordinate (*e.g.* bond distance) was constrained during the conformational sampling. If a facile confirmational rearrangement was suspected, the geometry was submitted to a short molecular dynamics run. Initial velocities corresponding to a Boltzmann distribution for 100 K were set, and the system was left to equilibrate with a CSVR thermostat set to 300 K with a time constant of 100 fs. The time step was set to 1.0 fs and the trajectory was run for a total of 50 ps using the UMA-s-1 model with the ALPB solvation additive contribution for toluene. Evaporation of explicit solvent molecules or undesired breaking of bonds was prevented by implementing upper wall restraints between the atoms of interest associated with a spring strength constant of 400 kJ/mol/Å.

10.1 Geometry benchmarks

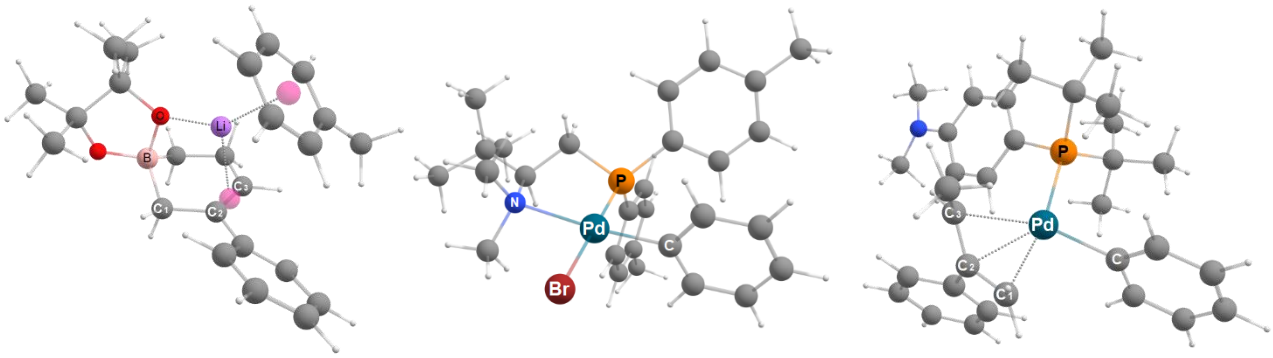

7a•Tol **16** **Model-1**

	X-Ray	UMA-s-1 vacuum	ωB97M-V[a] vacuum	UMA-s-1 ALPB[b]	ωB97M-V[a] CPCM[b]	TPSS-D3[a] CPCM[b]
			**7a•Tol**			
d(B–C1)	-	1.636	1.635	1.640	1.633	1.639
d(C2=C3)	-	1.346	1.346	1.346	1.345	1.358
d(Li–O)	-	1.786	1.790	1.789	1.807	1.805
d(Li–$Tol_{centroid}$)	-	2.167	2.195	2.201	2.144	2.174
d(Li–C2=$C3_{centroid}$)	-	2.343	2.346	2.351	2.380	2.364
			**16**			
d(Pd–P)	*2.200*	2.211	2.233	2.207	2.228	2.222
d(Pd–N)	*2.306*	2.316	2.316	2.325	2.299	2.299
d(Pd–Br)	*2.503*	2.475	2.483	2.515	2.519	2.526
d(Pd–C_{Ph})	*1.999*	1.983	1.990	1.988	1.996	2.012
			**Model-1**			
d(Pd–P)	-	2.365	2.375	2.378	2.380	2.361
d(Pd–C)	-	2.051	2.053	2.055	2.056	2.065
d(Pd–C1)	-	2.134	2.139	2.138	2.140	2.163
d(Pd–C2)	-	2.174	2.189	2.178	2.189	2.184
d(Pd–C3)	-	2.302	2.302	2.305	2.300	2.327
A(P–Pd–C)	-	100.8	101.4	101.0	101.6	101.3

Table S4. Comparison of calculated metrics with the UMA-s-1 model with DFT and X-ray structure reference geometries. [a] DFT calculations employ the def2-mTZVPP basis set on all atoms; [b]ALPB or CPCM denotes that the optimization was carried out with implicit toluene solvation with the corresponding model.

The performance of the UMA-s-1 model in conjunction with the ALPB solvation contribution in toluene was evaluated on 3 geometries, covering the substrate (**7a•Tol**), both ligands employed in the subsequent study as either an oxidative addition complex (**16**) or a π-allyl

complex (**Model-1**). Overall, UMA-s-1 is able to replicate the ωB97M-V calculated geometries, with only slight deviations on the critical bond lengths reported in Table S4. One has to keep in mind that DFT calculated geometries in this case made use of the def2-mTZVPP basis set to keep the calculation time tractable, and as such, slight deviations may be expected from the geometries that would be obtained with UMA's target level of theory with the heavier def2-TZVPD basis set. Of particular interest, UMA-s-1 outperforms TPSS-D3 in describing the immediate coordination sphere around Pd in both complexes, namely the Pd–Br, Pd–C_{Ph} and Pd–C_{allyl} bonds. The influence of implicit solvation with CPCM(Toluene) in the case of ωB97M-V can be seen mainly in the lengthening of the Pd–Br bond in **16**, a trend that the ALPB additive contribution is able to reproduce well.

Indicative timings, using 10 CPU cores, for one energy+gradient evaluation of **16** :

- 0.8 s for UMA-s-1 in vacuum
- 1.2 s for UMA-s-1 with ALPB contribution
- 60-100 s for TPSS-D3/ def2-mTZVPP with CPCM(toluene)
- >350 s for ωB97M-V /def2-mTZVPP with CPCM(toluene)

The overall accuracy and speedup observed for the UMA-s-1 model makes it a very pragmatic choice for the rest of this study, both for conformational sampling and to provide DFT quality geometries directly. Note that all pathways presented in the manuscript have been recalculated with single point energy evaluations at the ωB97M-V/def2-TZVPD level of theory, so that agreement between UMA and the target DFT level of theory can be monitored throughout.

10.2 Substrate speciation

Molecular dynamics parameters. The calculations were carried out with ORCA 6.0.0. The substrate dimer **[7a]$_2$** (or the monomeric **7a**) was centered on (0,0,0) and solvated with 25 explicit toluene molecules using the ORCA SOLVATOR's stochastic mode at the gfn2-xTB level of theory. The resulting xyz geometry was placed in a spherical cell centered on (0,0,0) with a radius of 13.50 Å for the dimeric system (or 10.56 Å for the monomeric system), corresponding approximately to toluene's experimental density of 0.867 at room temperature (Figure S13). The sphere is bounded by a harmonic wall with a spring strength constant of 50 kJ/mol/Å^2. Initial velocities corresponding to a Boltzmann distribution for 100 K were set, and the system was left to equilibrate with a CSVR thermostat set to 300 K with a time constant of 10 fs. The time step was set to 1.0 fs and the trajectory was run for a total of 50 ps using the UMA-s-1 model.

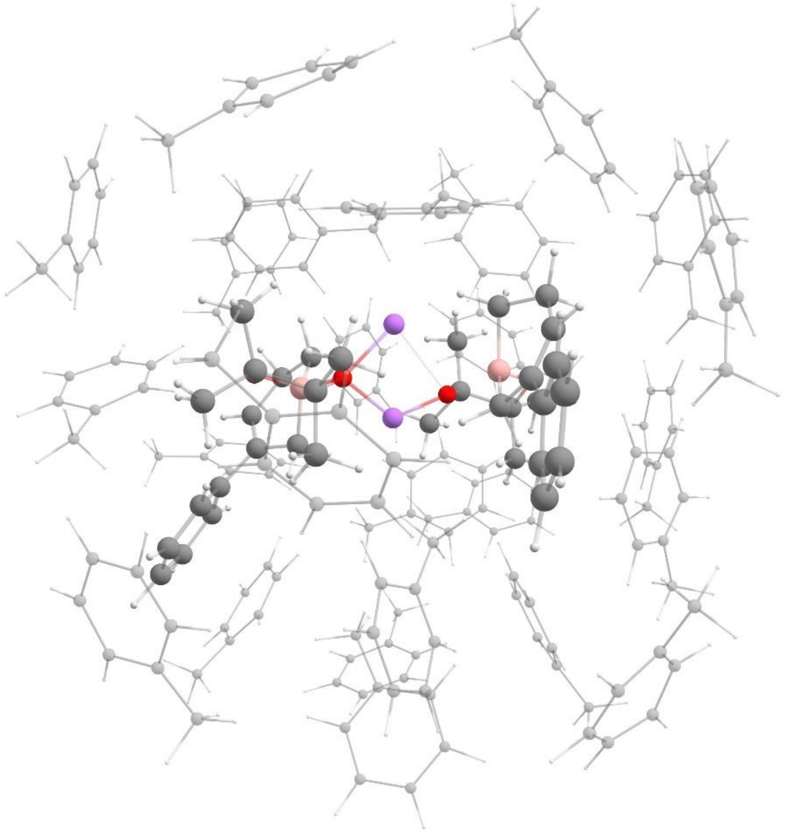

Figure S13. Snapshot (2.6 ps) of the spherical simulation cell with **[7a]$_2$** (in bold) and 25 toluene molecules (in transparency).

The final geometries for **[7a]$_2$•Tol** and **7a•Tol** were taken as the last frame from their respective molecular dynamics simulation, together with the explicit toluene molecule nearest Li, and reoptimized using the level of theory described above. The calculated data is presented in Table S5.

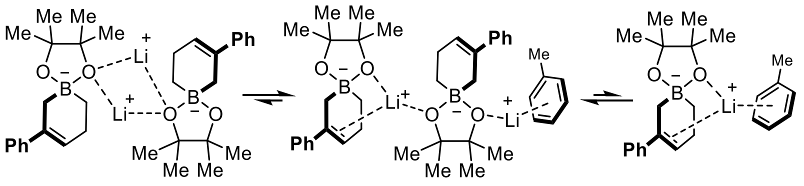

+3.8 [7a]$_2$	0.0 [7a]$_2$•Tol	+4.6 7a•Tol

Name	*E*(UMA-s-1)	*E*(DFT)	ΔG_{solv}	G_{corr}	*G*
**[7a]$_2$**	-1690.391097	-1690.392398	-0.050882	0.698102	-1689.745178
**[7a]$_2$•Tol**	-1961.949485	-1961.947722	-0.067058	0.819108	-1961.195671
**7a•Tol**	-1116.732356	-1116.732015	-0.035377	0.457650	-1116.309742
**Tol**	-271.5348506	-271.5348263	-0.007742	0.104222	-271.438346

Table S5. Calculated data for the geometries relevant to substrate speciation in toluene.

10.3 Pd-catalyzed C3-arylation pathway with Pd3

10.3.1 Main mechanism

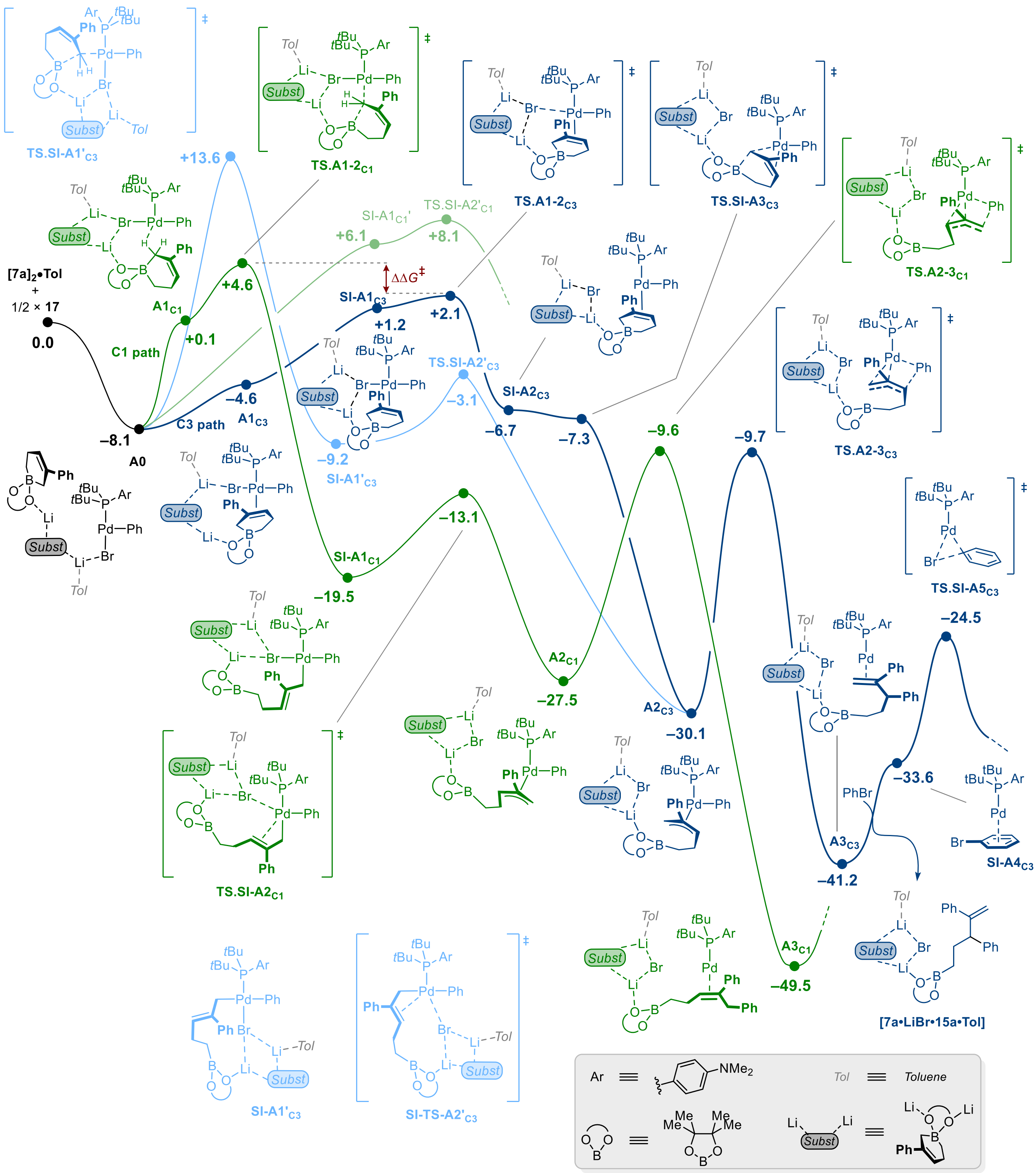

Figure S14. Full computed energy profile for the Pd-catalyzed C3-selective arylation of cyclic allylboronate **7a** using the APhos-based catalytic system (**Pd3**).

Name	*E*(UMA-s-1)	*E*(DFT)	ΔG_{solv}	G_{corr}	*G*
**[7a]$_2$•Tol**	-1961.949485	-1961.947722	-0.067058	0.819108	-1961.195671
**17**	-7912.329803	-7912.342346	-0.074389	0.915778	-7911.500956
**PhBr**	-2805.647964	-2805.647979	-0.009448	0.060044	-2805.597383
**A0**	-5918.144098	-5918.141925	-0.106901	1.289782	-5916.959044
**A1$_{C3}$**	-5918.145714	-5918.143410	-0.100818	1.290711	-5916.953518
**SI-A1$_{C3}$**	-5918.139028	-5918.137090	-0.100492	1.293361	-5916.944221
**TS.A1-2$_{C3}$**	-5918.136183	-5918.134285	-0.102358	1.293786	-5916.942858
**SI-A2$_{C3}$**	-5918.146659	-5918.142187	-0.105227	1.290604	-5916.956811
**TS.SI-A3$_{C3}$**	-5918.145871	-5918.143244	-0.104095	1.289621	-5916.957718
**A2$_{C3}$**	-5918.181373	-5918.182104	-0.104200	1.292198	-5916.994106
**TS.A2-3$_{C3}$**	-5918.143690	-5918.148662	-0.104213	1.291289	-5916.961585
**A3$_{C3}$**	-5918.196878	-5918.196284	-0.108567	1.293057	-5917.011795
**[7a•LiBr•15a•Tol]**	-4767.712898	-4767.710048	-0.078513	0.906403	-4766.882159
**SI-A4$_{C3}$**	-3956.115165	-3956.115162	-0.040246	0.440542	-3955.714866
**TS.SI-A5$_{C3}$**	-3956.097612	-3956.101283	-0.040230	0.441150	-3955.700363
**A1$_{C1}$**	-5918.137812	-5918.133792	-0.103962	1.291721	-5916.946033
**TS.A1-2$_{C1}$**	-5918.131832	-5918.126730	-0.102726	1.290681	-5916.938775
**SI-A1$_{C1}$**	-5918.169061	-5918.166960	-0.102962	1.292645	-5916.977277
**TS.SI-A2$_{C1}$**	-5918.155953	-5918.153817	-0.104249	1.291082	-5916.966985
**A2$_{C1}$**	-5918.175241	-5918.176479	-0.105240	1.291806	-5916.989913
**TS.A2-3$_{C1}$**	-5918.143678	-5918.147545	-0.104392	1.290510	-5916.961428
**A3$_{C1}$**	-5918.210388	-5918.210242	-0.105865	1.291079	-5917.025028
**SI-A1'$_{C1}$**	-5918.128633	-5918.125958	-0.101775	1.291312	-5916.936422
**TS.SI-A2'$_{C1}$**	-5918.123787	-5918.122254	-0.103380	1.292420	-5916.933215
**TS.SI-A1'$_{C3}$**	-5918.116770	-5918.112884	-0.104710	1.293170	-5916.924424
**SI-A1'$_{C3}$**	-5918.154267	-5918.151774	-0.103746	1.294636	-5916.960884
**TS.SI-A2'$_{C3}$**	-5918.140425	-5918.138782	-0.105410	1.293089	-5916.951104

Table S6. Calculated data for the C3-selective arylation pathway with **Pd3**.

Note that the release of the product was calculated as an adduct with one unreacted molecule of **7a** and one molecule of LiBr. Loss of LiBr was computed to be significantly uphill even at this stage of the pathway and even considering aggregation up to $[LiBr]_{32}$ (see section 9.3.3). While we were not able to probe further the kinetics and thermodynamics of LiBr loss and subsequent precipitation, it is likely that intermediate **[7a•LiBr•15a•Tol]** accumulates initially in the reaction mixture until it reaches a concentration sufficient to rearrange into (**[7a]$_2$•Tol**

and the C3-arylation product (**15a**) and simultaneously form lithium bromide aggregates that precipitate irreversibly.

Microkinetic modelling was performed based on the mechanism described in Figure S14 (see C3_arylation_model.cps and associated Excel file) using COPASI v4.45 (build 298). The product ratio predicted through microkinetic modelling was 1:68 in favor of the C3-arylation product, in exact agreement with the relative Boltzmann population ratio expected for two states of equal degeneracy and with an energy difference of 2.5 kcal/mol at 298.15 K. Here, this corresponds to the gap delineated in red between **TS.A1-2**$_{C3}$ and **TS-A1-2**$_{C1}$, confirming this early step in the mechanism is fully regiodetermining.

10.3.2 Compared energetics with monomeric 7a

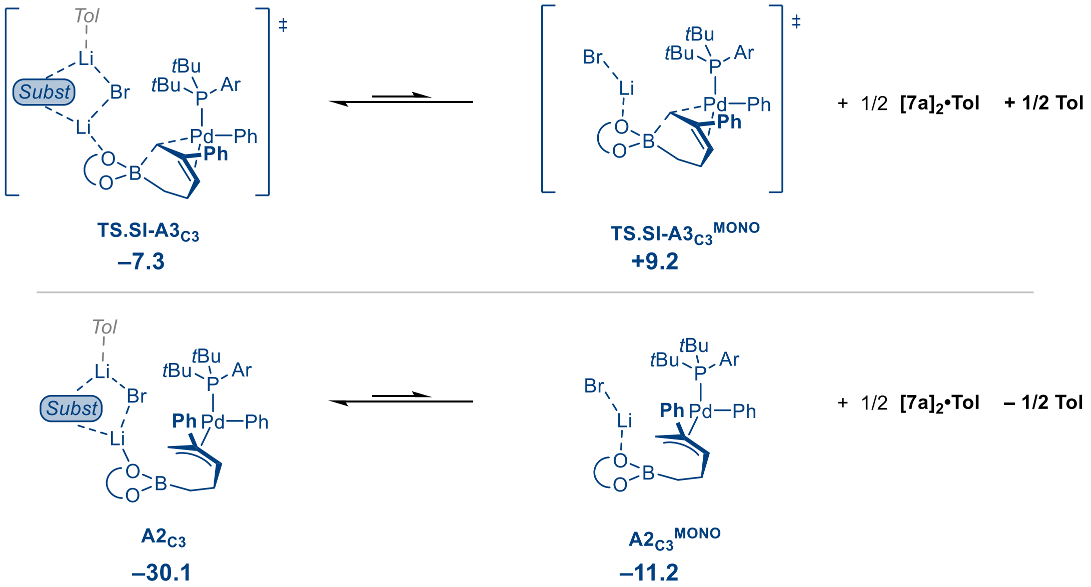

Name	*E*(UMA-s-1)	*E*(DFT)	ΔG_{solv}	G_{corr}	G
**[7a]$_2$•Tol**	-1961.949485	-1961.947722	-0.067058	0.819108	-1961.195671
**Tol**	-271.534851	-271.534826	-0.007742	0.104222	-271.438346
**TS.SI-A3$_{C3}$**	-5918.145871	-5918.143244	-0.104095	1.289621	-5916.957718
**A2$_{C3}$**	-5918.181373	-5918.182104	-0.104200	1.292198	-5916.994106
**TS.SI-A3$_{C3}^{MONO}$**	-4801.345945	-4801.345503	-0.072333	0.803368	-4800.614468
**A2$_{C3}^{MONO}$**	-4801.375331	-4801.377401	-0.073586	0.804073	-4800.646914

Table S7. Calculated data for the energy comparison of dimeric and monomeric substrate pathways at the ring-opening stage.

Table S7 shows the energetic penalty associated with the monomeric substrate pathway with respect to the dimeric pathway. An energy penalty of +16.5 kcal/mol is found at the ring-opening transition state **TS.SI-A3$_{C3}$** and +18.9 kcal/mol for the adjacent intermediate **A2$_{C3}$**.

10.3.3 Compared energetics with loss of LiBr – LiBr aggregation

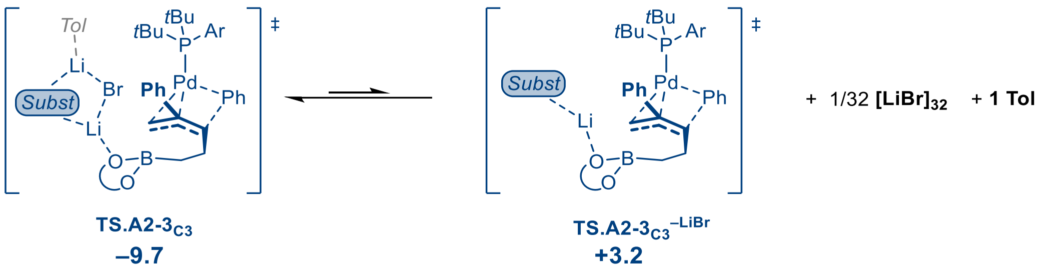

Name	*E*(UMA-s-1)	*E*(DFT)	ΔG_{solv}	G_{corr}	*G*
**TS.A2-3$_{C3}$**	-5918.143690	-5918.148662	-0.104213	1.291289	-5916.961585
**Tol**	-271.534851	-271.534826	-0.007742	0.104222	-271.438346
**TS.A2-3$_{C3}$$^{-LiBr}$**	-3064.899775	-3064.907794	-0.085425	1.168524	-3063.824695
**[LiBr]**	-2581.602002	-2581.602245	-0.016274	-0.020871	-2581.639390
**[LiBr]$_2$**	-5163.280980	-5163.281012	-0.022249	-0.027130	-5163.330391
**[LiBr]$_4$**	-10326.633780	-10326.633917	-0.030980	-0.030474	-10326.695370
**[LiBr]$_8$**	-20653.318814	-20653.314334	-0.049303	-0.038320	-20653.401958
**[LiBr]$_{16}$**	-41306.704271	-41306.694986	-0.080412	-0.052341	-41306.827738
**[LiBr]$_{32}$**	-82613.504517	-82613.507810	-0.111483	-0.078623	-82613.697916

Table S8. Calculated data for the energy comparison of the reductive elimination transition state **TS.A2-3$_{C3}$** with LiBr or with loss of LiBr.

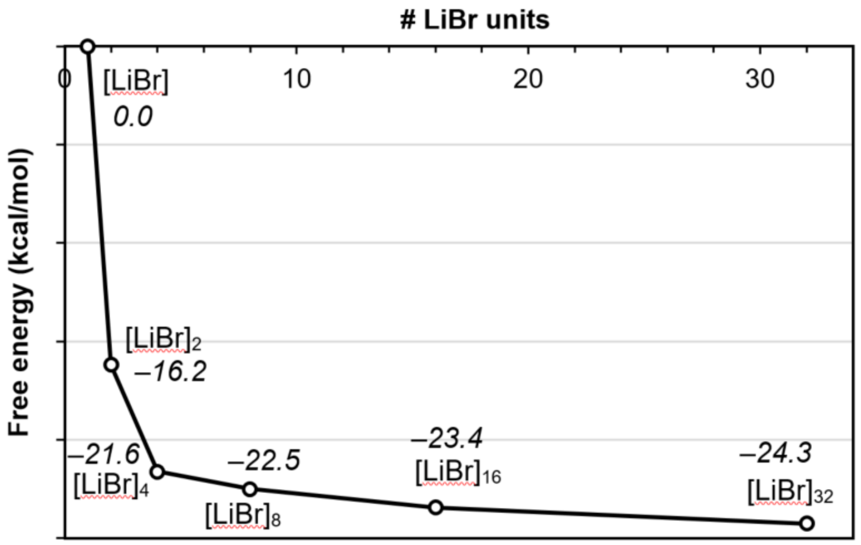

Figure S15. Stabilization energy of LiBr in implicit toluene with aggregation, normalized to one LiBr unit.

10.4 Pd-catalyzed C1-arylation pathway with L6/Pd1

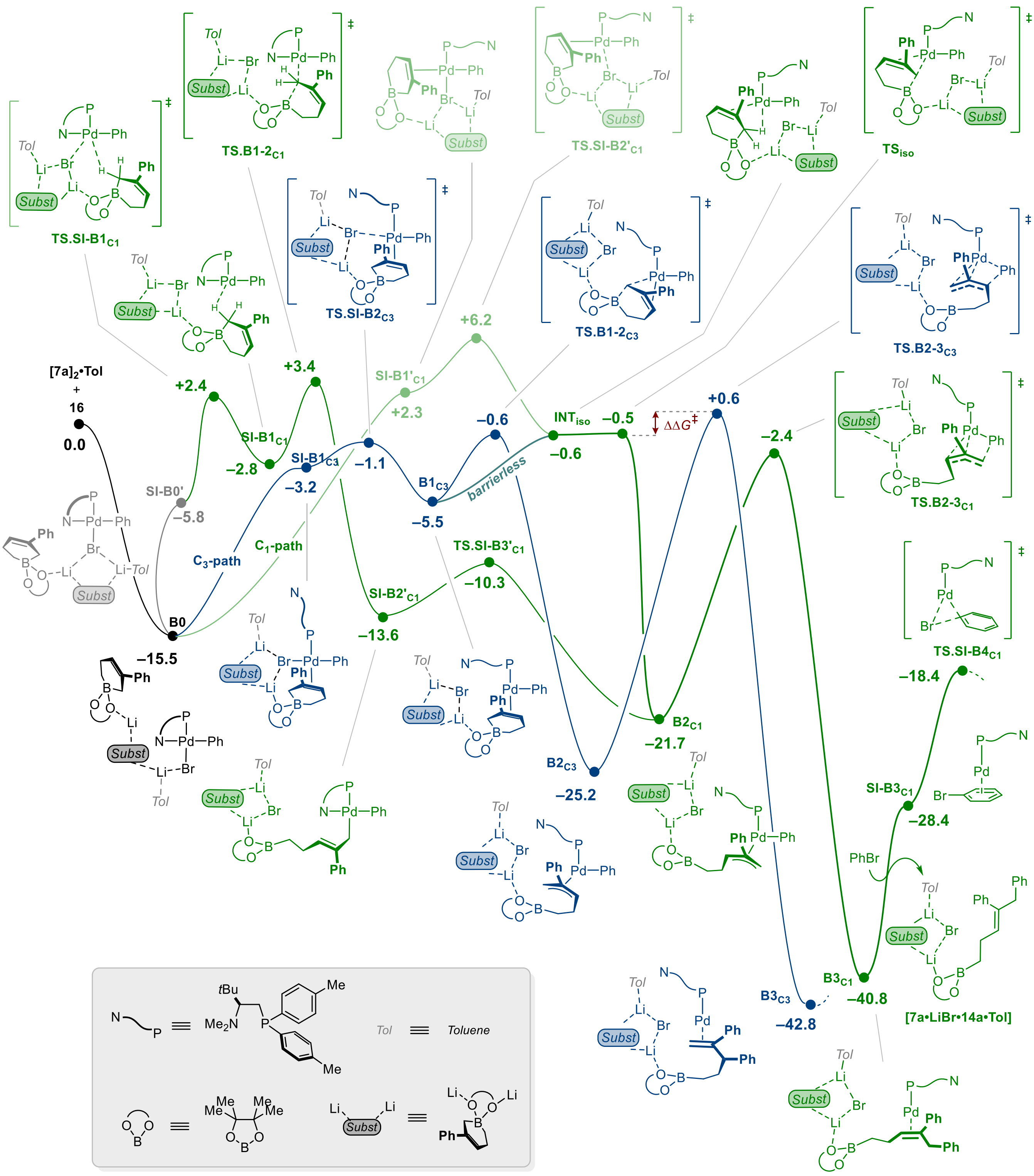

Figure S16. Full computed energy profile for the Pd-catalyzed C1-selective arylation of cyclic allylboronate **7a** using the catalytic system using **L6**/**Pd1**

Name	*E*(UMA-s-1)	*E*(DFT)	ΔG_{solv}	G_{corr}	*G*
**[7a]$_2$•Tol**	-1961.949485	-1961.947722	-0.067058	0.819108	-1961.195671
**16**	-4187.216892	-4187.217096	-0.049617	0.521404	-4186.745309
**B0**	-6149.221710	-6149.220980	-0.110035	1.365274	-6147.965741
**SI-B1$_{C3}$**	-6149.205905	-6149.204611	-0.107522	1.365985	-6147.946148
**TS.SI-B2$_{C3}$**	-6149.202134	-6149.200922	-0.108660	1.366835	-6147.942747
**B1$_{C3}$**	-6149.208900	-6149.204708	-0.111040	1.366001	-6147.949747
**TS.B1-2$_{C3}$**	-6149.202711	-6149.200009	-0.108383	1.366530	-6147.941862
**B2$_{C3}$**	-6149.235526	-6149.235833	-0.110457	1.365101	-6147.981188
**TS.B2-3$_{C3}$**	-6149.191682	-6149.193275	-0.110919	1.364154	-6147.940041
**B3$_{C3}$**	-6149.261066	-6149.261206	-0.116425	1.368436	-6148.009195
**B1$_{C1}$**	-6149.206762	-6149.202994	-0.110101	1.367629	-6147.945465
**TS.B1-2$_{C1}$**	-6149.199785	-6149.193776	-0.108527	1.367033	-6147.935270
**B2$_{C1}$**	-6149.229643	-6149.230765	-0.111134	1.366332	-6147.975567
**TS.SI-B2-3$_{C1}$**	-6149.195436	-6149.200558	-0.110407	1.366175	-6147.944789
**B3$_{C1}$**	-6149.266403	-6149.262261	-0.112793	1.368991	-6148.006063
**[7a•LiBr•14a•Tol]**	-4767.720512	-4767.717803	-0.077776	0.907800	-4766.887779
**SI-B3$_{C1}$**	-4187.164887	-4187.164488	-0.045936	0.514605	-4186.695819
**TS.SI-B4$_{C1}$**	-4187.146563	-4187.149200	-0.046061	0.515333	-4186.679927
**INT$_{iso}$**	-6149.199003	-6149.193645	-0.113190	1.364939	-6147.941895
**TS$_{iso}$**	-6149.198905	-6149.194492	-0.112257	1.365049	-6147.941701
**SI-B0’**	-6149.210506	-6149.209109	-0.108415	1.367264	-6147.950260
**TS.SI-B1$_{C1}$**	-6149.201600	-6149.199071	-0.108205	1.370058	-6147.937217
**SI-B1’$_{C1}$**	-6149.196329	-6149.194356	-0.107915	1.364939	-6147.937331
**TS.SI-B2’$_{C1}$**	-6149.191848	-6149.190209	-0.109673	1.368856	-6147.931026
**SI-B2’$_{C1}$**	-6149.225645	-6149.222220	-0.108294	1.367938	-6147.962576
**TS.SI-B3’$_{C1}$**	-6149.209823	-6149.209503	-0.113282	1.365381	-6147.957405

Table S9. Calculated data for the C1-selective arylation pathway with **L6**/**Pd1**.

Microkinetic modelling was performed based on the mechanism described in Figure S16 (see C1_arylation_model.cps and associated Excel file) using COPASI v4.45 (build 298). The product ratio predicted through microkinetic modelling was 3:1 in favor of the C1-arylation product, a slight deviation from the predicted Boltzmann population ratio based on the energy difference in red defined by **TS$_{iso}$** and **TS.B2-3$_{C3}$** of 4.8:1 at 353.15 K. This suggests that regioselectivity determination is dominated by these transition states but equilibration effects in the interconversion process do play an additional role in the final product distribution.

10.5 Deviation between UMA-s-1, UMA-m-1, DFT.

Name	*E*(UMA-s-1)	*E*(UMA-m-1)	*E*(DFT)	deviation UMA-s-1	deviation UMA-m-1
**$[7a]_2$•Tol**	-1961.949485	-1961.949355	-1961.947722	-1.1	-1.0
**17**	-7912.329803	-7912.338491	-7912.342346	7.9	2.4
**PhBr**	-2805.647964	-2805.647994	-2805.647979	0.0	0.0
**A0**	-5918.144098	-5918.144066	-5918.141925	-1.4	-1.3
**$A1_{C3}$**	-5918.145714	-5918.146027	-5918.143410	-1.4	-1.6
**SI-$A1_{C3}$**	-5918.139028	-5918.140346	-5918.137090	-1.2	-2.0
**TS.A1-2_{C3}**	-5918.136183	-5918.137373	-5918.134285	-1.2	-1.9
**SI-$A2_{C3}$**	-5918.146659	-5918.145772	-5918.142187	-2.8	-2.2
**TS.SI-$A3_{C3}$**	-5918.145871	-5918.146555	-5918.143244	-1.6	-2.1
**$A2_{C3}$**	-5918.181373	-5918.183433	-5918.182104	0.5	-0.8
**TS.A2-3_{C3}**	-5918.143690	-5918.149109	-5918.148662	3.1	-0.3
**$A3_{C3}$**	-5918.196878	-5918.197638	-5918.196284	-0.4	-0.8
**[7a•LiBr• 15a•Tol]**	-4767.712898	-4767.711743	-4767.710048	-1.8	-1.1
**SI-$A4_{C3}$**	-3956.115165	-3956.115557	-3956.115162	0.0	-0.2
**TS.SI-$A5_{C3}$**	-3956.097612	-3956.100340	-3956.101283	2.3	0.6
**$A1_{C1}$**	-5918.137812	-5918.136468	-5918.133792	-2.5	-1.7
**TS.A1-2_{C1}**	-5918.131832	-5918.130270	-5918.126730	-3.2	-2.2
**SI-$A1_{C1}$**	-5918.169061	-5918.169858	-5918.166960	-1.3	-1.8
**TS.SI-$A2_{C1}$**	-5918.155953	-5918.156916	-5918.153817	-1.3	-1.9
**$A2_{C1}$**	-5918.175241	-5918.178419	-5918.176479	0.8	-1.2
**TS.A2-3_{C1}**	-5918.143678	-5918.149550	-5918.147545	2.4	-1.3
**$A3_{C1}$**	-5918.210388	-5918.211719	-5918.210242	-0.1	-0.9
**SI-A1'$_{C1}$**	-5918.128633	-5918.129068	-5918.125958	-1.7	-2.0
**TS.SI-A2'$_{C1}$**	-5918.123787	-5918.125034	-5918.122254	-1.0	-1.7

Table S10. Comparison of the calculated data for the C3-selective arylation pathway with **Pd3**, with the small model UMA-s-1, the medium model UMA-m-1 *vs* the DFT reference. Deviation between the UMA models and the DFT reference in kcal/mol.

Overall, switching to the UMA-m-1 model reduces the extent of the largest deviations, illustrated here with the energy of Pd dimer **17**. The RMSD for UMA-s-1 and UMA-m-1 across this table are 1.71 and 1.38 kcal/mol, respectively. Of note, the UMA-m-1 model seems to nearly consistently give absolute energy lower than the DFT reference: the average deviation for UMA-m-1 against the DFT reference is -1.125 kcal/mol. With this shift compensated, the RMSD for UMA-m-1 falls to 0.76 kcal/mol, while for UMA-s-1 RMSD falls to 1.63 kcal/mol after application of a smaller shift of ca. 0.3 kcal/mol. This suggests UMA-m-1 indeed performs

better when considering relative free energy values. The same observations can be made for the data pertaining to the C1-selective arylation pathway shown below in Table S11.

Name	*E*(UMA-s-1)	*E*(UMA-m-1)	*E*(DFT)	deviation UMA-s-1	deviation UMA-m-1
**[7a]$_2$•Tol**	-1961.949485	-1961.949355	-1961.947722	-1.1	-1.0
**16**	-4187.216892	-4187.217563	-4187.217096	0.1	-0.3
**B0**	-6149.221710	-6149.223500	-6149.220980	-0.5	-1.6
**SI-B1$_{C3}$**	-6149.205905	-6149.207514	-6149.204611	-0.8	-1.8
**TS.SI-B2$_{C3}$**	-6149.202134	-6149.204043	-6149.200922	-0.8	-2.0
**B1$_{C3}$**	-6149.208900	-6149.208393	-6149.204708	-2.6	-2.3
**TS.B1-2$_{C3}$**	-6149.202711	-6149.203949	-6149.200009	-1.7	-2.5
**B2$_{C3}$**	-6149.235526	-6149.237106	-6149.235833	0.2	-0.8
**TS.B2-3$_{C3}$**	-6149.191682	-6149.194258	-6149.193275	1.0	-0.6
**B3$_{C3}$**	-6149.261066	-6149.263698	-6149.261206	0.1	-1.6
**B1$_{C1}$**	-6149.206762	-6149.205628	-6149.202994	-2.4	-1.7
**TS.B1-2$_{C1}$**	-6149.199785	-6149.197182	-6149.193776	-3.8	-2.1
**B2$_{C1}$**	-6149.229643	-6149.232265	-6149.230765	0.7	-0.9
**TS.SI-B2-3$_{C1}$**	-6149.195436	-6149.201816	-6149.200558	3.2	-0.8
**B3$_{C1}$**	-6149.266403	-6149.264761	-6149.262261	-2.6	-1.6
**[7a•LiBr• 14a•Tol]**	-4767.720512	-4767.719833	-4767.717803	-1.7	-1.3
**SI-B3$_{C1}$**	-4187.164887	-4187.164474	-4187.164488	-0.3	0.0
**TS.SI-B4$_{C1}$**	-4187.146563	-4187.148465	-4187.149200	1.7	0.5
**INT$_{iso}$**	-6149.199003	-6149.196758	-6149.193645	-3.4	-2.0
**TS$_{iso}$**	-6149.198905	-6149.197297	-6149.194492	-2.8	-1.8
**SI-B0'**	-6149.210506	-6149.211752	-6149.209109	-0.9	-1.7
**TS.SI-B1'$_{C1}$**	-6149.201600	-6149.201837	-6149.199071	-1.6	-1.7
**SI-B1'$_{C1}$**	-6149.196329	-6149.197212	-6149.194356	-1.2	-1.8
**TS.SI-B2'$_{C1}$**	-6149.191848	-6149.193164	-6149.190209	-1.0	-1.9
**SI-B2'$_{C1}$**	-6149.225645	-6149.225763	-6149.222220	-2.1	-2.2
**TS.SI-B3'$_{C1}$**	-6149.209823	-6149.211386	-6149.209503	-0.2	-1.2

Table S11. Comparison of the calculated data for the C1-selective arylation pathway with **L6**/**Pd1**, with the small model UMA-s-1, the medium model UMA-m-1 *vs* the DFT reference. Deviation between the UMA models and the DFT reference in kcal/mol.

10.6 Deviation between DFT and g-xTB

Table S12 reports the g-xTB calculated data,[20] to which thermodynamic contributions were added to obtain *G*(g-xTB). As g-xTB targets a different basis set reference as UMA and therefore the reference DFT level of theory herein, direct comparison of absolute energies is precluded. Instead, a comparison on relative energies throughout the pathway can be made, with values reported in Table S13.

Name	*E*(DFT)	*E*(g-xTB)	ΔG_{solv}	G_{corr}	*G*(DFT)	*G*(g-xTB)
**[7a]$_2$•Tol**	-1961.947722	-1961.971340	-0.067058	0.819108	-1961.195671	-1961.219290
**17**	-7912.342346	-7912.380882	-0.074389	0.915778	-7911.500956	-7911.539492
**PhBr**	-2805.647979	-2805.651421	-0.009448	0.060044	-2805.597383	-2805.600826
**A0**	-5918.141925	-5918.182409	-0.106901	1.289782	-5916.959044	-5916.999528
**A1$_{C3}$**	-5918.143410	-5918.196123	-0.100818	1.290711	-5916.953518	-5917.006230
**SI-A1$_{C3}$**	-5918.137090	-5918.182998	-0.100492	1.293361	-5916.944221	-5916.990129
**TS.A1-2$_{C3}$**	-5918.134285	-5918.176999	-0.102358	1.293786	-5916.942858	-5916.985572
**SI-A2$_{C3}$**	-5918.142187	-5918.186583	-0.105227	1.290604	-5916.956811	-5917.001207
**TS.SI-A3$_{C3}$**	-5918.143244	-5918.185442	-0.104095	1.289621	-5916.957718	-5916.999916
**A2$_{C3}$**	-5918.182104	-5918.222876	-0.104200	1.292198	-5916.994106	-5917.034877
**TS.A2-3$_{C3}$**	-5918.148662	-5918.190175	-0.104213	1.291289	-5916.961585	-5917.003099
**A3$_{C3}$**	-5918.196284	-5918.229340	-0.108567	1.293057	-5917.011795	-5917.044851
**[7a•LiBr• 15a•Tol]**	-4767.710048	-4767.730874	-0.078513	0.906403	-4766.882159	-4766.902984
**SI-A4$_{C3}$**	-3956.115162	-3956.133262	-0.040246	0.440542	-3955.714866	-3955.732966
**TS.SI-A5$_{C3}$**	-3956.101283	-3956.130215	-0.040230	0.441150	-3955.700363	-3955.729294
**A1$_{C1}$**	-5918.133792	-5918.179868	-0.103962	1.291721	-5916.946033	-5916.992109
**TS.A1-2$_{C1}$**	-5918.126730	-5918.171773	-0.102726	1.290681	-5916.938775	-5916.983819
**SI-A1$_{C1}$**	-5918.166960	-5918.214710	-0.102962	1.292645	-5916.977277	-5917.025027
**TS.SI-A2$_{C1}$**	-5918.153817	-5918.201152	-0.104249	1.291082	-5916.966985	-5917.014320
**A2$_{C1}$**	-5918.176479	-5918.214236	-0.105240	1.291806	-5916.989913	-5917.027669
**TS.A2-3$_{C1}$**	-5918.147545	-5918.188046	-0.104392	1.290510	-5916.961428	-5917.001929
**A3$_{C1}$**	-5918.210242	-5918.239322	-0.105865	1.291079	-5917.025028	-5917.054108
**SI-A1'$_{C1}$**	-5918.125958	-5918.170843	-0.101775	1.291312	-5916.936422	-5916.981306
**TS.SI-A2'$_{C1}$**	-5918.122254	-5918.163661	-0.103380	1.292420	-5916.933215	-5916.974622

Table S12. Calculated data for the C3-selective arylation pathway with **Pd3** with g-xTB against the DFT reference.

Name	*G*(DFT)	*G*(g-xTB)	Δ*G*(DFT)	Δ*G*(g-xTB)	Dev.
**[7a]$_2$•Tol**	-1961.195671	-1961.219290	-	-	-
**17**	-7911.500956	-7911.539492	-	-	-
**PhBr**	-2805.597383	-2805.600826	-	-	-
**A0**	-5916.959044	-5916.999528	-8.1	-6.6	1.5
**A1$_{C3}$**	-5916.953518	-5917.006230	-4.6	-10.8	-6.2
**SI-A1$_{C3}$**	-5916.944221	-5916.990129	1.2	-0.7	-1.9
**TS.A1-2$_{C3}$**	-5916.942858	-5916.985572	2.1	2.2	0.1
**SI-A2$_{C3}$**	-5916.956811	-5917.001207	-6.7	-7.6	-0.9
**TS.SI-A3$_{C3}$**	-5916.957718	-5916.999916	-7.3	-6.8	0.4
**A2$_{C3}$**	-5916.994106	-5917.034877	-30.1	-28.8	1.3
**TS.A2-3$_{C3}$**	-5916.961585	-5917.003099	-9.7	-8.8	0.9
**A3$_{C3}$**	-5917.011795	-5917.044851	-41.2	-35.0	6.2
**[7a•LiBr•15a•Tol]**	-4766.882159	-4766.902984	-	-	-
**SI-A4$_{C3}$**	-3955.714866	-3955.732966	-33.6	-28.9	4.6
**TS.SI-A5$_{C3}$**	-3955.700363	-3955.729294	-24.5	-26.6	-2.2
**A1$_{C1}$**	-5916.946033	-5916.992109	0.1	-1.9	-2
**TS.A1-2$_{C1}$**	-5916.938775	-5916.983819	4.6	3.3	-1.4
**SI-A1$_{C1}$**	-5916.977277	-5917.025027	-19.5	-22.6	-3.1
**TS.SI-A2$_{C1}$**	-5916.966985	-5917.014320	-13.1	-15.9	-2.8
**A2$_{C1}$**	-5916.989913	-5917.027669	-27.5	-24.2	3.2
**TS.A2-3$_{C1}$**	-5916.961428	-5917.001929	-9.6	-8.1	1.5
**A3$_{C1}$**	-5917.025028	-5917.054108	-49.5	-40.8	8.7
**SI-A1'$_{C1}$**	-5916.936422	-5916.981306	6.1	4.9	-1.3
**TS.SI-A2'$_{C1}$**	-5916.933215	-5916.974622	8.1	9.0	0.9

Table S13. Calculated data for the C3-selective arylation pathway with **Pd3** with g-xTB against the DFT reference. Relative free energies in kcal/mol.

We found g-xTB also performs well against the DFT calculated energy reference. The method effectively captures the entire pathway for the C3-arylation pathway with Pd3 qualitatively and, with few exceptions, quantitatively. This will constitute a physically grounded and robust alternative to consider for future studies for fast conformational sampling and reactivity exploration, even with challenging transition metal systems.

11. References

(1) C.Zhang, and C. Mazet, *Org. Lett.*, 2024, **26**, 5386–5390.

(2) N. C. Bruno, M. T. Tudge and S. L. Buchwald, *Chem. Sci.*, 2013, **4**, 916–920.

(3) K. Li, B. Leforestier, A. I. Poblador-Bahamonde, C. Besnard, L. Guénée, S. Kucher and C. Mazet, *ACS Catal.*, 2025, **15**, 392–402.

(4) K. Harano, R. M. Gorgoll and E. Nakamura, *Chem. Commun.*, 2013, **49**, 7629–7631.

(5) R. Ruzi, K. Liu, C. Zhu and J. Xie, *Nat Commun*, 2020, **11**, 3312.

(6) X. Yan, M. Liu, D. Pan, Q. Wang, Q. Tang, Y. Dai, P. Hu, B. Wang, G. Huang and F. Song, *Angew. Chem. Int. Ed.*, 2024, **63**, e202317433.

(7) P. Jolly, N. Fleary-Roberts, S. Sullivan, E. Doni, S. Zhou and J. A. Murphy, *Org. Biomol. Chem.*, 2012, **10**, 5807–5810.

(8) R. Mandal, B. Emayavaramban and B. Sundararaju, *Org. Lett.*, 2018, **20**, 2835−2838.

(9) F. Neese, *WIREs Comput. Mol. Sci.*, 2012, **2**, 73–78.

(10) F. Neese, *WIREs Comput. Mol. Sci.*, 2022, **12**, e1606.

(11) F. Neese, *WIREs Comput. Mol. Sci.*, 2025, **15**, e70019.

(12) B. M. Wood, M. Dzamba, X. Fu, M. Gao, M. Shuaibi, L. Barroso-Luque, K. Abdelmaqsoud, V. Gharakhanyan, J. R. Kitchin, D. S. Levine, K. Michel, A. Sriram, T. Cohen, A. Das, A. Rizvi, S. J. Sahoo, Z. W. Ulissi and C. L. Zitnick, UMA: A Family of Universal Models for Atoms. *arXiv* 2025.

(13) C. Bannwarth, S. Ehlert and S. Grimme, *J. Chem. Theory Comput.*, 2019, **15**, 1652–1671.

(14) S. Ehlert, M. Stahn, S. Spicher and S. Grimme, *J. Chem. Theory Comput.*, 2021, **17**, 4250–4261.

(15) O. A. Vydrov and T. Van Voorhis, *J. Chem. Phys.*, 2010, **133**, 244103.

(16) W. Hujo and S. Grimme, *J. Chem. Theory Comput.*, 2011, **7**, 3866–3871.

(17) N. Mardirossian and M. Head-Gordon, *J. Chem. Phys.*, 2016, **144**, 214110.

(18) F. Weigend and R. Ahlrichs, *Phys. Chem. Chem. Phys.*, 2005, **7**, 3297.

(19) F. Weigend, *Phys. Chem. Chem. Phys.*, 2006, **8**, 1057.

(20) T. Froitzheim, M. Müller, A. Hansen and S. Grimme, *ChemRxiv* Chemistry June 24, 2025.

12. NMR spectra

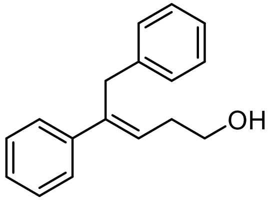

14a

^{1}H NMR (400 MHz, 298 K, $CDCl_3$)

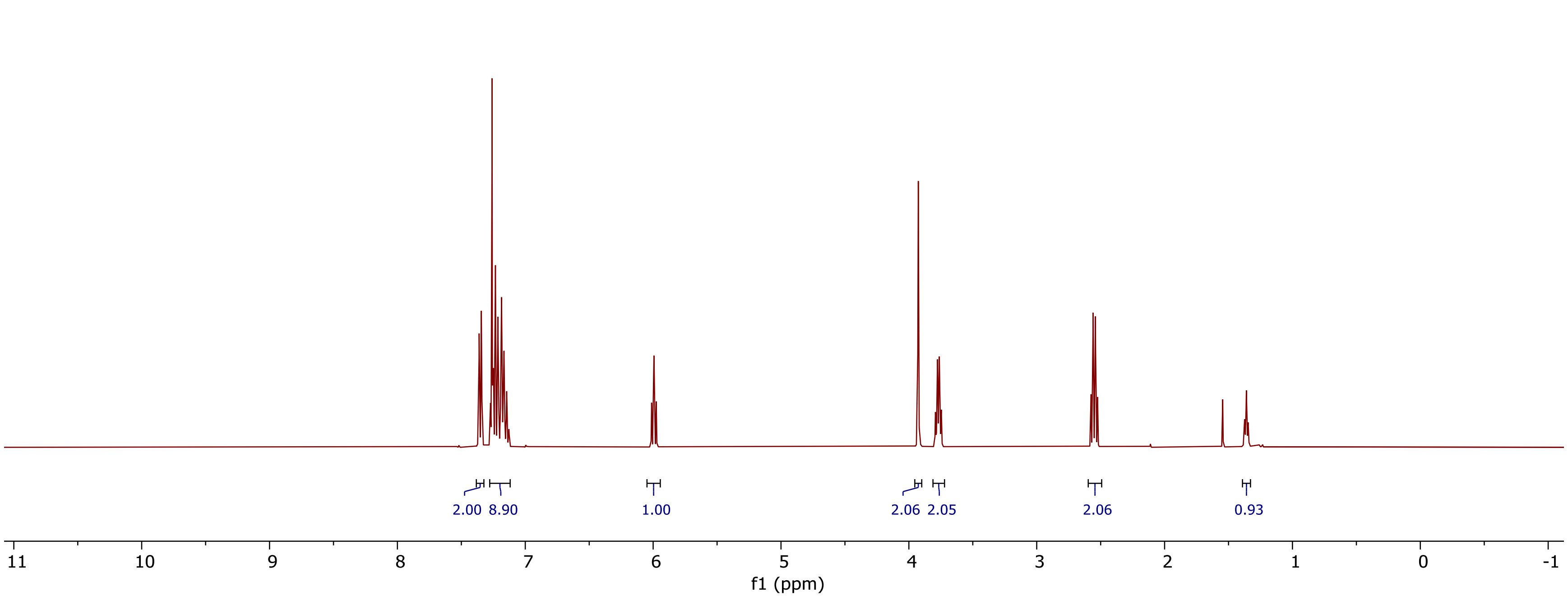

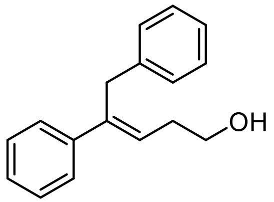

14a

$^{13}C\{^1H\}$ NMR (101 MHz, 298 K, $CDCl_3$)

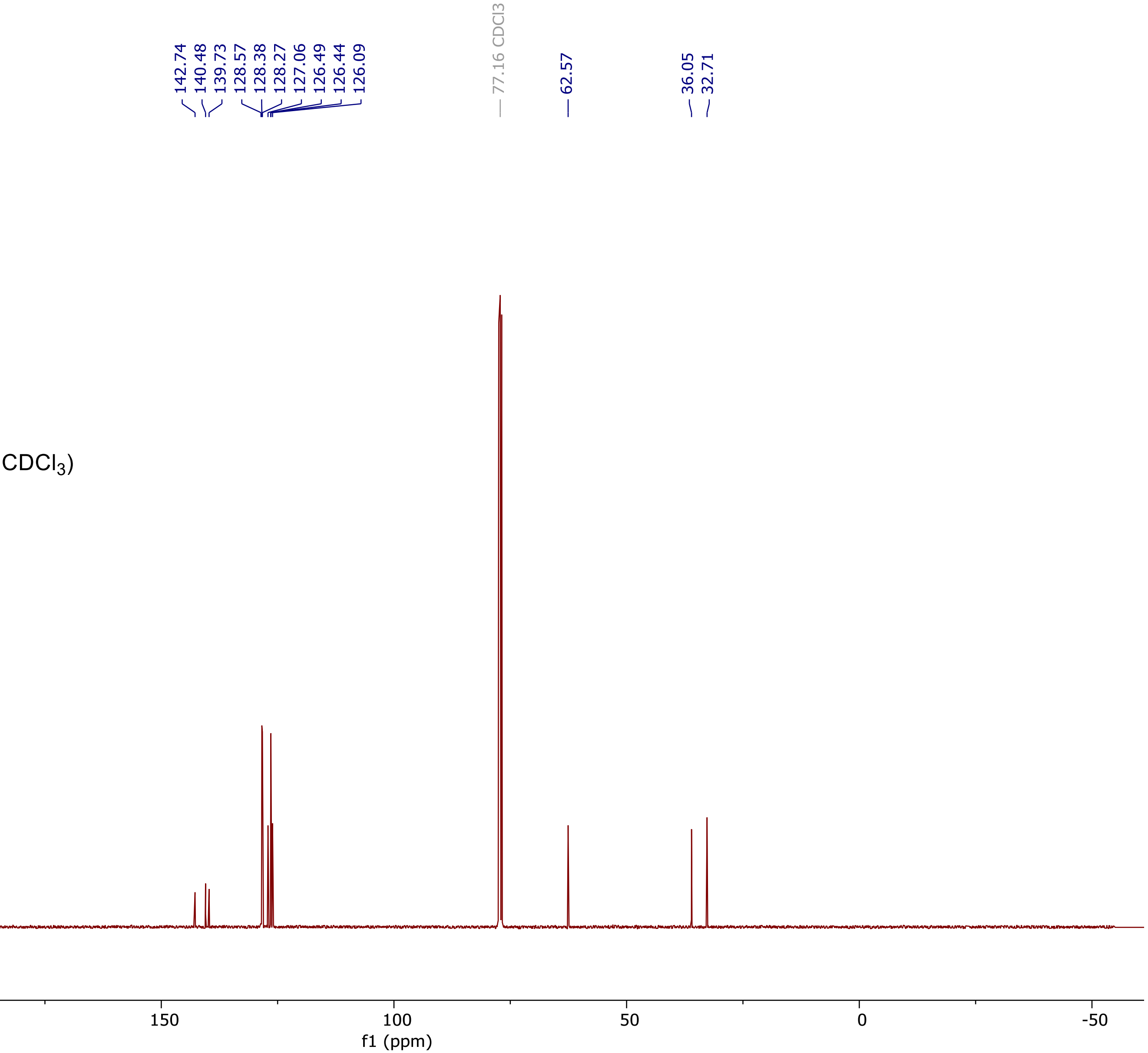

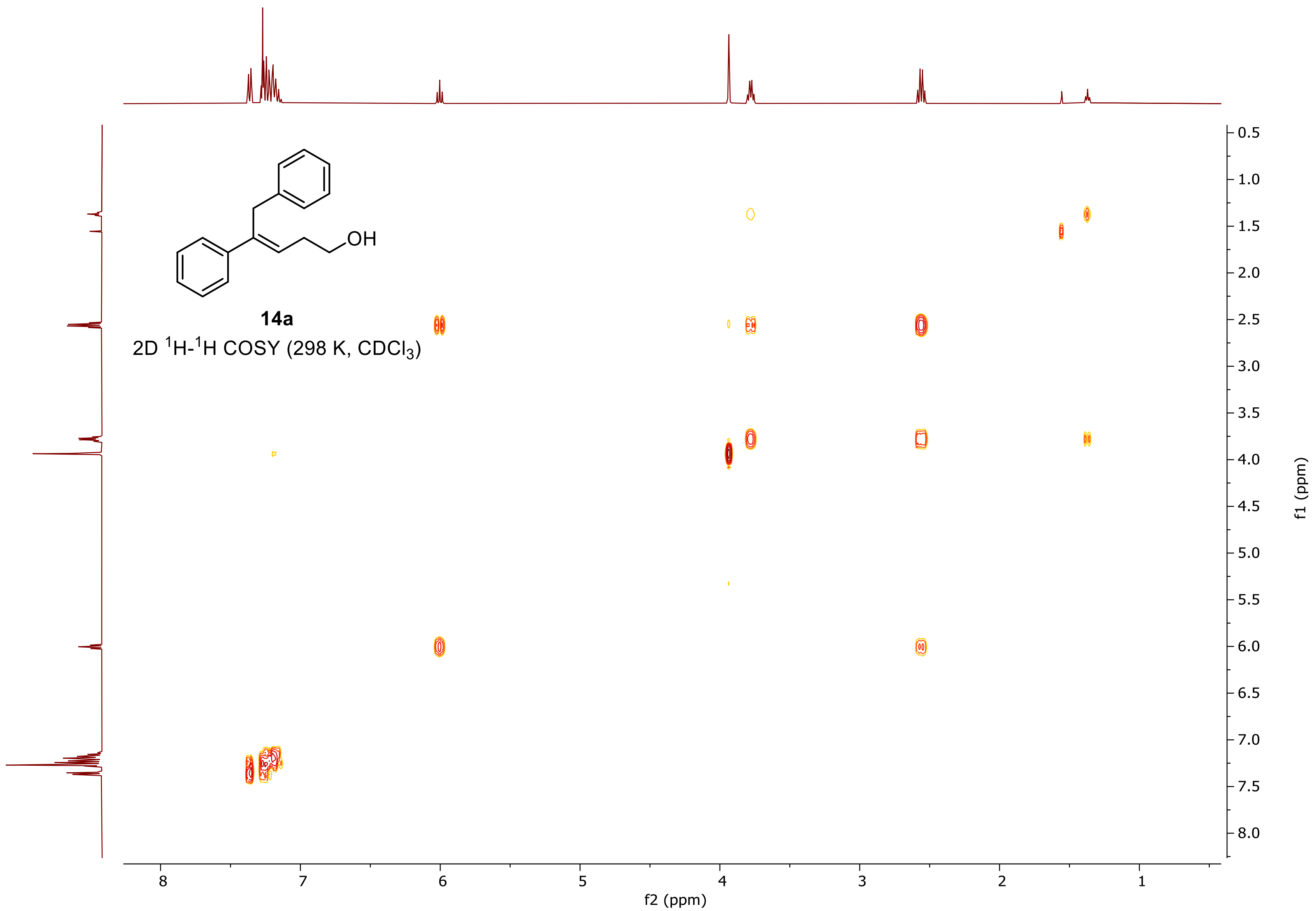

14a

2D ^{1}H-^{1}H COSY (298 K, $CDCl_3$)

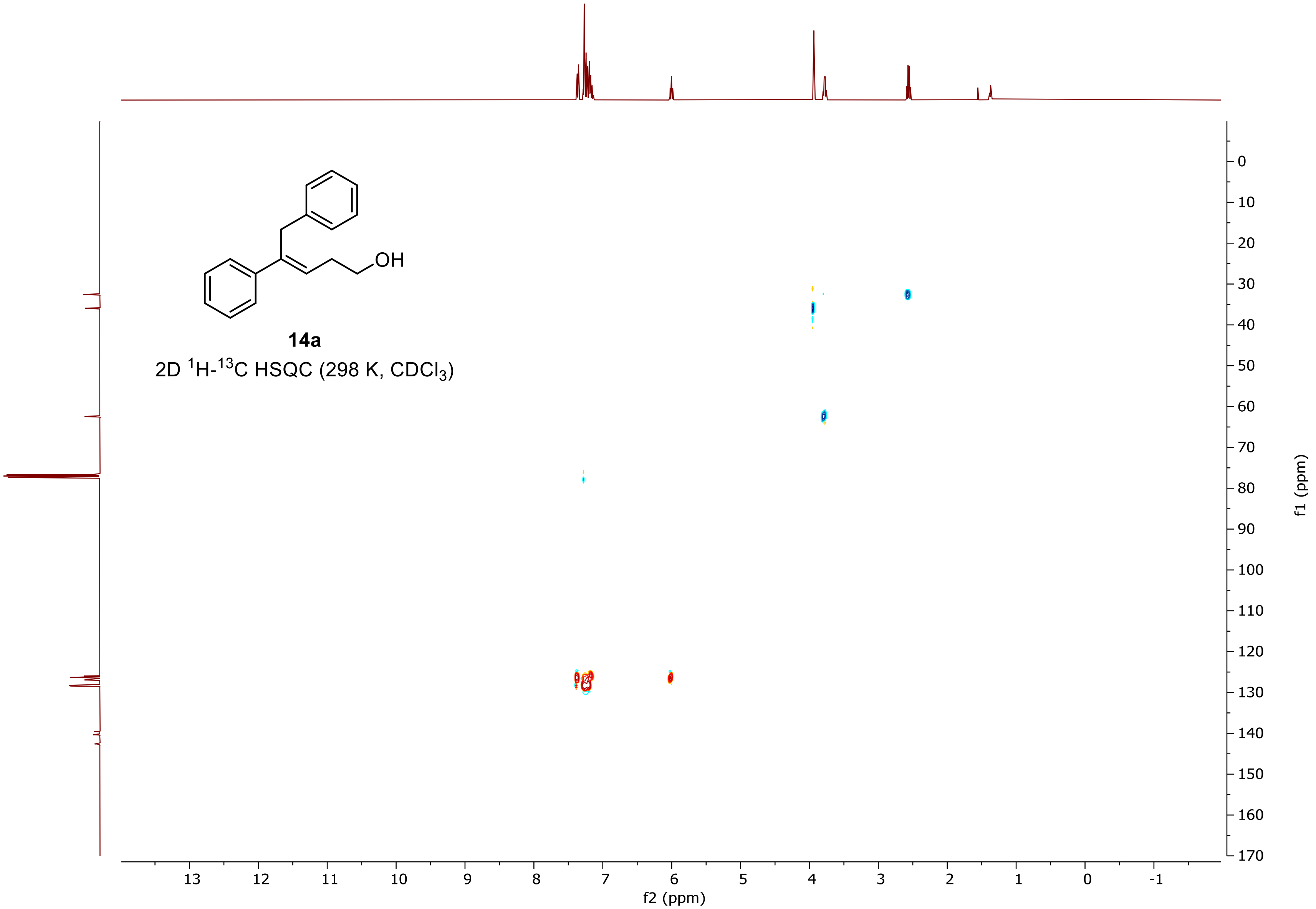

14a

2D ^{1}H-^{13}C HSQC (298 K, $CDCl_3$)

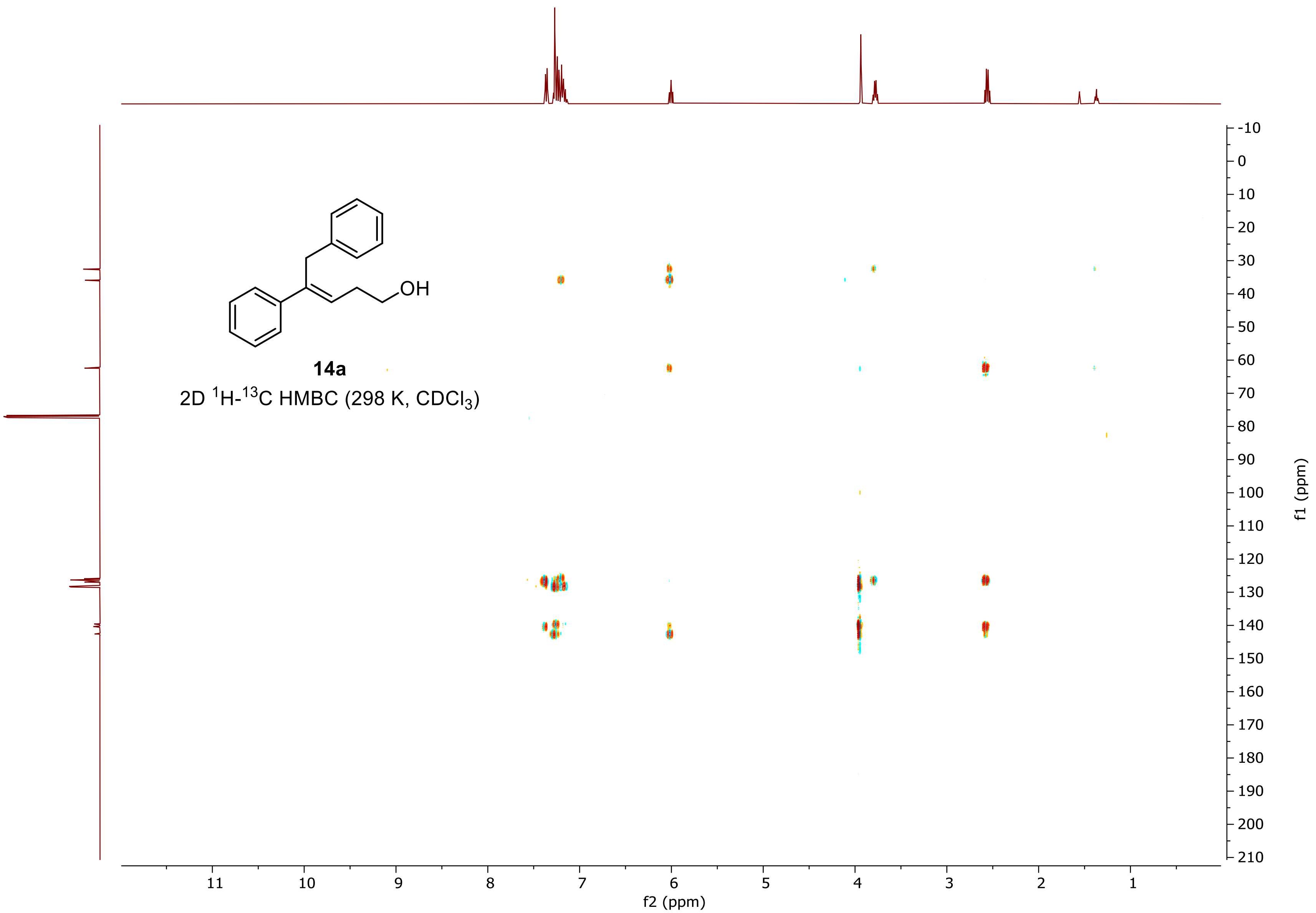

14a

2D ^{1}H-^{13}C HMBC (298 K, $CDCl_3$)

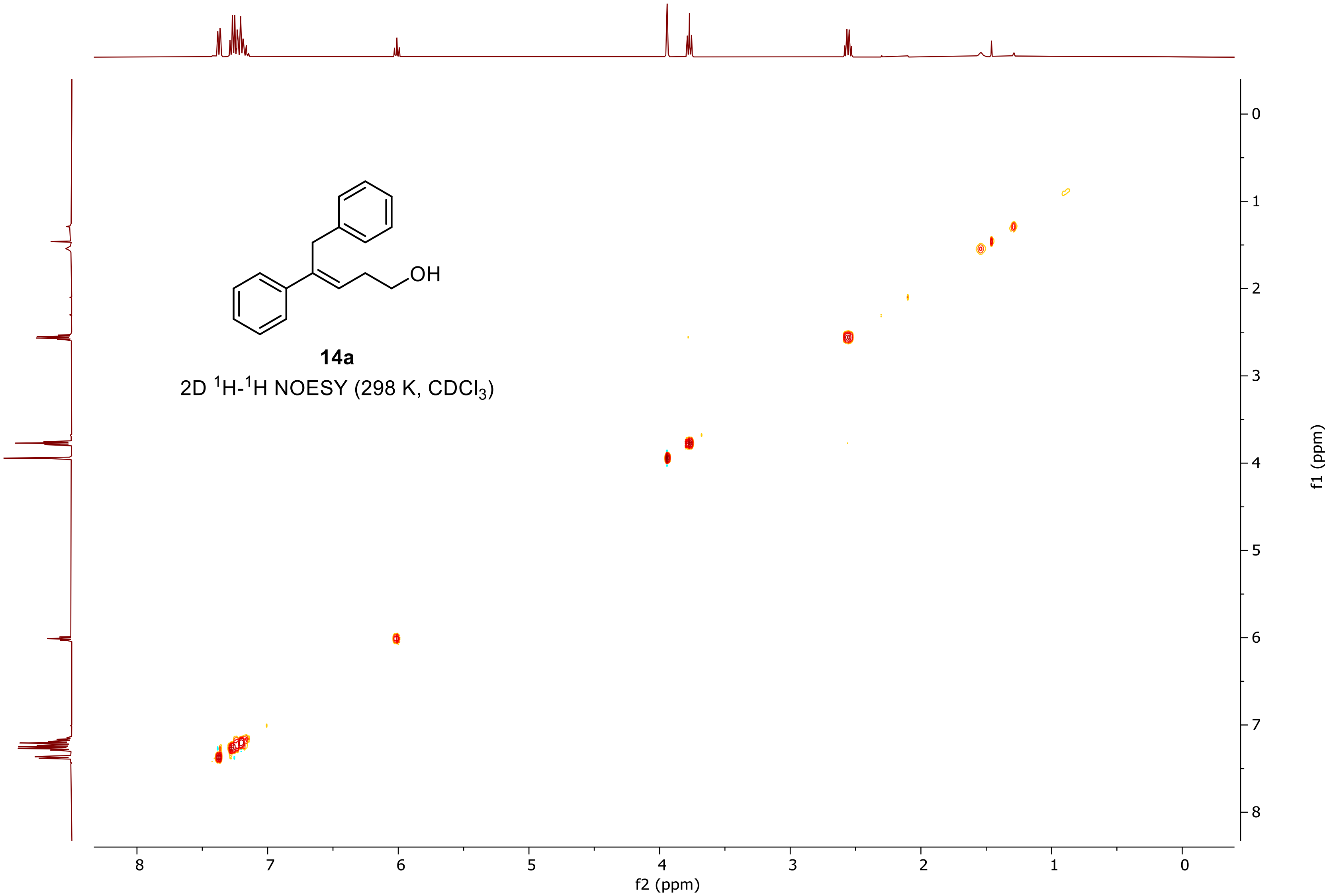
OH
14a
2D 1H-1H NOESY (298 K, CDCl3)
f2 (ppm)
f1 (ppm)

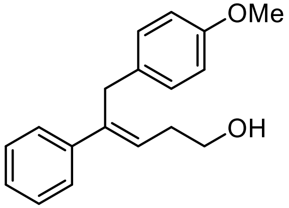

14b

^{1}H NMR (400 MHz, 298 K, $CDCl_3$)

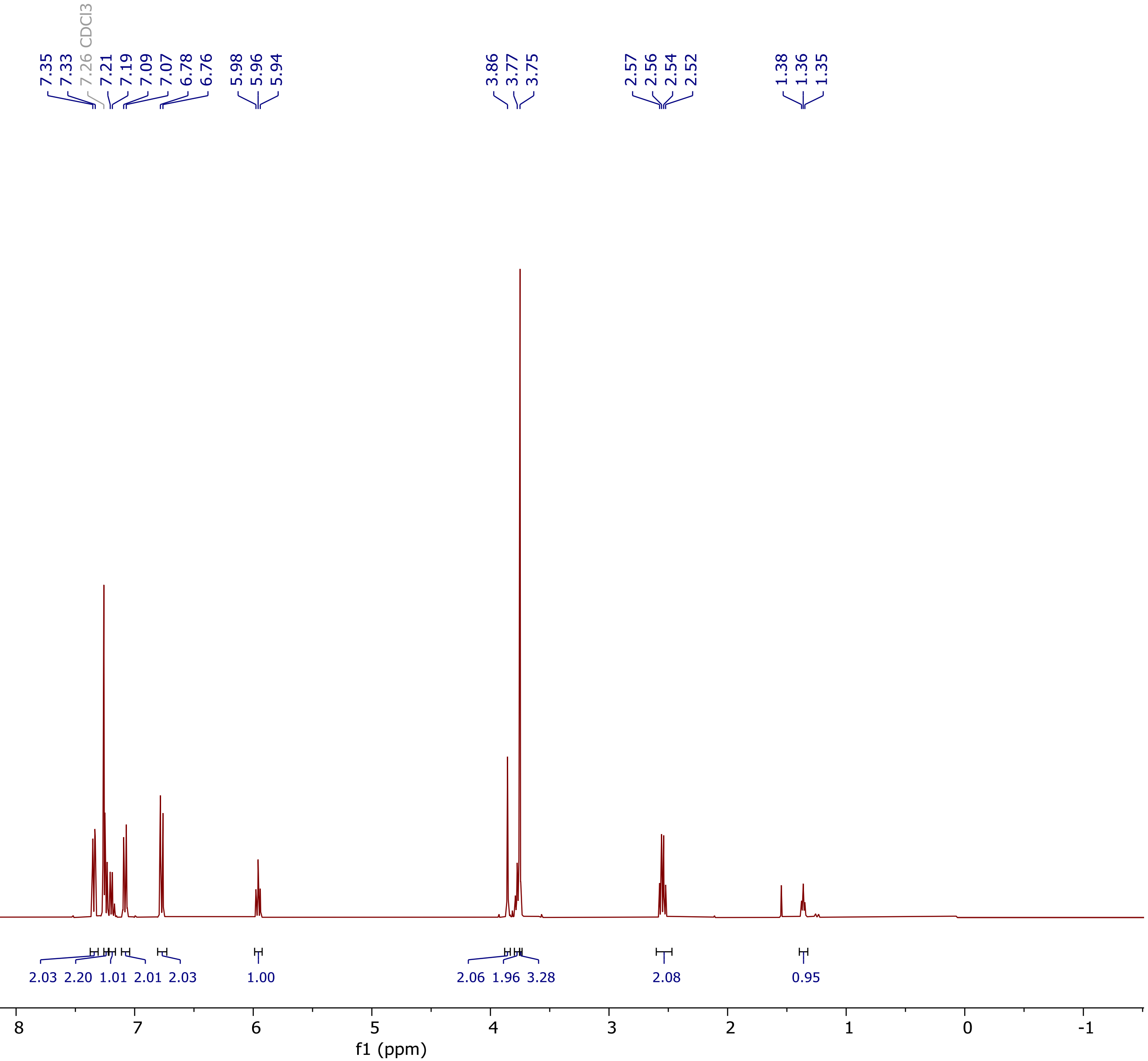

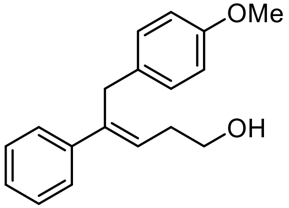

14b

$^{13}C\{^{1}H\}$ NMR (101 MHz, 298 K, $CDCl_3$)

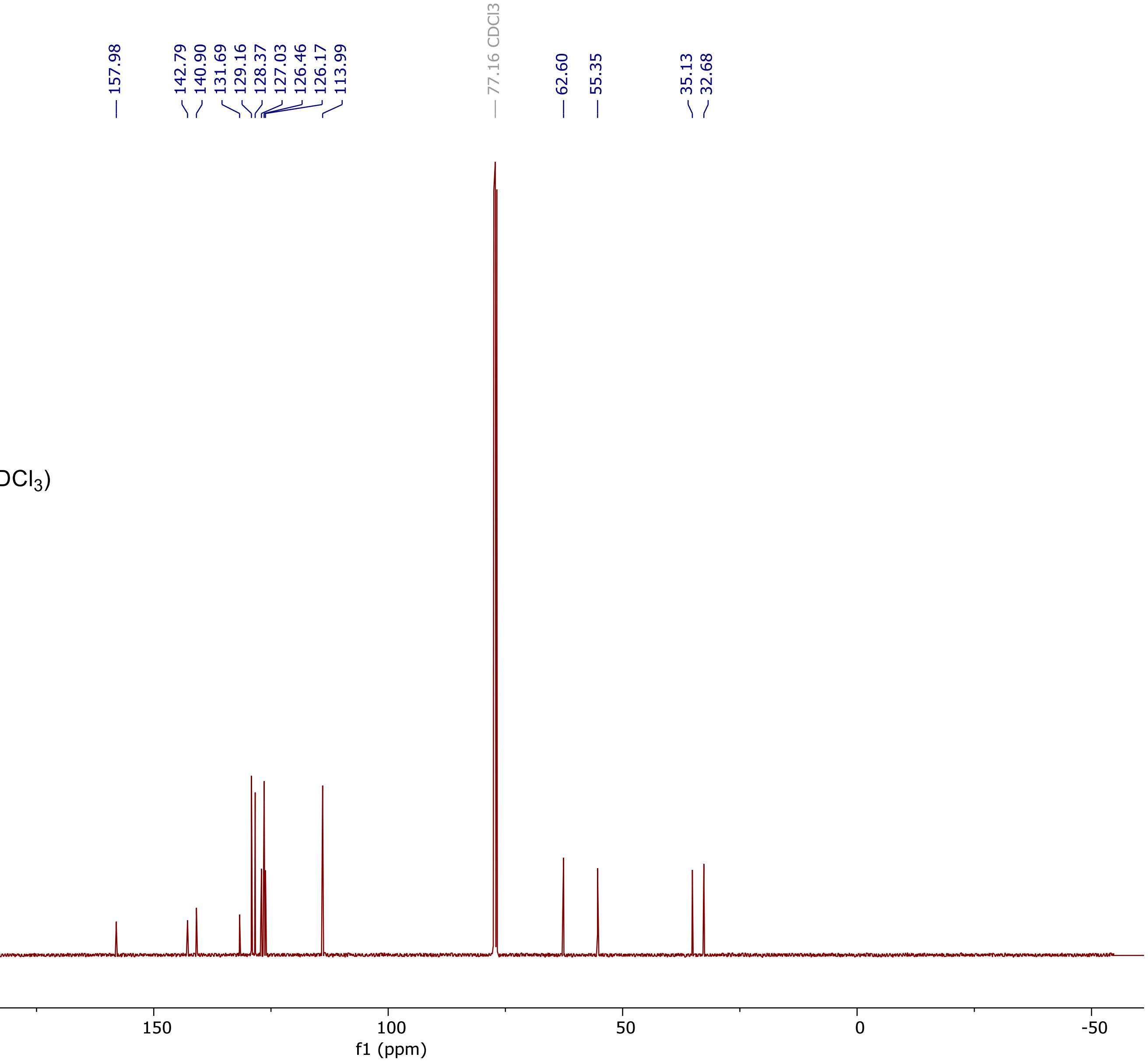

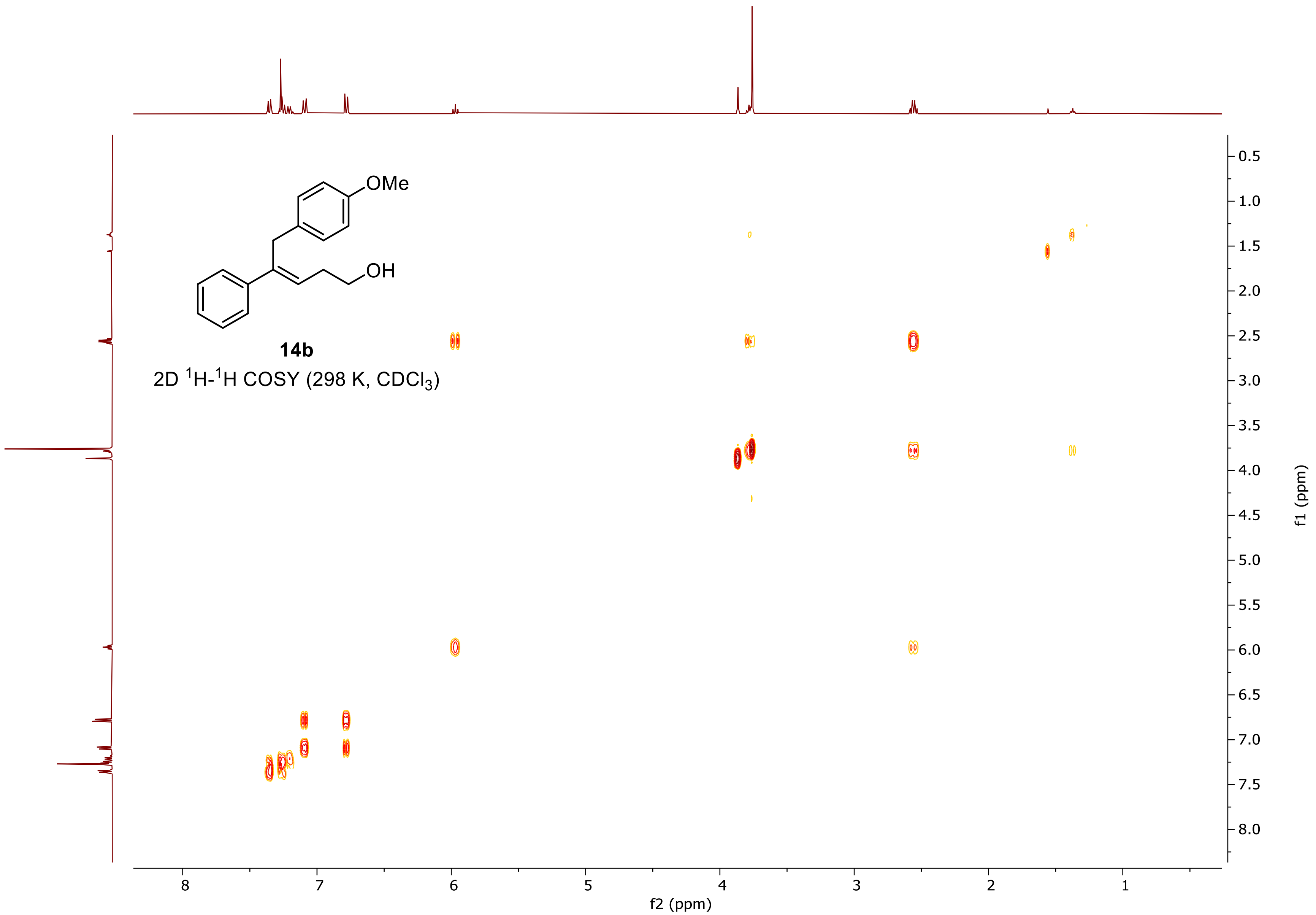

14b

2D ^{1}H-^{1}H COSY (298 K, $CDCl_3$)

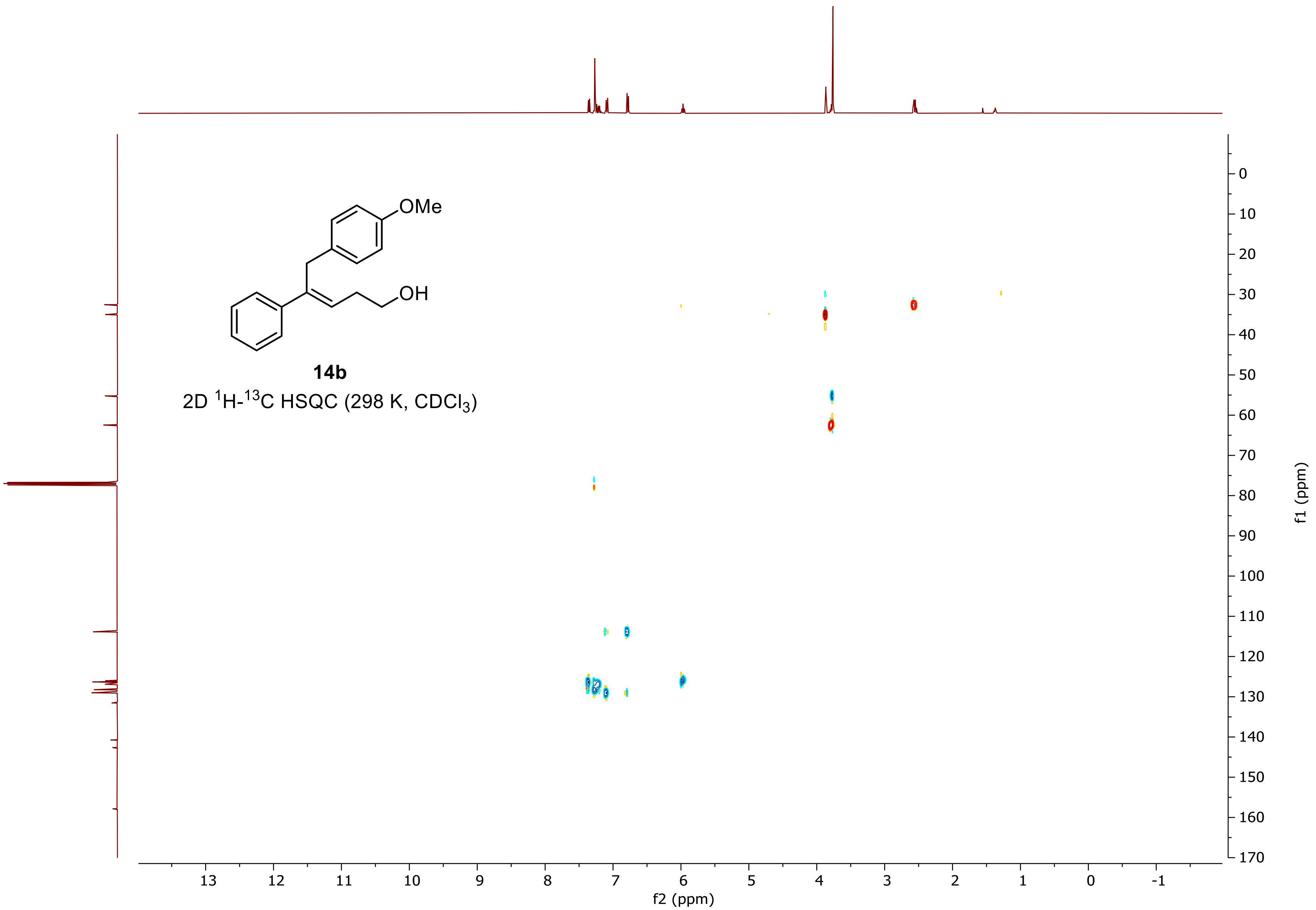
OMe
OH
14b
2D ^{1}H-^{13}C HSQC (298 K, $CDCl_3$)
f2 (ppm)
f1 (ppm)
13
12
11
10
9
8
7
6
5
4
3
2
1
0
-1
0
10
20
30
40
50
60
70
80
90
100
110
120
130
140
150
160
170

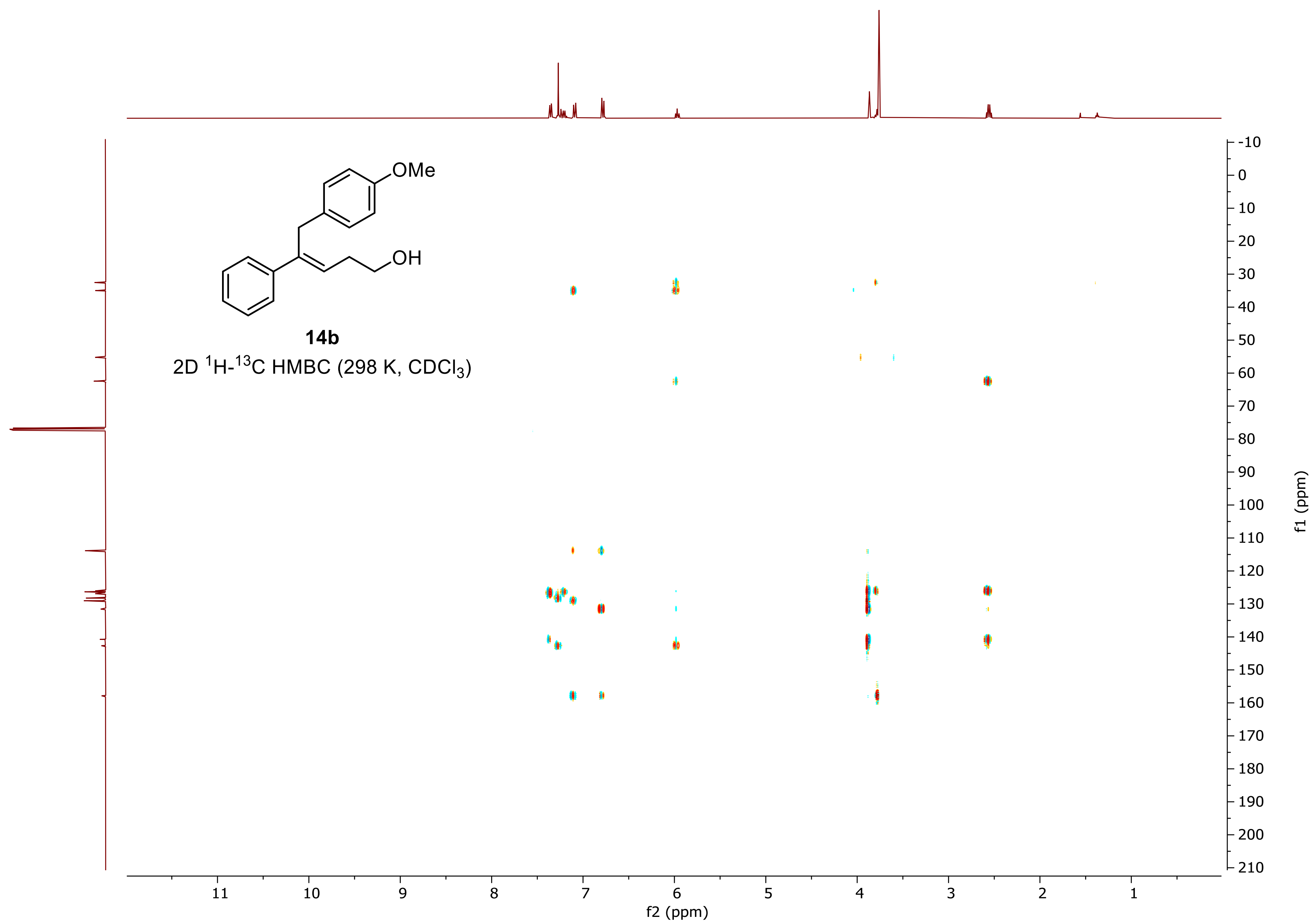
OMe
OH
14b
2D ^{1}H-^{13}C HMBC (298 K, $CDCl_3$)
f1 (ppm)
f2 (ppm)
11
10
9
8
7
6
5
4
3
2
1
-10
0
10
20
30
40
50
60
70
80
90
100
110
120
130
140
150
160
170
180
190
200
210

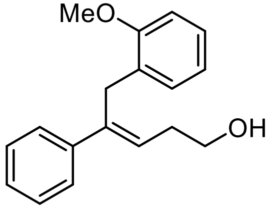

14c

^{1}H NMR (400 MHz, 298 K, $CDCl_3$)

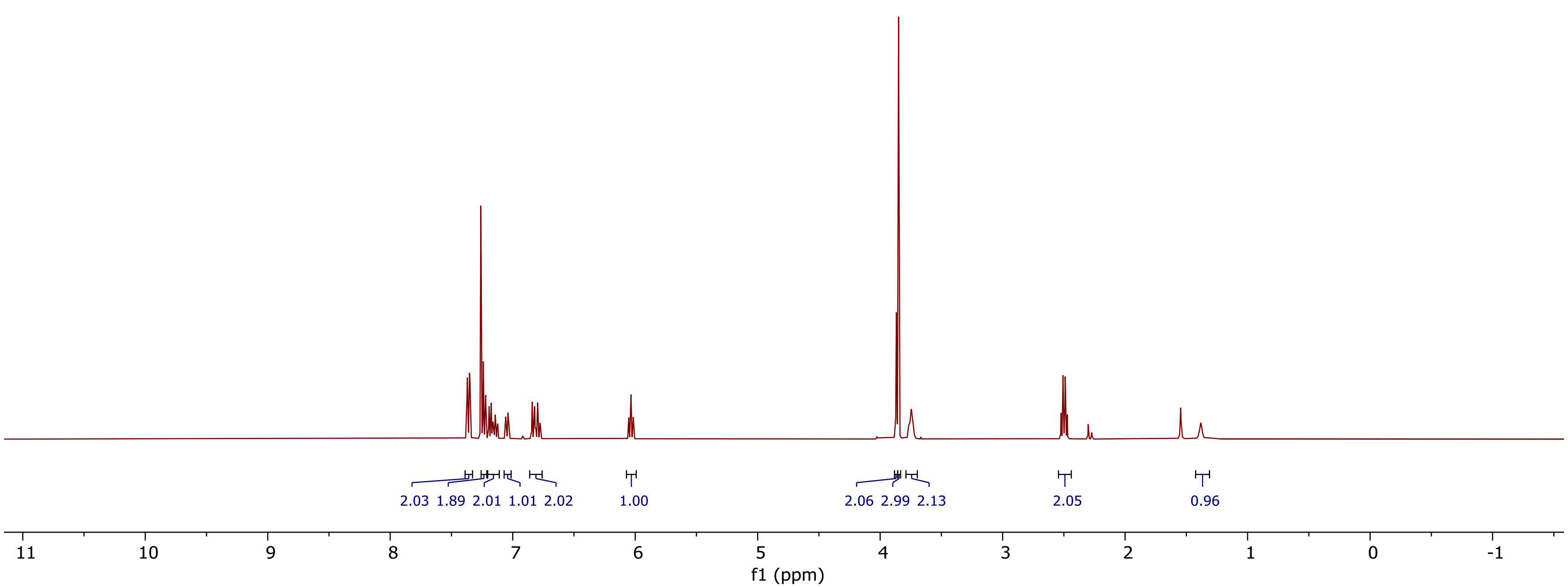

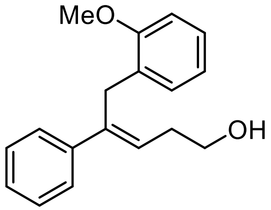

14c

$^{13}C\{^{1}H\}$ NMR (101 MHz, 298 K, $CDCl_3$)

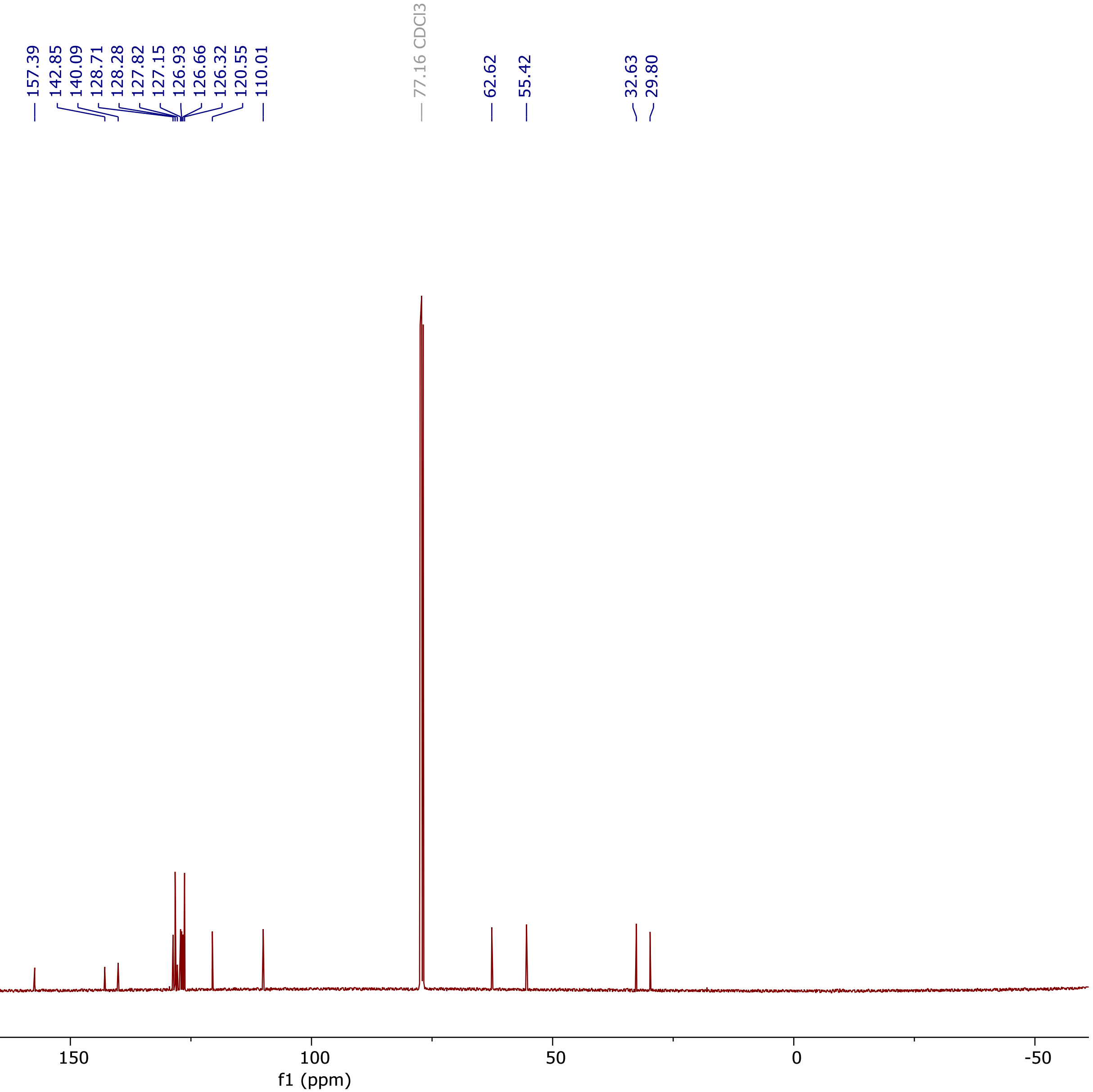

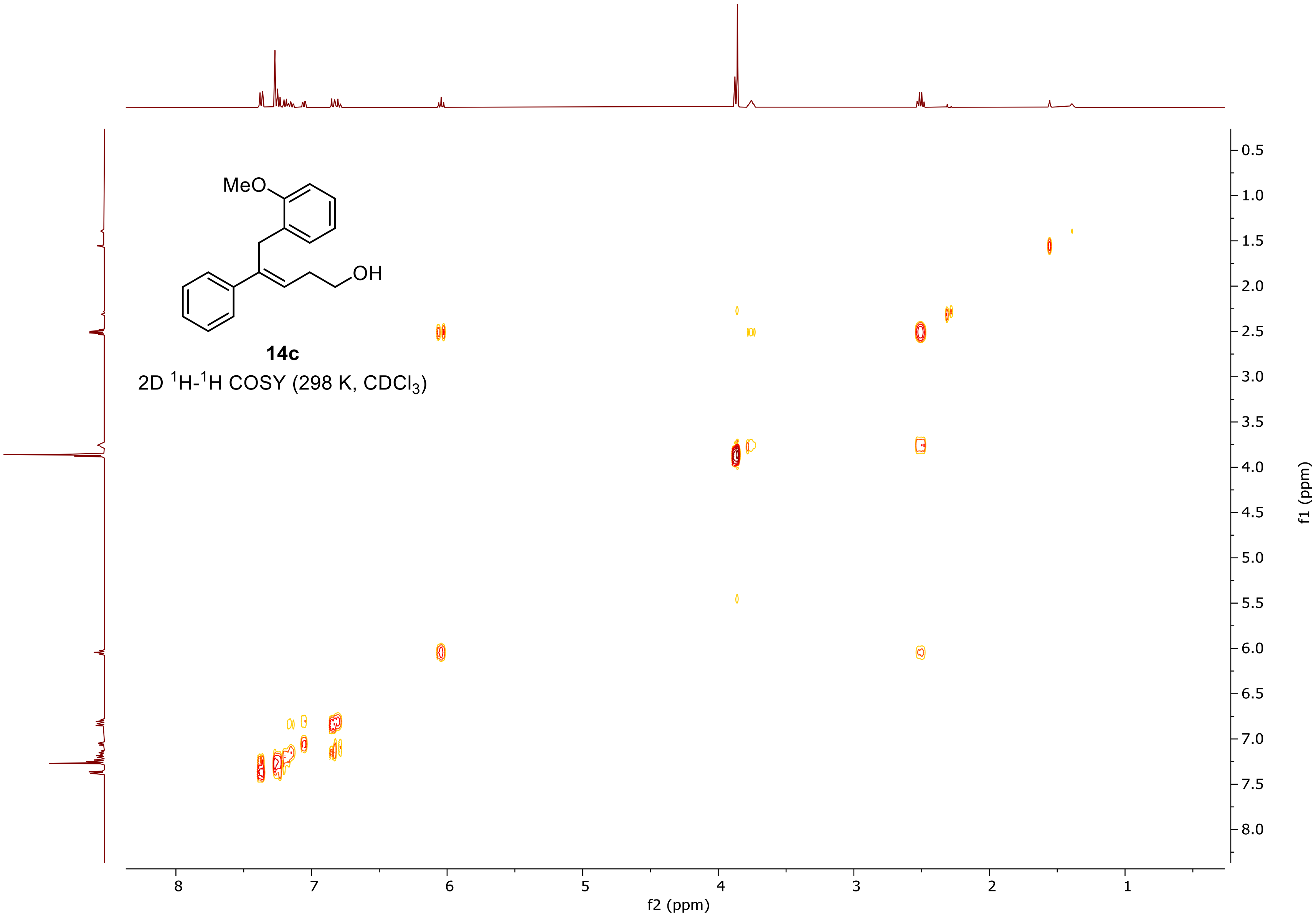

14c

2D ^{1}H-^{1}H COSY (298 K, $CDCl_3$)

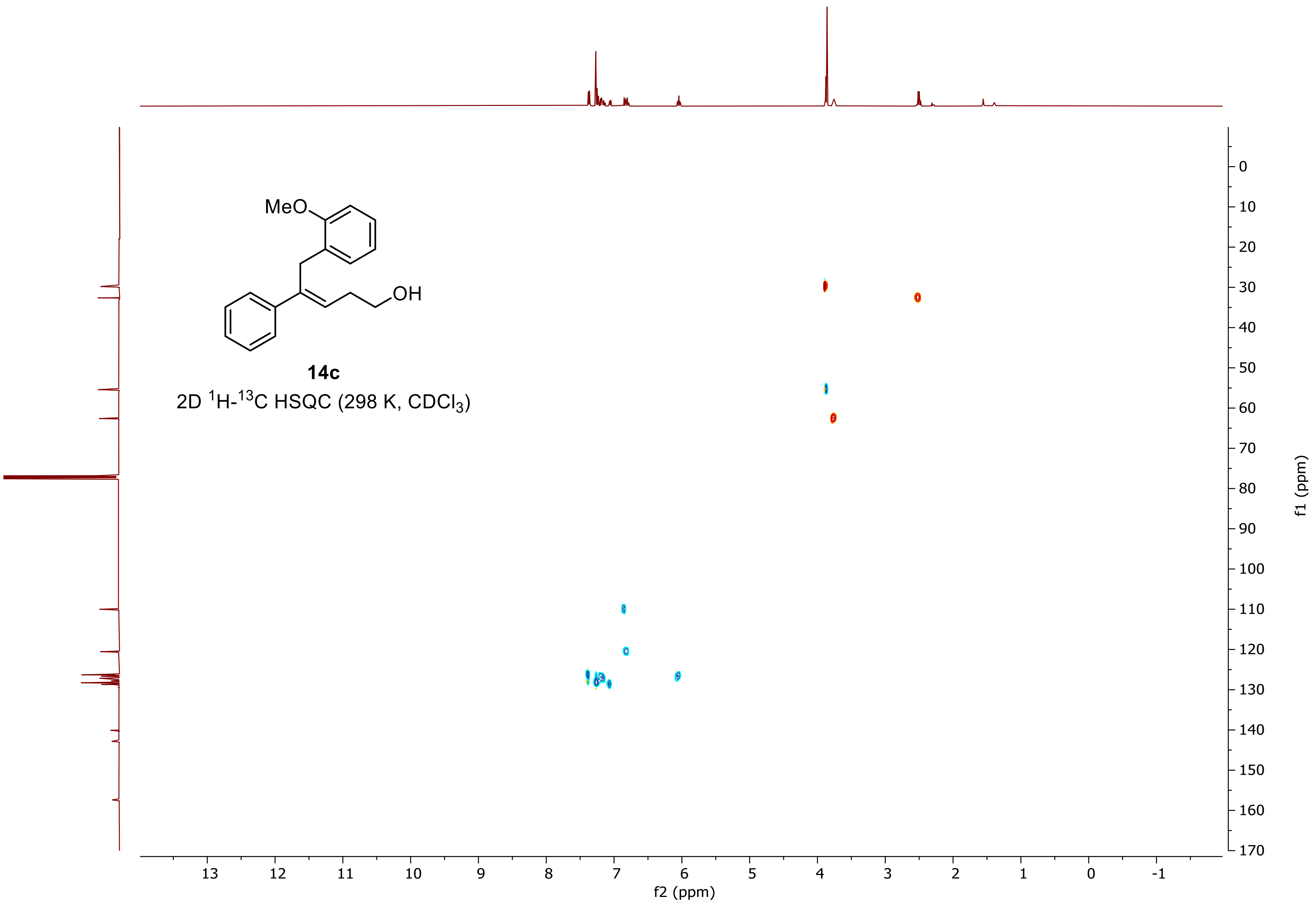
MeO
OH
14c
2D 1H-13C HSQC (298 K, CDCl3)
f1 (ppm)
f2 (ppm)

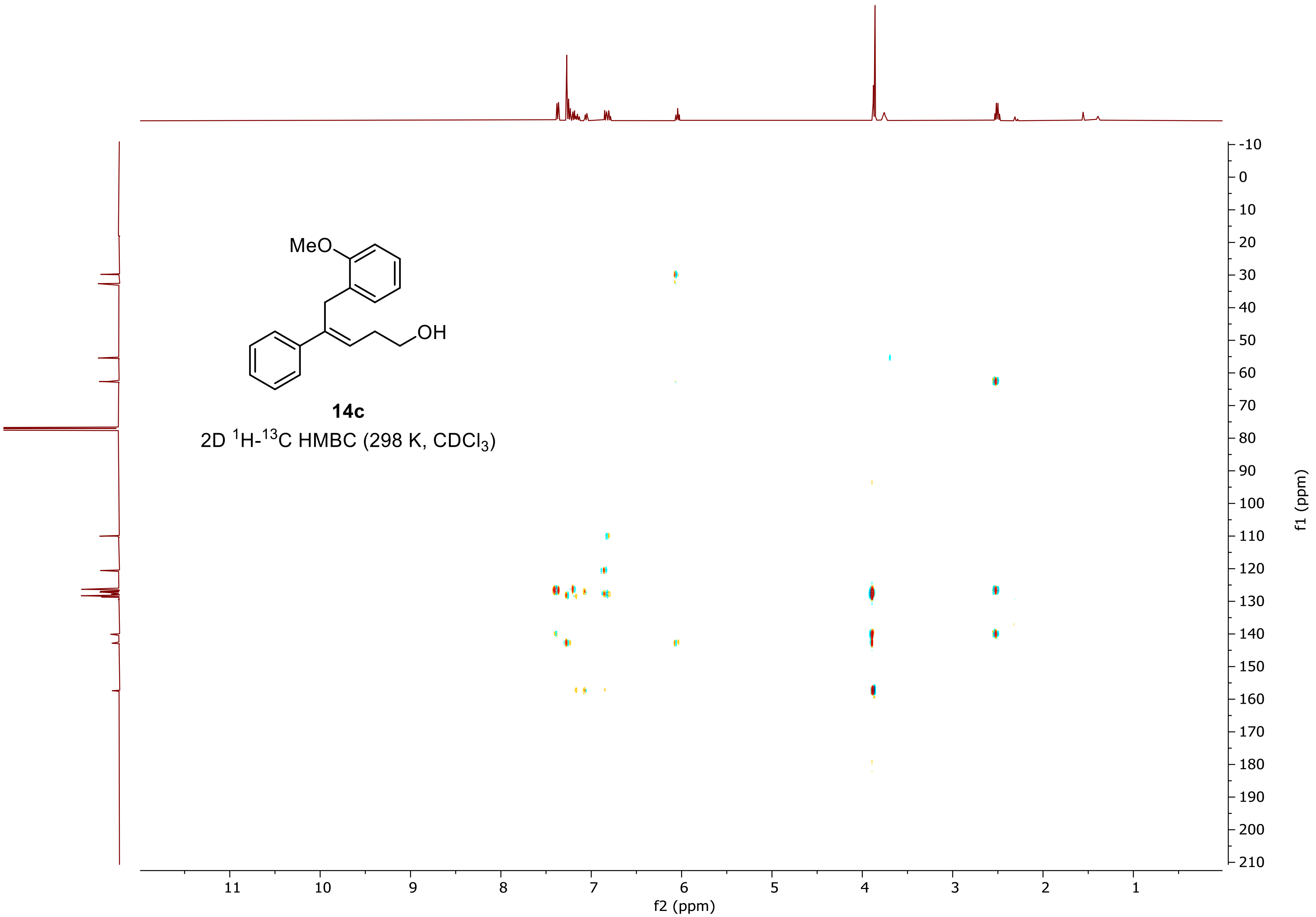

14c

2D ^{1}H-^{13}C HMBC (298 K, $CDCl_3$)

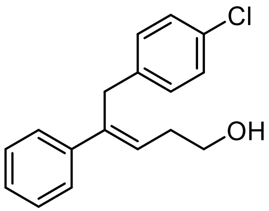

14d

^{1}H NMR (400 MHz, 298 K, $CDCl_3$)

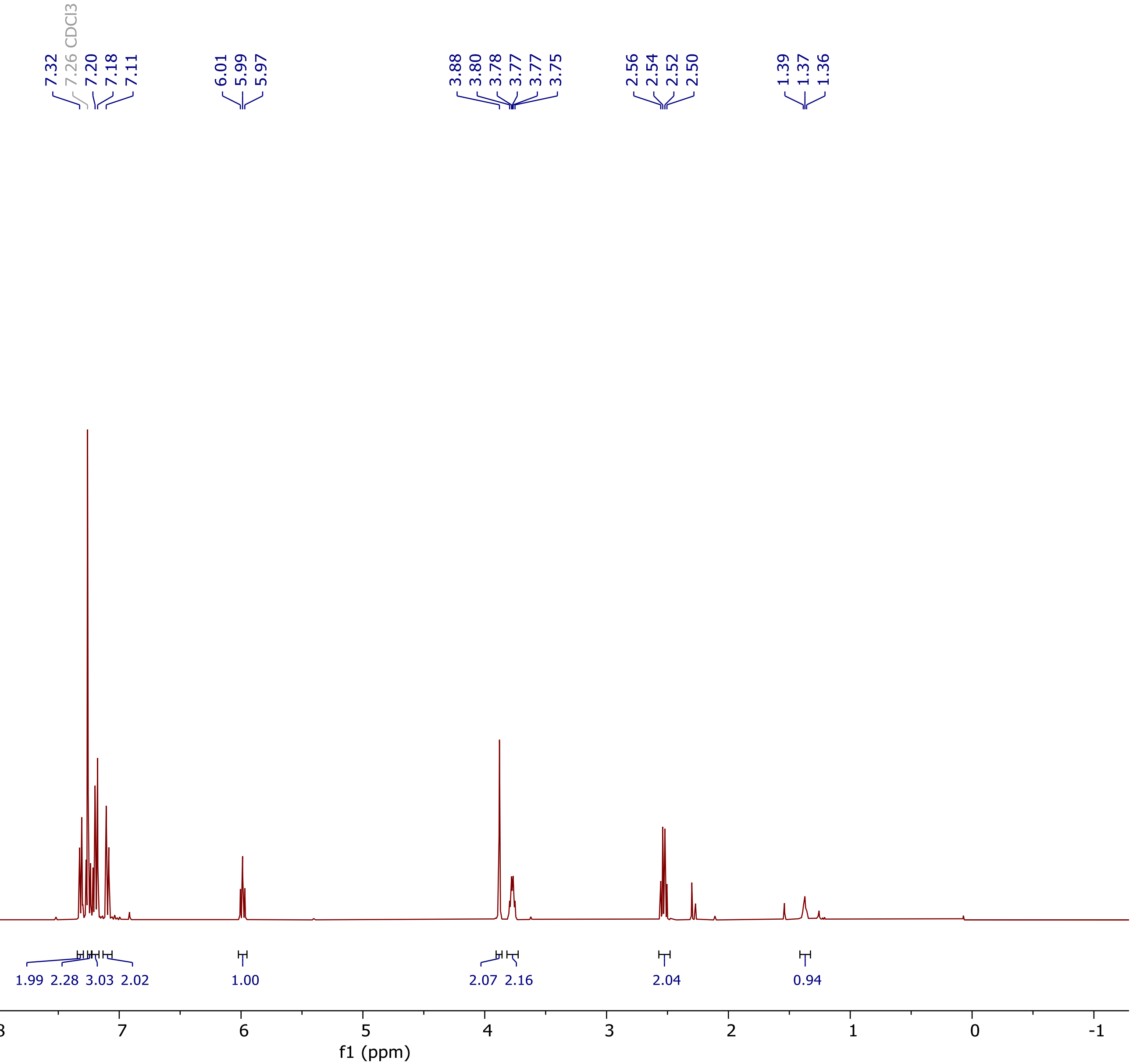

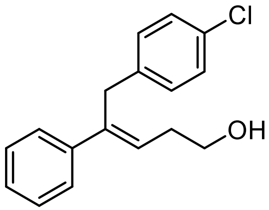

14d

$^{13}C\{^{1}H\}$ NMR (101 MHz, 298 K, $CDCl_3$)

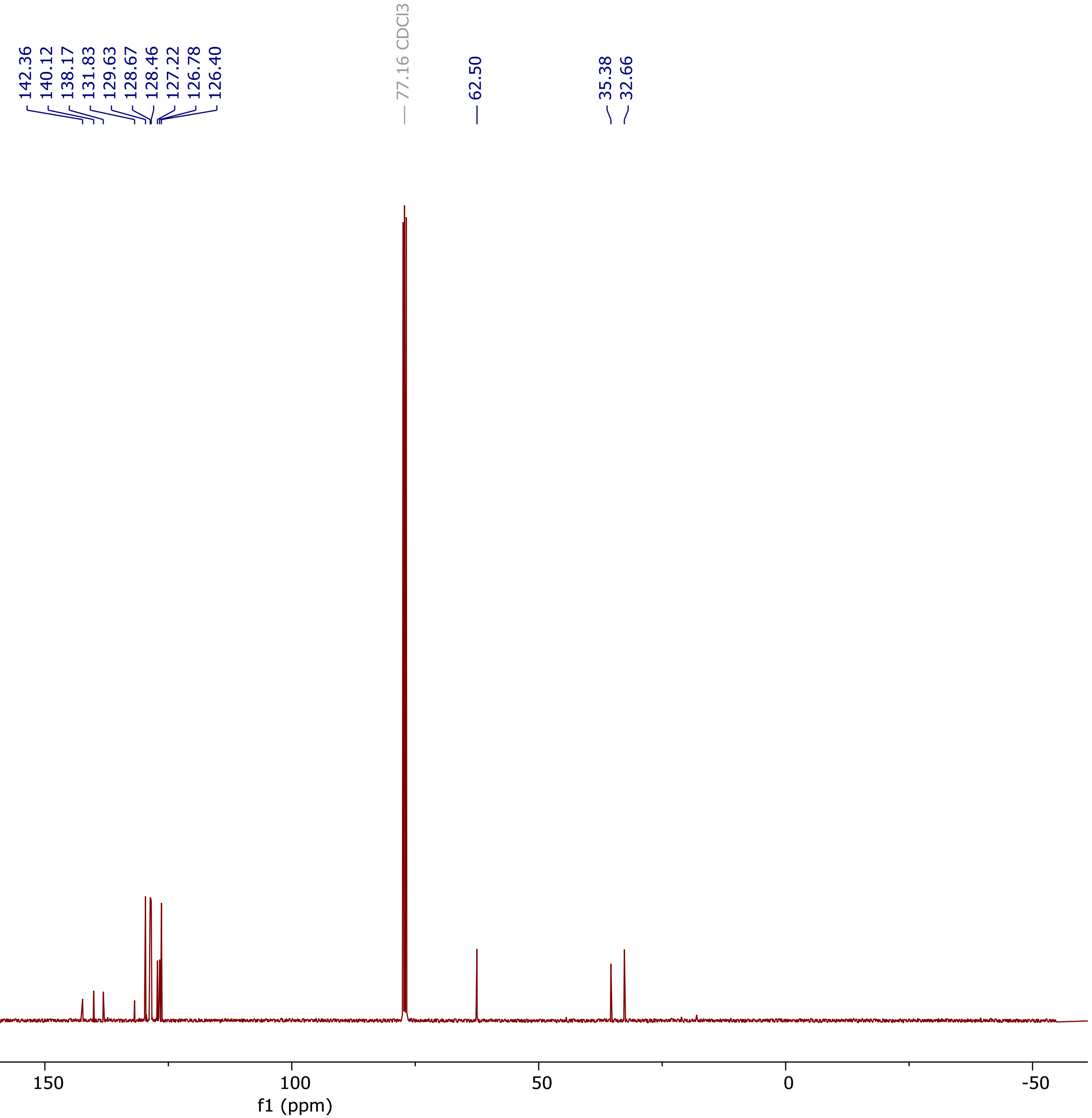

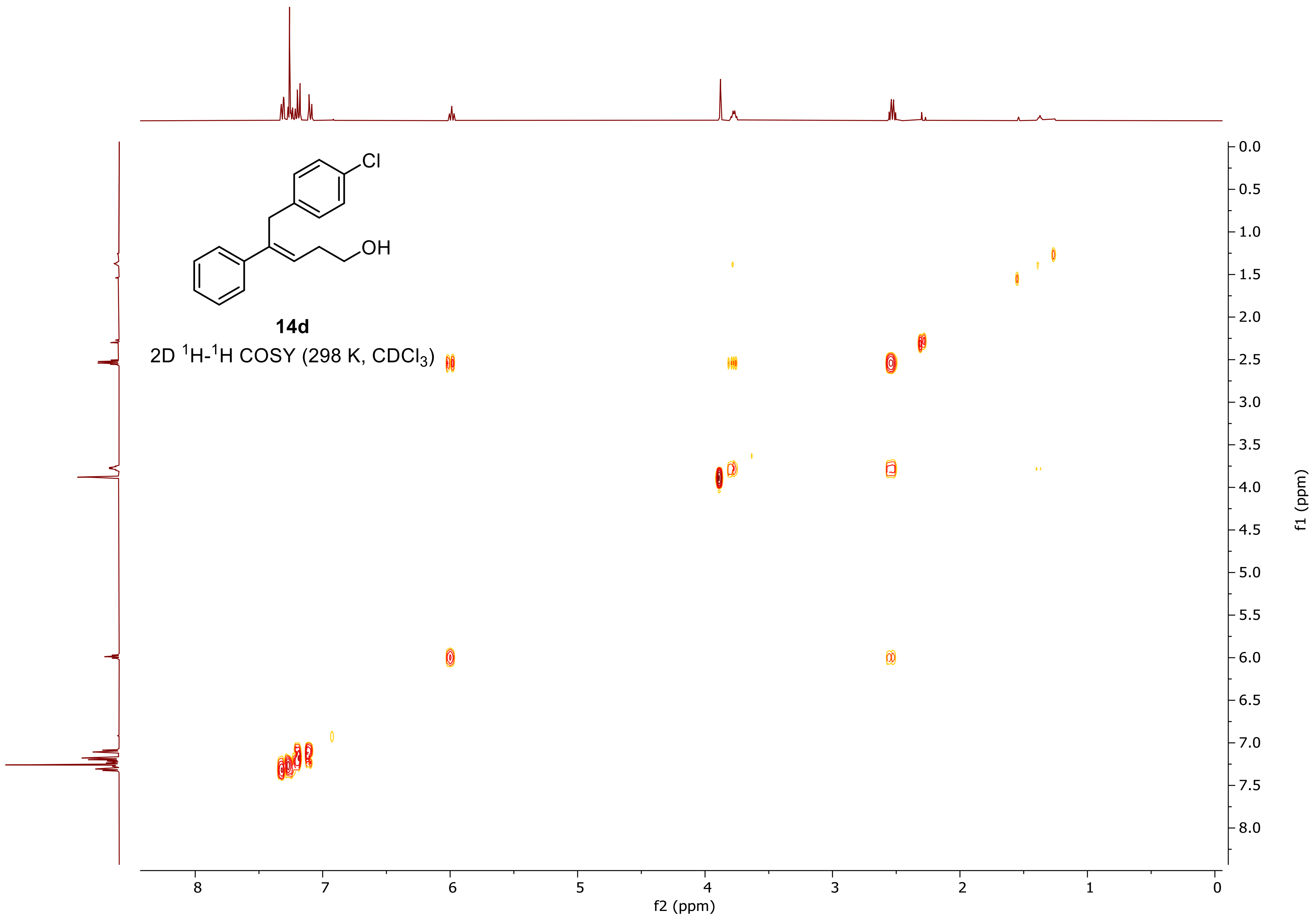

14d

2D ^{1}H-^{1}H COSY (298 K, $CDCl_3$)

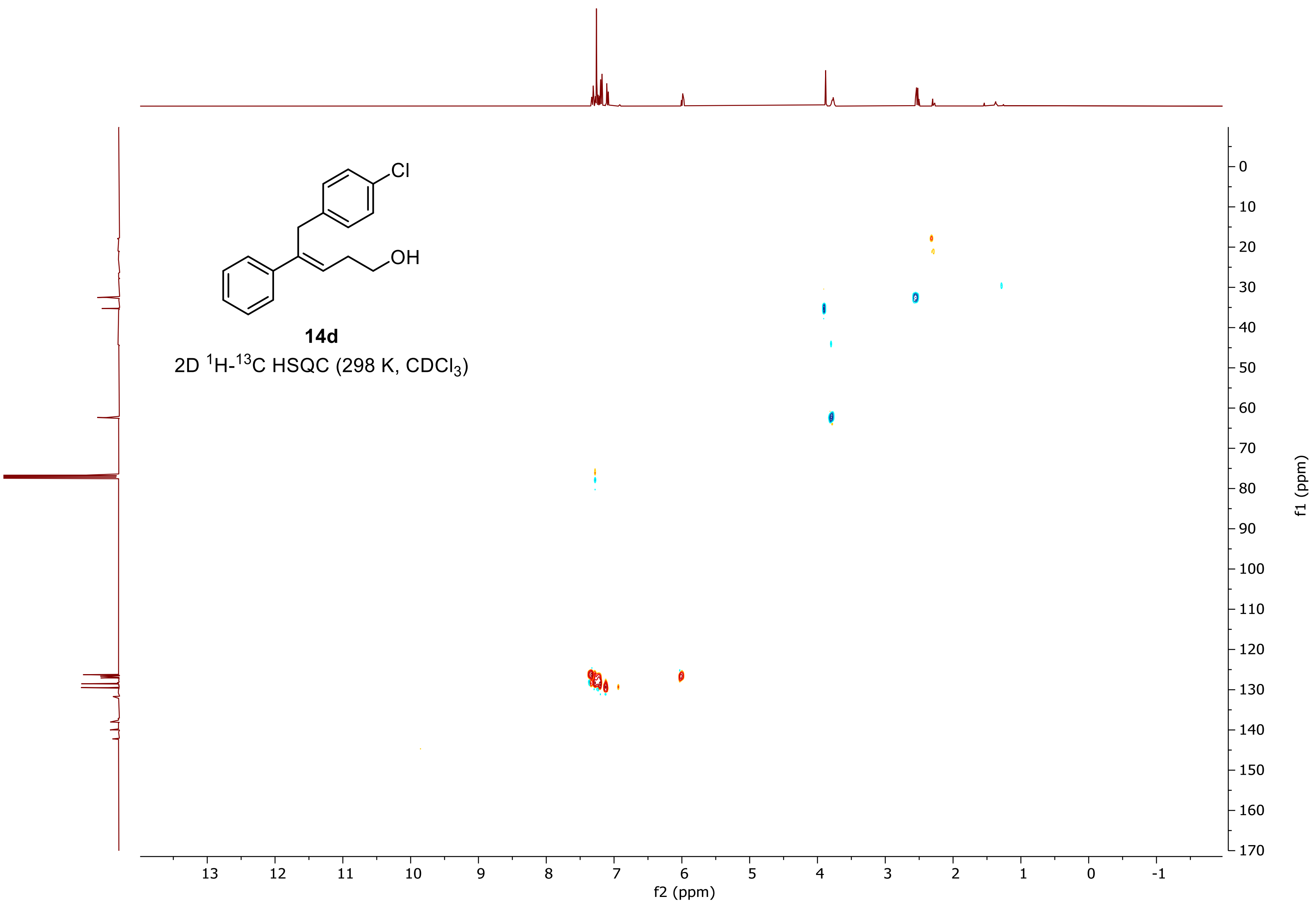
Cl
OH
14d
2D 1H-13C HSQC (298 K, CDCl3)
f2 (ppm)
f1 (ppm)

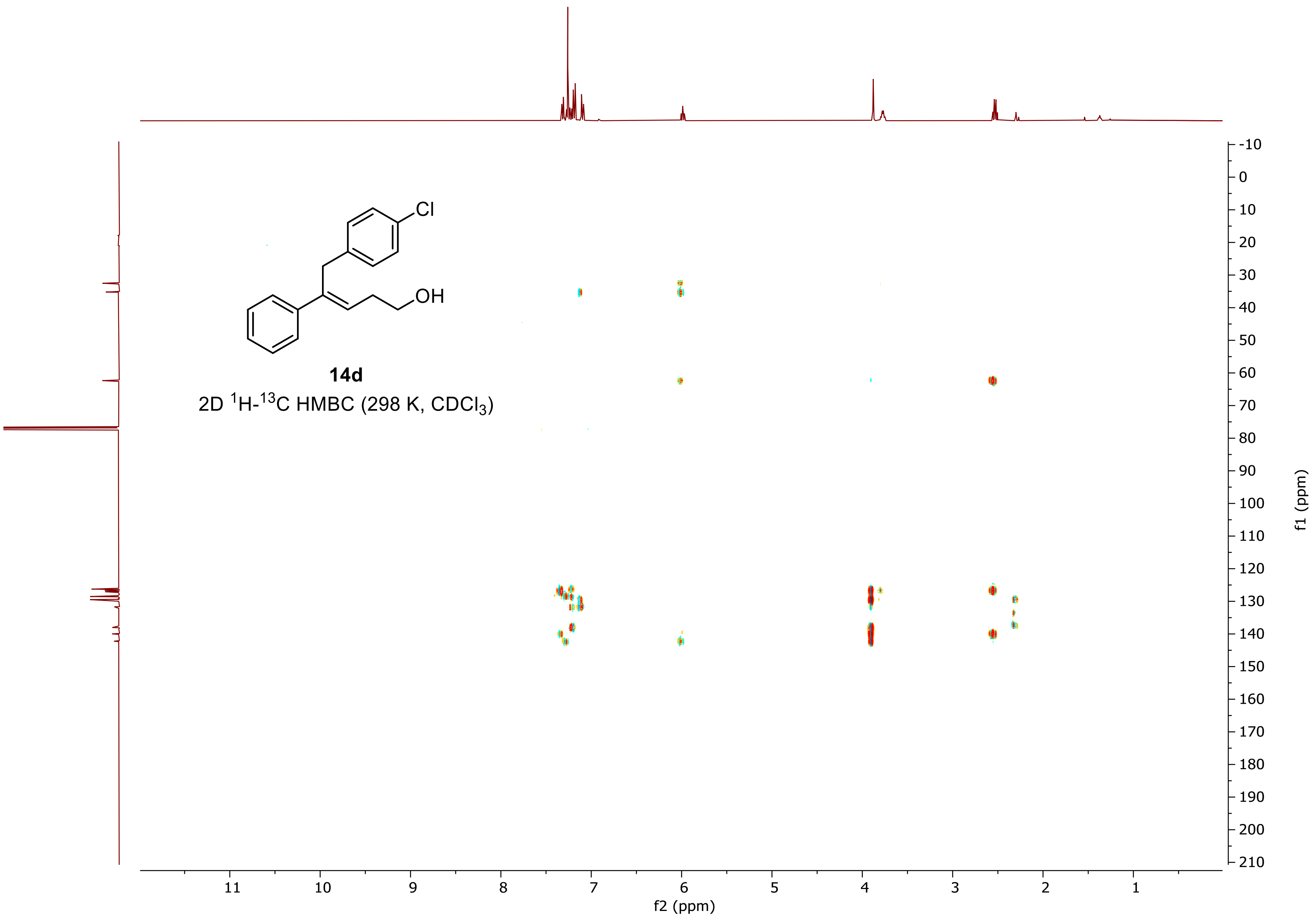
Cl
OH
14d
2D ^{1}H-^{13}C HMBC (298 K, $CDCl_3$)
f1 (ppm)
f2 (ppm)

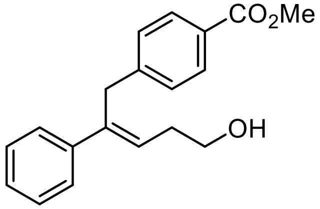

14e

^{1}H NMR (400 MHz, 298 K, $CDCl_3$)

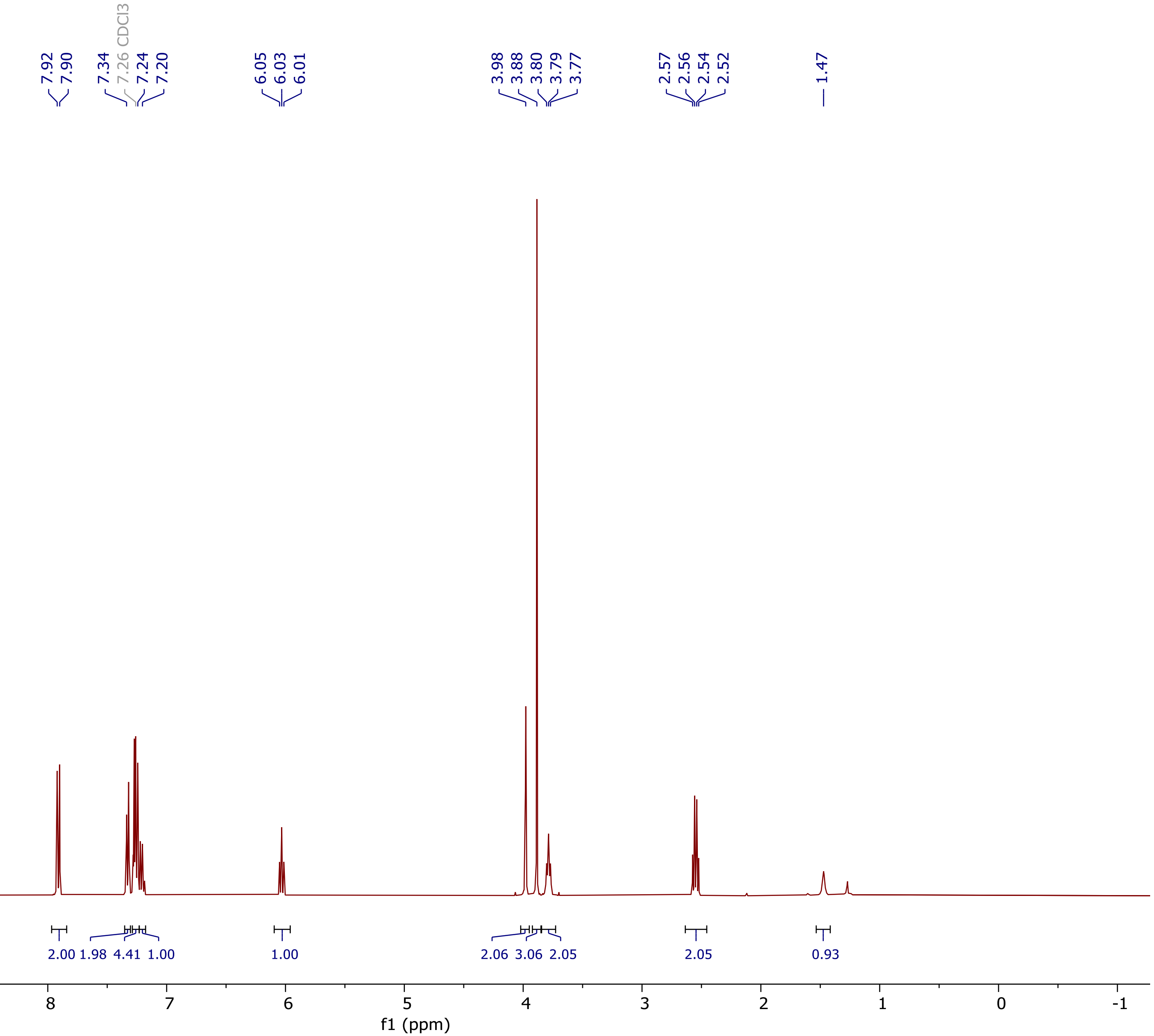

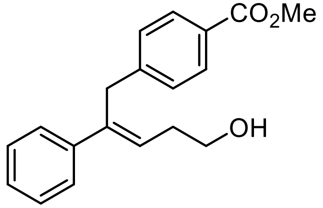

14e

$^{13}C\{^{1}H\}$ NMR (101 MHz, 298 K, $CDCl_3$)

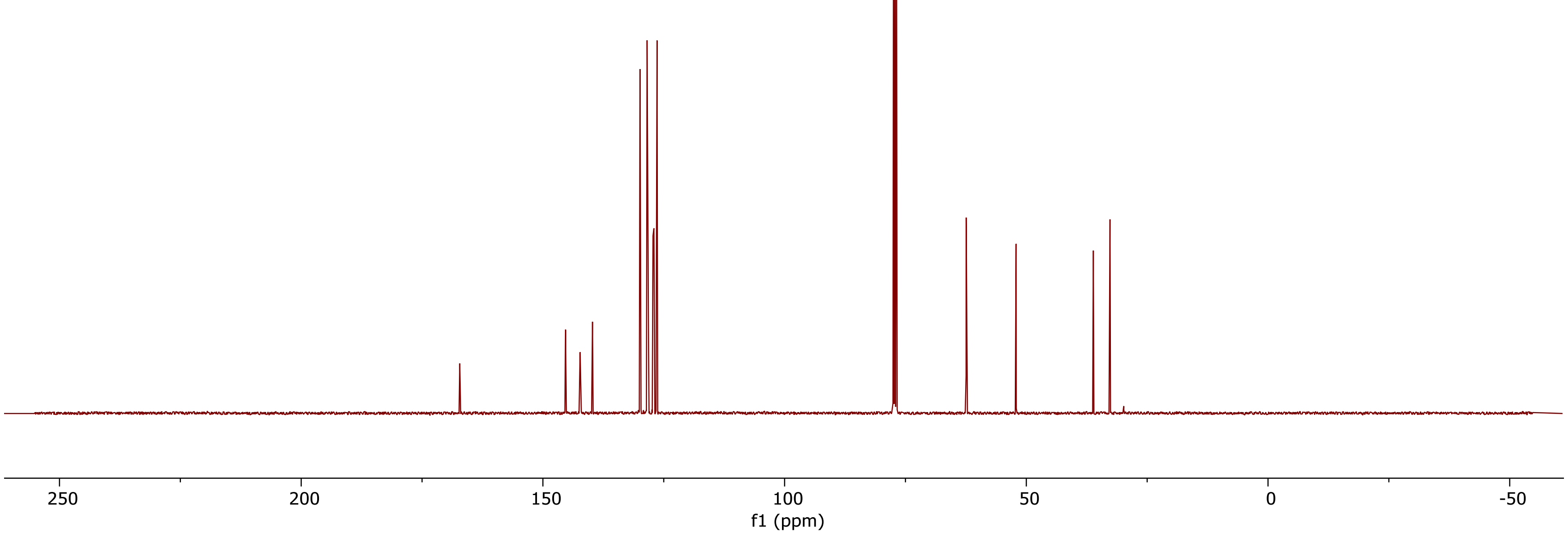

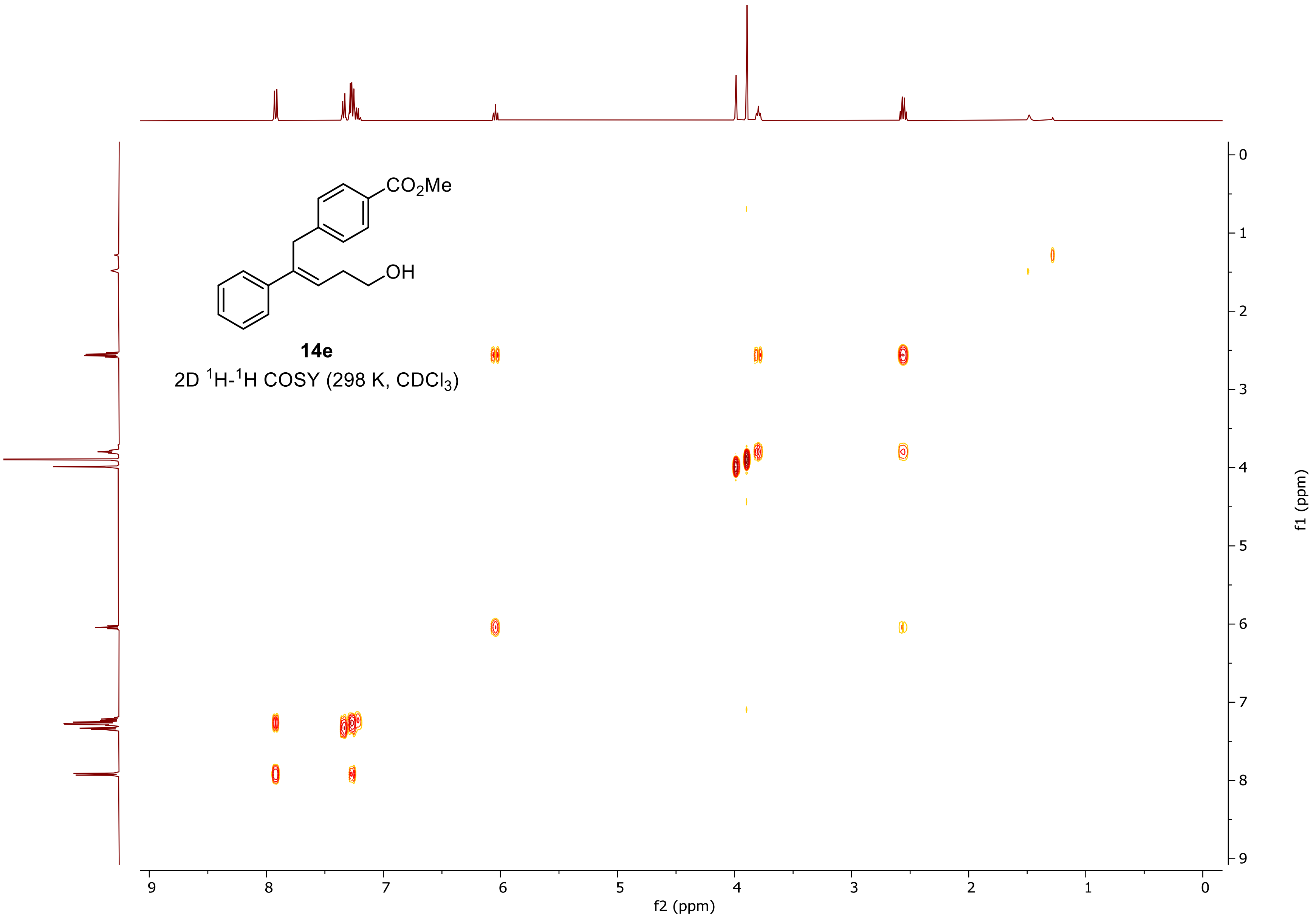

CO2Me
OH
14e
2D 1H-1H COSY (298 K, CDCl3)
f2 (ppm)
f1 (ppm)

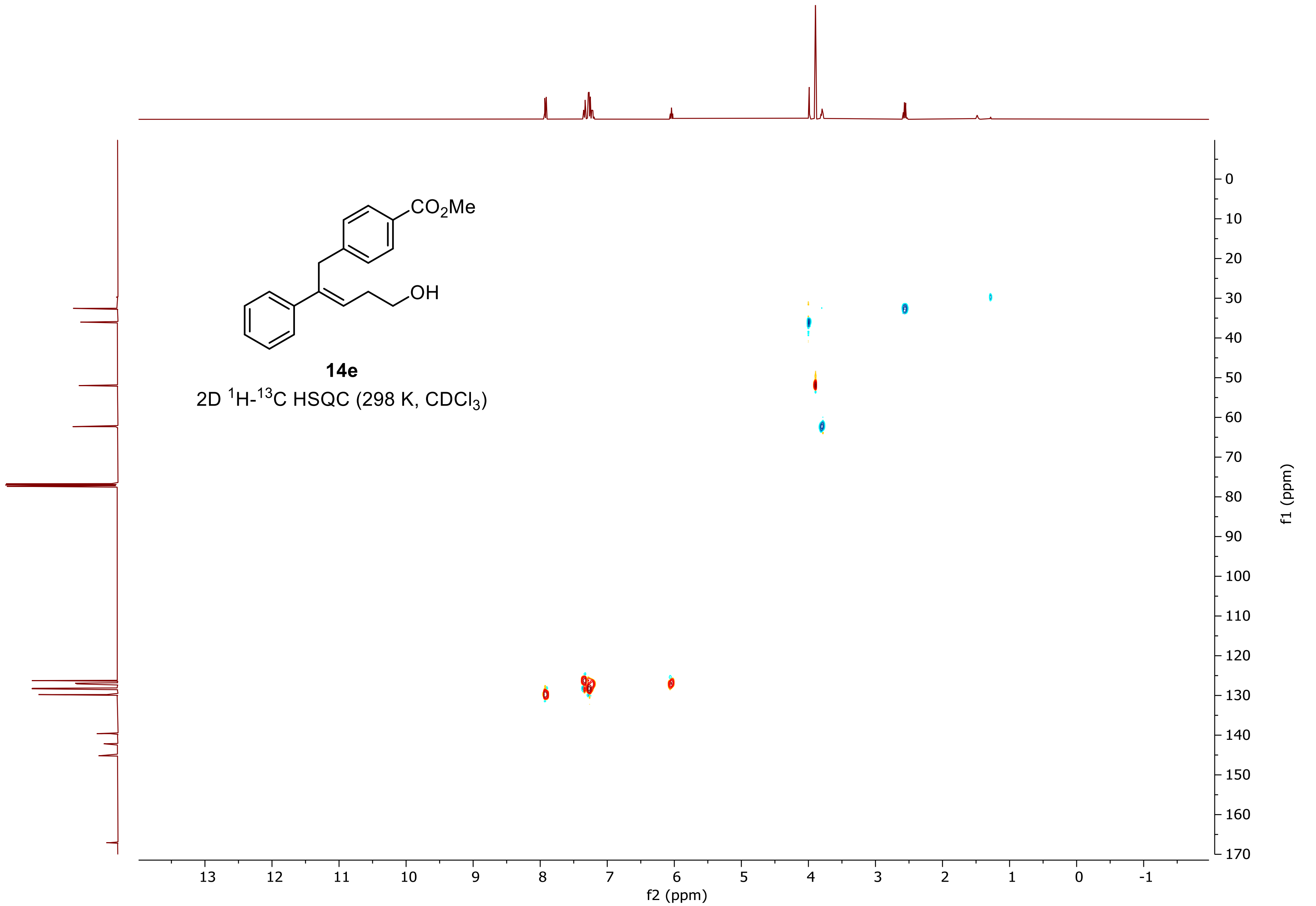

CO2Me
OH
14e
2D 1H-13C HSQC (298 K, CDCl3)
f2 (ppm)
f1 (ppm)

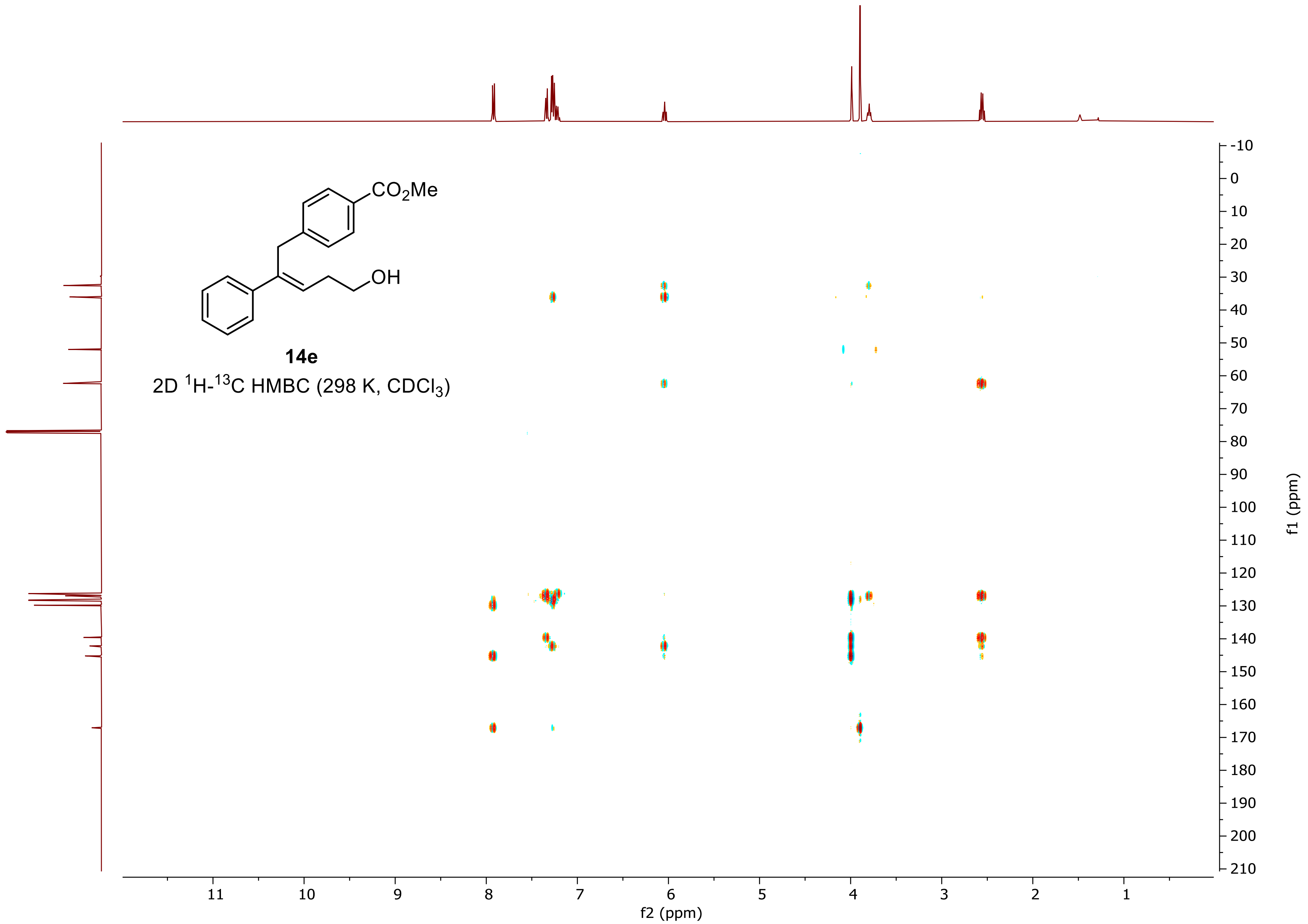
CO2Me
OH
14e
2D 1H-13C HMBC (298 K, CDCl3)
f2 (ppm)
f1 (ppm)

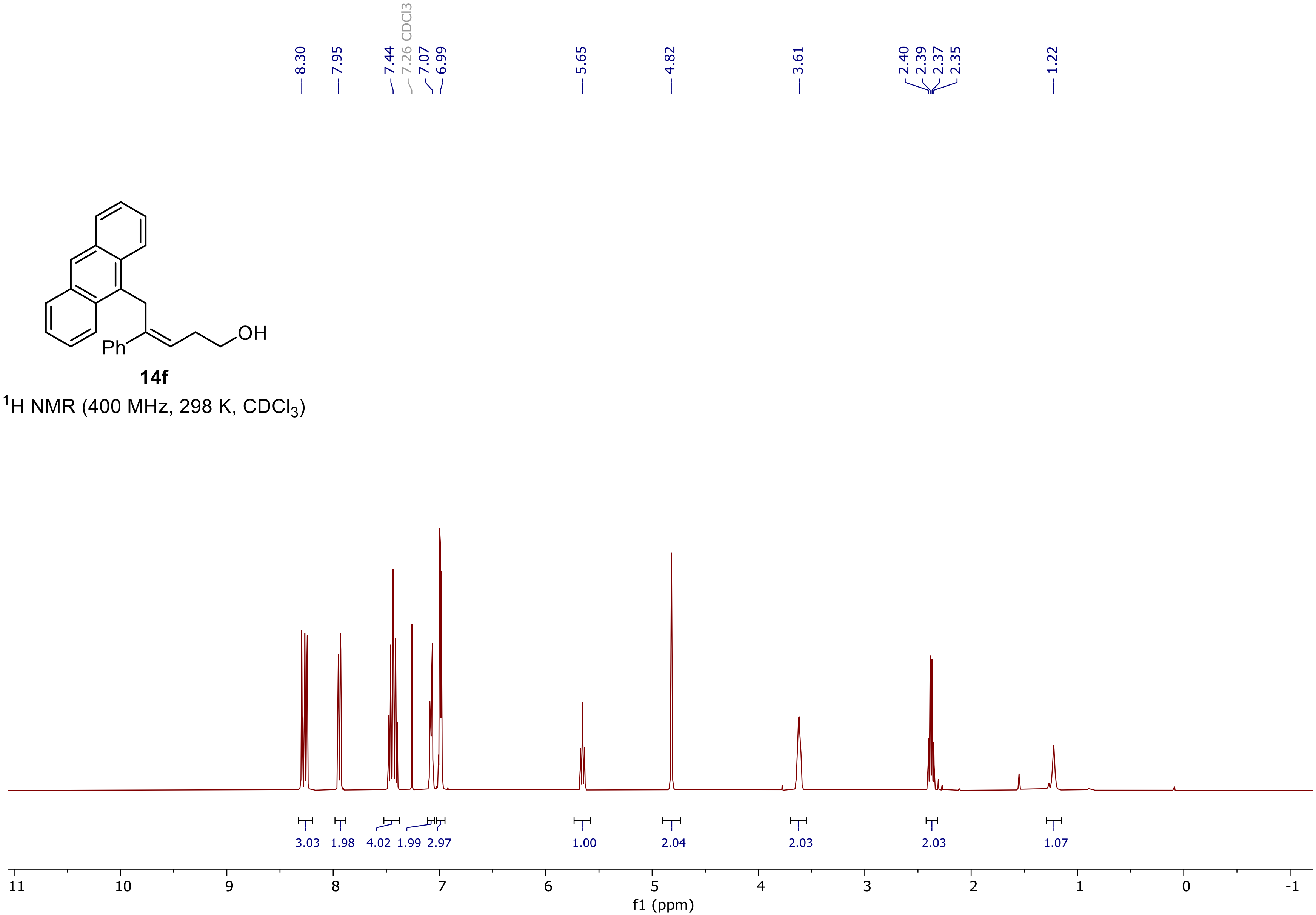

14f

^{1}H NMR (400 MHz, 298 K, $CDCl_3$)

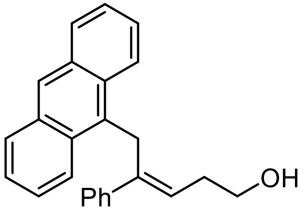

14f

$^{13}C\{^{1}H\}$ NMR (101 MHz, 298 K, $CDCl_3$)

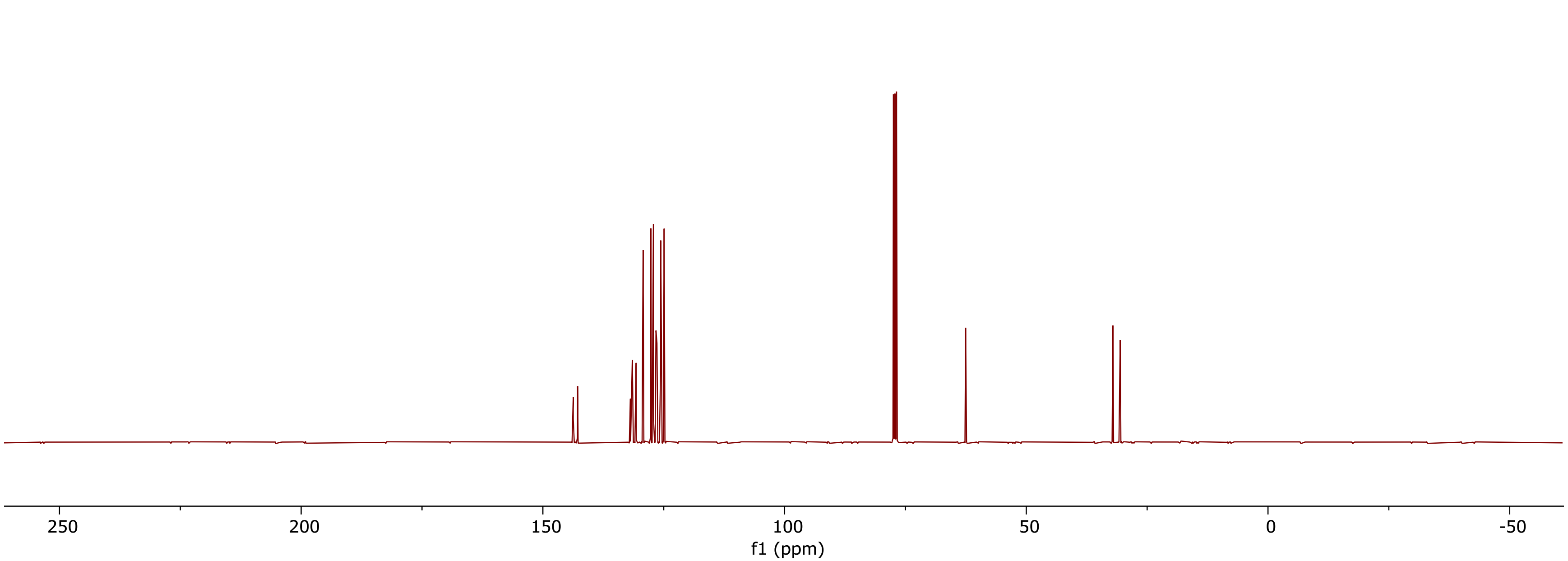

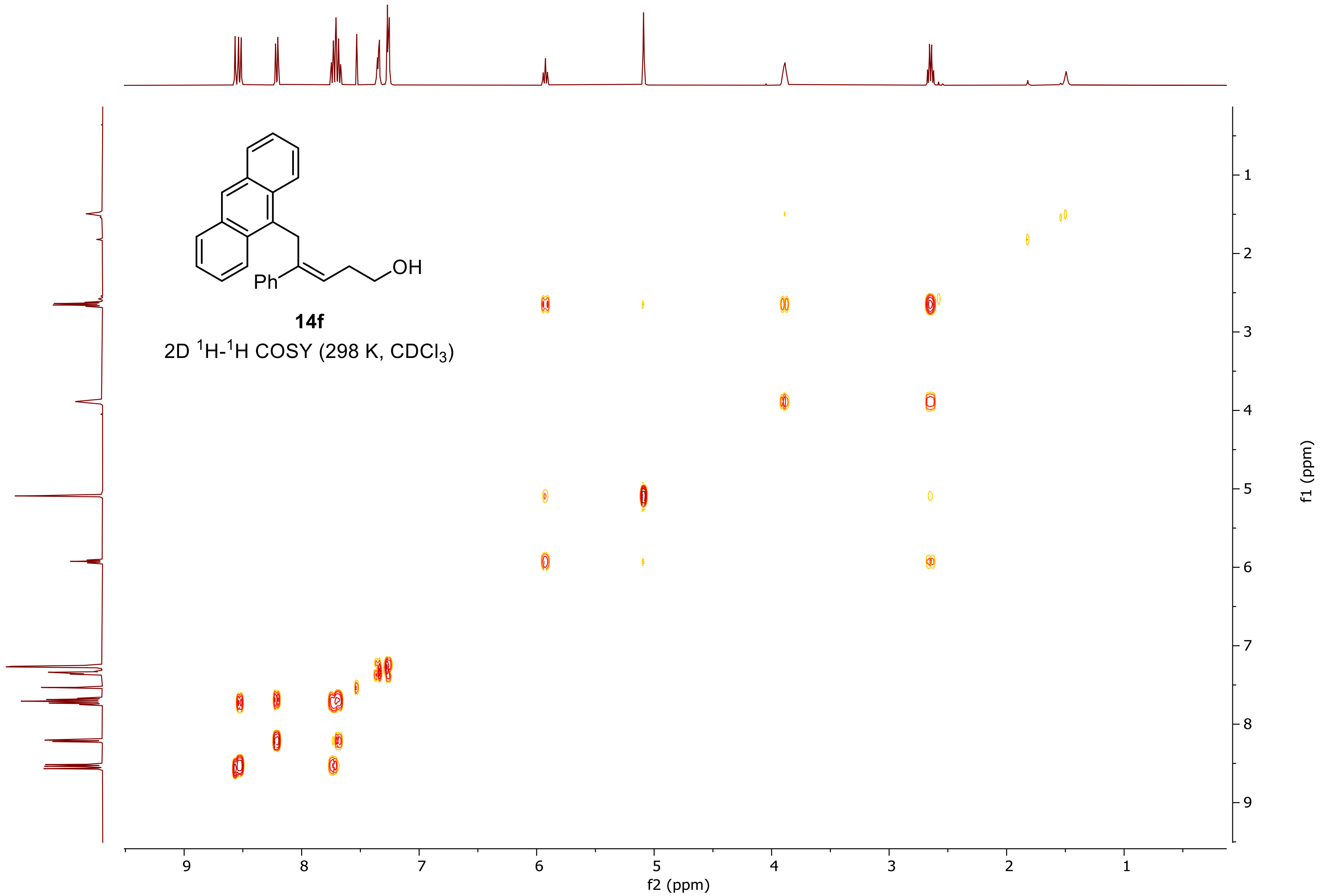

OH
Ph
14f
2D ¹H-¹H COSY (298 K, CDCl3)
f2 (ppm)
f1 (ppm)

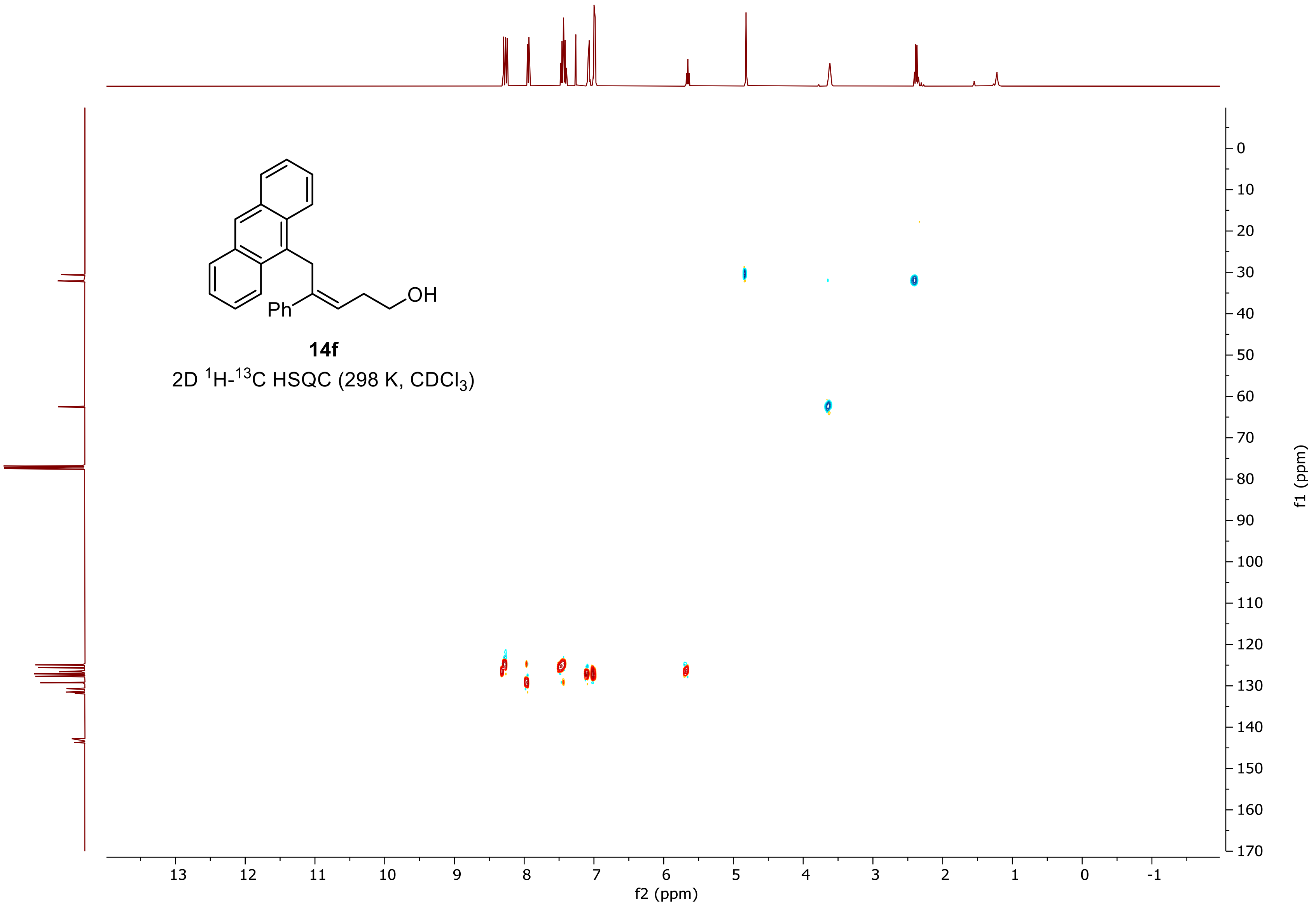
Ph
OH
14f
2D ^{1}H-^{13}C HSQC (298 K, $CDCl_3$)
f2 (ppm)
f1 (ppm)

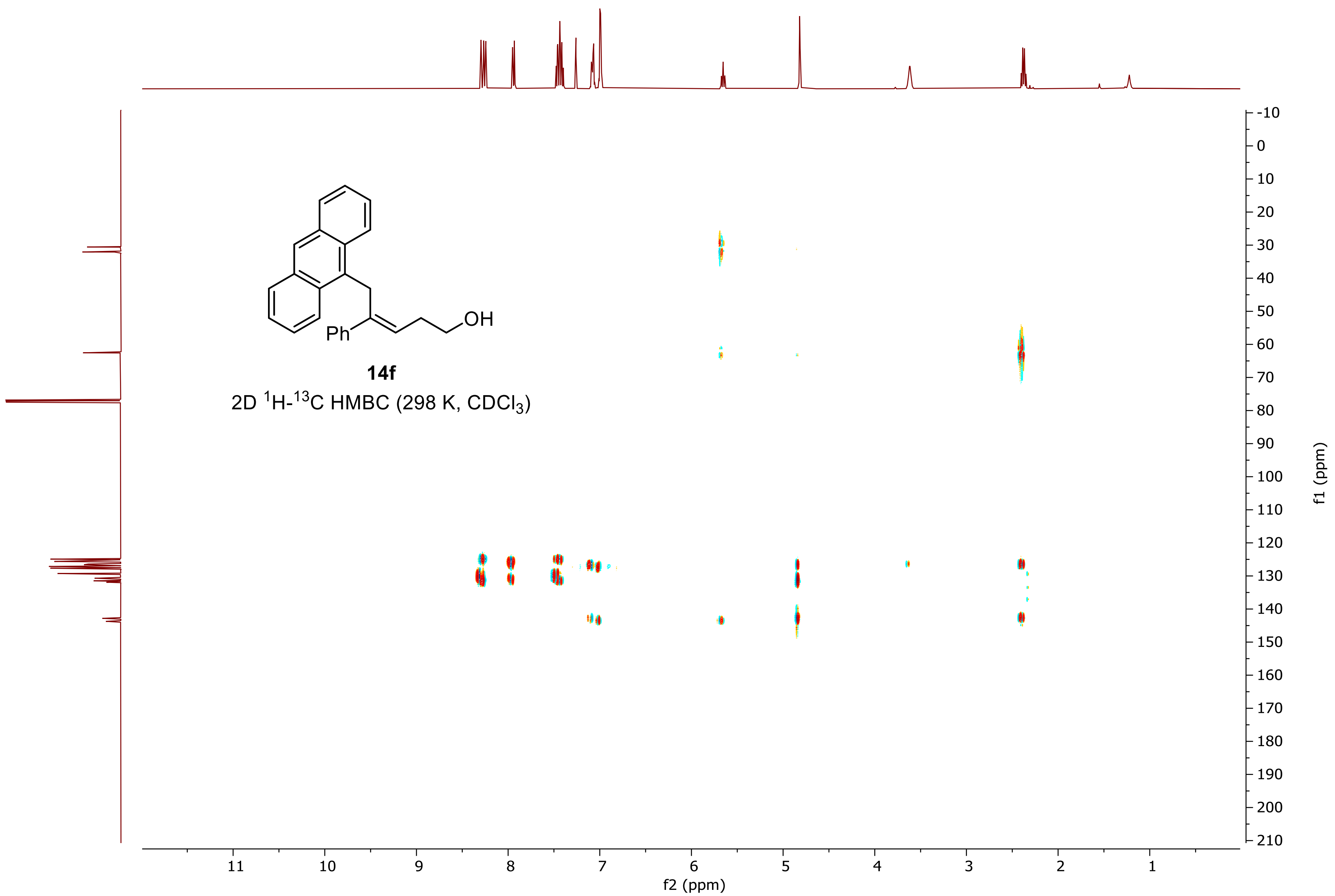
Ph
OH
14f
2D ^{1}H-^{13}C HMBC (298 K, $CDCl_3$)
f2 (ppm)
f1 (ppm)

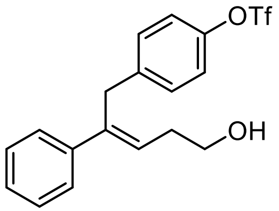

14g

^{1}H NMR (400 MHz, 298 K, $CDCl_3$)

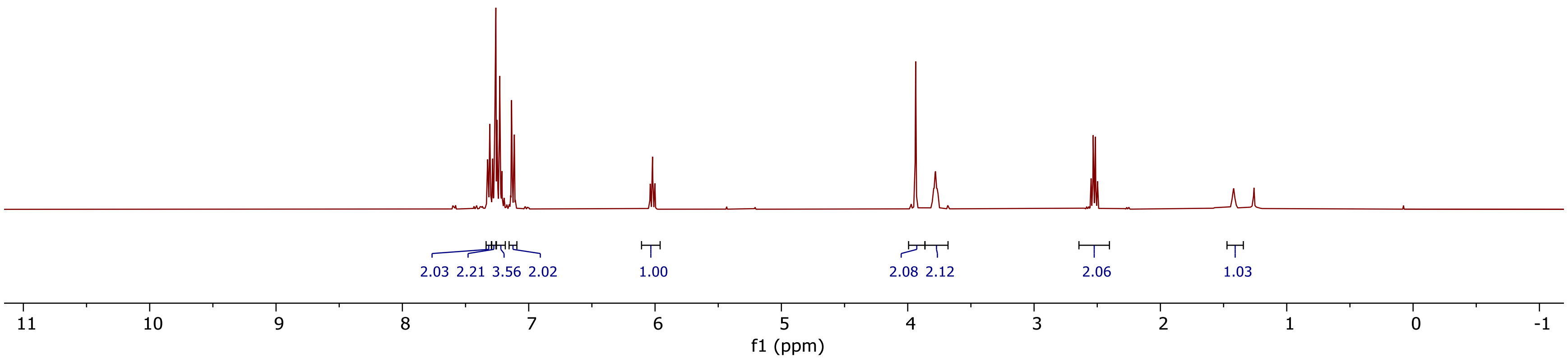

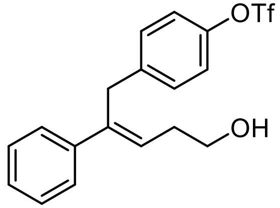

14g

$^{13}C\{^{1}H\}$ NMR (101 MHz, 298 K, $CDCl_3$)

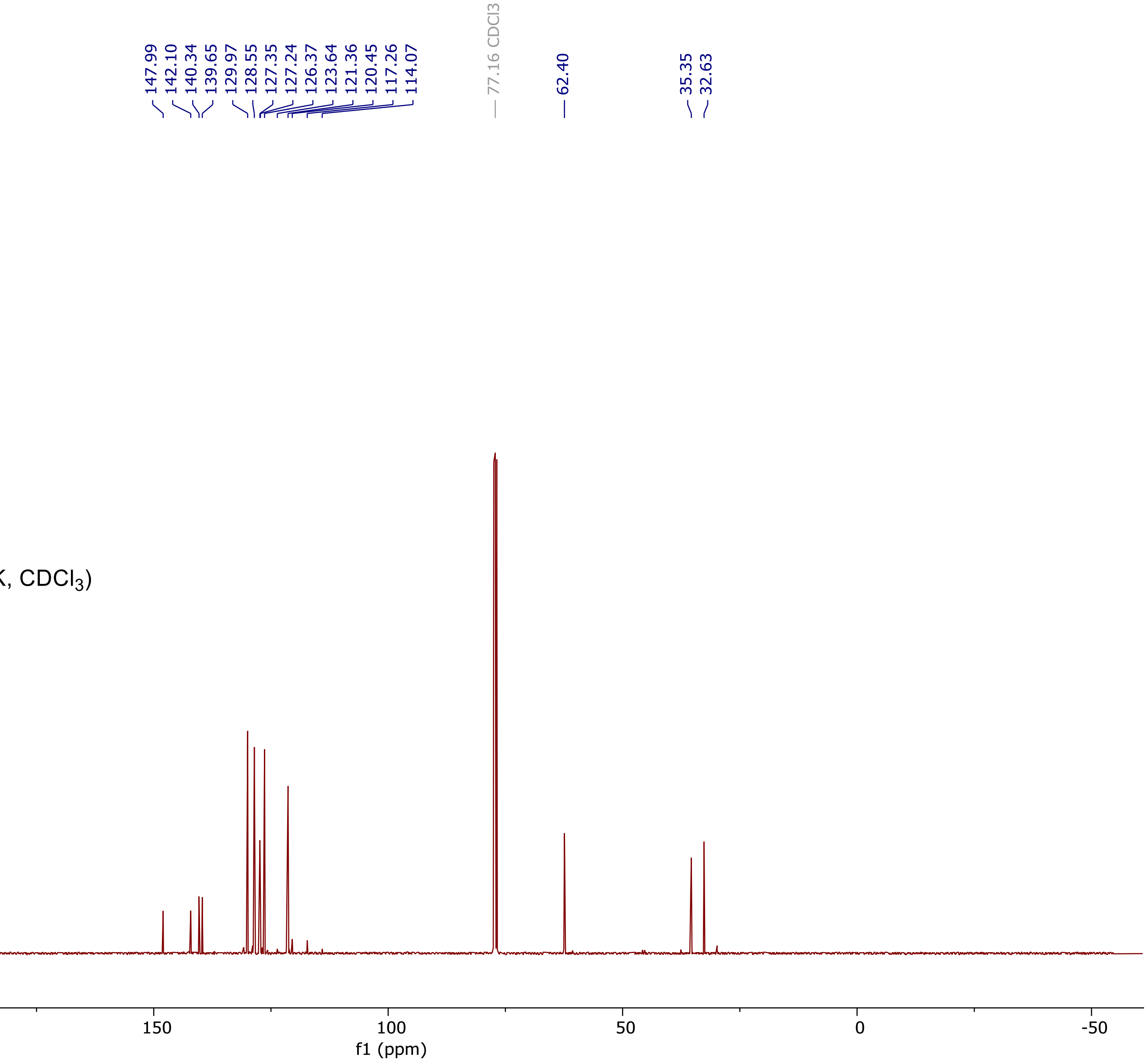

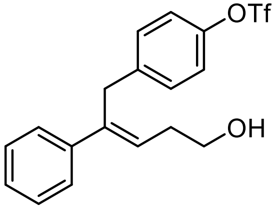

14g

$^{19}F\{^{1}H\}$ NMR (282 MHz, 298 K, $CDCl_3$)

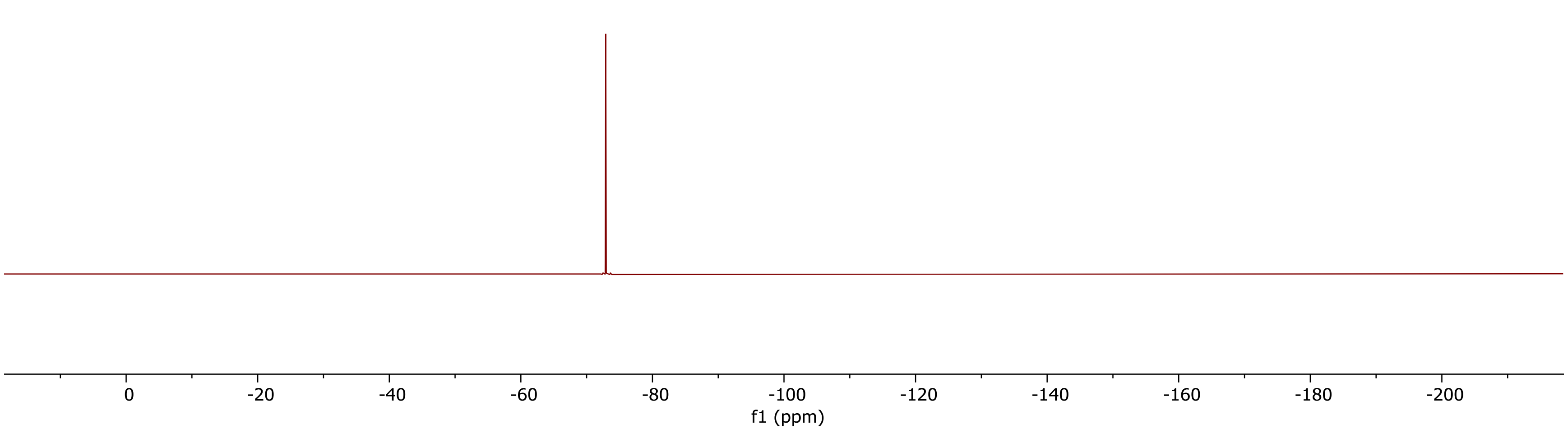

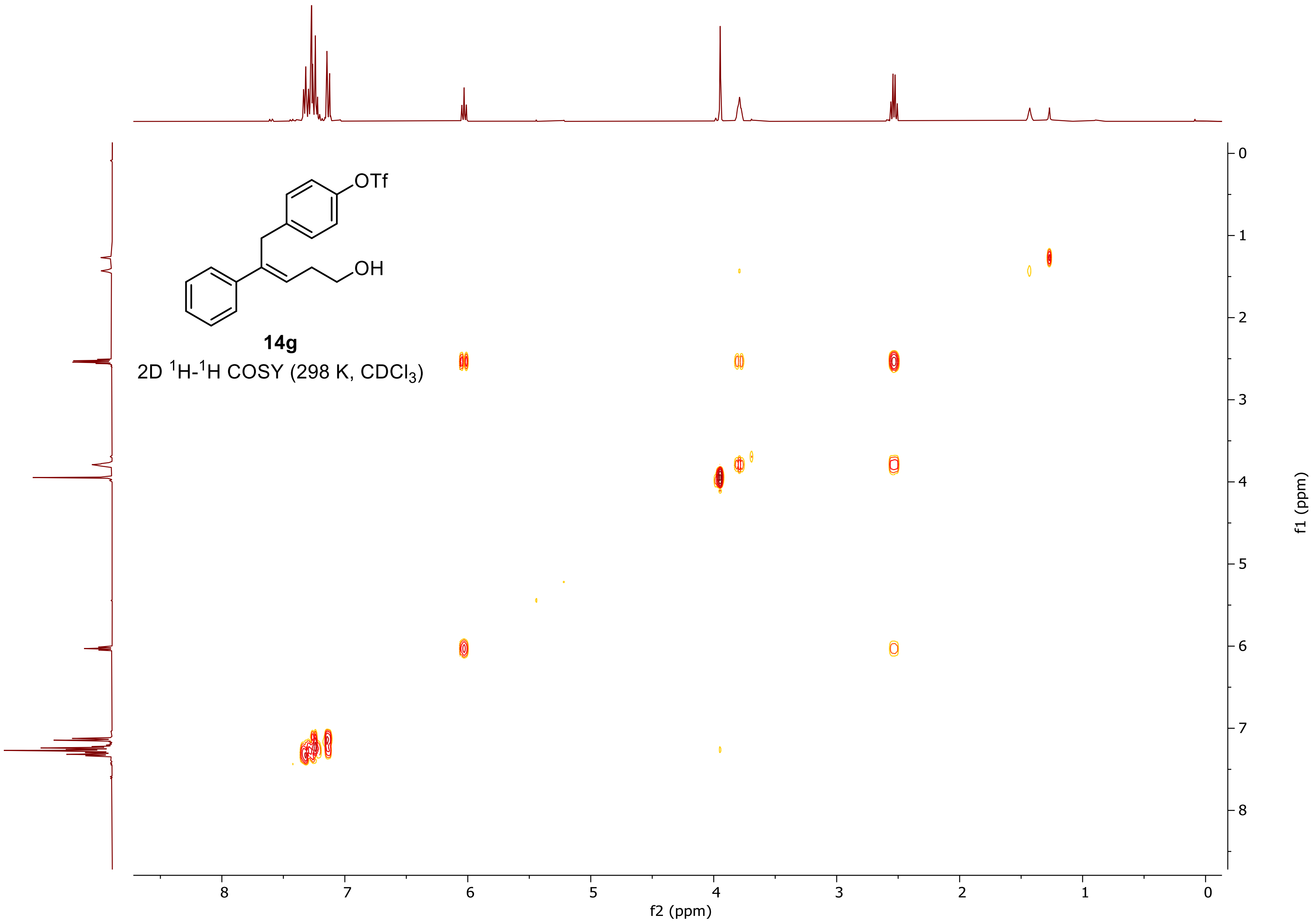
OTf
OH
14g
2D 1H-1H COSY (298 K, CDCl3)
f2 (ppm)
f1 (ppm)
8
7
6
5
4
3
2
1
0

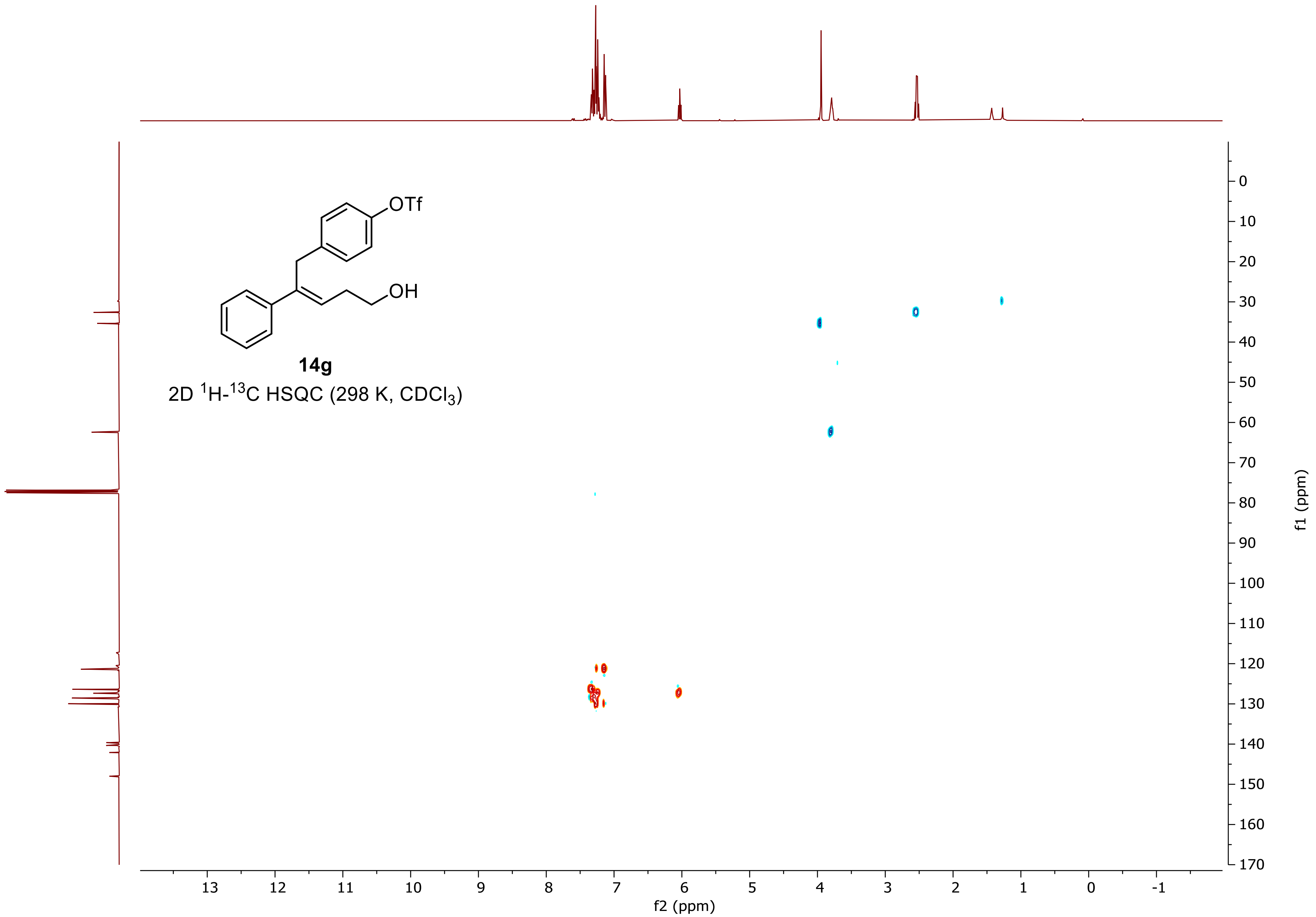

14g

2D ^{1}H-^{13}C HSQC (298 K, $CDCl_3$)

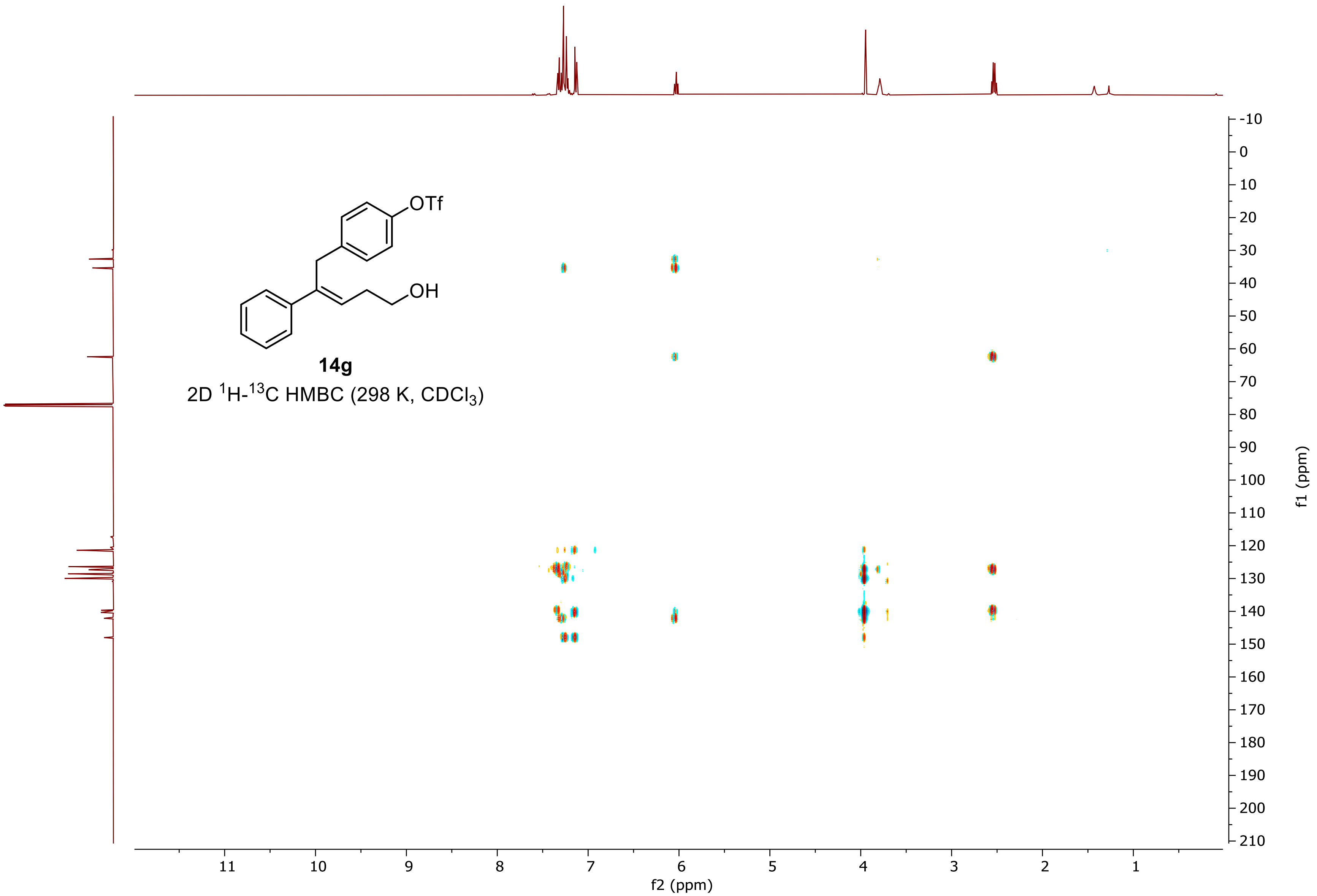

14g

2D ^{1}H-^{13}C HMBC (298 K, $CDCl_3$)

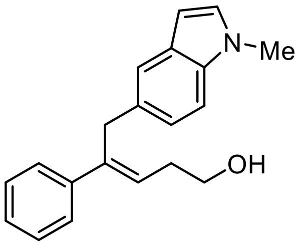

14h

^{1}H NMR (400 MHz, 298 K, $CDCl_3$)

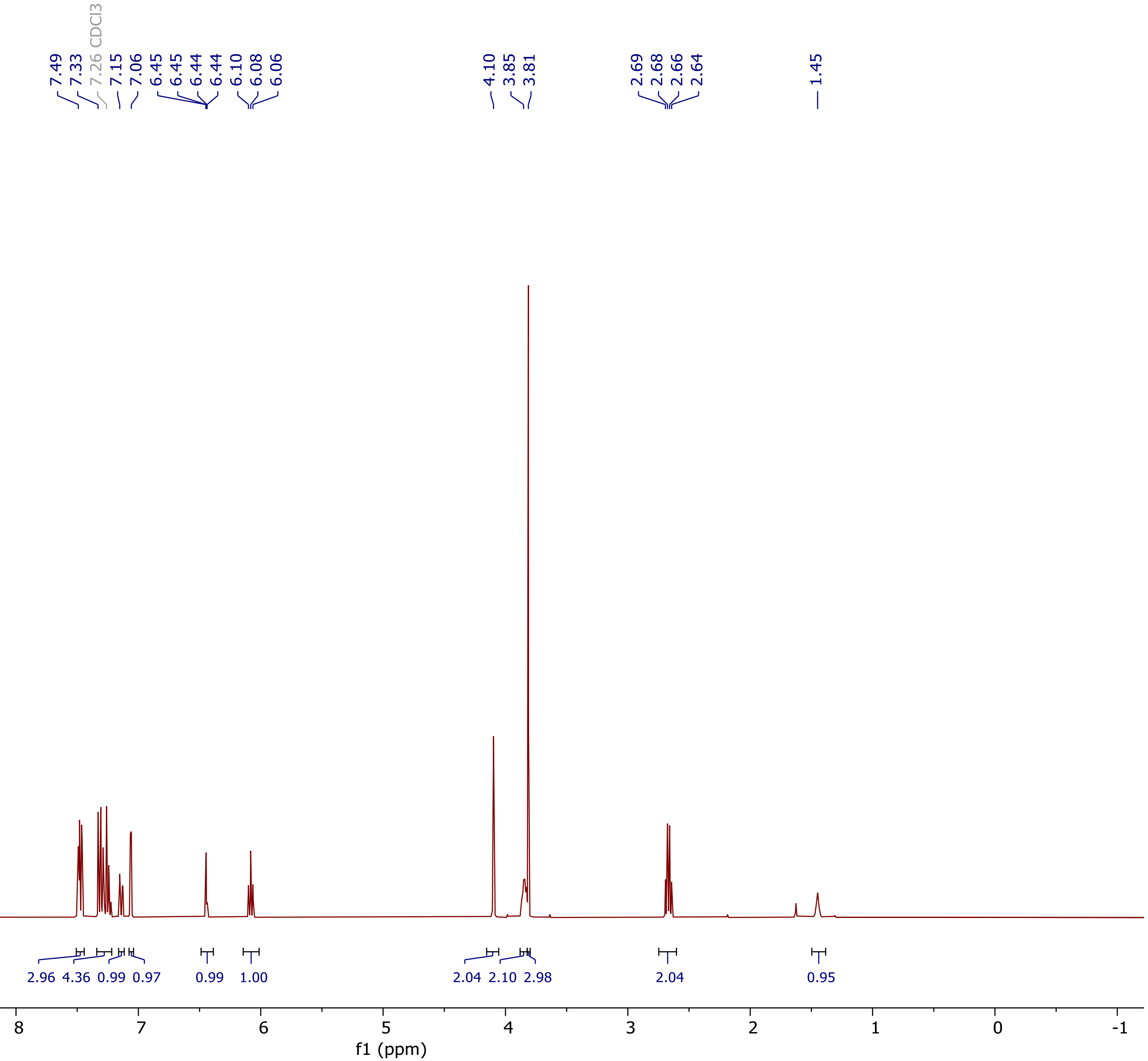

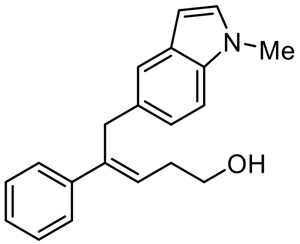

14h

$^{13}C\{^{1}H\}$ NMR (101 MHz, 298 K, $CDCl_3$)

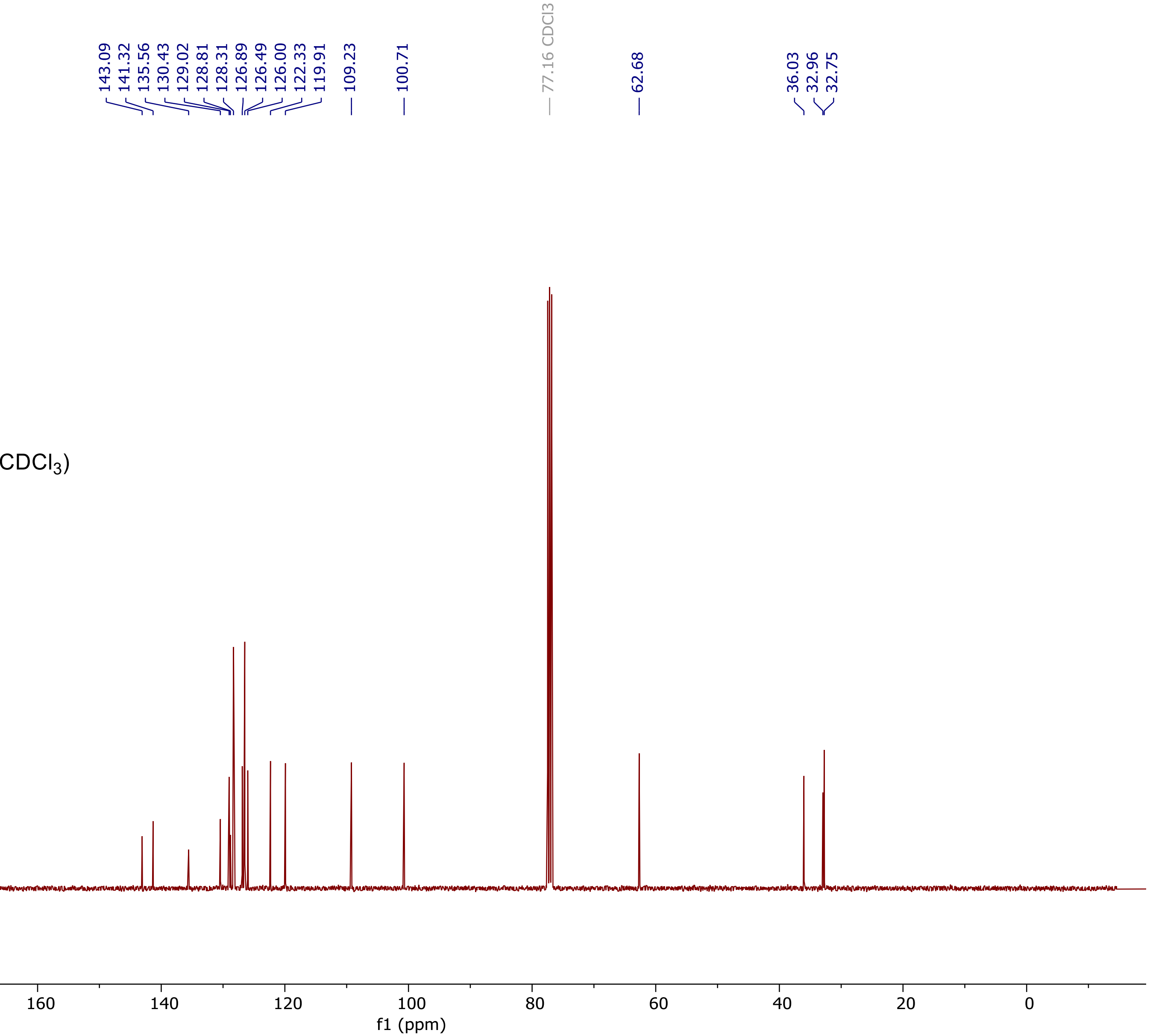

14h

2D ^{1}H-^{1}H COSY (298 K, $CDCl_3$)

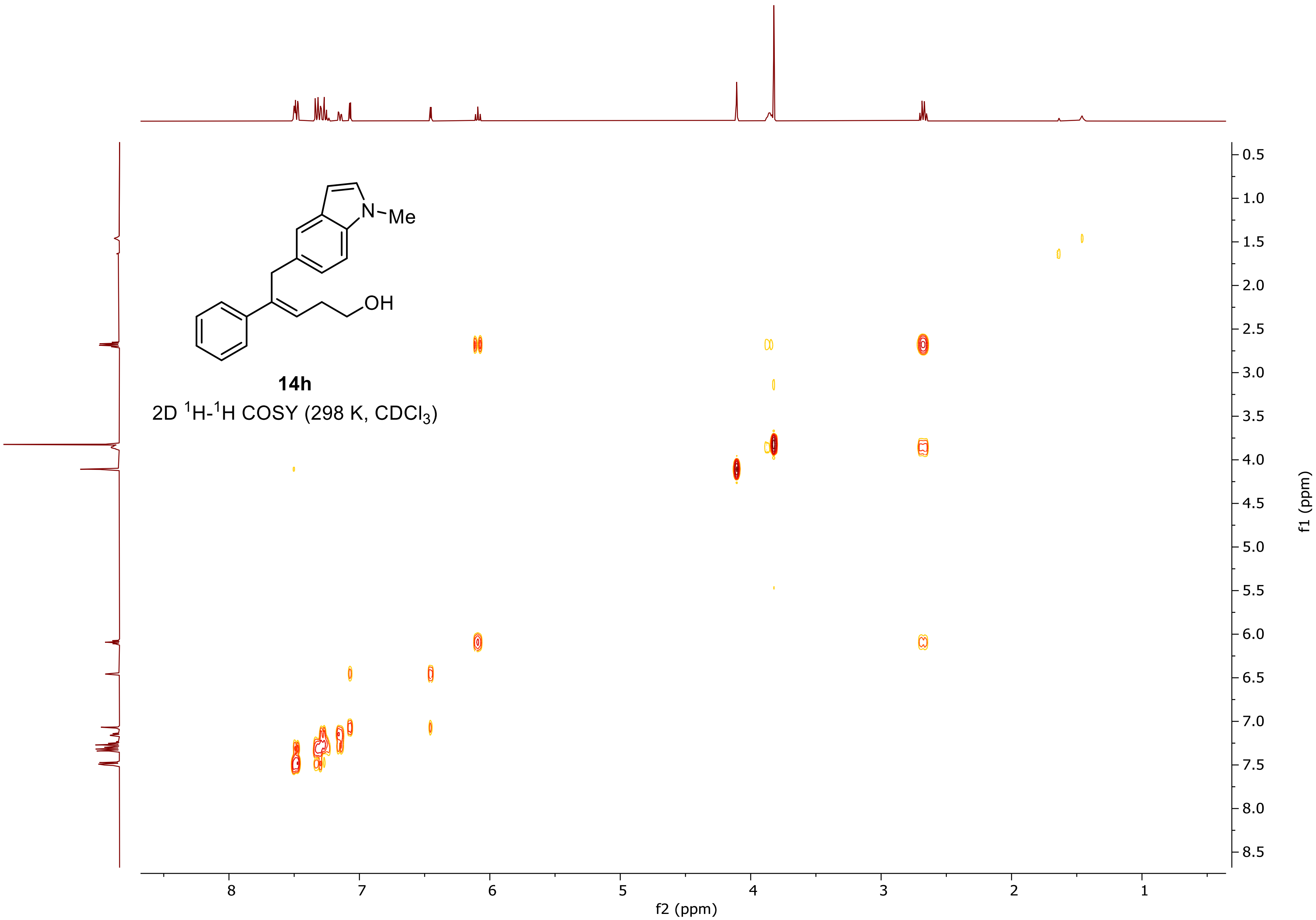

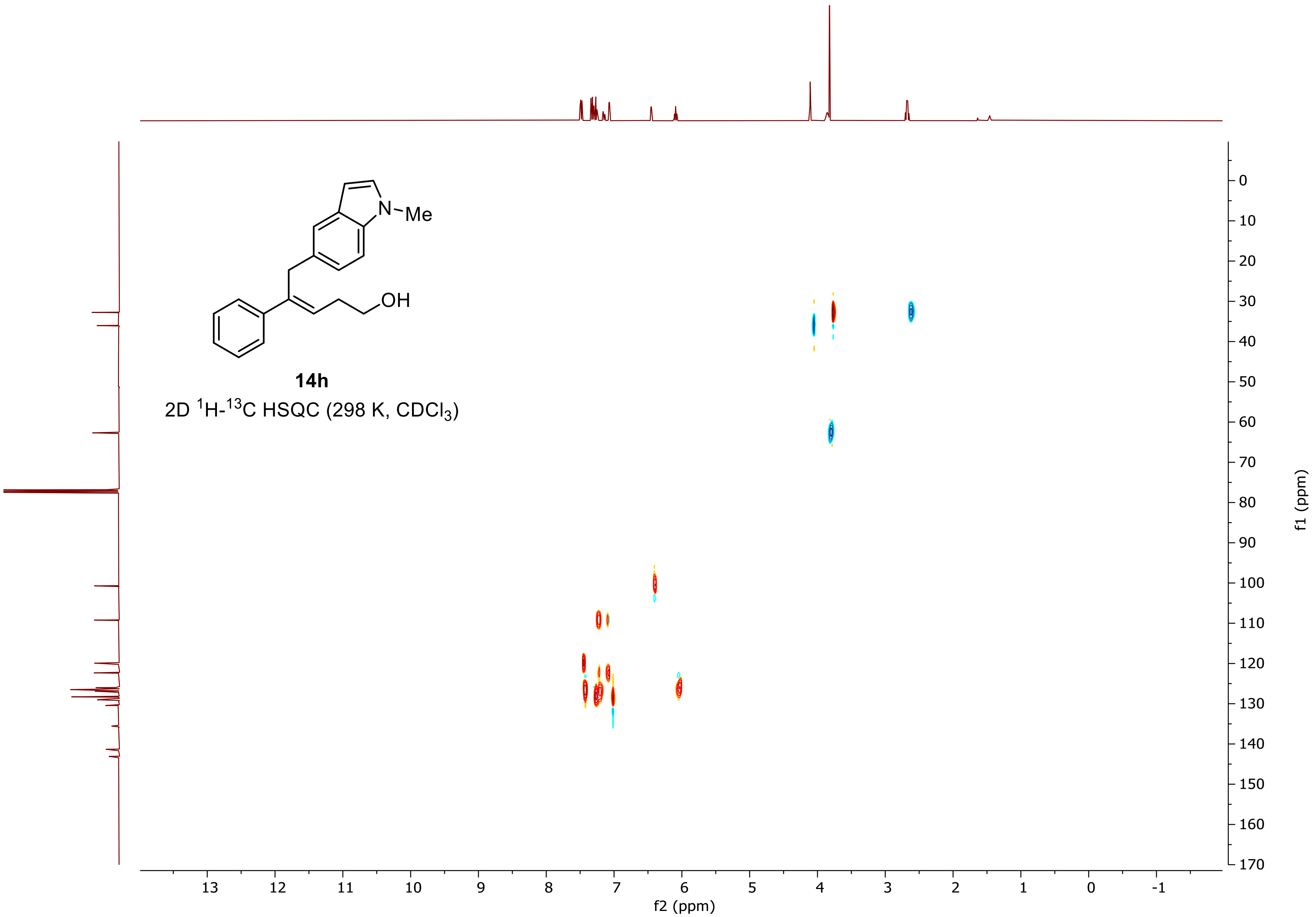
N
Me
OH
14h
2D ¹H-¹³C HSQC (298 K, CDCl₃)
f2 (ppm)
f1 (ppm)

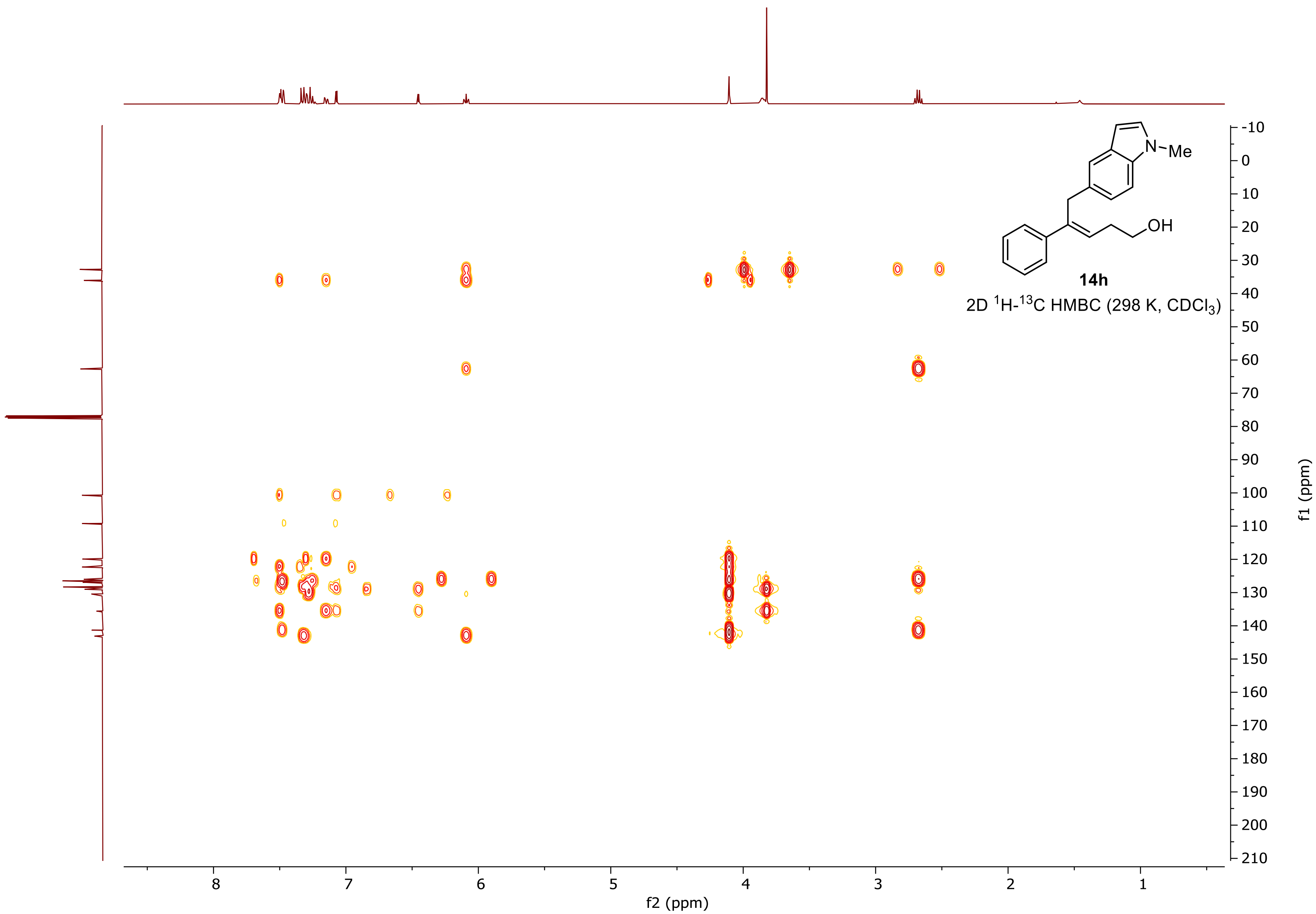
N-Me
OH
14h
2D ^{1}H-^{13}C HMBC (298 K, $CDCl_3$)
f2 (ppm)
f1 (ppm)

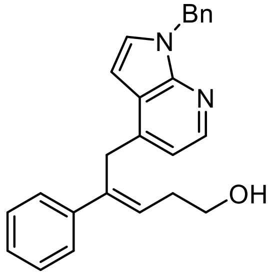

14i

$^{13}C\{^{1}H\}$ NMR (101 MHz, 298 K, $CDCl_3$)

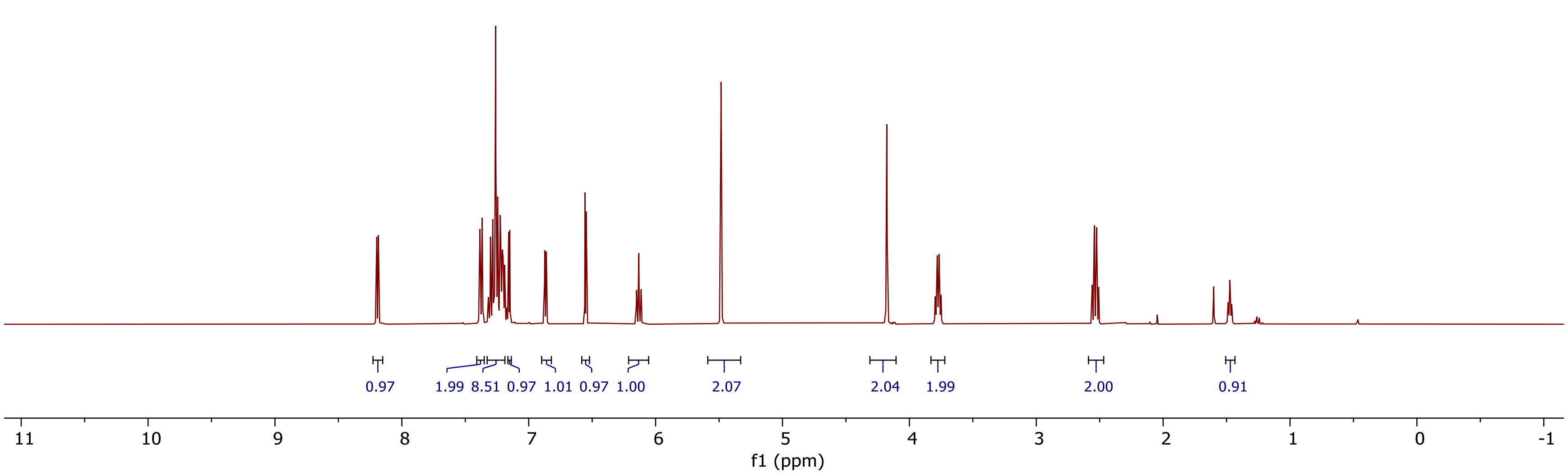

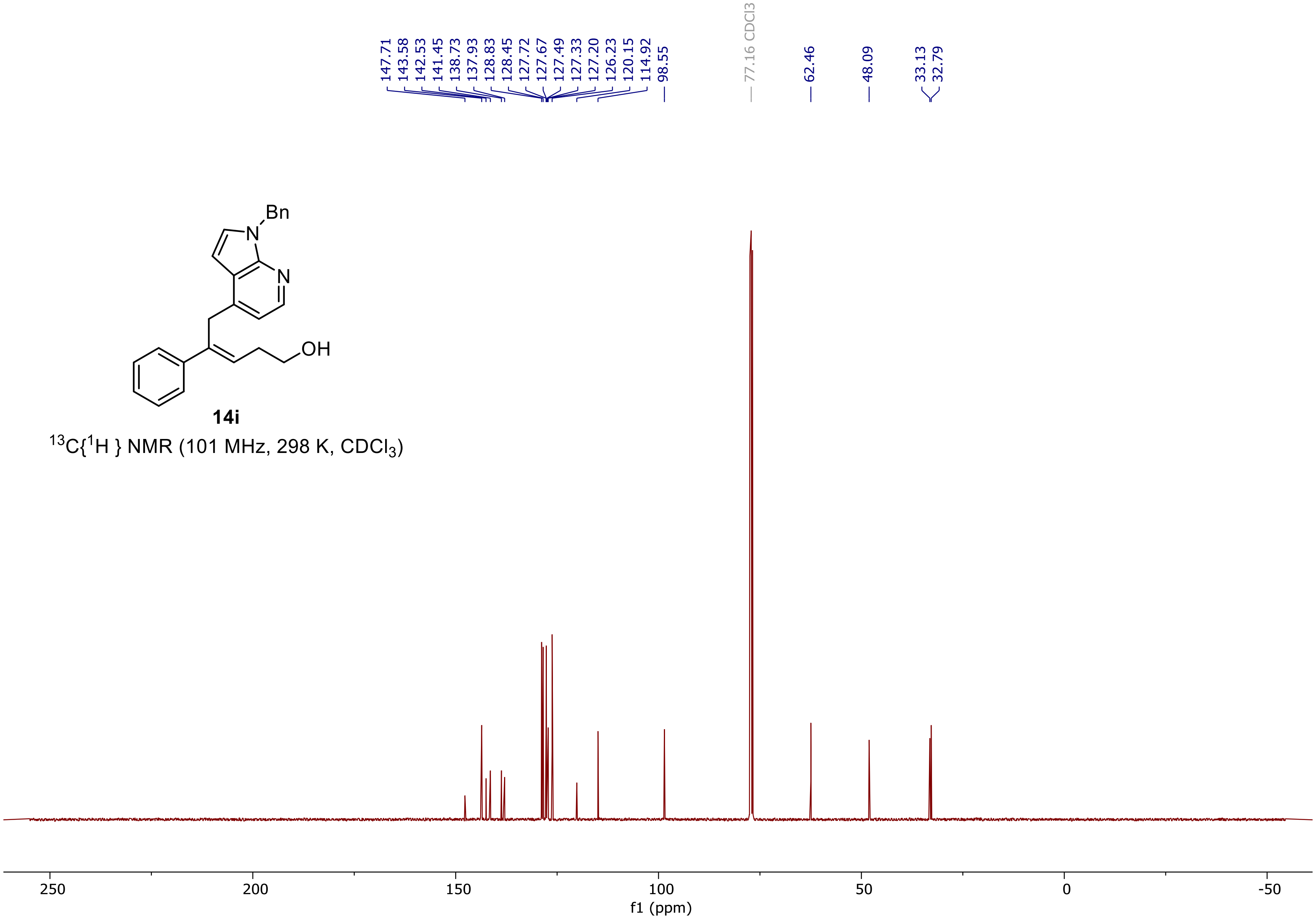

14i

$^{13}C\{^{1}H\}$ NMR (101 MHz, 298 K, $CDCl_3$)

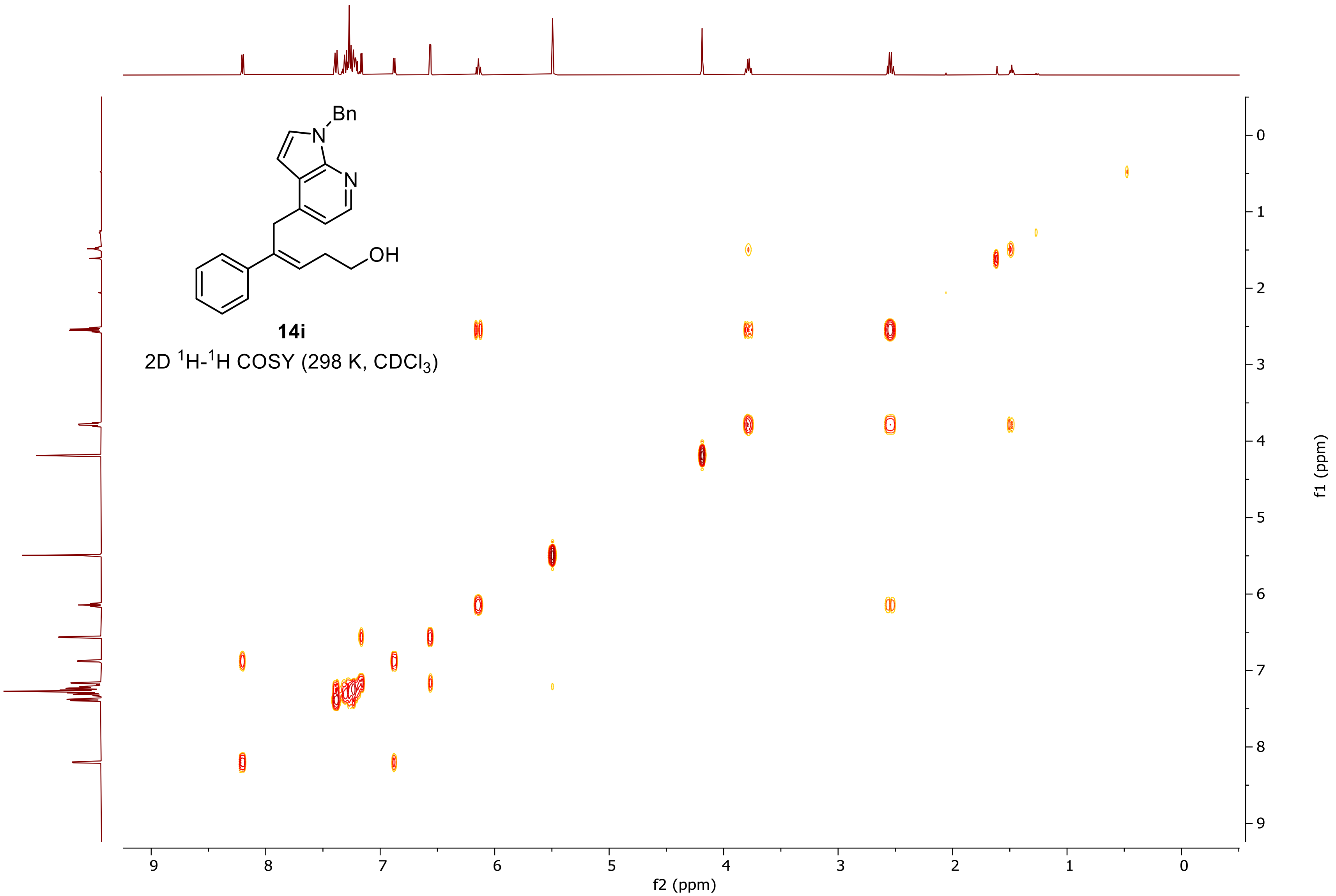
Bn
N
N
OH
14i
2D 1H-1H COSY (298 K, CDCl3)
f2 (ppm)
f1 (ppm)
9 8 7 6 5 4 3 2 1 0

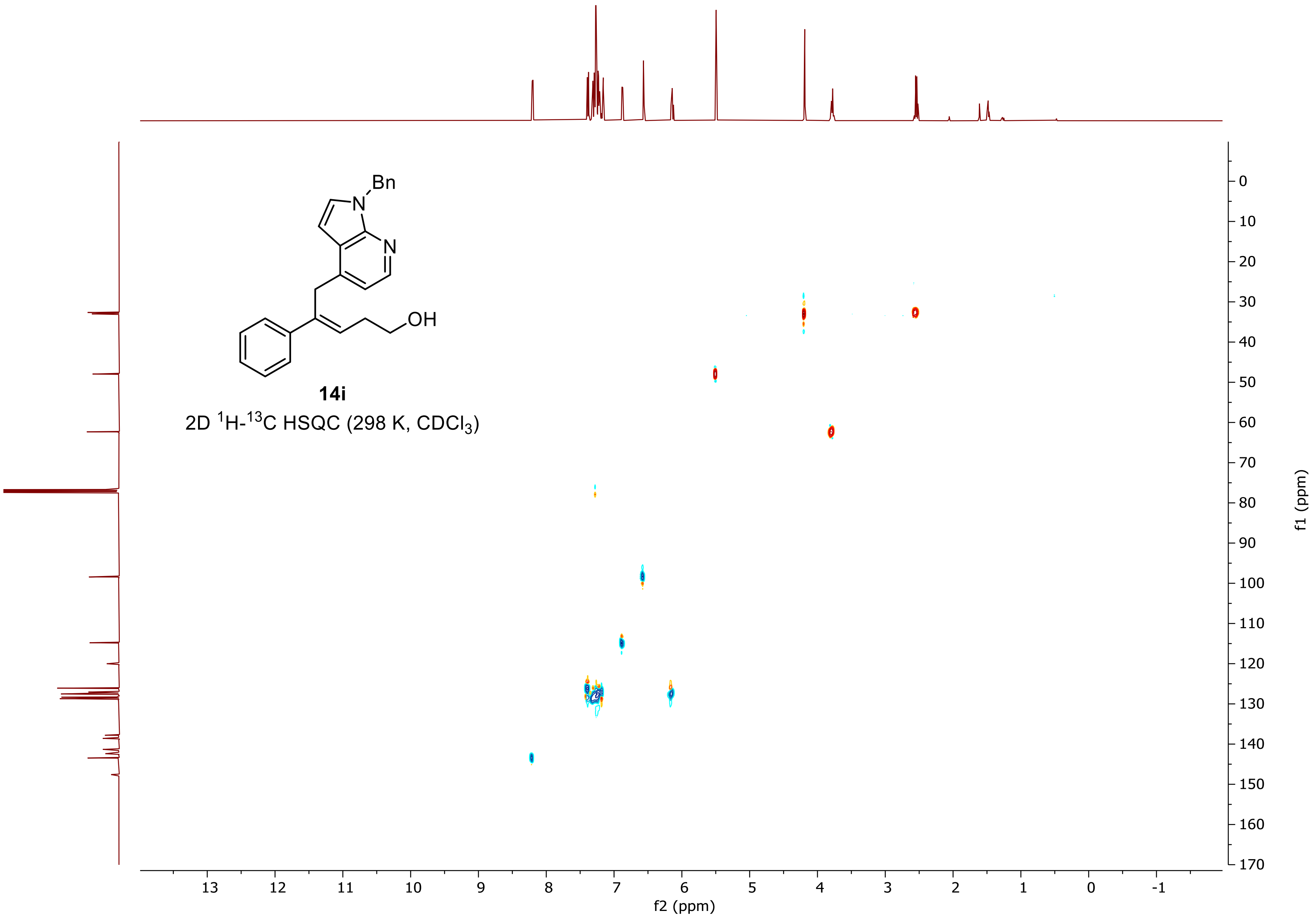

14i

2D ^{1}H-^{13}C HSQC (298 K, $CDCl_3$)

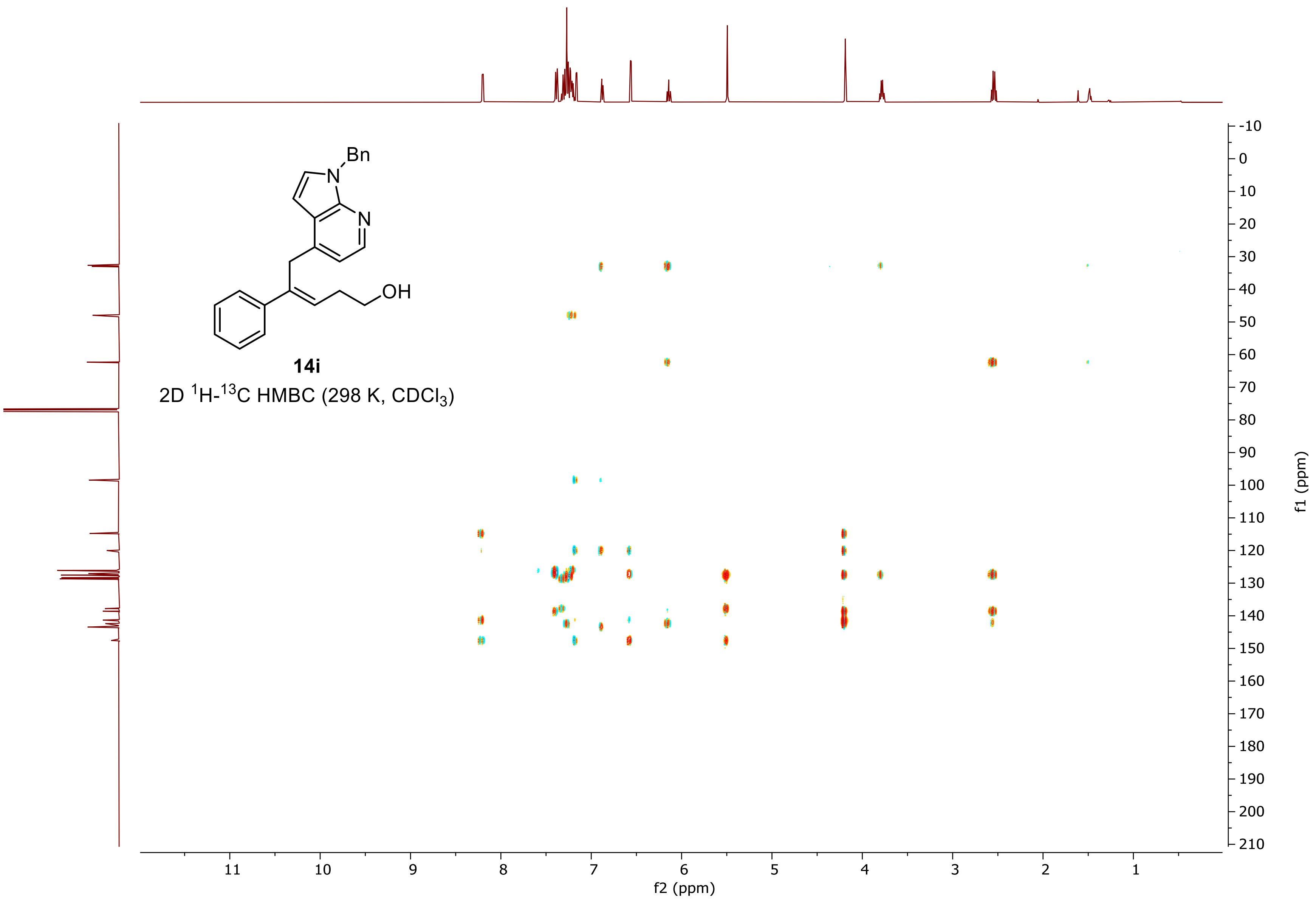

14i

2D ^{1}H-^{13}C HMBC (298 K, $CDCl_3$)

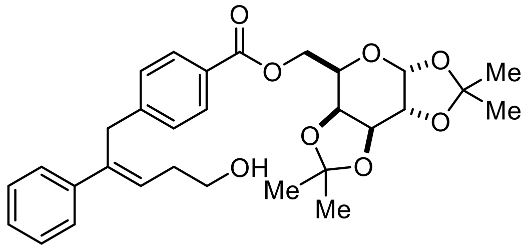

14j

^{1}H NMR (400 MHz, 298 K, $CDCl_3$)

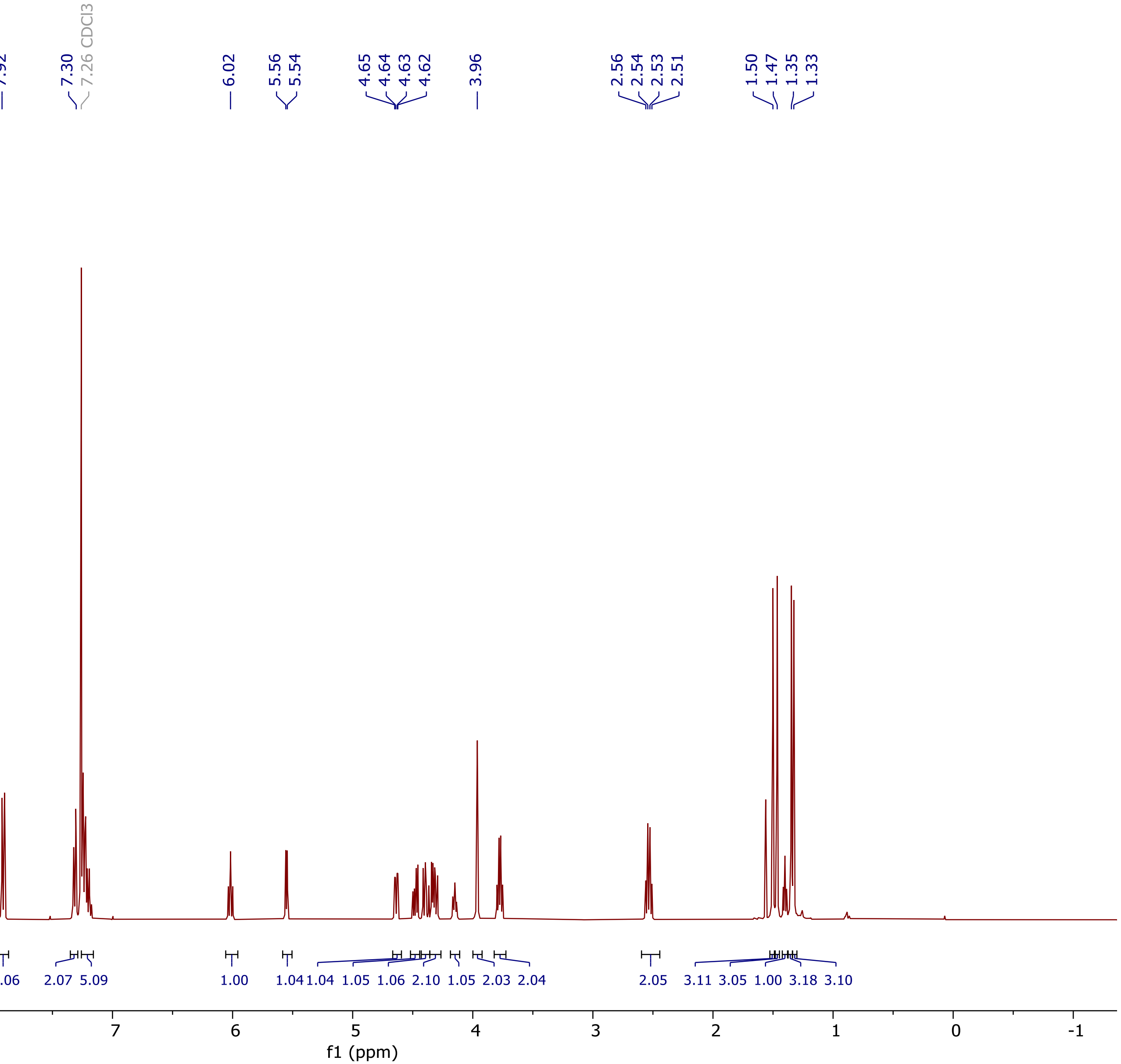

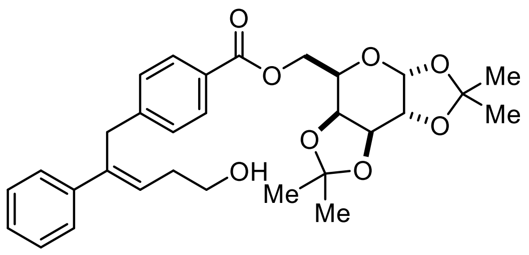

14j

$^{13}C\{^1H\}$ NMR (101 MHz, 298 K, $CDCl_3$)

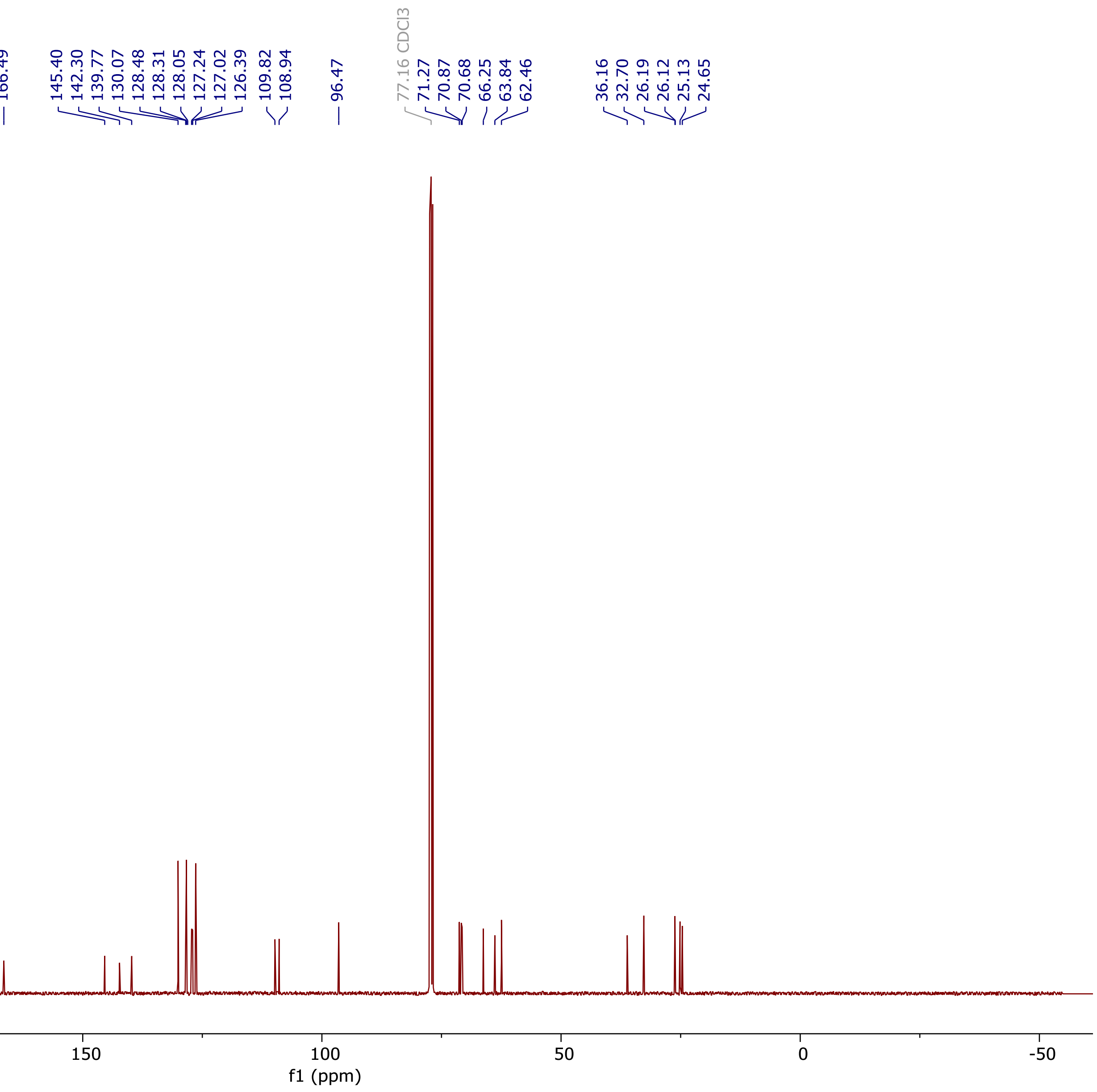

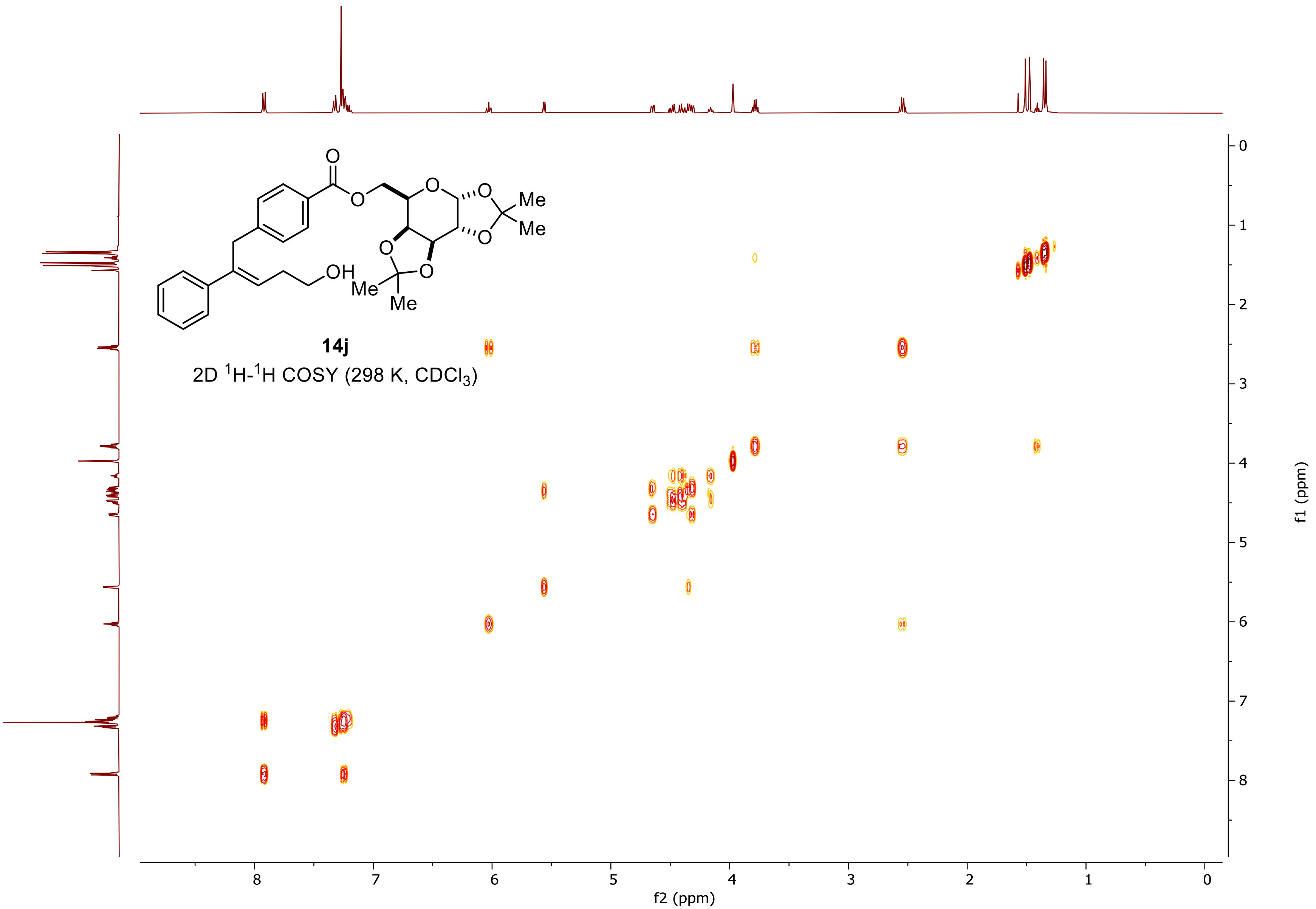

14j

2D ^{1}H-^{1}H COSY (298 K, $CDCl_3$)

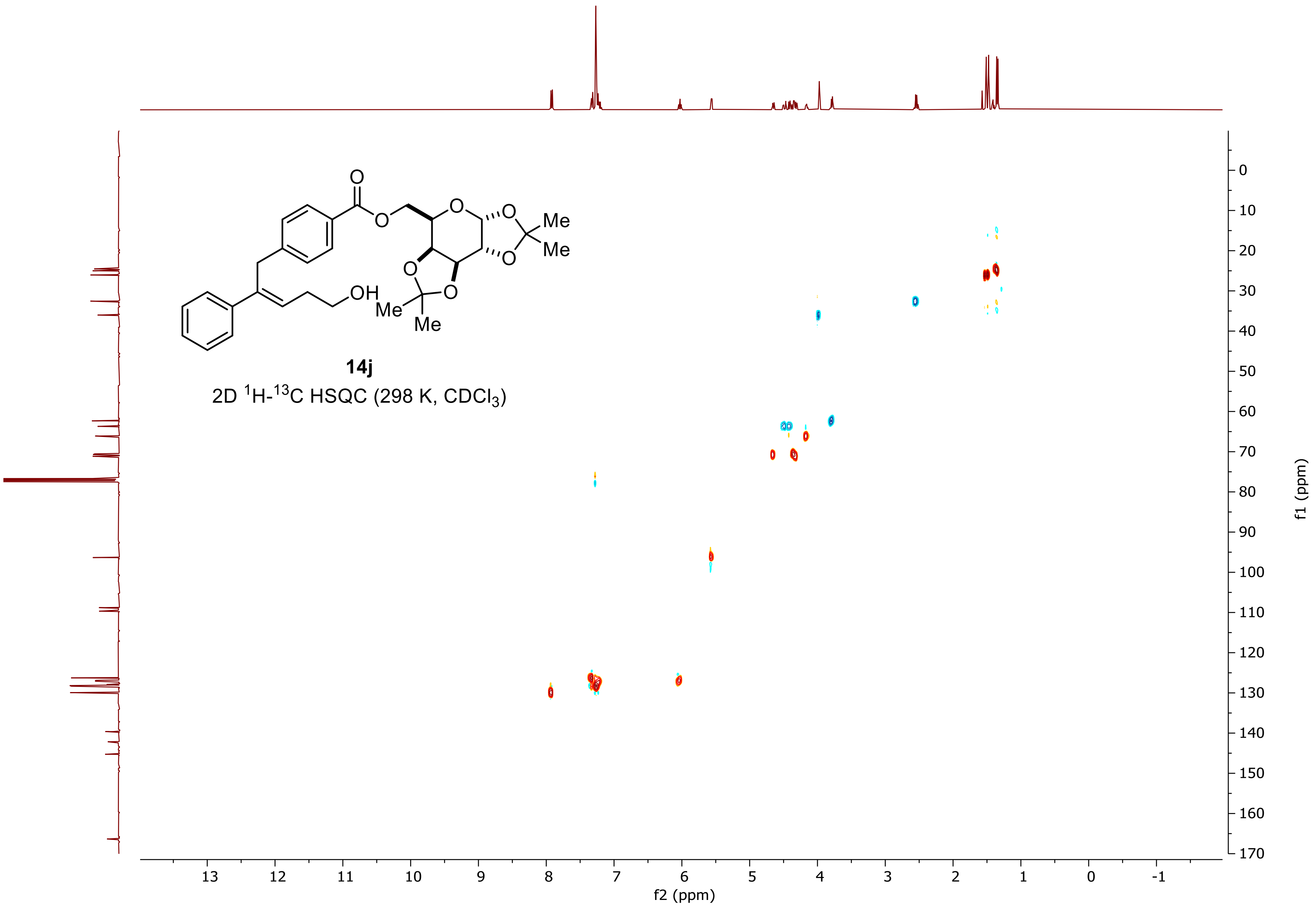
14j
2D ^{1}H-^{13}C HSQC (298 K, $CDCl_3$)
OH
Me
Me
Me
Me
O
f2 (ppm)
f1 (ppm)

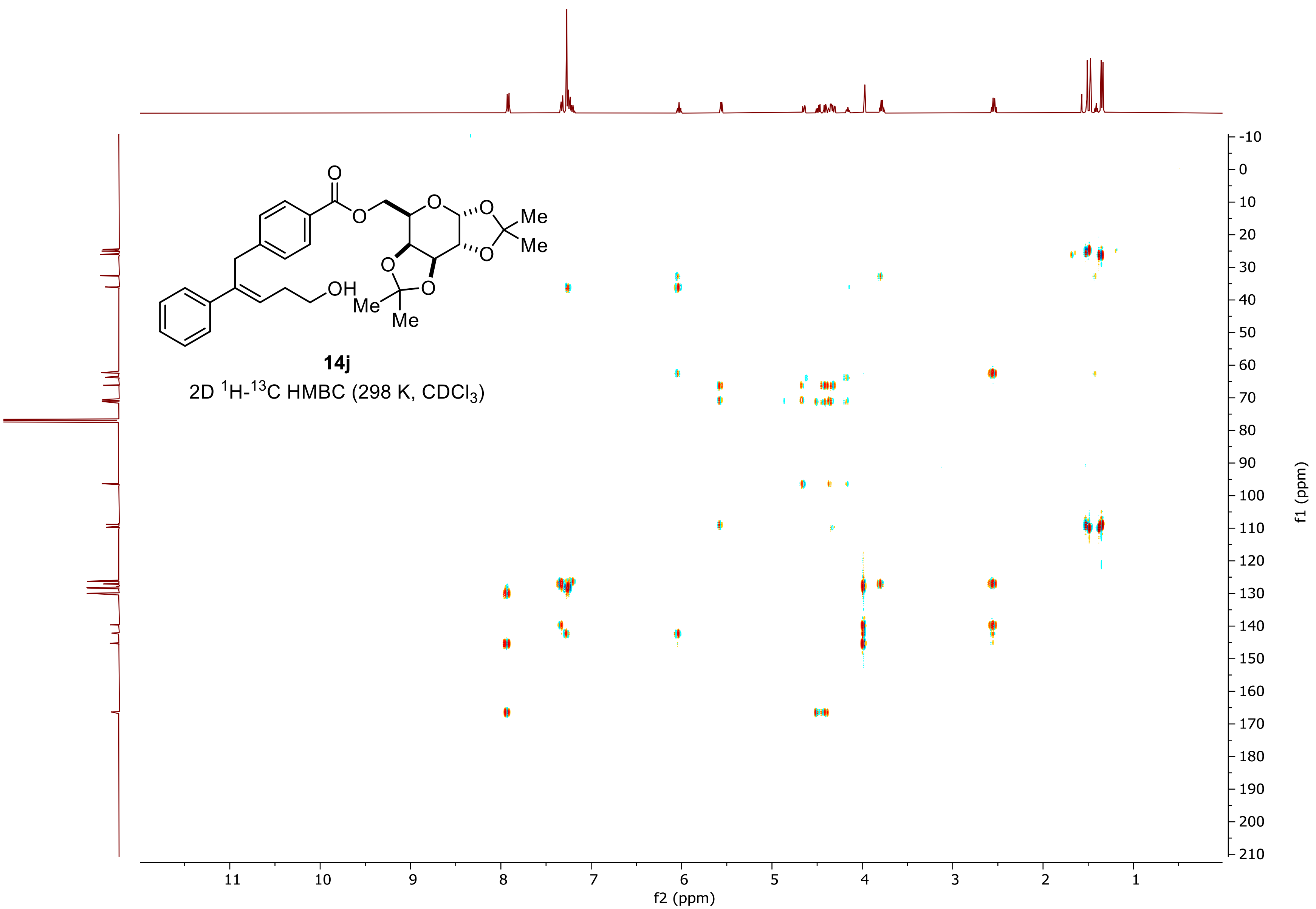
O
O
O
O
O
O
O
OH
Me
Me
Me
Me
14j
2D 1H-13C HMBC (298 K, CDCl3)
f1 (ppm)
f2 (ppm)

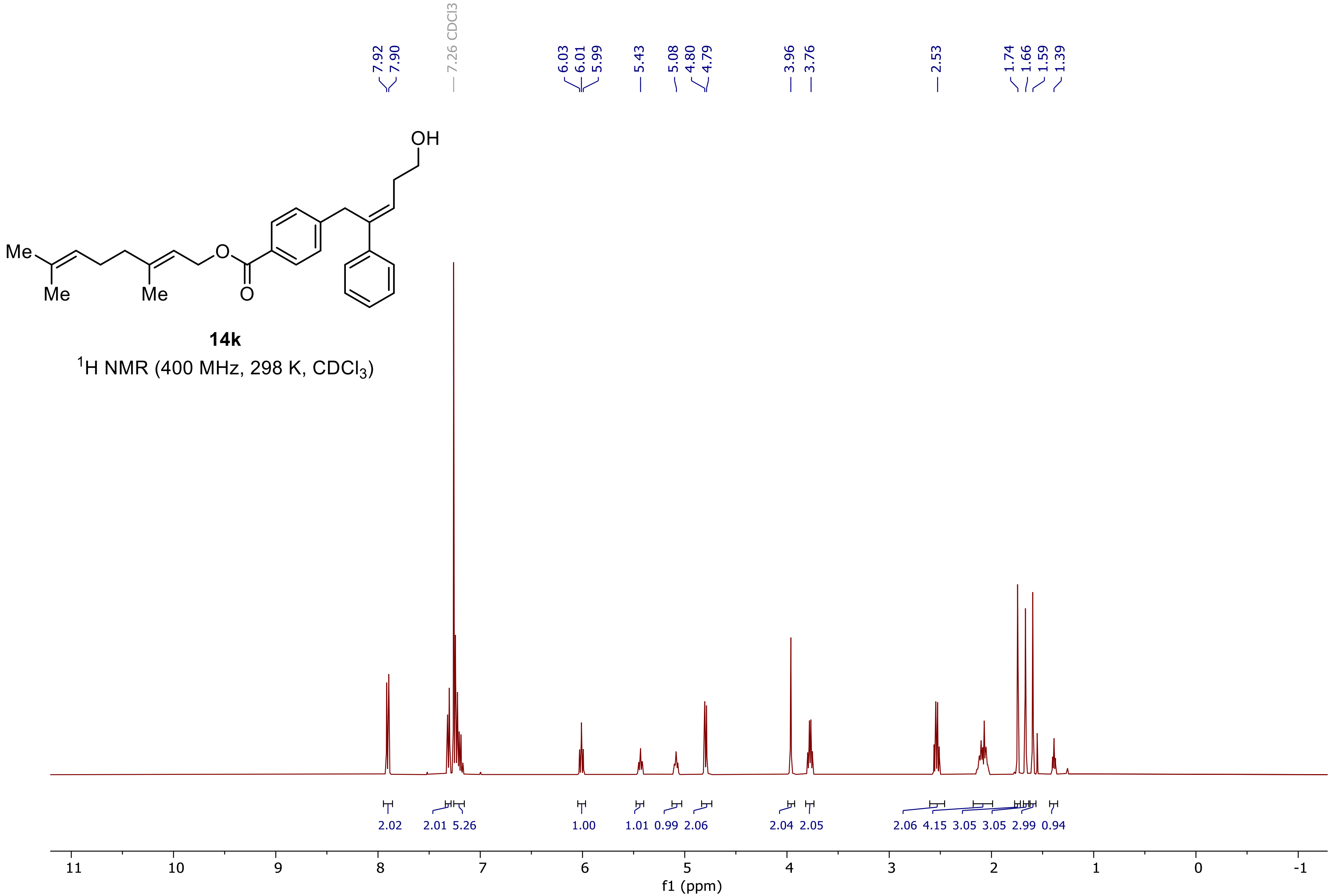

14k

^{1}H NMR (400 MHz, 298 K, $CDCl_3$)

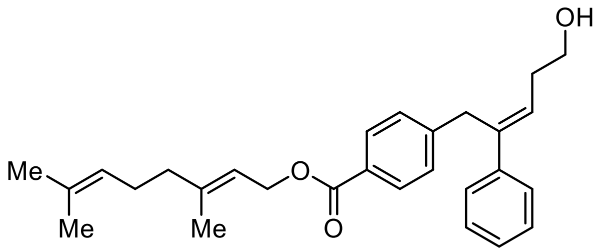

14k

$^{13}C\{^1H\}$ NMR (101 MHz, 298 K, $CDCl_3$)

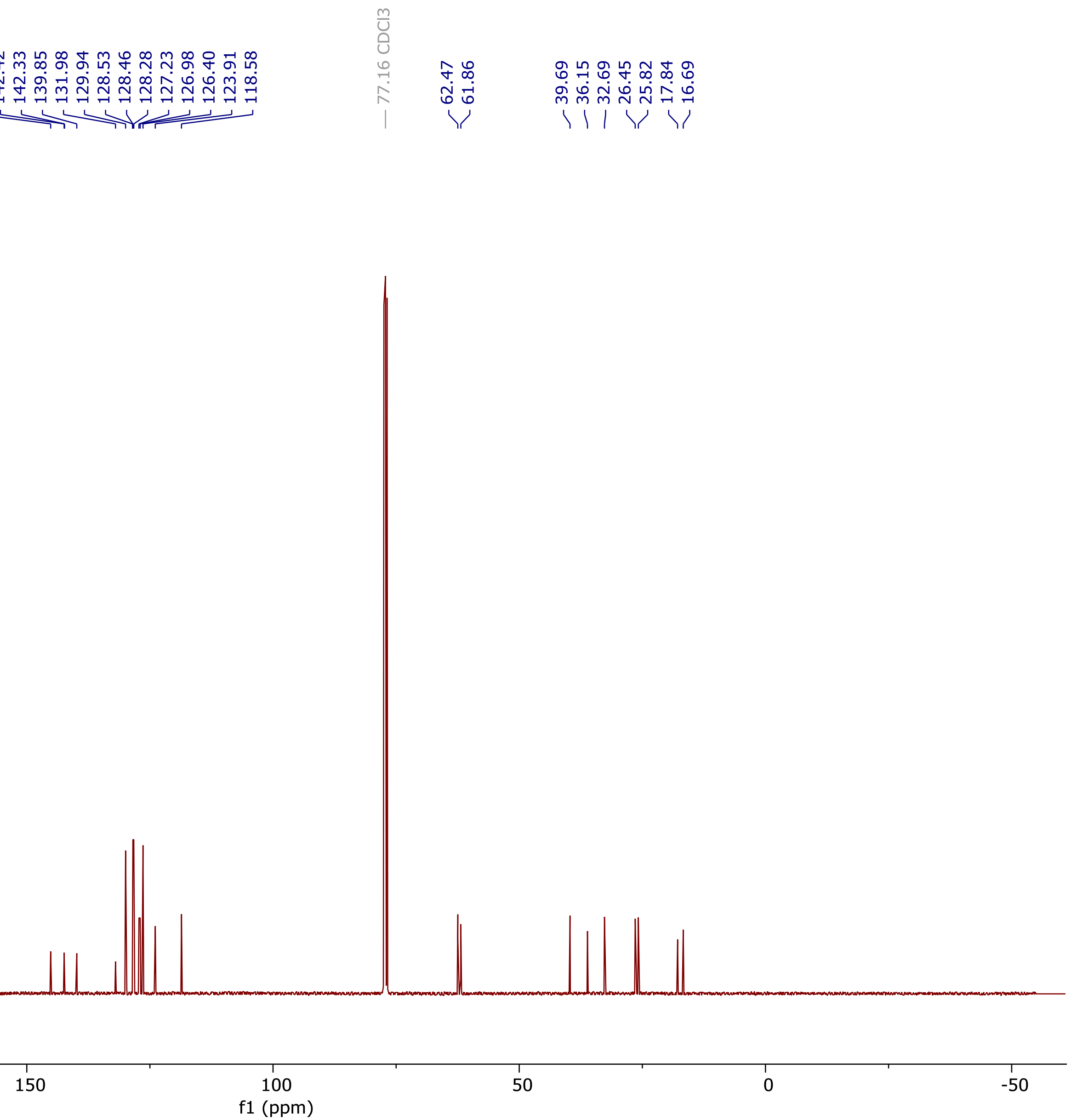

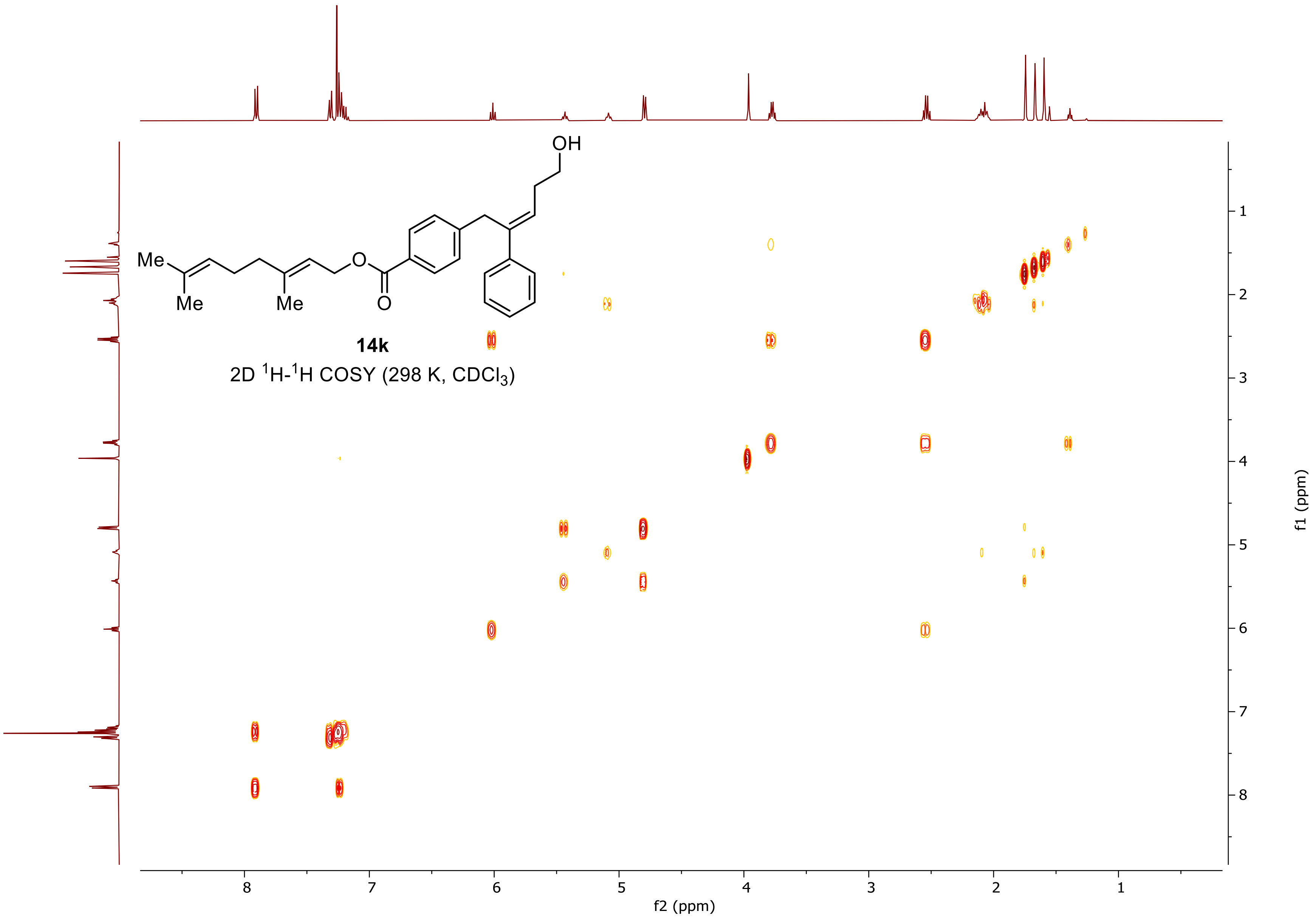
OH
Me
Me
Me
O
O
14k
2D ¹H-¹H COSY (298 K, CDCl₃)
f2 (ppm)
f1 (ppm)

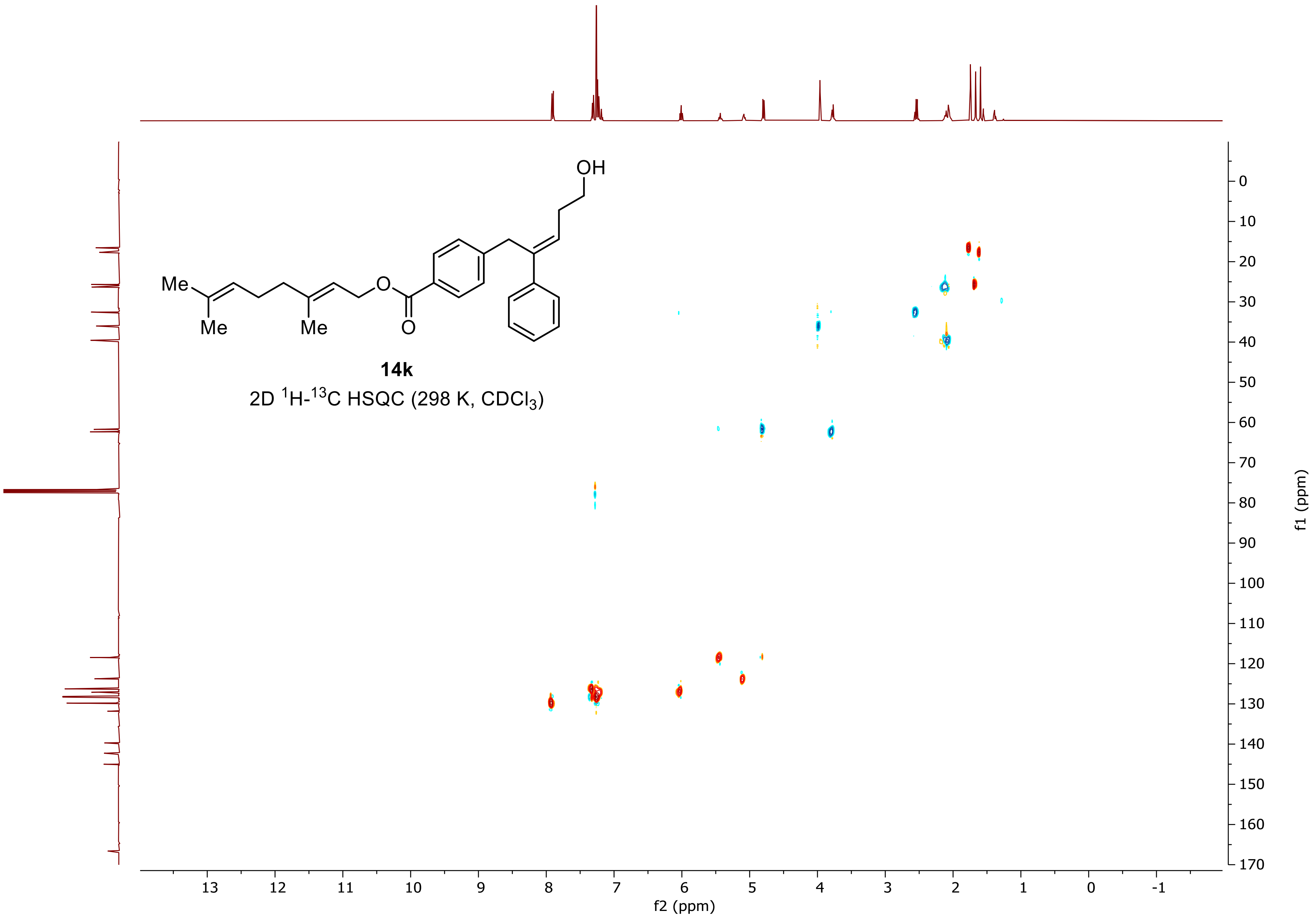

14k

2D ^{1}H-^{13}C HSQC (298 K, $CDCl_3$)

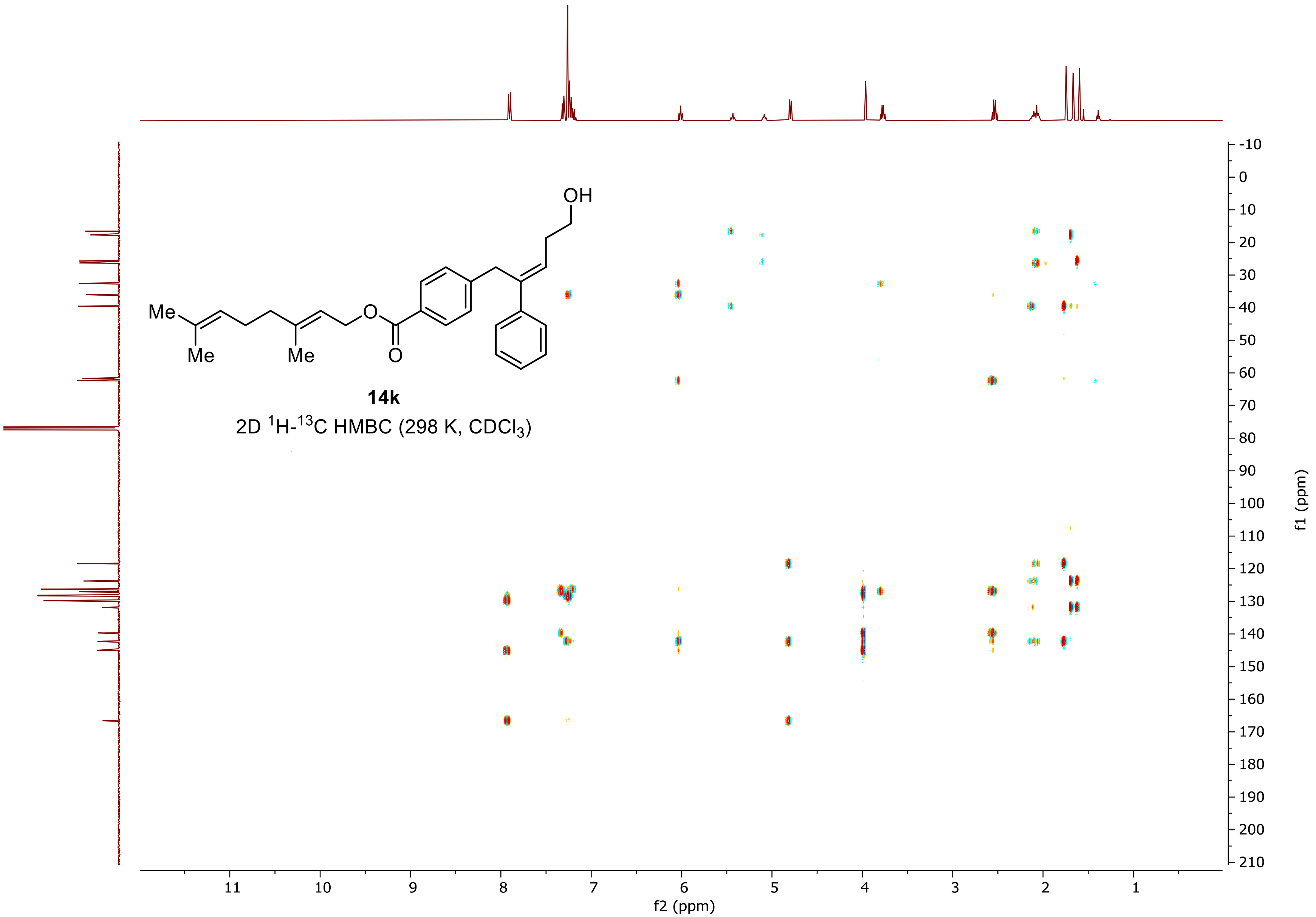
OH
Me
Me
Me
O
O
14k
2D 1H-13C HMBC (298 K, CDCl3)
f1 (ppm)
f2 (ppm)

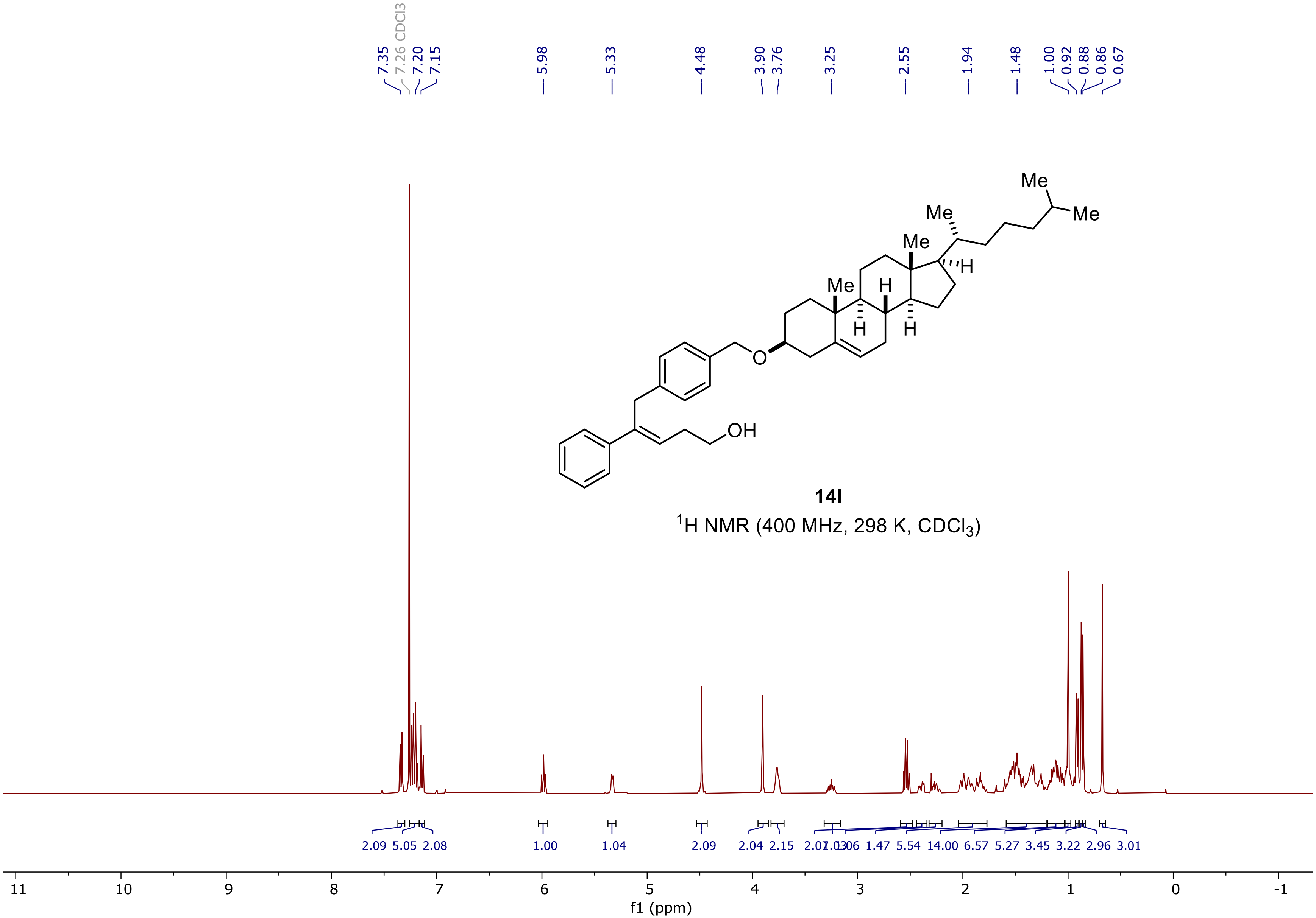

14l

^{1}H NMR (400 MHz, 298 K, $CDCl_3$)

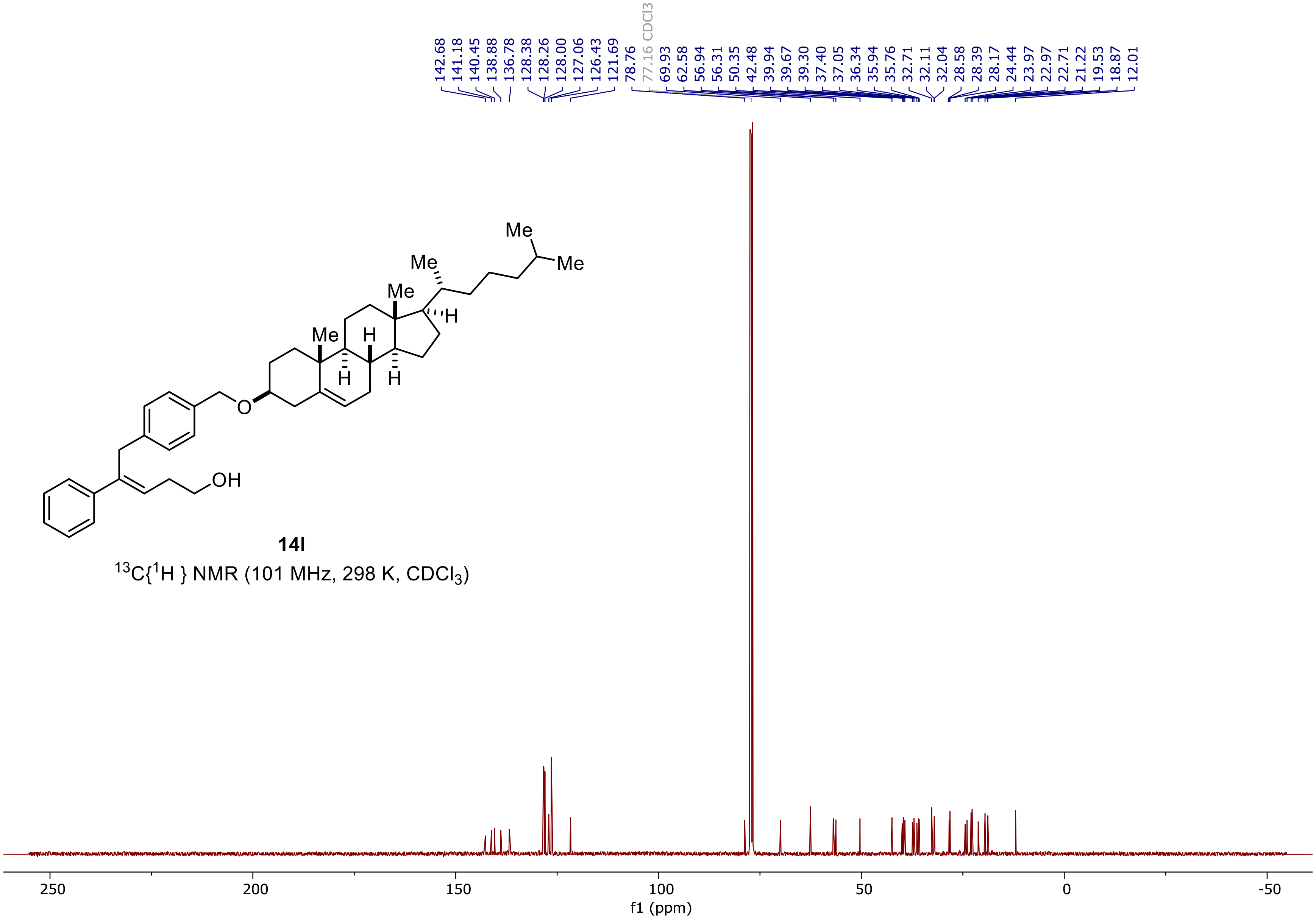

14l

$^{13}C\{^{1}H\}$ NMR (101 MHz, 298 K, $CDCl_3$)

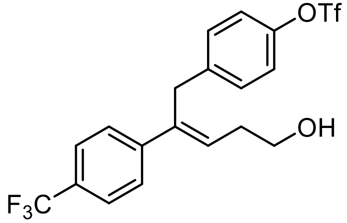

14m

^{1}H NMR (400 MHz, 298 K, $CDCl_3$)

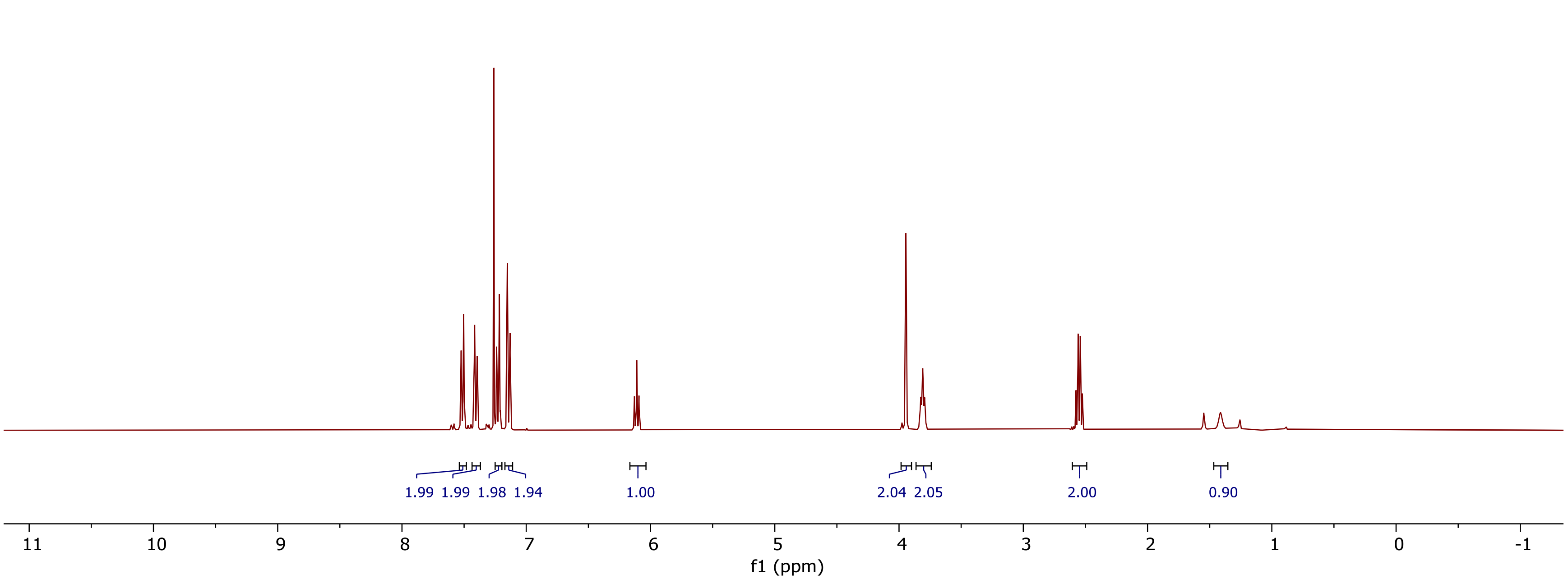

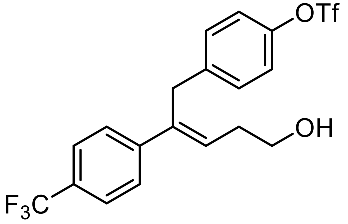

14m

$^{13}C\{^1H\}$ NMR (101 MHz, 298 K, $CDCl_3$)

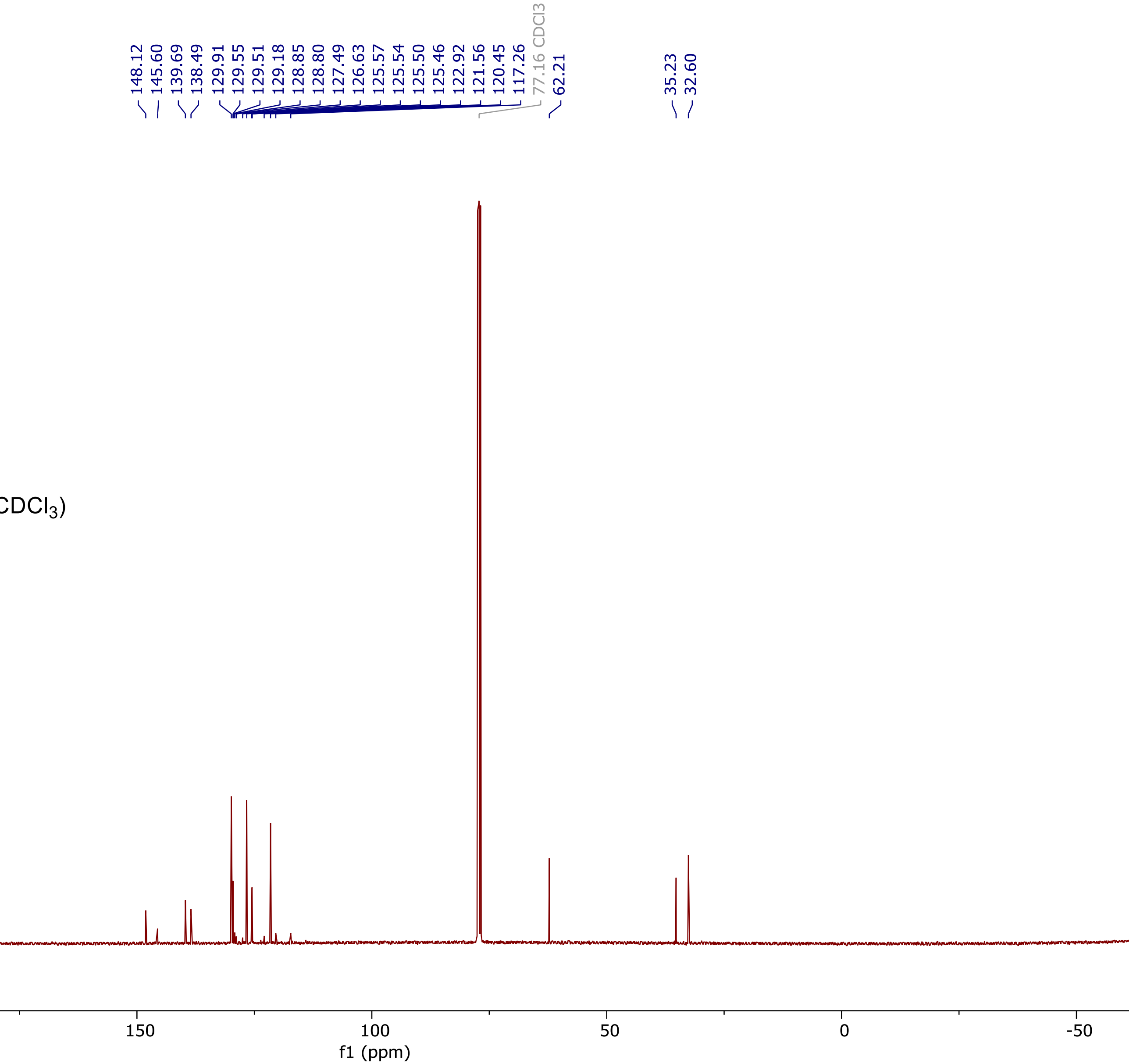

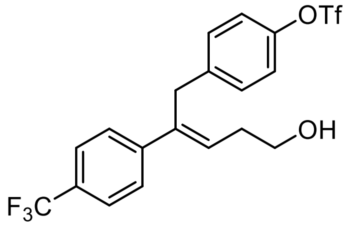

14m

$^{19}F\{^1H\}$ NMR (282 MHz, 298 K, $CDCl_3$)

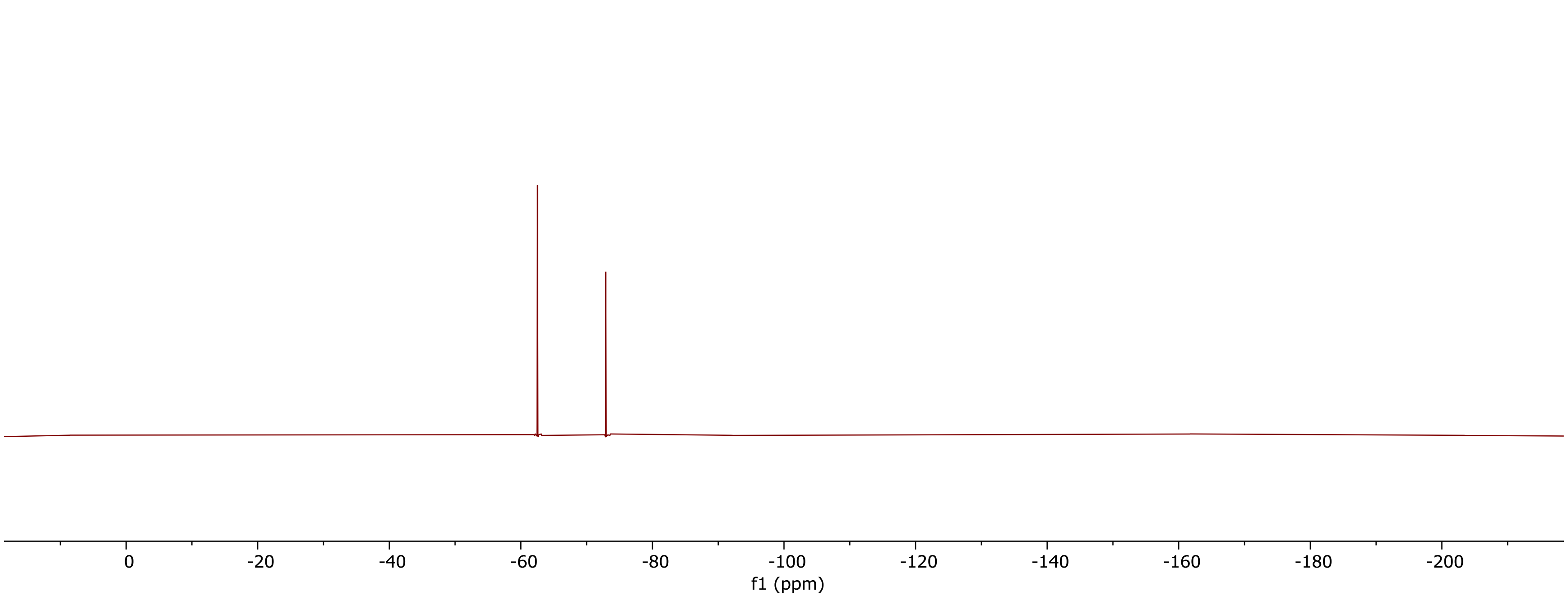

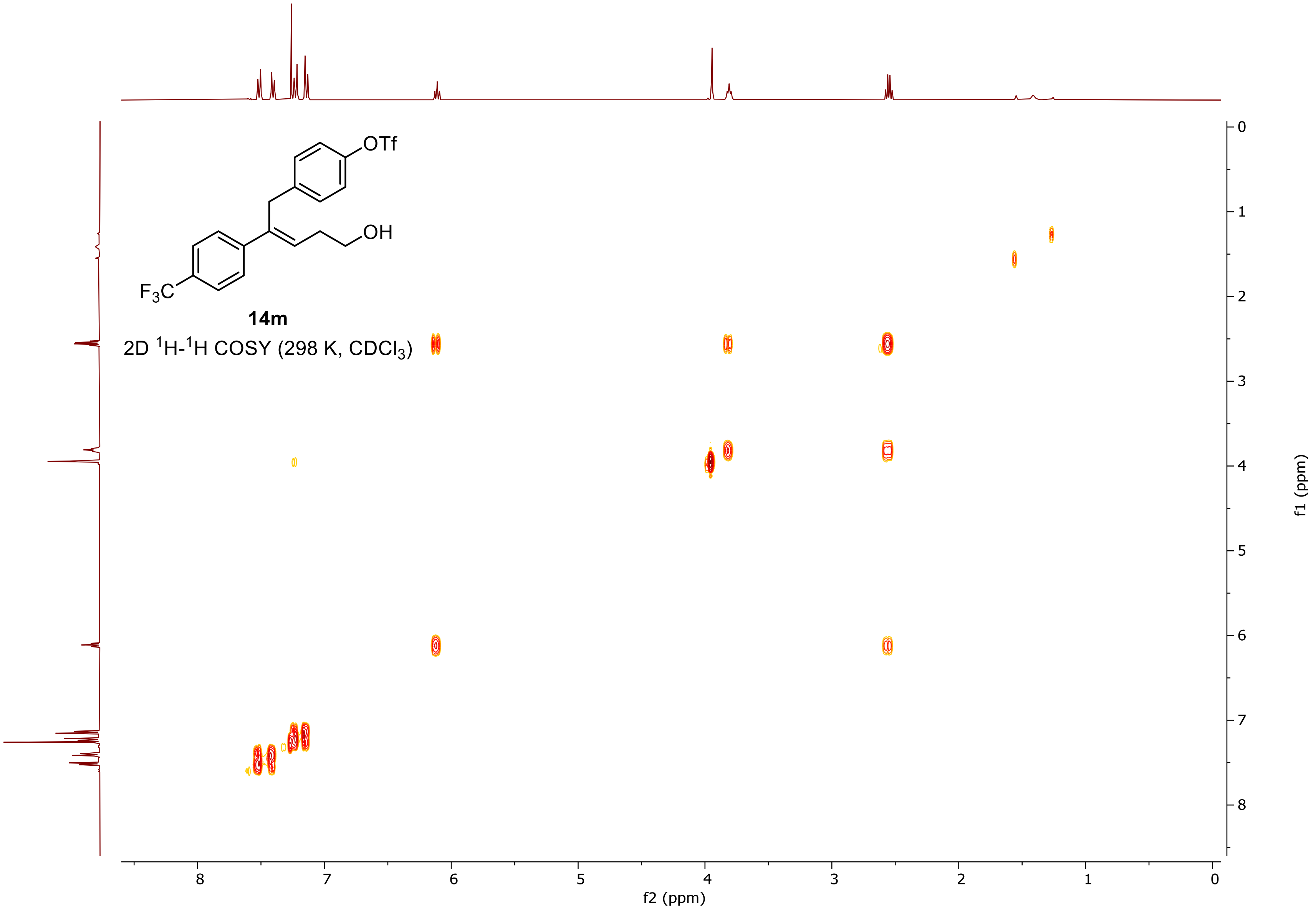
OTf
OH
F3C
14m
2D 1H-1H COSY (298 K, CDCl3)
f1 (ppm)
f2 (ppm)

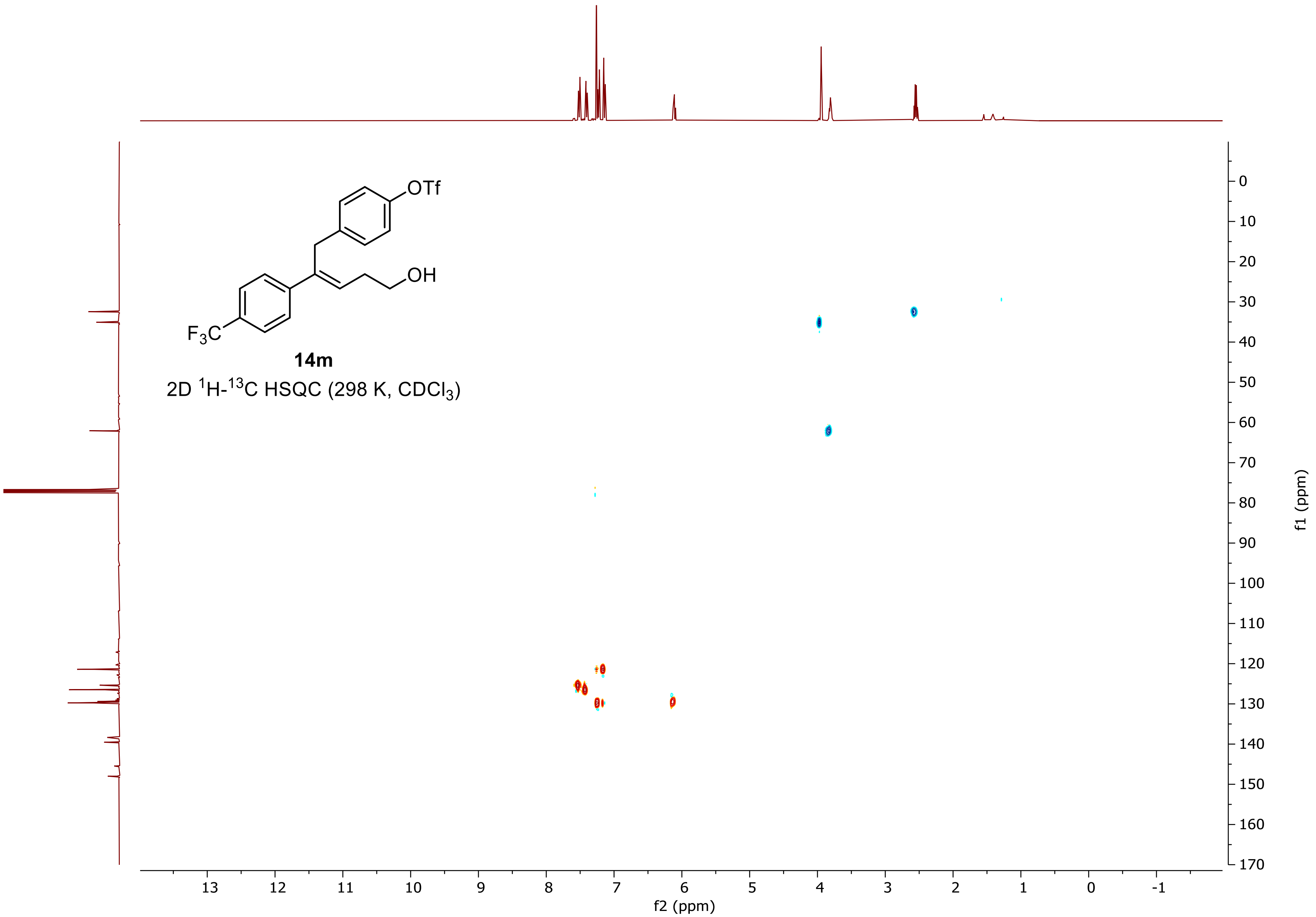

14m

2D ^{1}H-^{13}C HSQC (298 K, $CDCl_3$)

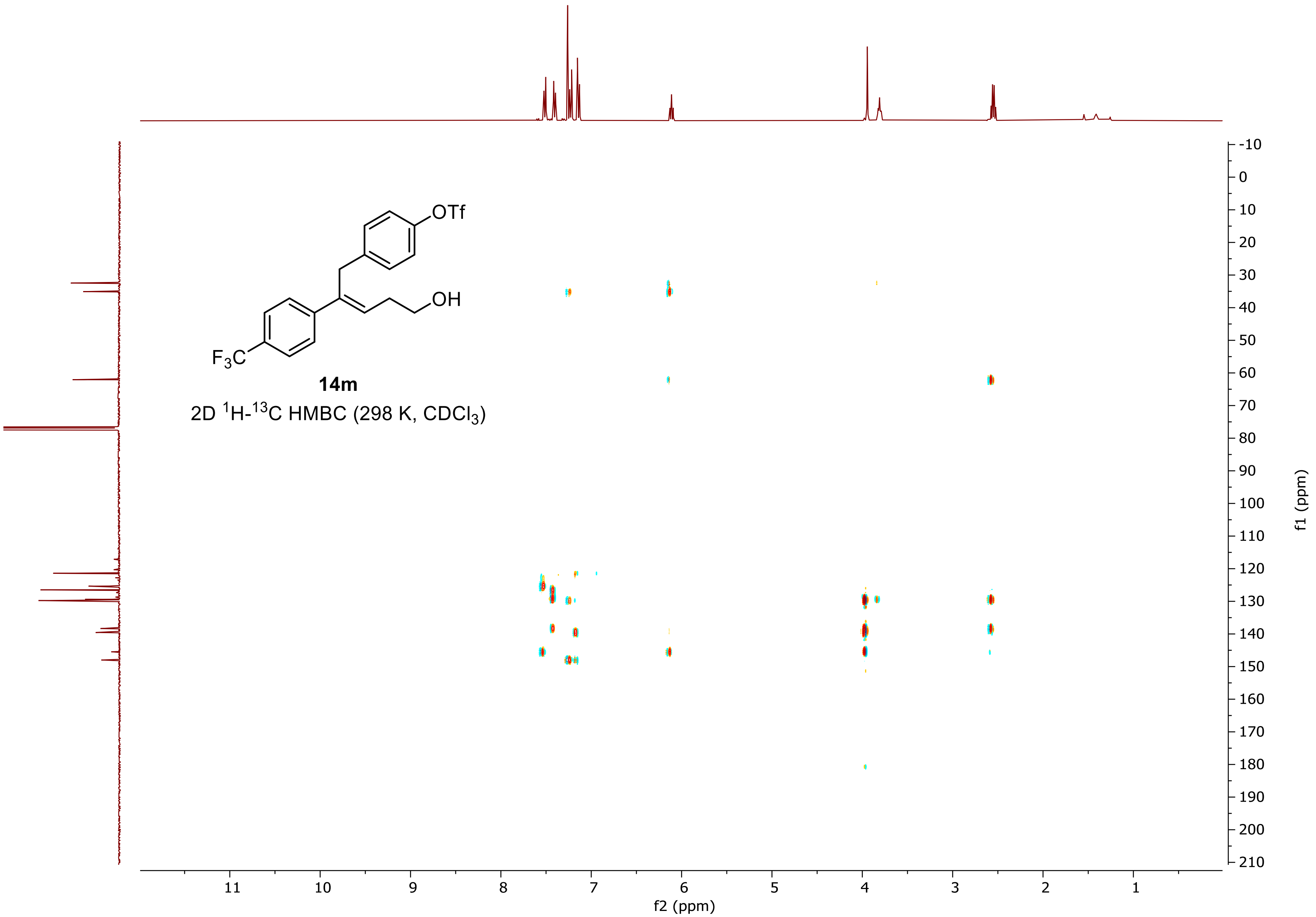
OTf
OH
F3C
14m
2D 1H-13C HMBC (298 K, CDCl3)
f1 (ppm)
f2 (ppm)

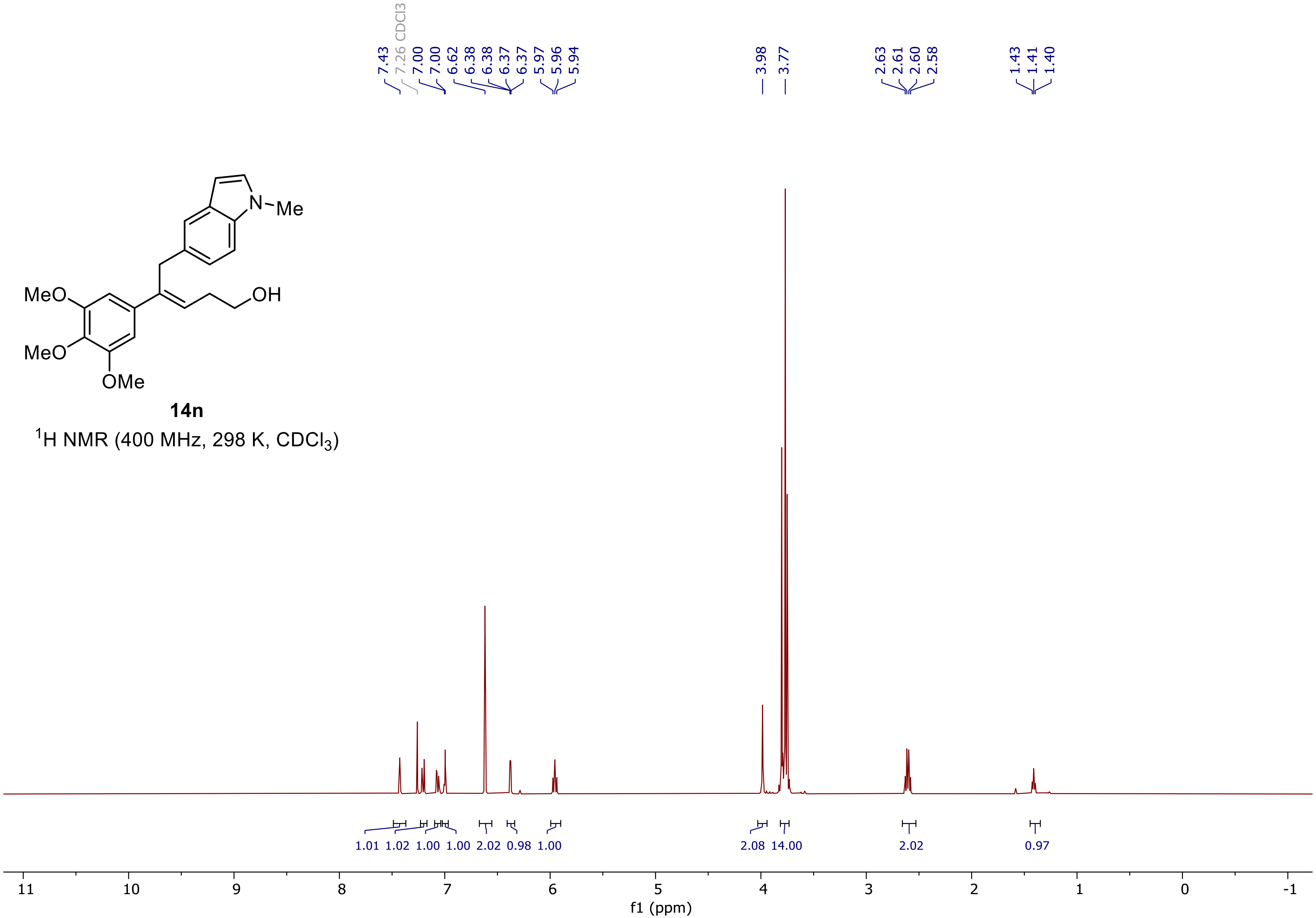

14n

^{1}H NMR (400 MHz, 298 K, $CDCl_3$)

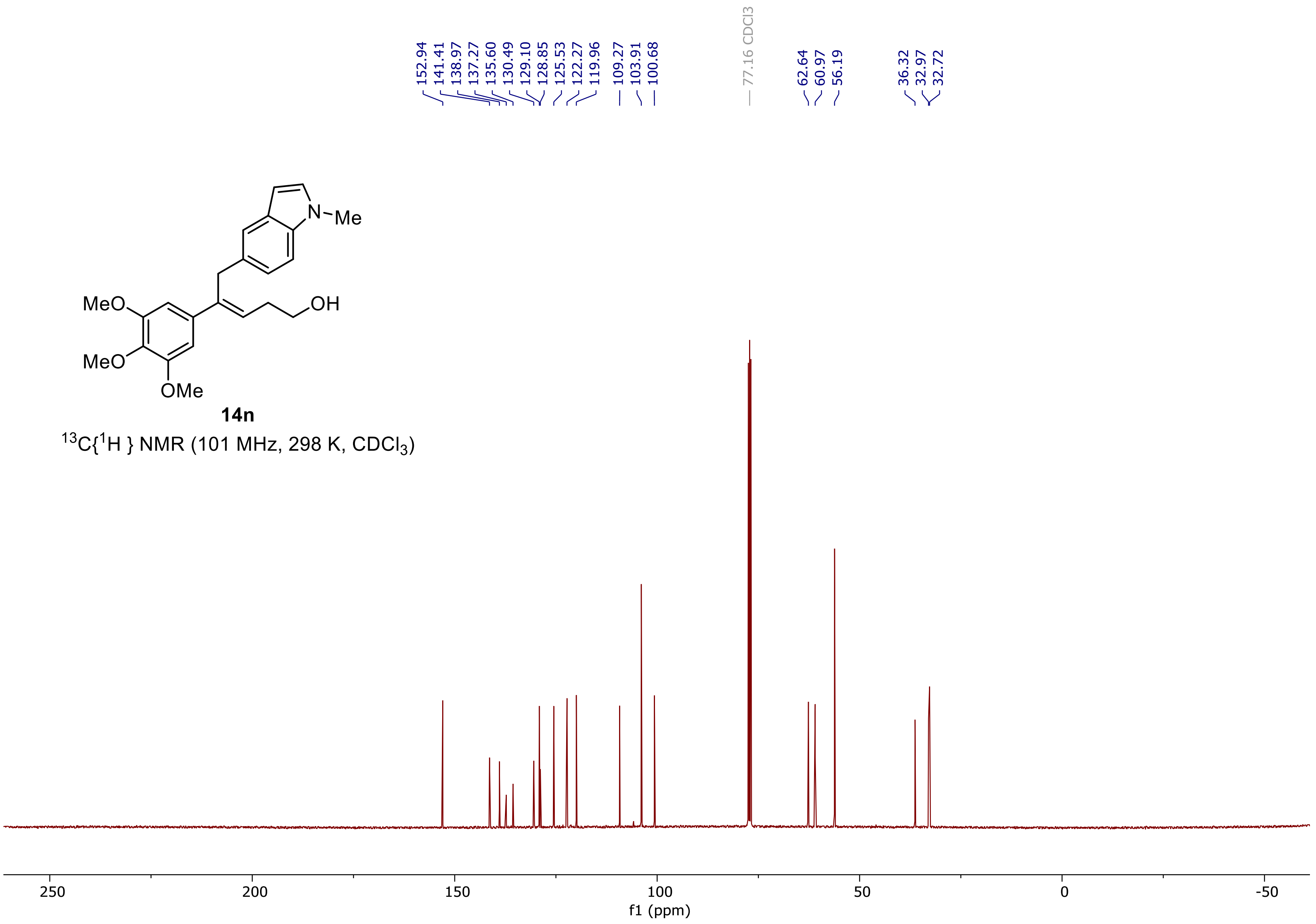

14n

$^{13}C\{^{1}H\}$ NMR (101 MHz, 298 K, $CDCl_3$)

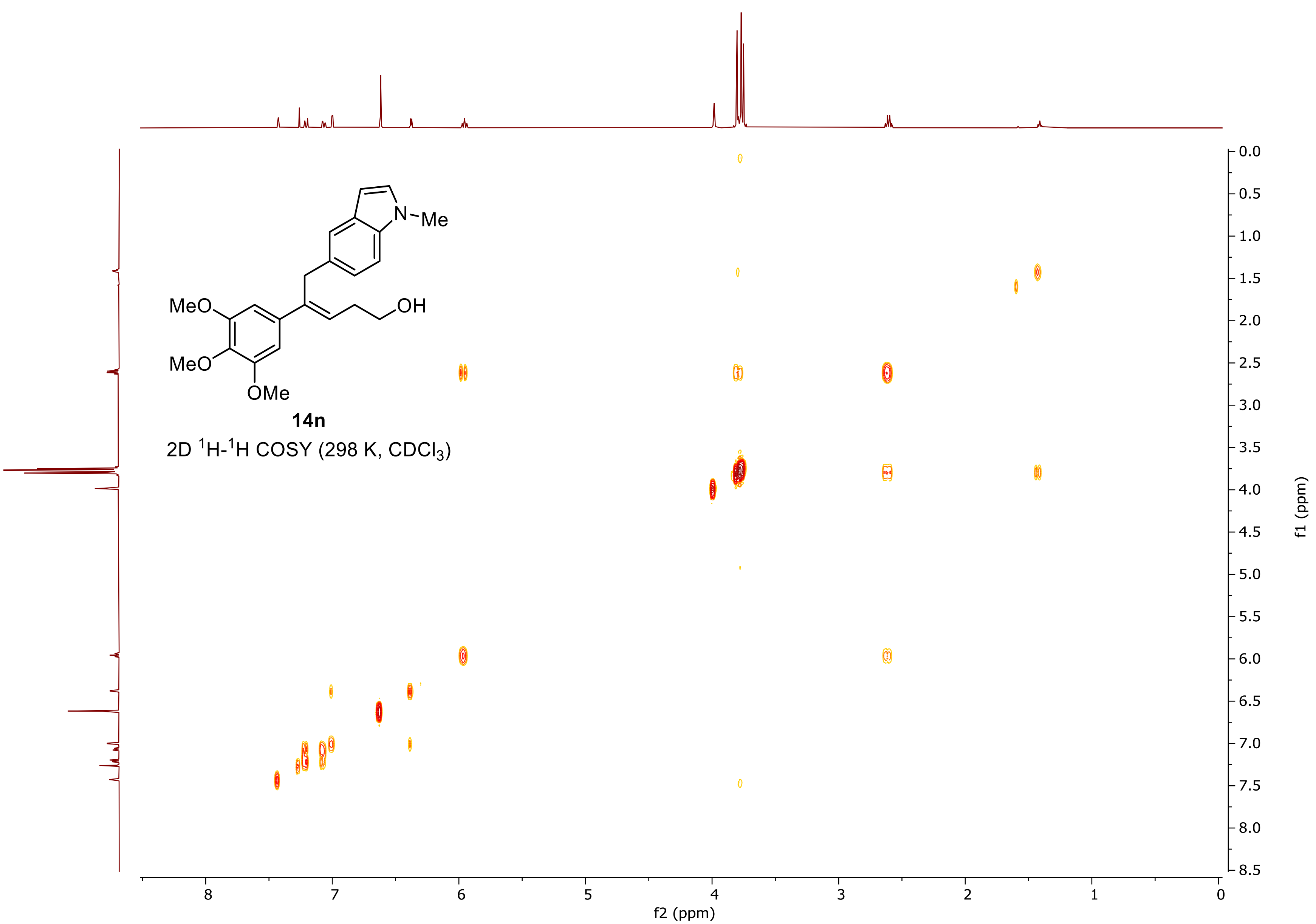

14n

2D ^{1}H-^{1}H COSY (298 K, $CDCl_3$)

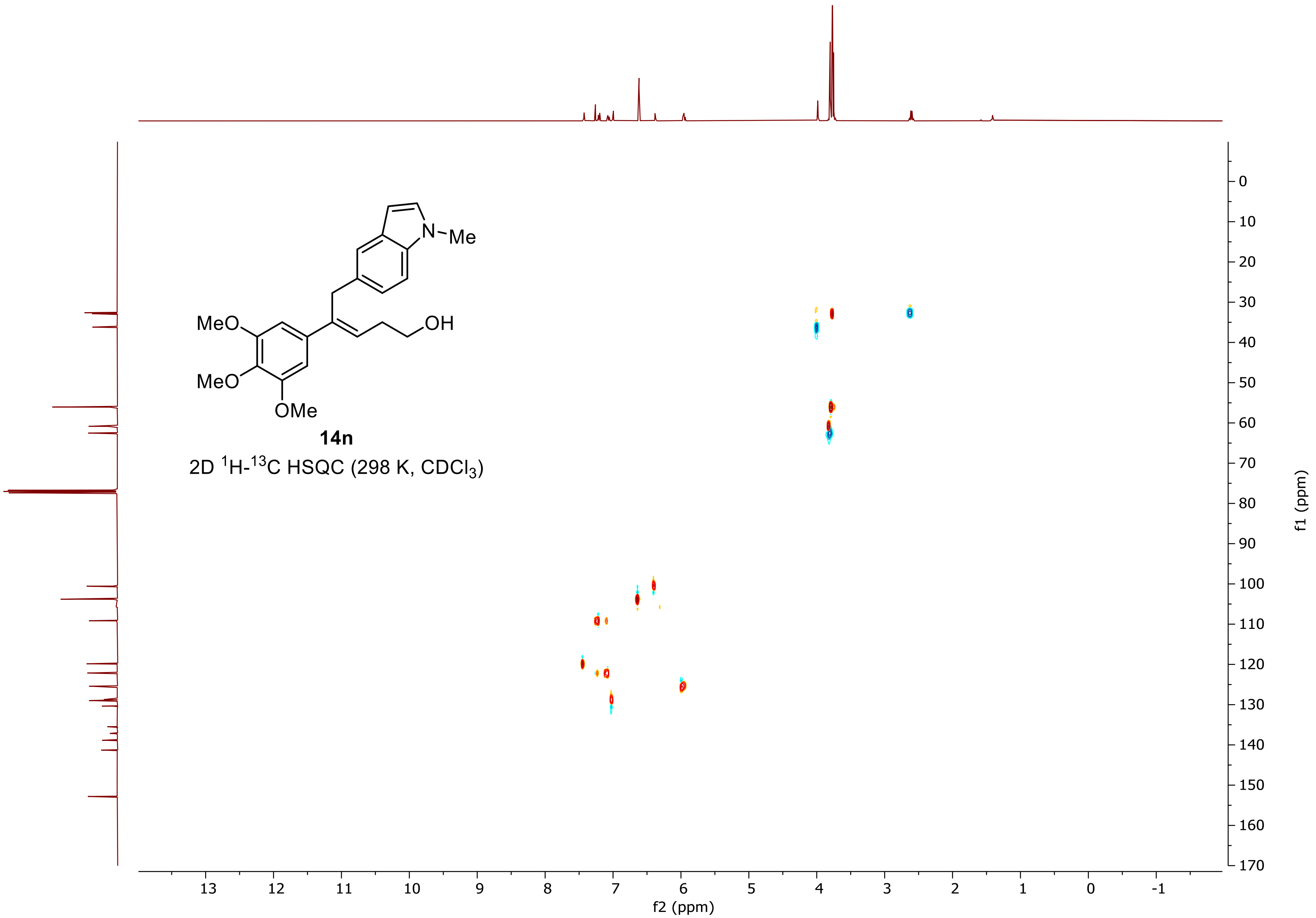
MeO
MeO
OMe
N
Me
OH
14n
2D ¹H-¹³C HSQC (298 K, CDCl₃)
f2 (ppm)
f1 (ppm)

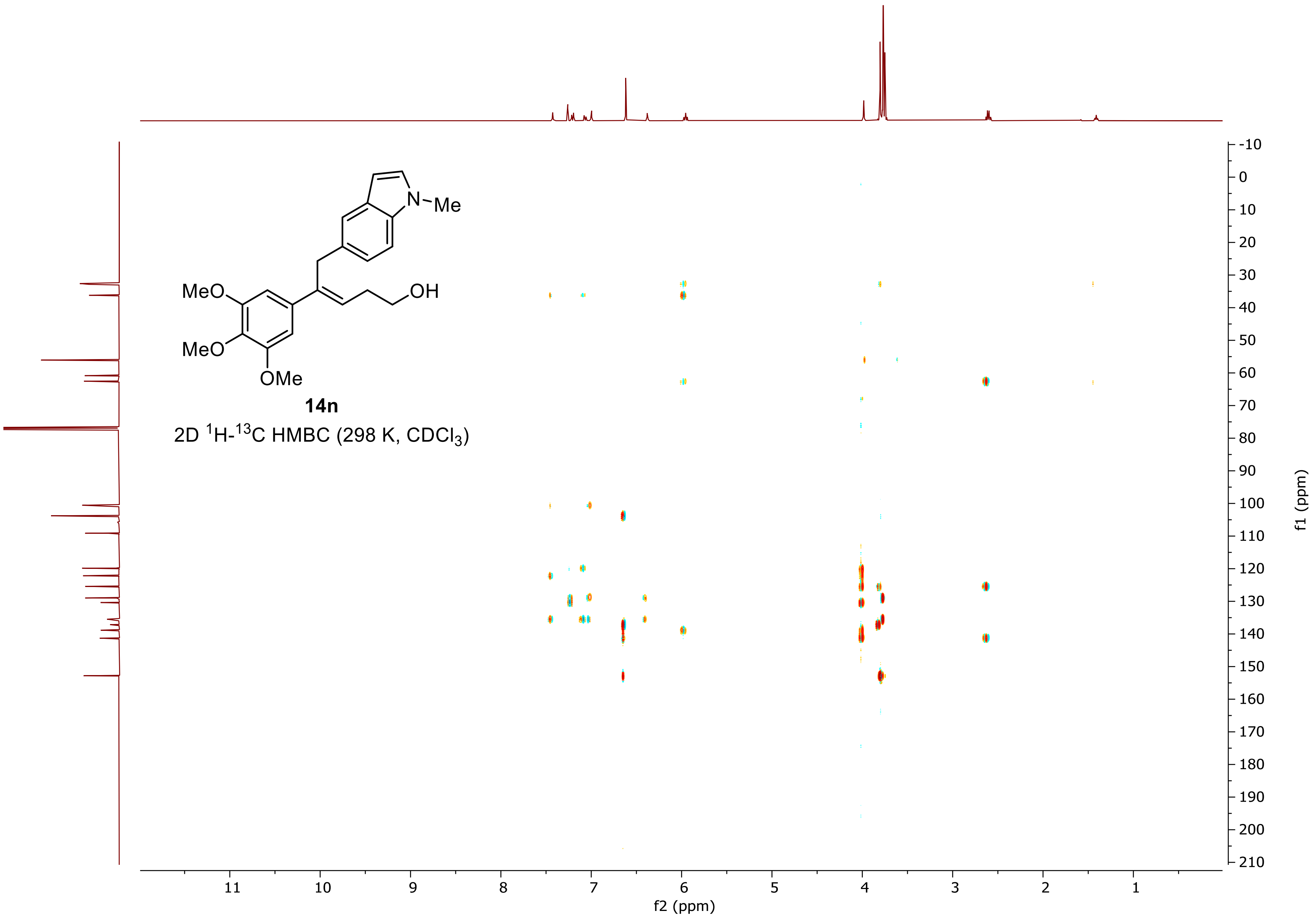

14n

2D ^{1}H-^{13}C HMBC (298 K, $CDCl_3$)

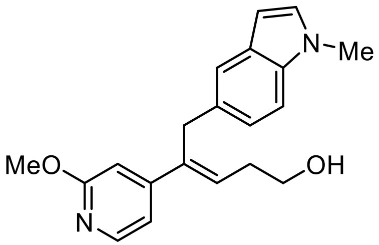

14o

^{1}H NMR (400 MHz, 298 K, $CDCl_3$)

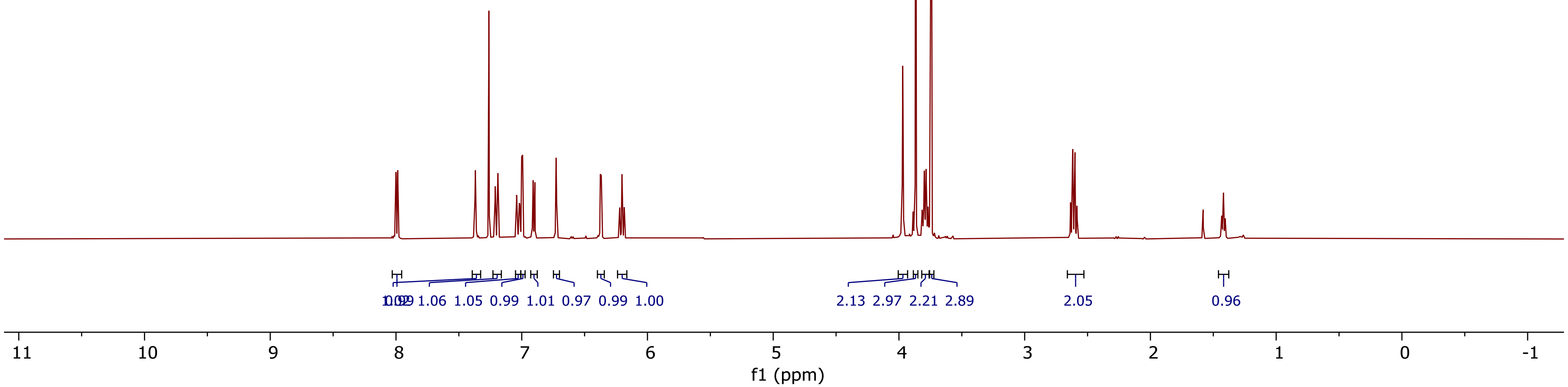

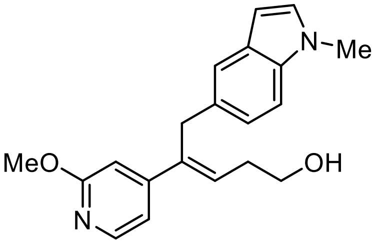

14o

$^{13}C\{^{1}H\}$ NMR (101 MHz, 298 K, $CDCl_3$)

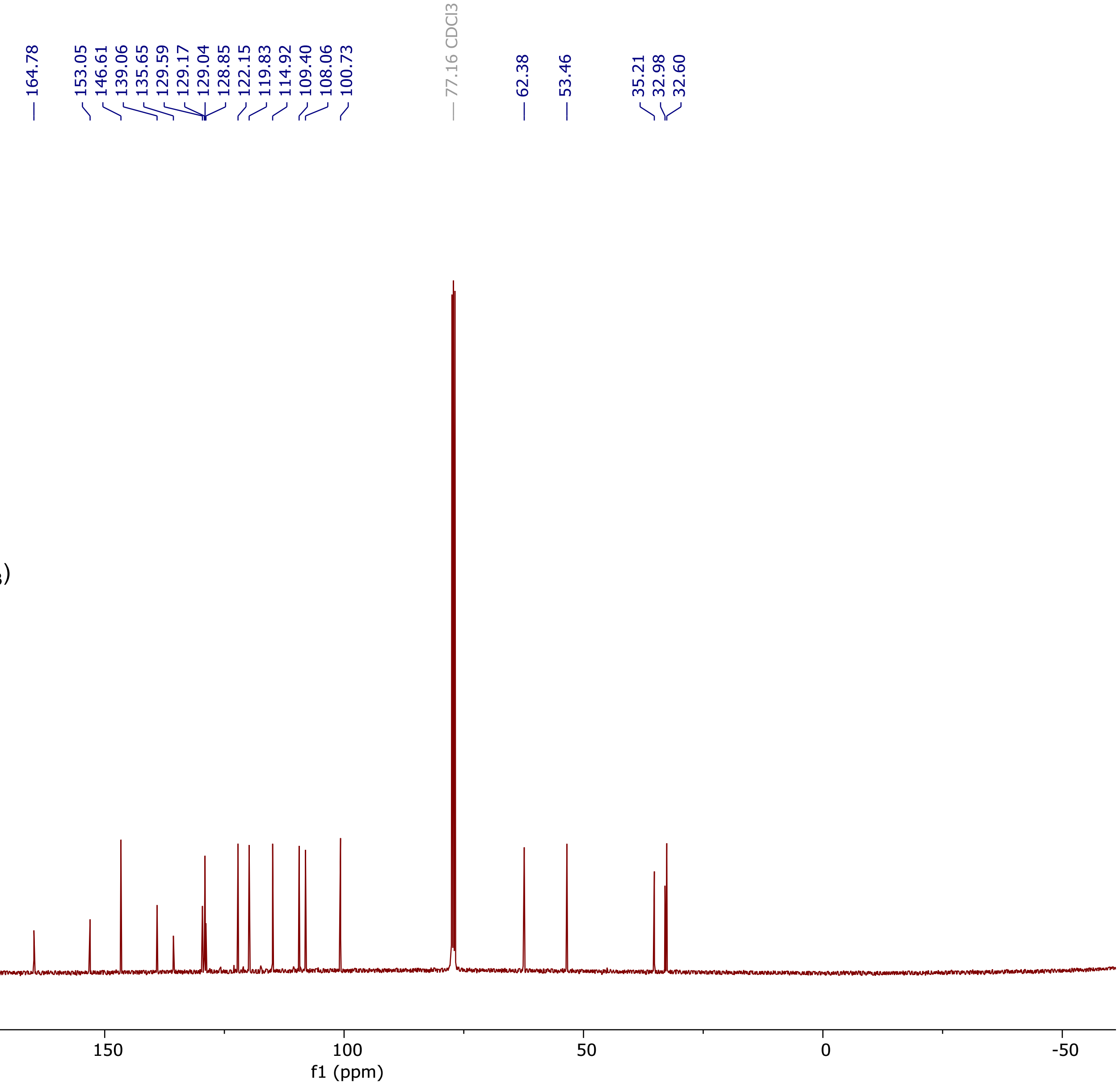

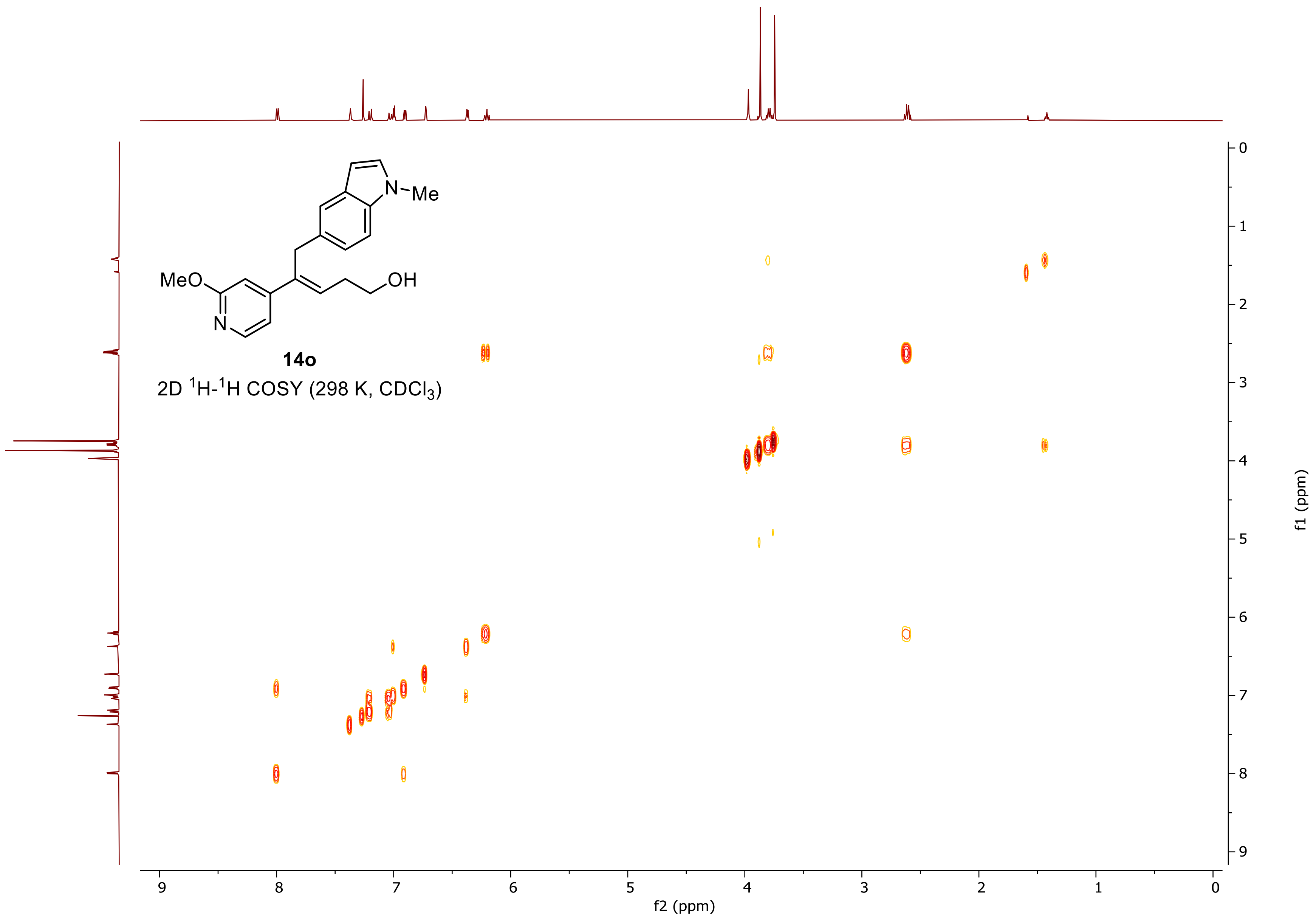

14o

2D ^{1}H-^{1}H COSY (298 K, $CDCl_3$)

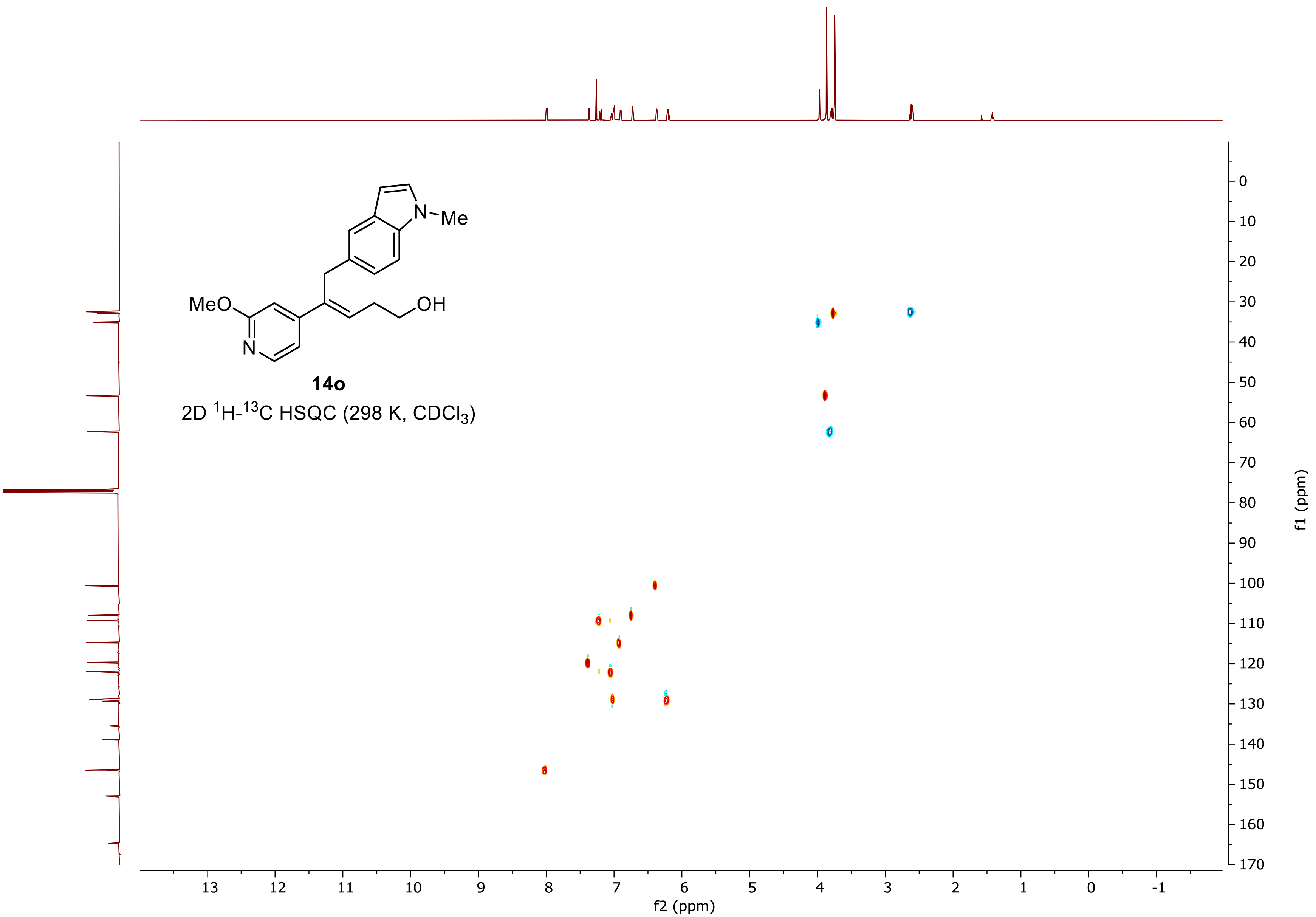
MeO
N
N–Me
OH
14o
2D 1H-13C HSQC (298 K, CDCl3)
f2 (ppm)
f1 (ppm)
13
12
11
10
9
8
7
6
5
4
3
2
1
0
-1
0
10
20
30
40
50
60
70
80
90
100
110
120
130
140
150
160
170

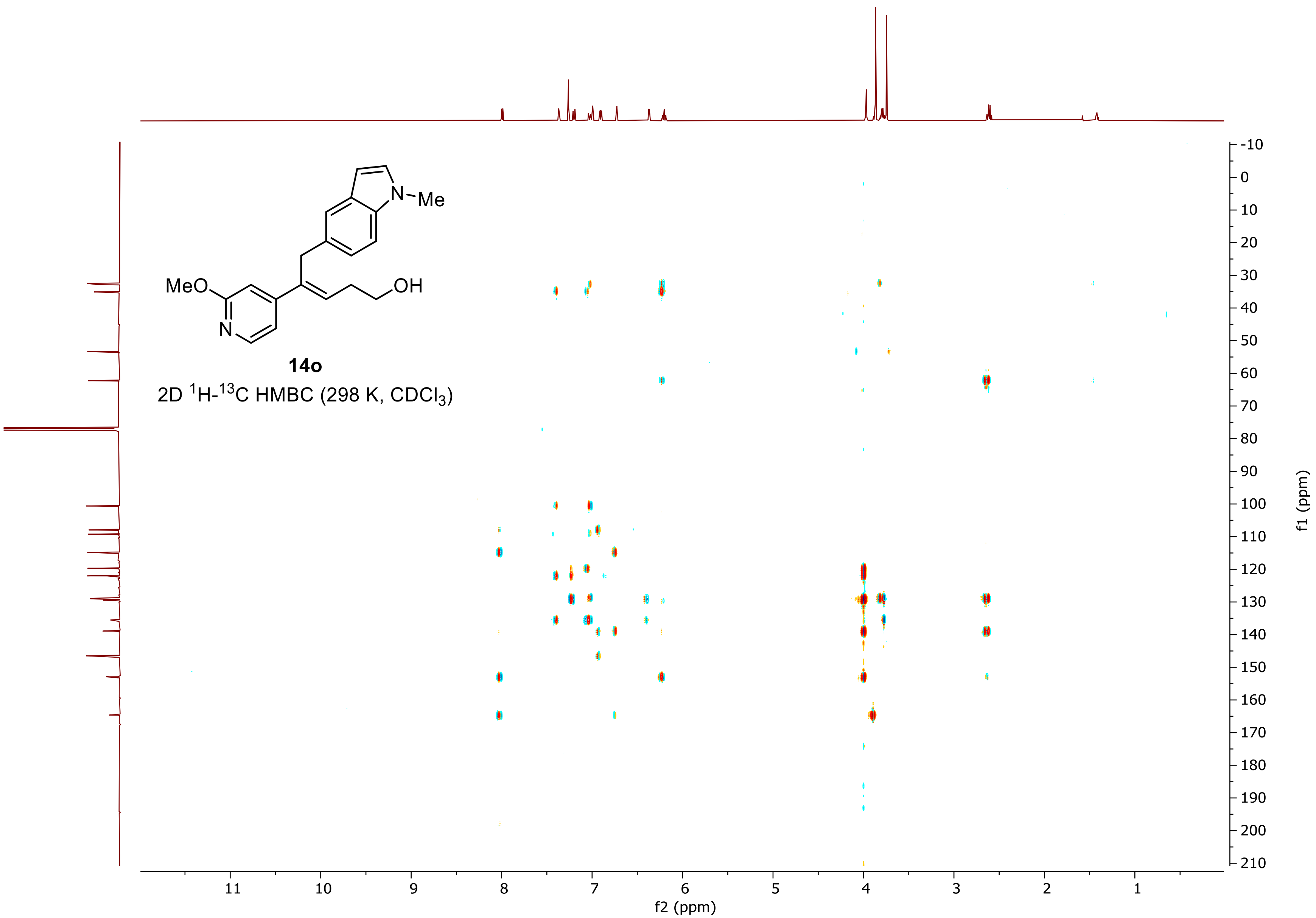
MeO
N
N-Me
OH
14o
2D ¹H-¹³C HMBC (298 K, CDCl₃)
f2 (ppm)
f1 (ppm)

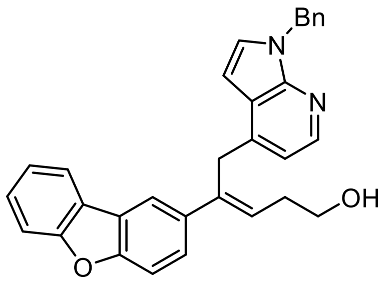

14p

^{1}H NMR (400 MHz, 298 K, $CDCl_3$)

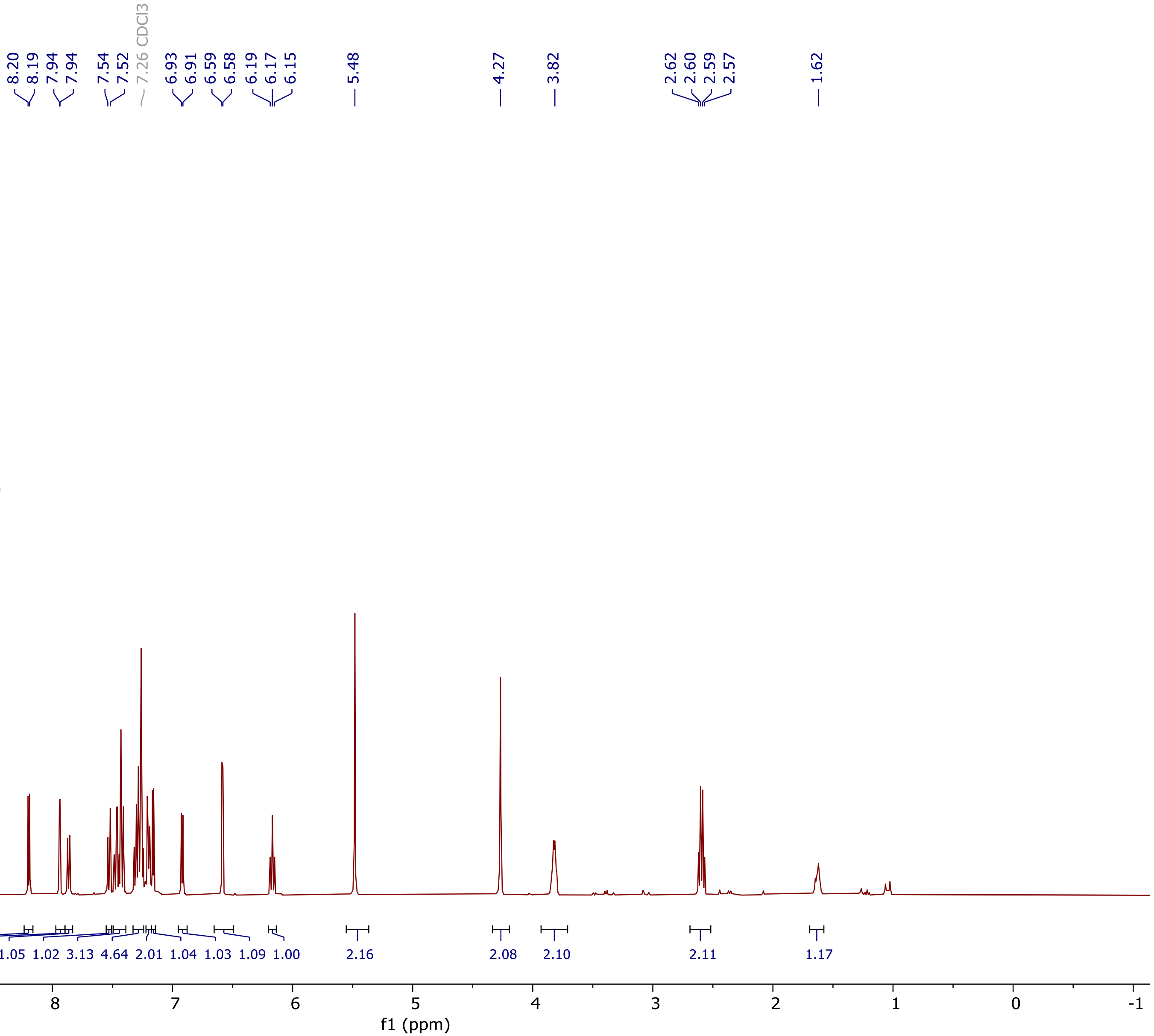

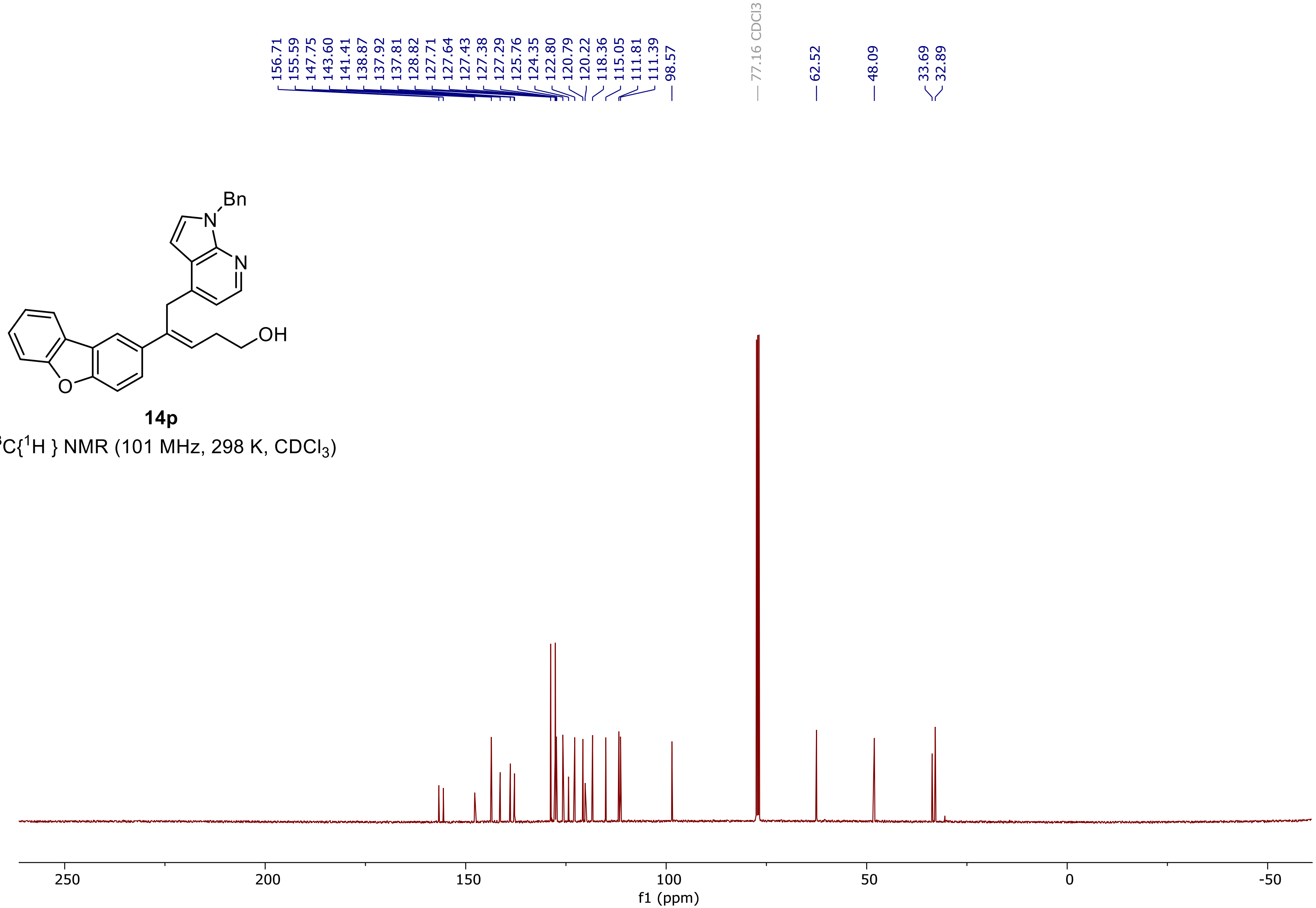

14p

$^{13}C\{^{1}H\}$ NMR (101 MHz, 298 K, $CDCl_3$)

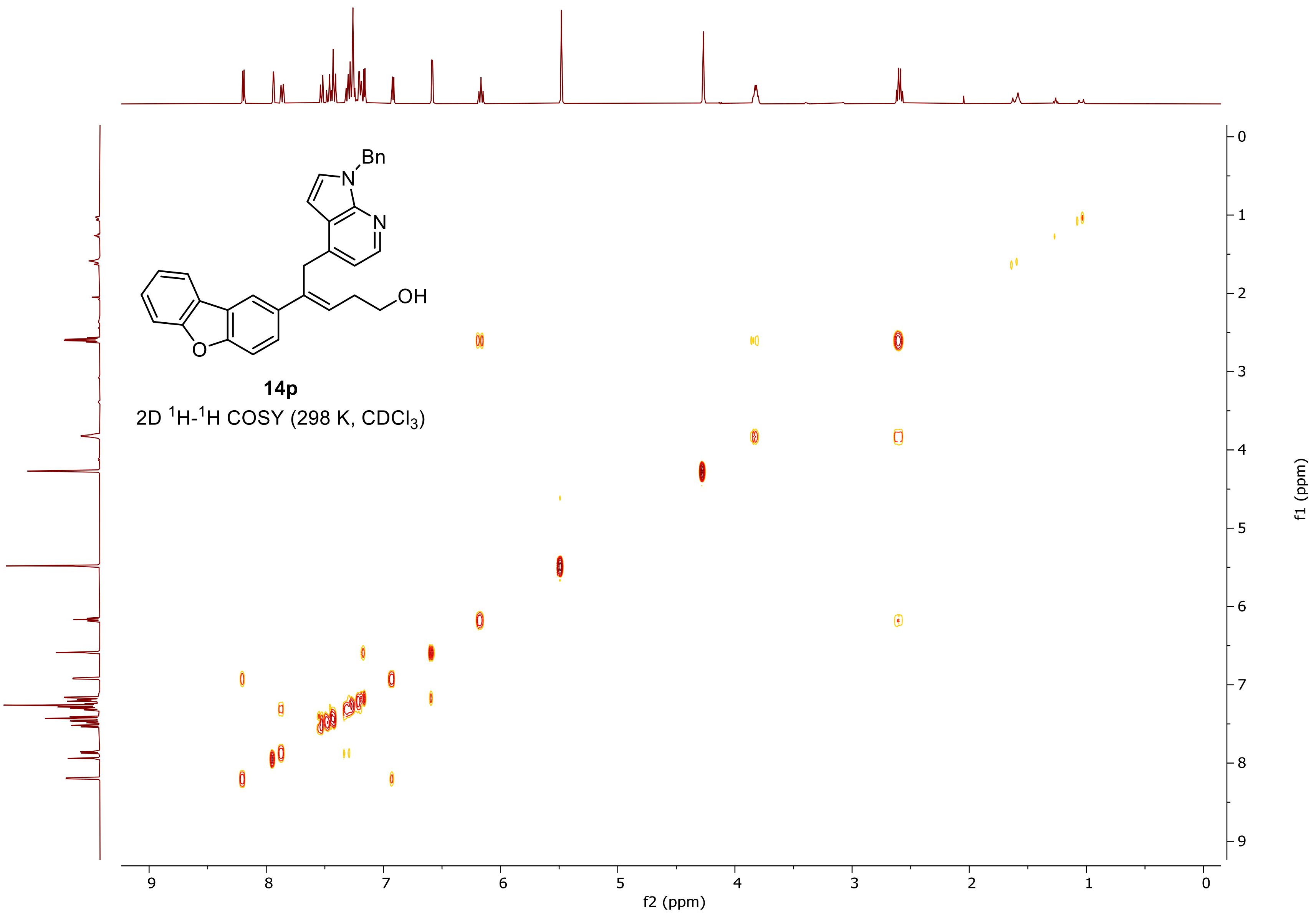

14p

2D ^{1}H-^{1}H COSY (298 K, $CDCl_3$)

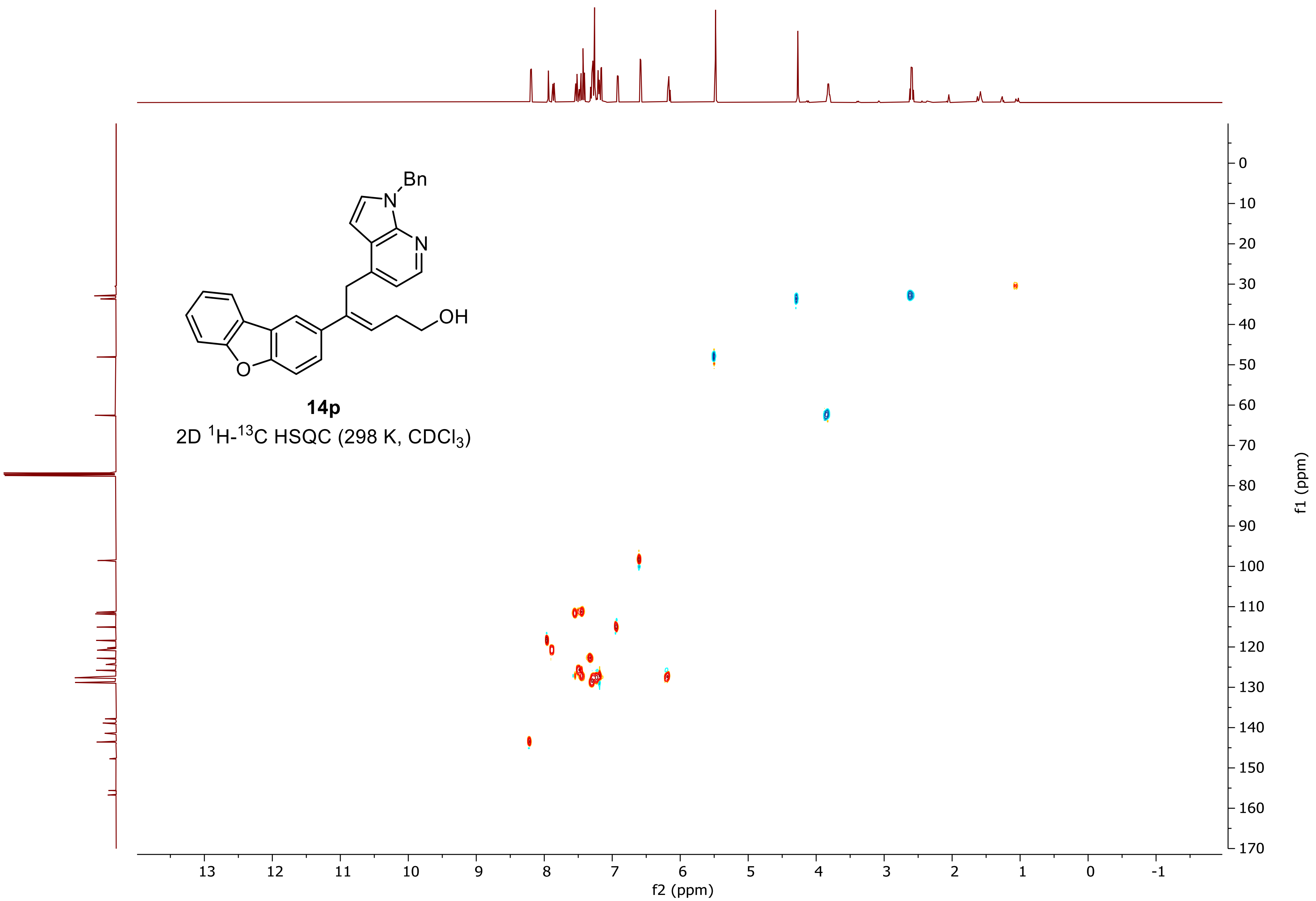
Bn
N
N
OH
O
14p
2D 1H-13C HSQC (298 K, CDCl3)
f2 (ppm)
f1 (ppm)

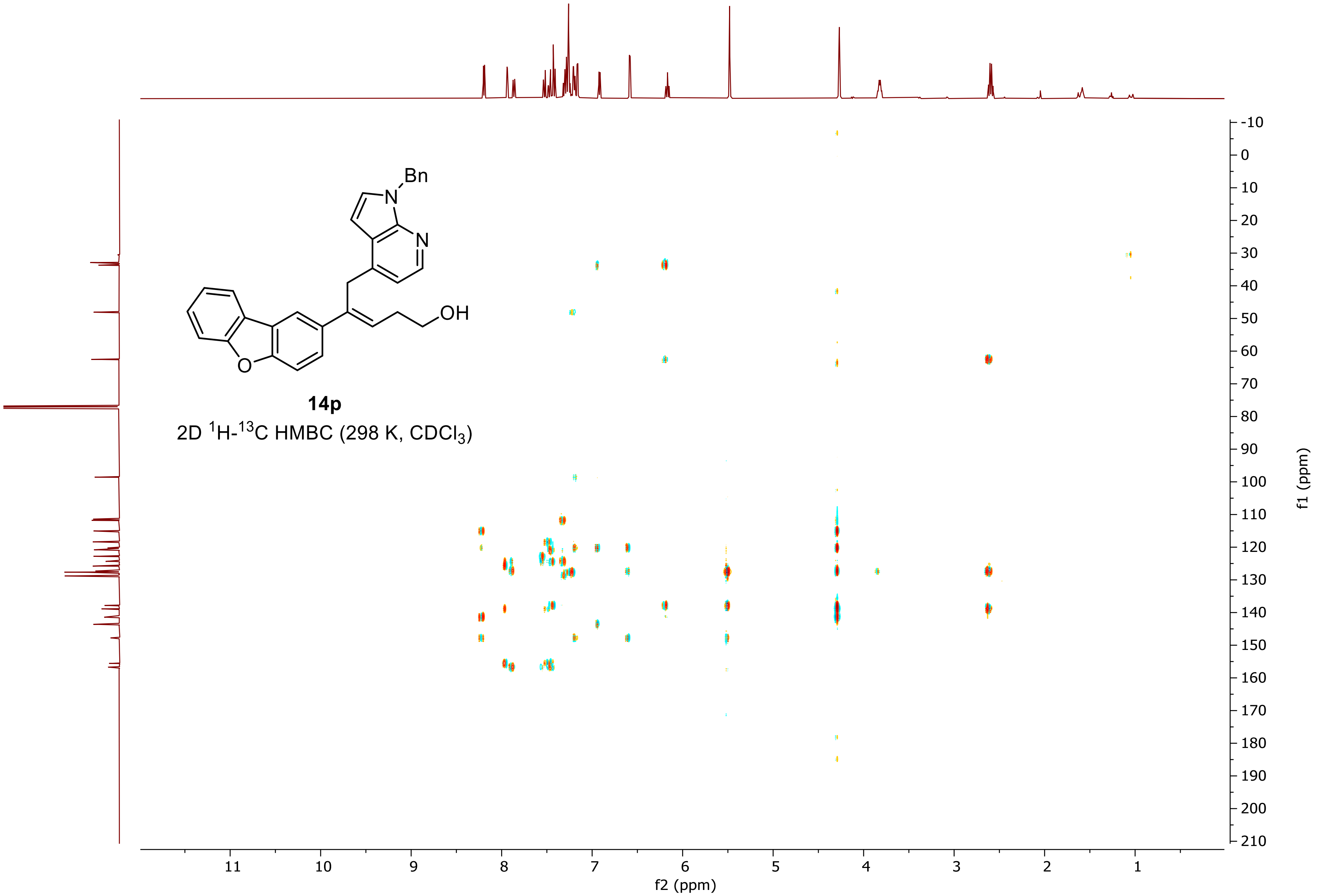
Bn
N
N
OH
O
14p
2D 1H-13C HMBC (298 K, CDCl3)
f1 (ppm)
f2 (ppm)
-10
0
10
20
30
40
50
60
70
80
90
100
110
120
130
140
150
160
170
180
190
200
210
11
10
9
8
7
6
5
4
3
2
1

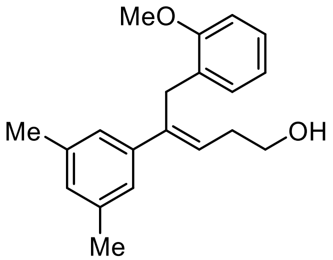

14q

^1H NMR (400 MHz, 298 K, $CDCl_3$)

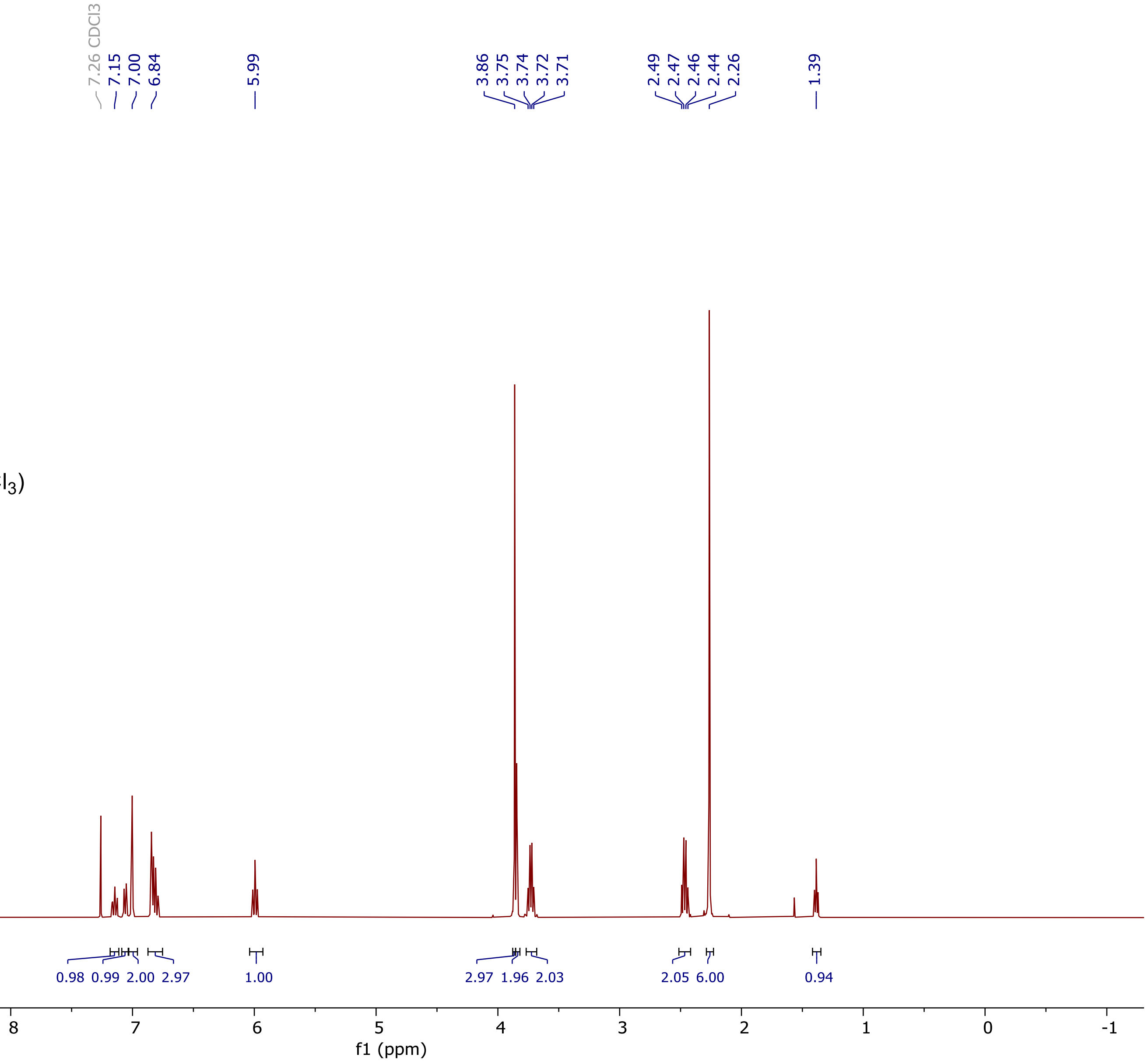

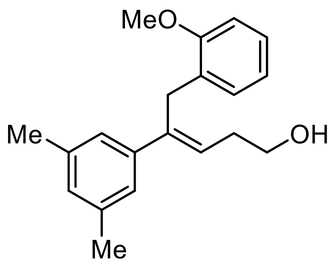

14q

$^{13}C\{^{1}H\}$ NMR (101 MHz, 298 K, $CDCl_3$)

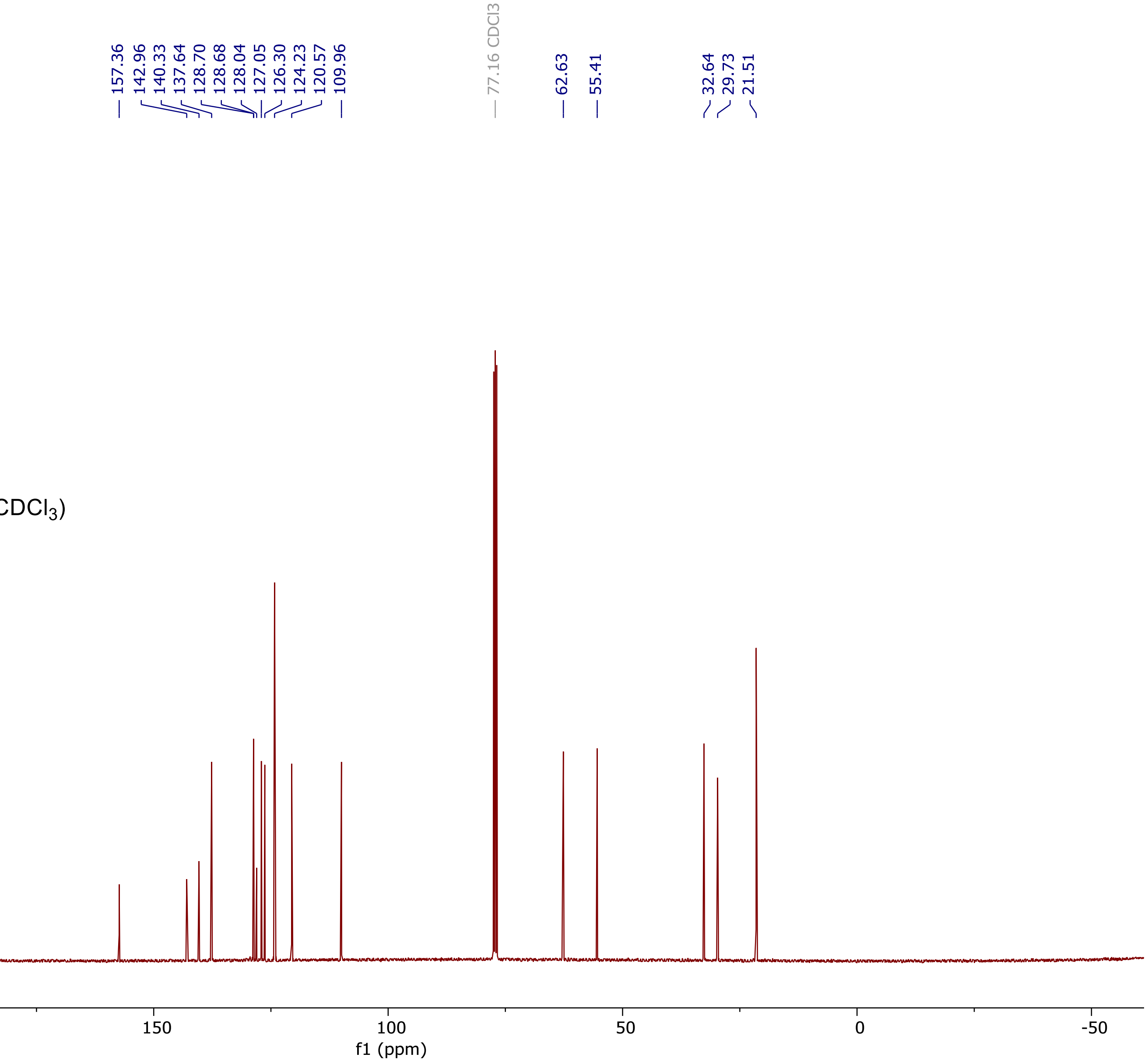

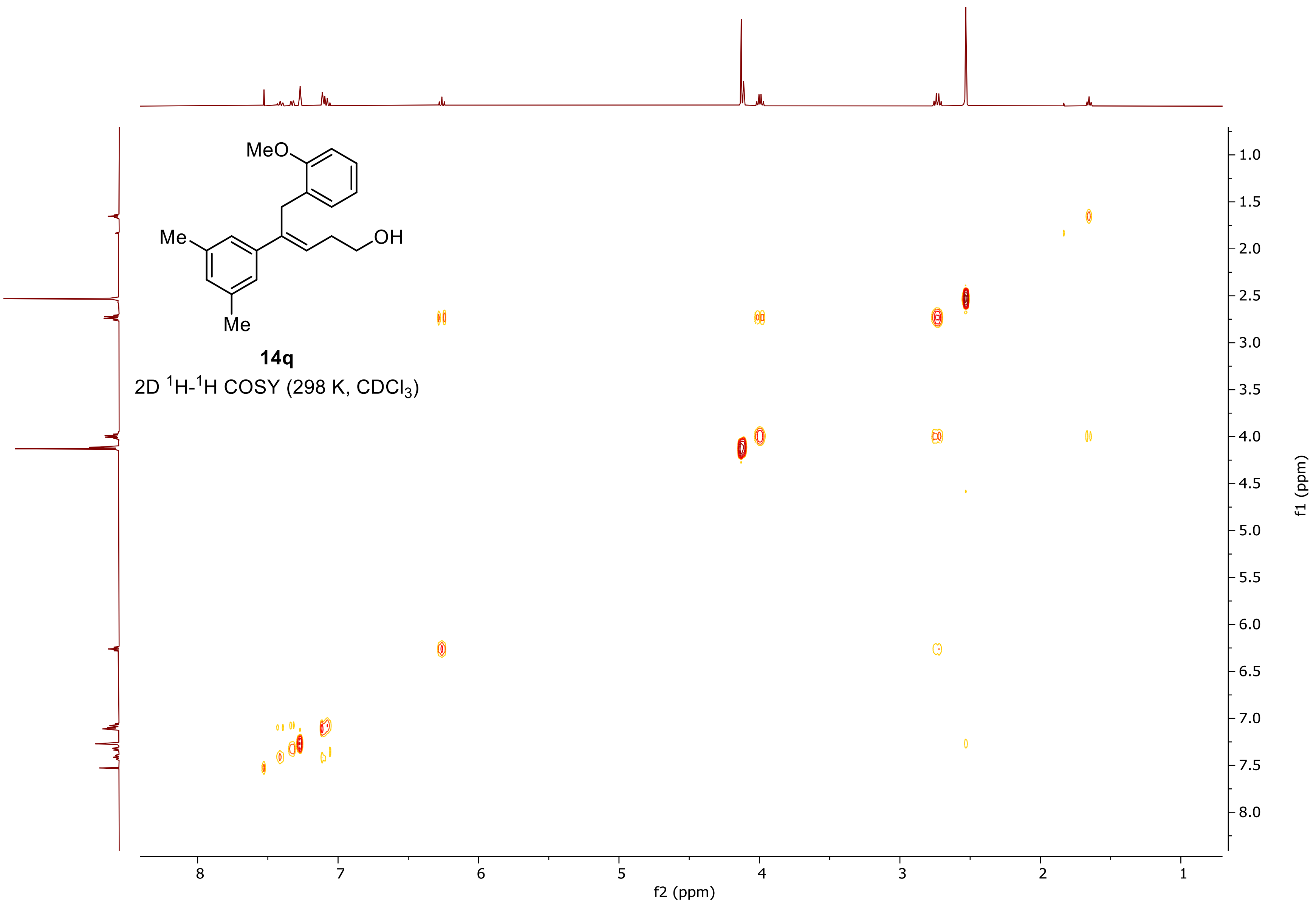

14q

2D ^{1}H-^{1}H COSY (298 K, $CDCl_3$)

14q

2D ^{1}H-^{13}C HSQC (298 K, $CDCl_3$)

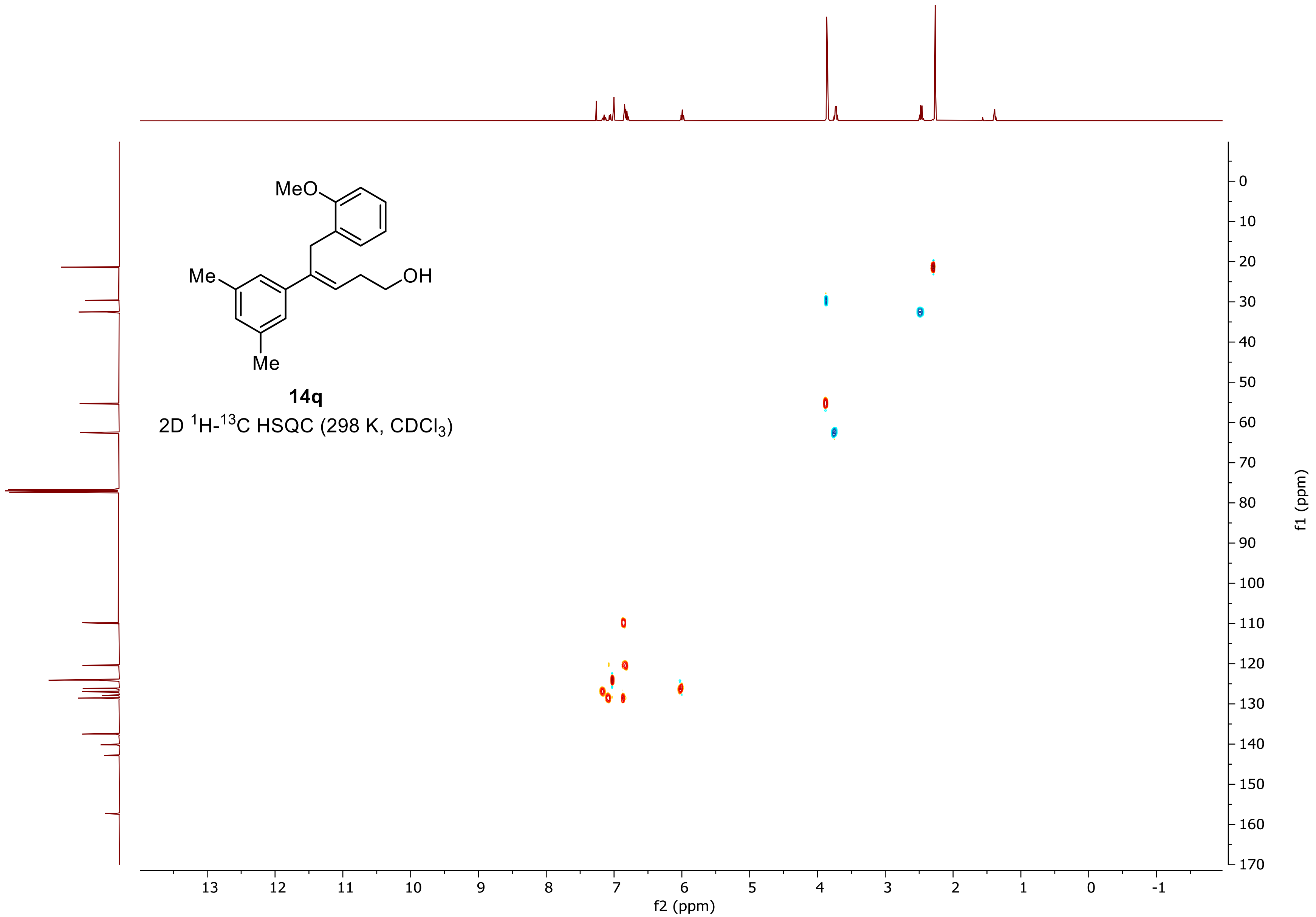

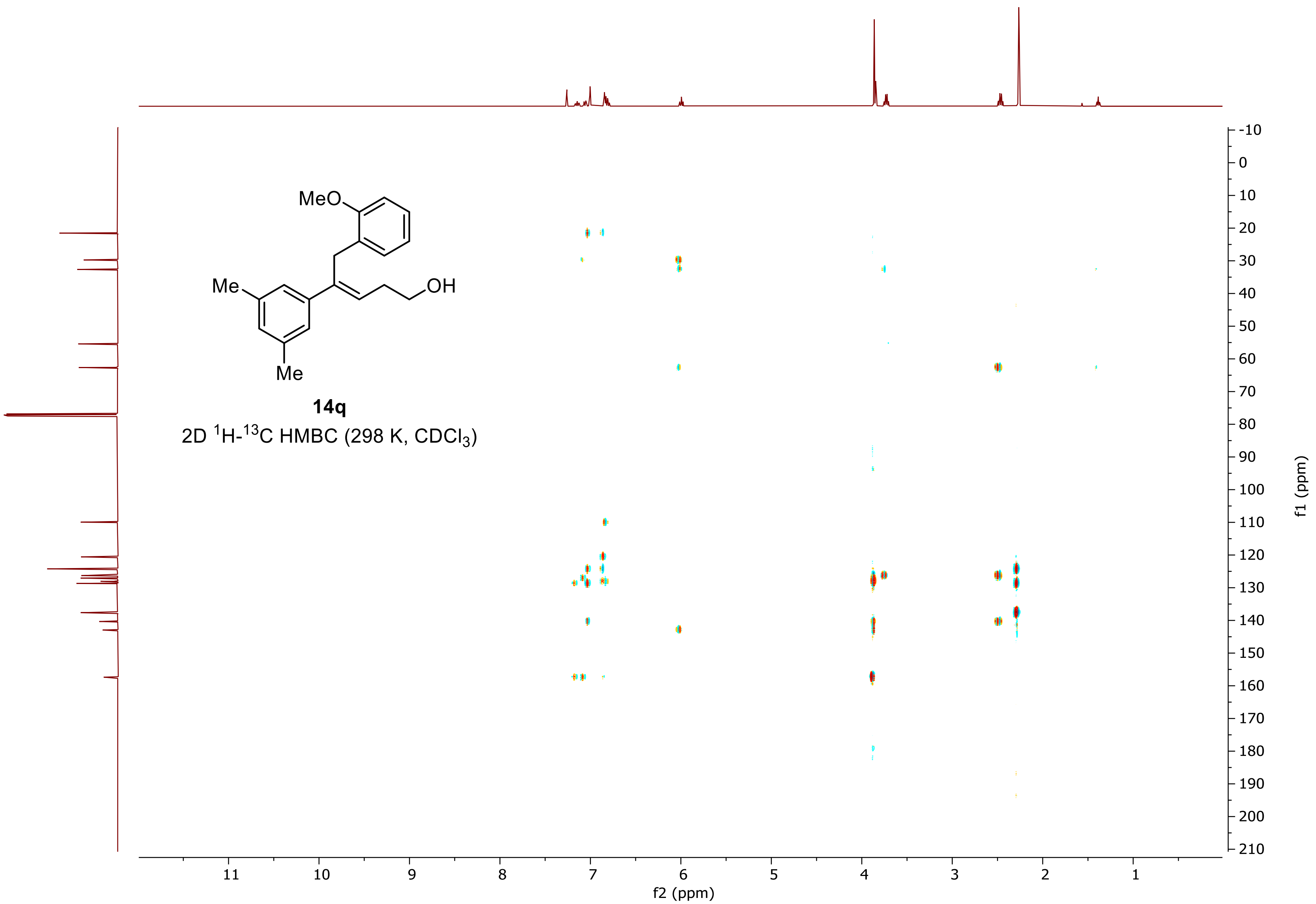

14q
2D ^{1}H-^{13}C HMBC (298 K, $CDCl_3$)

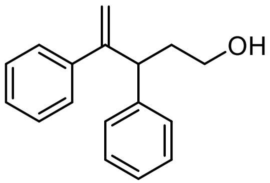

15a

^{1}H NMR (400 MHz, 298 K, $CDCl_3$)

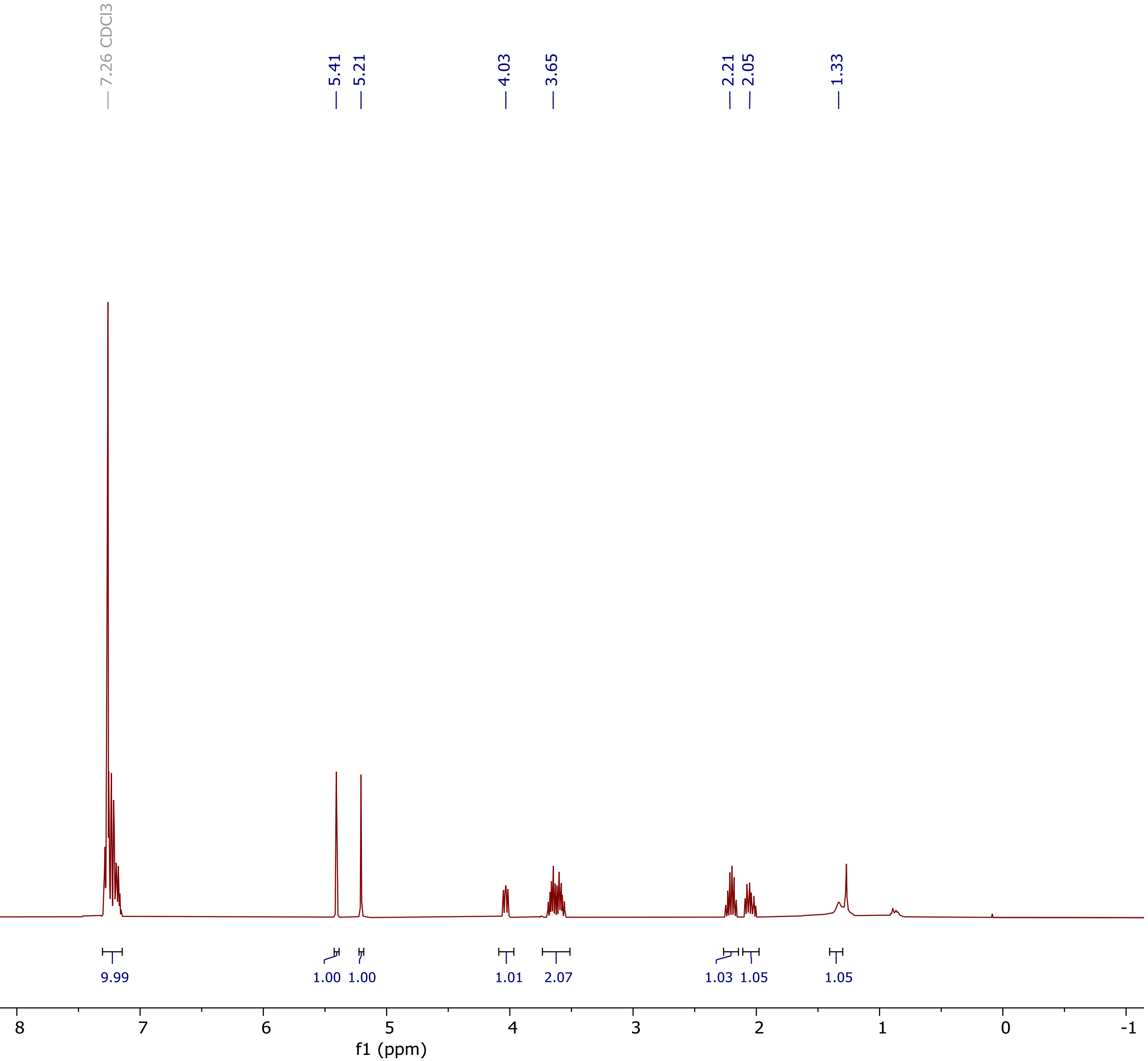

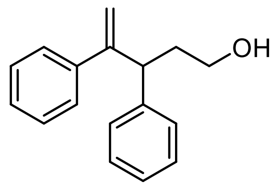

15a

$^{13}C\{^{1}H\}$ NMR (101 MHz, 298 K, $CDCl_3$)

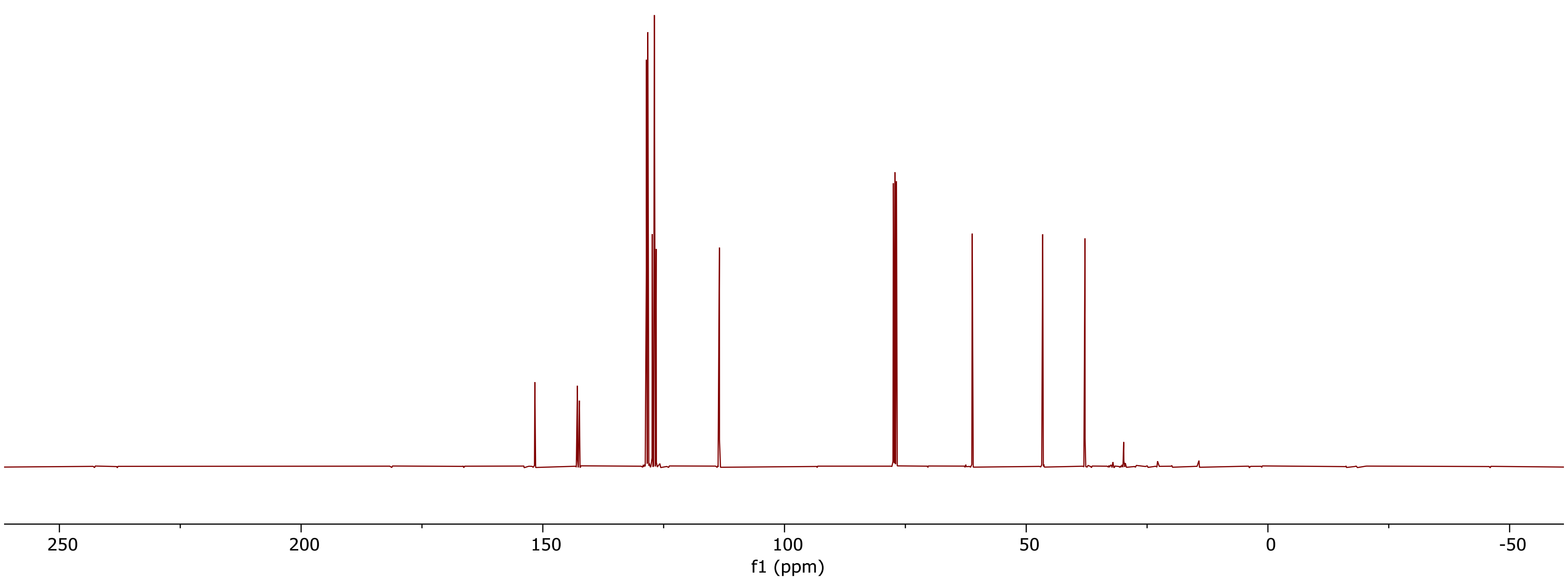

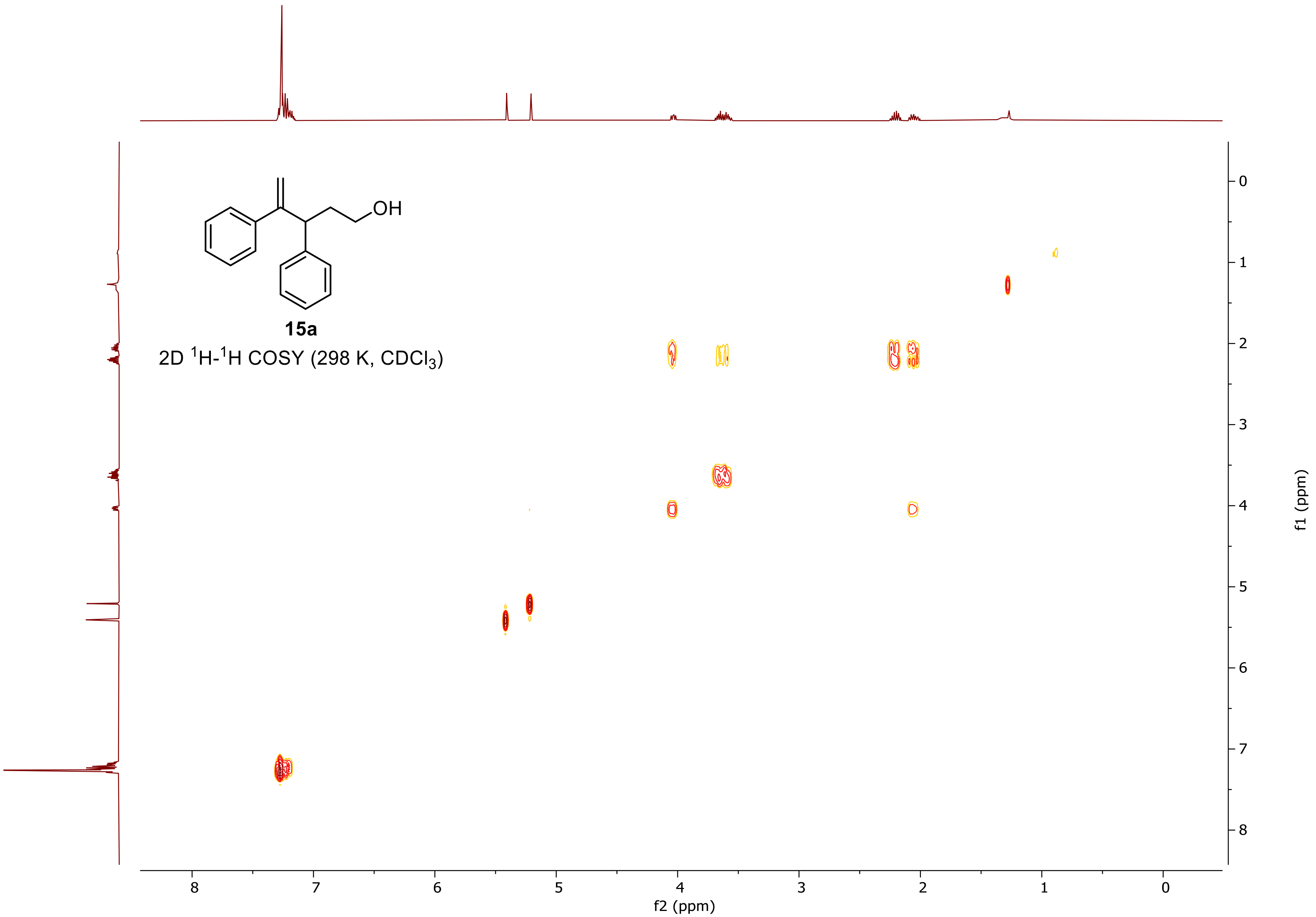
OH
15a
2D ¹H-¹H COSY (298 K, CDCl₃)
f2 (ppm)
f1 (ppm)
8
7
6
5
4
3
2
1
0

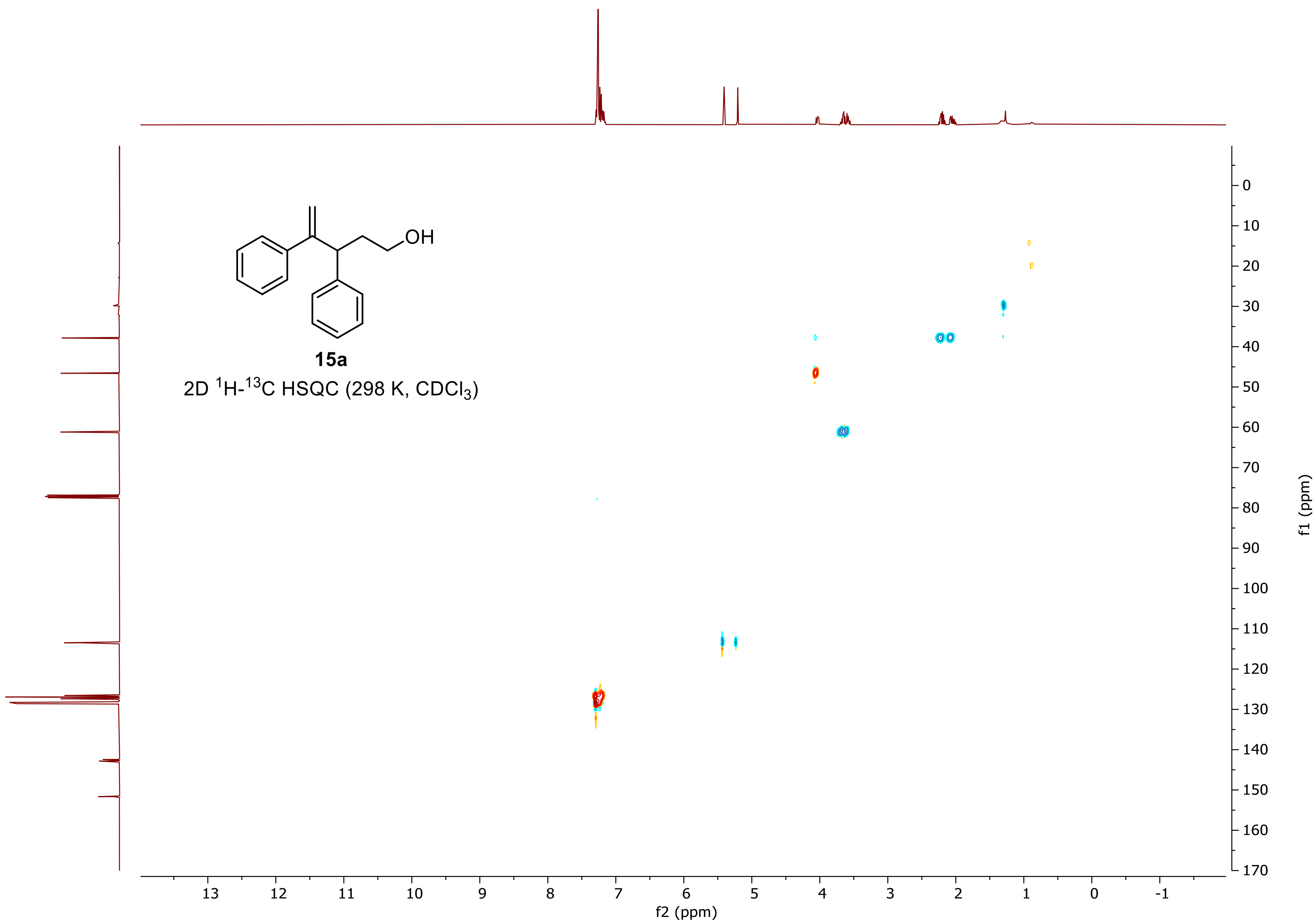

15a

2D ^{1}H-^{13}C HSQC (298 K, $CDCl_3$)

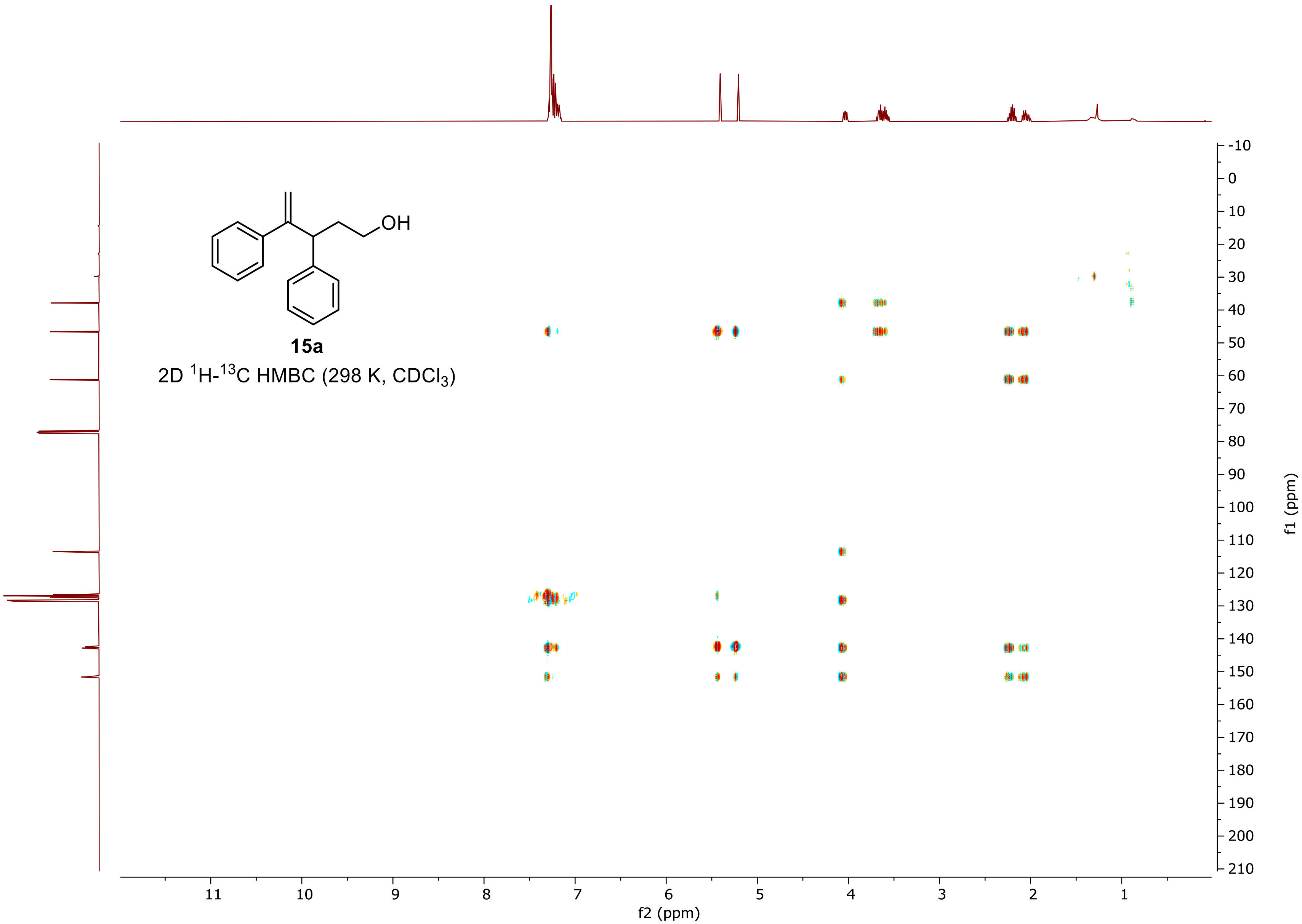
OH
15a
2D 1H-13C HMBC (298 K, CDCl3)
f2 (ppm)
f1 (ppm)

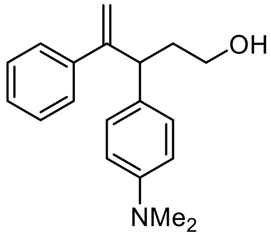

15b

^{1}H NMR (400 MHz, 298 K, $CDCl_3$)

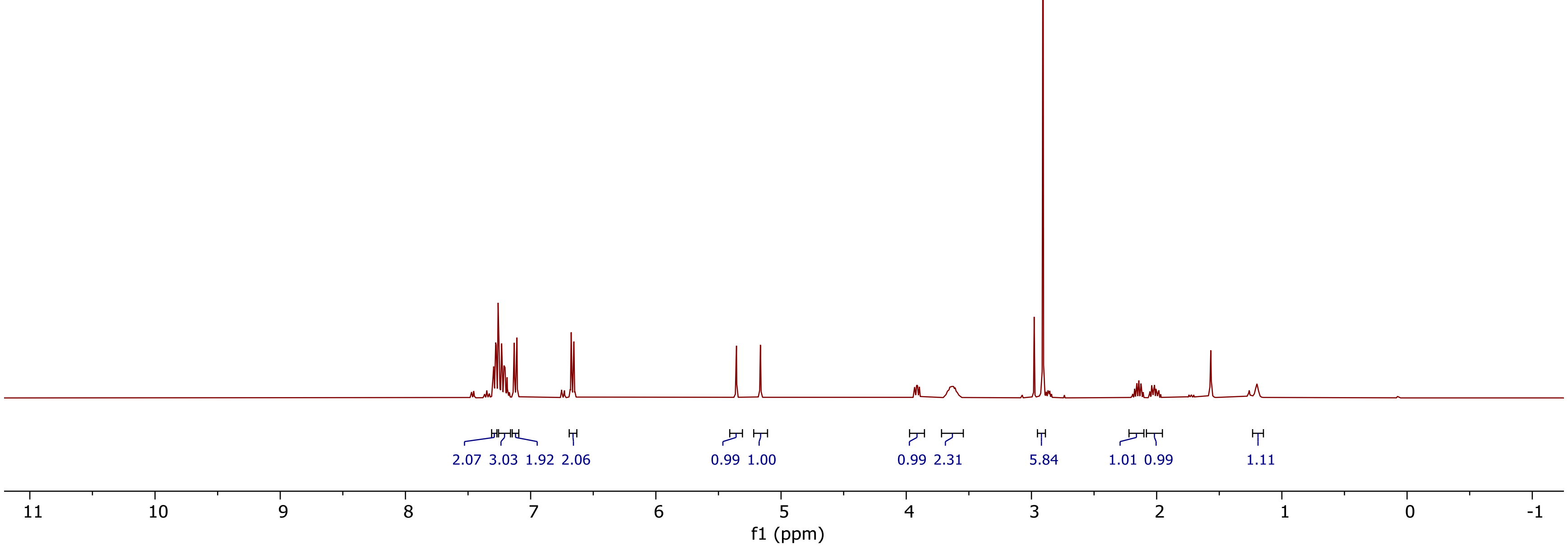

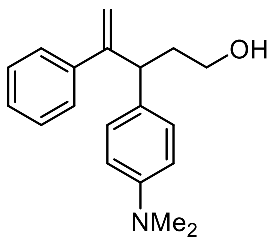

15b

$^{13}C\{^1H\}$ NMR (101 MHz, 298 K, $CDCl_3$)

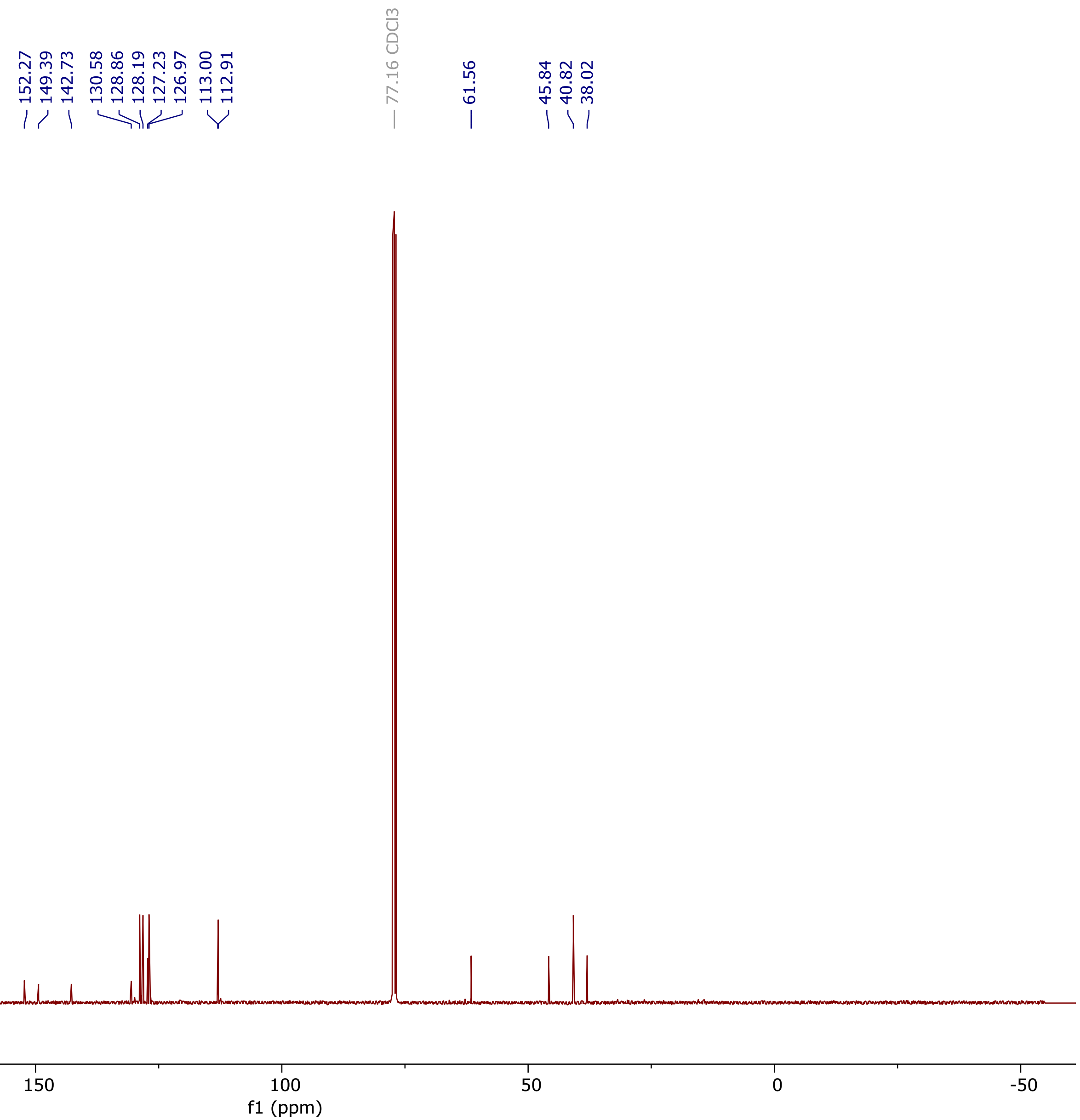

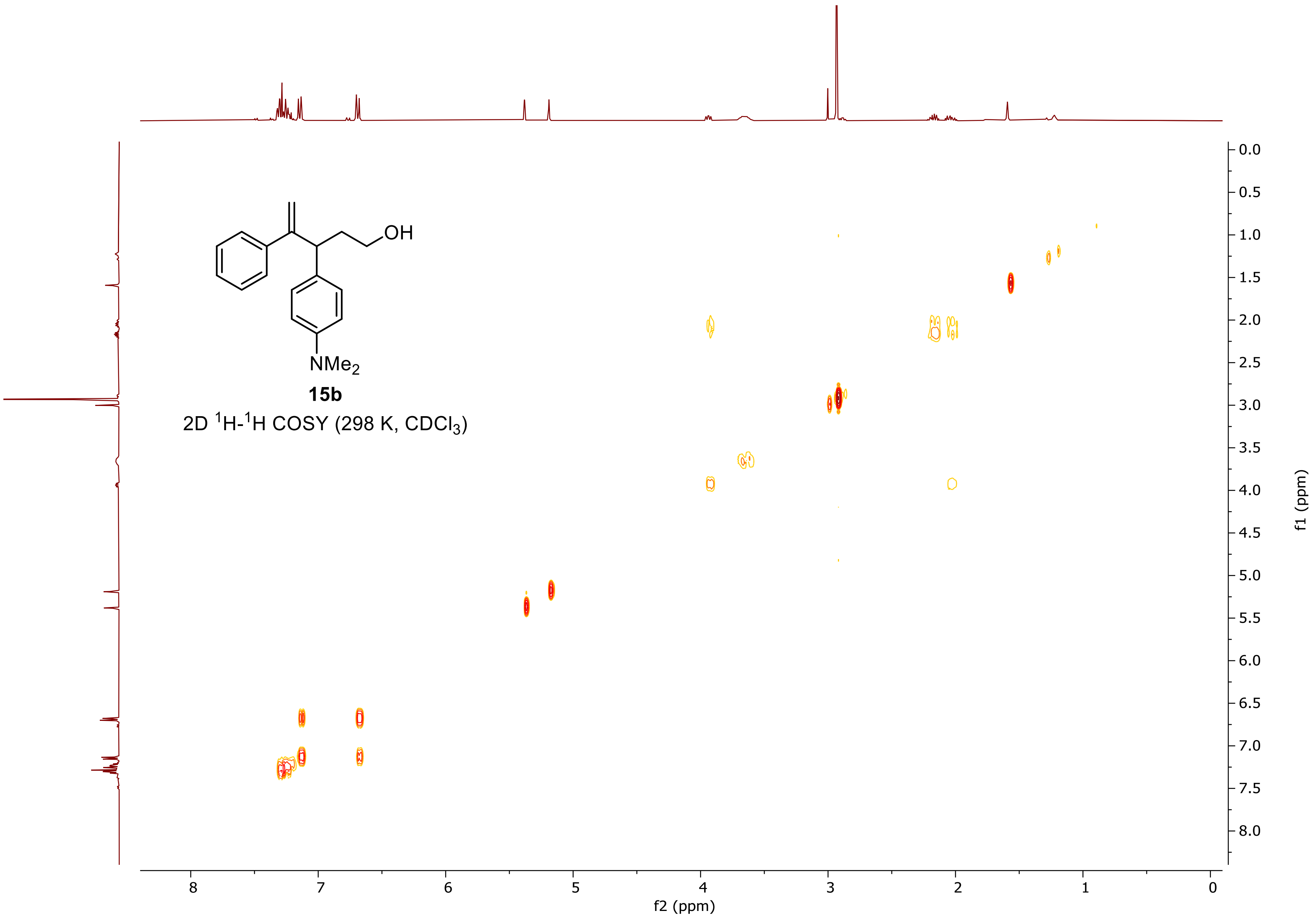
OH
NMe2
15b
2D 1H-1H COSY (298 K, CDCl3)
f2 (ppm)
f1 (ppm)

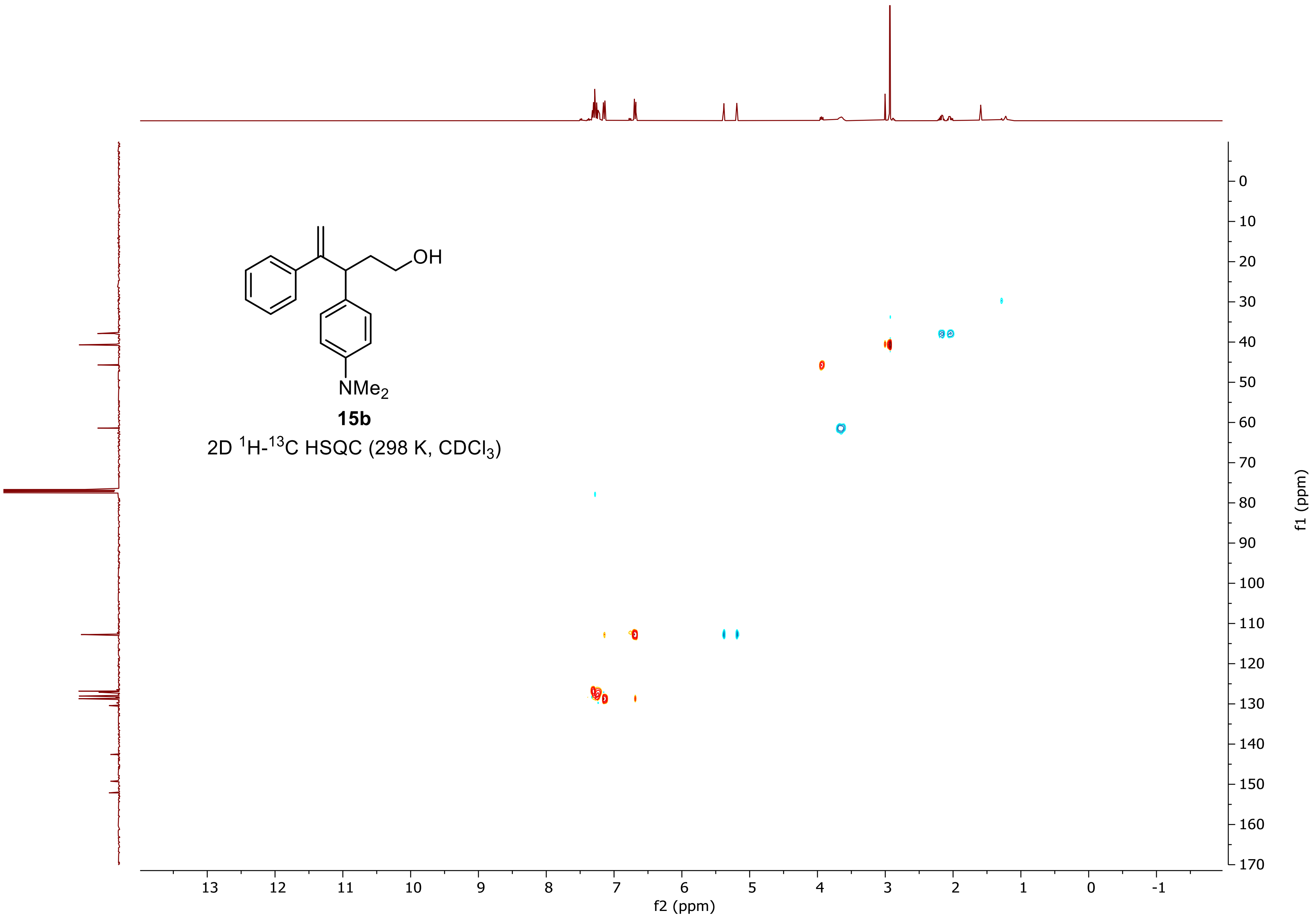

15b

2D ^{1}H-^{13}C HSQC (298 K, $CDCl_3$)

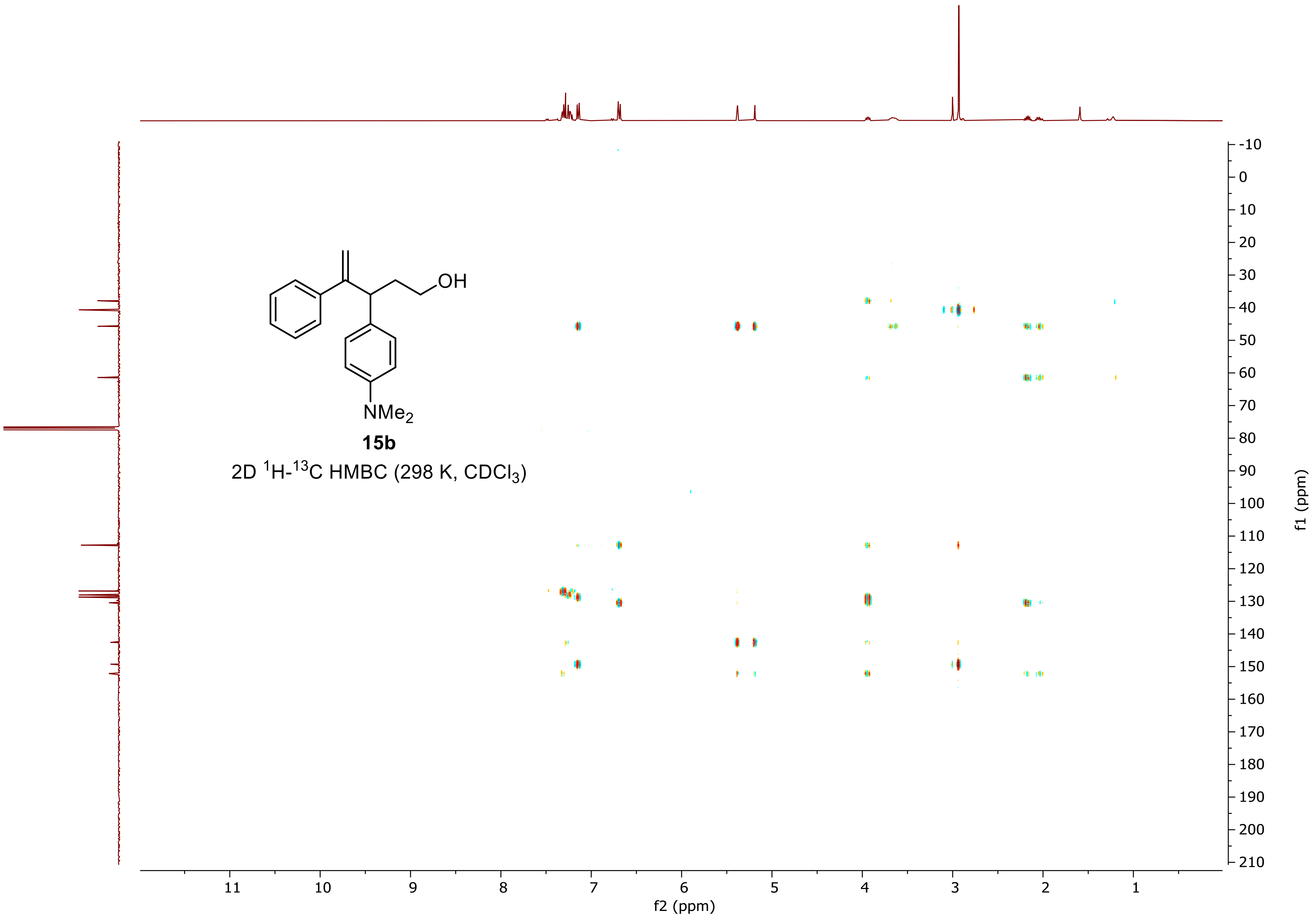

15b

2D ^{1}H-^{13}C HMBC (298 K, $CDCl_3$)

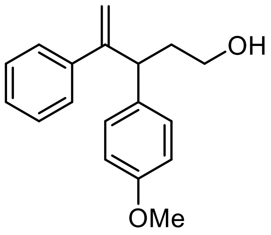

15c

^{1}H NMR (400 MHz, 298 K, $CDCl_3$)

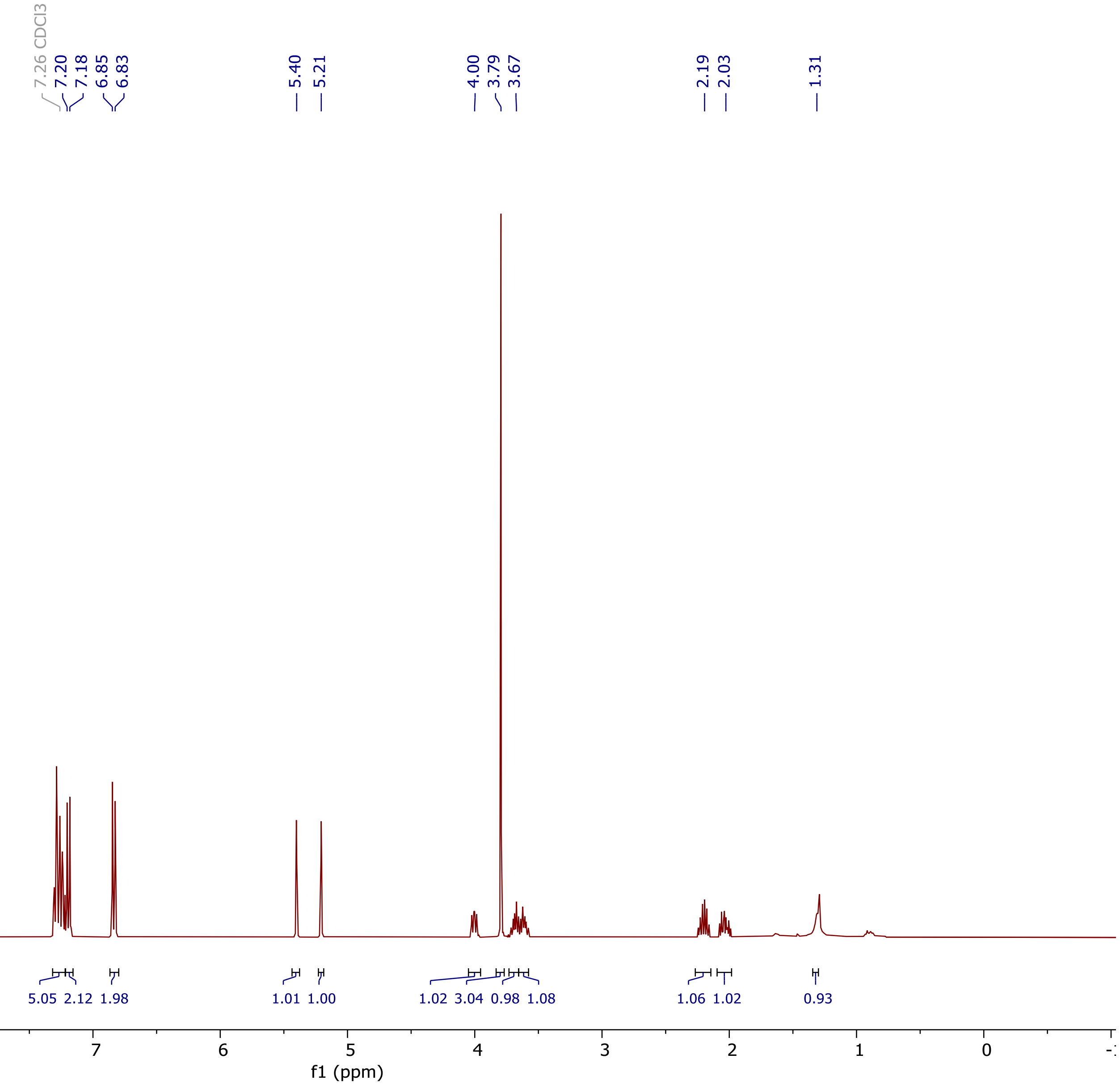

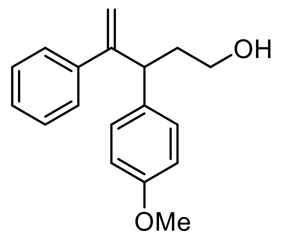

15c

$^{13}C\{^{1}H\}$ NMR (101 MHz, 298 K, $CDCl_3$)

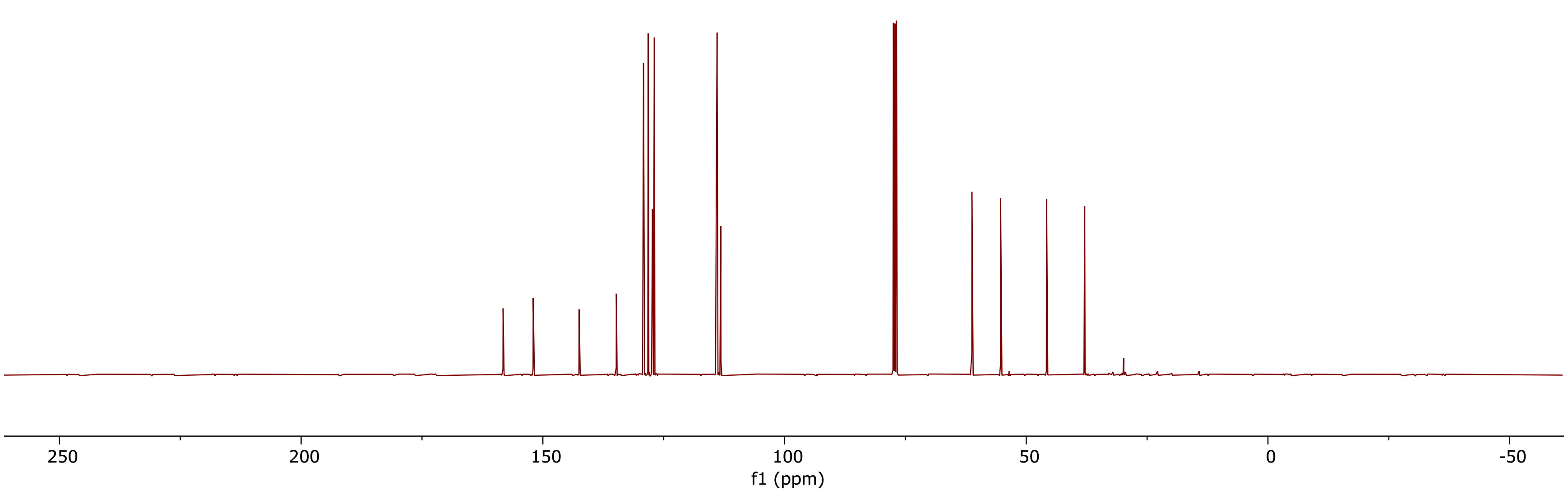

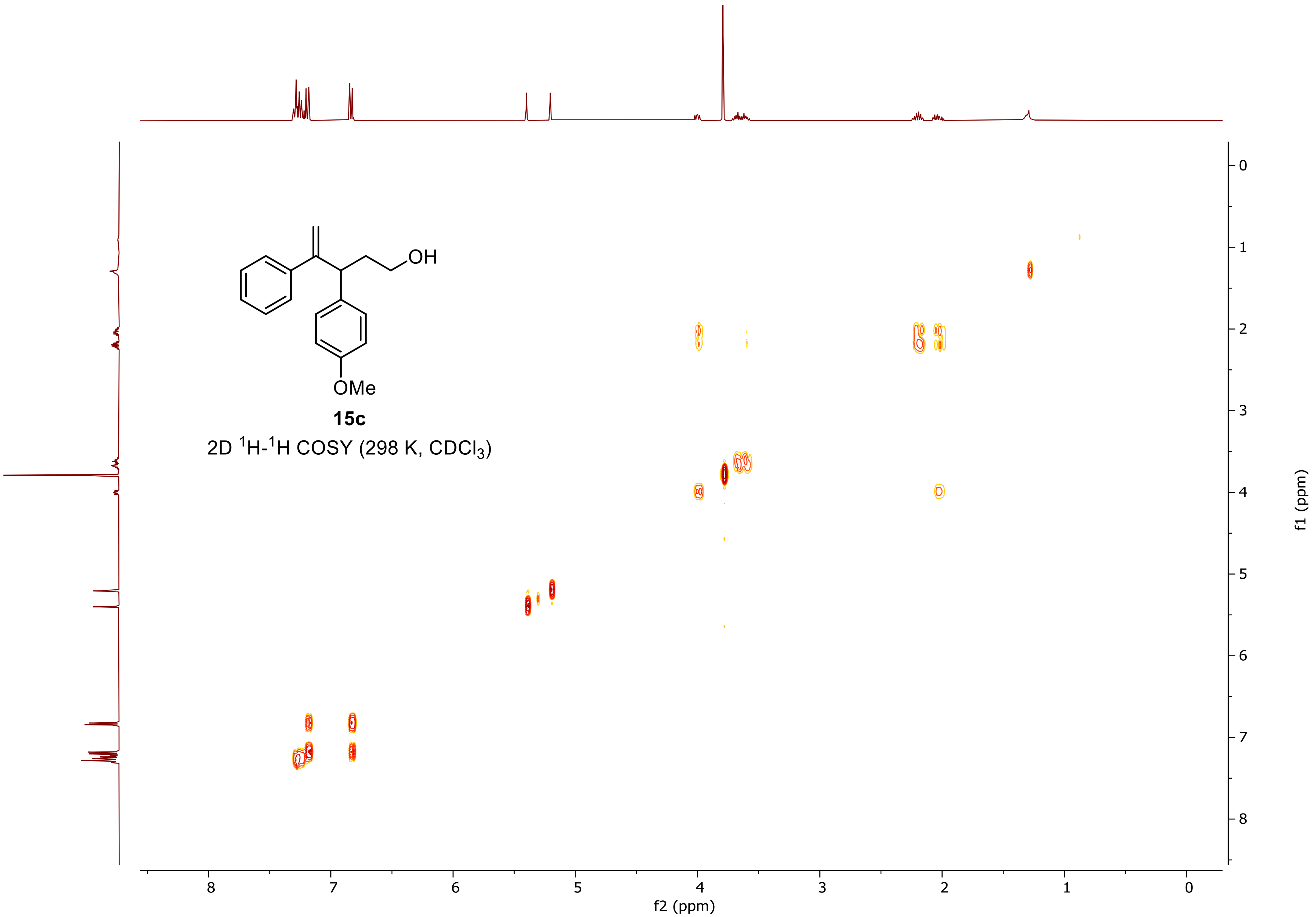

15c

2D ^{1}H-^{1}H COSY (298 K, $CDCl_3$)

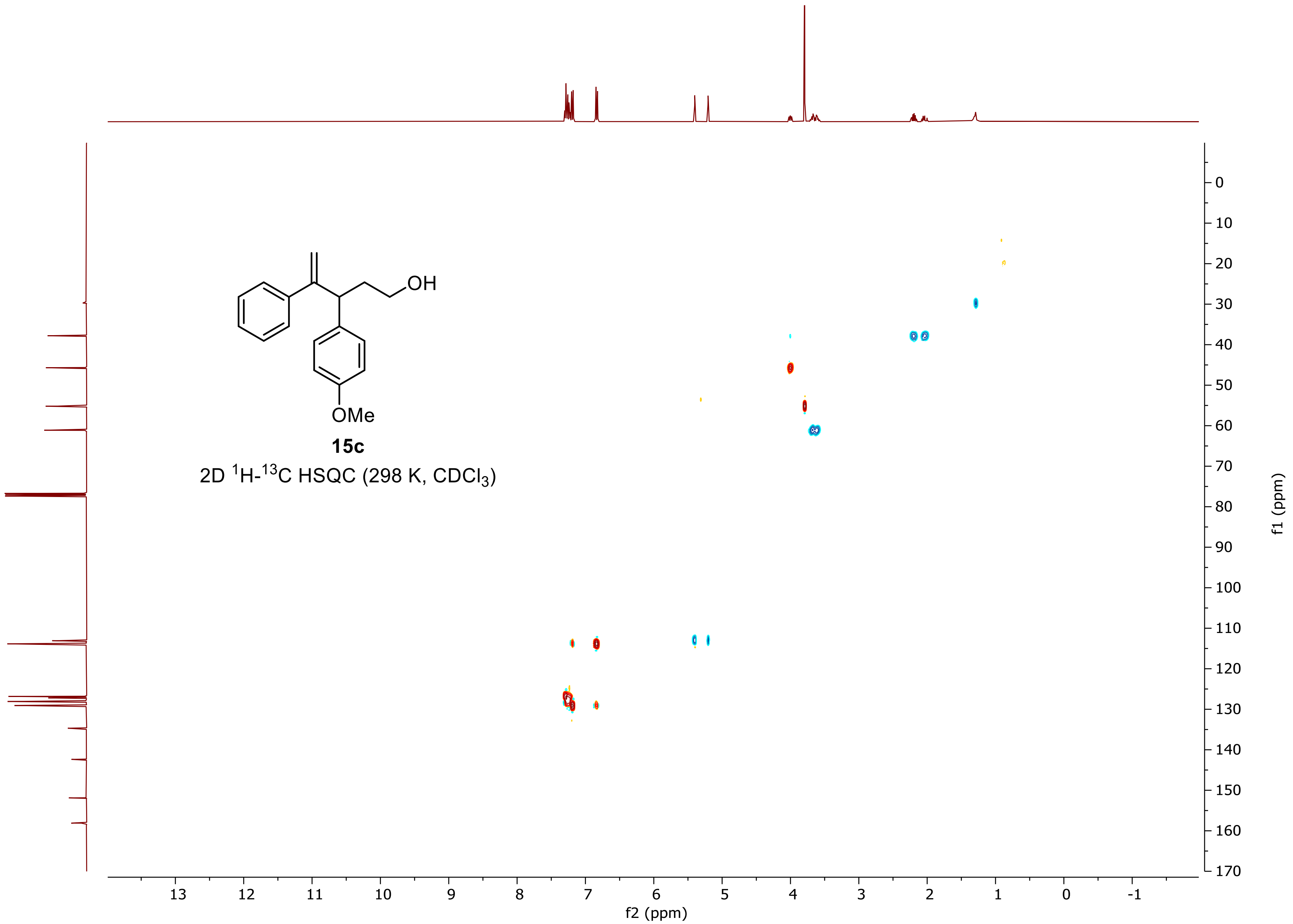
OH
OMe
15c
2D ^{1}H-^{13}C HSQC (298 K, $CDCl_3$)
f2 (ppm)
f1 (ppm)

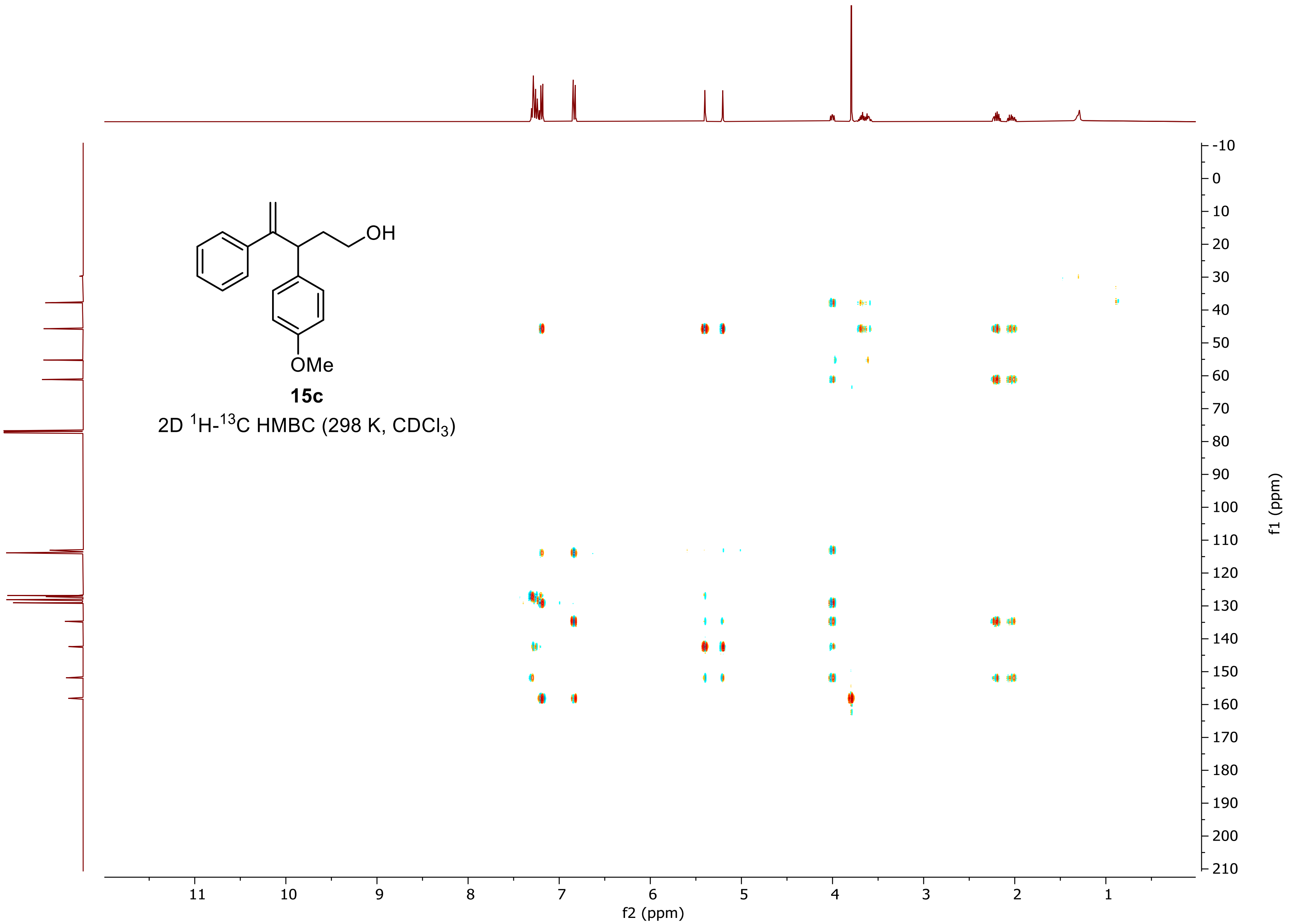
OH
OMe
15c
2D ¹H-¹³C HMBC (298 K, CDCl3)
f1 (ppm)
f2 (ppm)

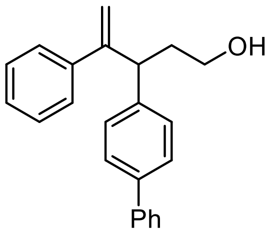

15d

^{1}H NMR (400 MHz, 298 K, $CDCl_3$)

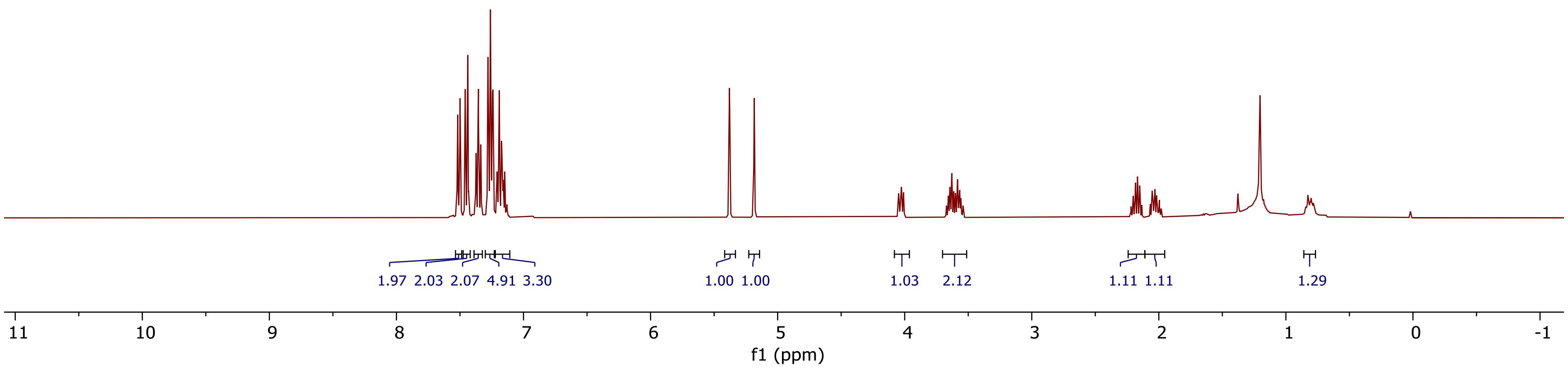

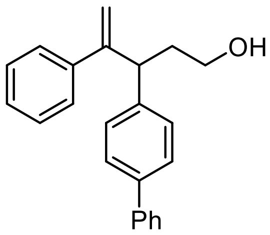

15d

$^{13}C\{^{1}H\}$ NMR (101 MHz, 298 K, $CDCl_3$)

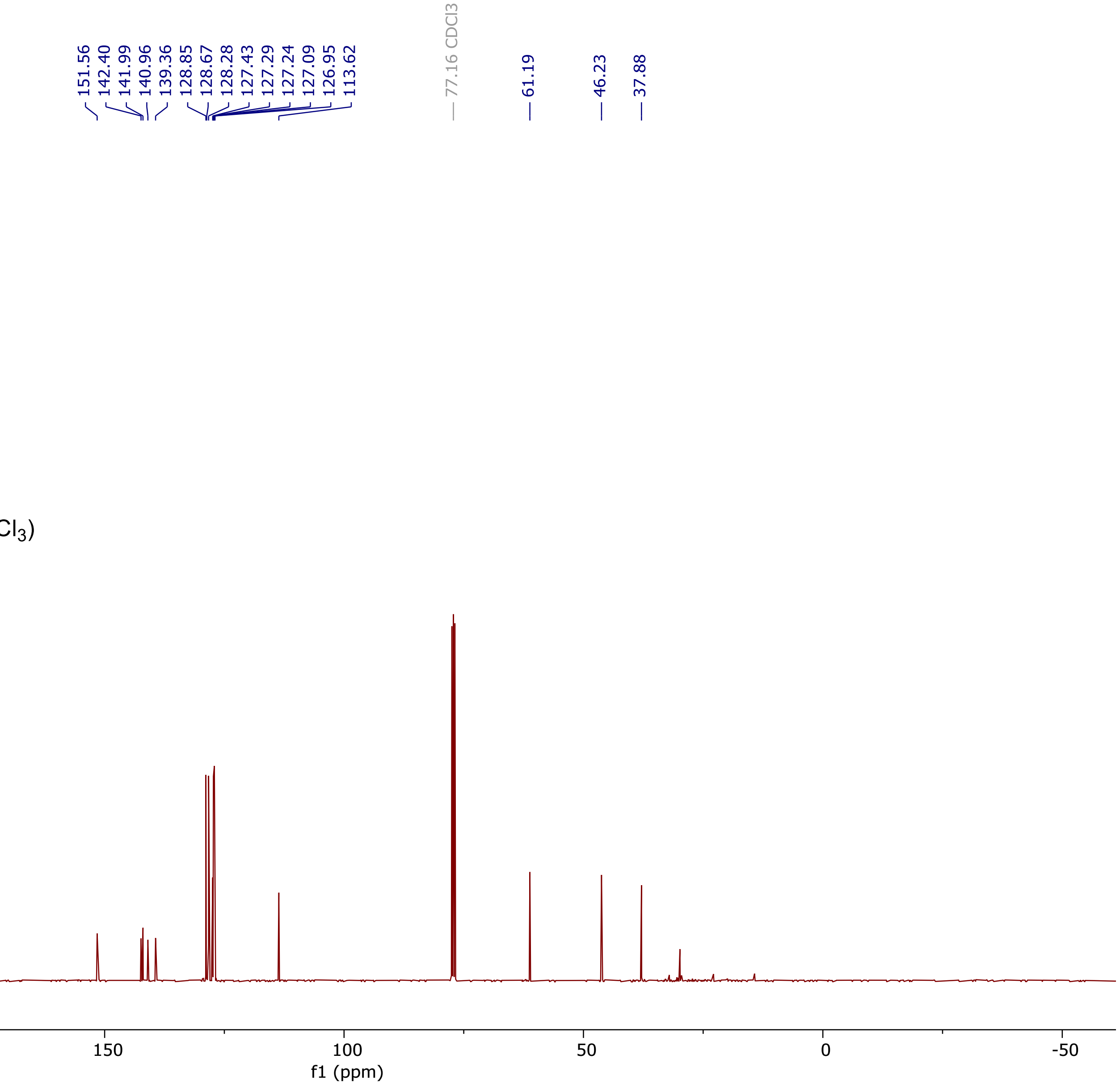

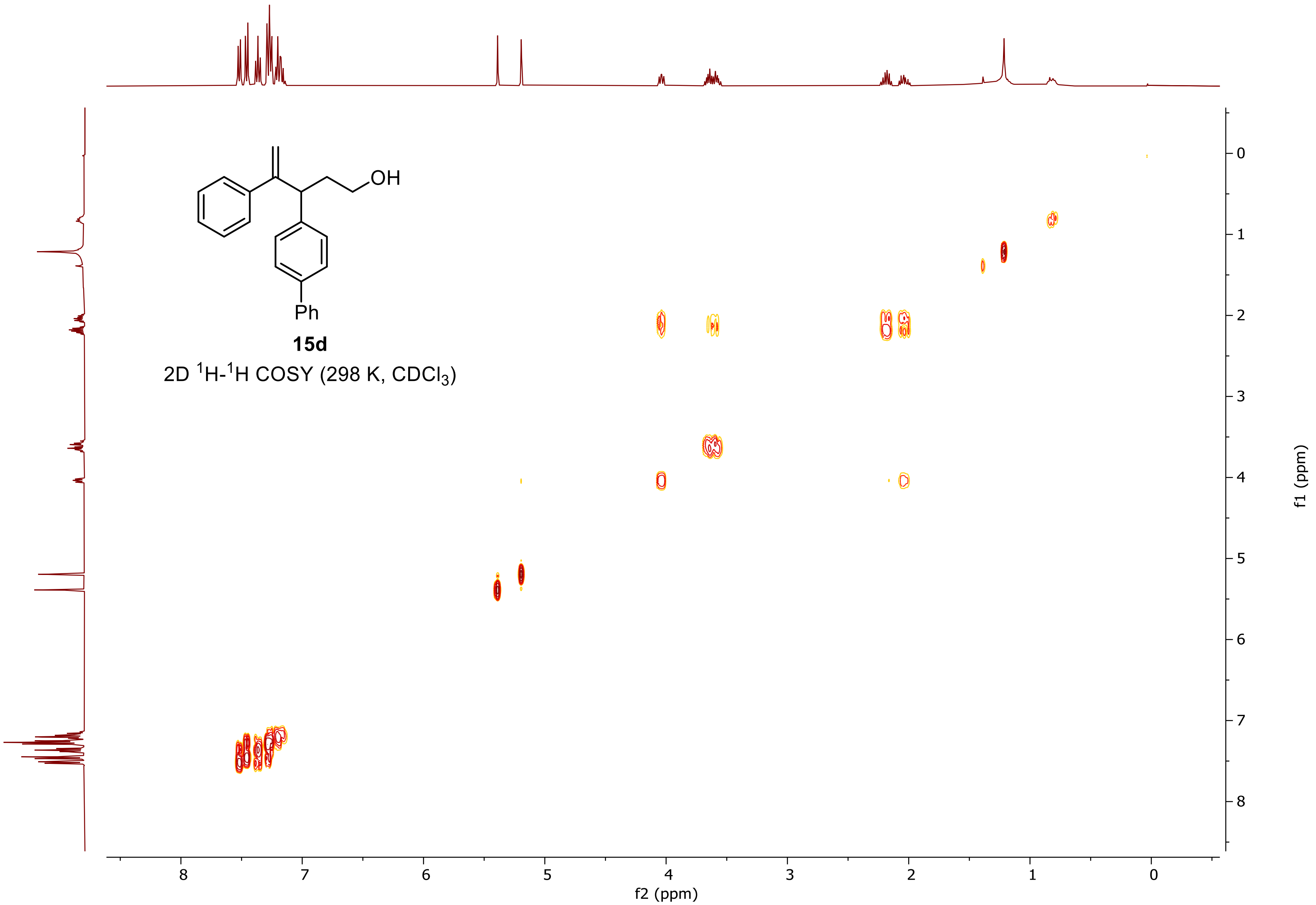

15d

2D ^{1}H-^{1}H COSY (298 K, $CDCl_3$)

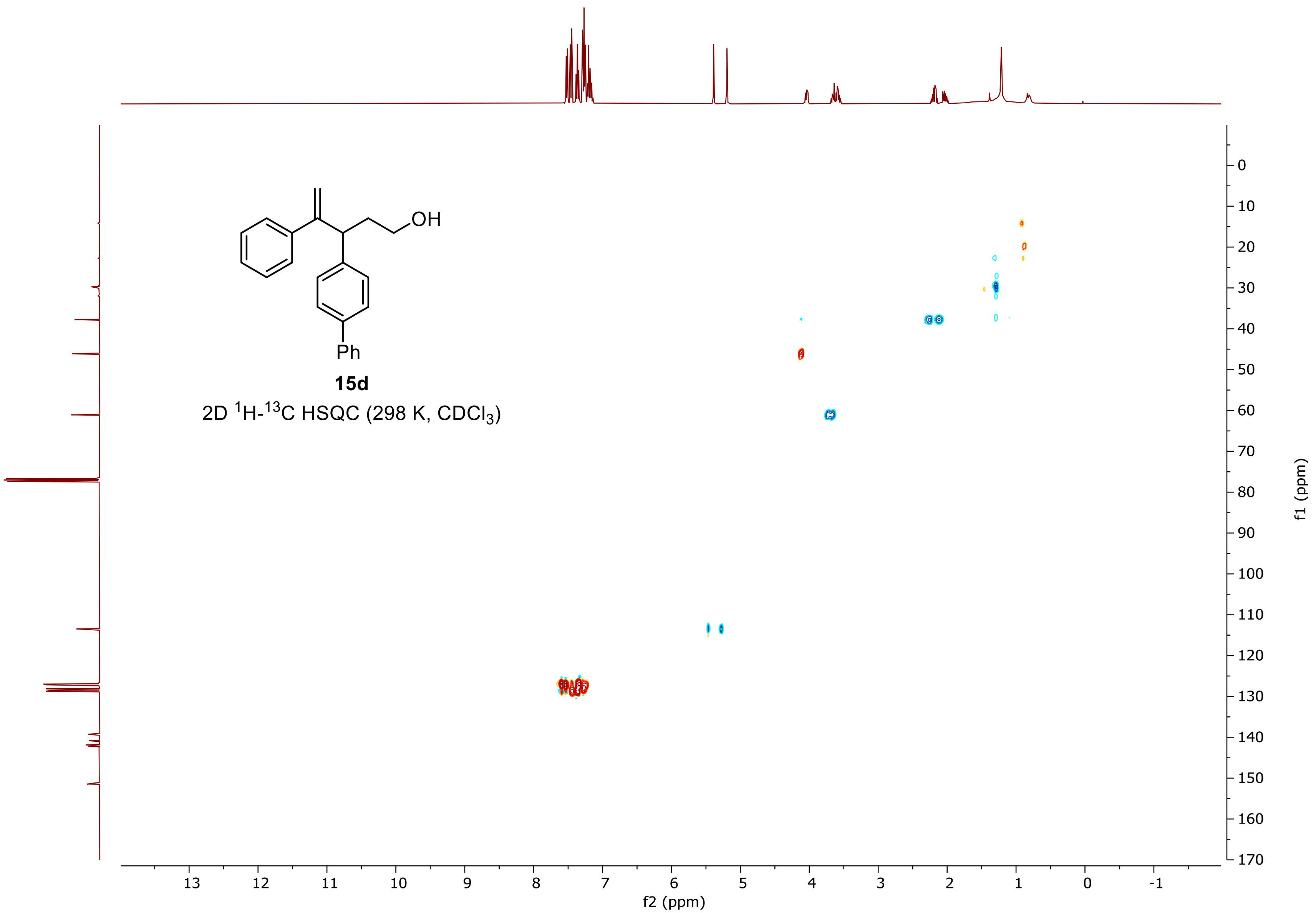
OH
Ph
15d
2D ¹H-¹³C HSQC (298 K, CDCl₃)
f2 (ppm)
f1 (ppm)

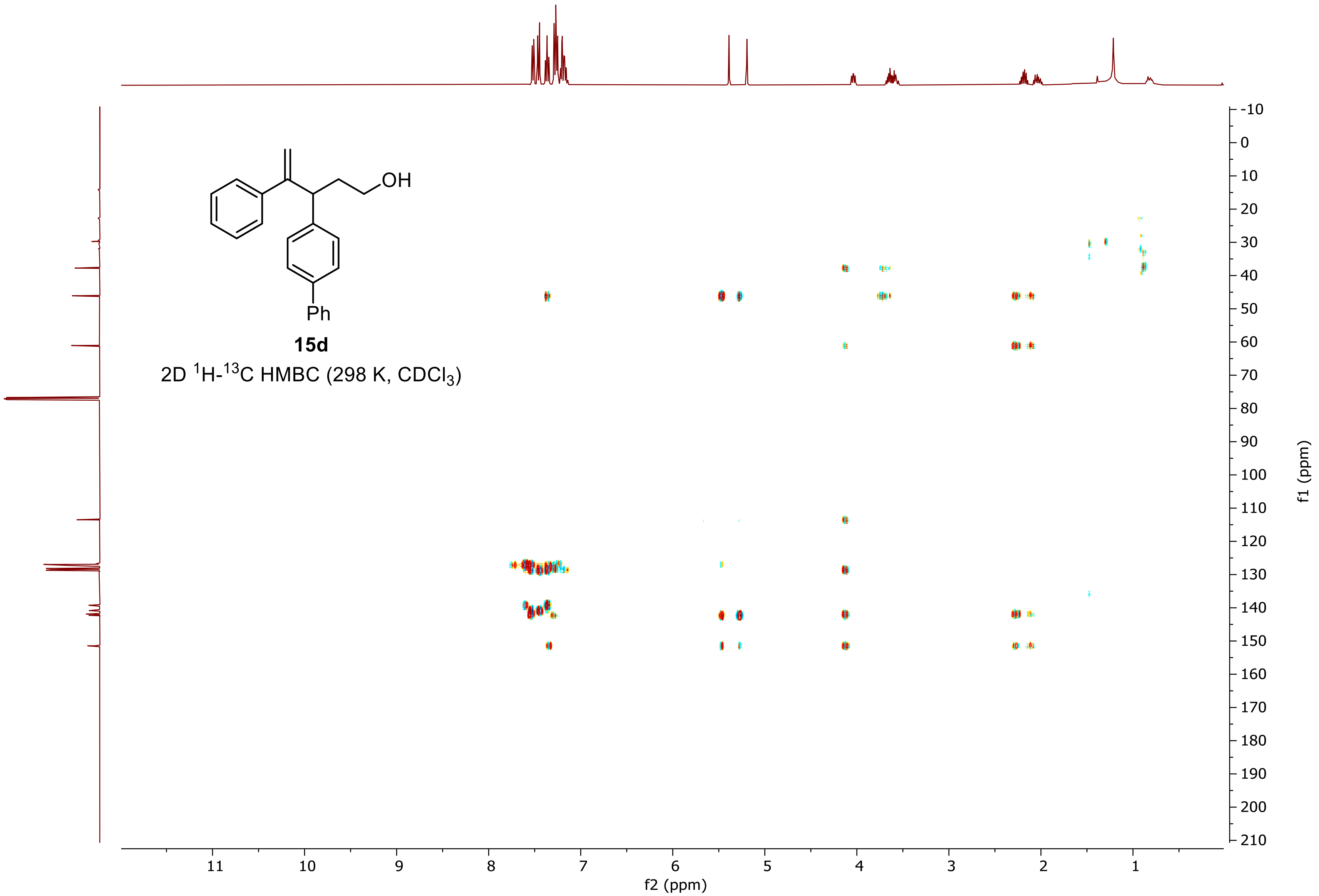
OH
Ph
15d
2D 1H-13C HMBC (298 K, CDCl3)
f1 (ppm)
f2 (ppm)

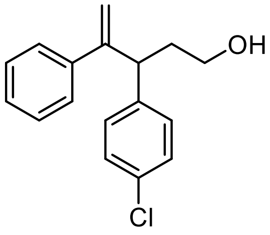

15e

^{1}H NMR (400 MHz, 298 K, $CDCl_3$)

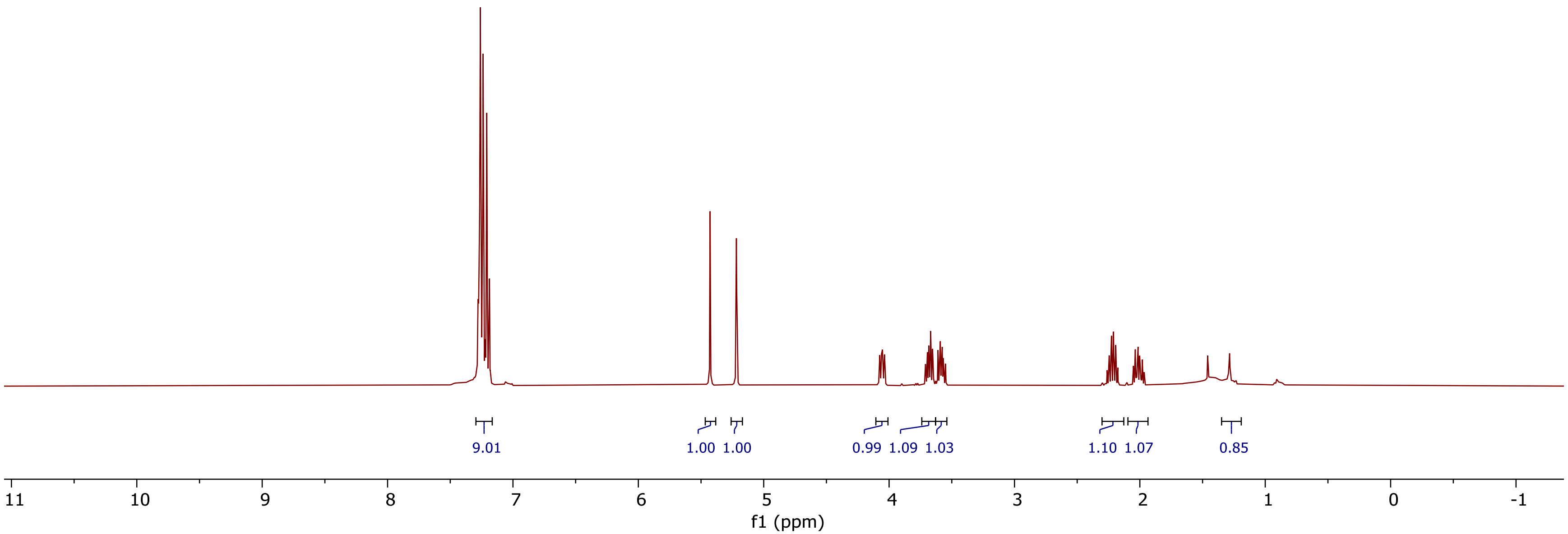

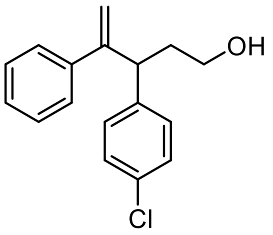

15e

$^{13}C\{^{1}H\}$ NMR (101 MHz, 298 K, $CDCl_3$)

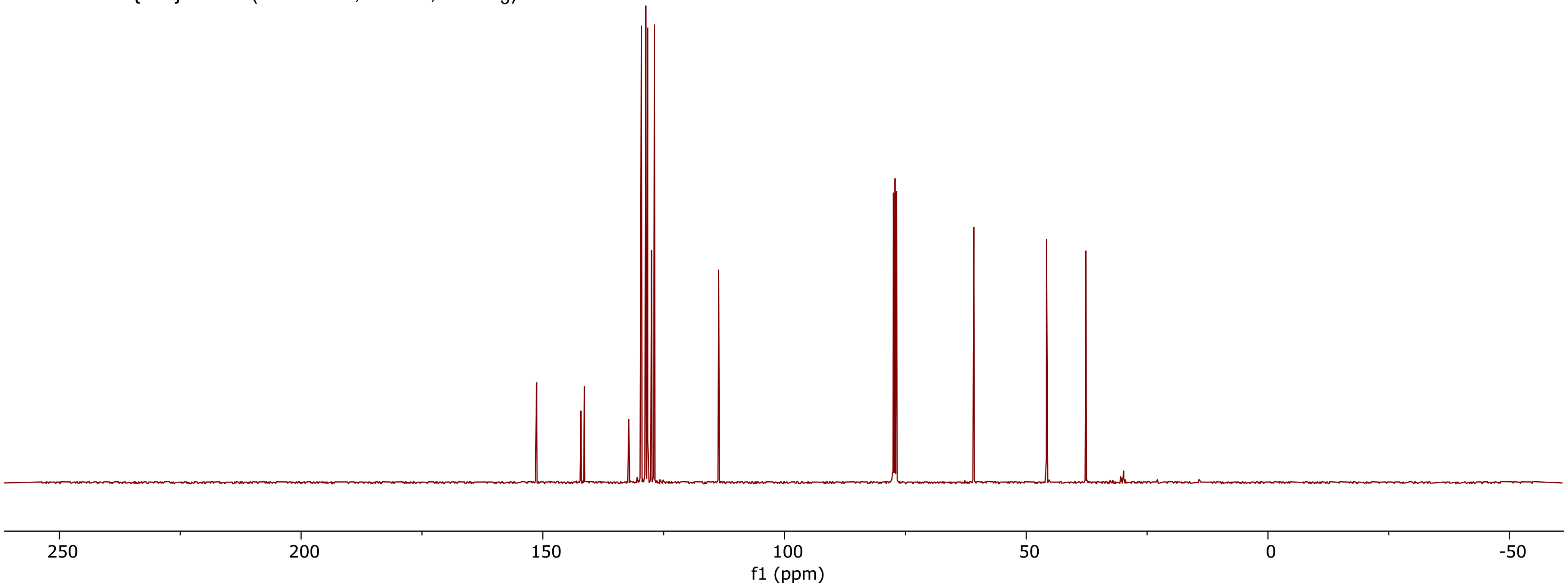

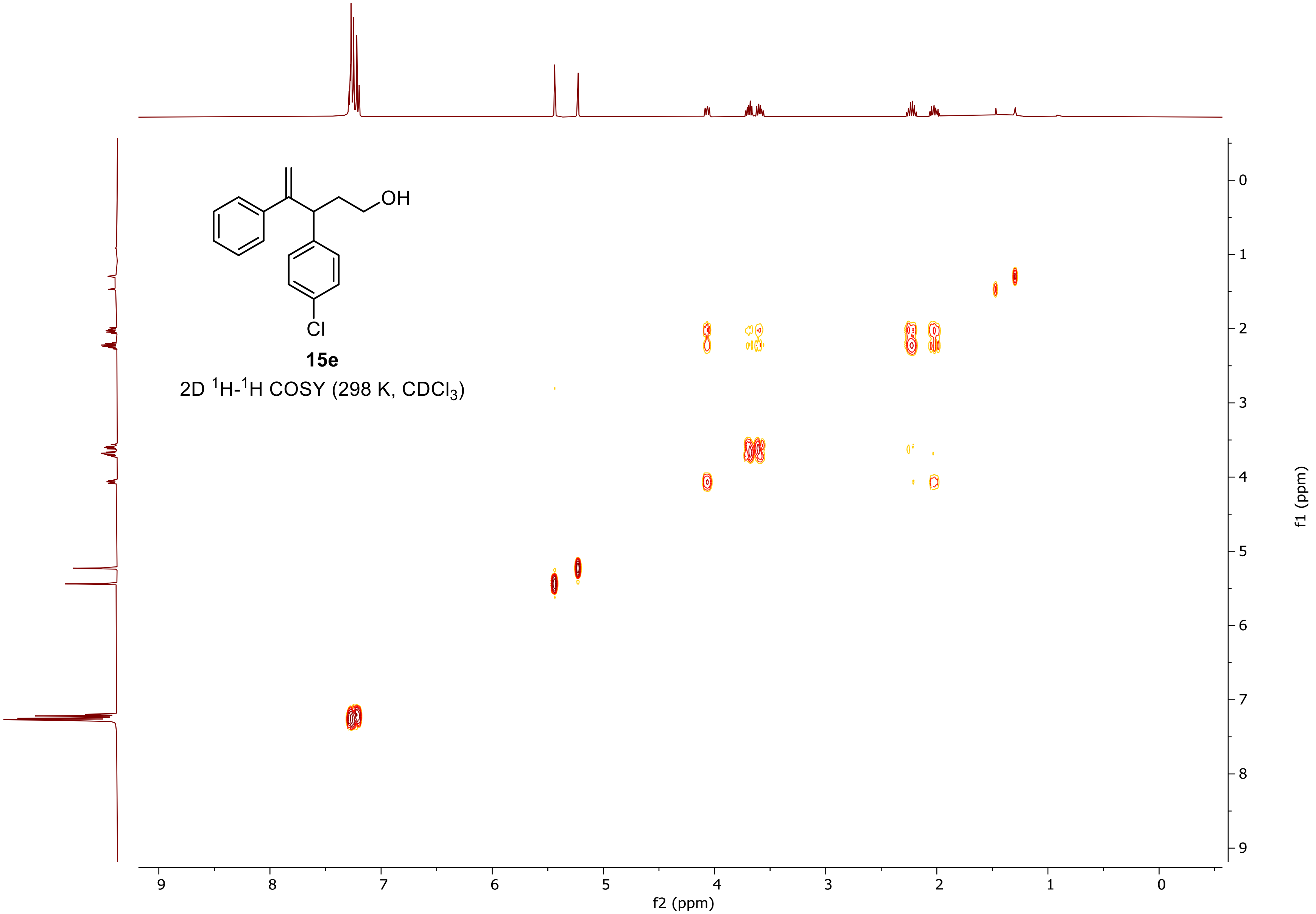

15e

2D ^{1}H-^{1}H COSY (298 K, $CDCl_3$)

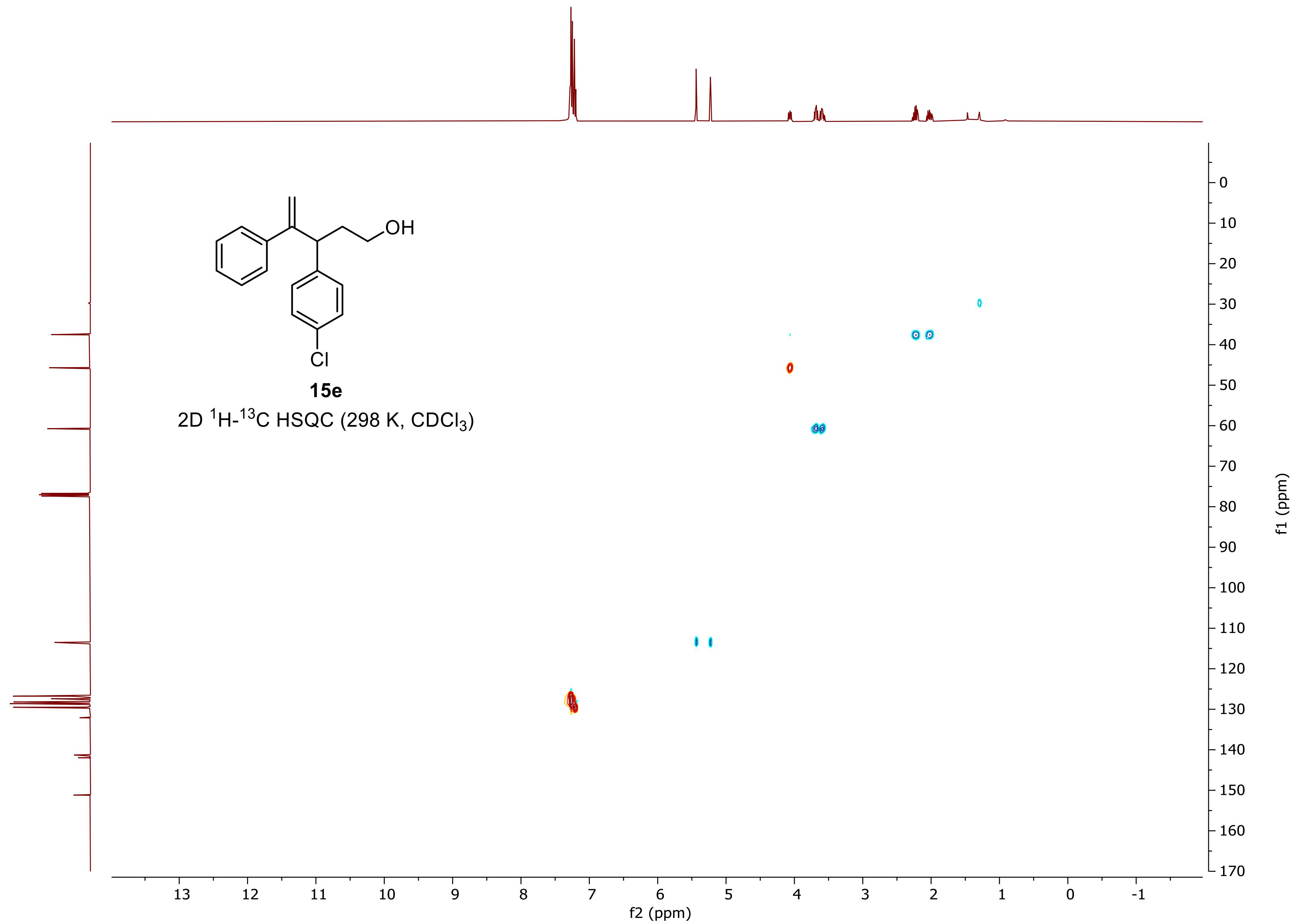
OH
Cl
15e
2D ^{1}H-^{13}C HSQC (298 K, $CDCl_3$)
f1 (ppm)
f2 (ppm)

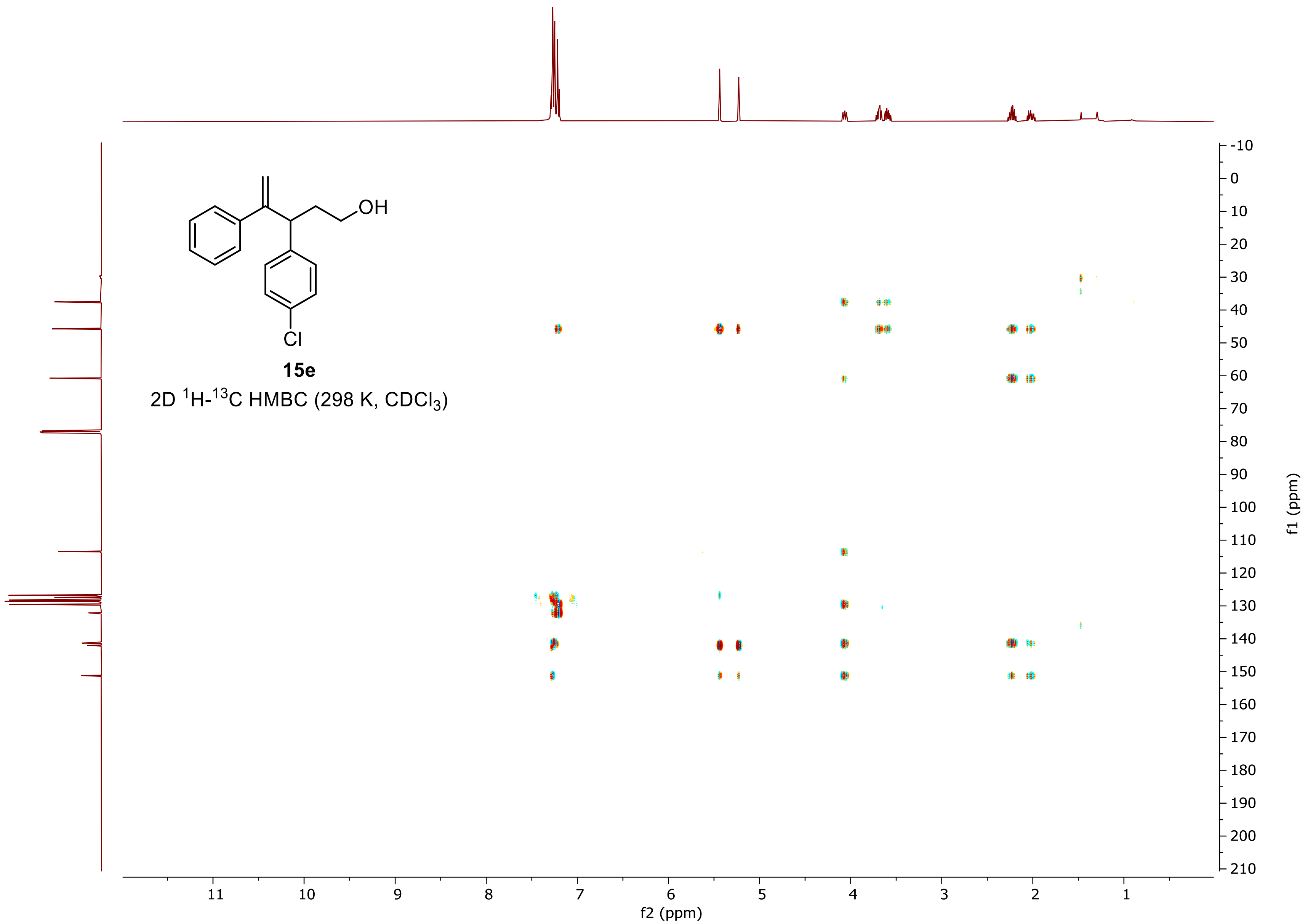

15e

2D ^{1}H-^{13}C HMBC (298 K, $CDCl_3$)

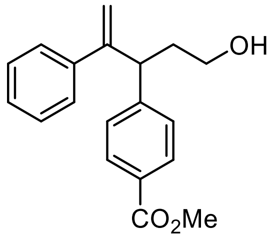

15f

^{1}H NMR (400 MHz, 298 K, $CDCl_3$)

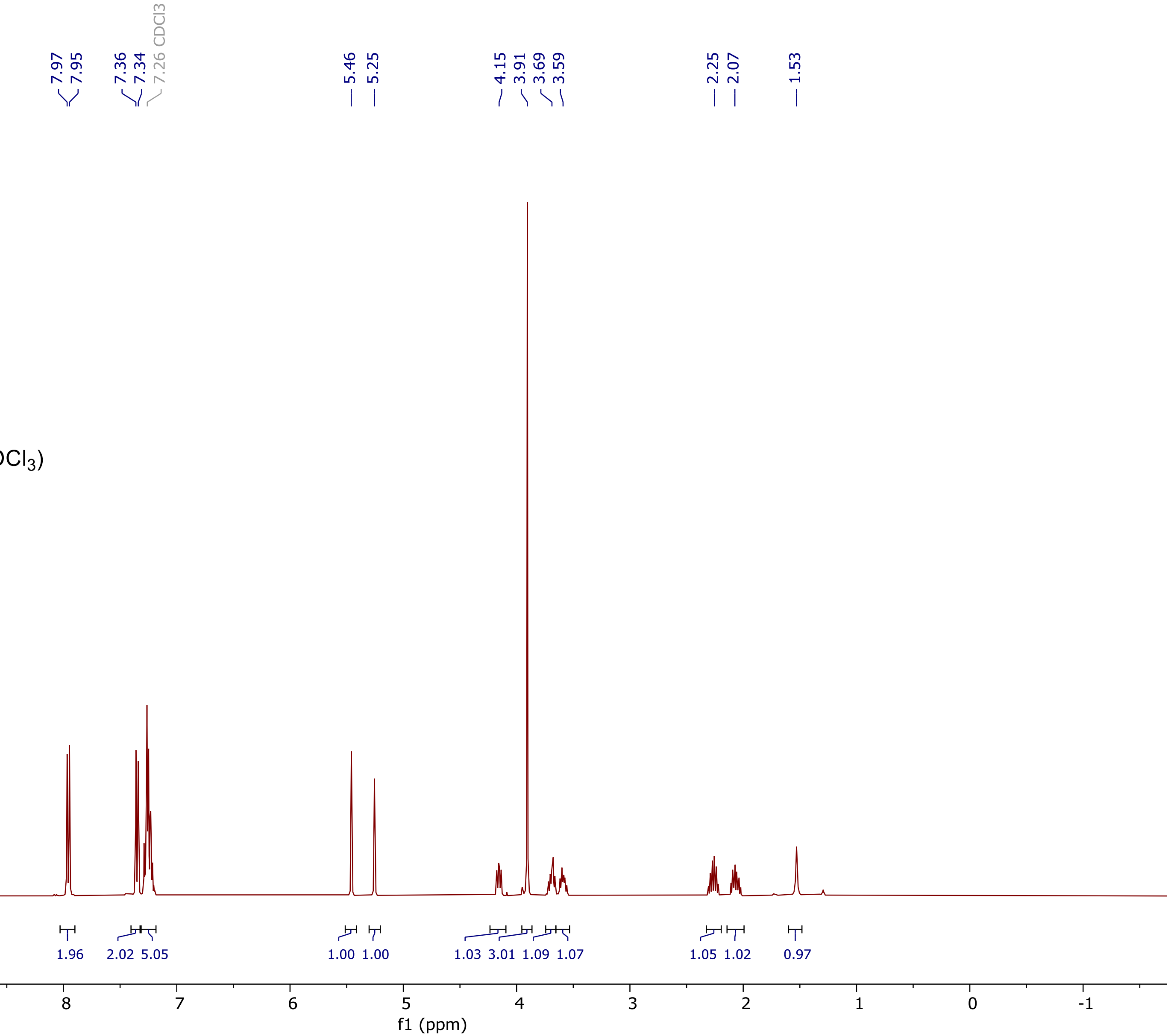

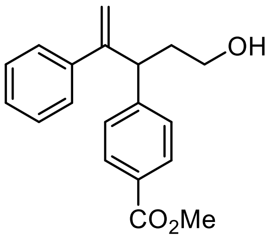

15f

$^{13}C\{^1H\}$ NMR (101 MHz, 298 K, $CDCl_3$)

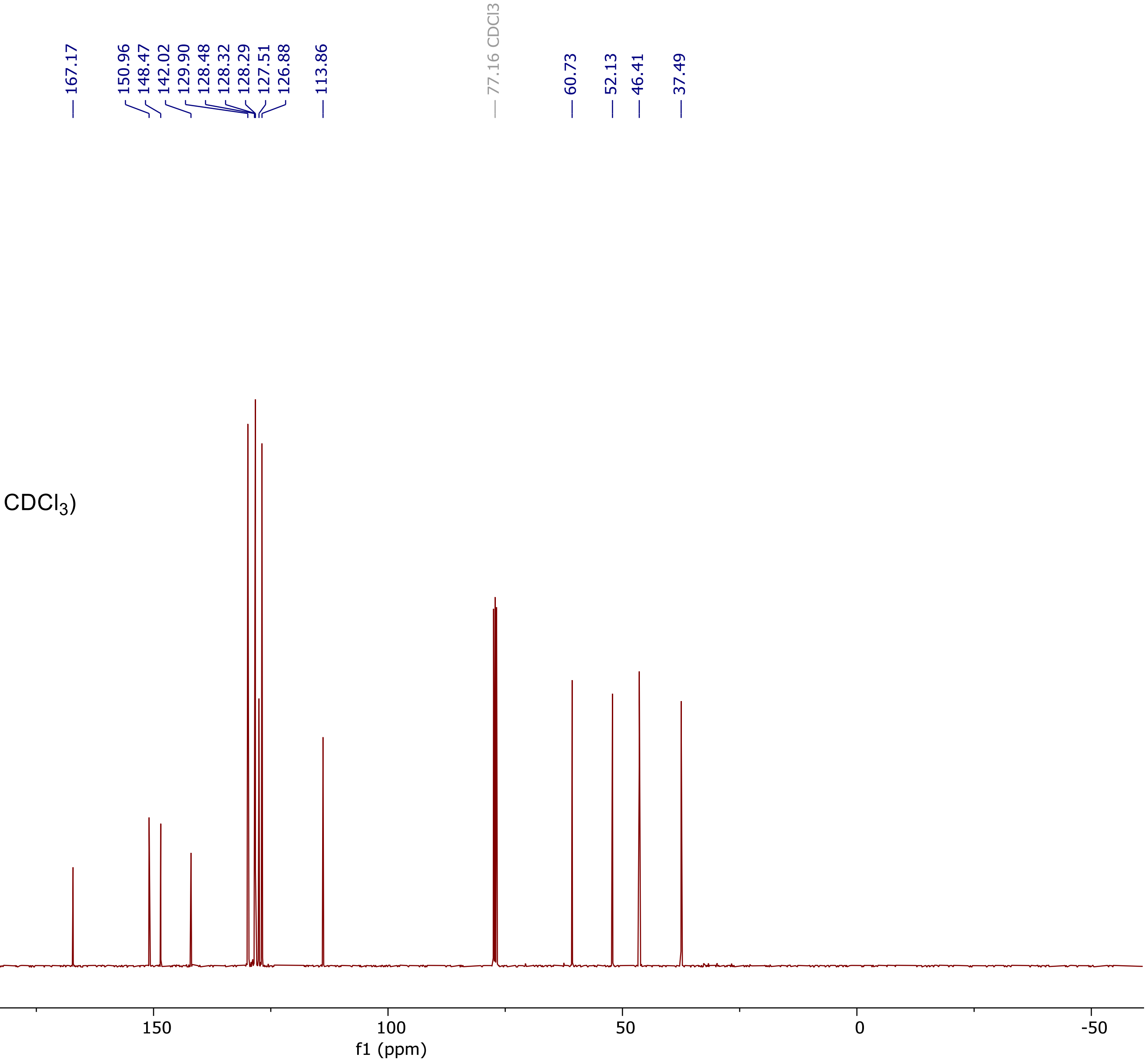

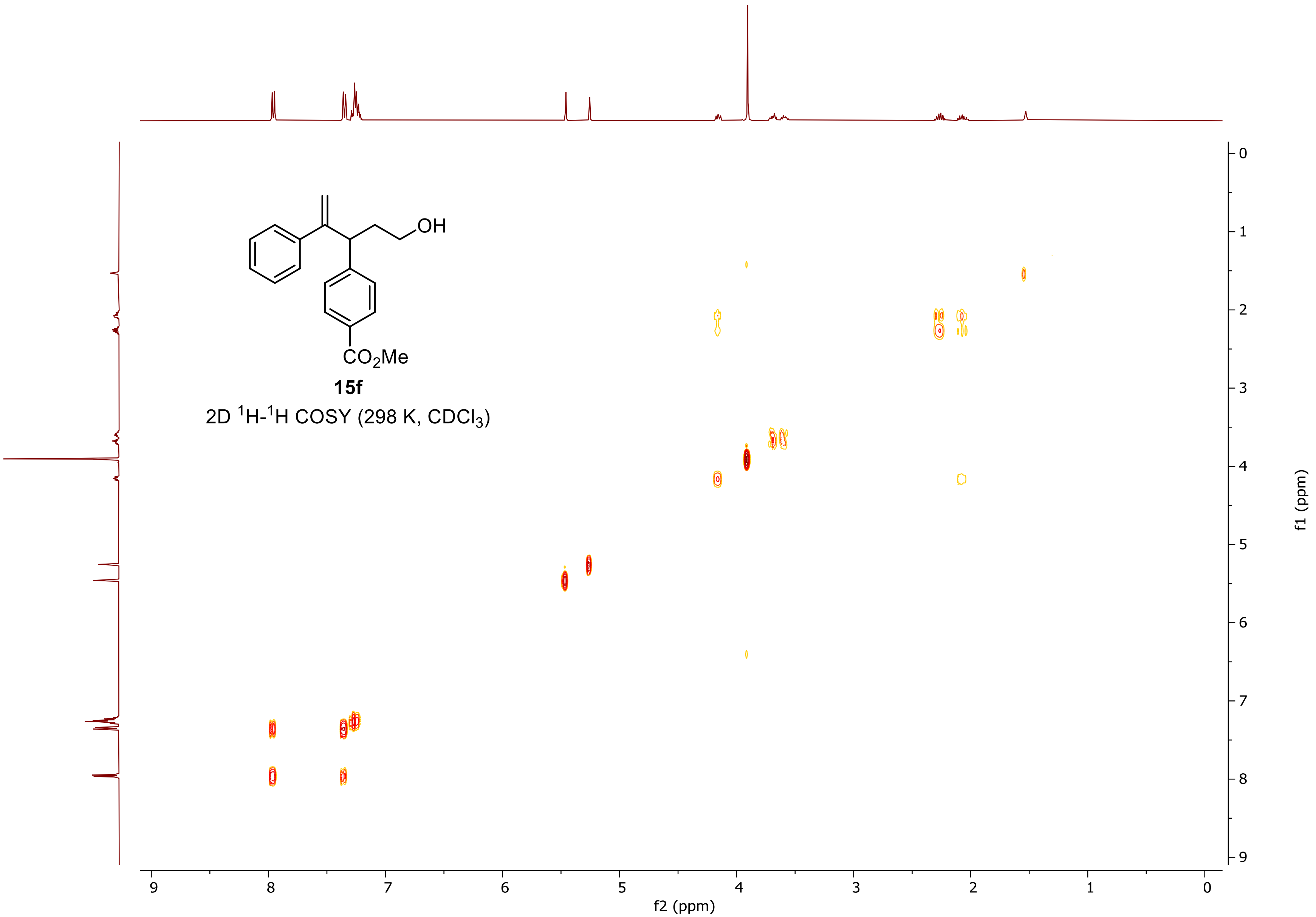
OH
CO_2Me
15f
2D ^1H-^1H COSY (298 K, $CDCl_3$)
f2 (ppm)
f1 (ppm)
9
8
7
6
5
4
3
2
1
0

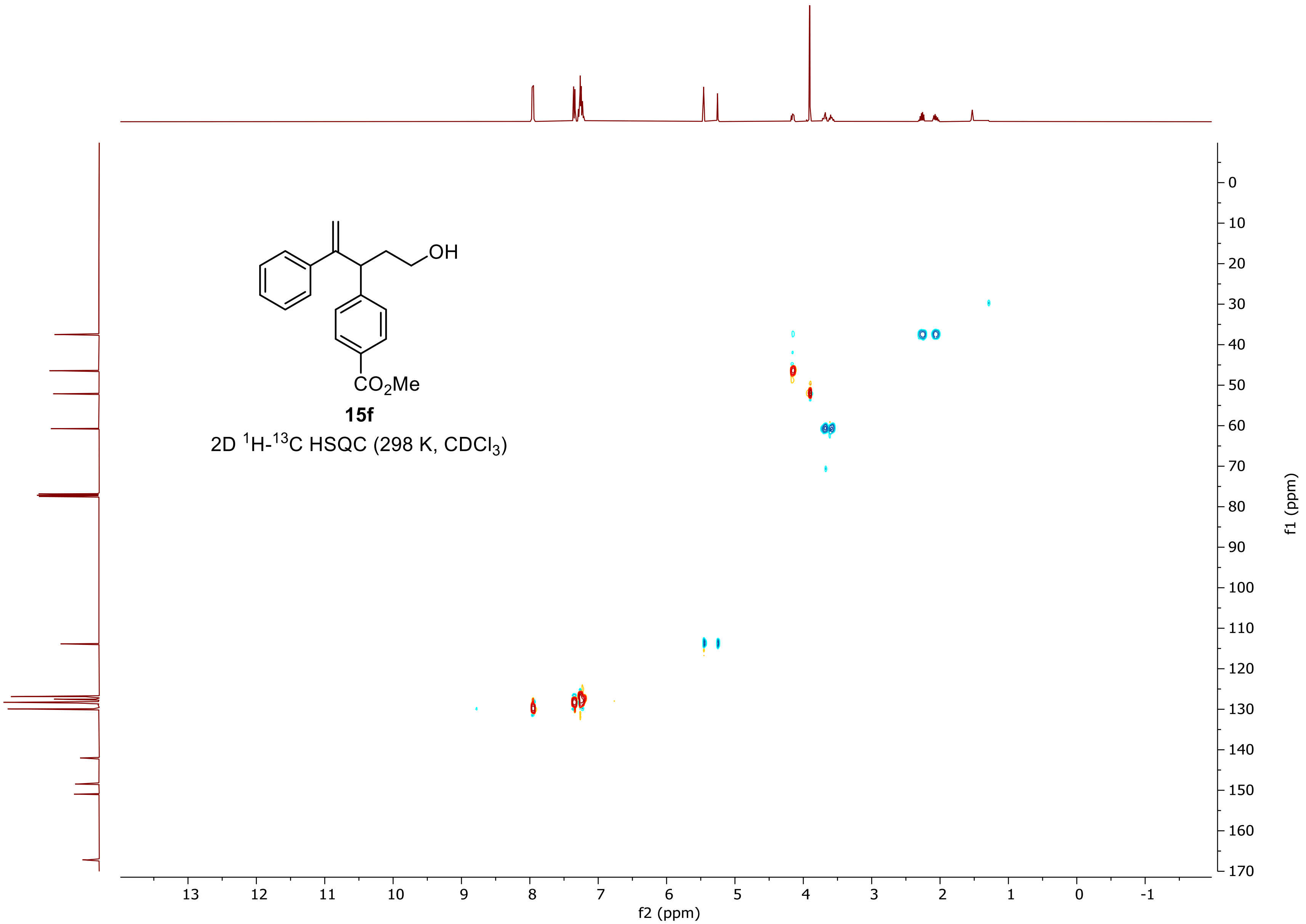
OH
CO2Me
15f
2D 1H-13C HSQC (298 K, CDCl3)
f1 (ppm)
f2 (ppm)
0
10
20
30
40
50
60
70
80
90
100
110
120
130
140
150
160
170
13
12
11
10
9
8
7
6
5
4
3
2
1
0
-1

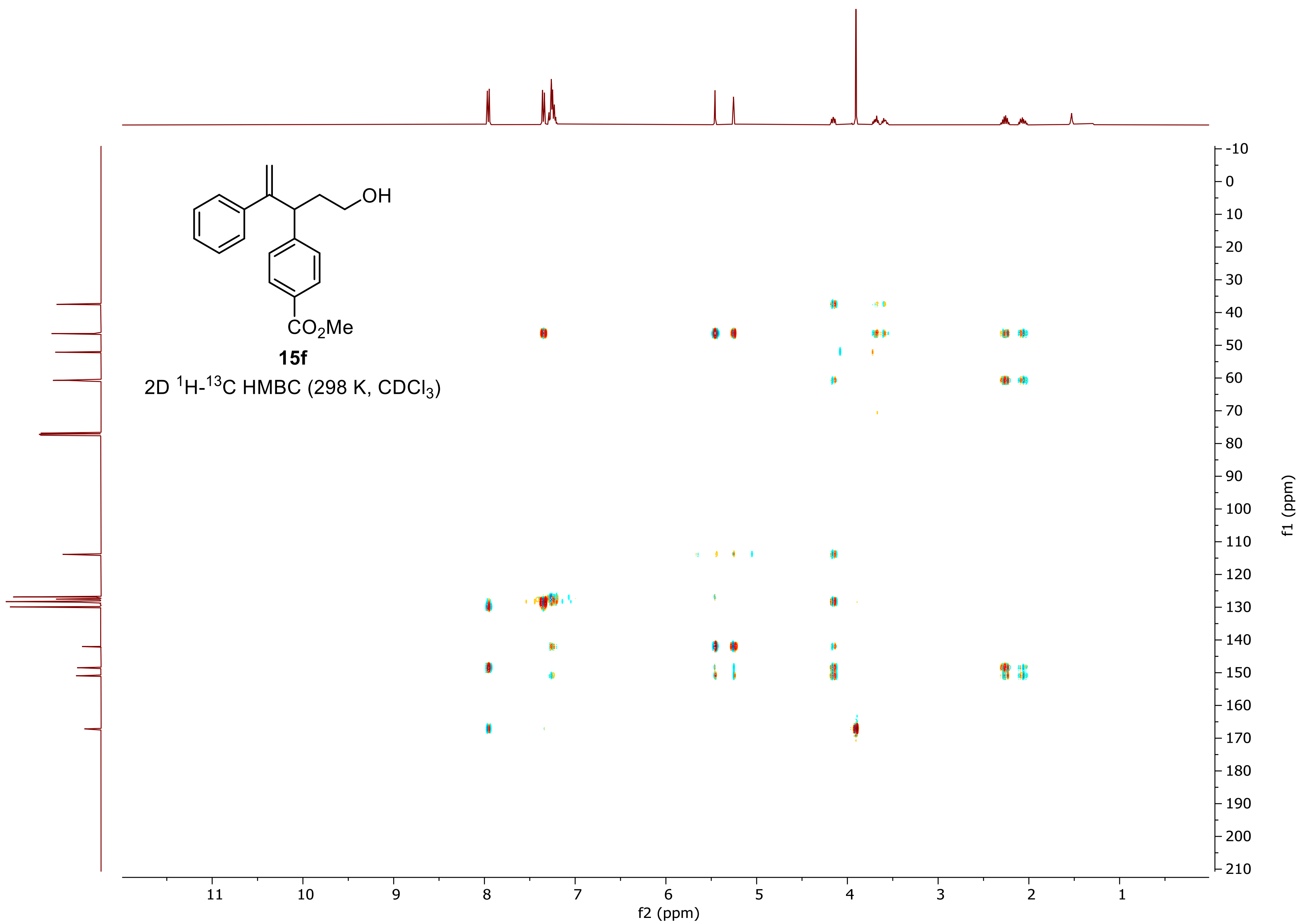
OH
CO_2Me
15f
2D ^{1}H-^{13}C HMBC (298 K, $CDCl_3$)
f1 (ppm)
f2 (ppm)

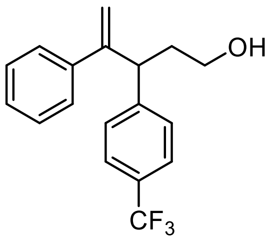

15g

^{1}H NMR (400 MHz, 298 K, $CDCl_3$)

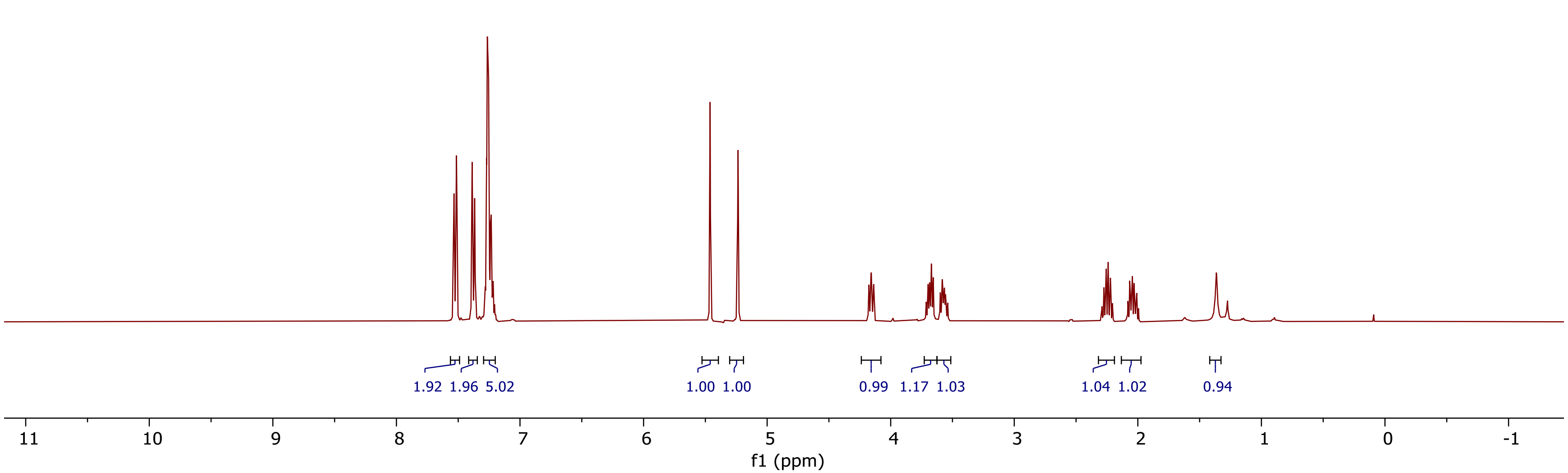

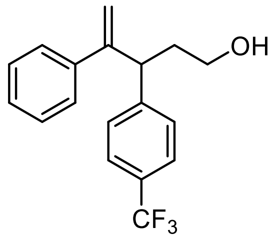

15g

$^{13}C\{^{1}H\}$ NMR (101 MHz, 298 K, $CDCl_3$)

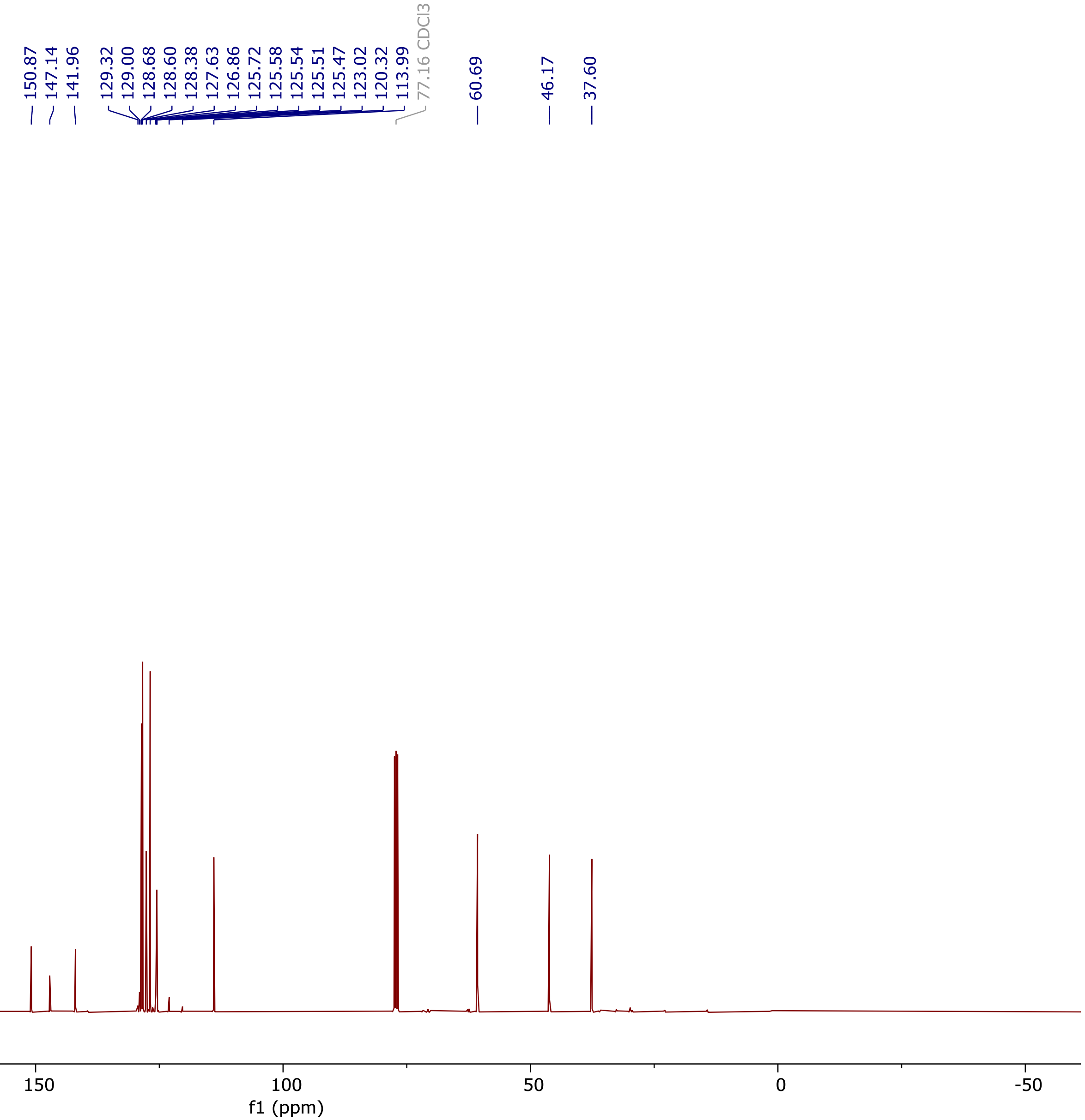

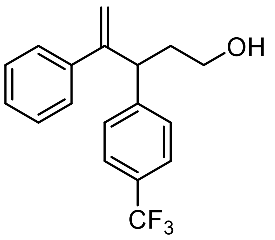

15g

$^{19}F\{^{1}H\}$ NMR (282 MHz, 298 K, $CDCl_3$)

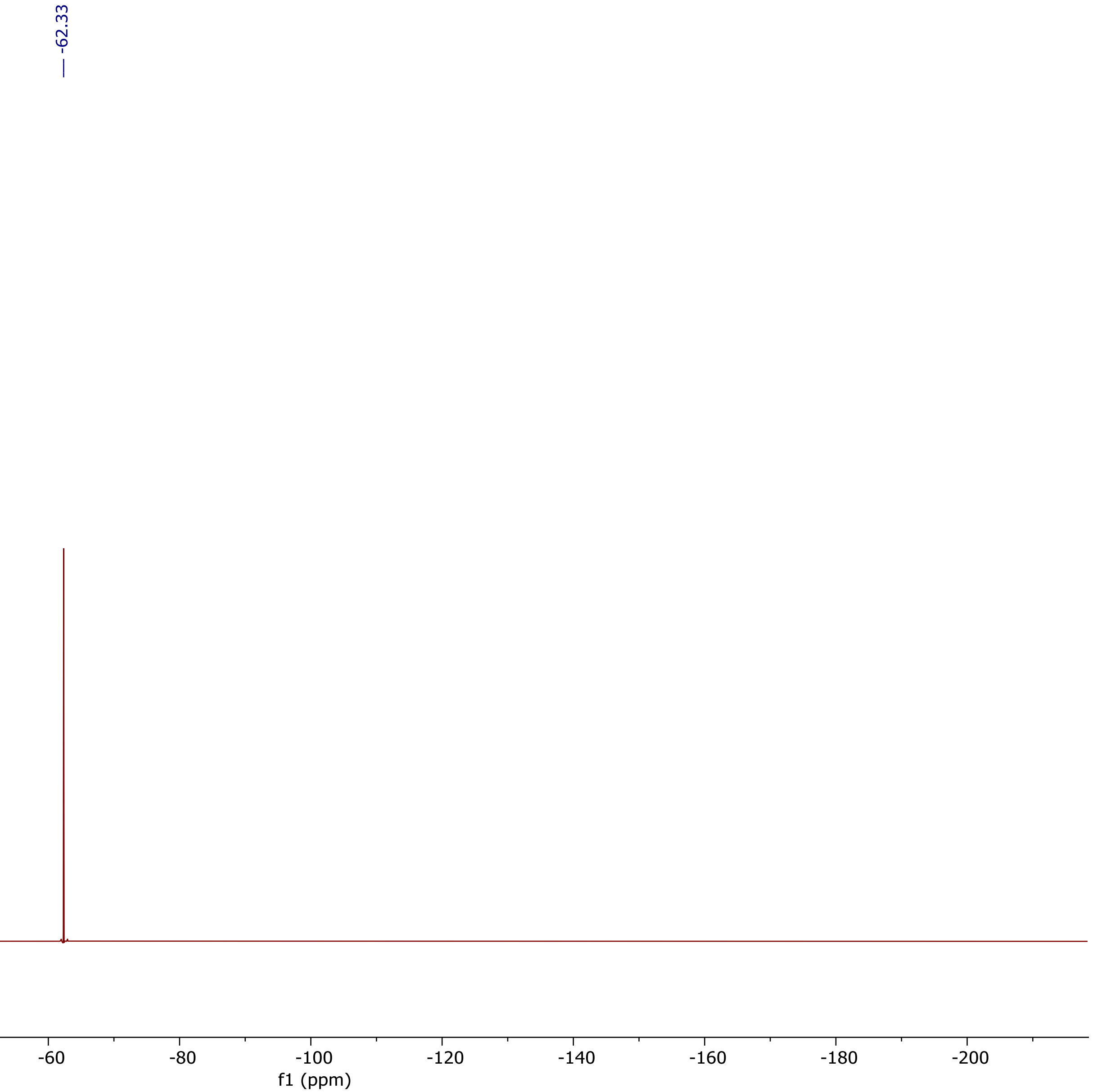

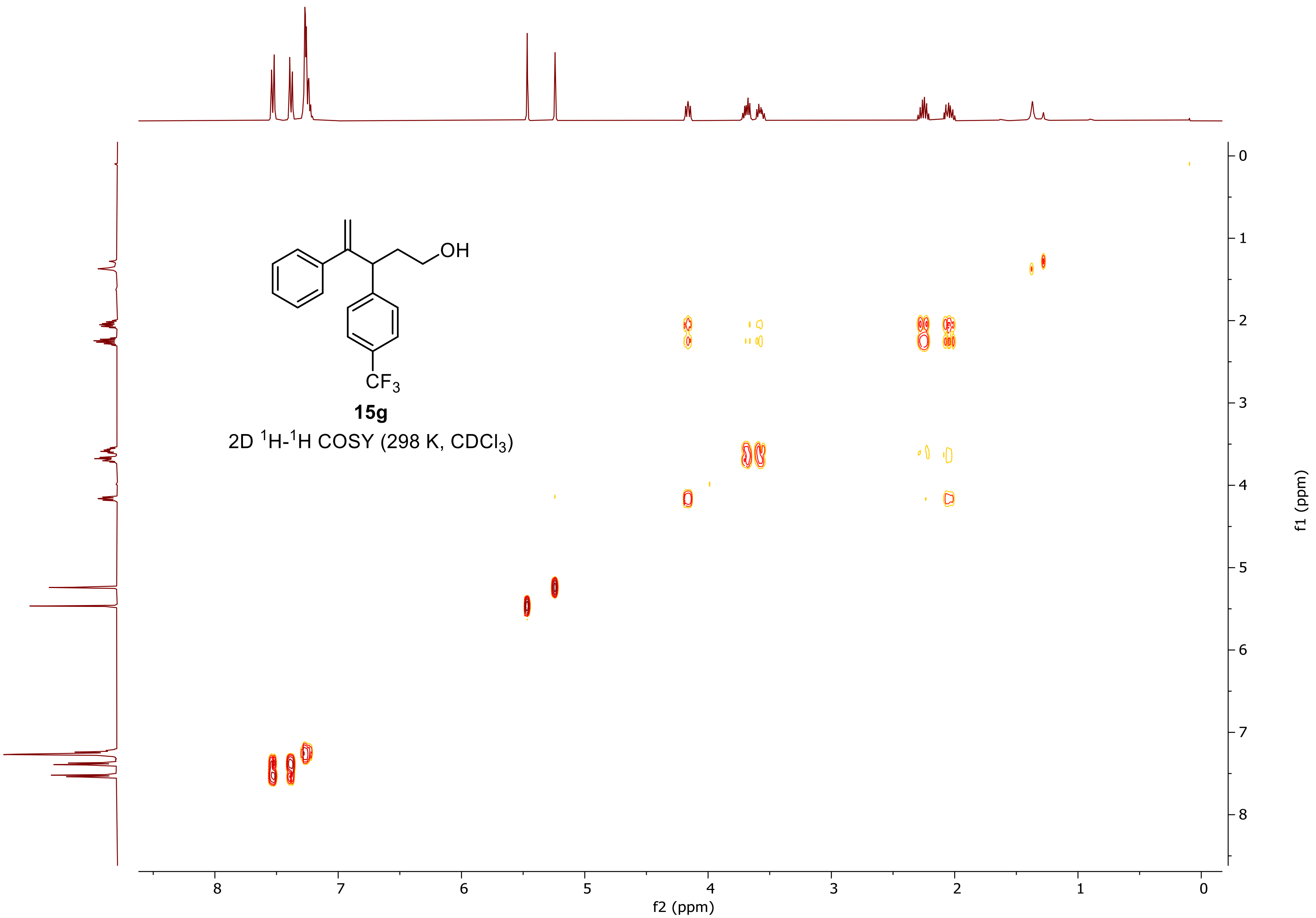
OH
CF_3
15g
2D ^1H-^1H COSY (298 K, $CDCl_3$)
f2 (ppm)
f1 (ppm)
8
7
6
5
4
3
2
1
0

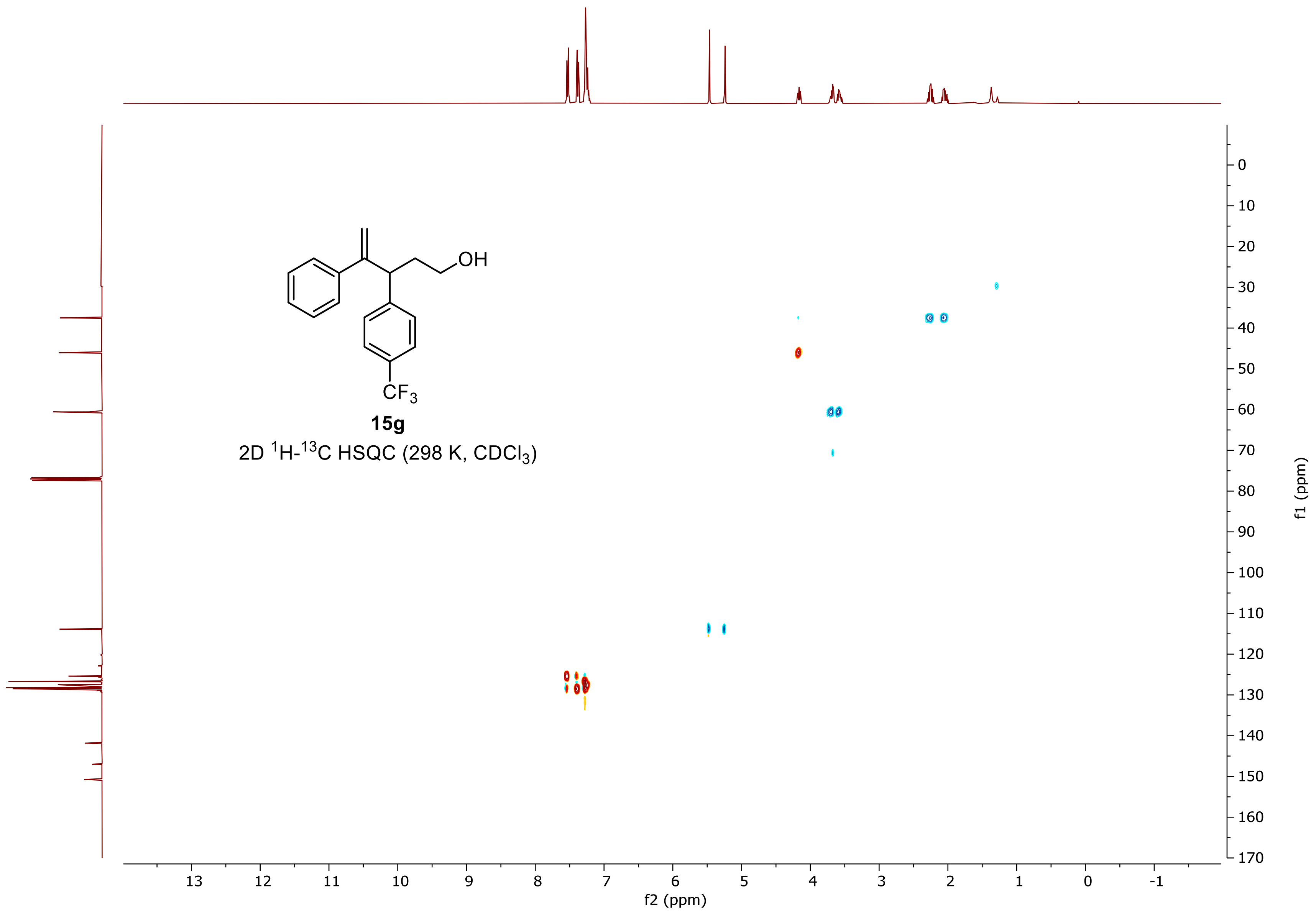

15g

2D ^{1}H-^{13}C HSQC (298 K, $CDCl_3$)

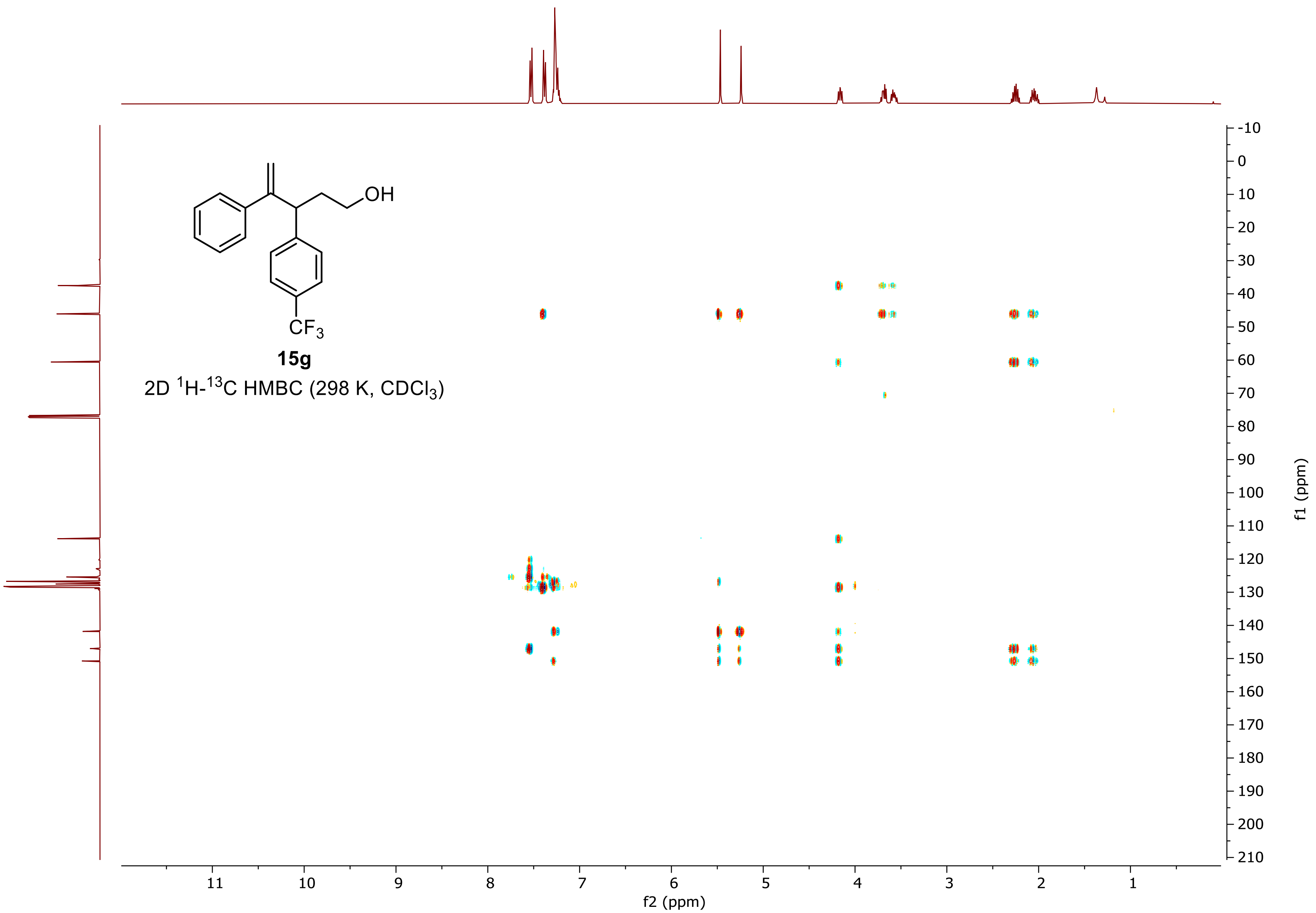
OH
CF3
15g
2D 1H-13C HMBC (298 K, CDCl3)
f1 (ppm)
f2 (ppm)

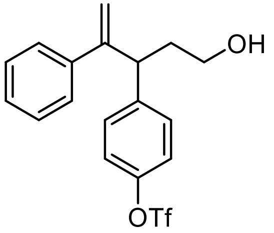

15h

^{1}H NMR (400 MHz, 298 K, $CDCl_3$)

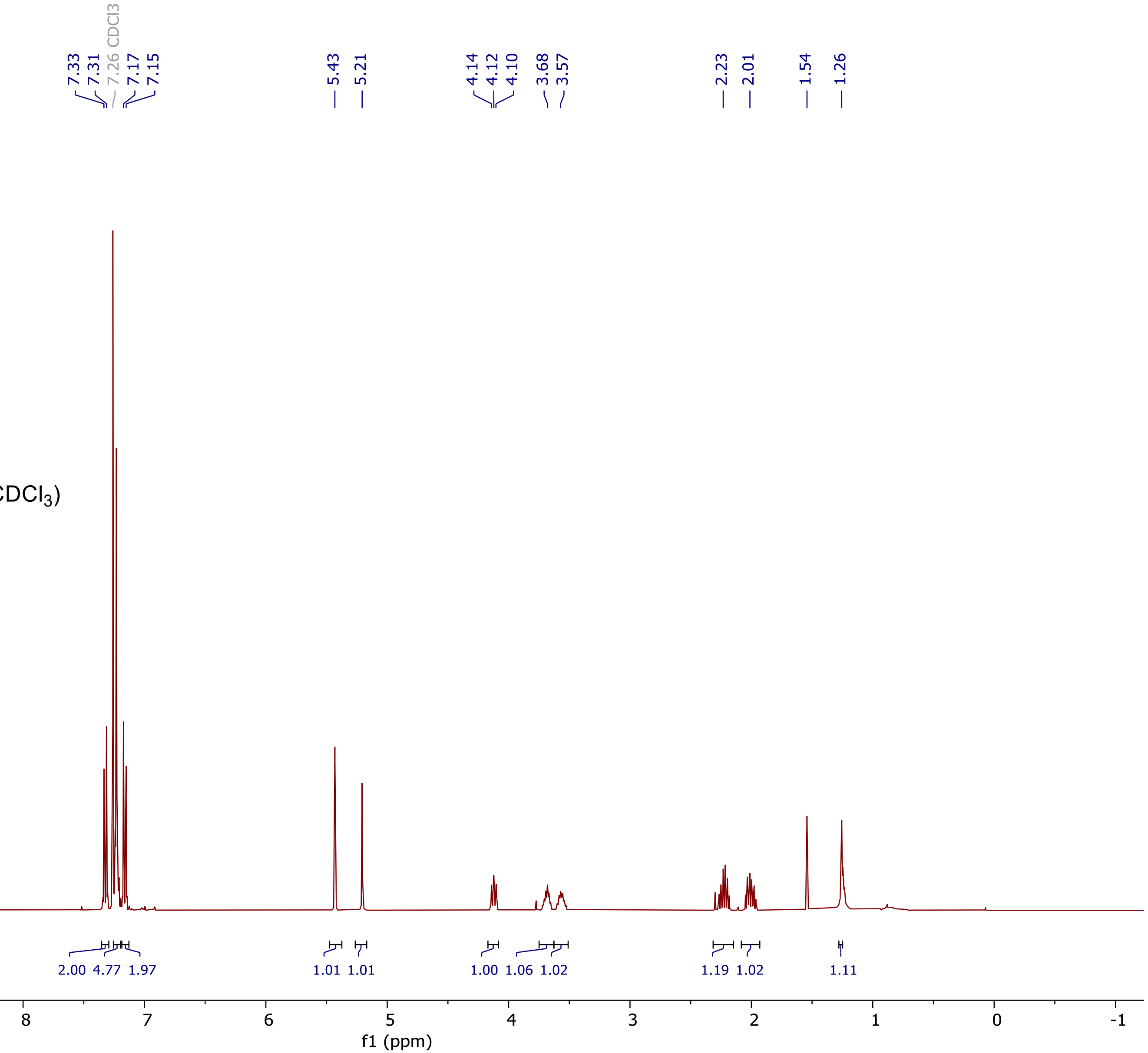

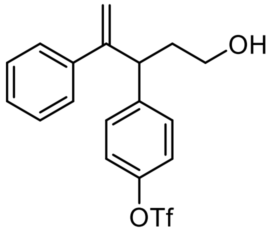

15h

$^{13}C\{^1H\}$ NMR (101 MHz, 298 K, $CDCl_3$)

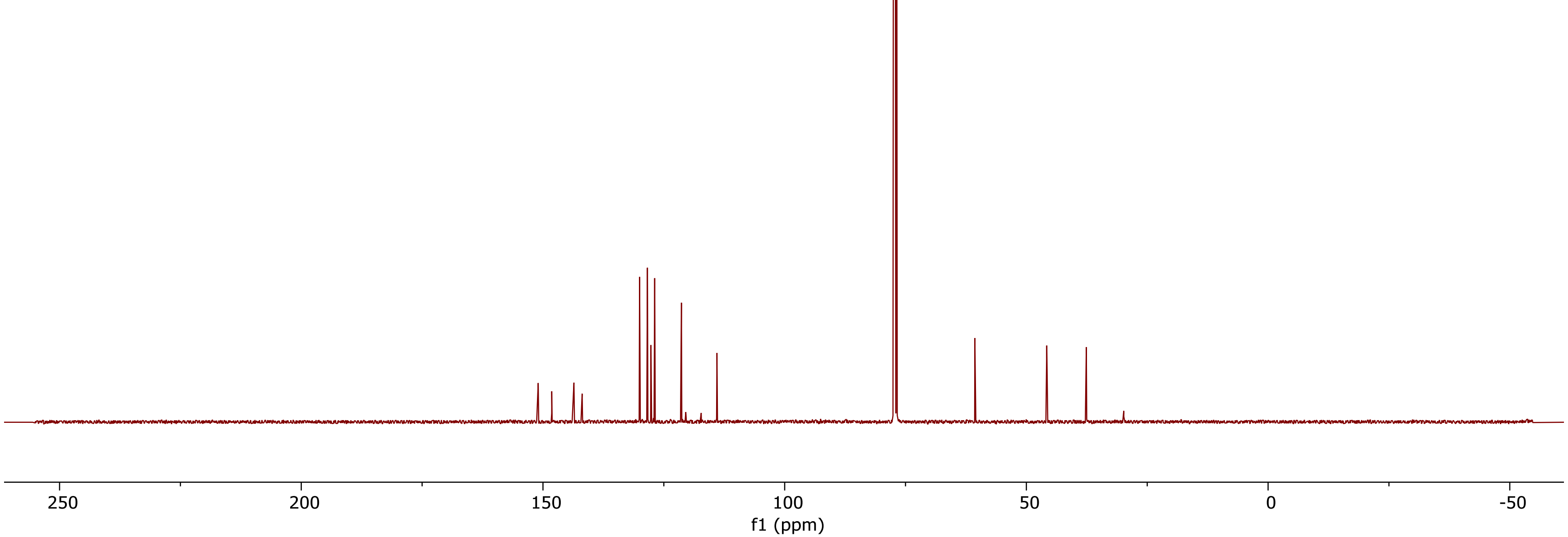

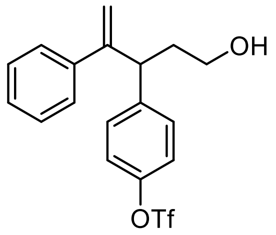

15h

$^{19}F\{^{1}H\}$ NMR (282 MHz, 298 K, $CDCl_3$)

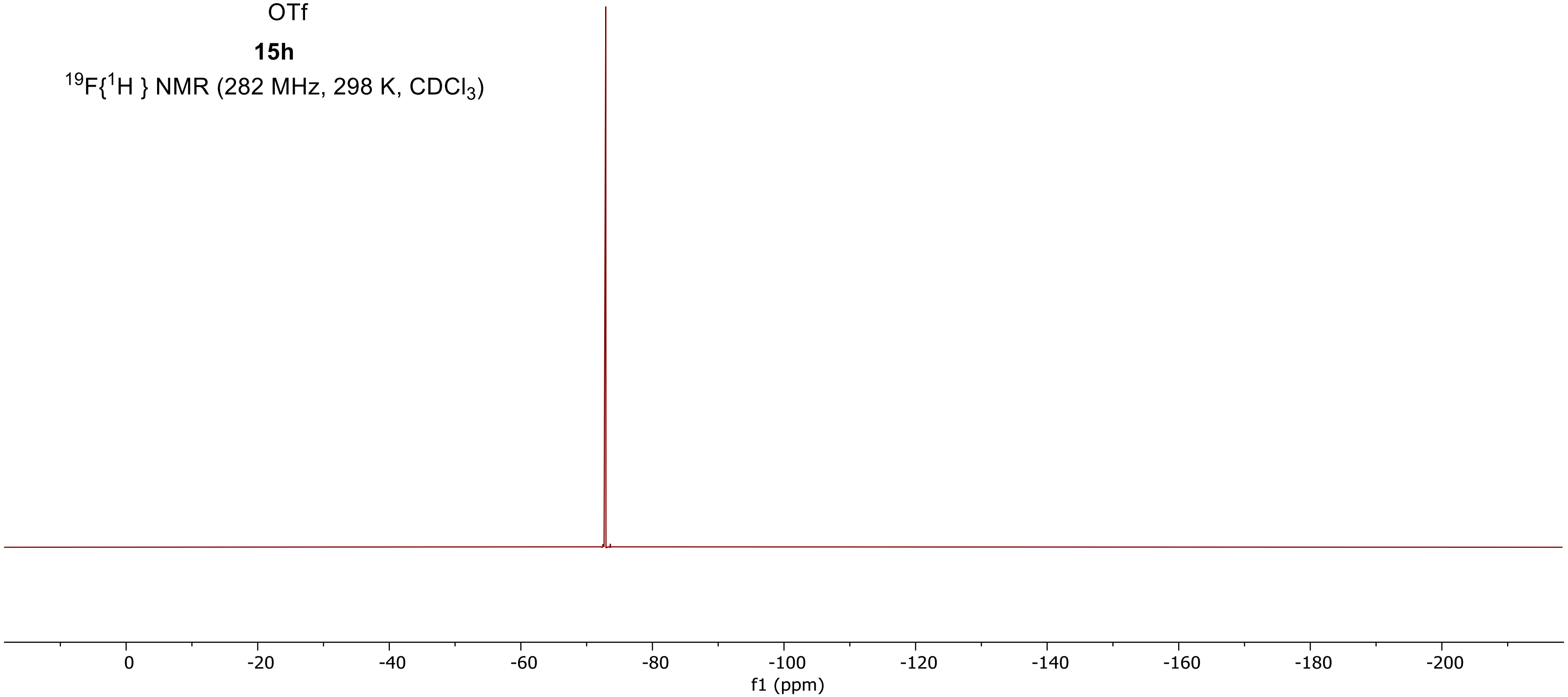

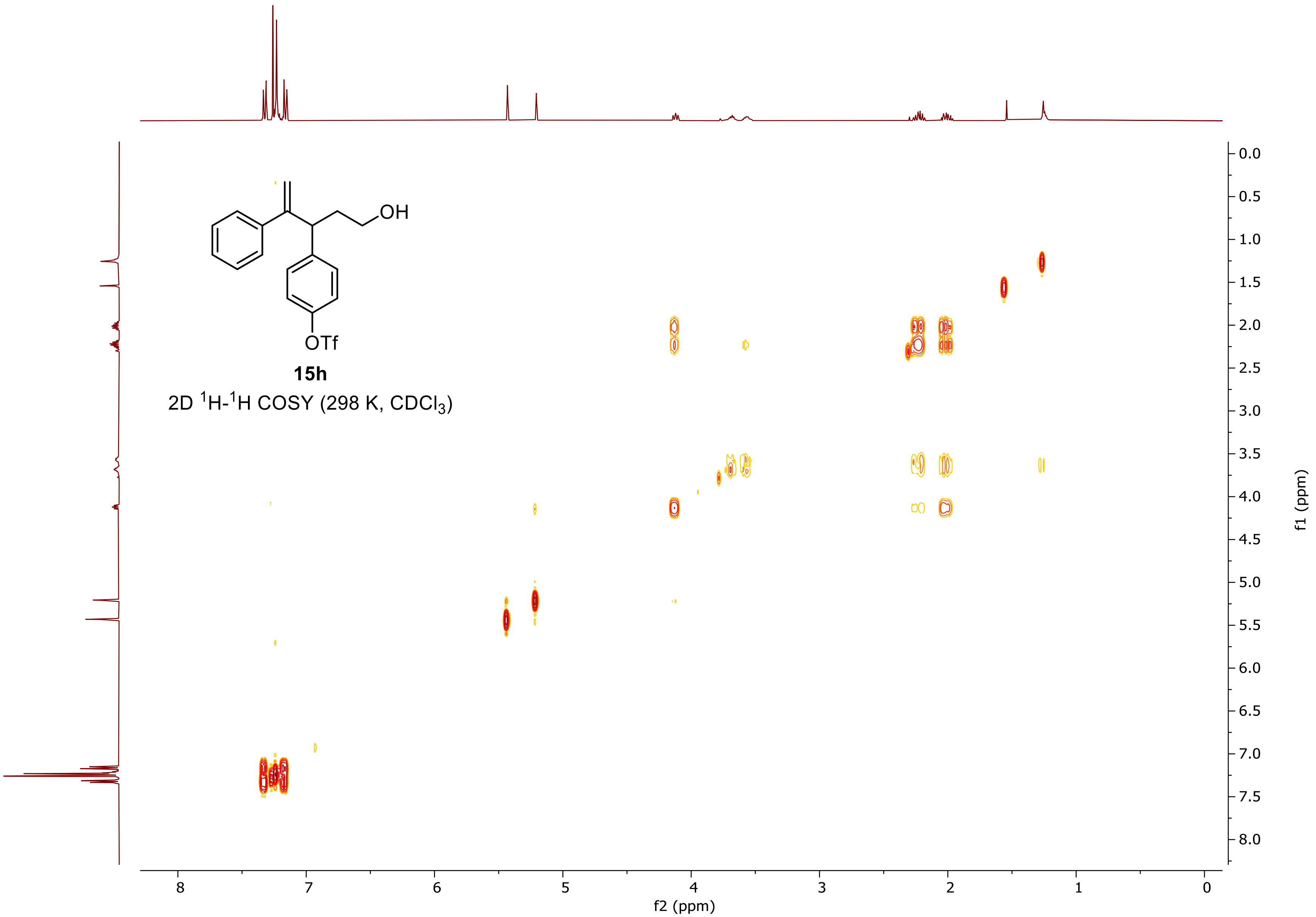

15h

2D ^{1}H-^{1}H COSY (298 K, $CDCl_3$)

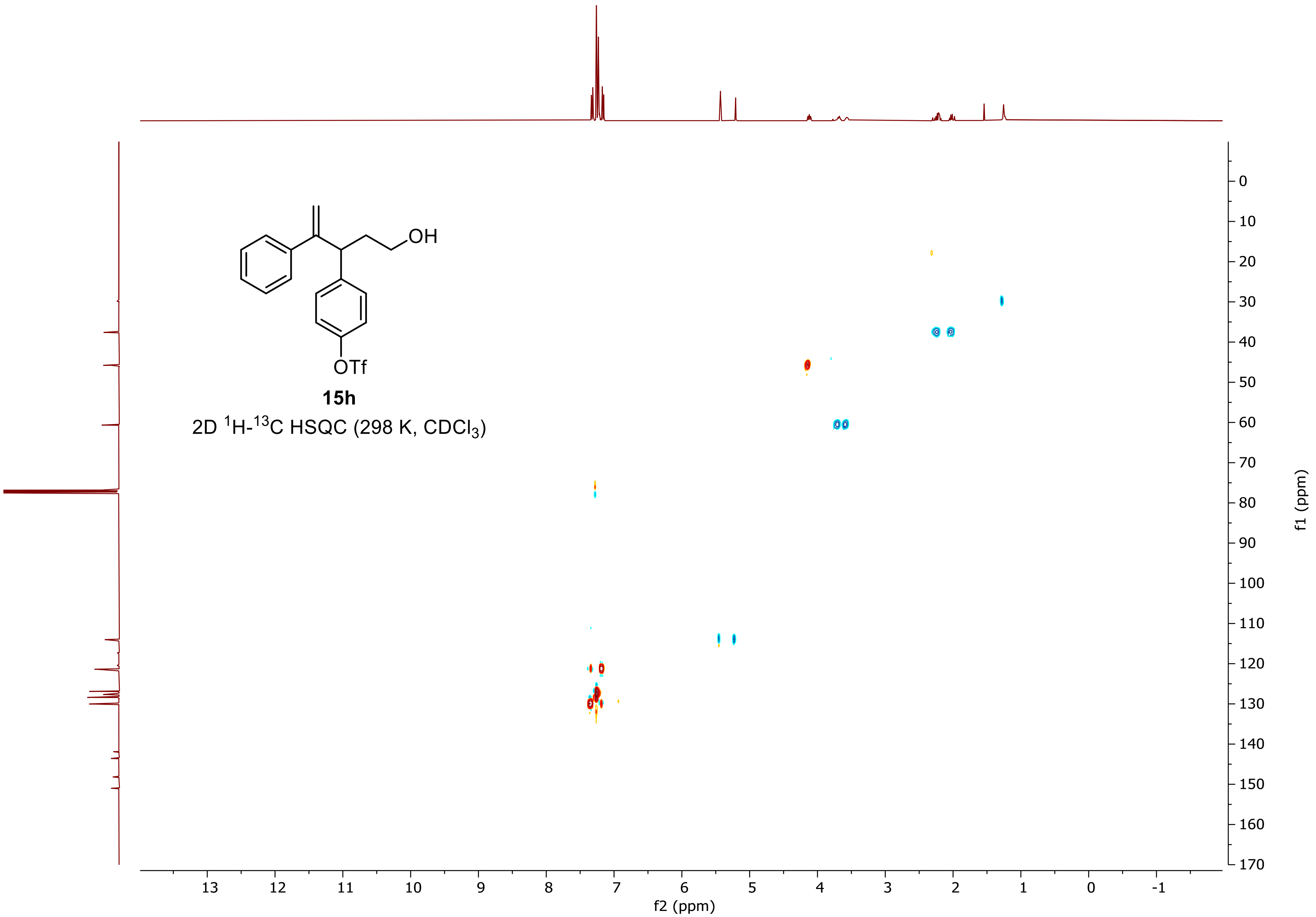
OH
OTf
15h
2D ¹H-¹³C HSQC (298 K, CDCl₃)
f2 (ppm)
f1 (ppm)

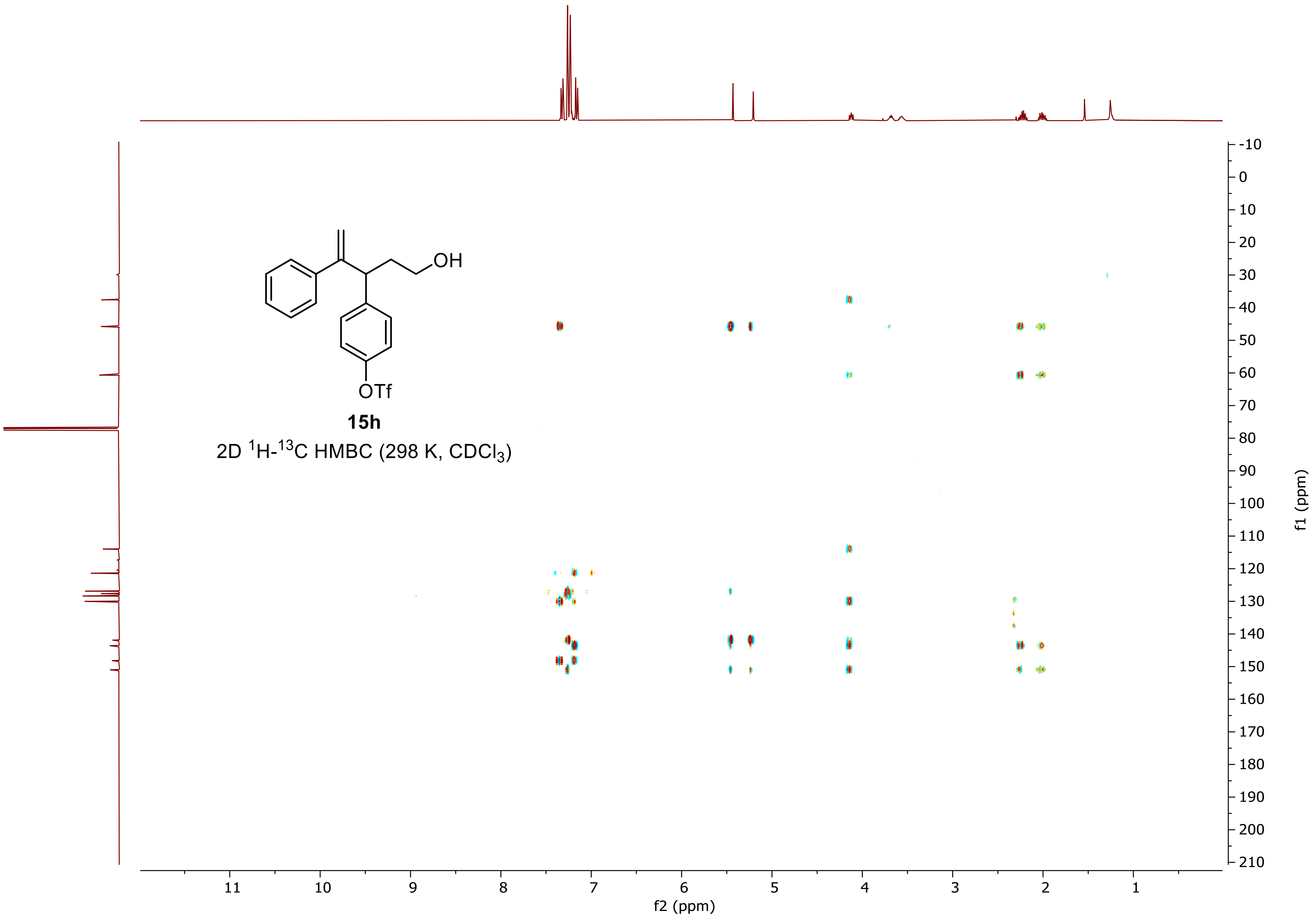

15h

2D ^{1}H-^{13}C HMBC (298 K, $CDCl_3$)

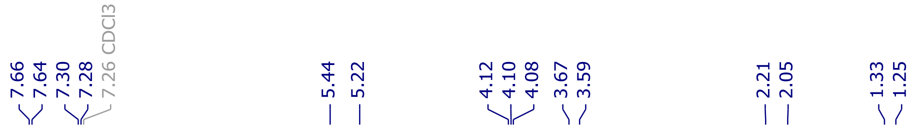

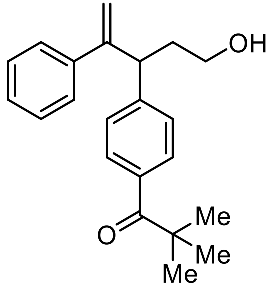

15i

^{1}H NMR (400 MHz, 298 K, $CDCl_3$)

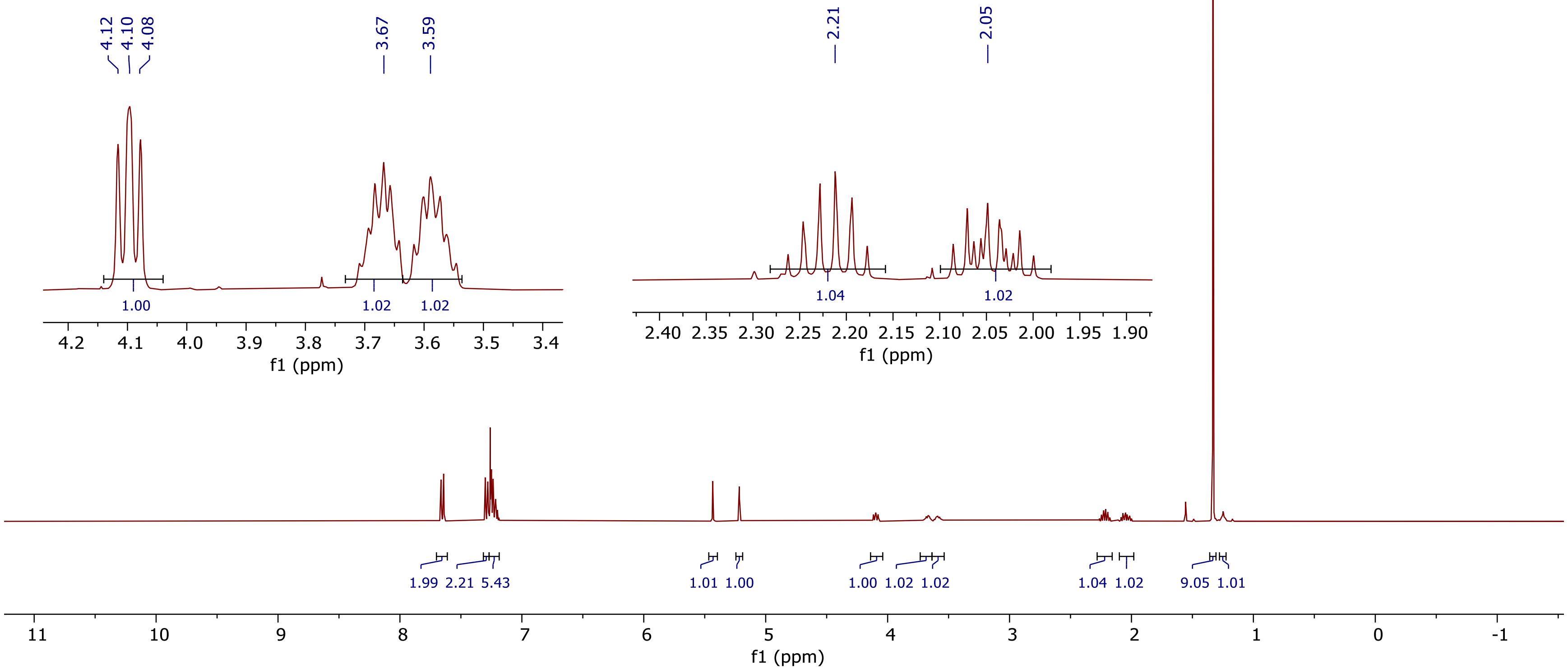

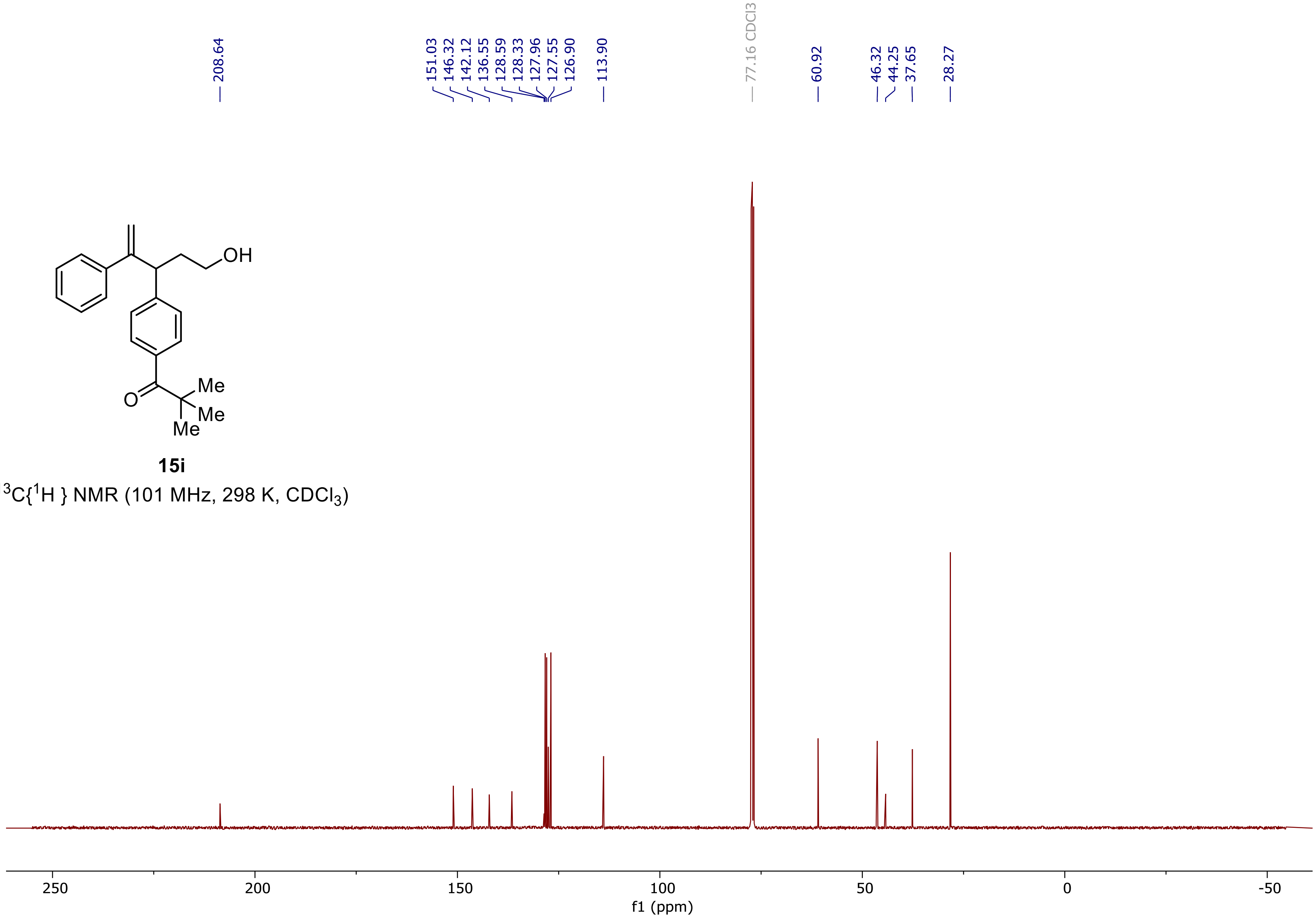

15i

$^{13}C\{^{1}H\}$ NMR (101 MHz, 298 K, $CDCl_3$)

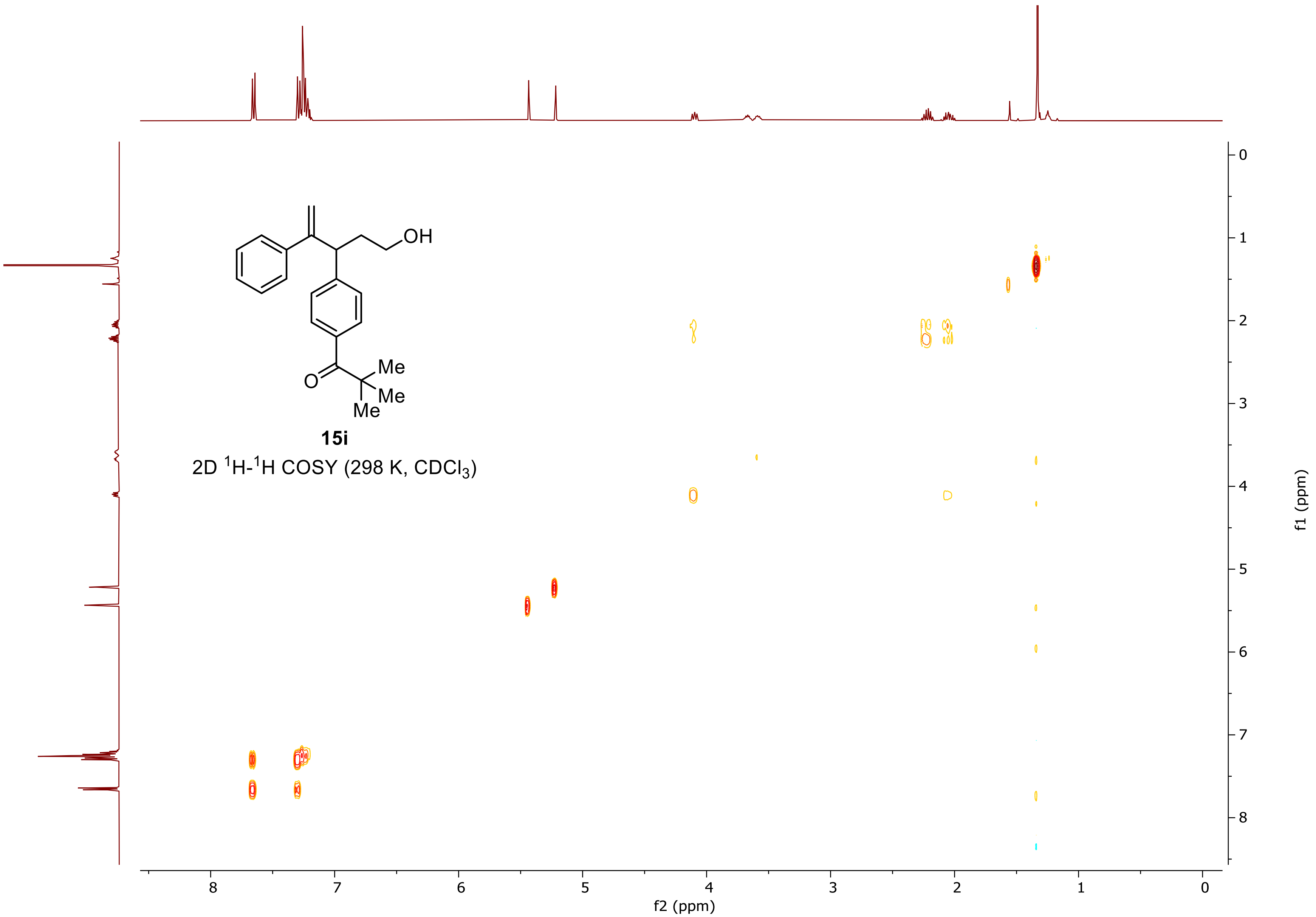

15i

2D ^{1}H-^{1}H COSY (298 K, $CDCl_3$)

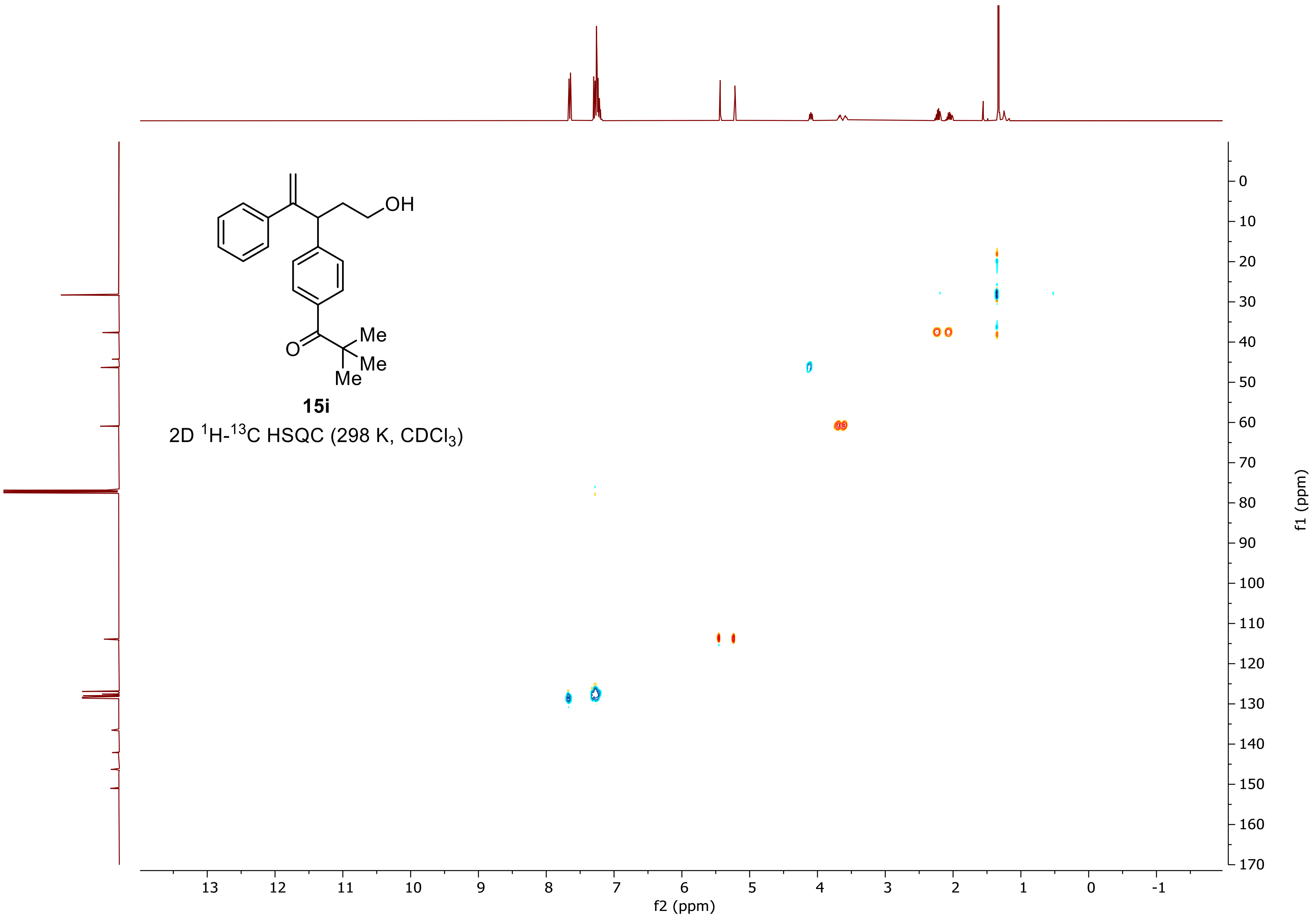

15i

2D ^{1}H-^{13}C HSQC (298 K, $CDCl_3$)

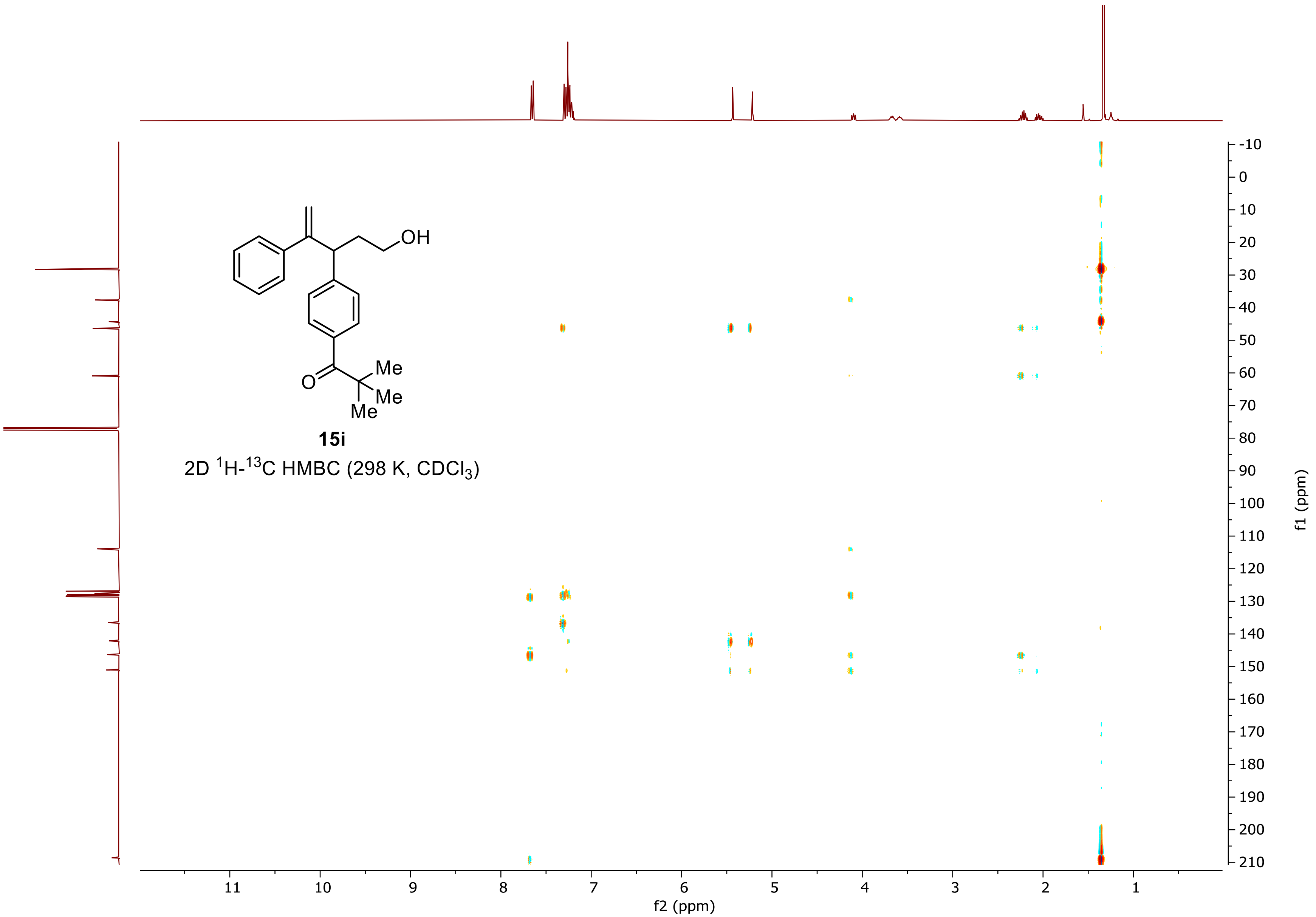
OH
O
Me
Me
Me
15i
2D 1H-13C HMBC (298 K, CDCl3)
f1 (ppm)
f2 (ppm)

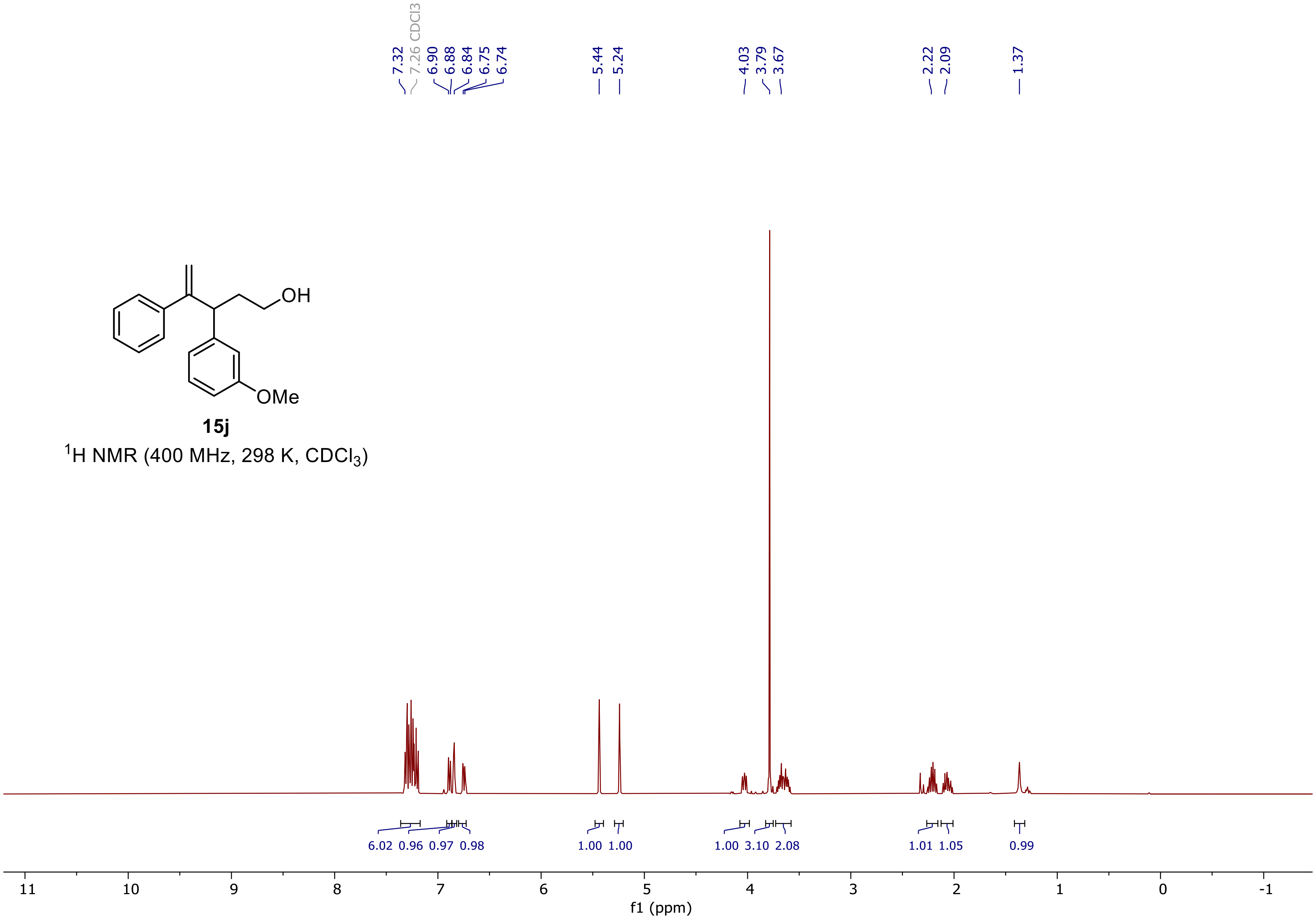

15j

^{1}H NMR (400 MHz, 298 K, $CDCl_3$)

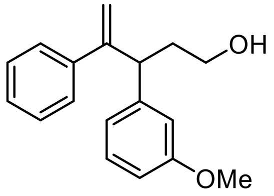

15j

$^{13}C\{^{1}H\}$ NMR (101 MHz, 298 K, $CDCl_3$)

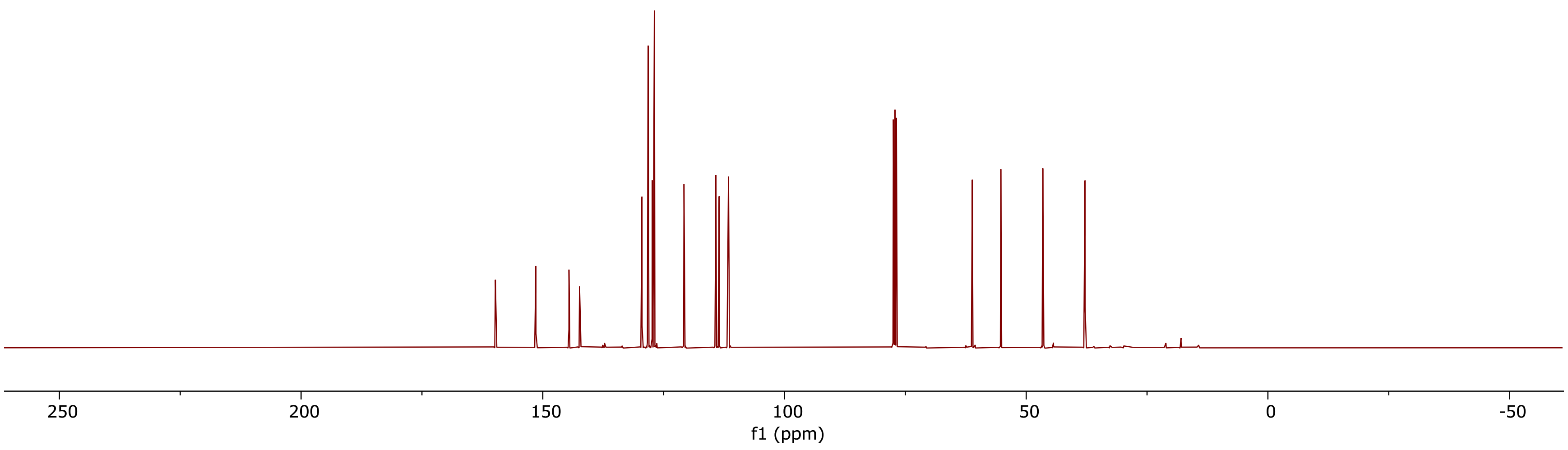

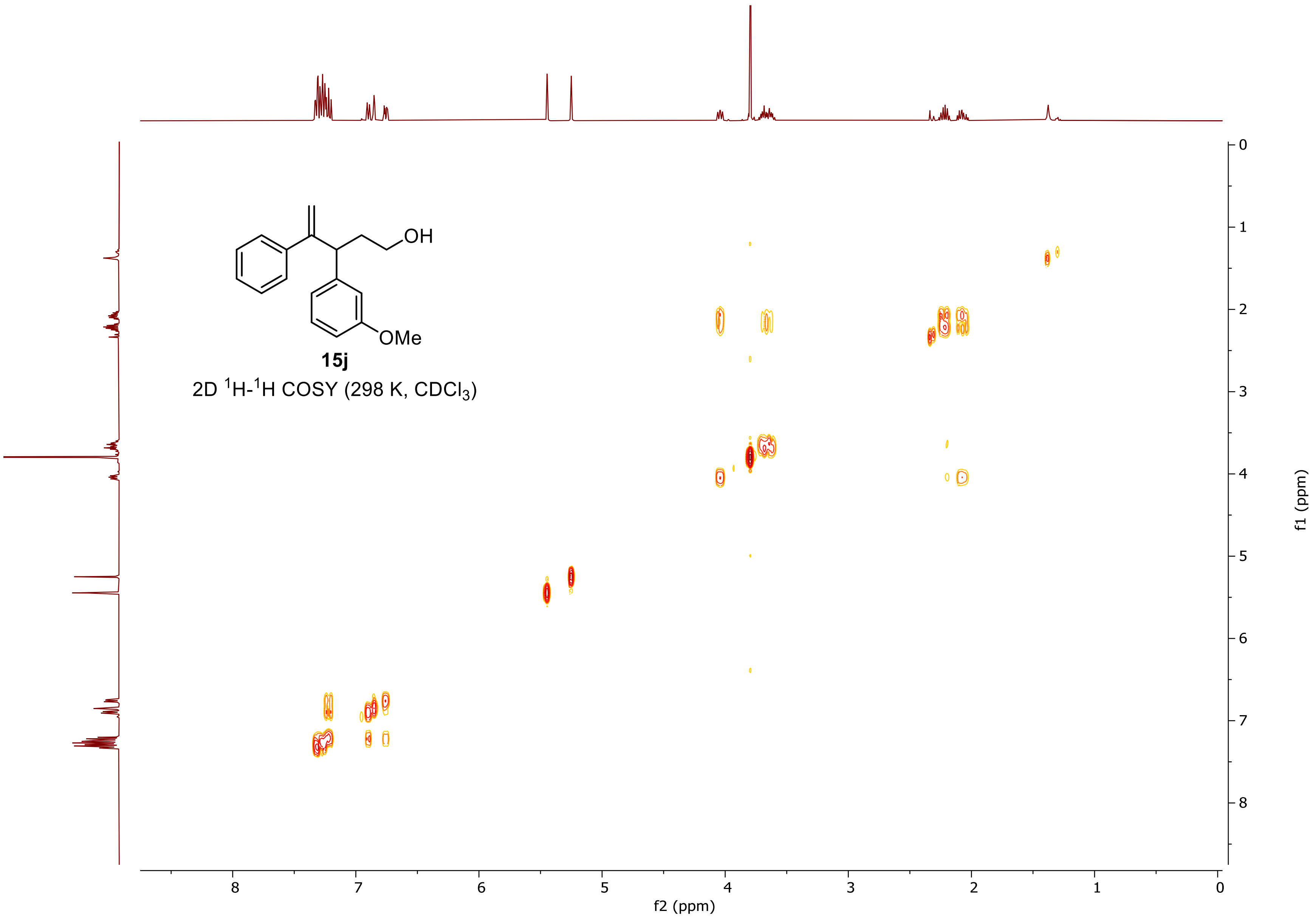
OH
OMe
15j
2D 1H-1H COSY (298 K, CDCl3)
f2 (ppm)
f1 (ppm)

15j

2D ^{1}H-^{13}C HSQC (298 K, $CDCl_3$)

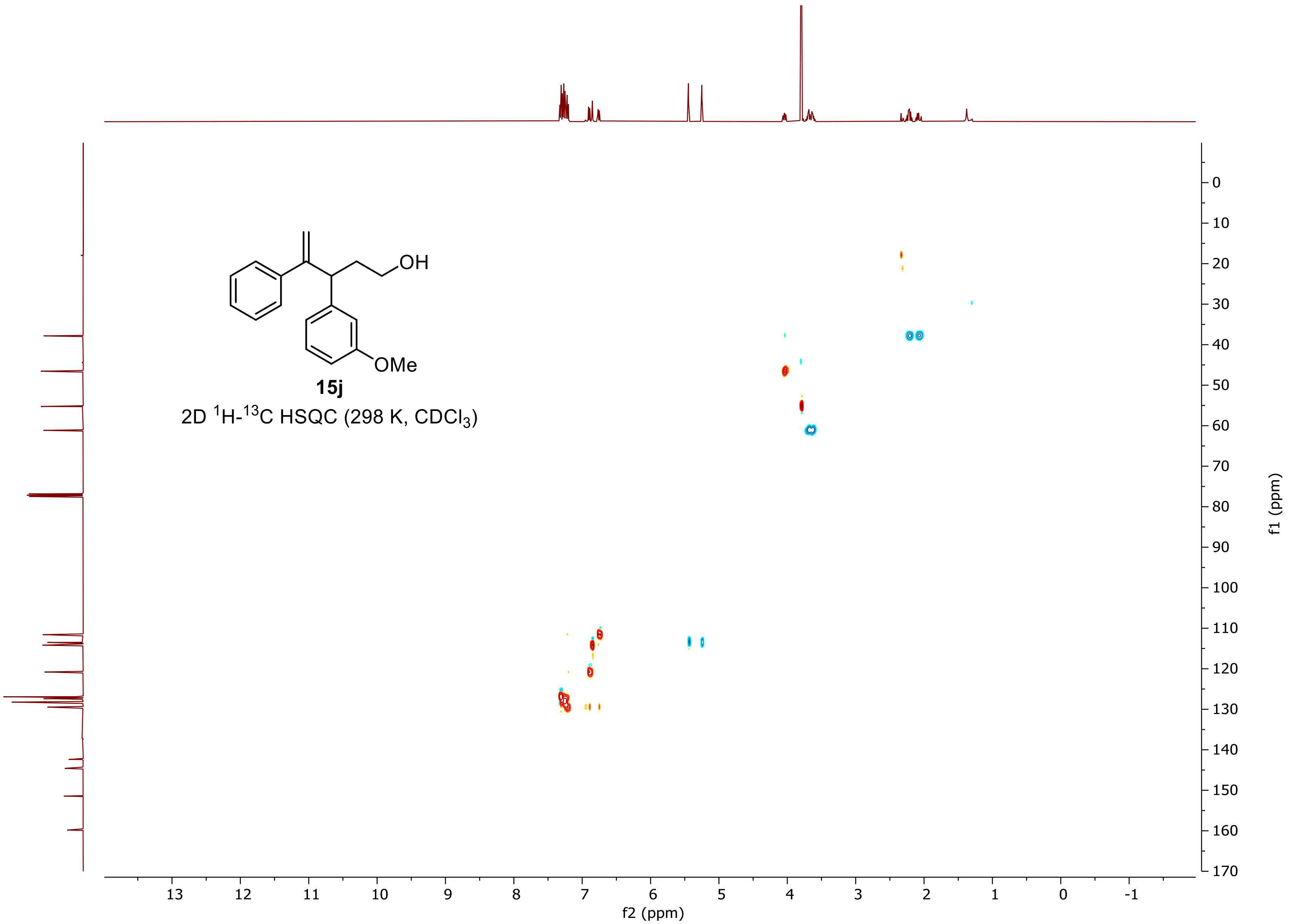

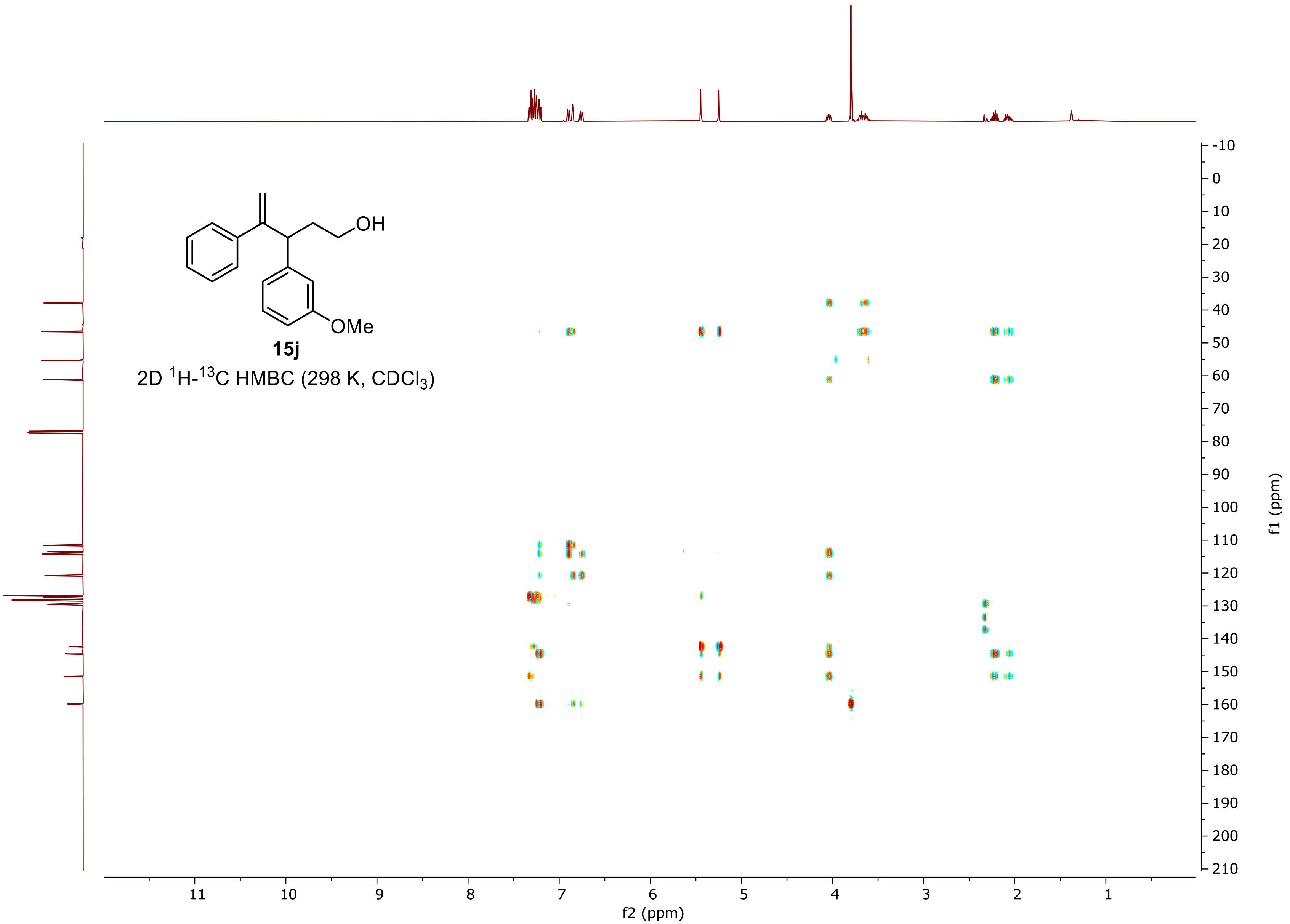

15j

2D ^{1}H-^{13}C HMBC (298 K, $CDCl_3$)

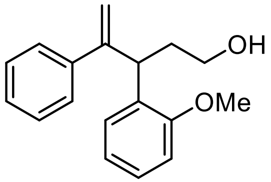

15k

^{1}H NMR (400 MHz, 298 K, $CDCl_3$)

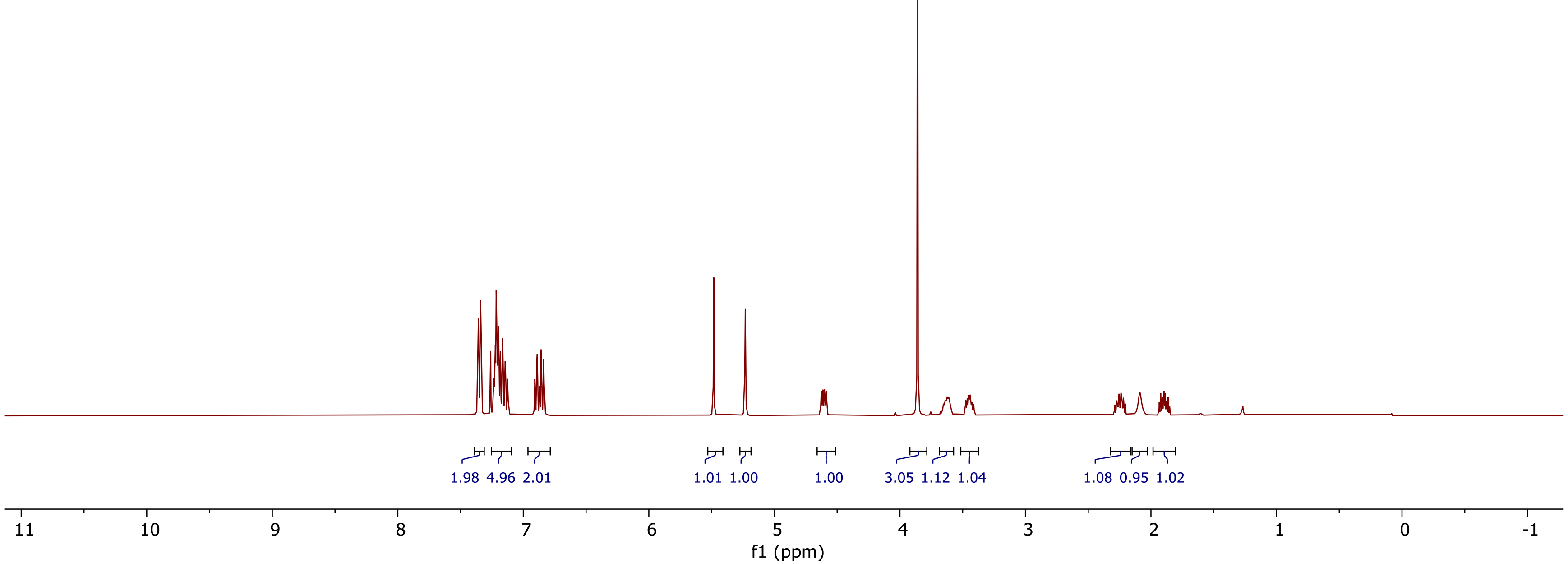

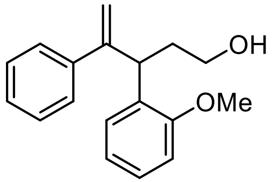

15k

$^{13}C\{^1H\}$ NMR (101 MHz, 298 K, $CDCl_3$)

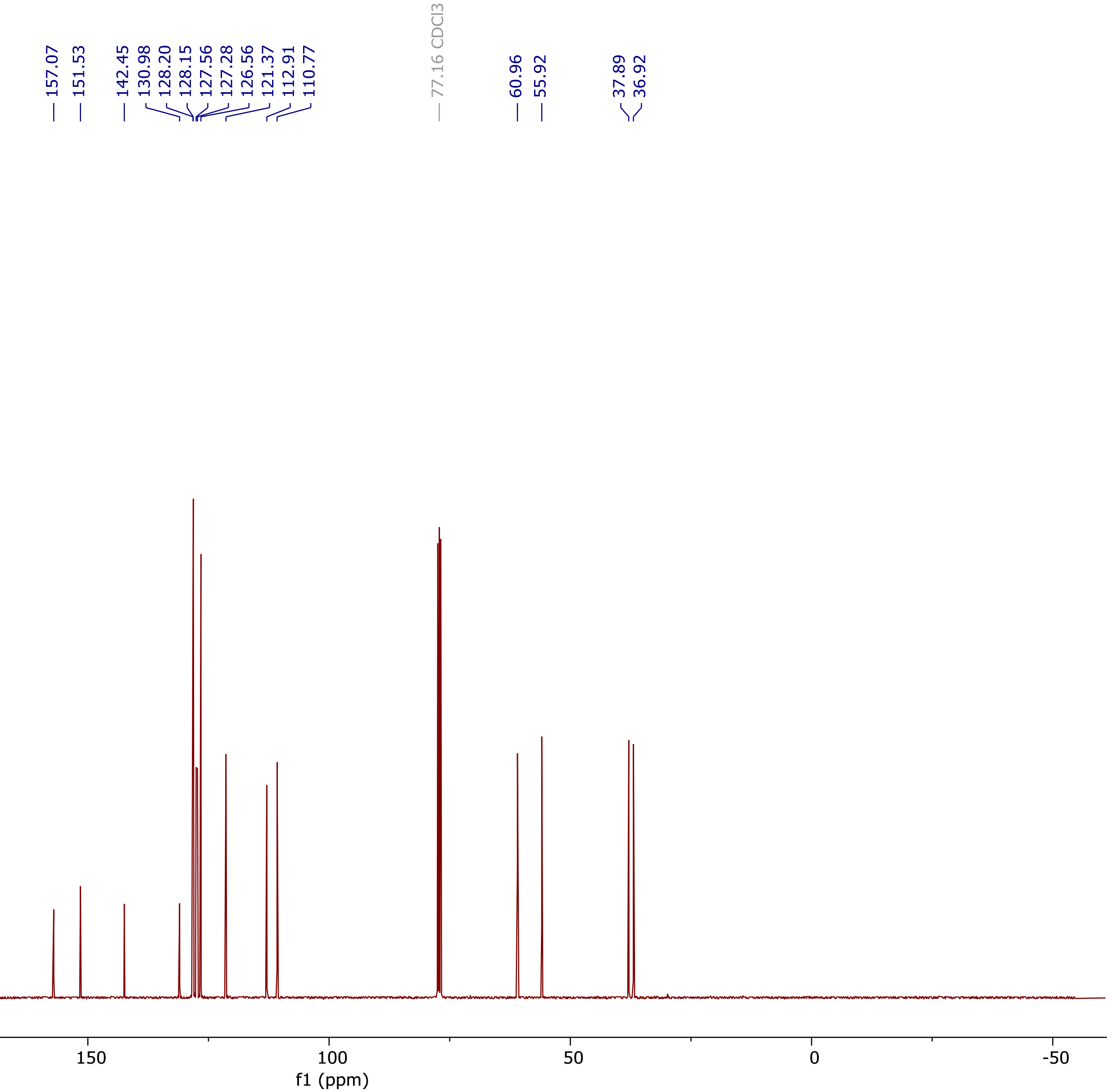

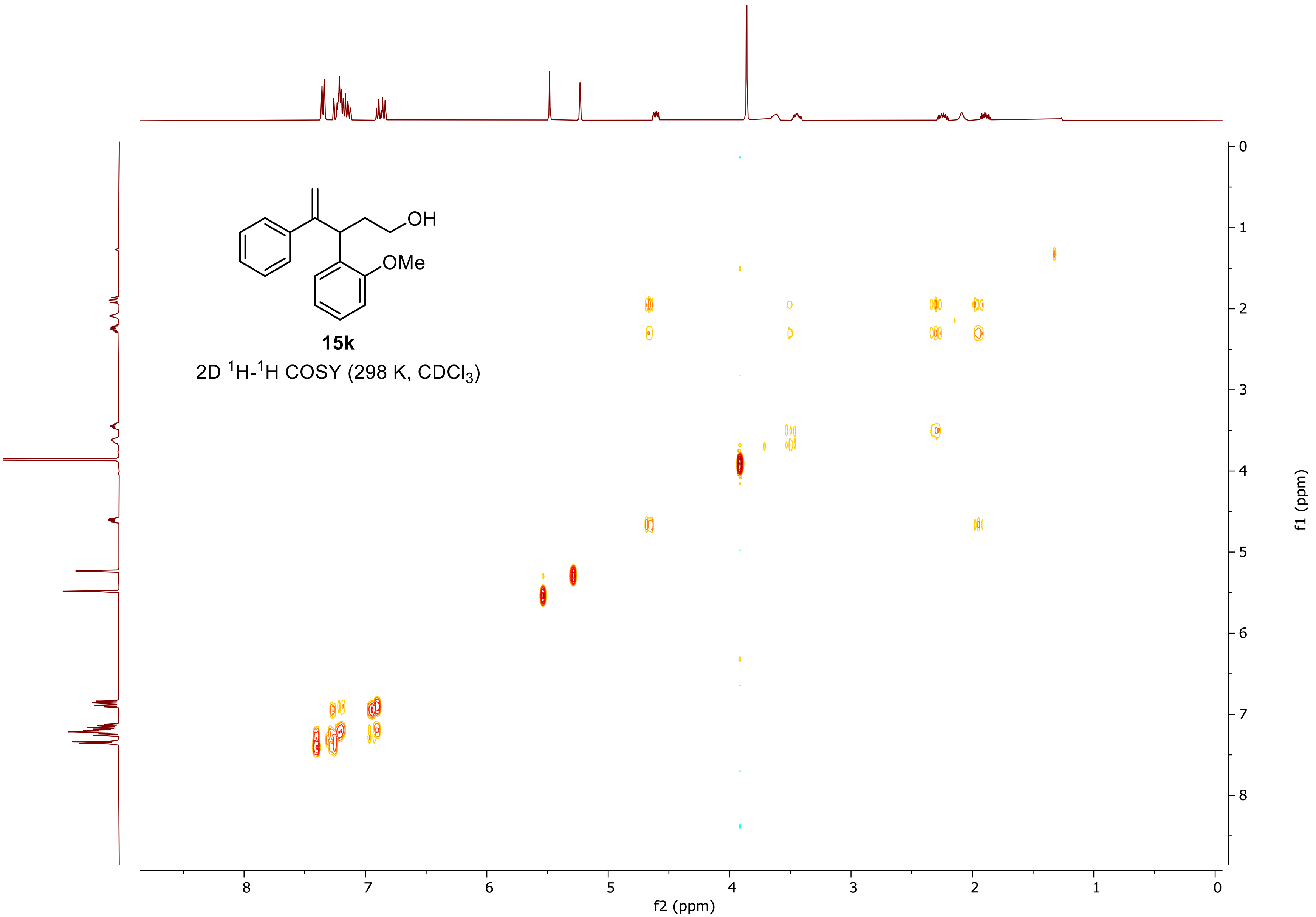
OH
OMe
15k
2D ¹H-¹H COSY (298 K, CDCl₃)
f1 (ppm)
f2 (ppm)
8
7
6
5
4
3
2
1
0

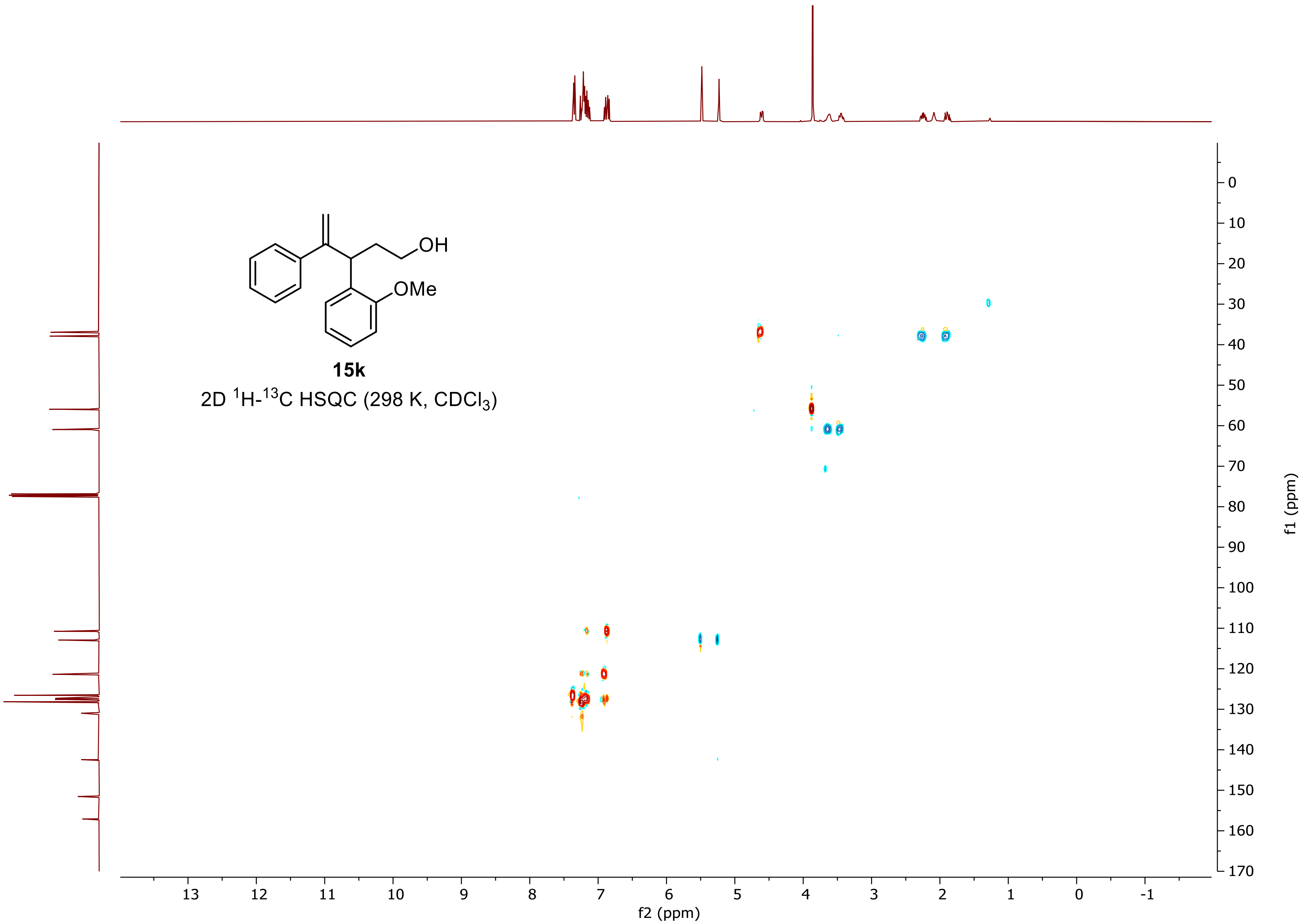

15k

2D ^{1}H-^{13}C HSQC (298 K, $CDCl_3$)

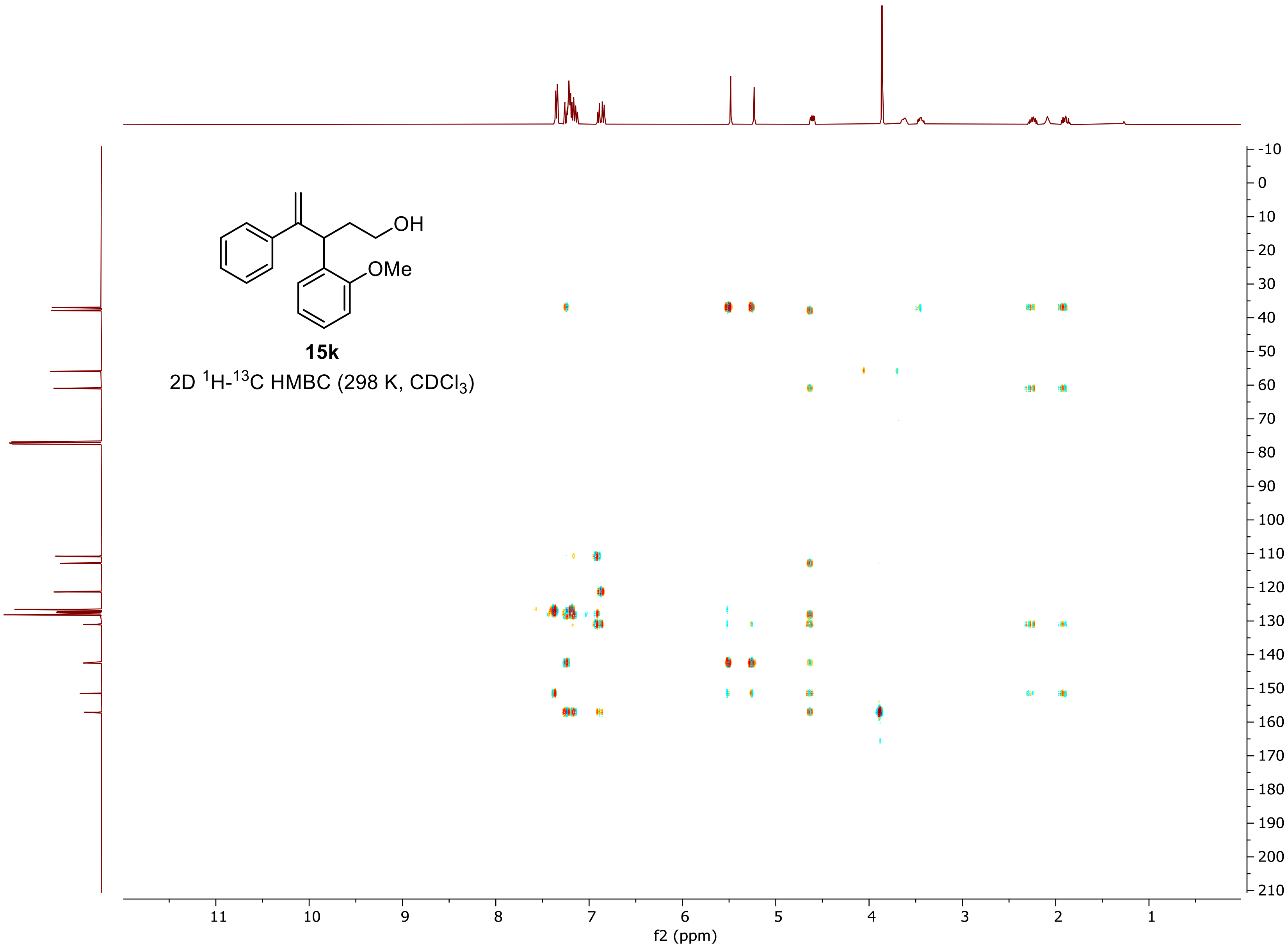

15k

2D ^{1}H-^{13}C HMBC (298 K, $CDCl_3$)

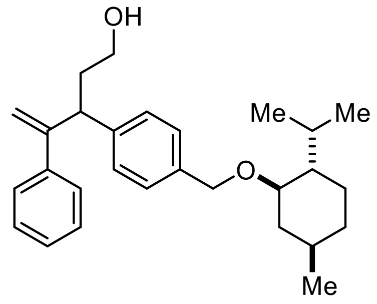

15l

$^{13}C\{^1H\}$ NMR (101 MHz, 298 K, $CDCl_3$)

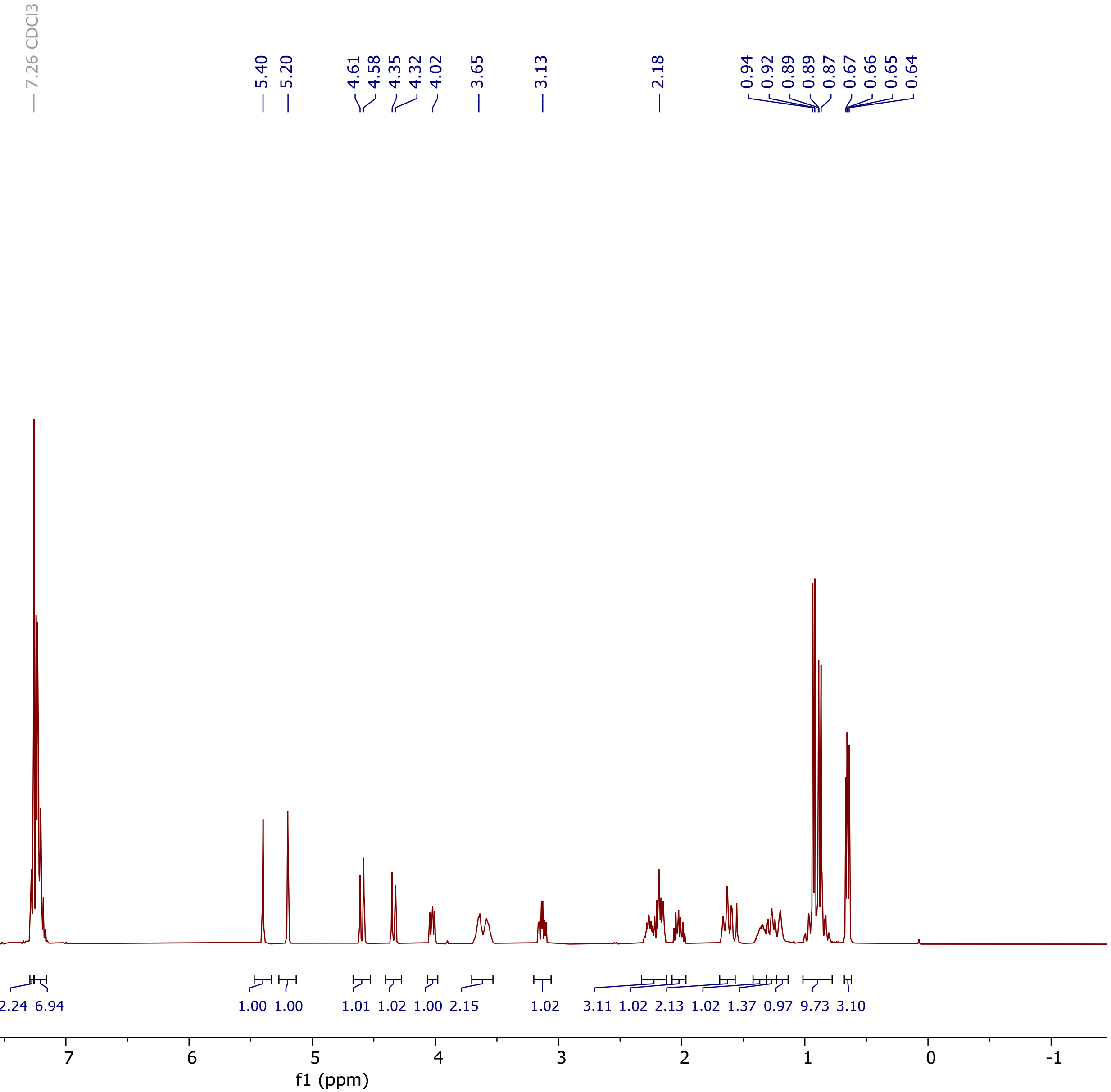

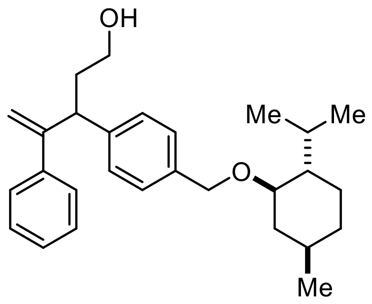

15l

$^{13}C\{^{1}H\}$ NMR (101 MHz, 298 K, $CDCl_3$)

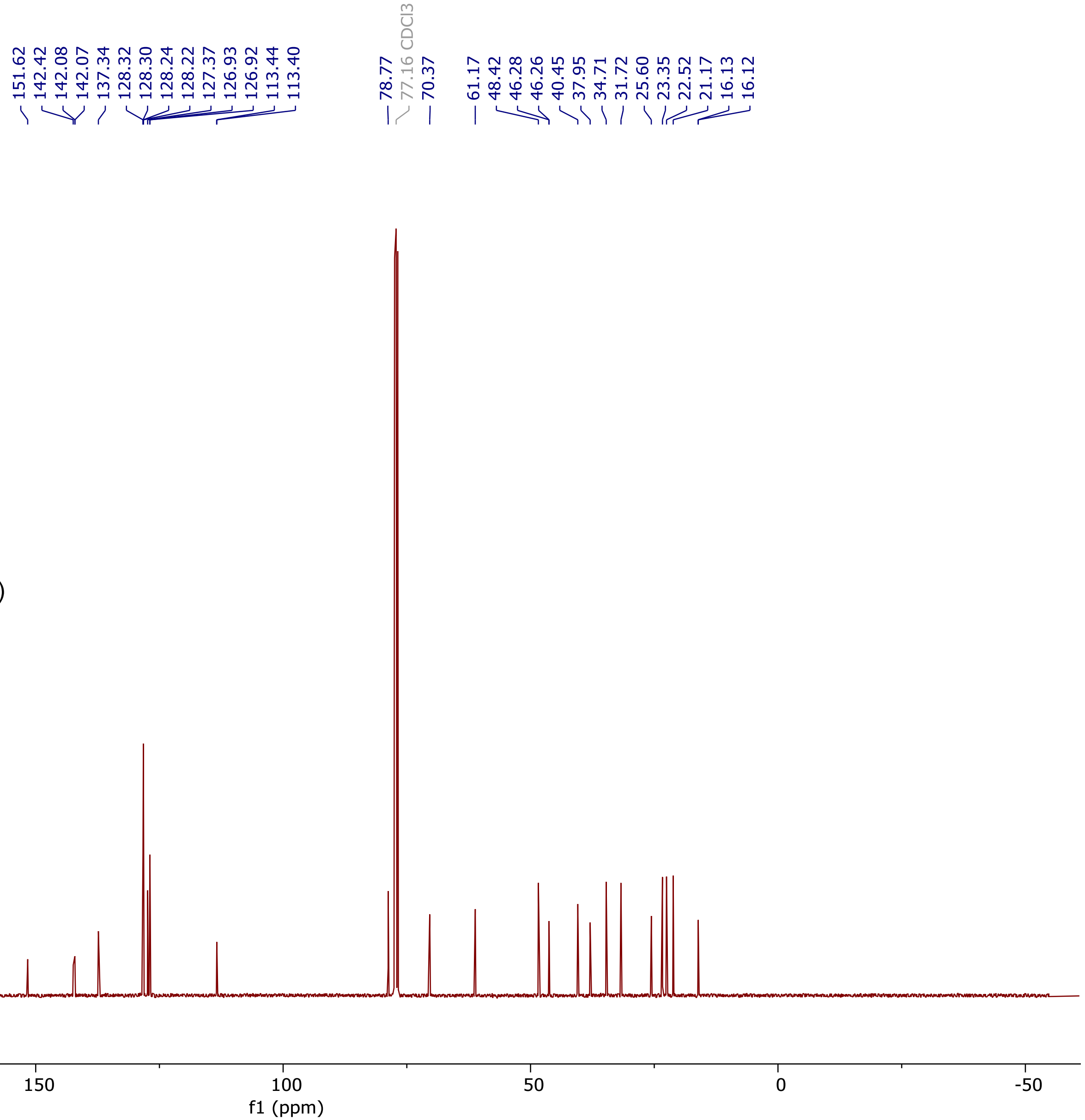

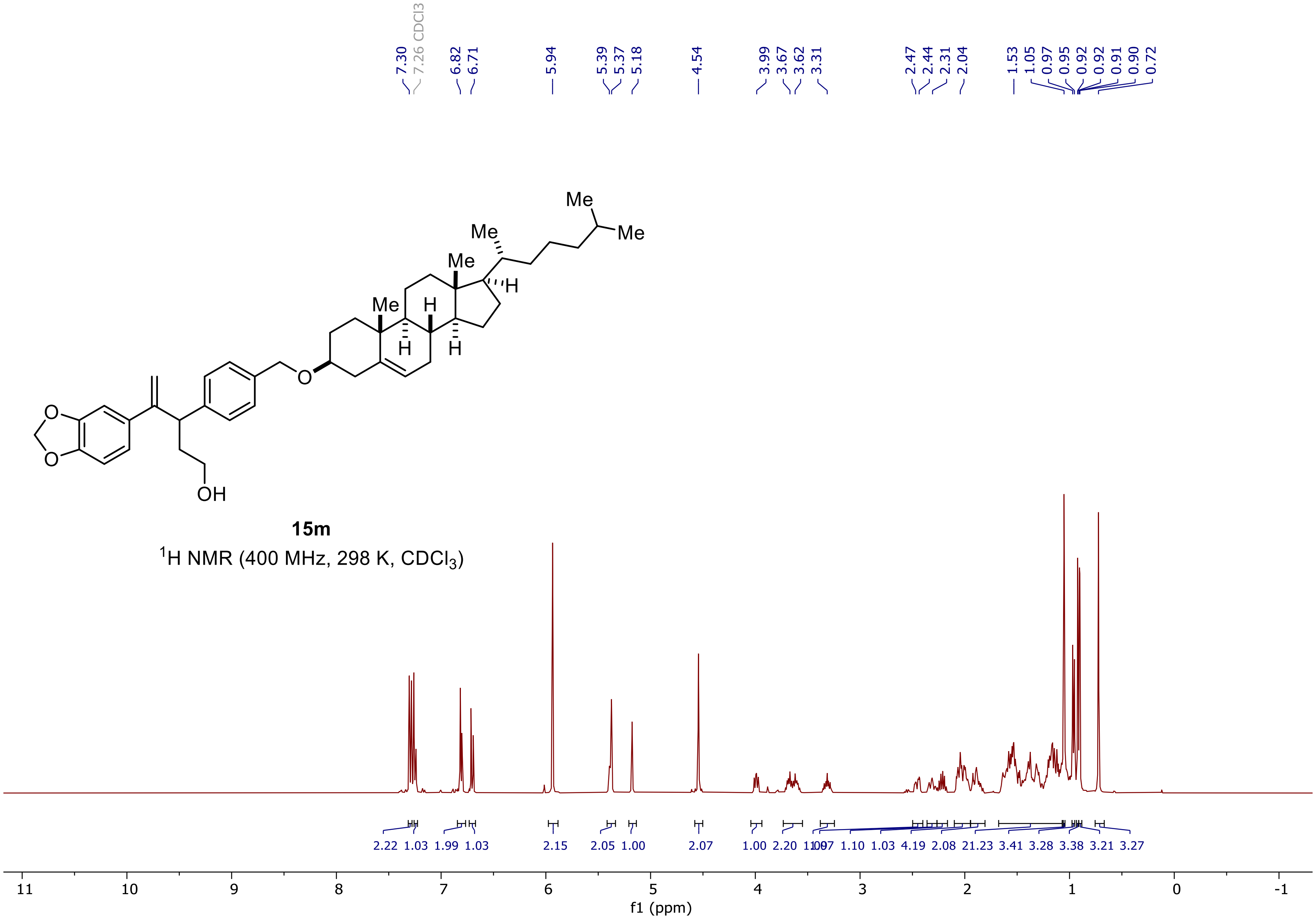
Me
Me
Me
Me
Me
H
Me
H
H
H
O
O
O
OH
15m
^{1}H NMR (400 MHz, 298 K, CDCl3)
7.30
7.26 CDCl3
6.82
6.71
5.94
5.39
5.37
5.18
4.54
3.99
3.67
3.62
3.31
2.47
2.44
2.31
2.04
1.53
1.05
0.97
0.95
0.92
0.92
0.91
0.90
0.72
2.22 1.03 1.99 1.03 2.15 2.05 1.00 2.07 1.00 2.20 1.07 1.10 1.03 4.19 2.08 21.23 3.41 3.28 3.38 3.21 3.27
11 10 9 8 7 6 5 4 3 2 1 0 -1
f1 (ppm)

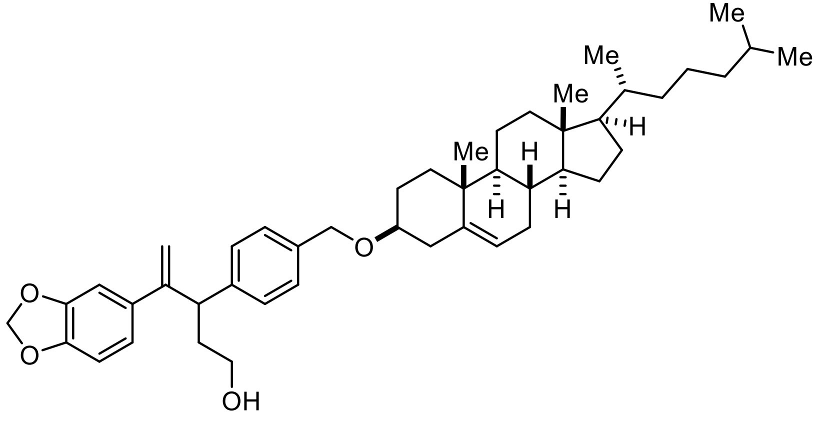

15m

$^{13}C\{^1H\}$ NMR (101 MHz, 298 K, $CDCl_3$)

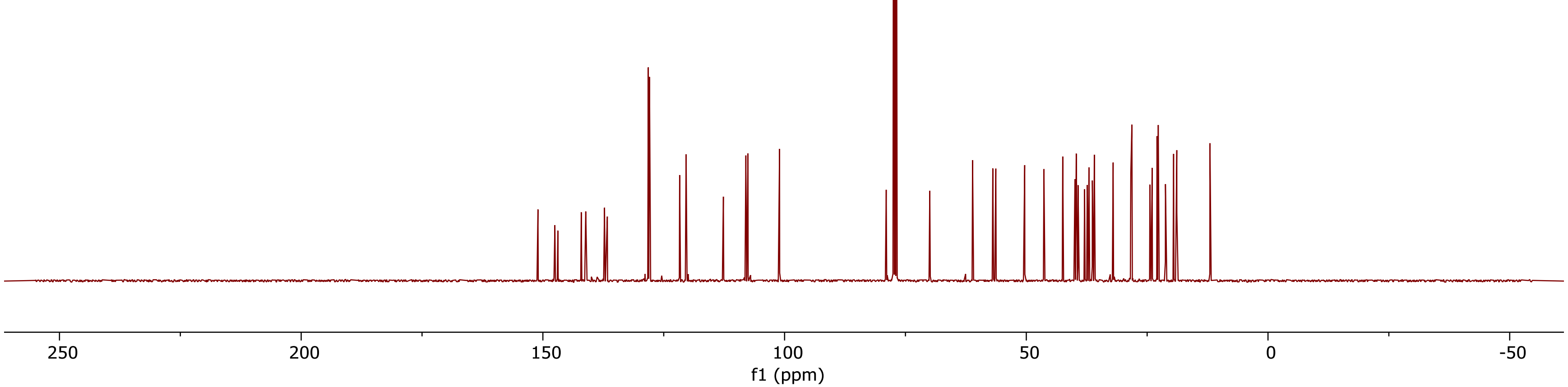

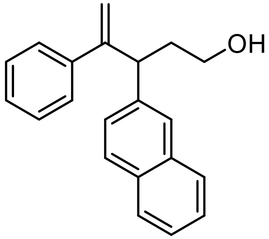

15n

^1H NMR (400 MHz, 298 K, $CDCl_3$)

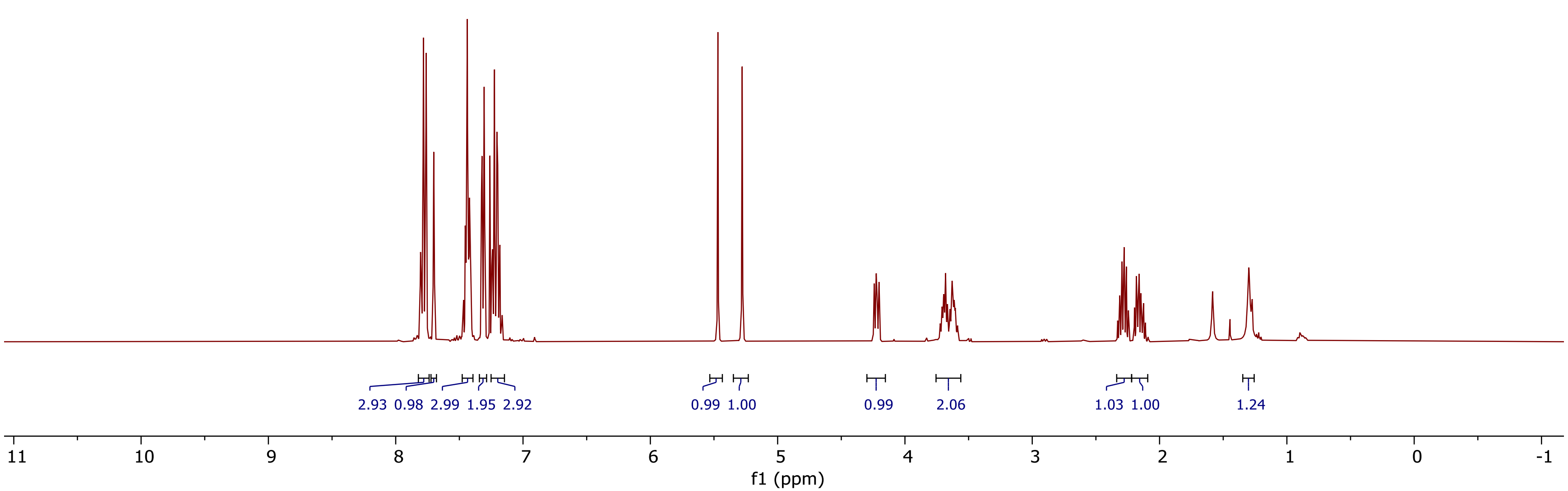

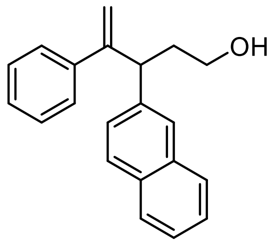

15n

^{1}H NMR (400 MHz, 298 K, $CDCl_3$)

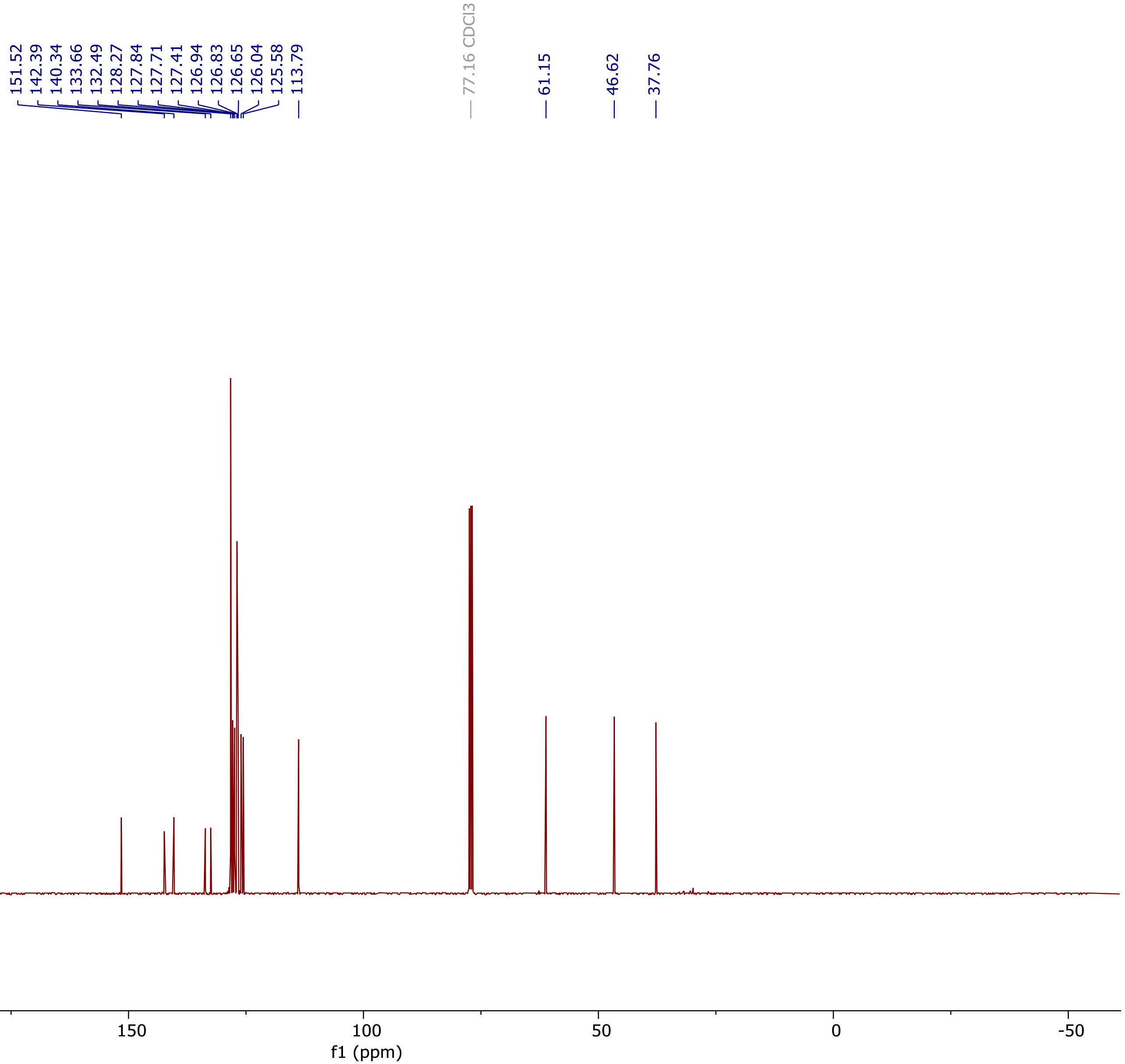

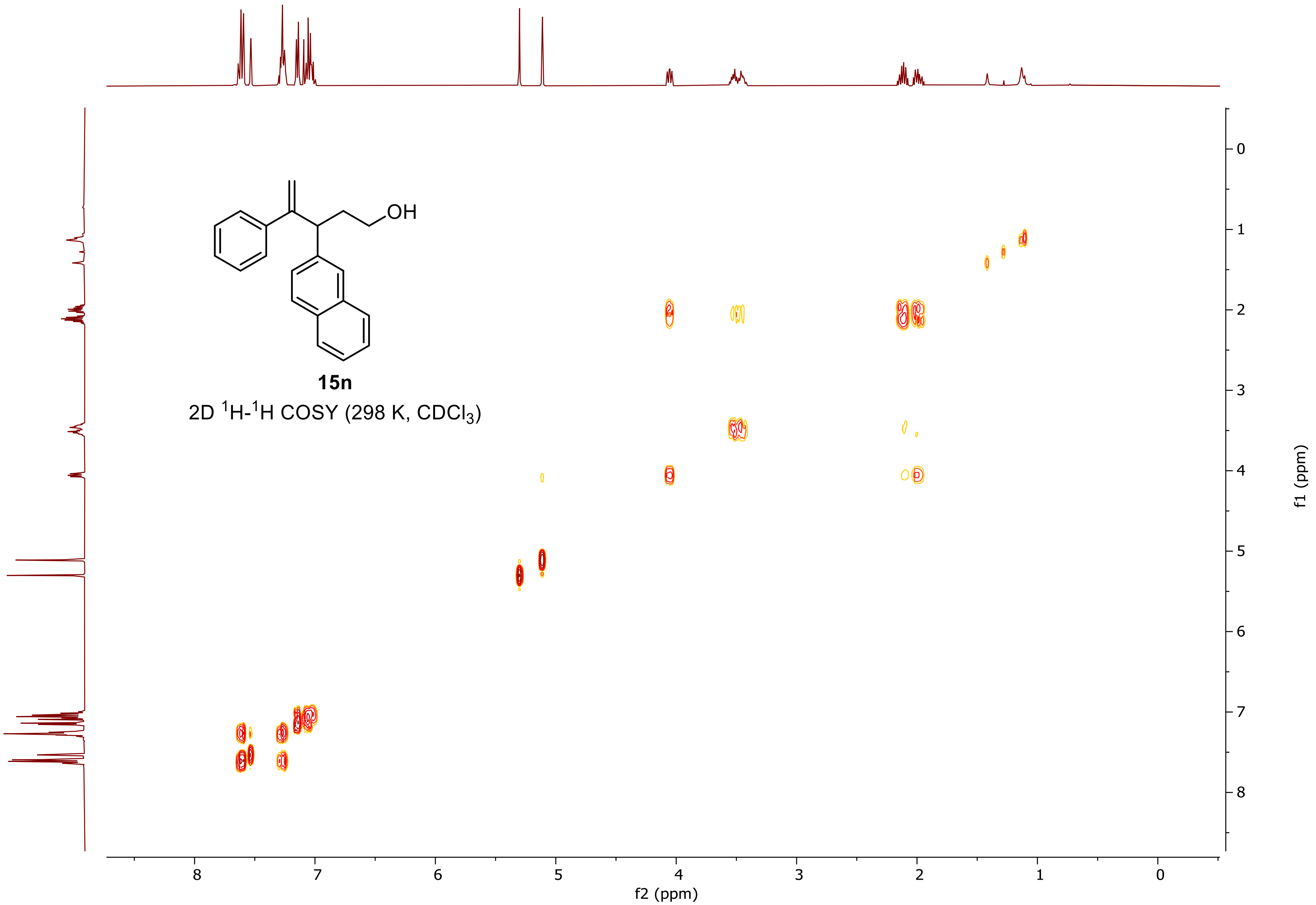

15n

2D ^{1}H-^{1}H COSY (298 K, $CDCl_3$)

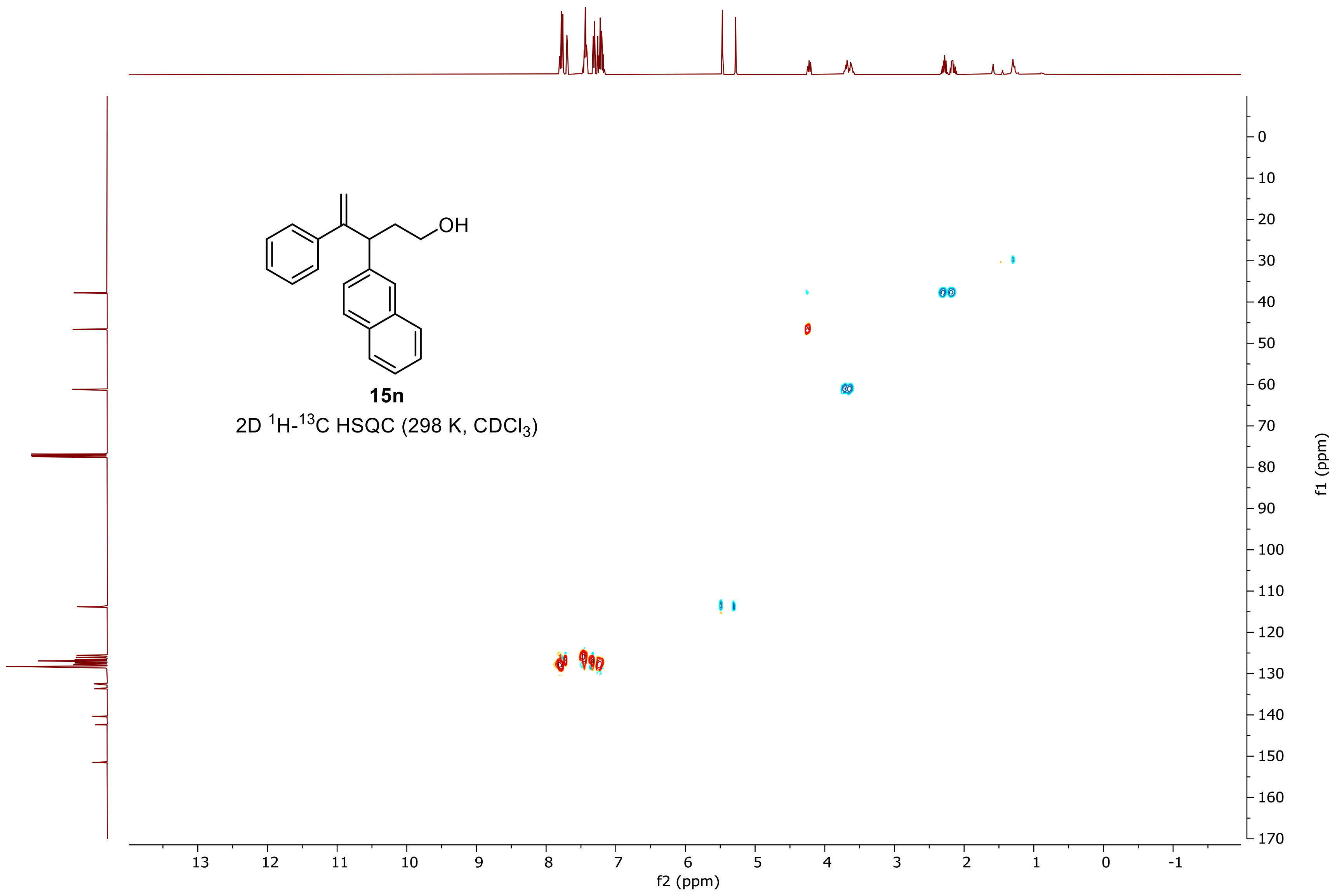

15n

2D ^{1}H-^{13}C HSQC (298 K, $CDCl_3$)

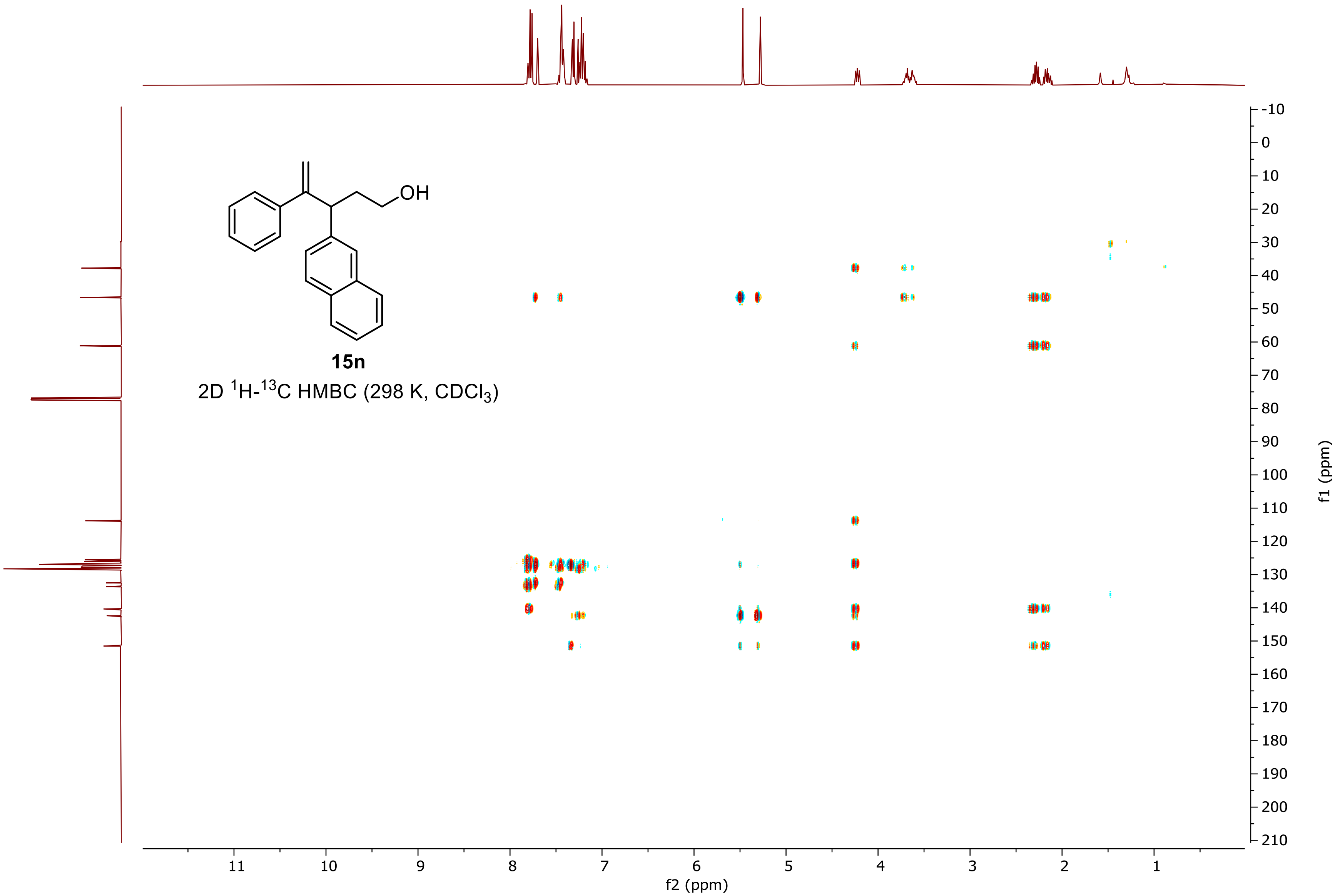
OH
15n
2D ^{1}H-^{13}C HMBC (298 K, CDCl$_3$)
f2 (ppm)
f1 (ppm)
11
10
9
8
7
6
5
4
3
2
1
-10
0
10
20
30
40
50
60
70
80
90
100
110
120
130
140
150
160
170
180
190
200
210

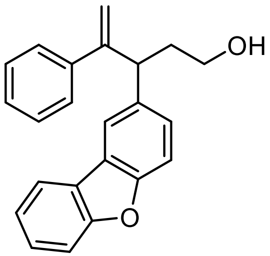

15o

^1H NMR (400 MHz, 298 K, $CDCl_3$)

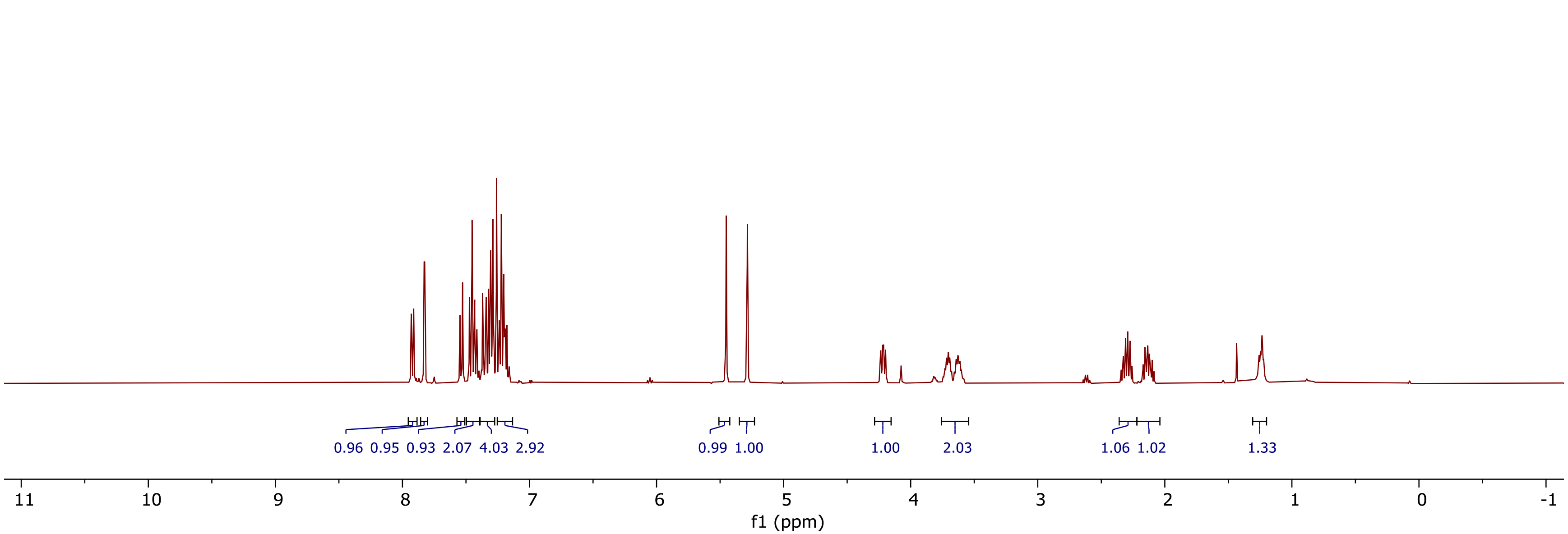

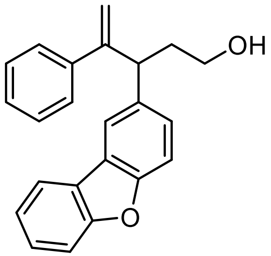

15o

$^{13}C\{^{1}H\}$ NMR (101 MHz, 298 K, $CDCl_3$)

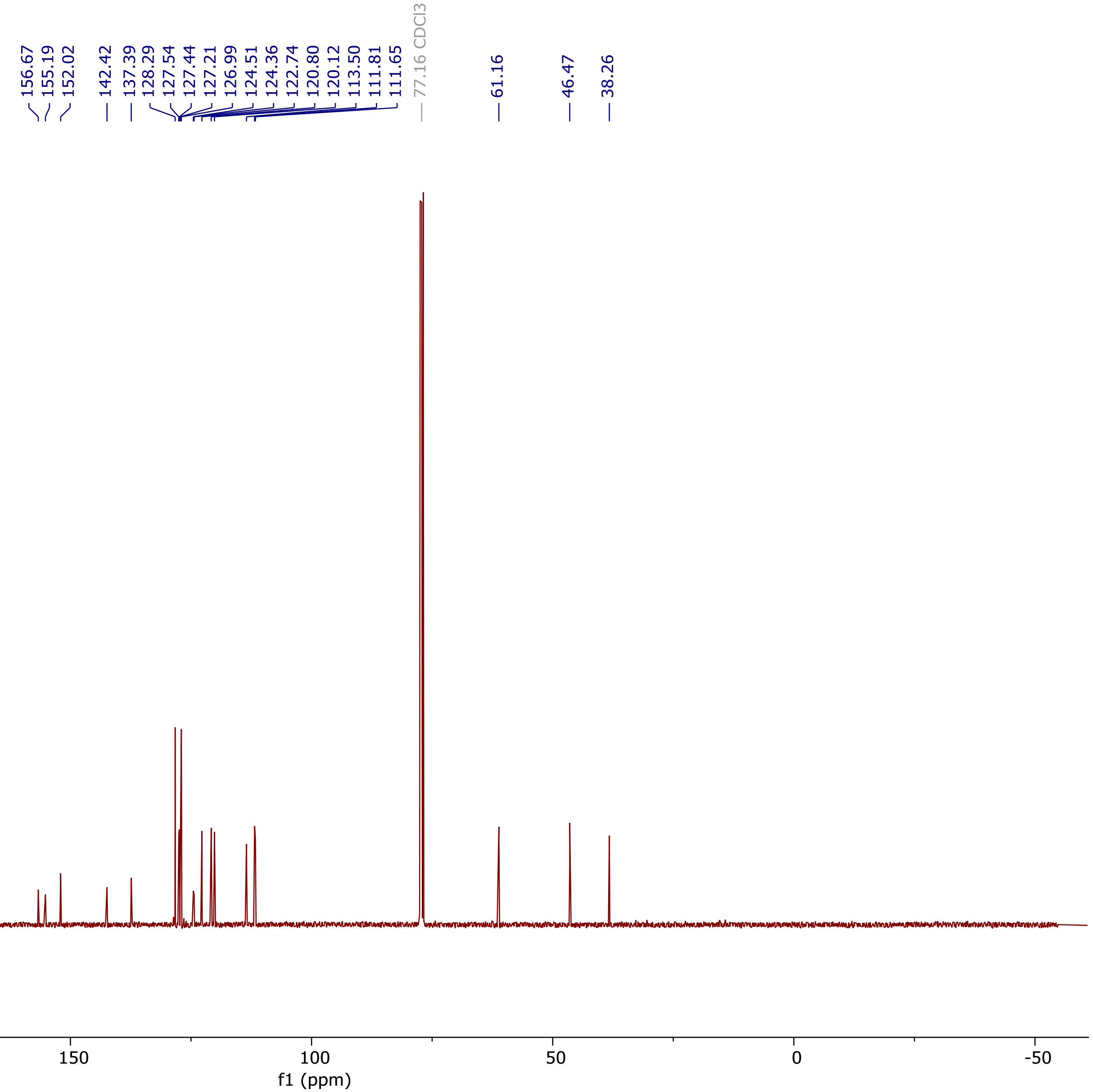

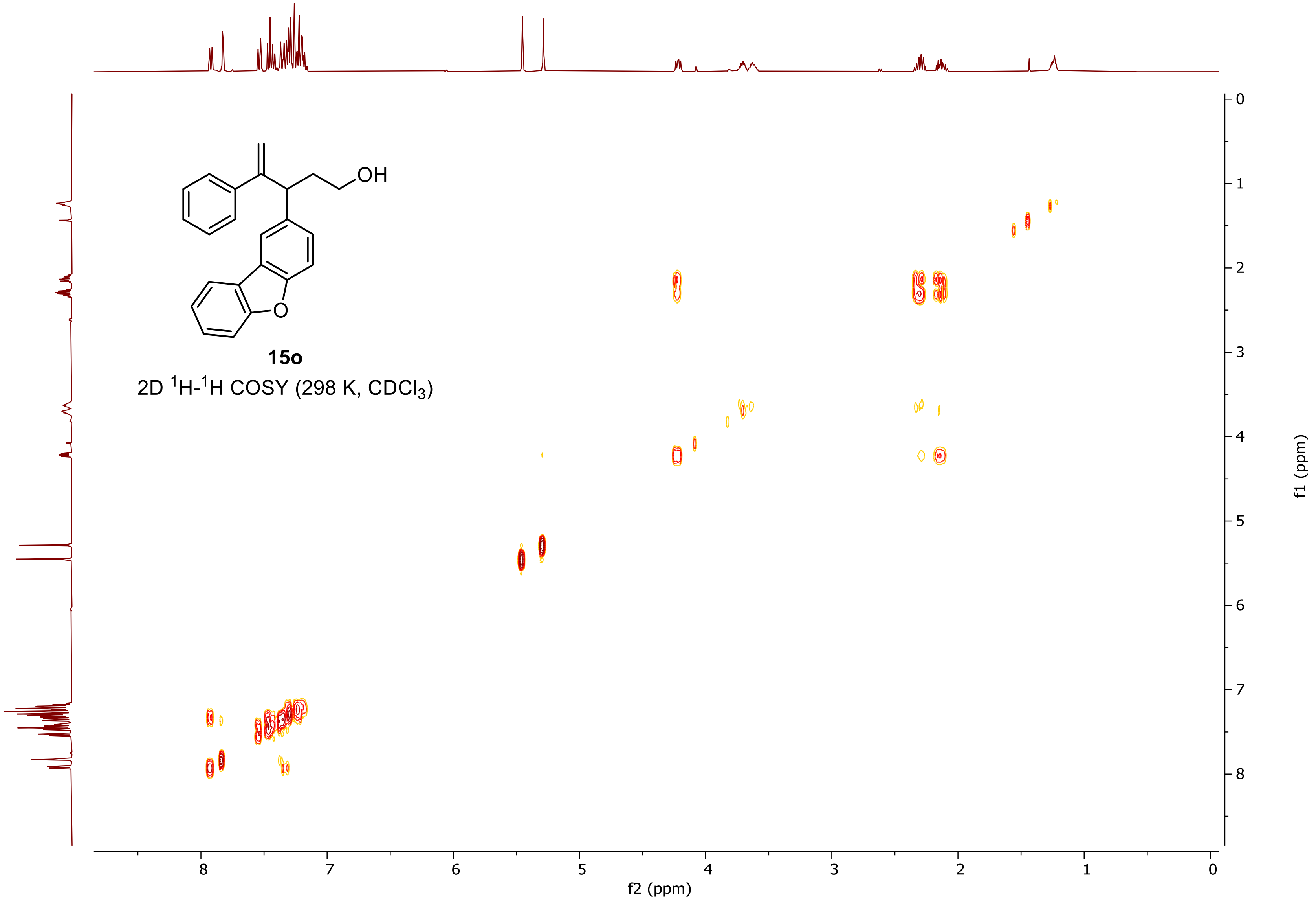
OH
O
15o
2D 1H-1H COSY (298 K, CDCl3)
f2 (ppm)
f1 (ppm)

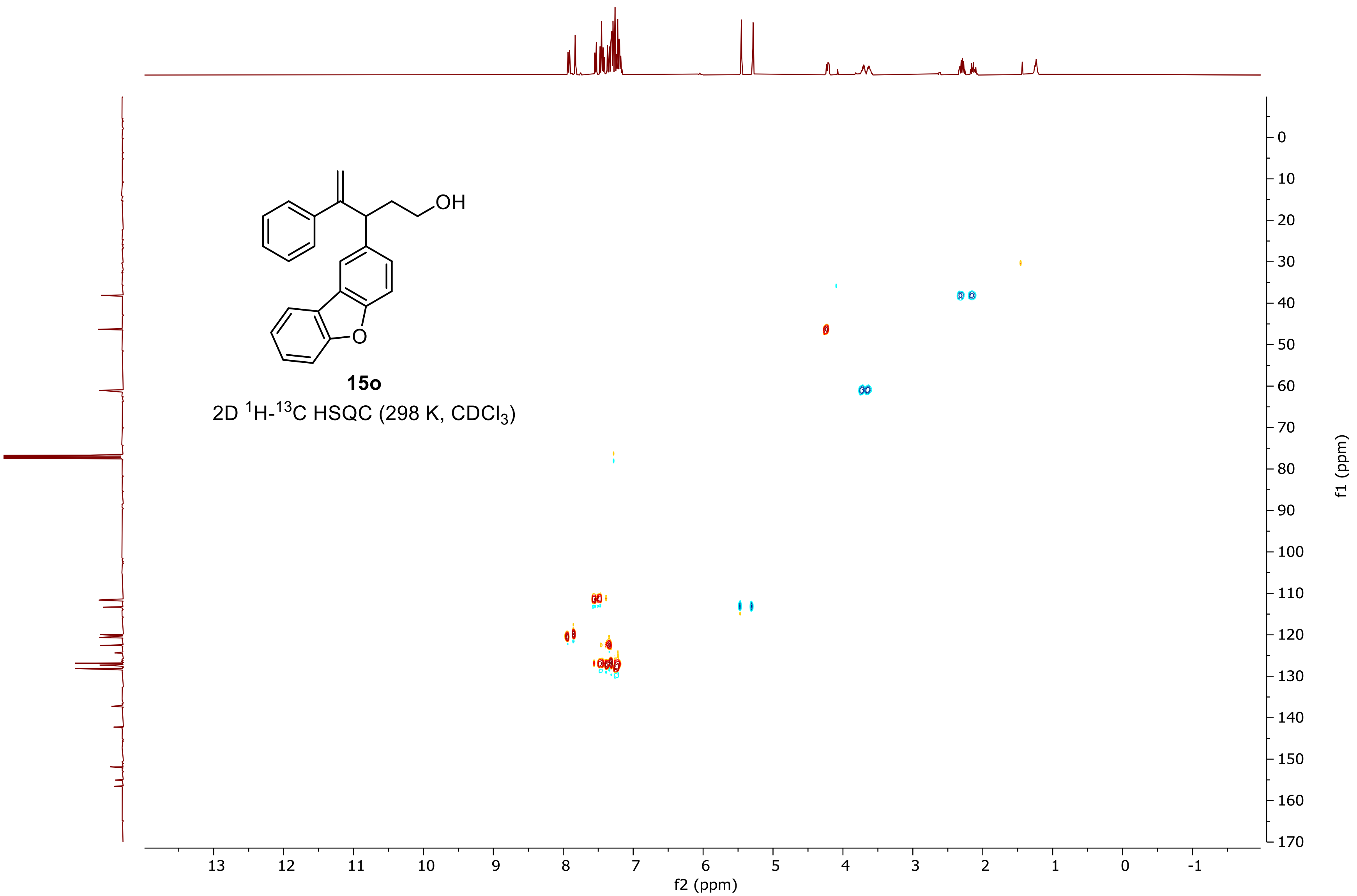
OH
O
15o
2D ¹H-¹³C HSQC (298 K, CDCl₃)
f2 (ppm)
f1 (ppm)

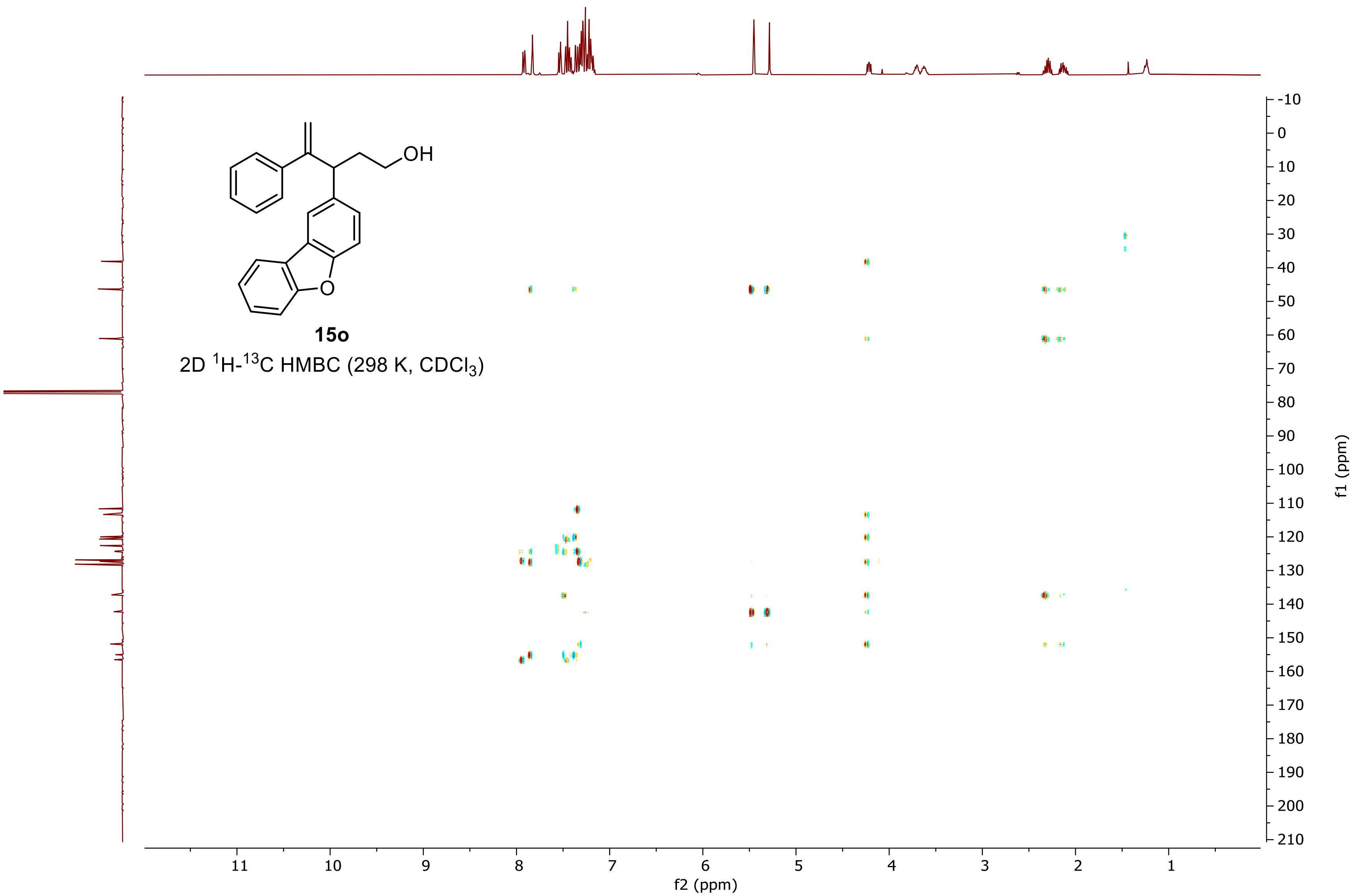
OH
O
15o
2D ¹H-¹³C HMBC (298 K, CDCl₃)
f2 (ppm)
f1 (ppm)

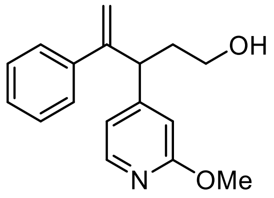

15p

^{1}H NMR (400 MHz, 298 K, $CDCl_3$)

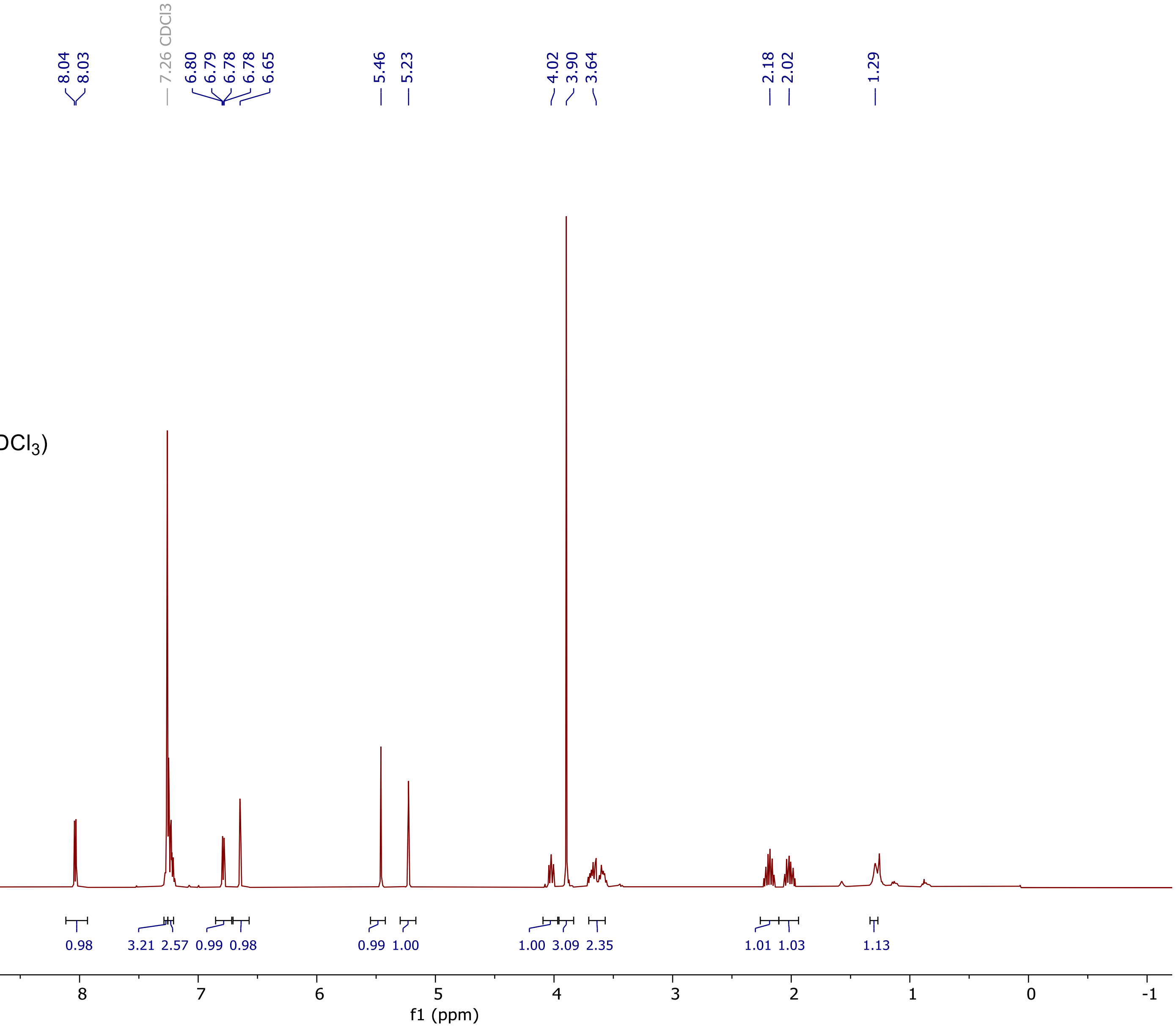

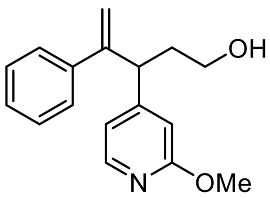

15p

$^{13}C\{^{1}H\}$ NMR (101 MHz, 298 K, $CDCl_3$)

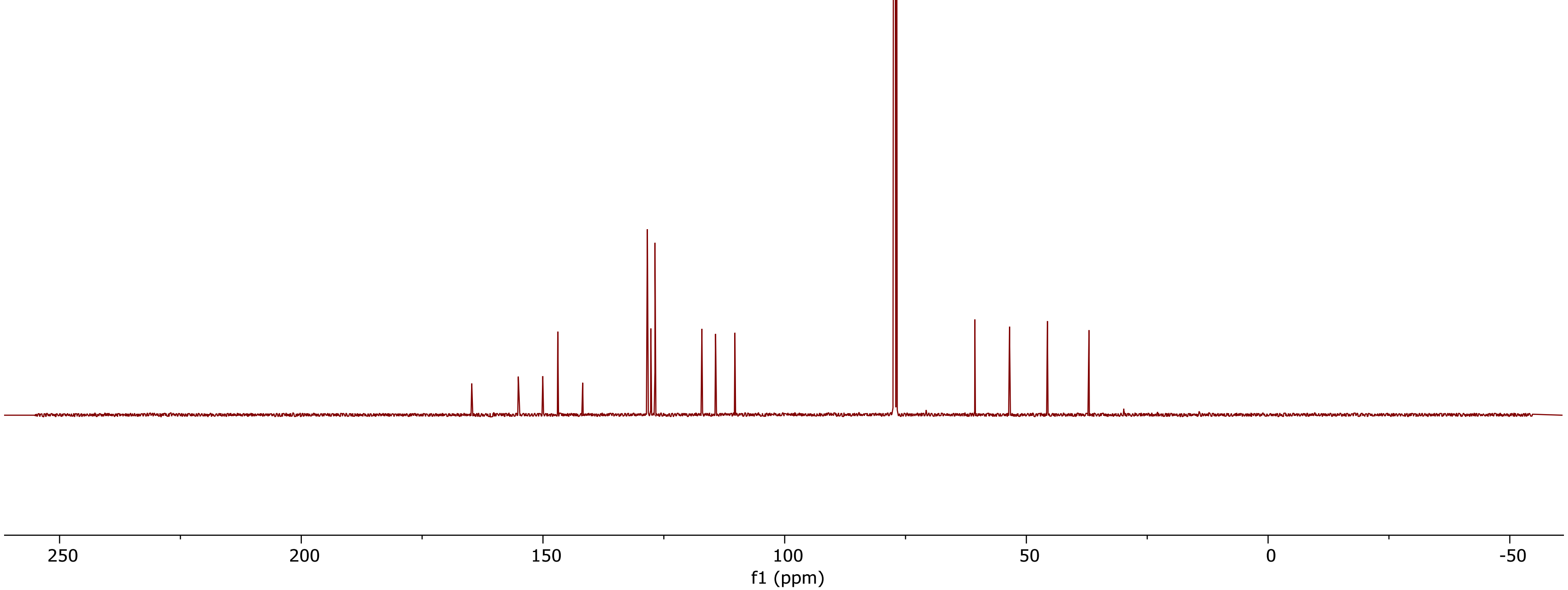

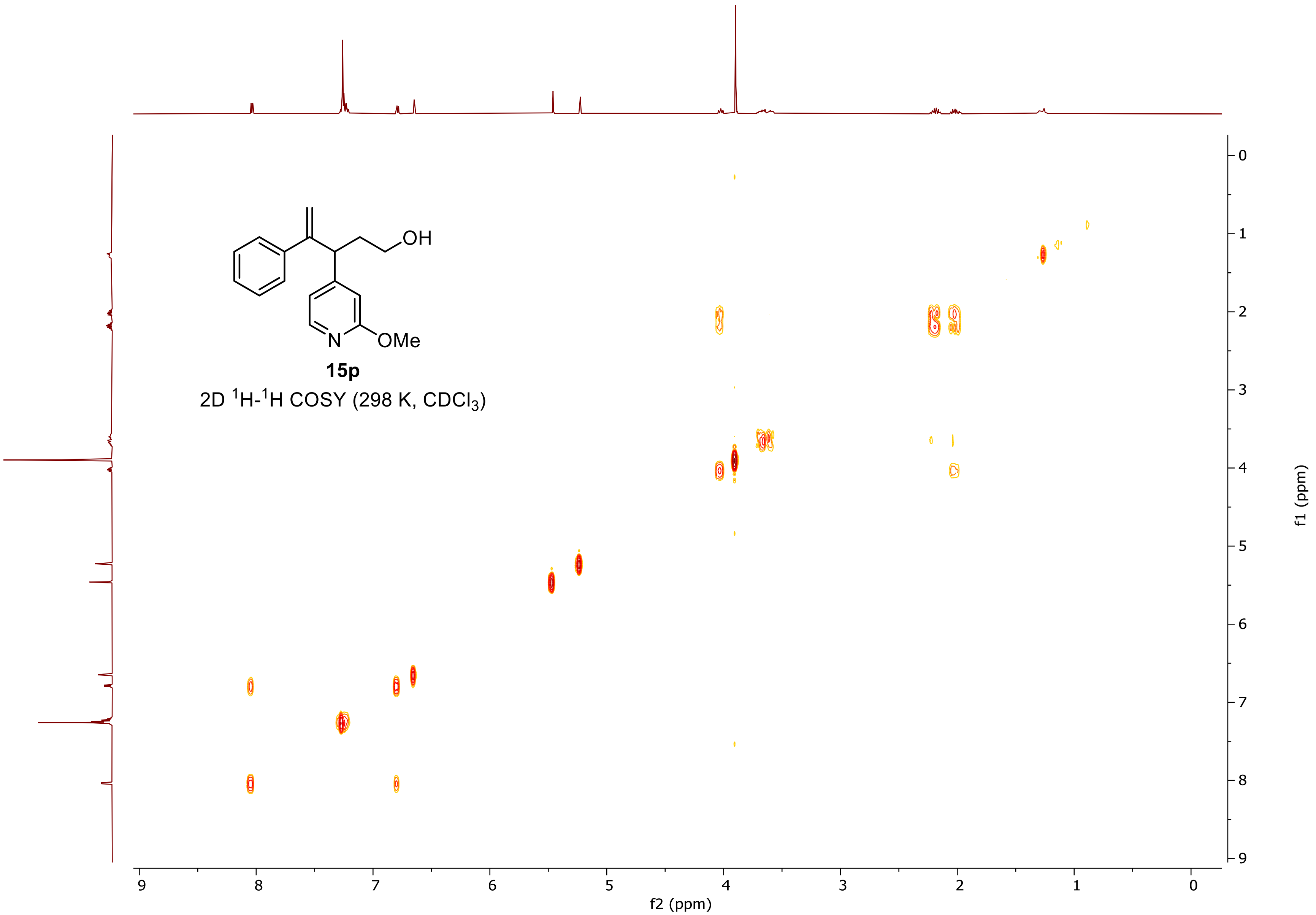
OH
N
OMe
15p
2D 1H-1H COSY (298 K, CDCl3)
f2 (ppm)
f1 (ppm)

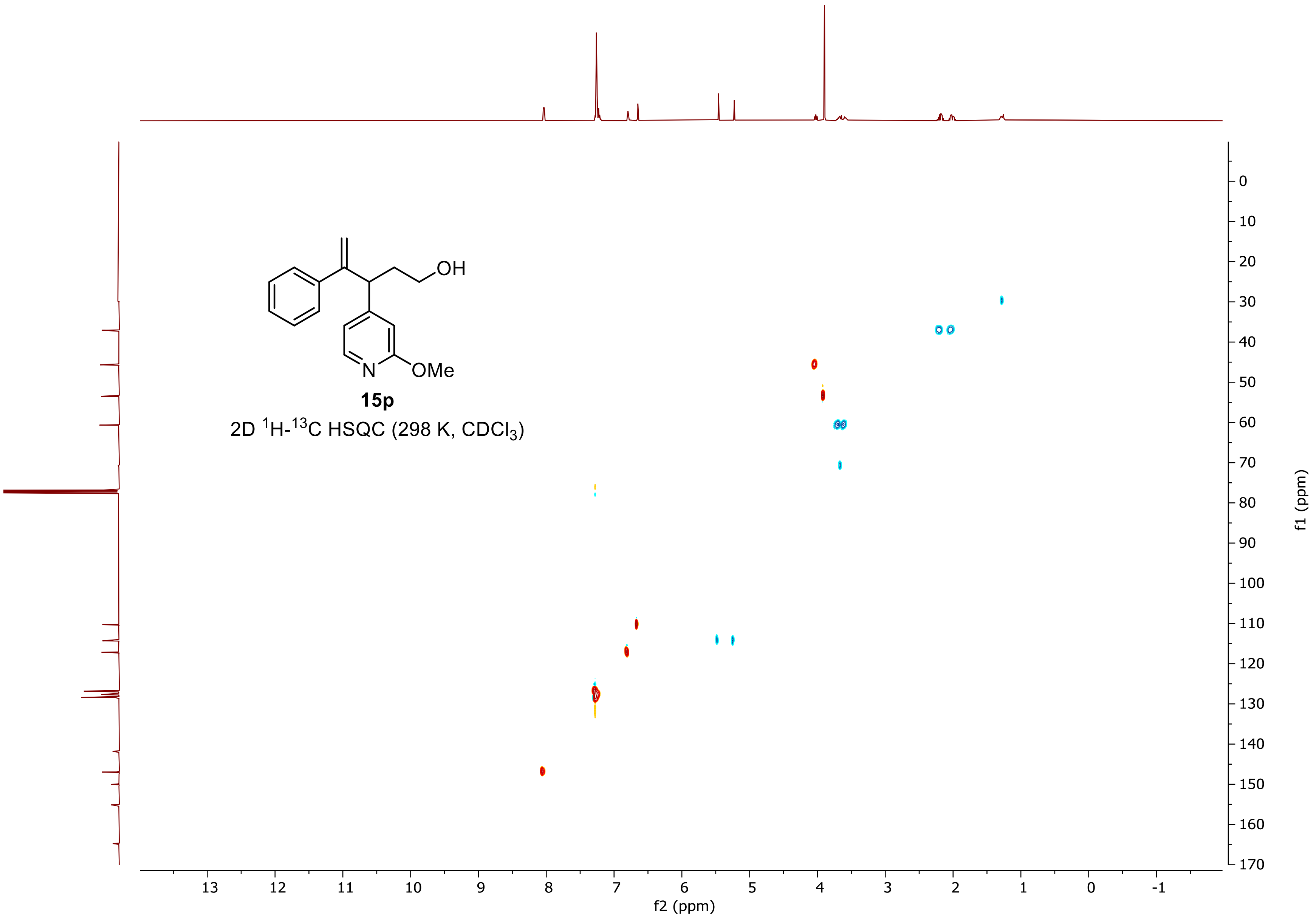

15p

2D ^{1}H-^{13}C HSQC (298 K, $CDCl_3$)

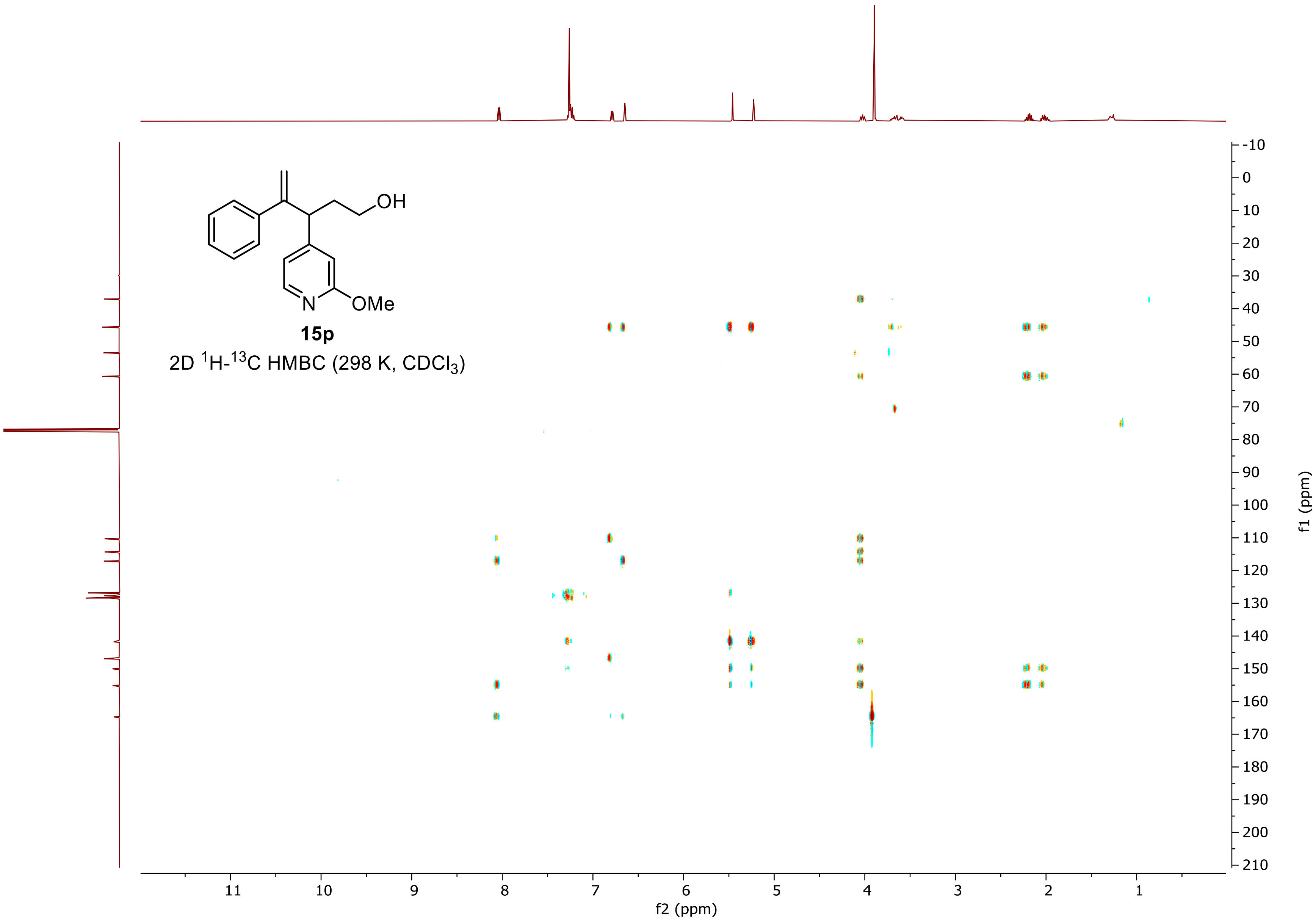
OH
N
OMe
15p
2D ¹H-¹³C HMBC (298 K, CDCl₃)
f2 (ppm)
f1 (ppm)

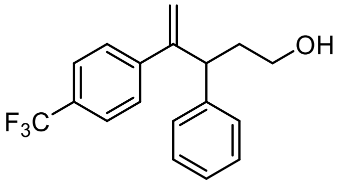

15q

^{1}H NMR (400 MHz, 298 K, $CDCl_3$)

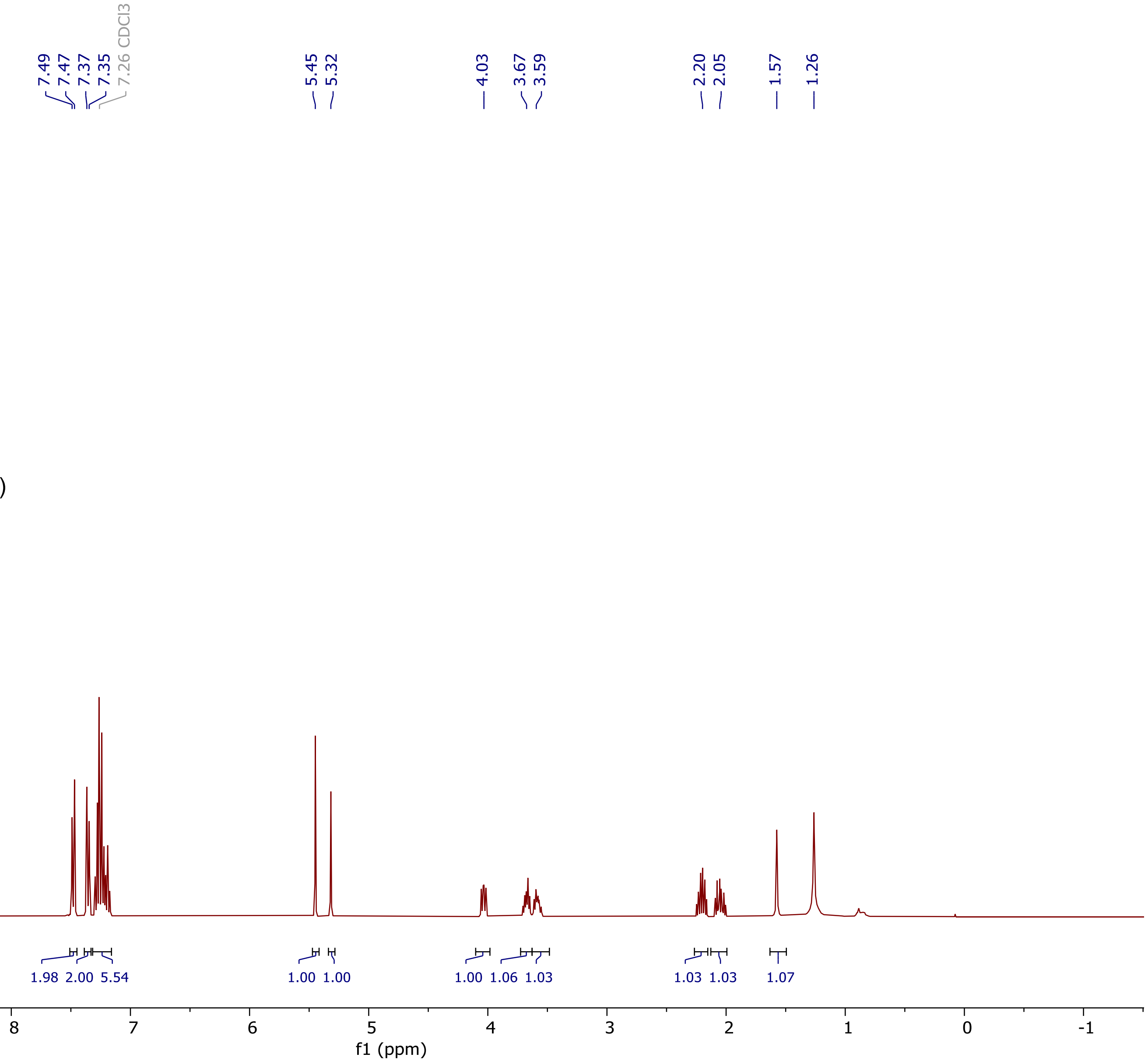

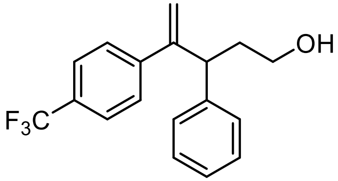

15q

$^{13}C\{^1H\}$ NMR (101 MHz, 298 K, $CDCl_3$)

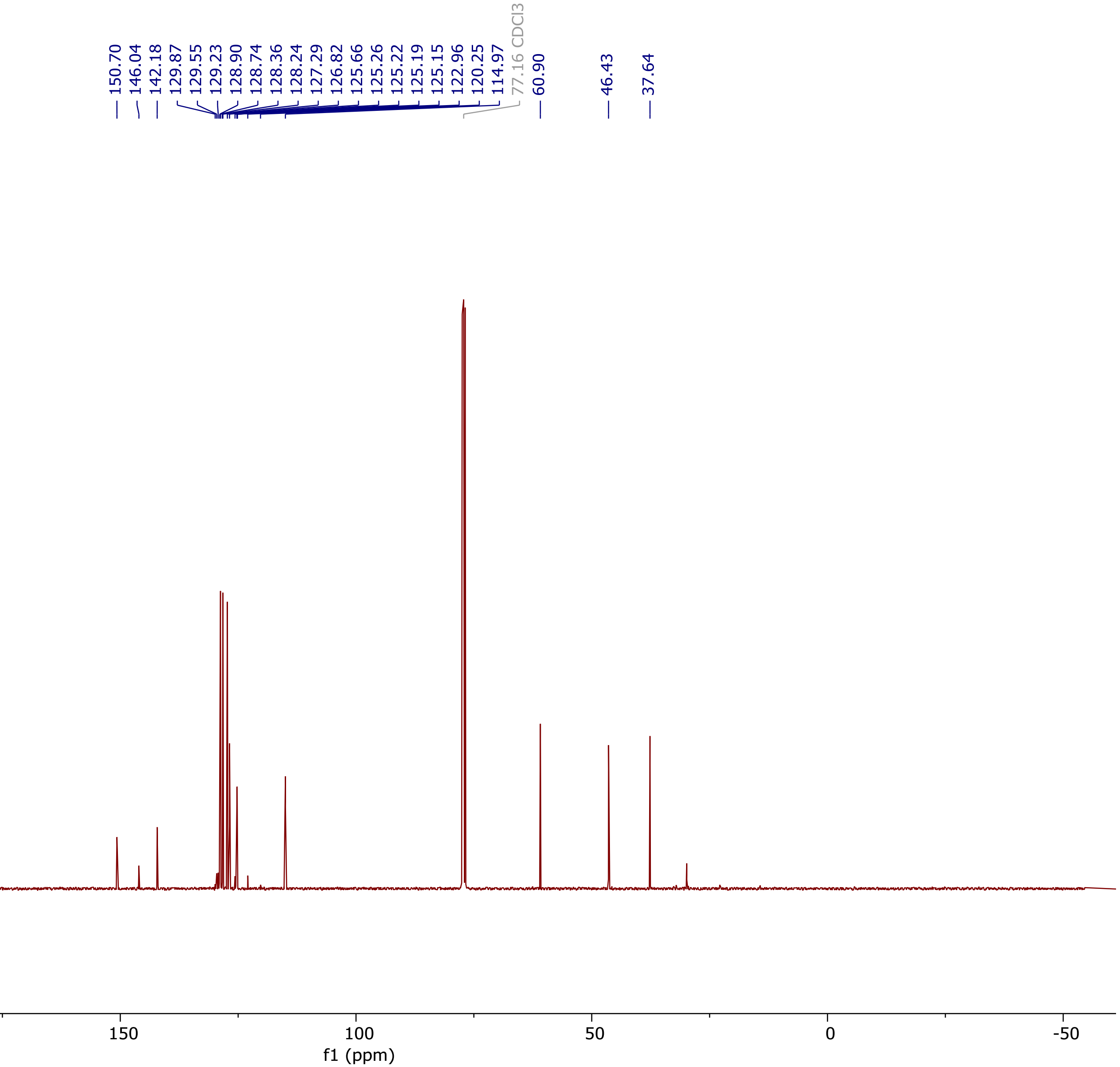

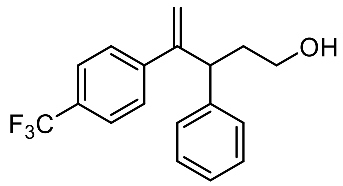

15q

$^{19}F\{^1H\}$ NMR (282 MHz, 298 K, $CDCl_3$)

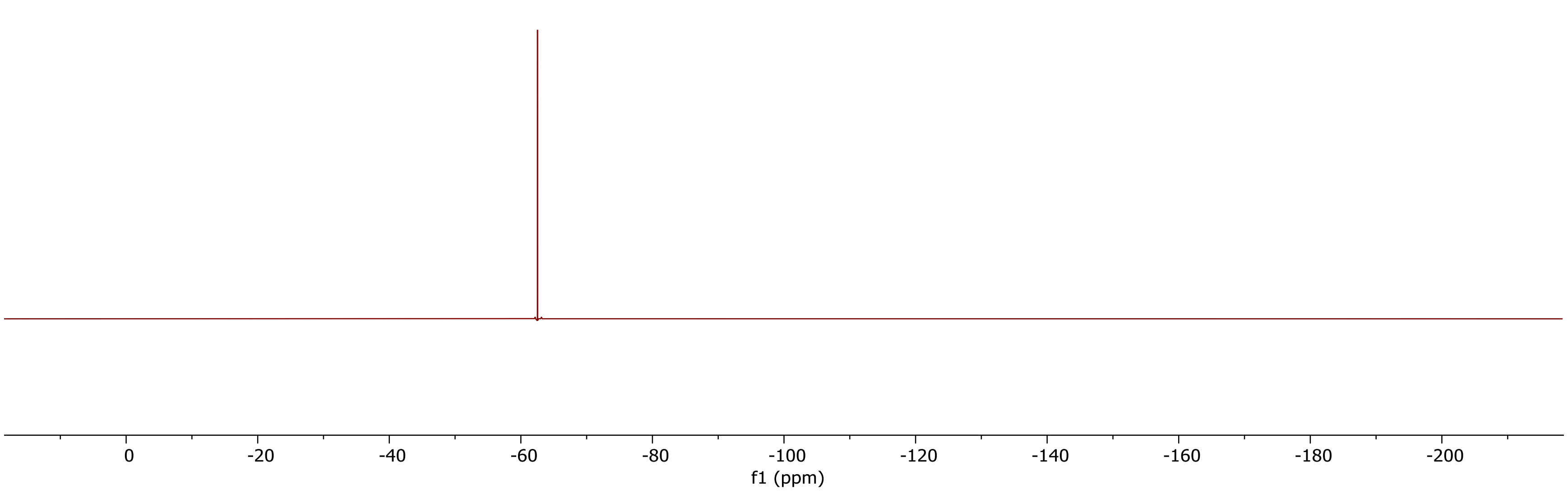

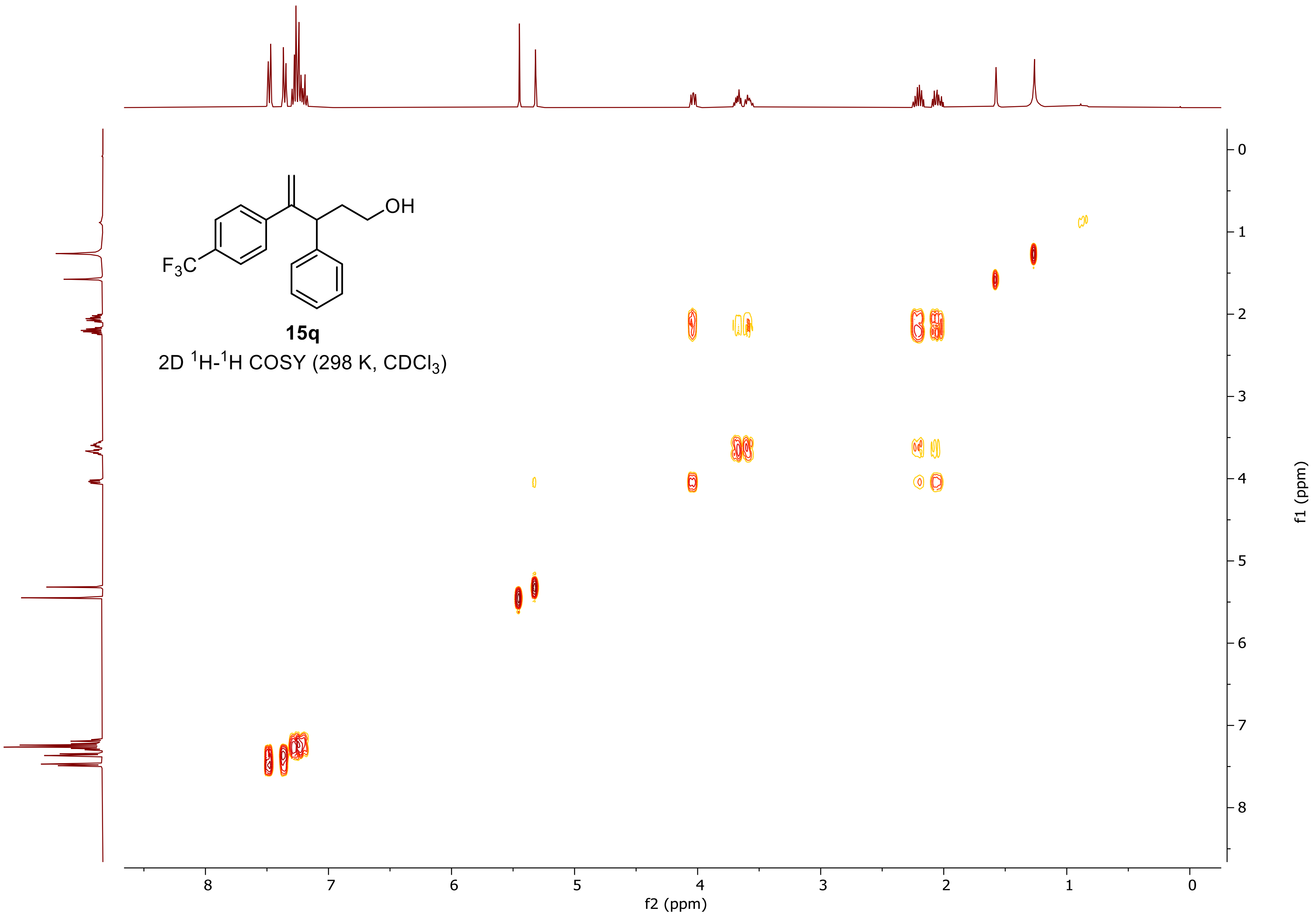

15q

2D ^1H-^1H COSY (298 K, $CDCl_3$)

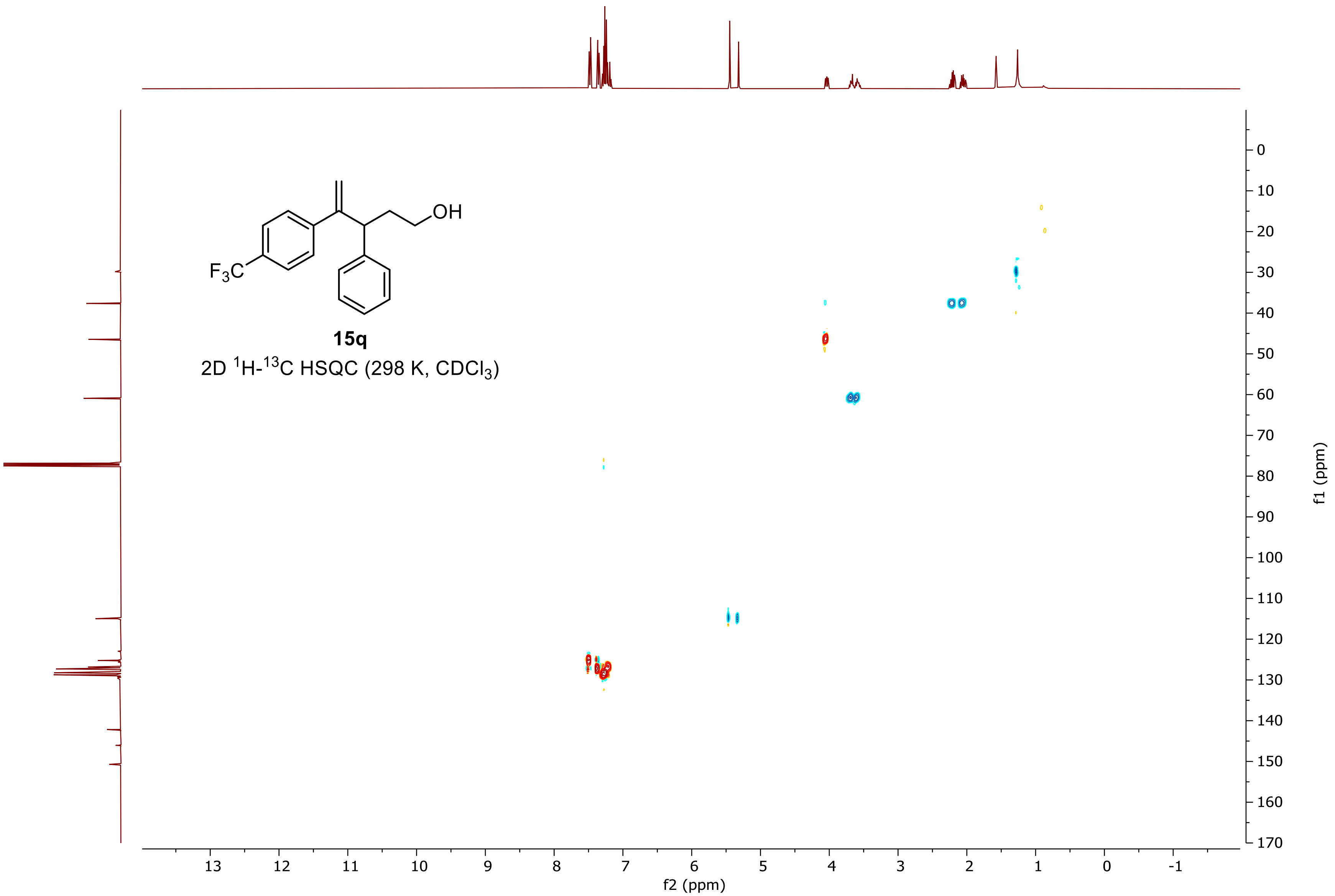
F3C
OH
15q
2D 1H-13C HSQC (298 K, CDCl3)
f2 (ppm)
f1 (ppm)

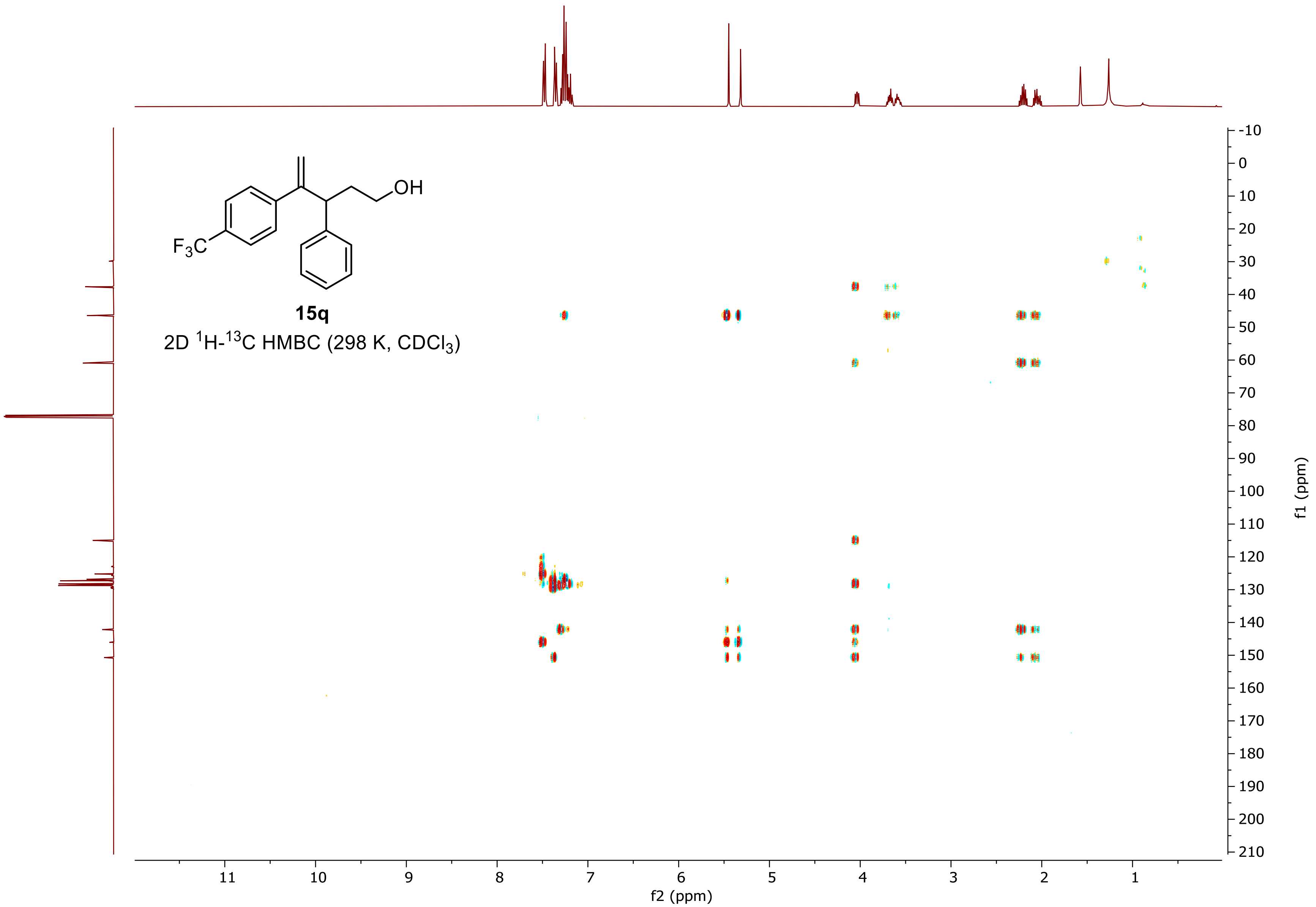
F3C
OH
15q
2D 1H-13C HMBC (298 K, CDCl3)
f2 (ppm)
f1 (ppm)

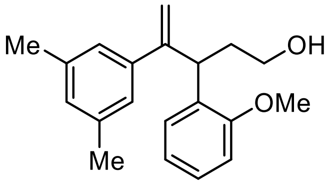

15s

^{1}H NMR (400 MHz, 298 K, $CDCl_3$)

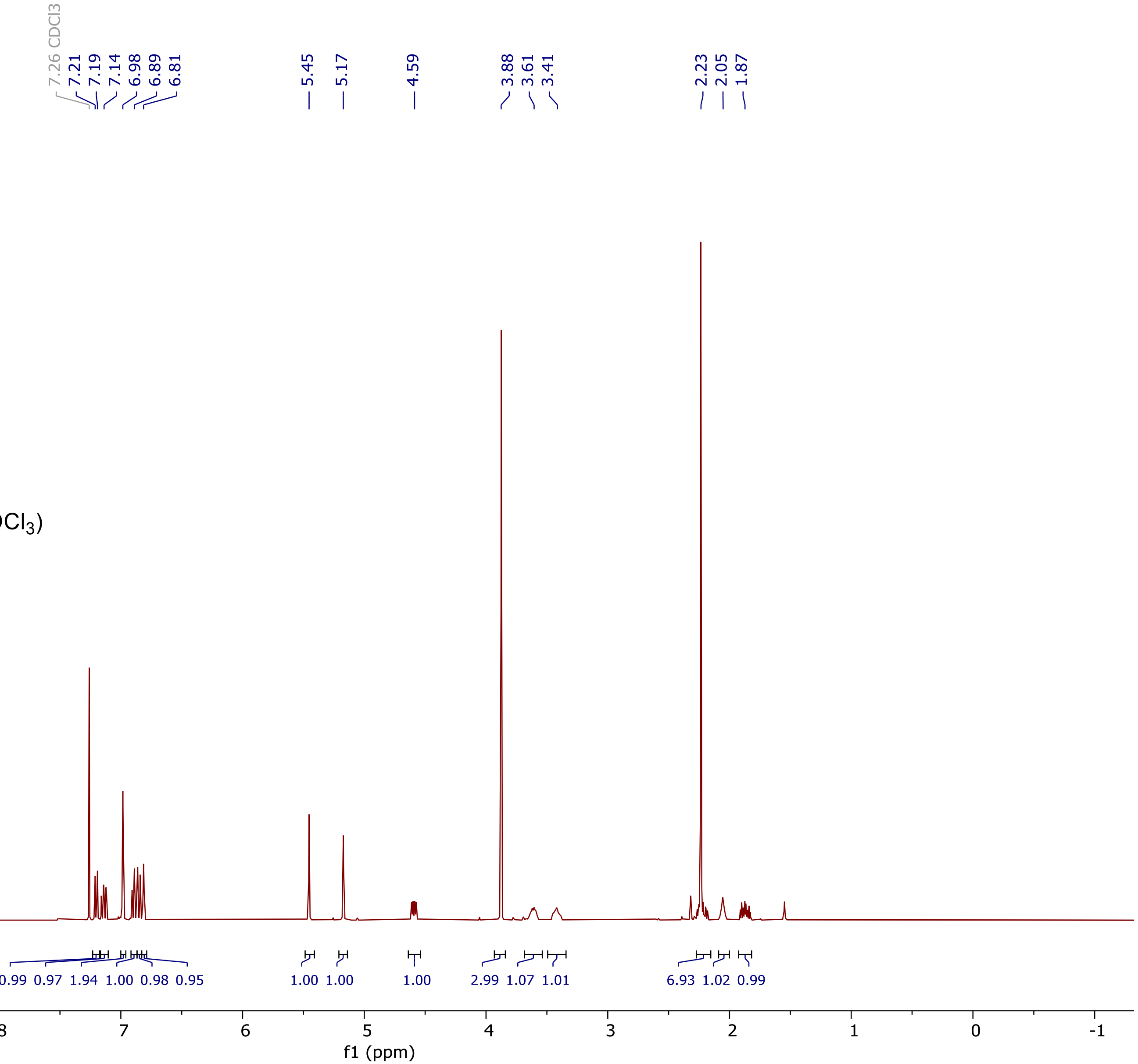

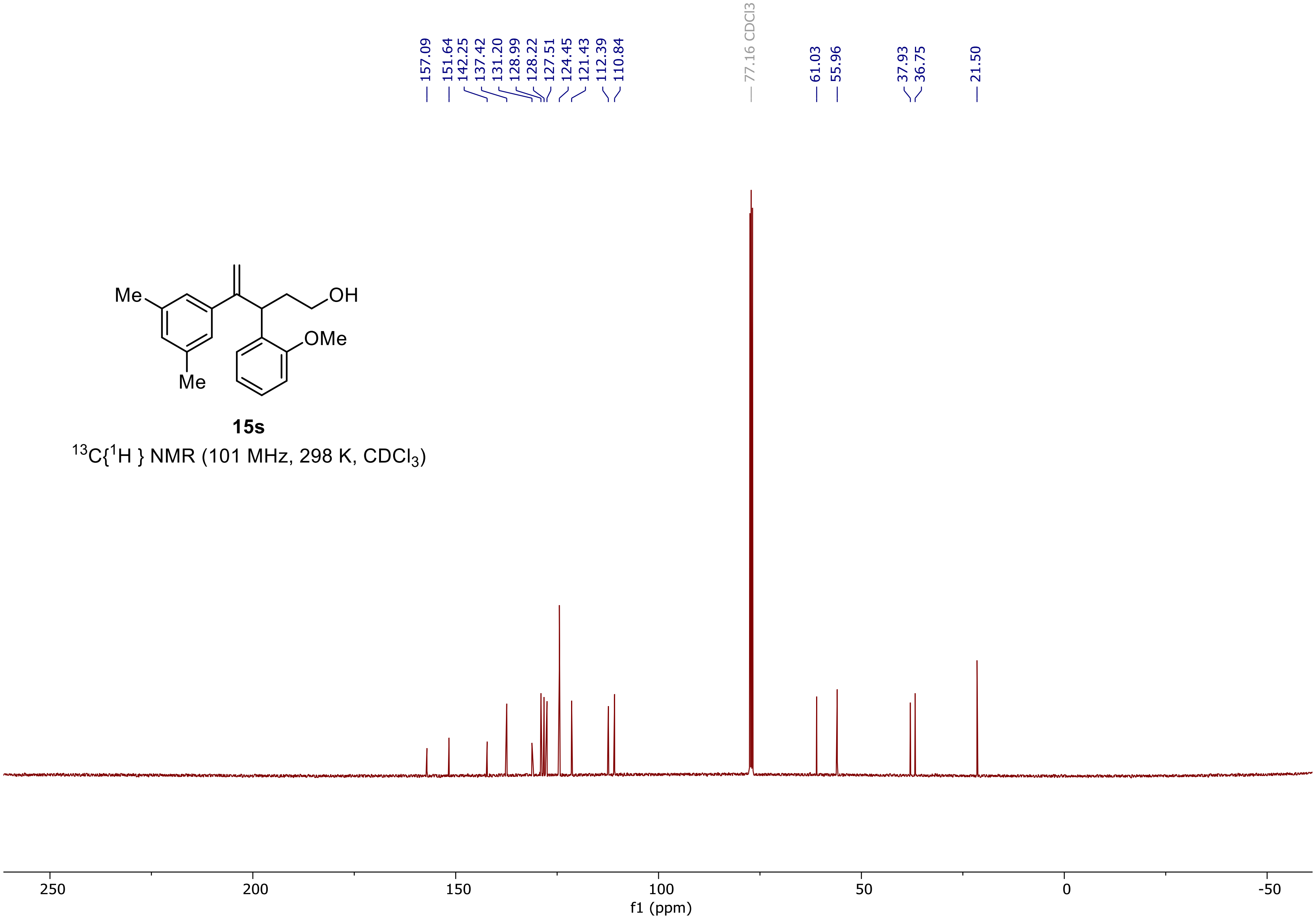

15s

$^{13}C\{^{1}H\}$ NMR (101 MHz, 298 K, $CDCl_3$)

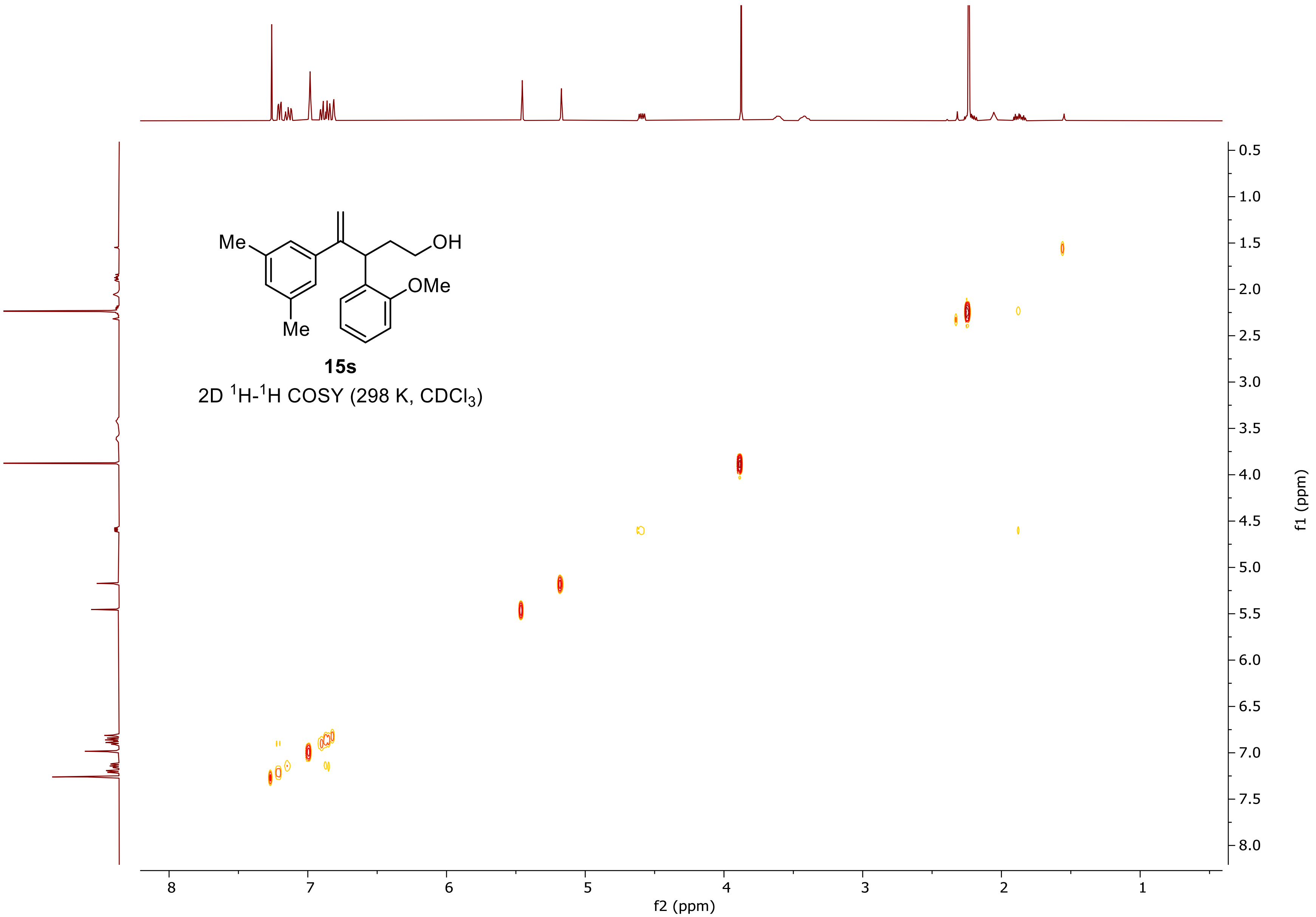
Me
OH
OMe
Me
15s
2D 1H-1H COSY (298 K, CDCl3)
f1 (ppm)
f2 (ppm)

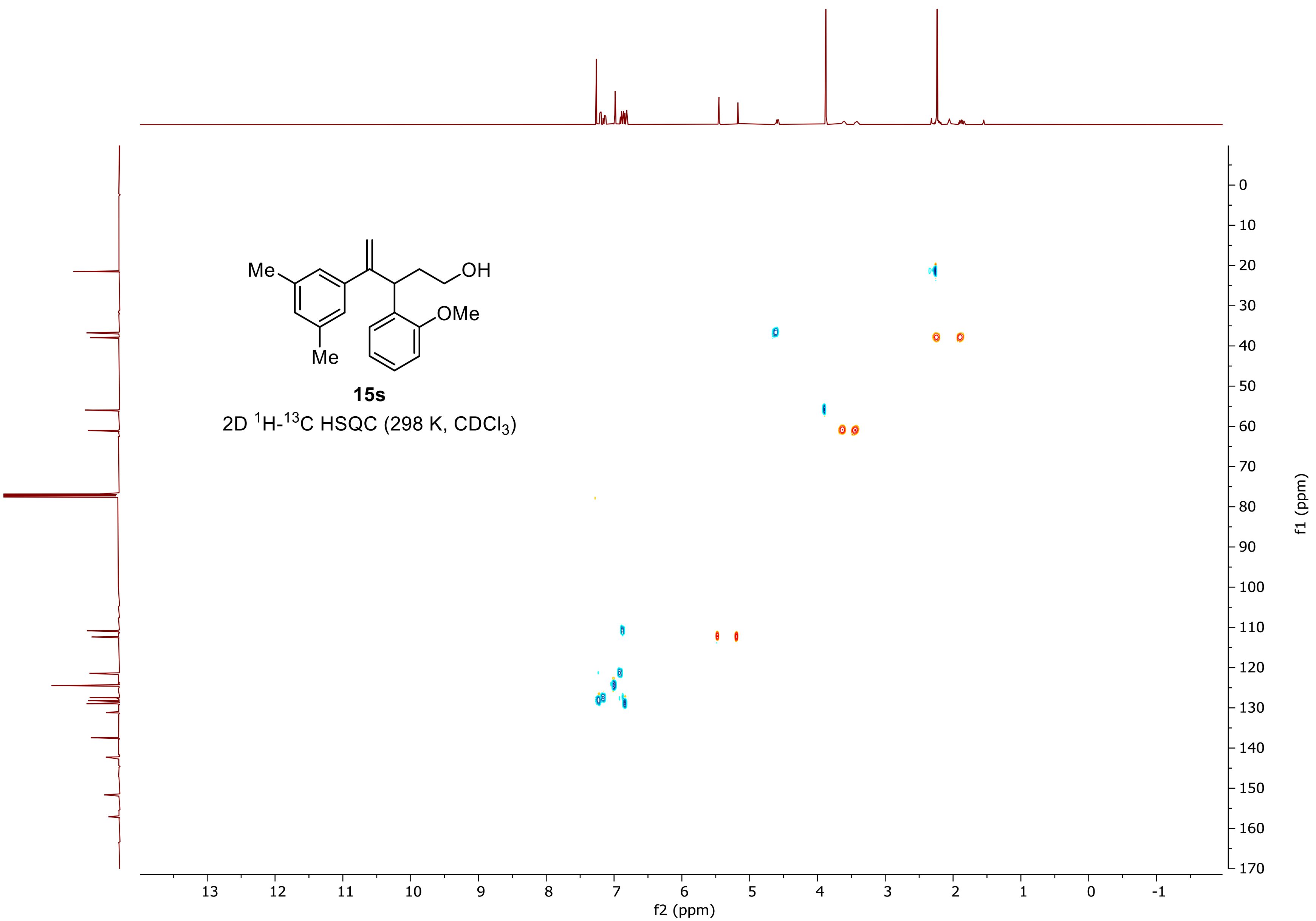
Me
OH
OMe
Me
15s
2D ^{1}H-^{13}C HSQC (298 K, CDCl$_3$)
f1 (ppm)
f2 (ppm)
13
12
11
10
9
8
7
6
5
4
3
2
1
0
-1
0
10
20
30
40
50
60
70
80
90
100
110
120
130
140
150
160
170

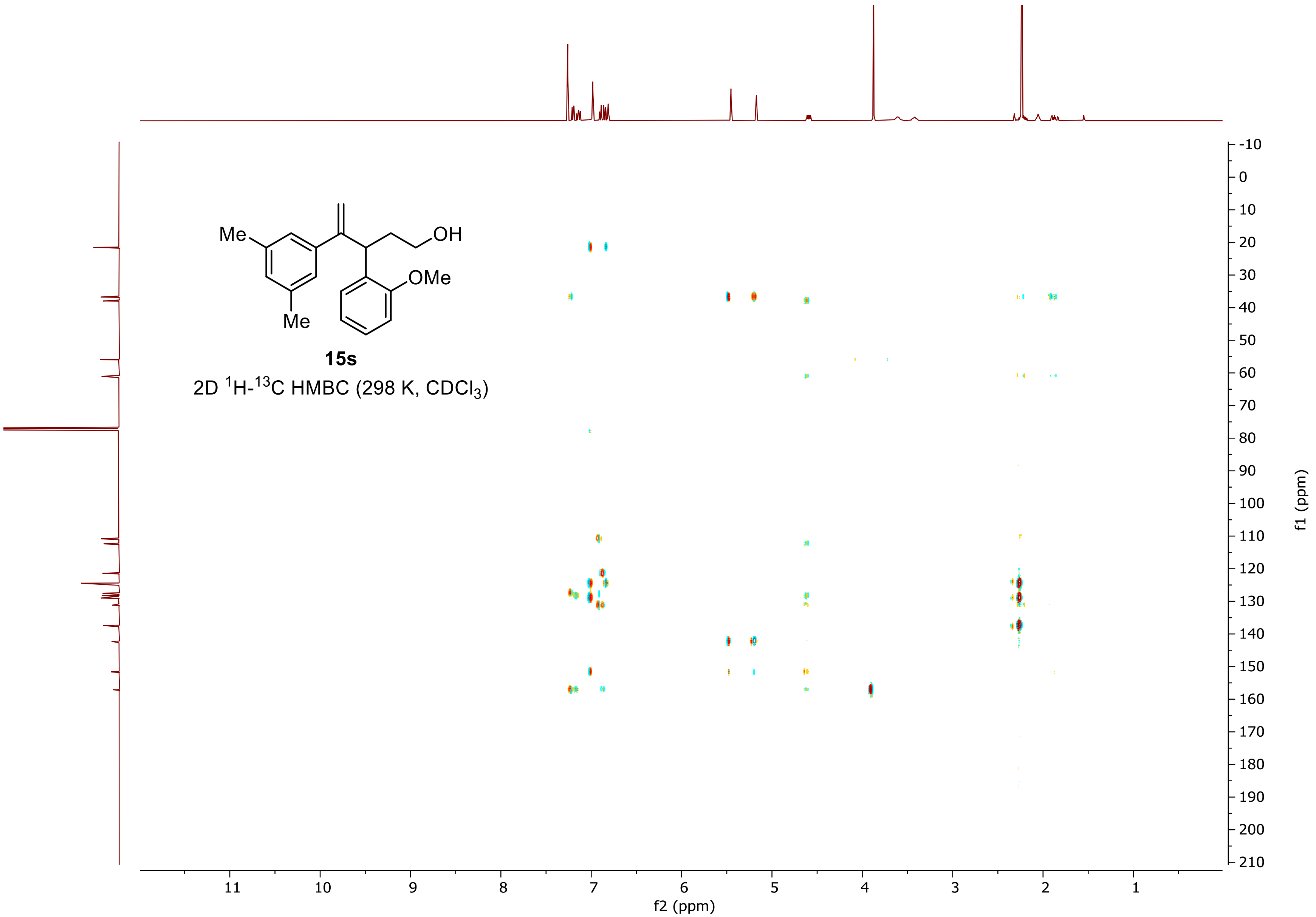
Me
OH
OMe
Me
15s
2D ^{1}H-^{13}C HMBC (298 K, $CDCl_3$)
f2 (ppm)
f1 (ppm)

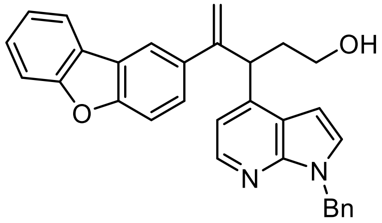

15t

^{1}H NMR (400 MHz, 298 K, $CDCl_3$)

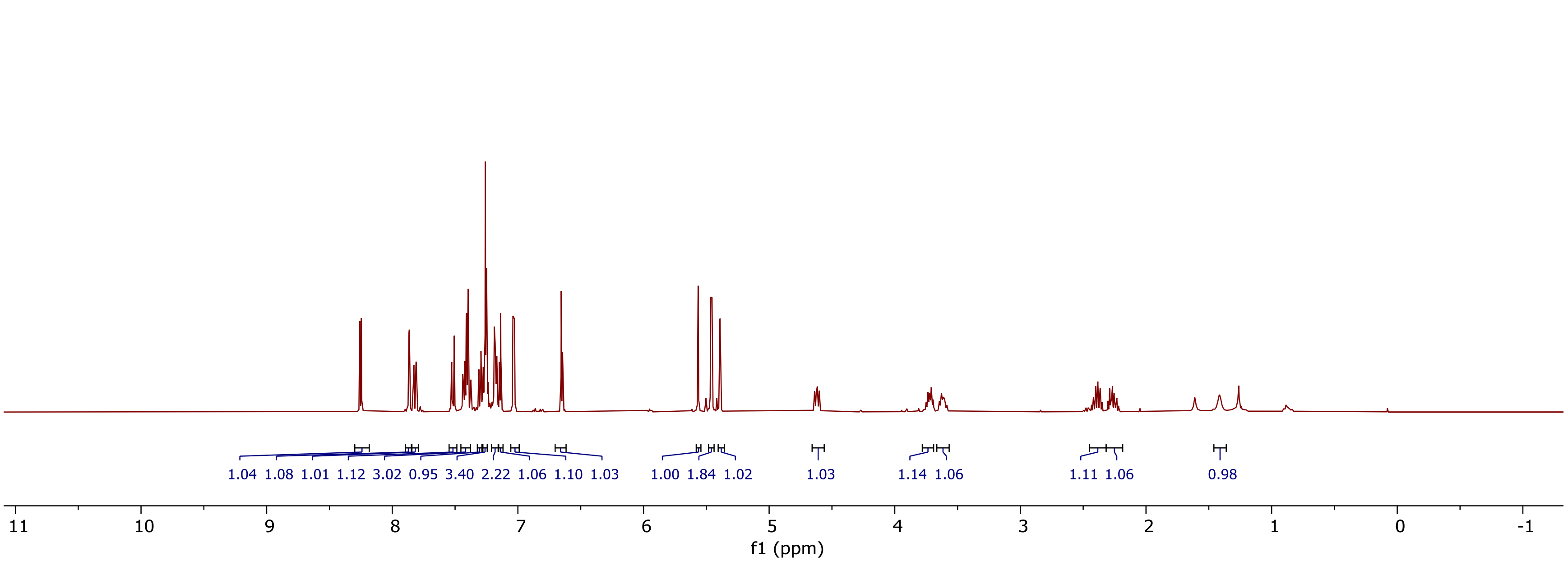

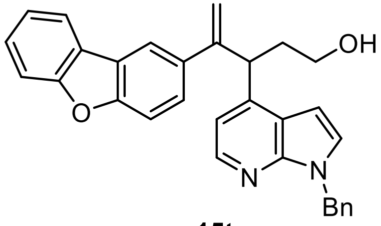

15t

$^{13}C\{^{1}H\}$ NMR (101 MHz, 298 K, $CDCl_3$)

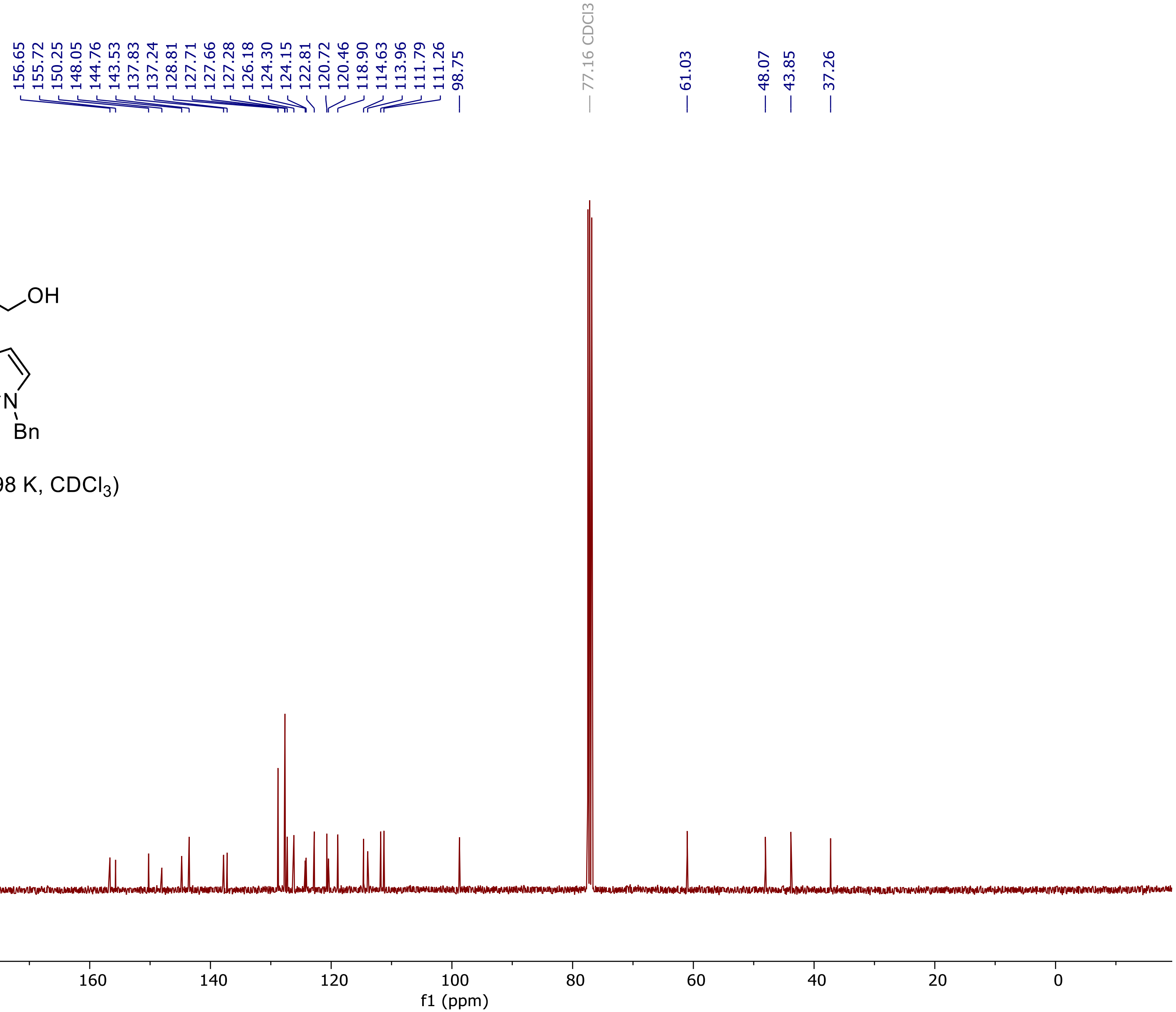

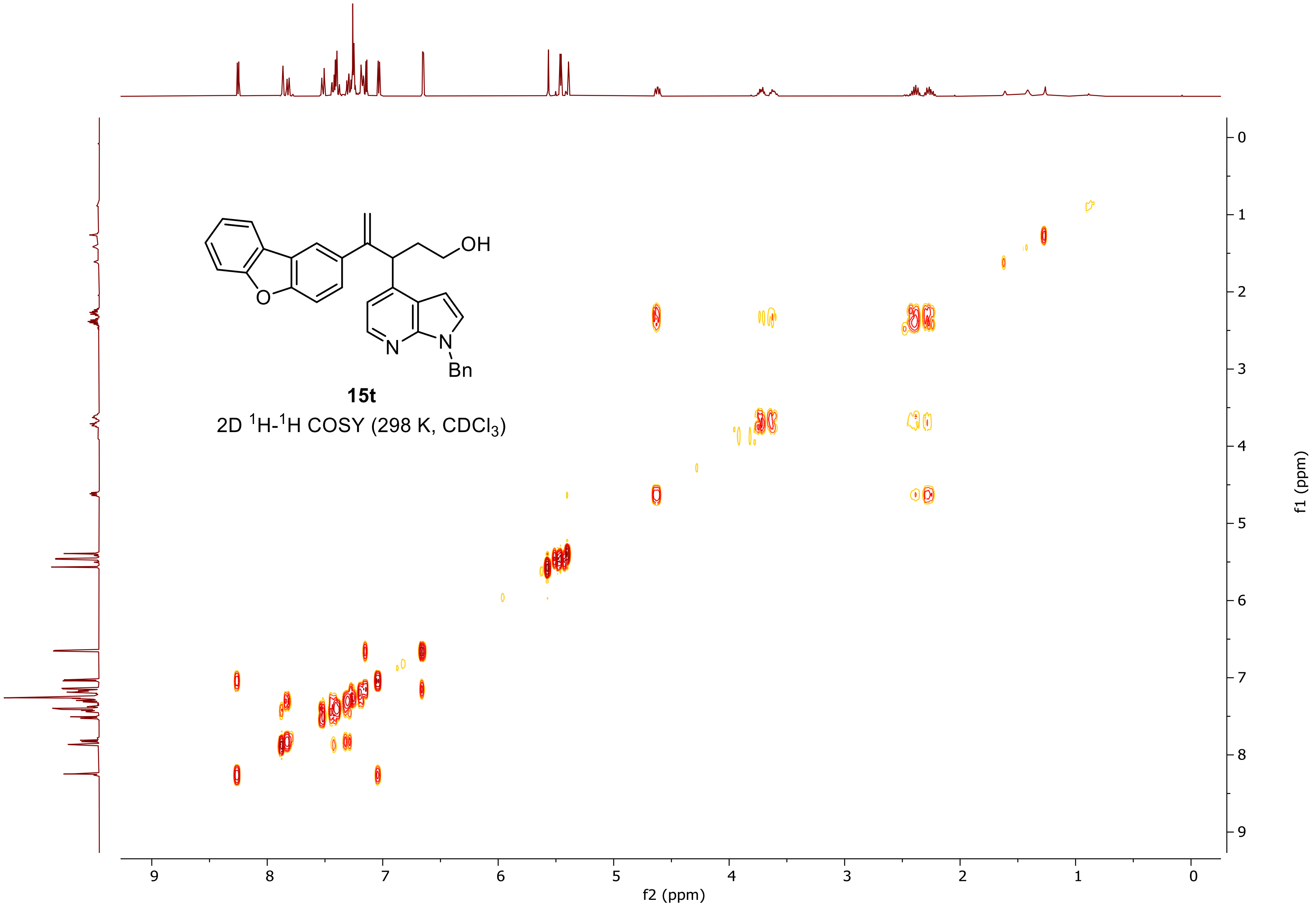
OH
O
N
N
Bn
15t
2D 1H-1H COSY (298 K, CDCl3)
f2 (ppm)
f1 (ppm)
9
8
7
6
5
4
3
2
1
0

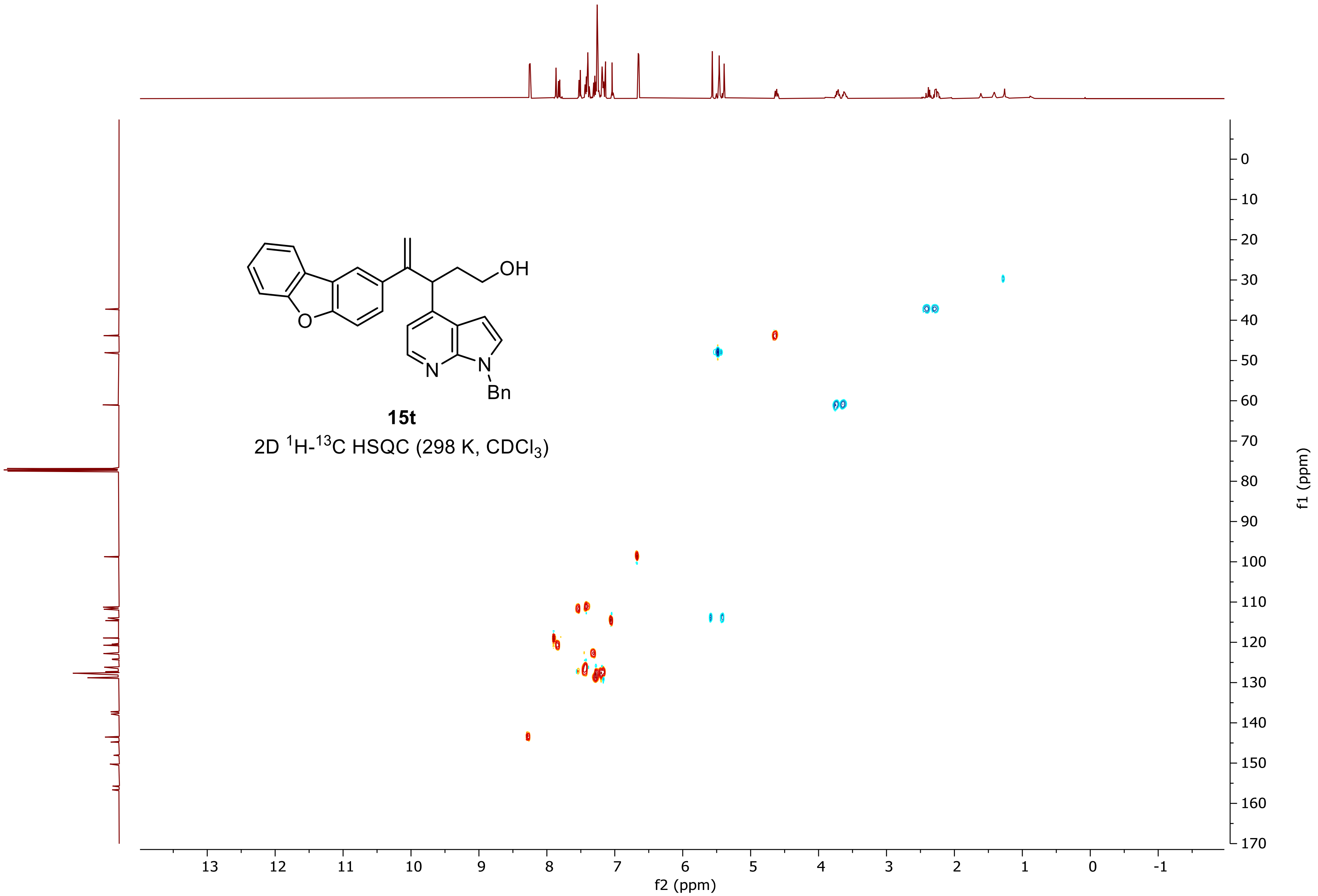
OH
O
N
N
Bn
15t
2D ¹H-¹³C HSQC (298 K, CDCl3)
13
12
11
10
9
8
7
6
5
4
3
2
1
0
-1
f2 (ppm)
0
10
20
30
40
50
60
70
80
90
100
110
120
130
140
150
160
170
f1 (ppm)

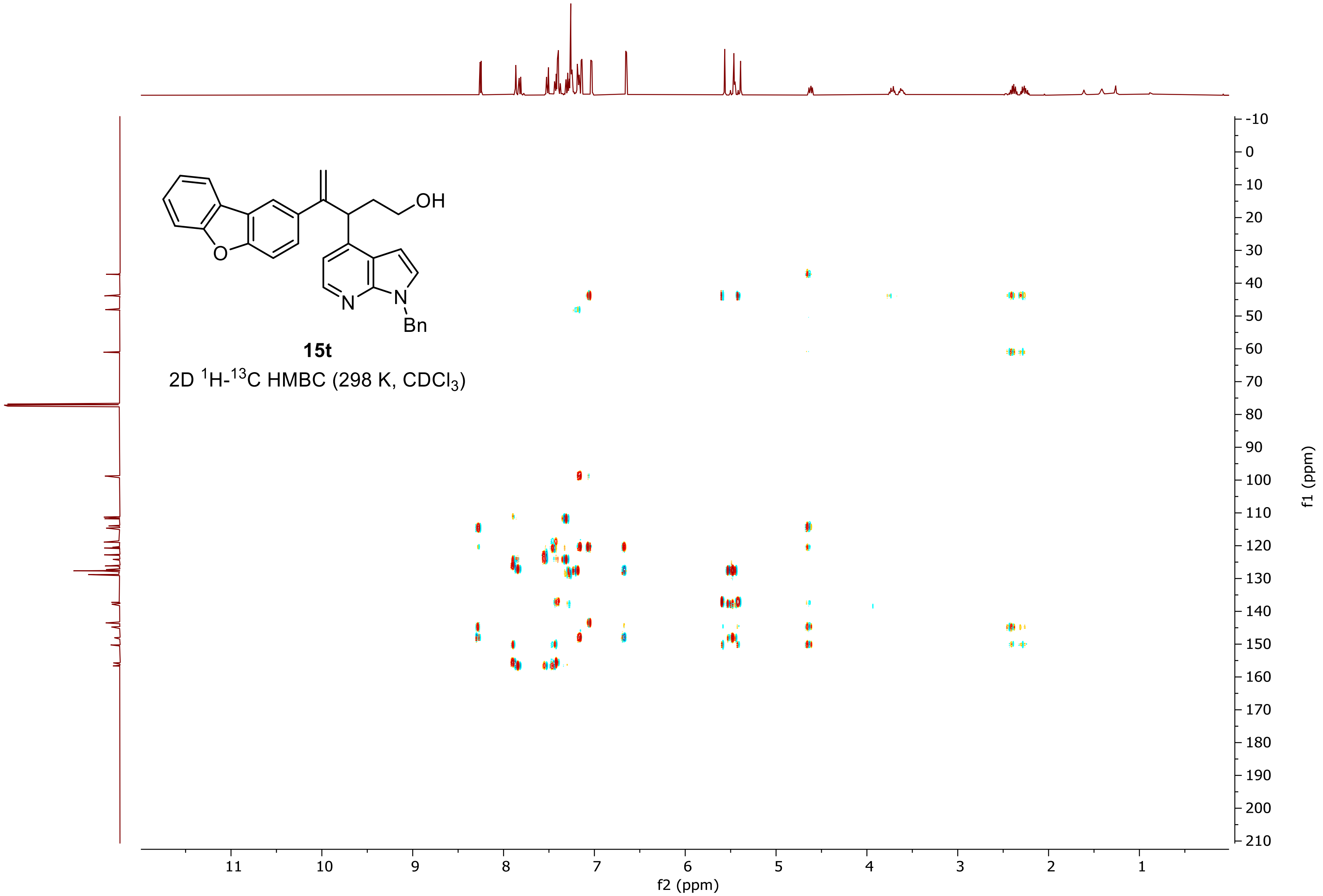
OH
O
N
N
Bn
15t
2D 1H-13C HMBC (298 K, CDCl3)
f1 (ppm)
f2 (ppm)

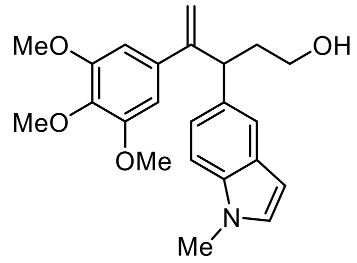

15u

^{1}H NMR (400 MHz, 298 K, $CDCl_3$)

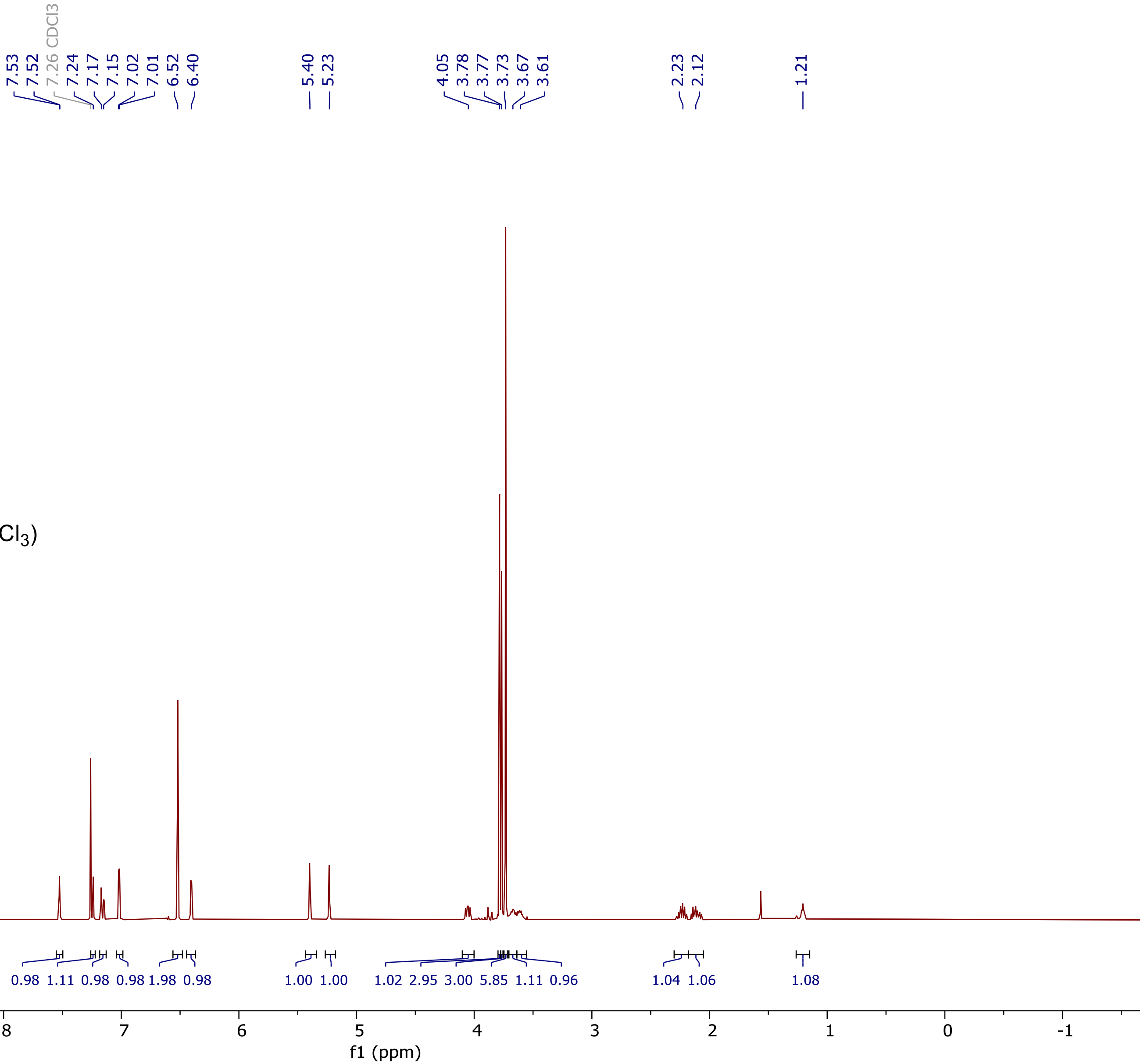

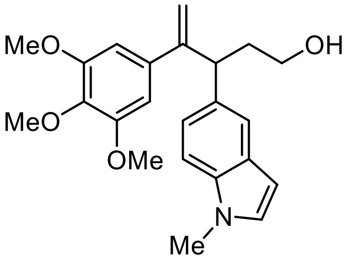

15u

$^{13}C\{^{1}H\}$ NMR (101 MHz, 298 K, $CDCl_3$)

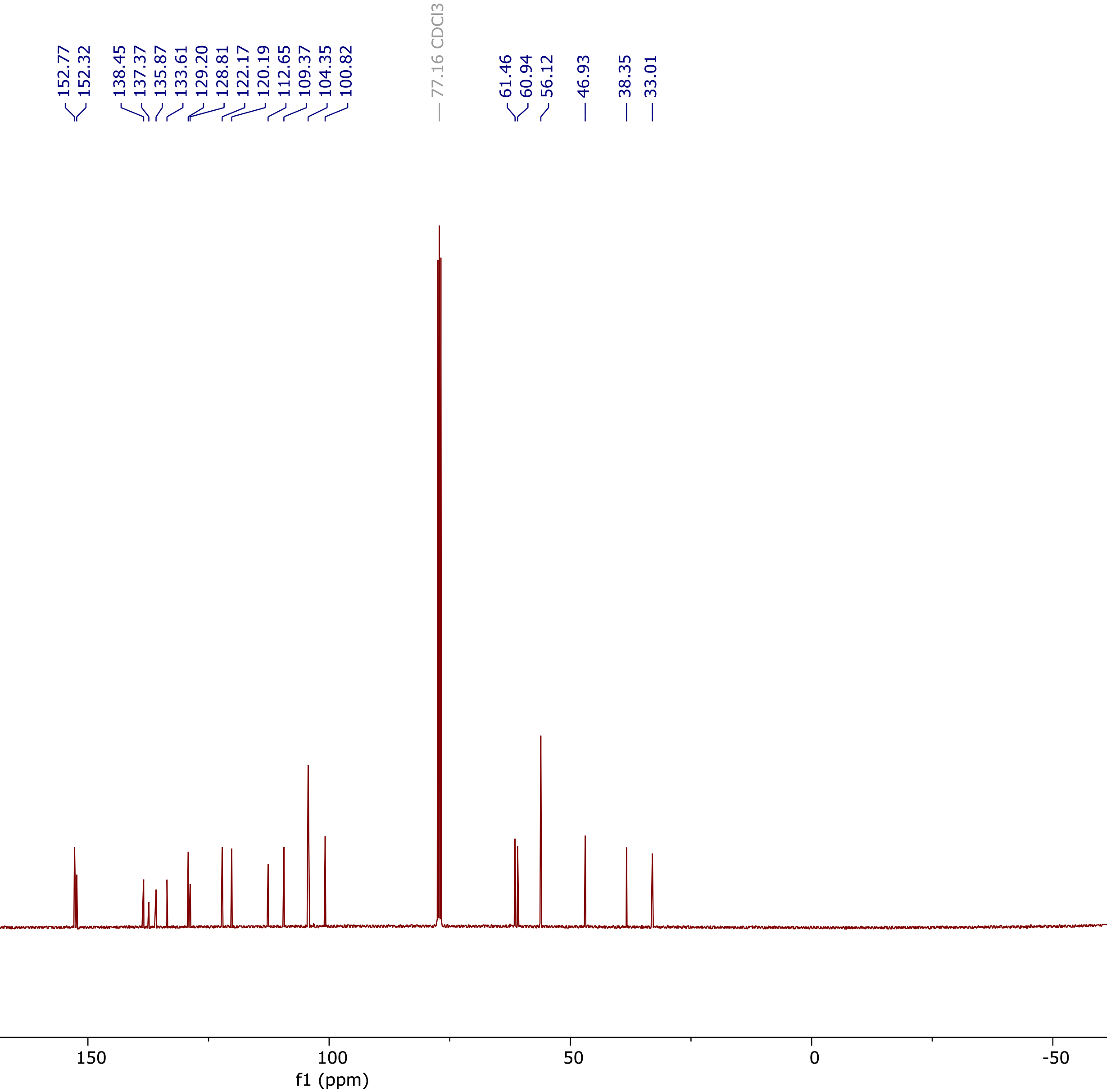

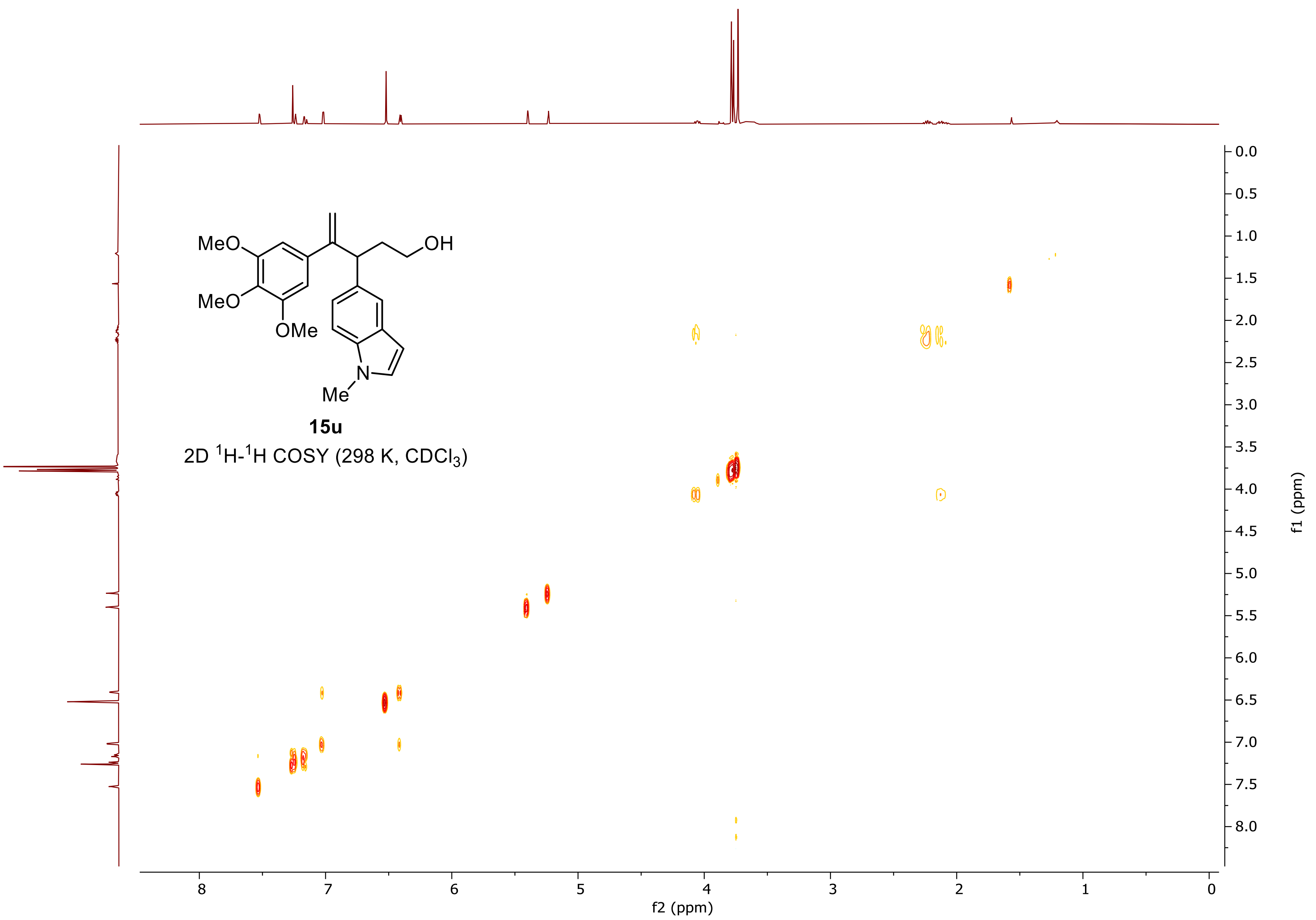
MeO
MeO
OMe
OH
N
Me
15u
2D 1H-1H COSY (298 K, CDCl3)
f1 (ppm)
f2 (ppm)

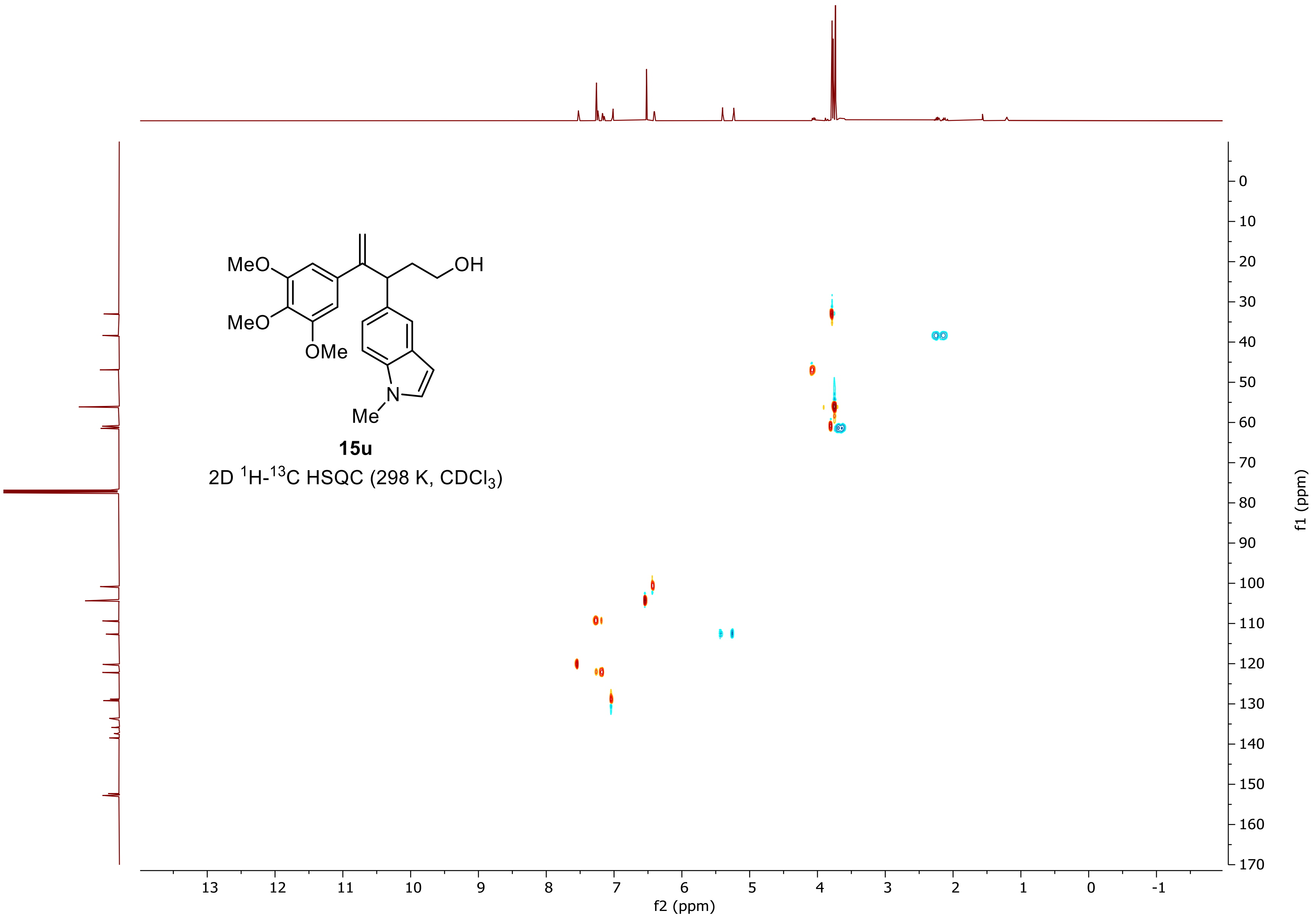
MeO
MeO
OMe
OH
N
Me
15u
2D 1H-13C HSQC (298 K, CDCl3)
f1 (ppm)
f2 (ppm)

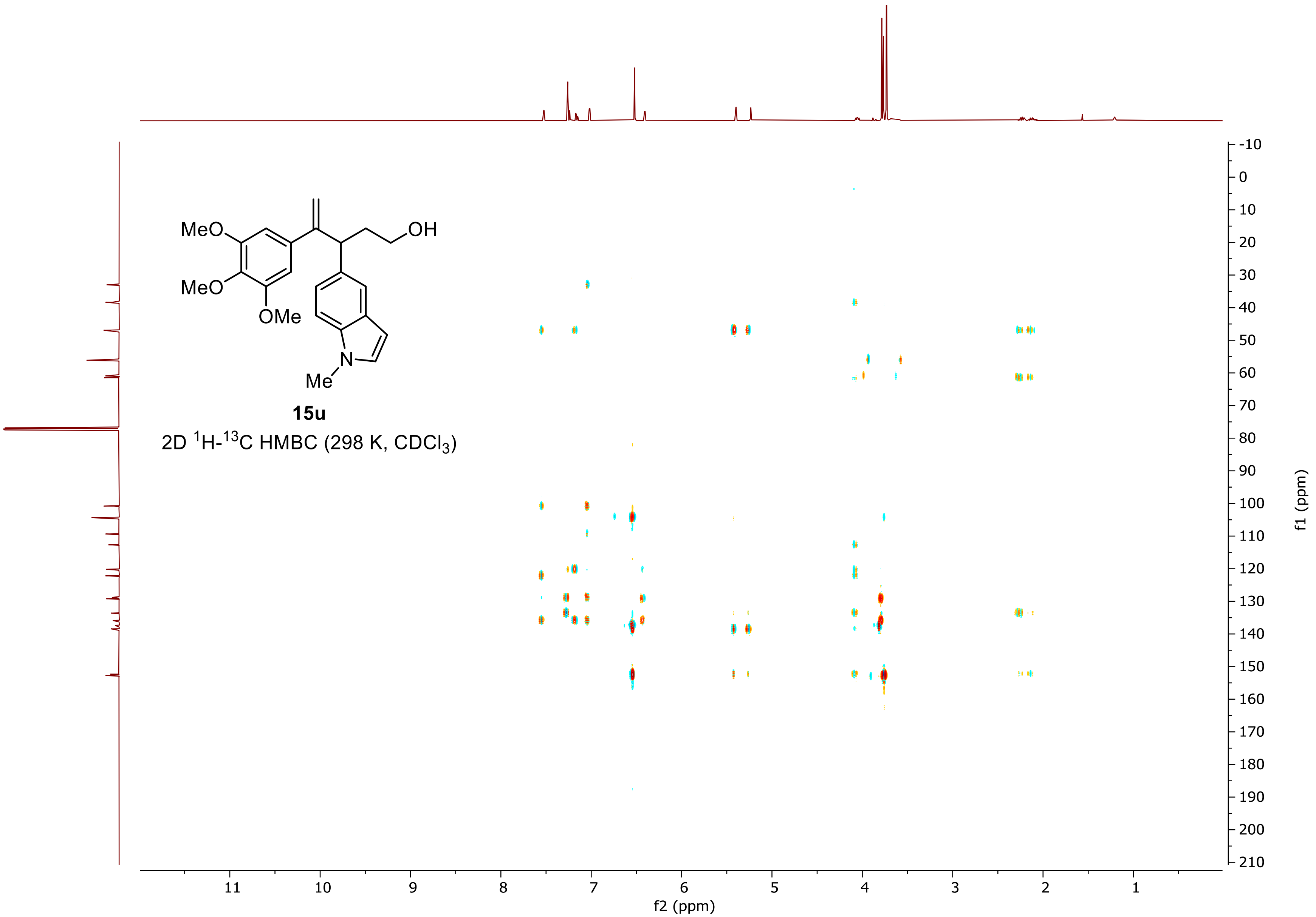

15u

2D ^{1}H-^{13}C HMBC (298 K, $CDCl_3$)

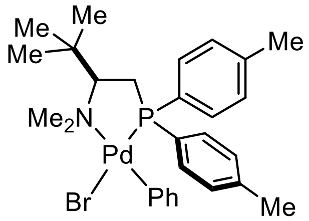

16

^{1}H NMR (400 MHz, 298 K, C_6D_6)

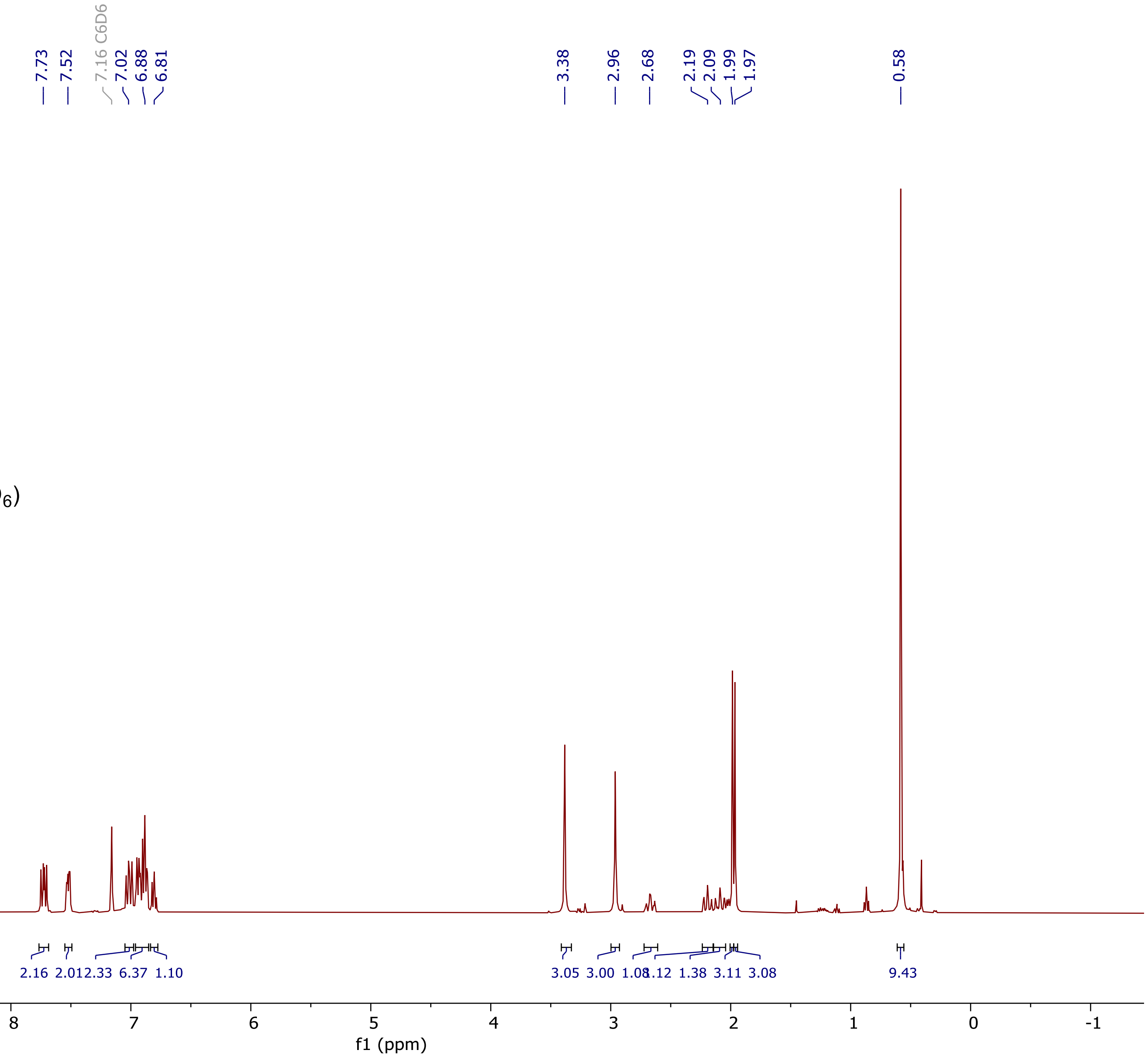

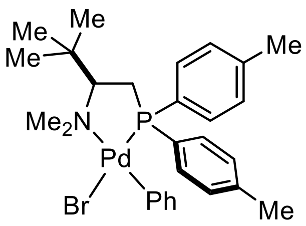

16

$^{13}C\{^{1}H\}$ NMR (101 MHz, 298 K, C_6D_6)

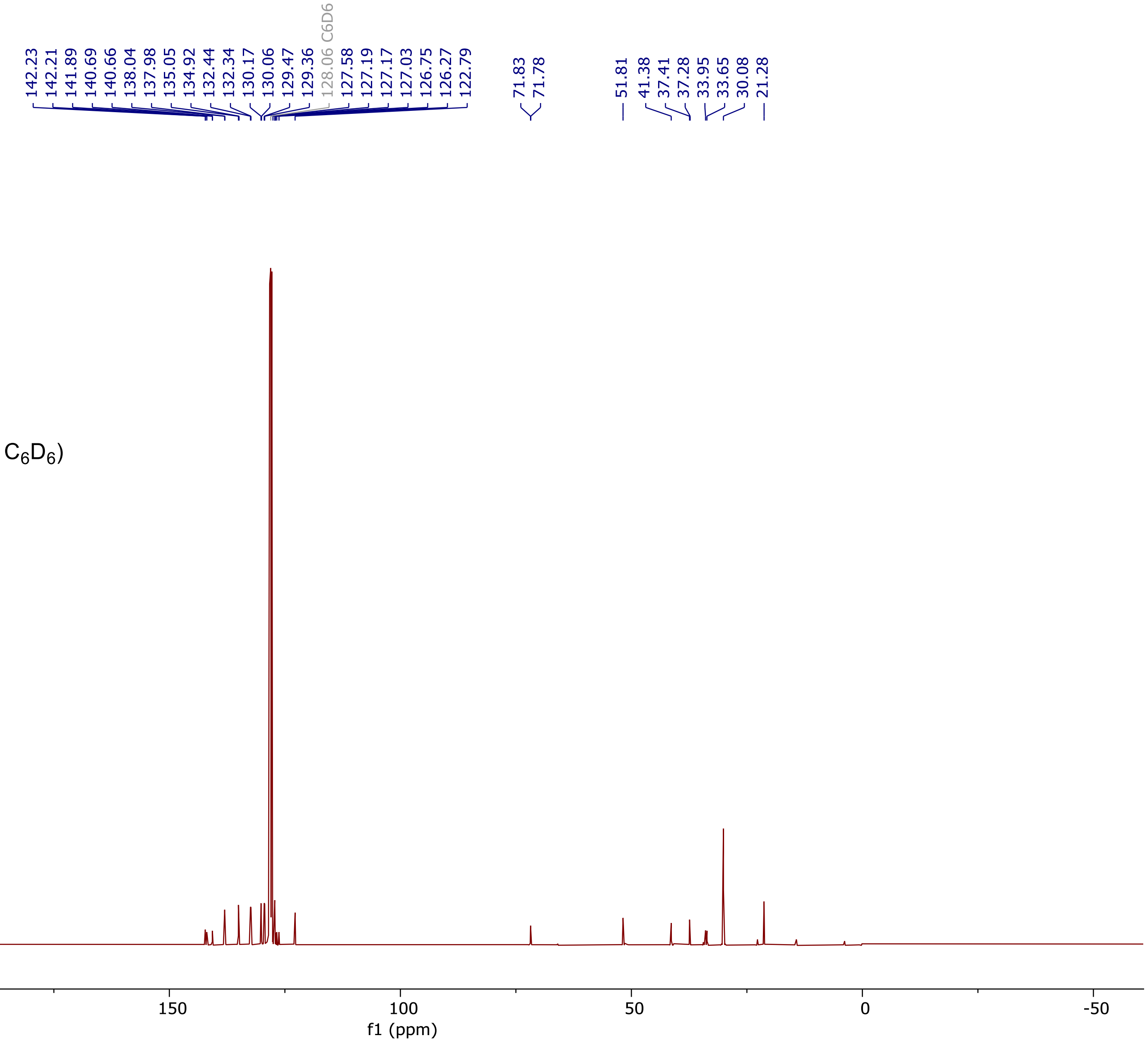

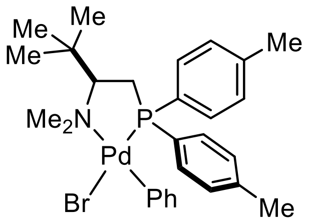

16

$^{31}P\{^{1}H\}$ NMR (162 MHz, 298 K, C_6D_6)

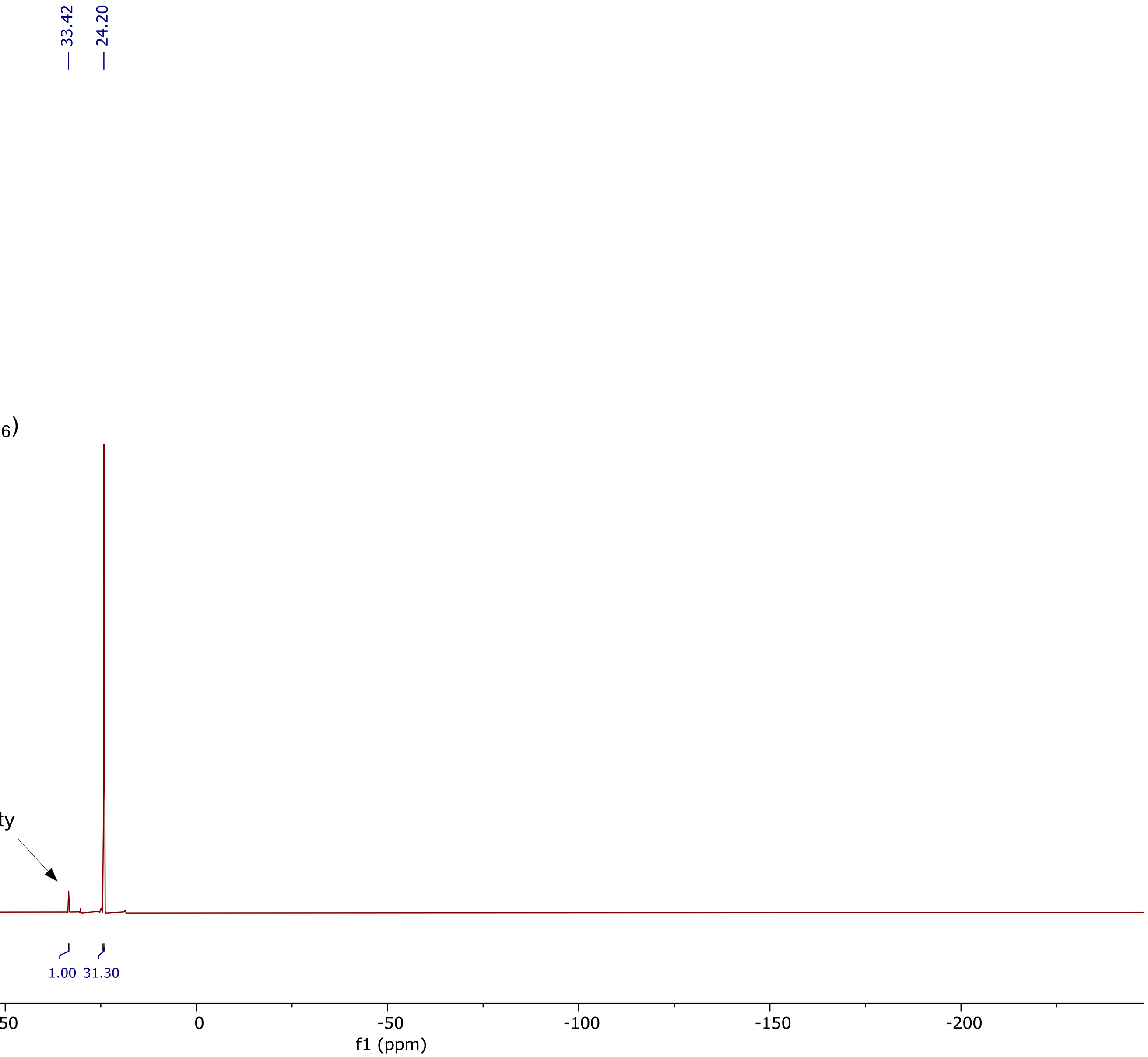

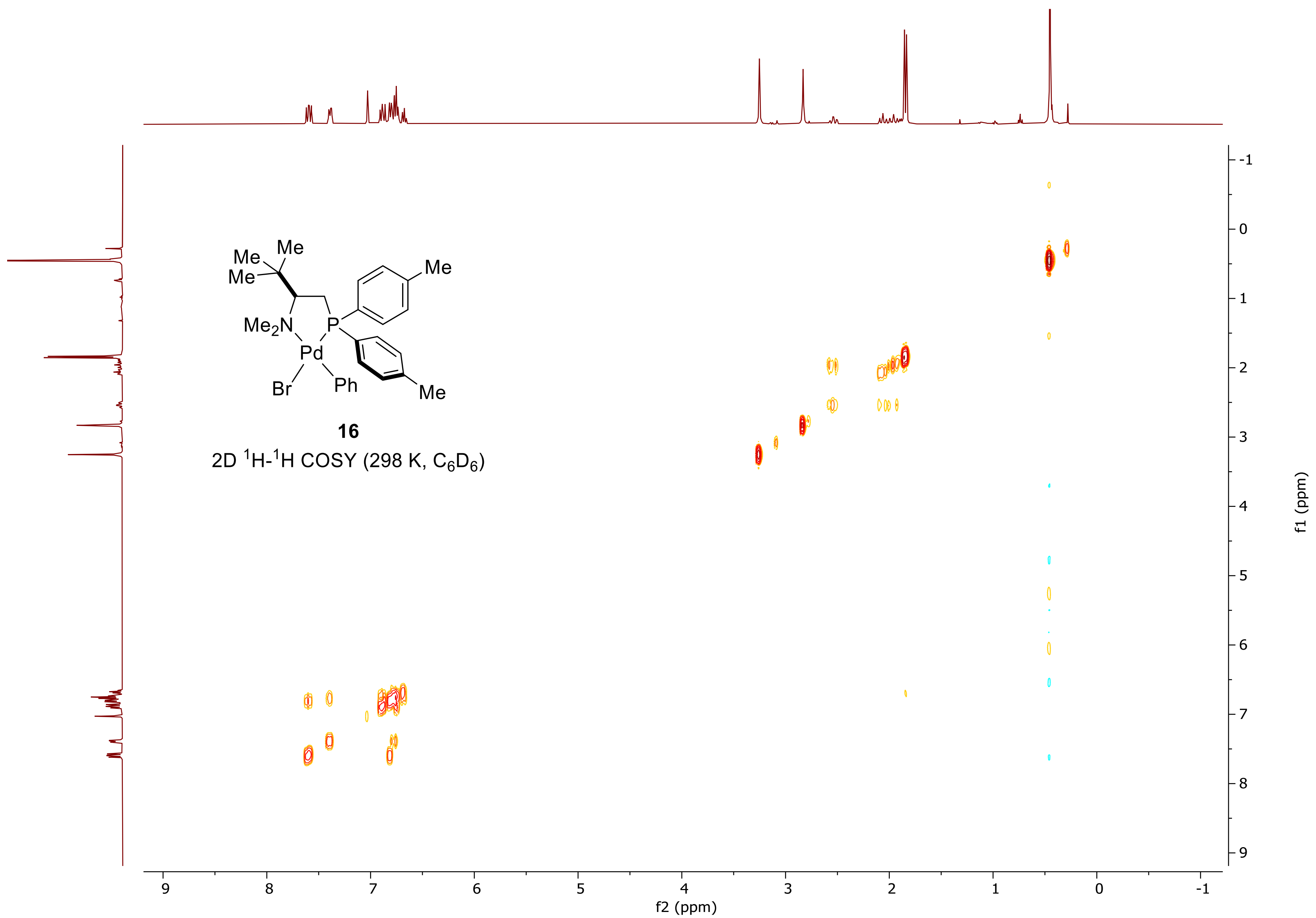

16

2D ^{1}H-^{1}H COSY (298 K, C_6D_6)

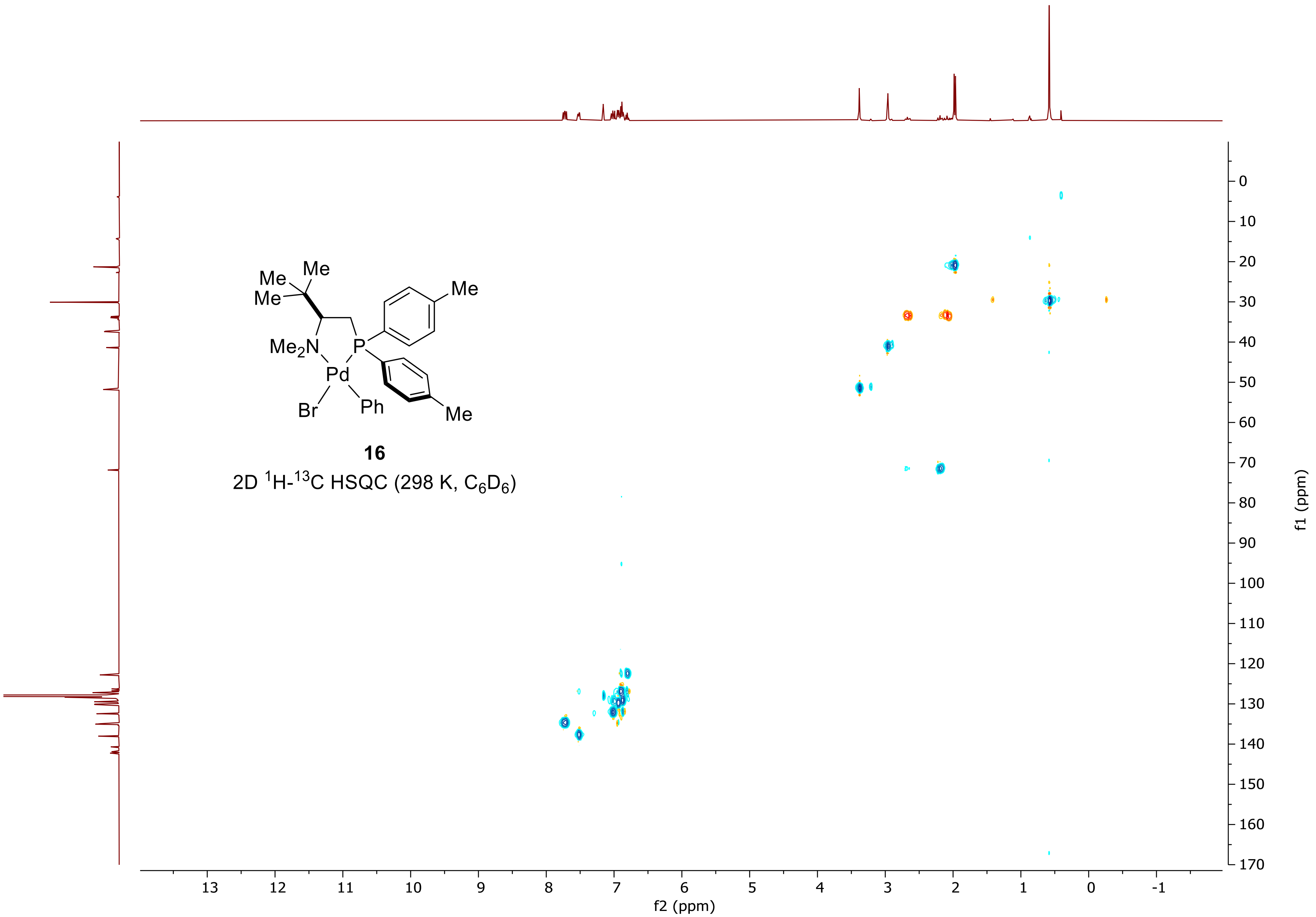
Me
Me
Me
Me
Me2N
P
Pd
Br
Ph
Me
16
2D 1H-13C HSQC (298 K, C6D6)
f2 (ppm)
f1 (ppm)

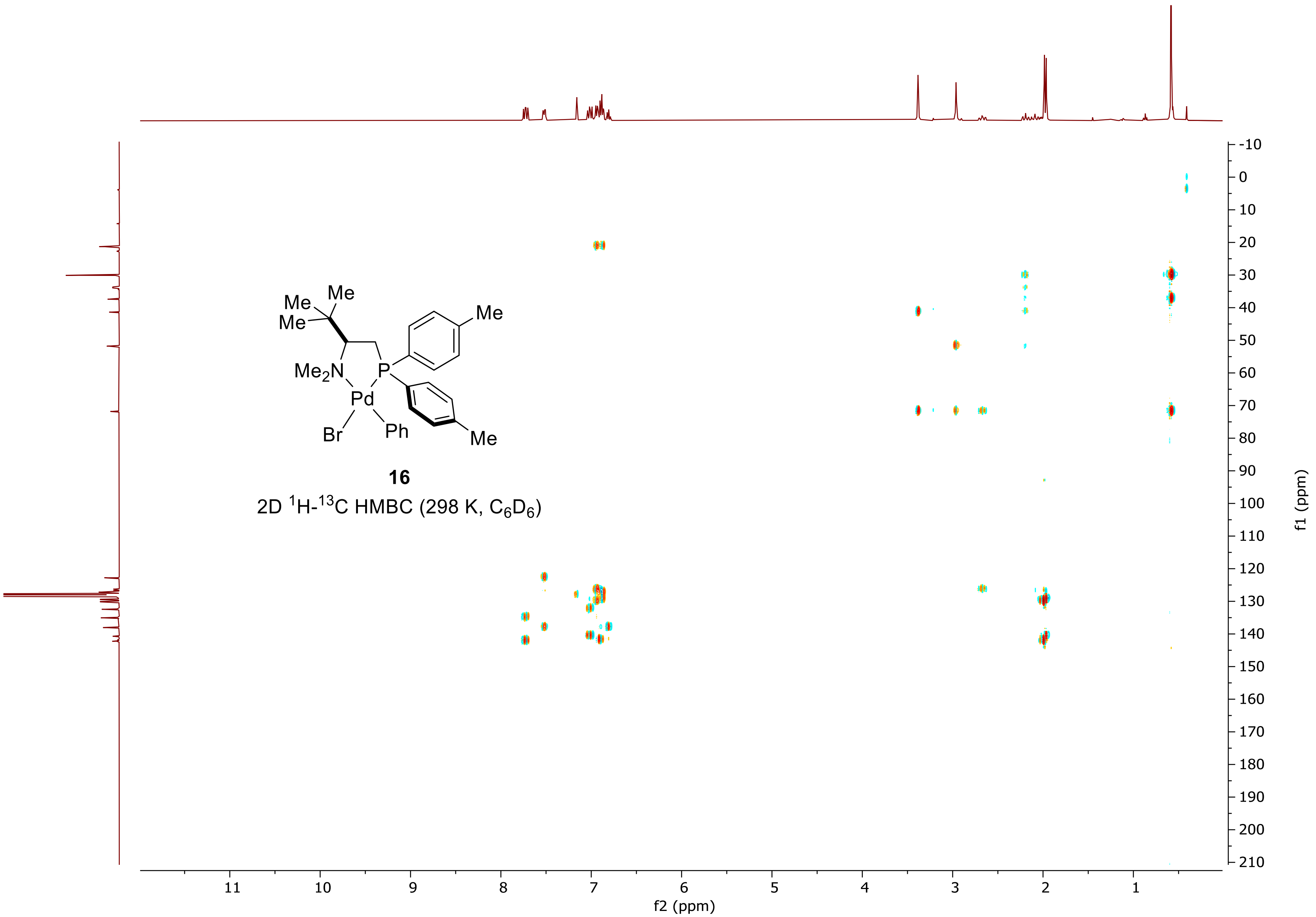

16

2D ^{1}H-^{13}C HMBC (298 K, C_6D_6)

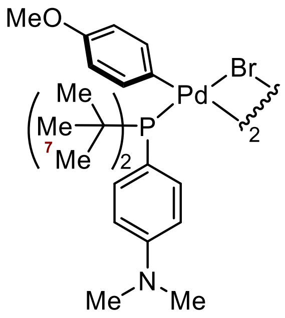

17

^{1}H NMR (400 MHz, 298 K, THF-d_8)

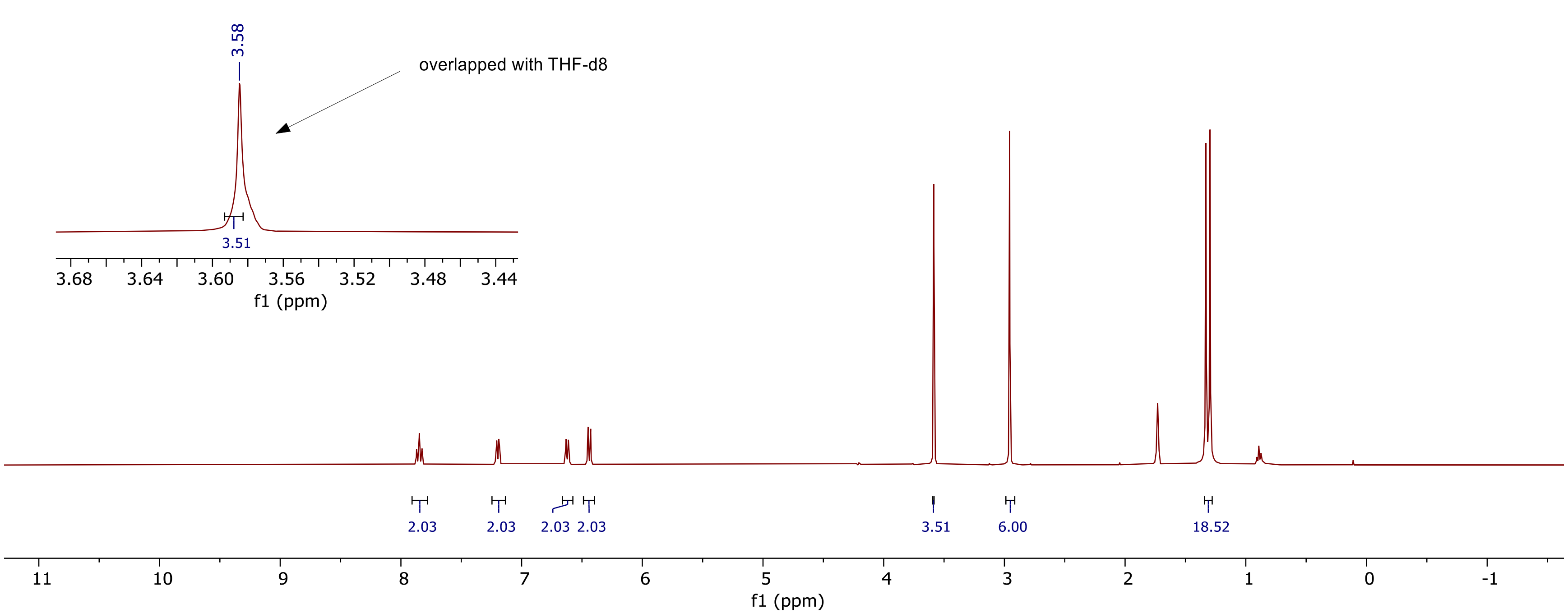

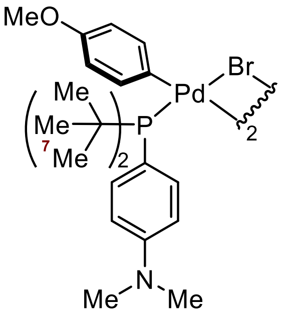

17

$^{13}C\{^{1}H\}$ NMR (126 MHz, 298 K, THF-d_8)

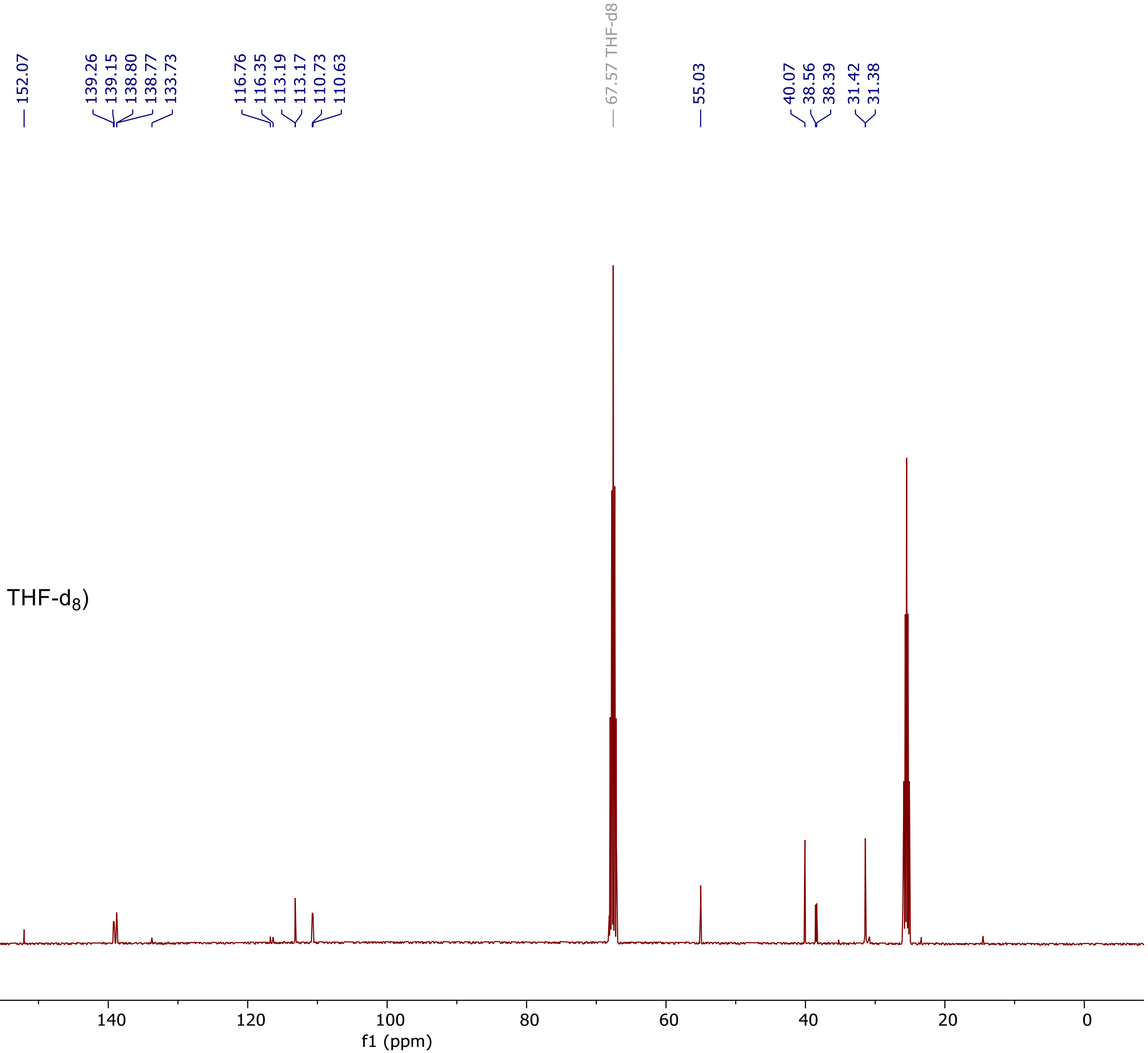

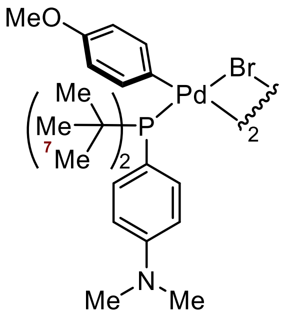

17

$^{31}P\{^{1}H\}$ NMR (126 MHz, 298 K, THF-d_8)

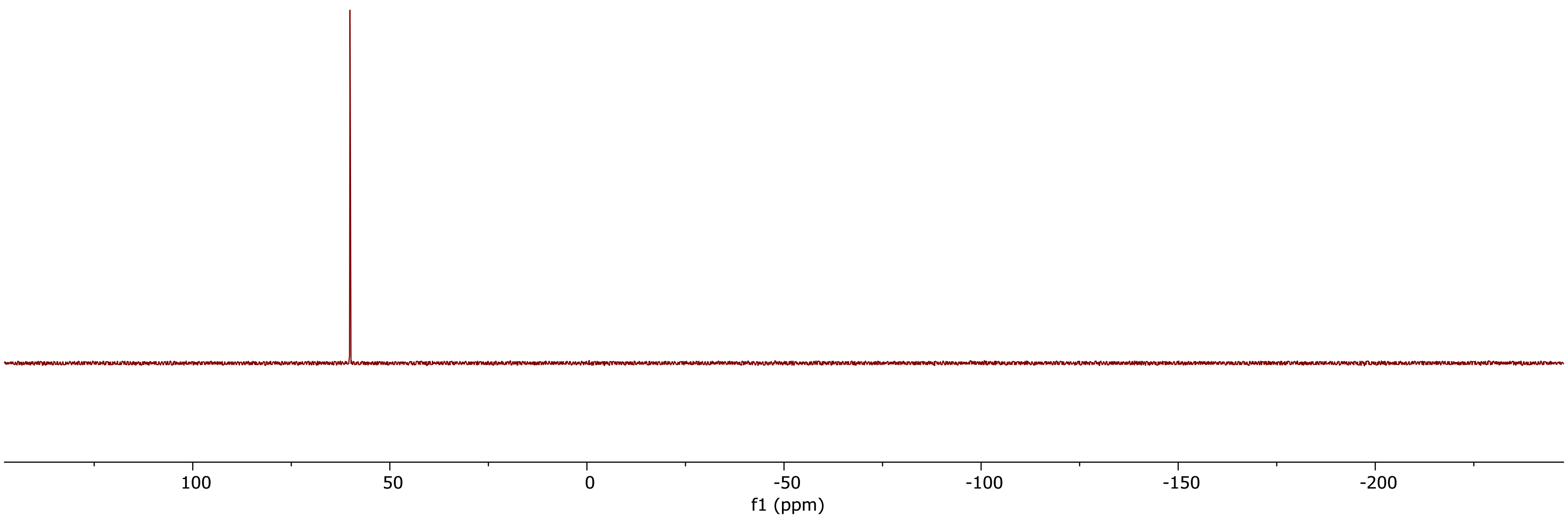

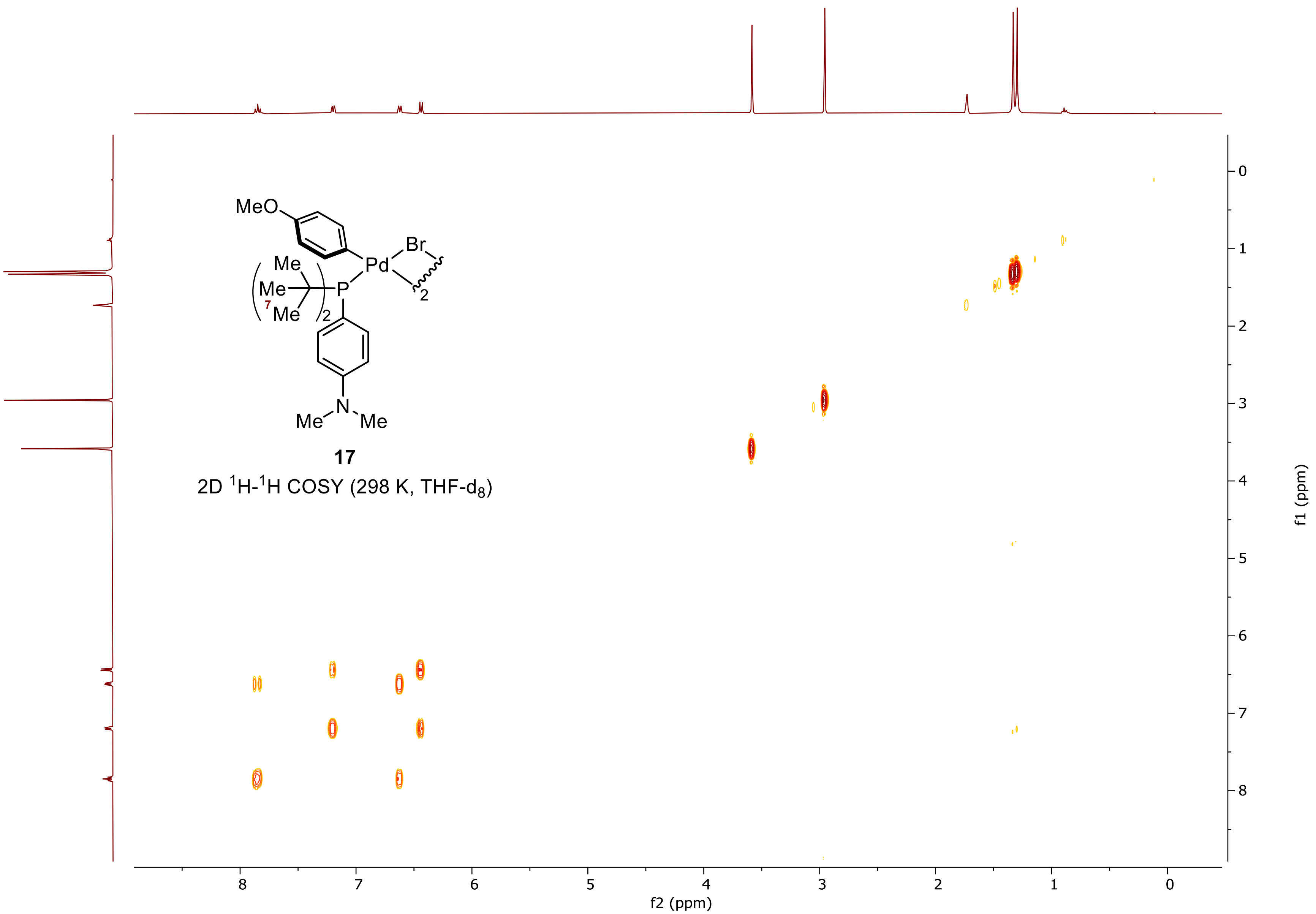

17

2D ^1H-^1H COSY (298 K, THF-d_8)

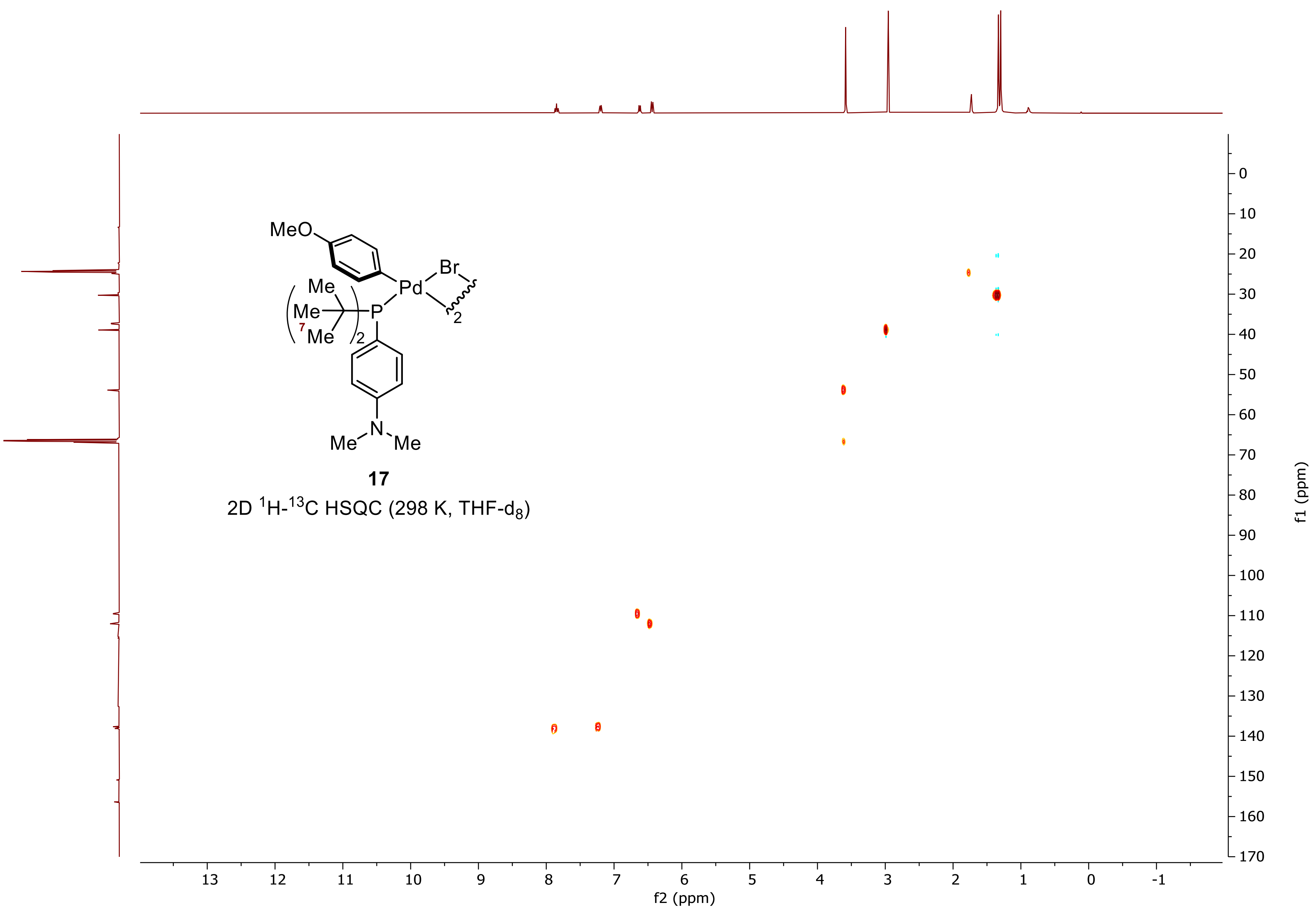
MeO
Br
Pd
Me
Me
Me
P
2
2
7
N
Me
Me
17
2D ^{1}H-^{13}C HSQC (298 K, THF-d$_8$)
f2 (ppm)
f1 (ppm)

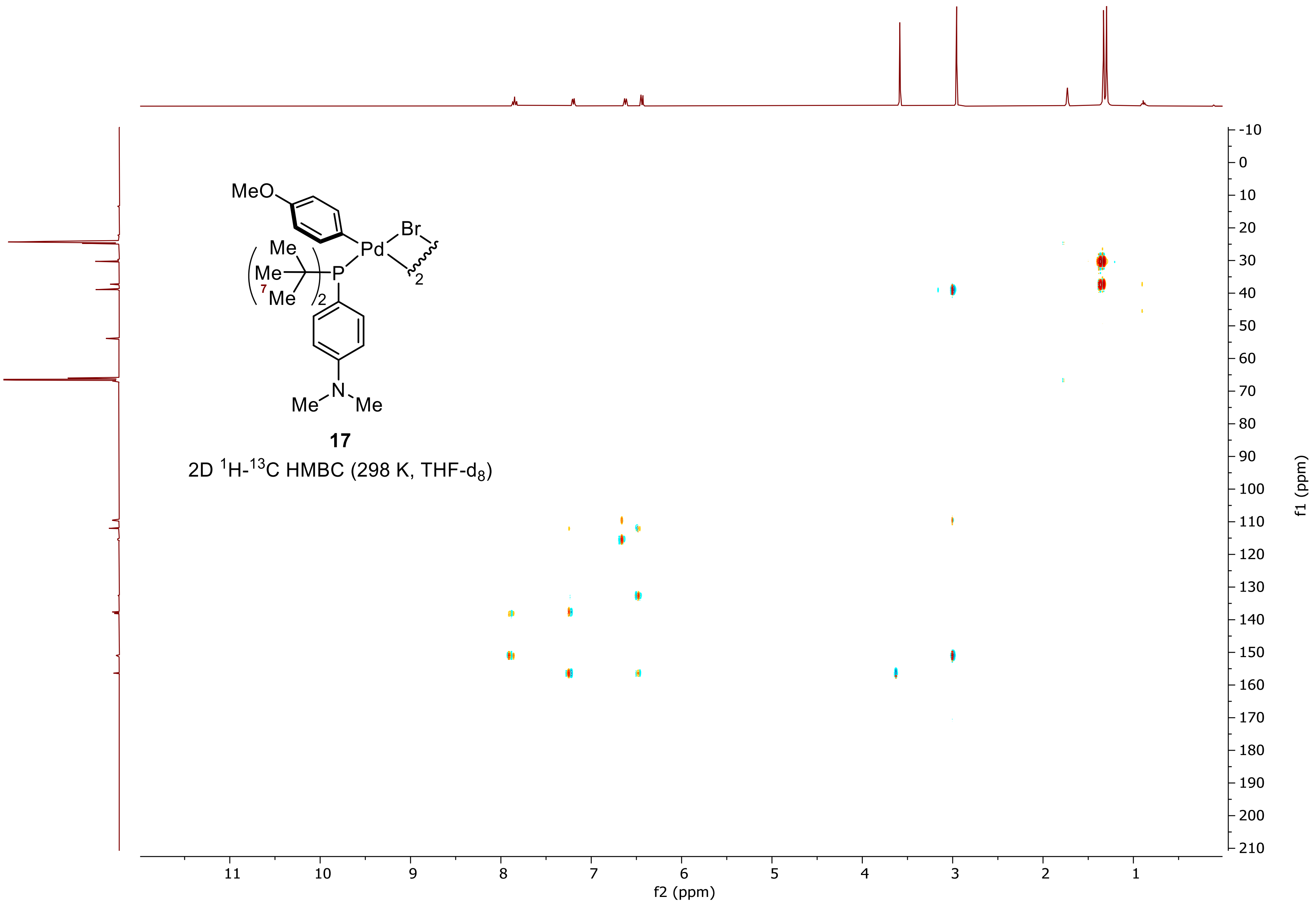
MeO
Br
Pd
Me
Me
P
7
Me
2
2
N
Me
Me
17
2D 1H-13C HMBC (298 K, THF-d8)
f1 (ppm)
f2 (ppm)

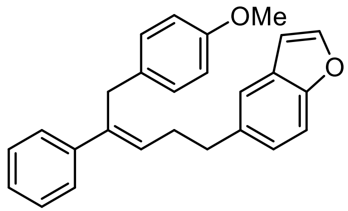

18

^{1}H NMR (400 MHz, 298 K, $CDCl_3$)

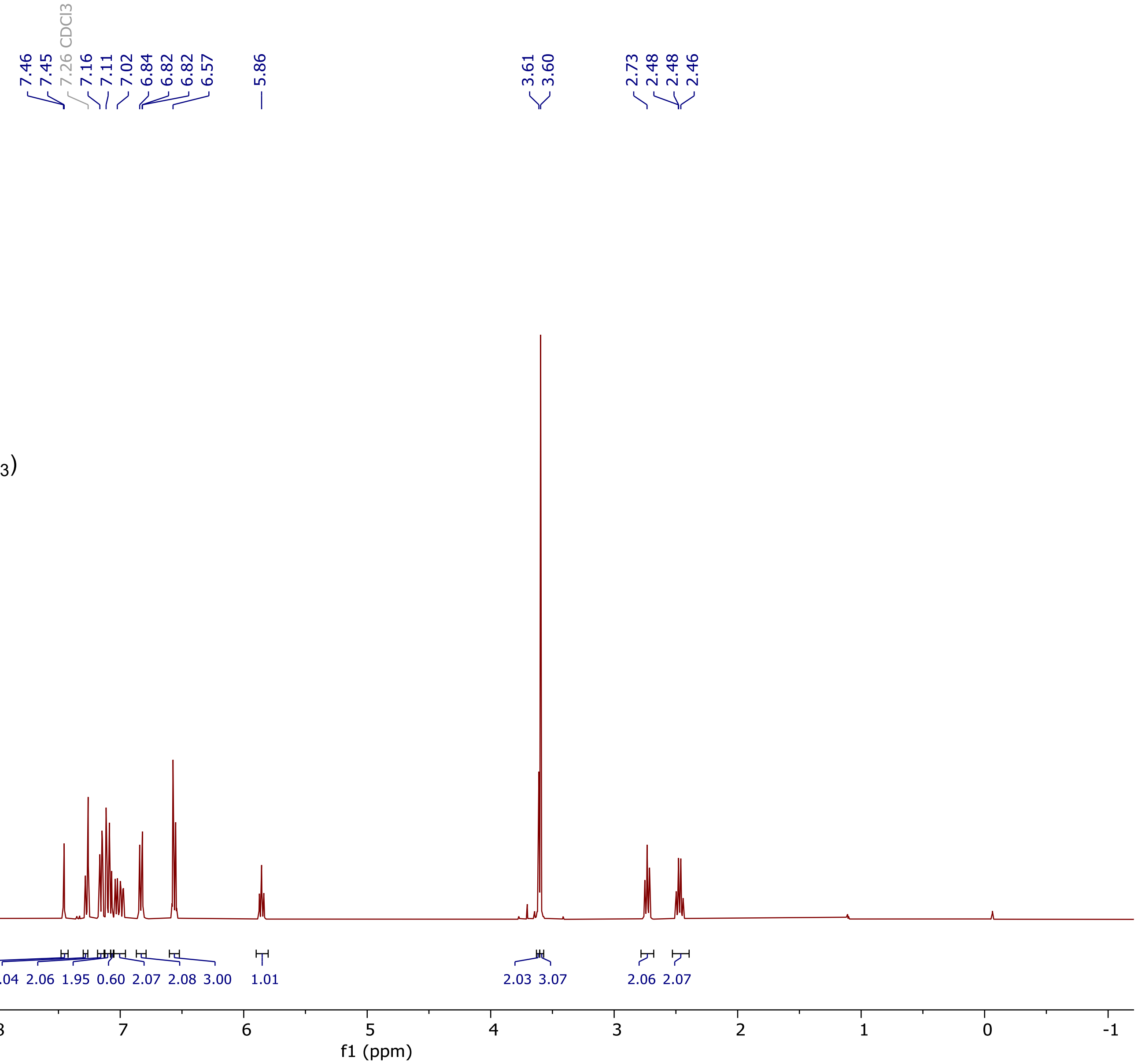

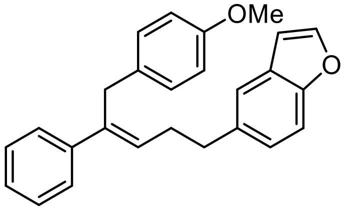

18

$^{13}C\{^{1}H\}$ NMR (101 MHz, 298 K, $CDCl_3$)

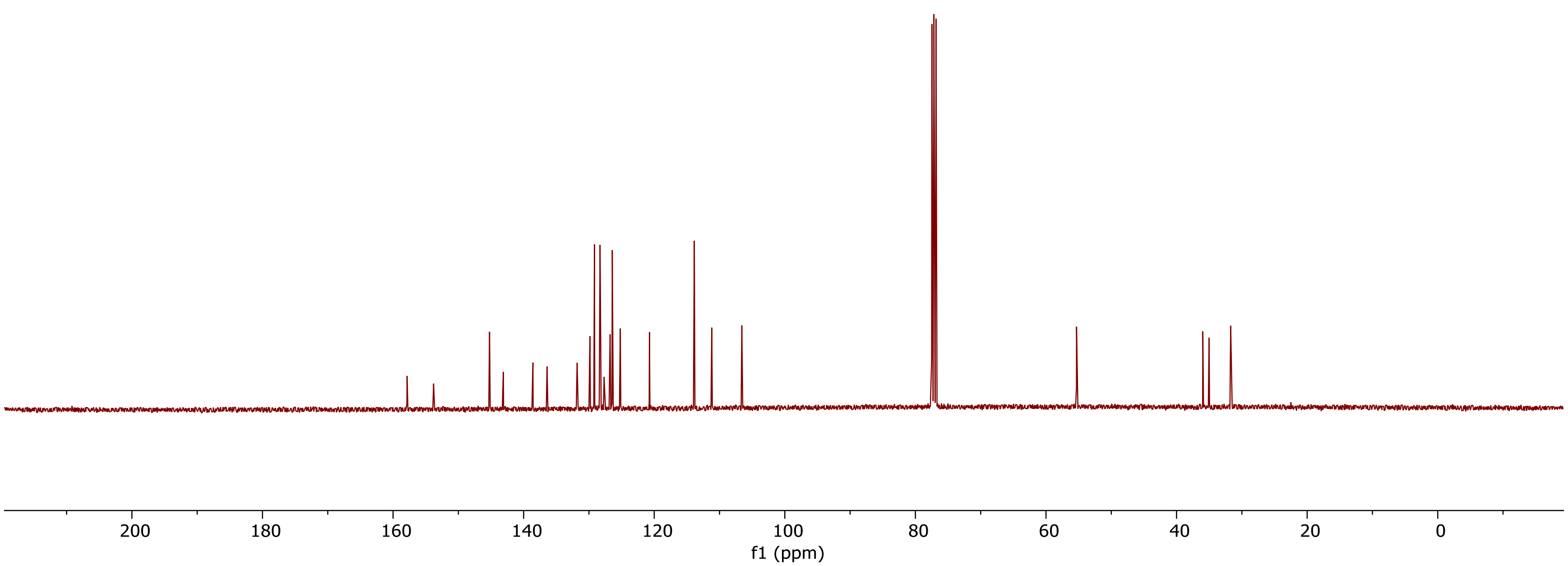

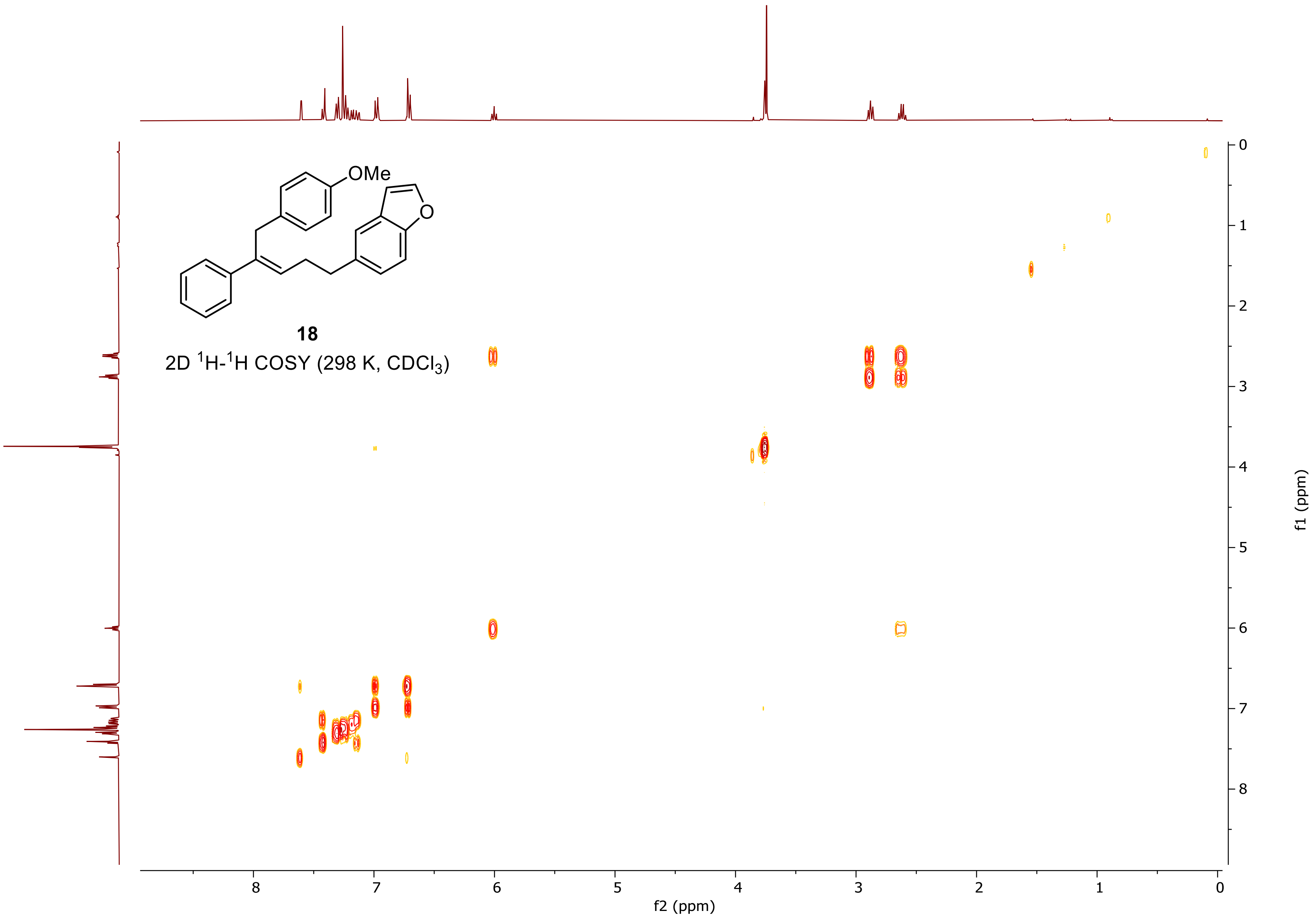

18

2D ^{1}H-^{1}H COSY (298 K, $CDCl_3$)

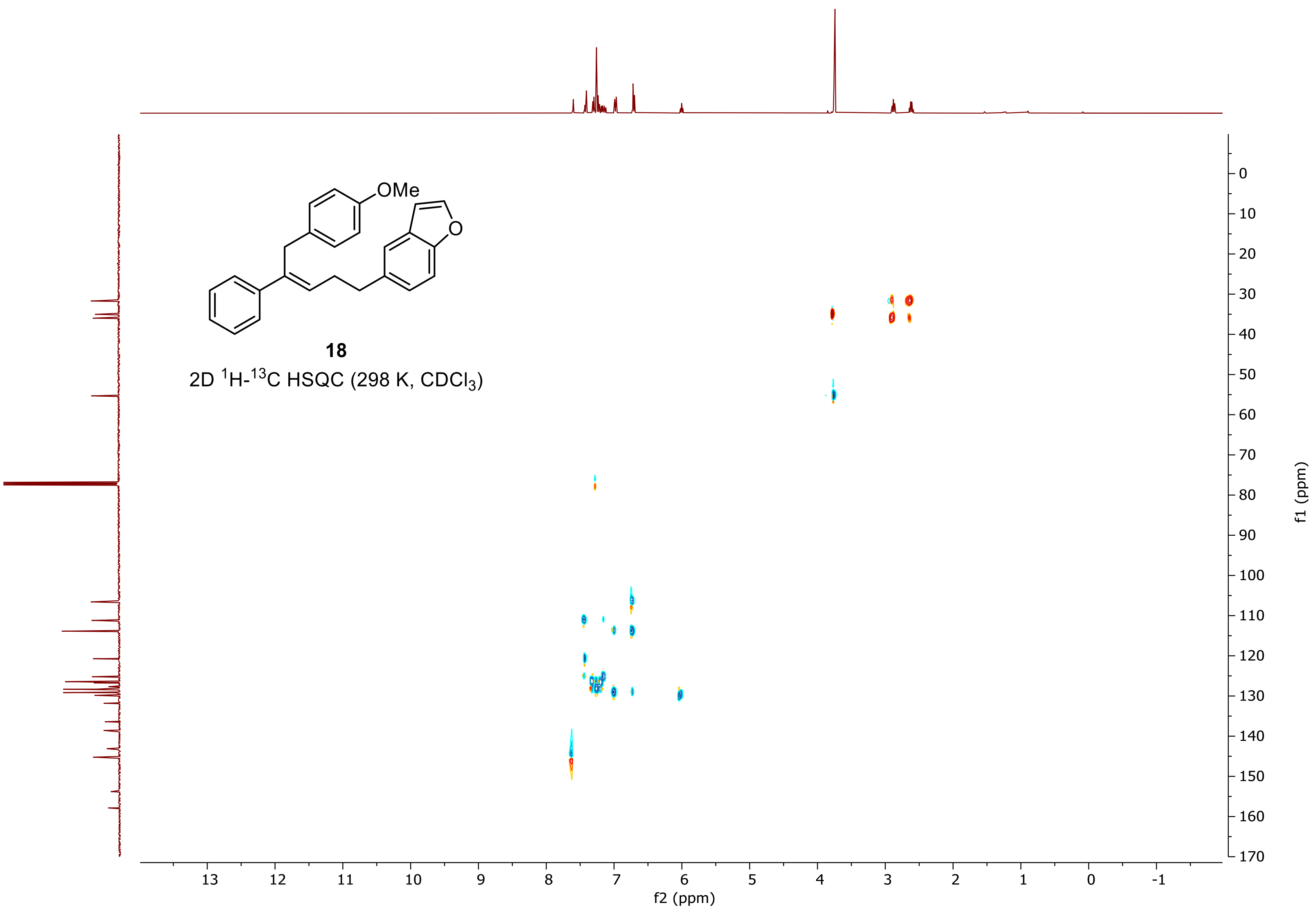
OMe
O
18
2D ¹H-¹³C HSQC (298 K, CDCl₃)
f2 (ppm)
f1 (ppm)

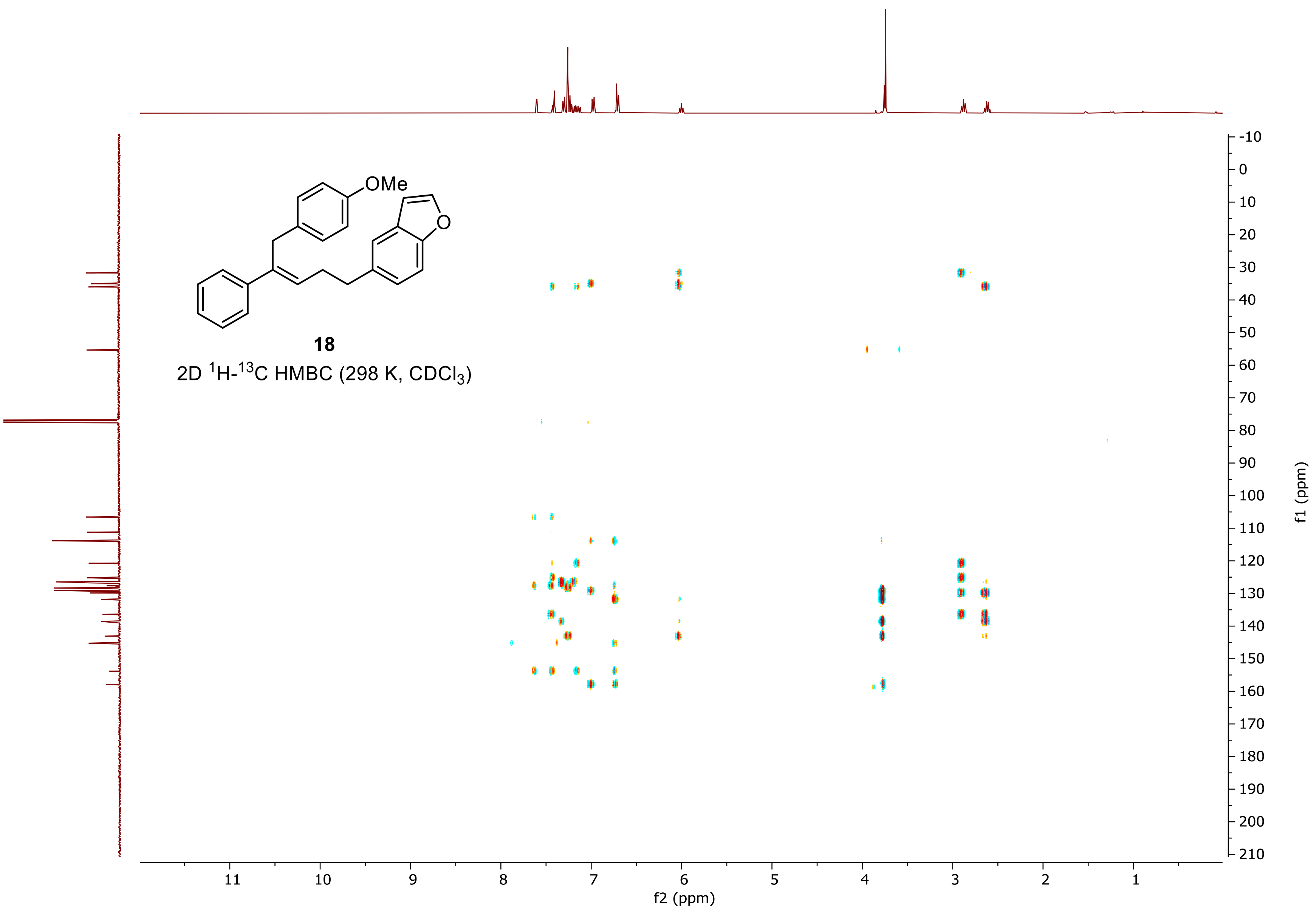
OMe
O
18
2D ^{1}H-^{13}C HMBC (298 K, $CDCl_3$)
f2 (ppm)
f1 (ppm)

13. NMR spectra for known compounds

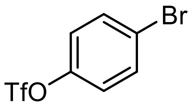

10g

^{1}H NMR (400 MHz, 298 K, $CDCl_3$)

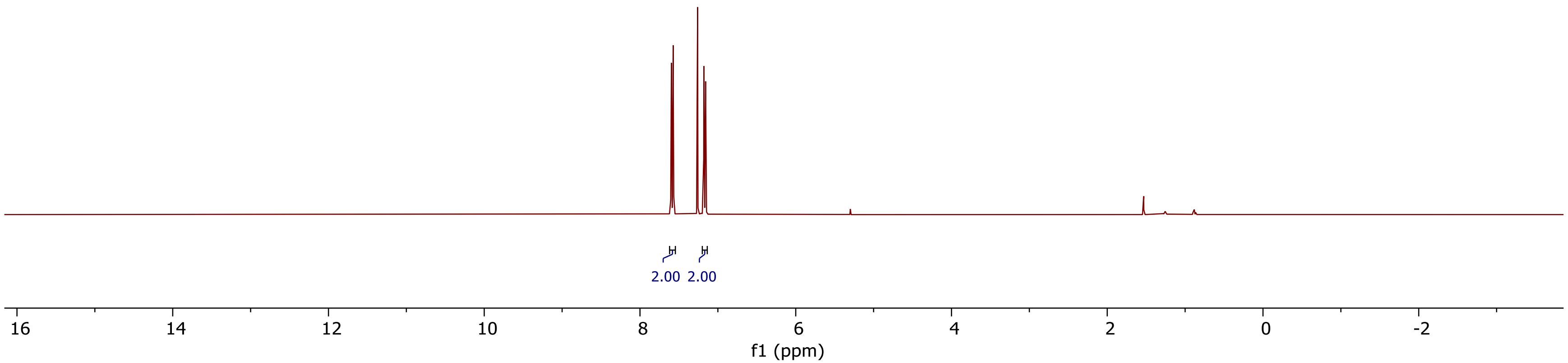

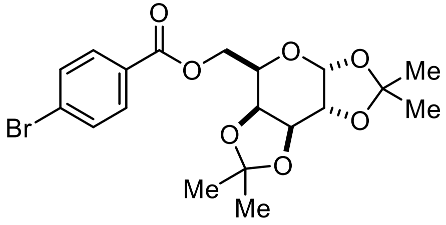

10j

^{1}H NMR (400 MHz, 298 K, $CDCl_3$)

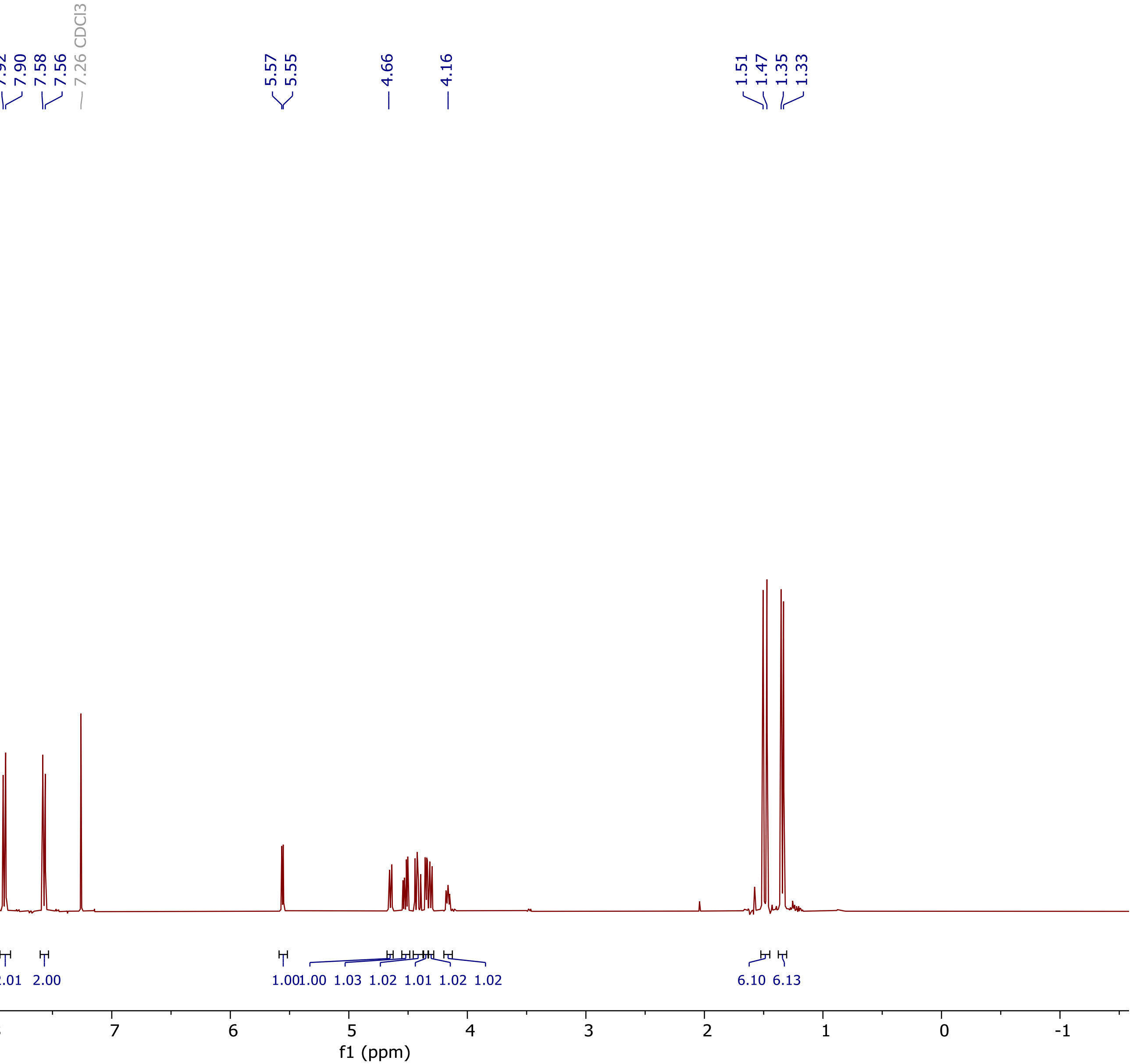

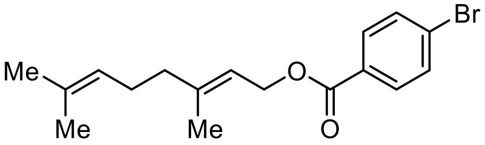

10k

^{1}H NMR (400 MHz, 298 K, $CDCl_3$)

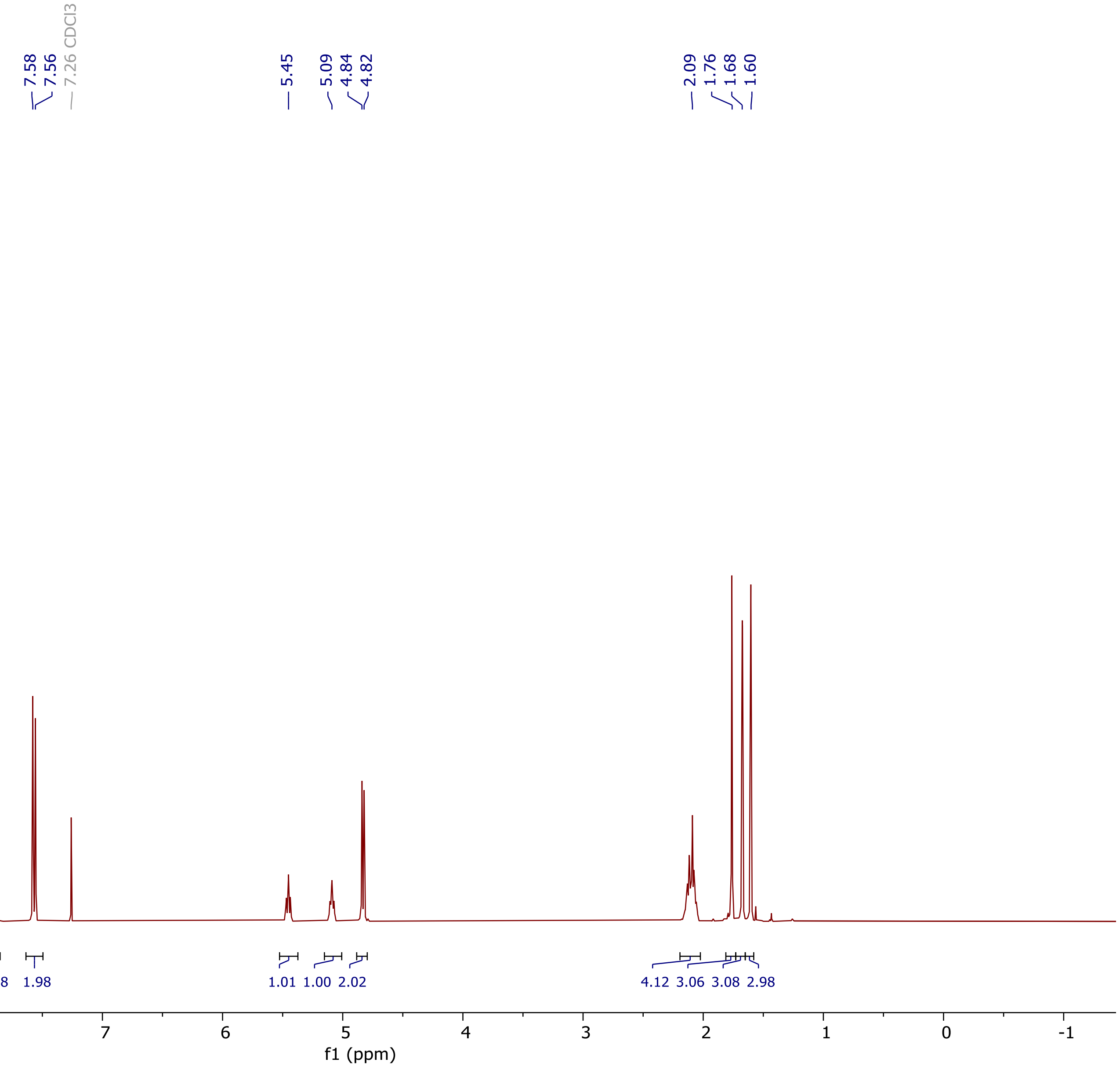

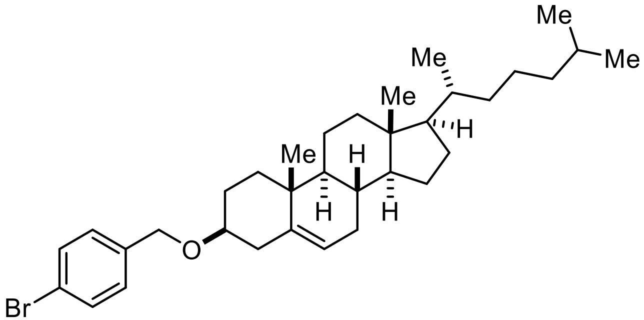

10l

^1H NMR (400 MHz, 298 K, $CDCl_3$)

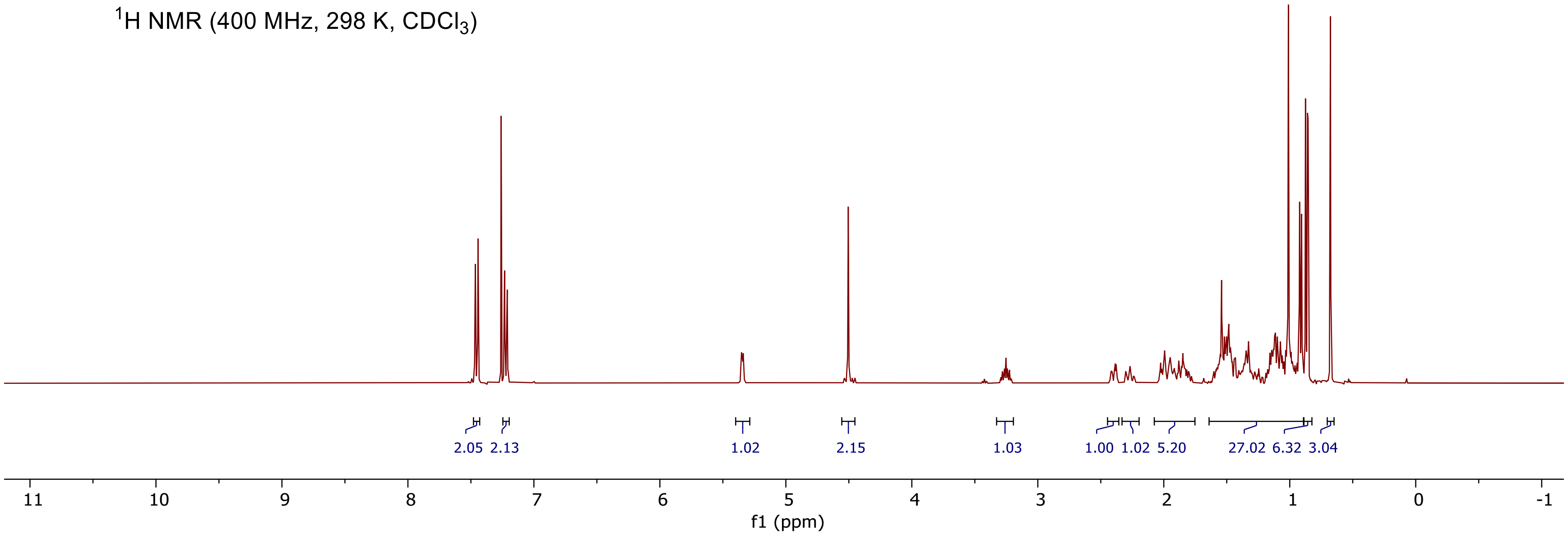

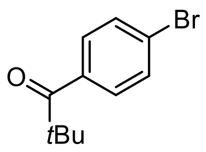

10r

^{1}H NMR (400 MHz, 298 K, $CDCl_3$)

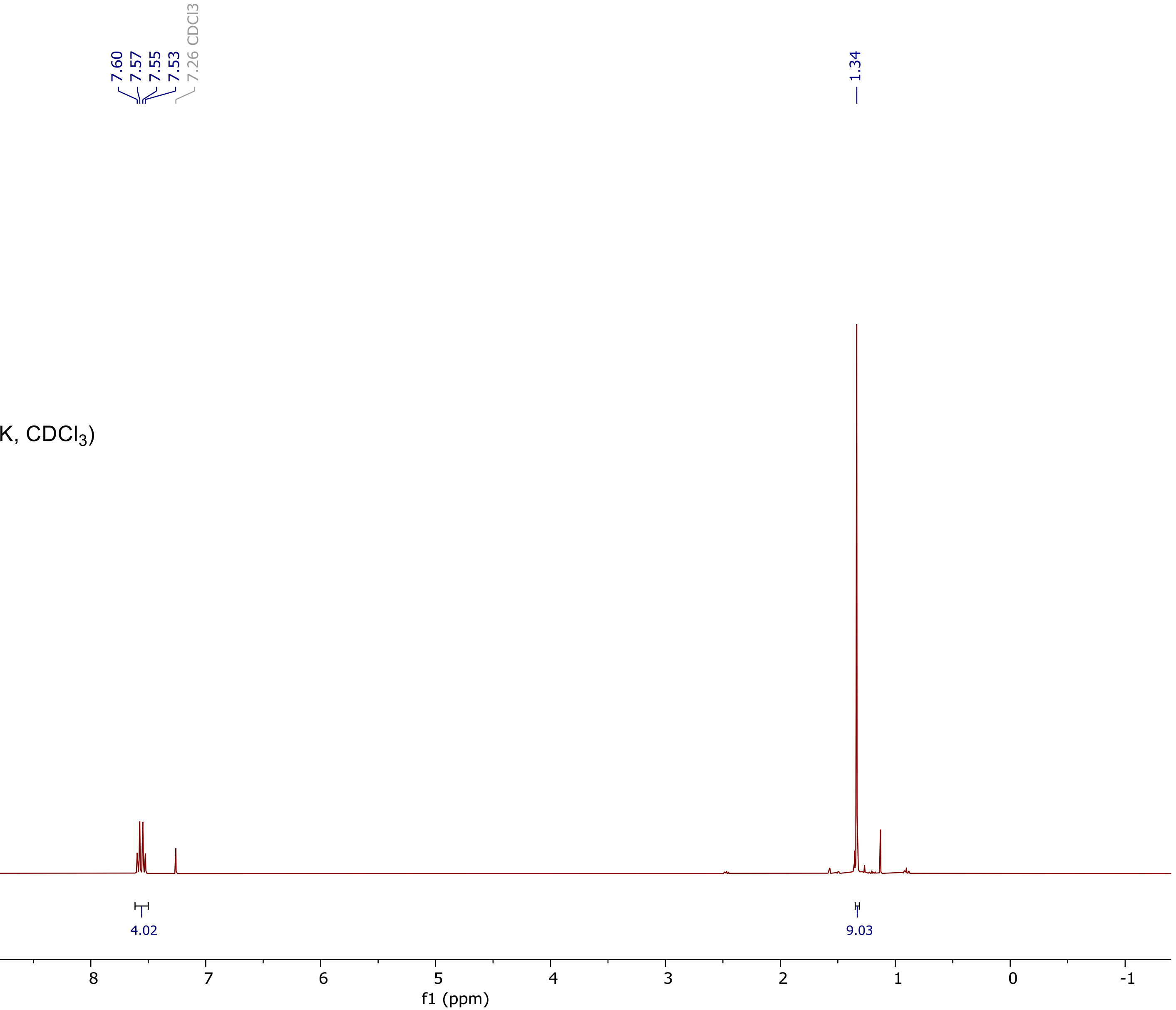

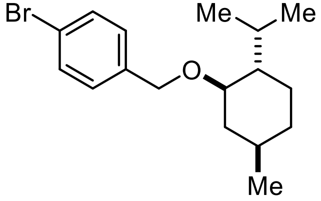

10s

^{1}H NMR (400 MHz, 298 K, $CDCl_3$)

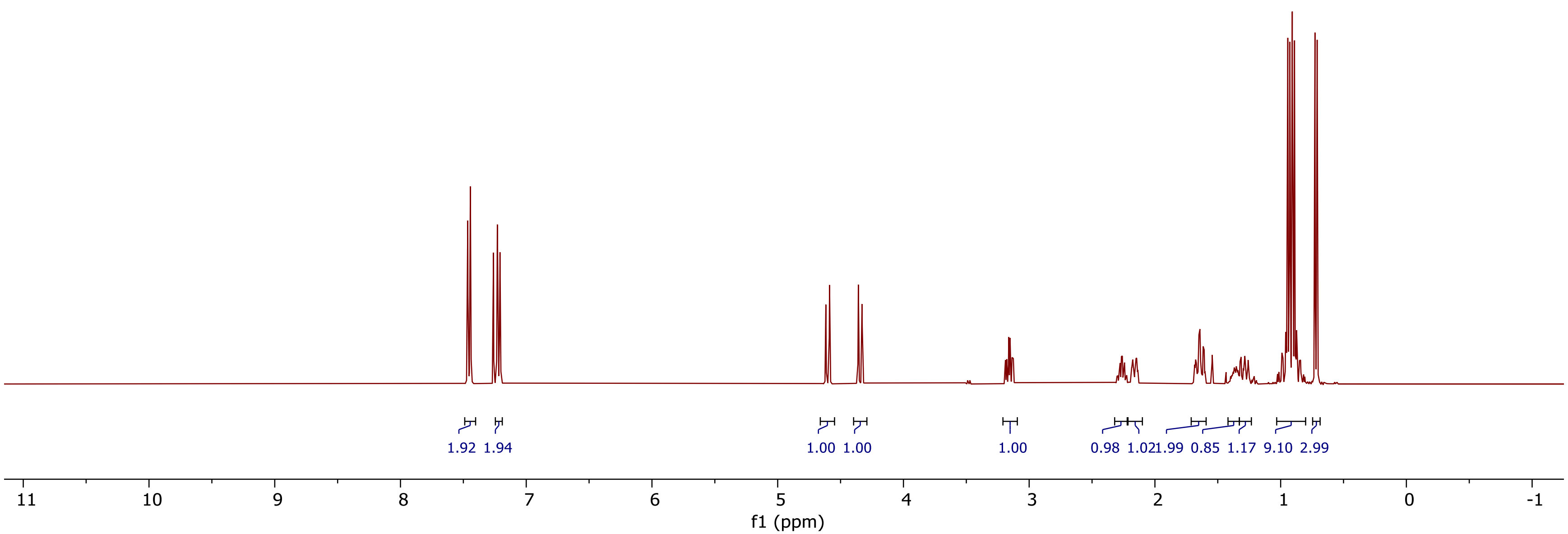

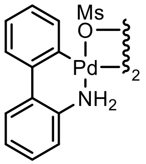

Pd1

^{1}H NMR (400 MHz, 298 K, CD_3CN)

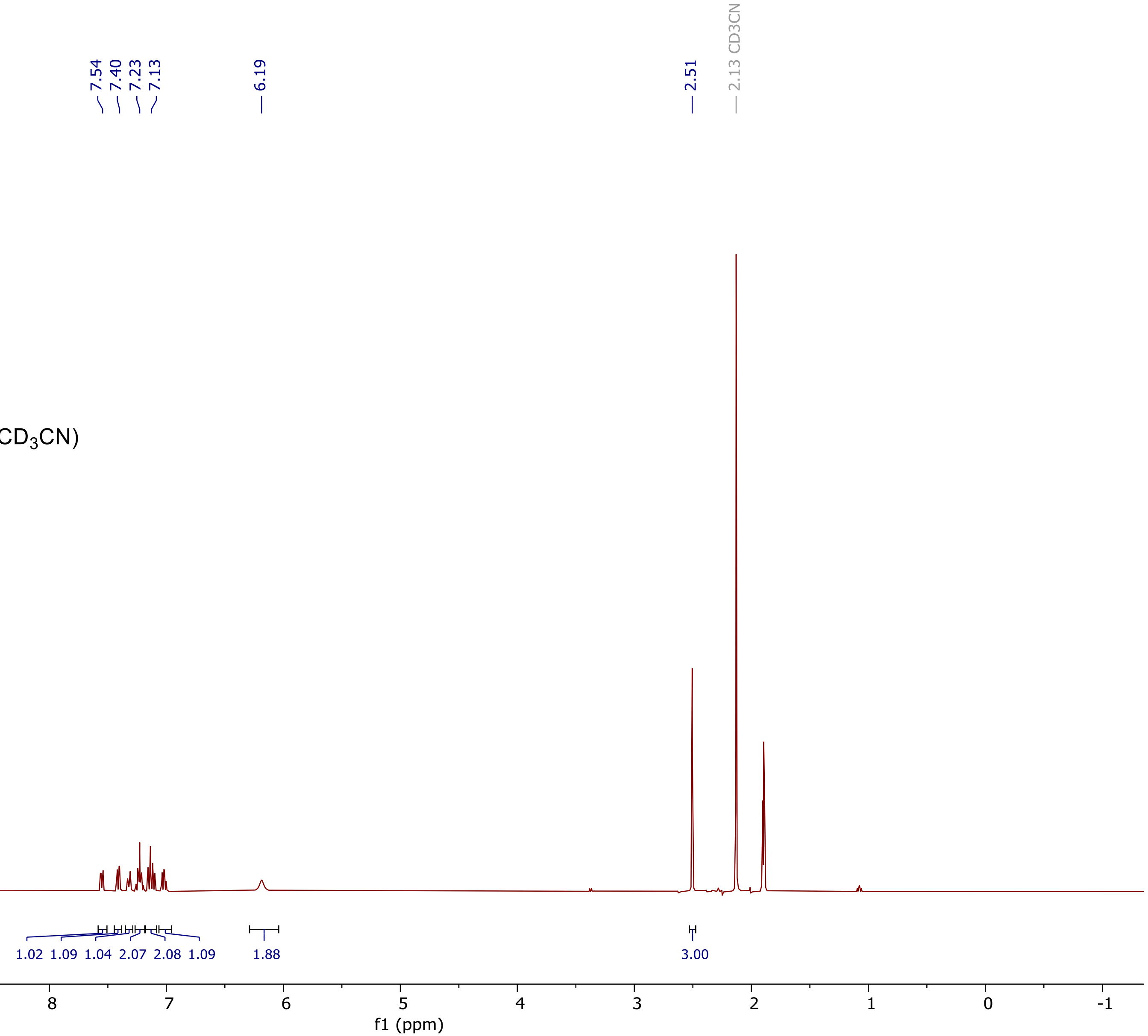

Supplement: SC-016-D5SC07577G-s001 [file SC-016-D5SC07577G-s001.zip › si_chemsci_mazet_revised.pdf]
